# Supplementary material for: DYRK4 upregulates antiviral innate immunity by promoting IRF3 activation
Source: EMBO Rep. 2024 Dec 19;26(3):690–719. doi: 10.1038/s44319-024-00352-x (PMC11811199; doi:10.1038/s44319-024-00352-x)
Supplement: Supplementary file 1 — Appendix [file 44319_2024_352_MOESM1_ESM.pdf]

DYRK4 upregulates antiviral innate immunity by promoting IRF3 activation

Xianhuang Zeng[1], Jiaqi Xu[1], Jiaqi Liu[2], Yang Liu[1], Siqi Yang[1], Junsong Huang[2], Chengpeng Fan[1], Mingxiong Guo[2,3]*, Guihong Sun[1,4]*

1 Taikang Medical School (School of Basic Medical Sciences), Wuhan University, Wuhan 430071, China;

2 Hubei Key Laboratory of Cell Homeostasis, College of Life Sciences, Wuhan University, Wuhan 430072, China;

3 School of Ecology and Environment, Tibet University, Lhasa 850000, Xizang, China;

4 Hubei Provincial Key Laboratory of Allergy and Immunology, Wuhan 430071, China.

* Correspondence: guomx@whu.edu.cn; ghsunlab@whu.edu.cn

Content	Page number
Appendix Table S1	2
Appendix Table S2	3
Appendix Table S3	4

Appendix Table S1 Primers for sgRNA

Gene	Species	Forward primer (5' - 3')	Reverse primer (5' - 3')
DYRK4-sgRNA#1	Human	CACCGGATCCTGGGTTTTAATGCT	AAACAGCATTAAAACCCAGGATCC
DYRK4-sgRNA#2	Human	CACCGCATTAAAACCCAGGATCCCA	AAACTGGGATCCTGGGTTTTAATGC
TRIM71-sgRNA	Human	CACCGCTCGCAGACGTCCACGTCGT	AAACACGACGTGGACGTCTGCGAG

Appendix Table S2 Primers for qRT-PCR

Gene	Forward	Reverse
GAPDH	ATGACATCAAGAAGGTGGTG	CATACCAGGAAATGAGCTTG
DYRK4	TCCACCCTAGCATTAAAACCCA	CGTAGCCCAGGATTTCACTTT
IFNB1	AGGACAGGATGAACTTTGAC	TGATAGACATTAGCCAGGAG
ISG15	GAGAGGCAGCGAACTCATCTT	CCAGCATCTTCACCGTCAGG
CXCL10	GCTCTACTGAGGTGCTATGTTC	GGAGGATGGCAGTGGAAGTC
IL-6	ACTCACCTCTTCAGAACGAATTG	CCATCTTTGGAAGGTTCAGGTTG
IRF3	AGAGGCTCGTGATGGTCAAG	AGGTCCACAGTATTCTCCAGG
IRF1	ATGCCCATCACTCGGATGC	CCCTGCTTTGTATCGGCCTG
TRIM71	GTACCCATCTGTCGTGAGTGC	CGGCTTTGACCTCCGACTG
Gapdh	ATGGTGAAGGTCGGTGTGAA	CGCTCCTGGAAGATGGTGAT
Ifnb1	CCGAGCAGAGATCTTCAGGAA	CCTGCAACCACCACTCATTCT
Isg15	CCTCTGAGCATCCTGGTGAG	ACTGGTCTTCGTGGACTTGTT
Cxcl10	TCAGGCTCGTCAGTTCTAAGTT	GATGGTGGTTAAGTTCGTGCTT
Il6	TTCCATCCAGTTGCCTTCTTG	AATTAAGCCTCCGACTTGTGAA
Dyrk4	GCAACAAAGTCCCATCAAAGG	GTCTTGGGCTTTGGTGTTAATG
VSV	TGATAGTACCGGAGGATTGACGAC	CCTTGCAGTGACATGACTGCTCTT

Appendix Table S3 Primers for shRNAs

shDYRK4#1F	Forward
CCGGACTGGTAGACGCTCCCAAGAACTCGAGTTCTTGGGAGCGTCTACCAGT TTTTTG	
shDYRK4#1R	Reverse
AATTCAAAAAACTGGTAGACGCTCCCAAGAACTCGAGTTCTTGGGAGCGTCTACCAGT	
shDYRK4#2F	Forward
CCGG CCAGAAAGTATACACGTACAT CTCGAG ATGTACGTGTATACTTTCTGG TTTTTG	
shDYRK4#2R	Reverse
AATTCAAAAA CCAGAAAGTATACACGTACAT CTCGAG ATGTACGTGTATACTTTCTGG	
shTRIM71#1F	Forward
CCGGGCAGCTTCCTGTGCAAGTTTGCTCGAGCAAACTTGCACAGGAAGCTGC TTTTTG	
shTRIM71#1R	Reverse
AATTCAAAAAGCAGCTTCCTGTGCAAGTTTGCTCGAGCAAACTTGCACAGGAAGCTGC	
shTRIM71#2F	Forward
CCGG CCTCGTCTTCTAATTGCATTT CTCGAG AAATGCAATTAGAAGACGAGG TTTTTG	
shTRIM71#2R	Reverse
AATTCAAAAA CCTCGTCTTCTAATTGCATTT CTCGAG AAATGCAATTAGAAGACGAGG	
shTRIM71#3F	Forward
CCGG TGAGCCTGTGAAGTGATAATT CTCGAG AATTATCACTTCACAGGCTCA TTTTTG	
shTRIM71#3R	Reverse
AATTCAAAAA TGAGCCTGTGAAGTGATAATT CTCGAG AATTATCACTTCACAGGCTCA	
