# Supplementary figures and images for: DYRK4 upregulates antiviral innate immunity by promoting IRF3 activation (part 1 of 3)
Source: EMBO Rep. 2024 Dec 19;26(3):690–719. doi: 10.1038/s44319-024-00352-x (PMC11811199; doi:10.1038/s44319-024-00352-x)

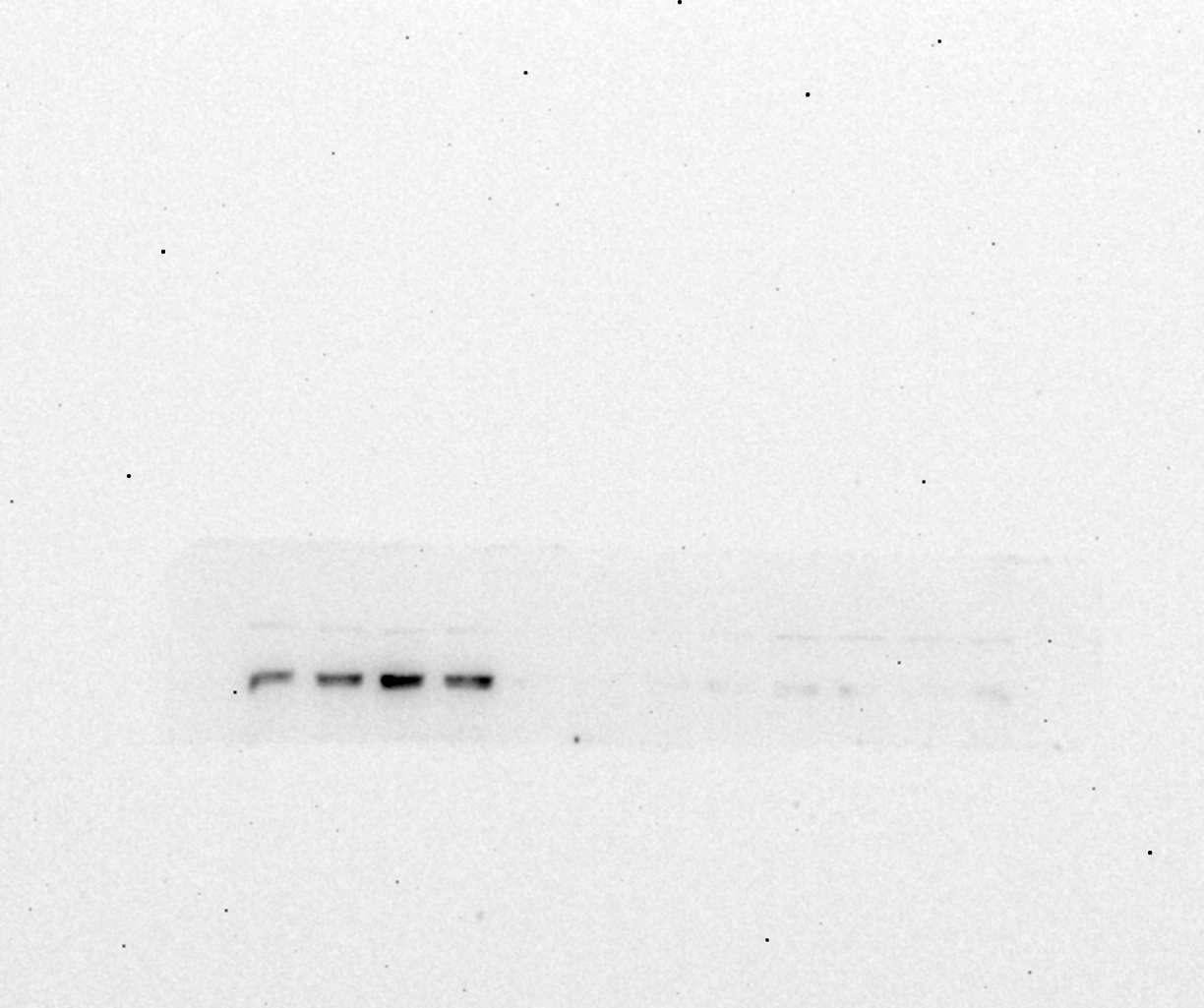

Supplement: Supplementary file 4 — Source data Fig. 1 [file 44319_2024_352_MOESM4_ESM.zip › Figure 1/1E/DYRK4 chunqian 1-80000 293T-WT 6-18 2-9+SEV 0 4 8 12h -2.tif]

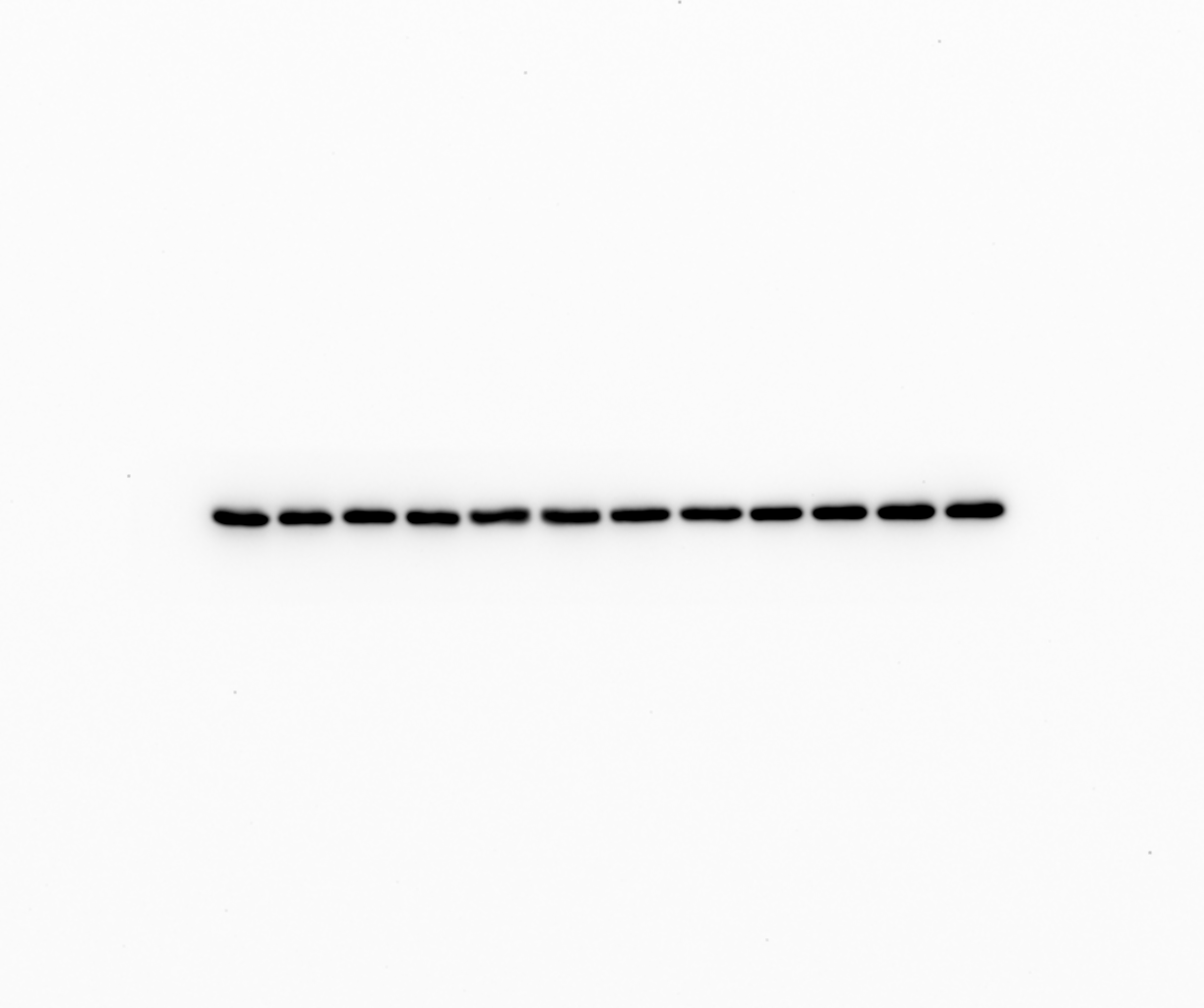

Supplement: Supplementary file 4 — Source data Fig. 1 [file 44319_2024_352_MOESM4_ESM.zip › Figure 1/1E/GAPDH 293T-WT 6-18 2-9+SeV-0 4 8 12h -5.tif]

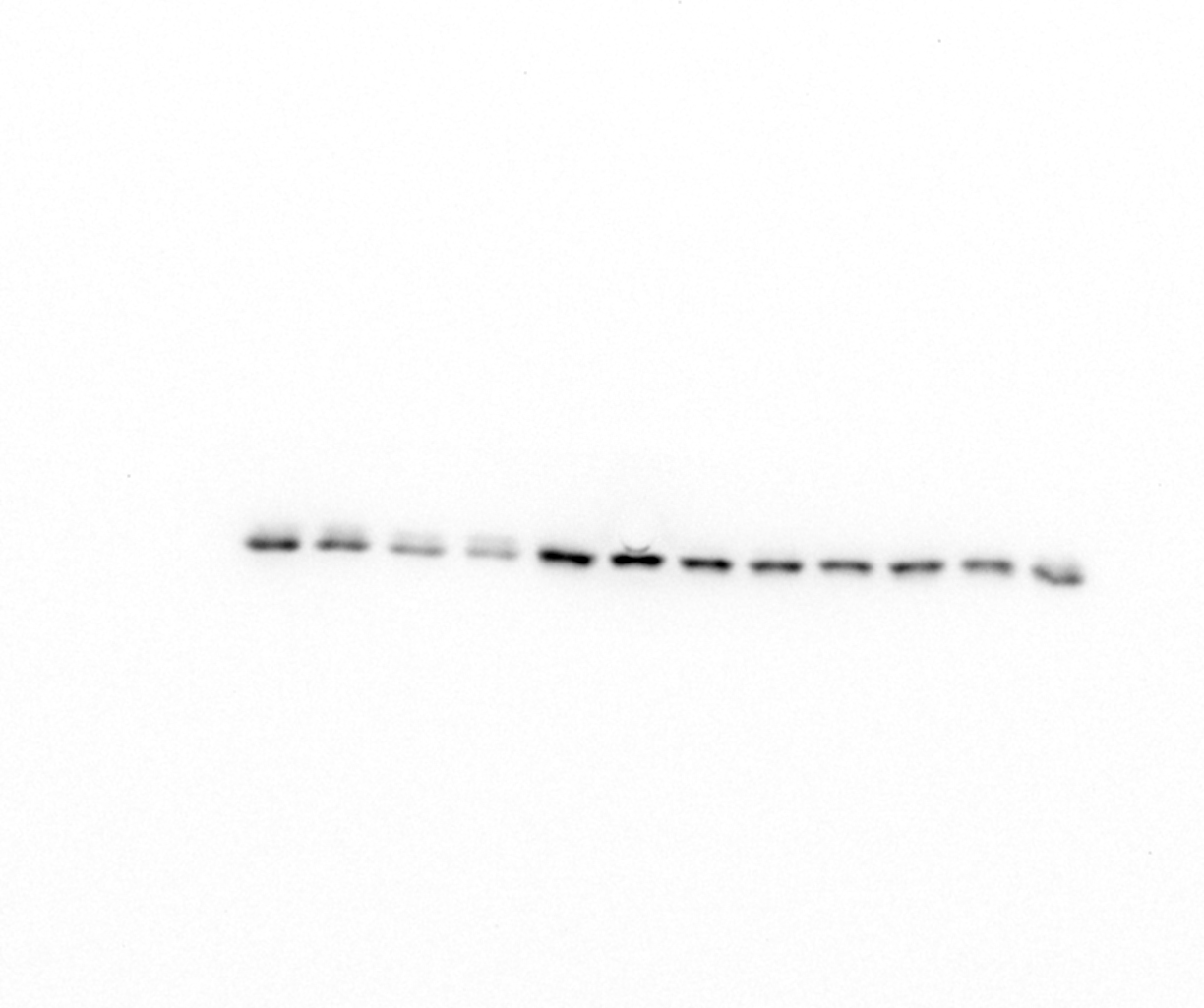

Supplement: Supplementary file 4 — Source data Fig. 1 [file 44319_2024_352_MOESM4_ESM.zip › Figure 1/1E/IKBa 293T-WT 2-9 6-18+SEV-0-4-8-12h -14.tif]

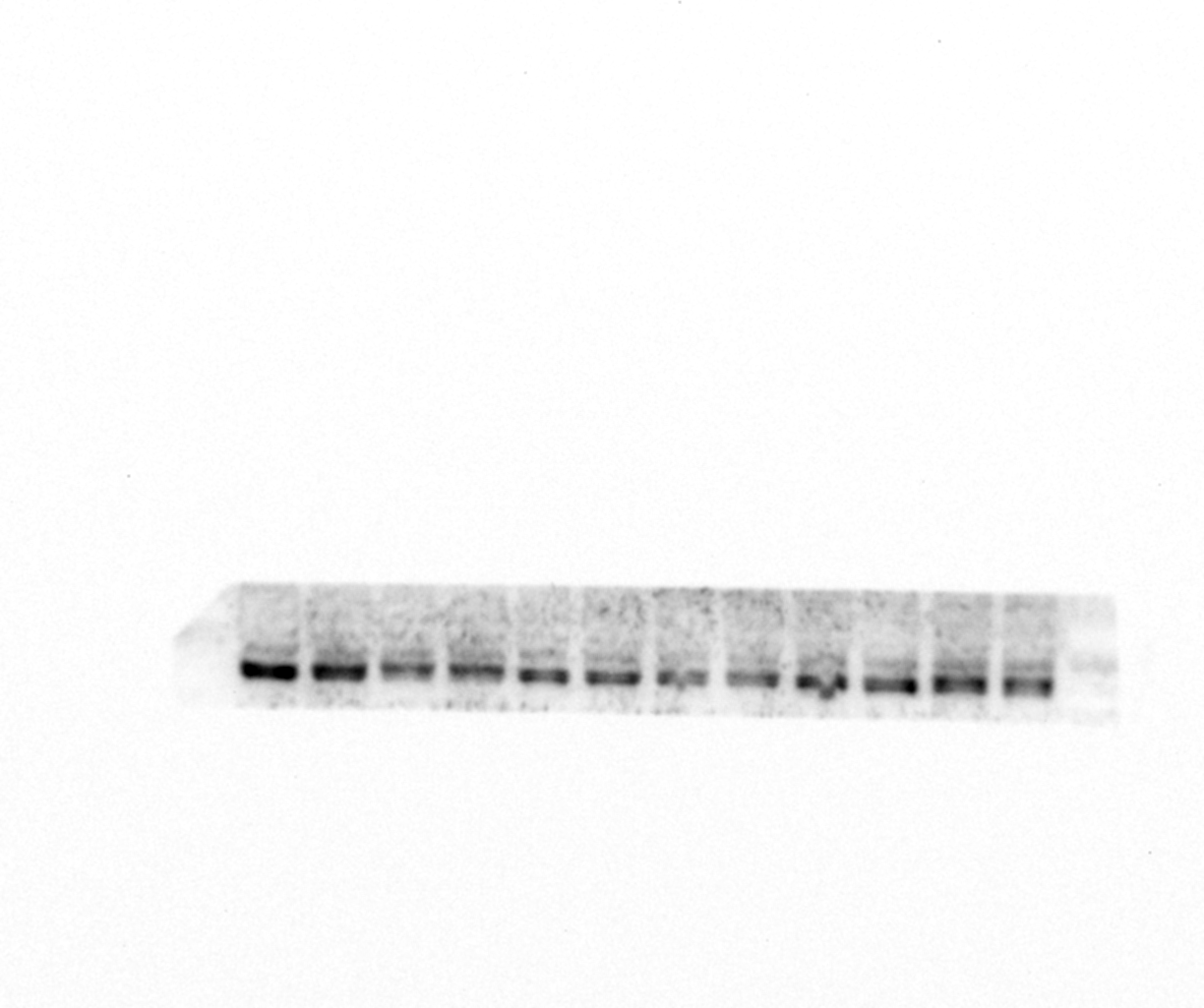

Supplement: Supplementary file 4 — Source data Fig. 1 [file 44319_2024_352_MOESM4_ESM.zip › Figure 1/1E/IRF3 293T-WT 2-9 6-18+SeV-0-4-8-12h -14.tif]

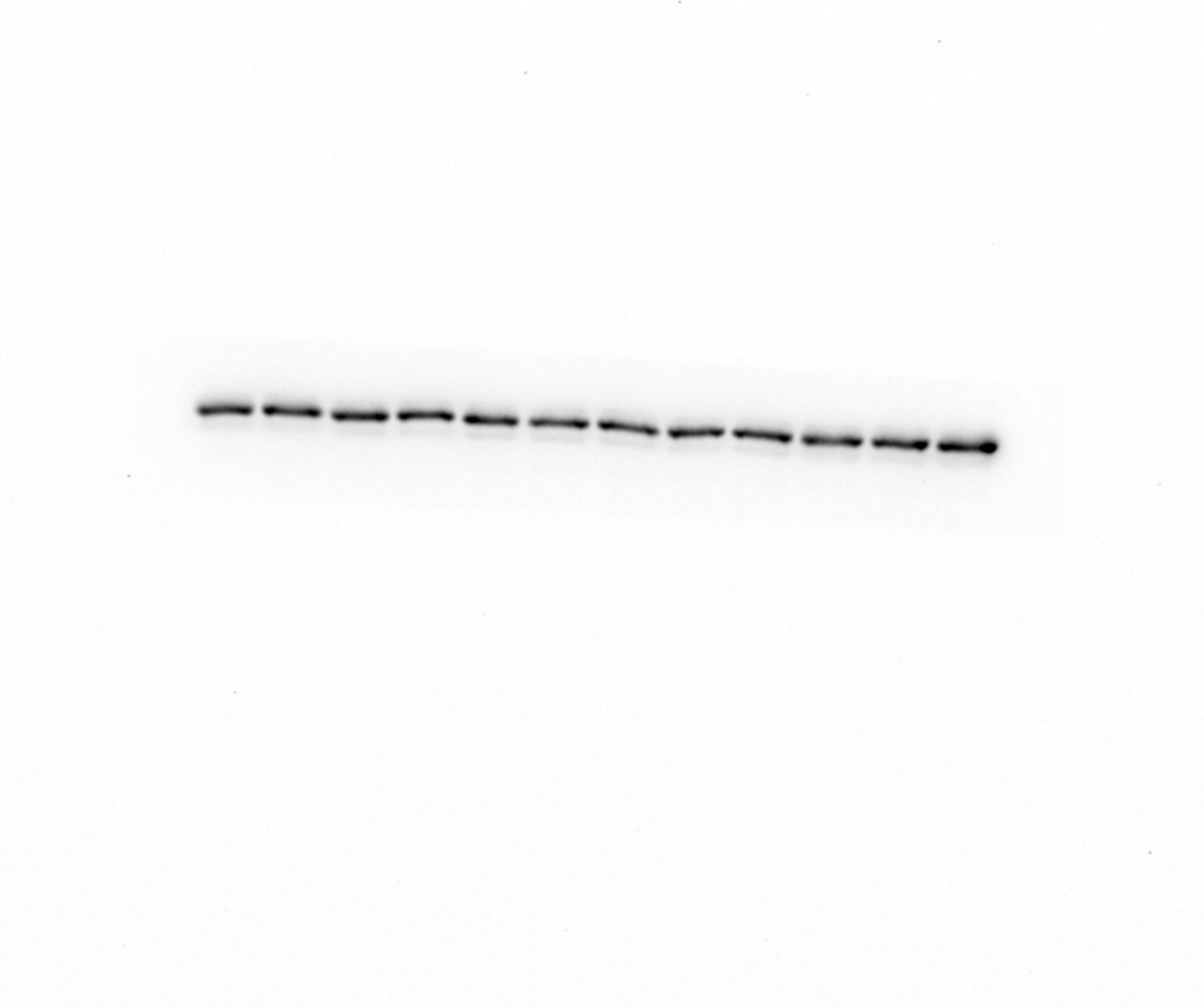

Supplement: Supplementary file 4 — Source data Fig. 1 [file 44319_2024_352_MOESM4_ESM.zip › Figure 1/1E/P65 293T-WT 2-9 6-18+SeV-0-4-8-12h -10.tif]

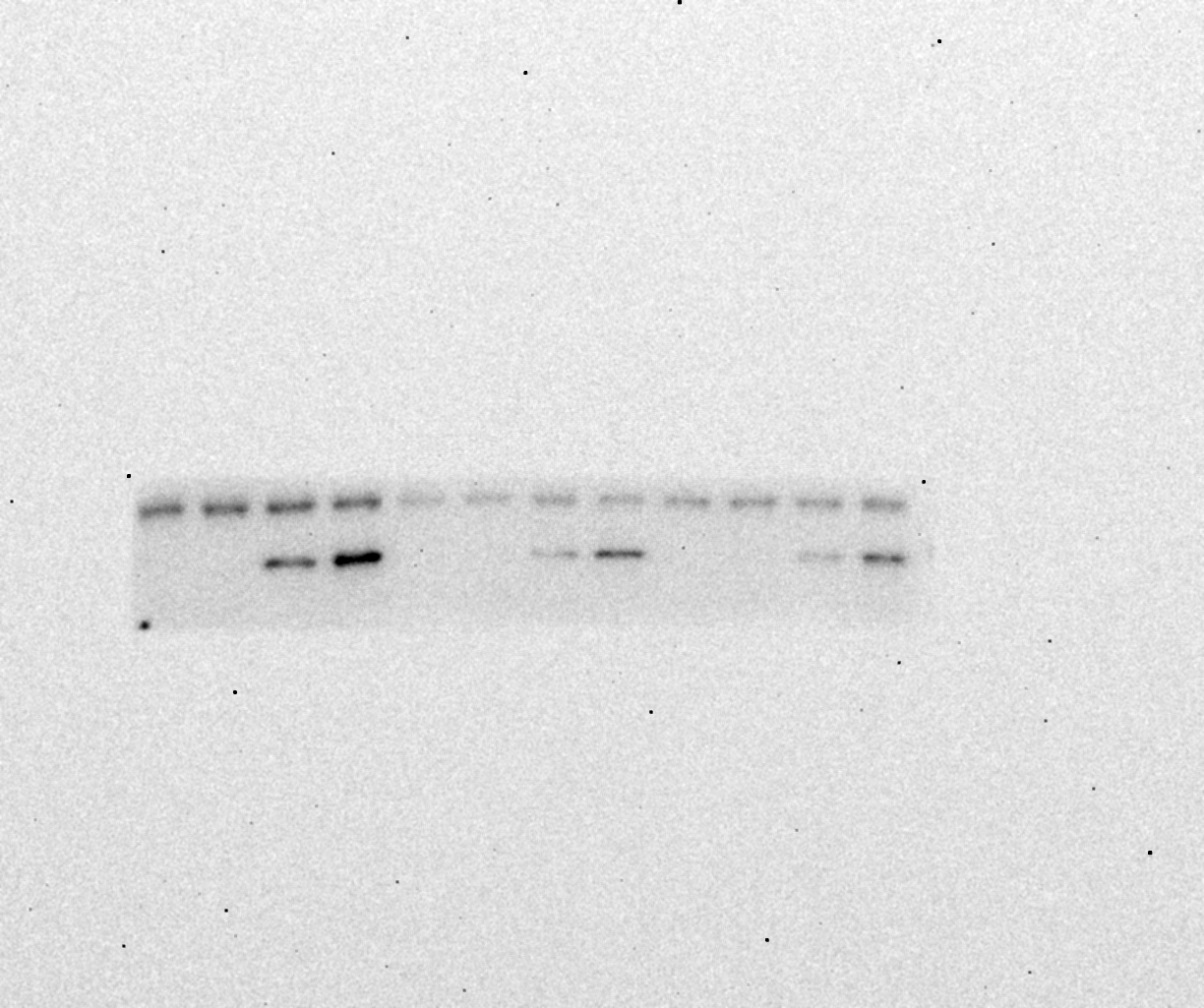

Supplement: Supplementary file 4 — Source data Fig. 1 [file 44319_2024_352_MOESM4_ESM.zip › Figure 1/1E/p-IKBa 293T-WT 6-18 2-9+SEV-0 4 8 12h -1.tif]

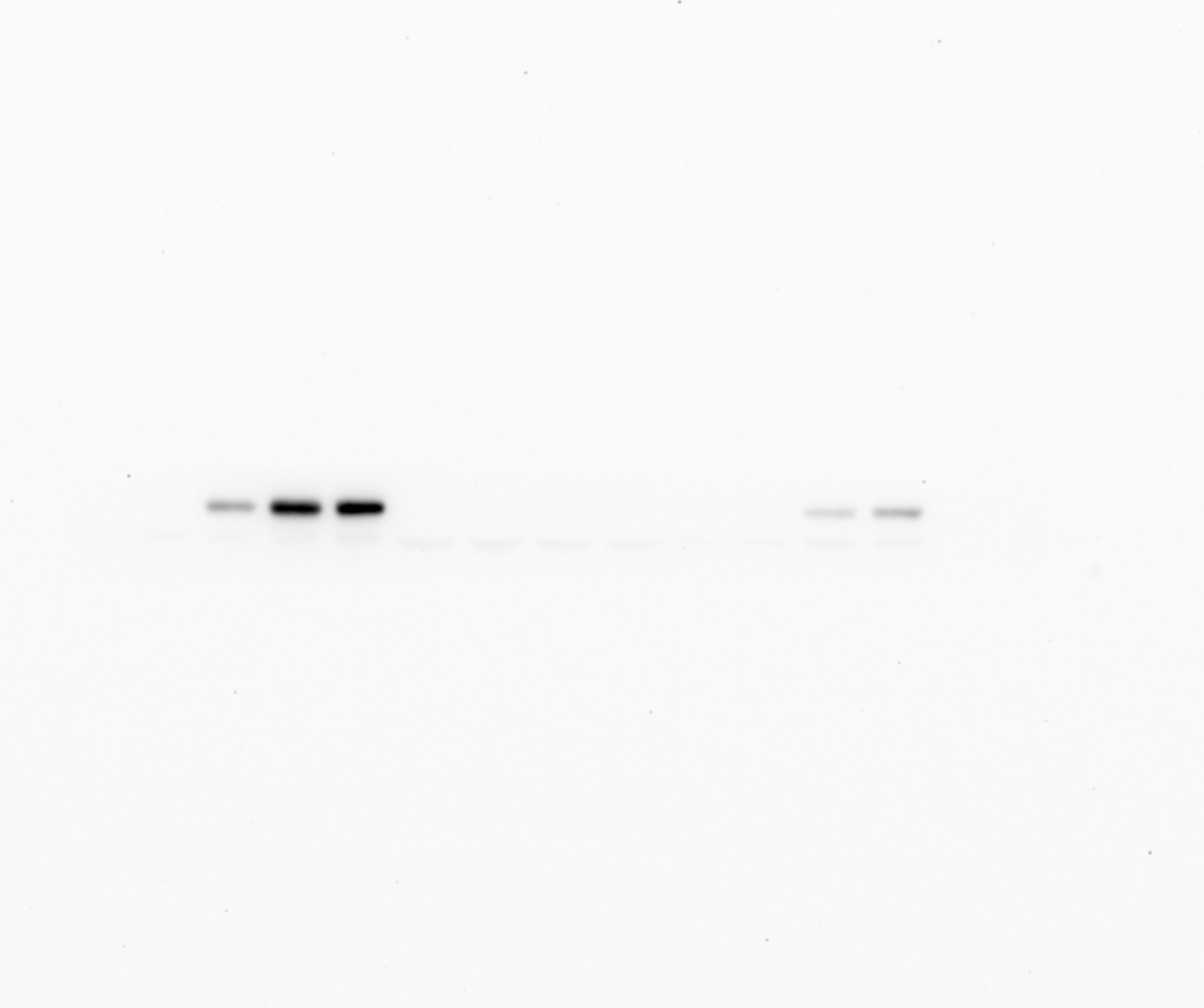

Supplement: Supplementary file 4 — Source data Fig. 1 [file 44319_2024_352_MOESM4_ESM.zip › Figure 1/1E/p-IRF3-S386 293T-WT 6-18 2-9+SeV-0 4 8 12h -3.tif]

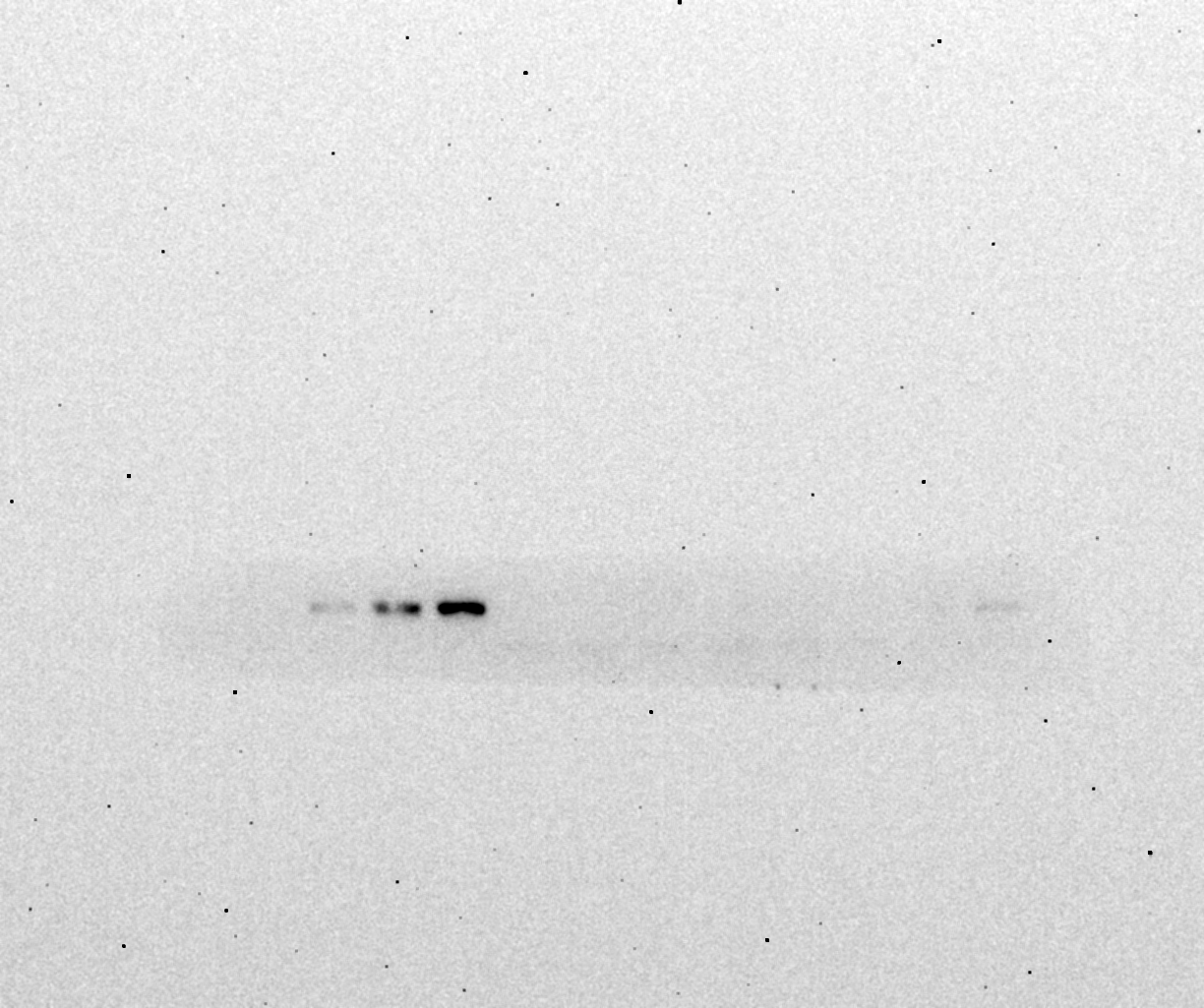

Supplement: Supplementary file 4 — Source data Fig. 1 [file 44319_2024_352_MOESM4_ESM.zip › Figure 1/1E/p-IRF3-S396 293T-WT 6-18 2-9+SeV-0 4 8 12h -3.tif]

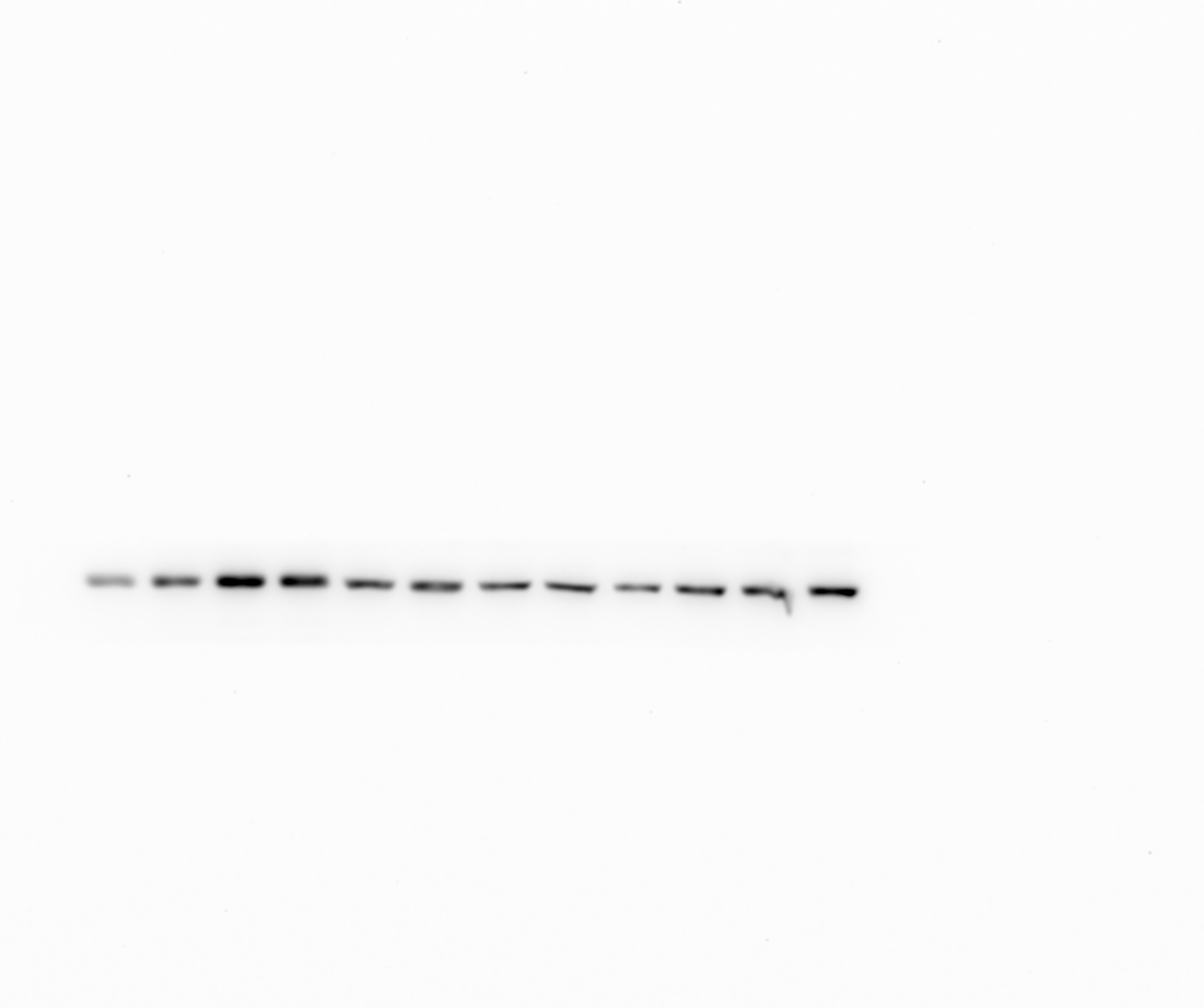

Supplement: Supplementary file 4 — Source data Fig. 1 [file 44319_2024_352_MOESM4_ESM.zip › Figure 1/1E/p-P65 293T-WT 6-18 2-9+SeV-0 4 8 12h -2.tif]

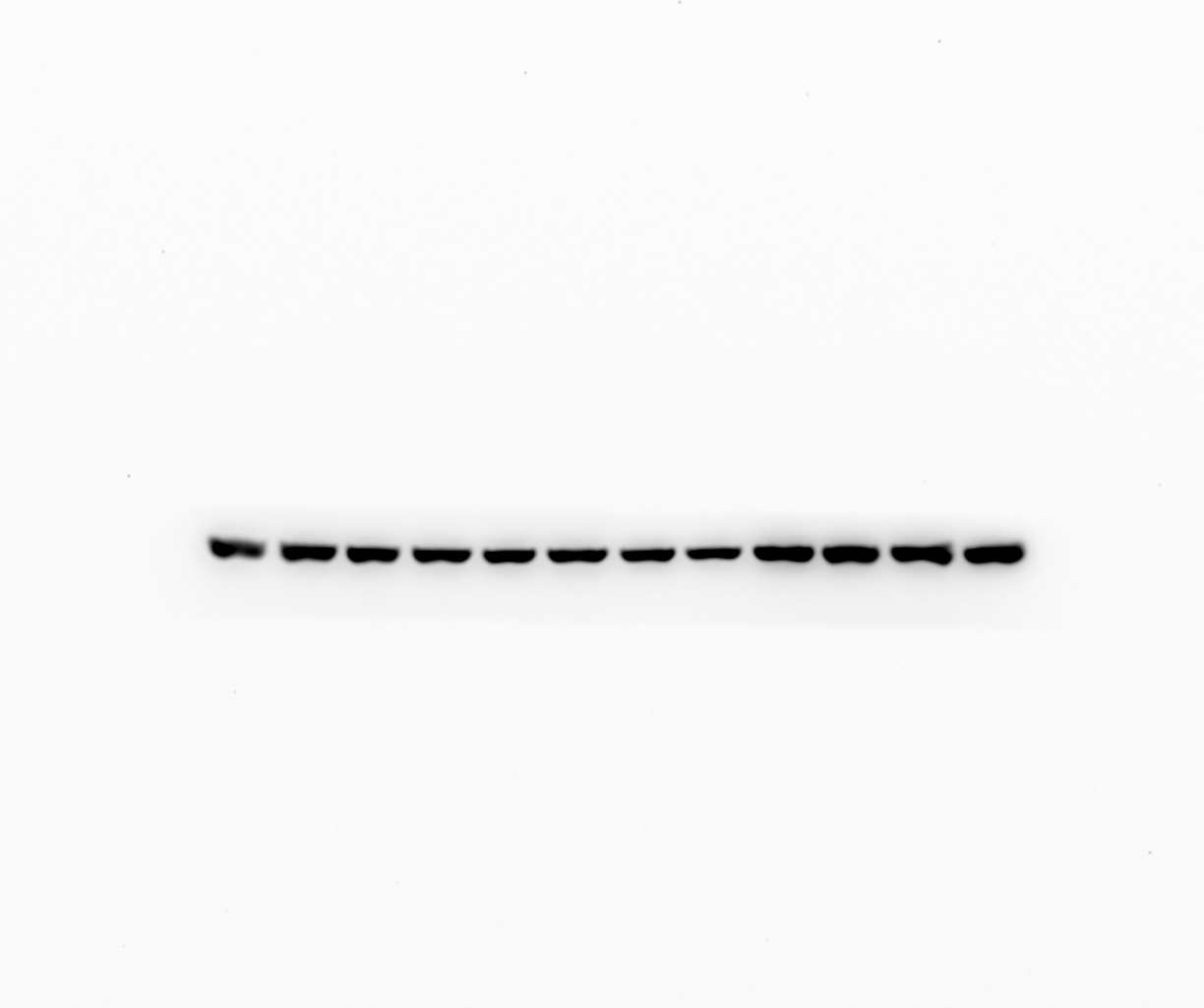

Supplement: Supplementary file 4 — Source data Fig. 1 [file 44319_2024_352_MOESM4_ESM.zip › Figure 1/1F/GAPDH A549-WT A46 A40+HSV-1 -0-4-8-12h -4.tif]

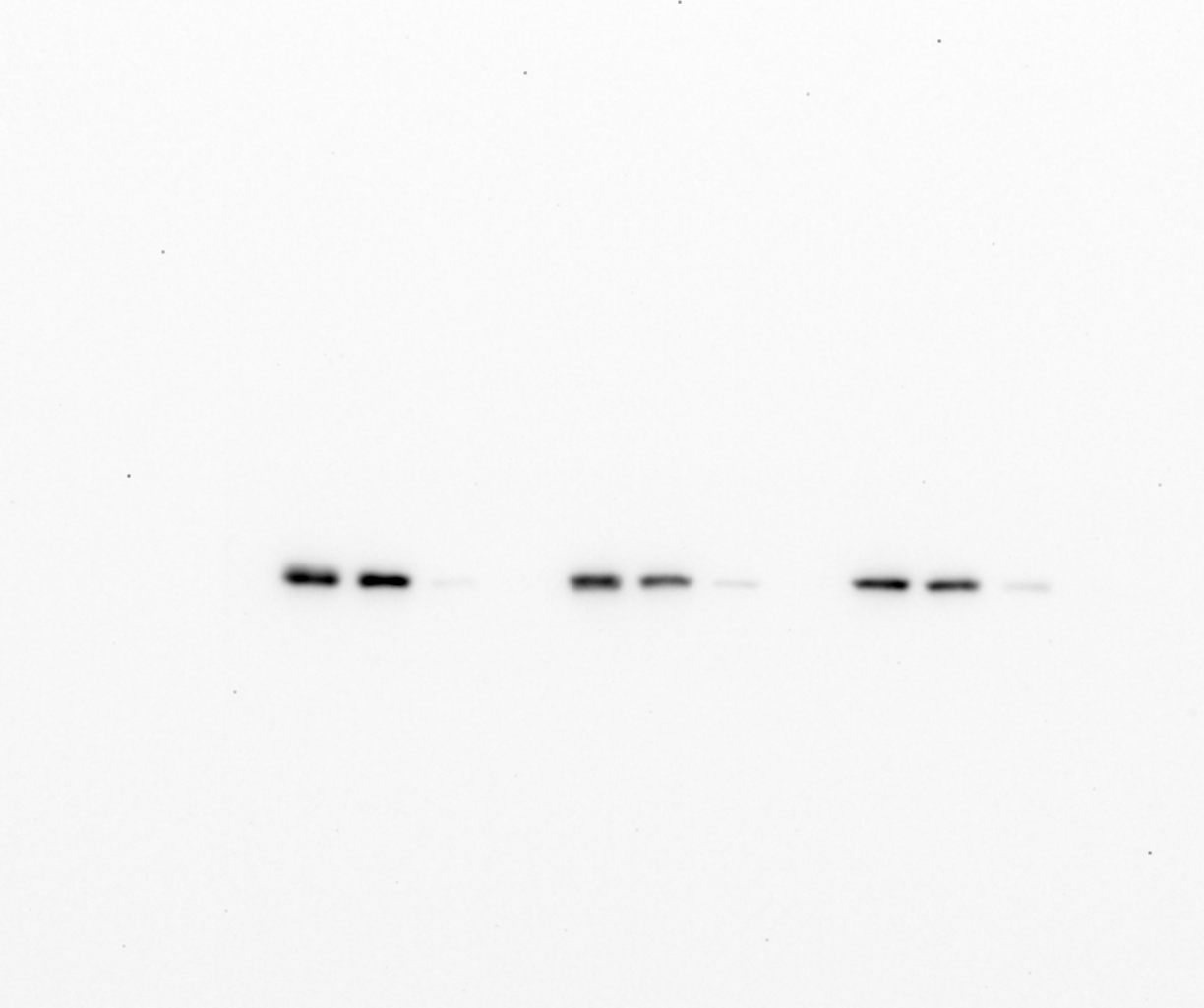

Supplement: Supplementary file 4 — Source data Fig. 1 [file 44319_2024_352_MOESM4_ESM.zip › Figure 1/1F/IKBa A549-WT A46 A40+HSV-1 -0-4-8-12h -2.tif]

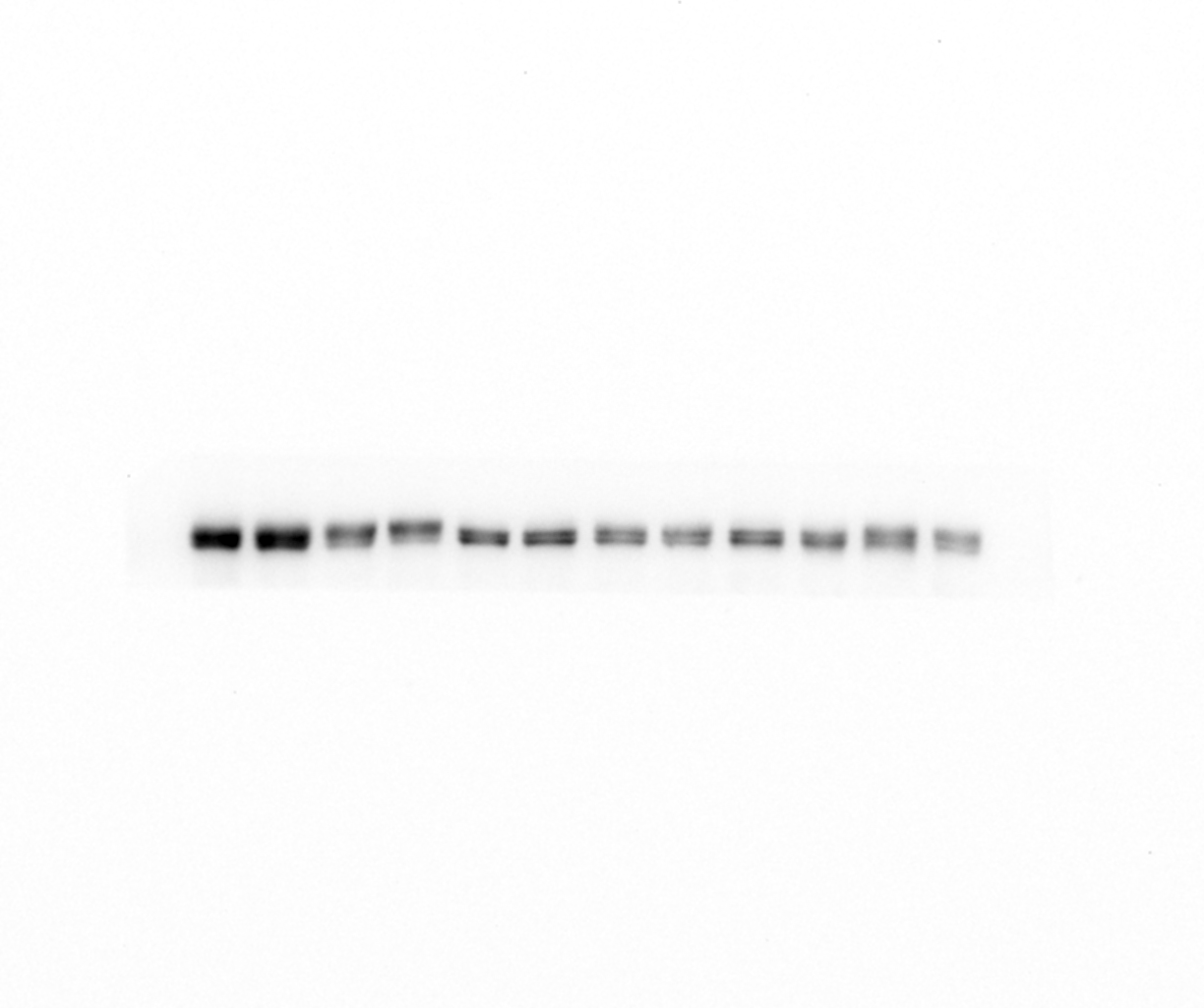

Supplement: Supplementary file 4 — Source data Fig. 1 [file 44319_2024_352_MOESM4_ESM.zip › Figure 1/1F/IRF3 A549-WT A46 A40+HSV-1 -0-4-8-12h -7.tif]

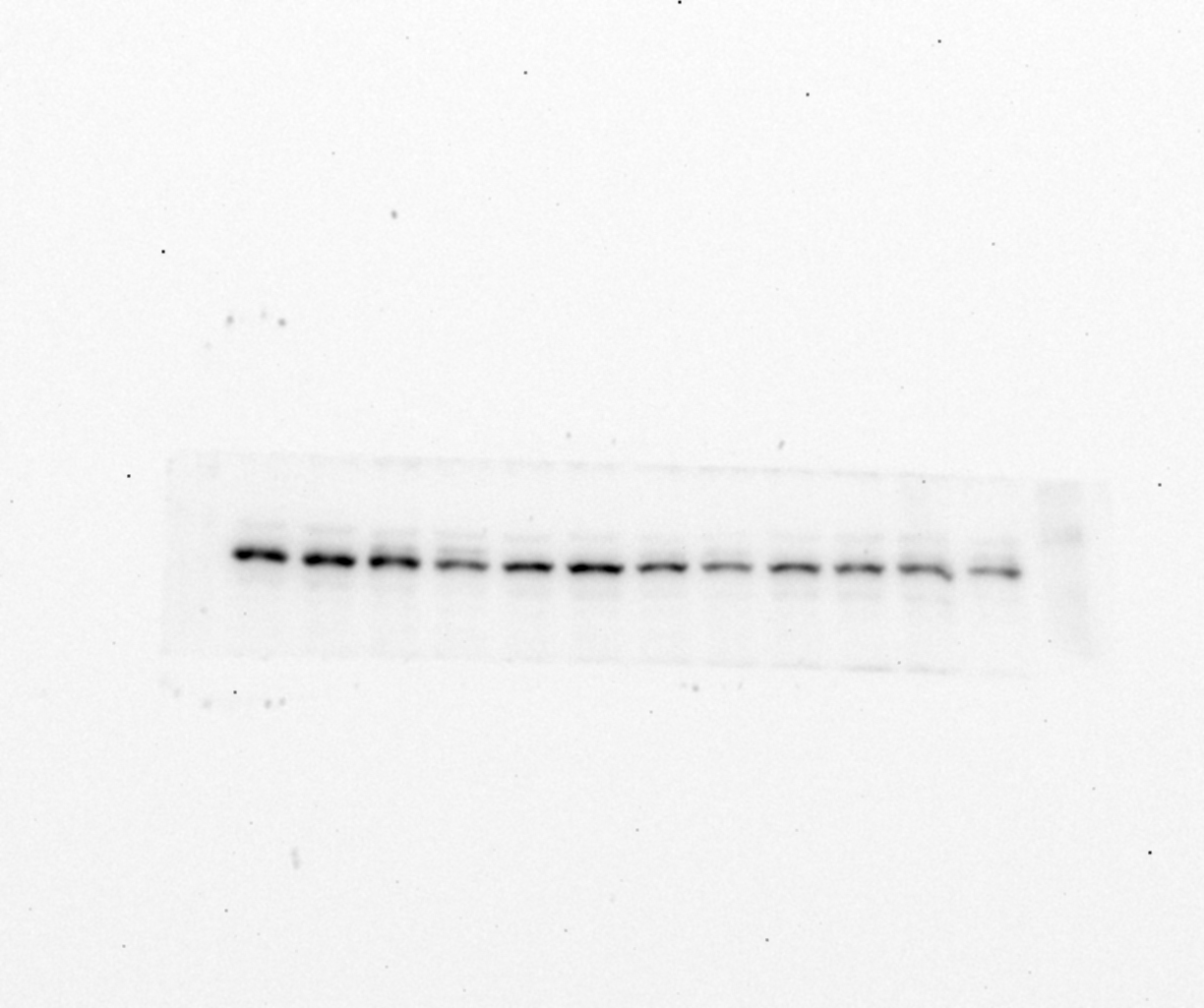

Supplement: Supplementary file 4 — Source data Fig. 1 [file 44319_2024_352_MOESM4_ESM.zip › Figure 1/1F/P65 A549-WT A46 A40+HSV-1 -0-4-8-12h -2.tif]

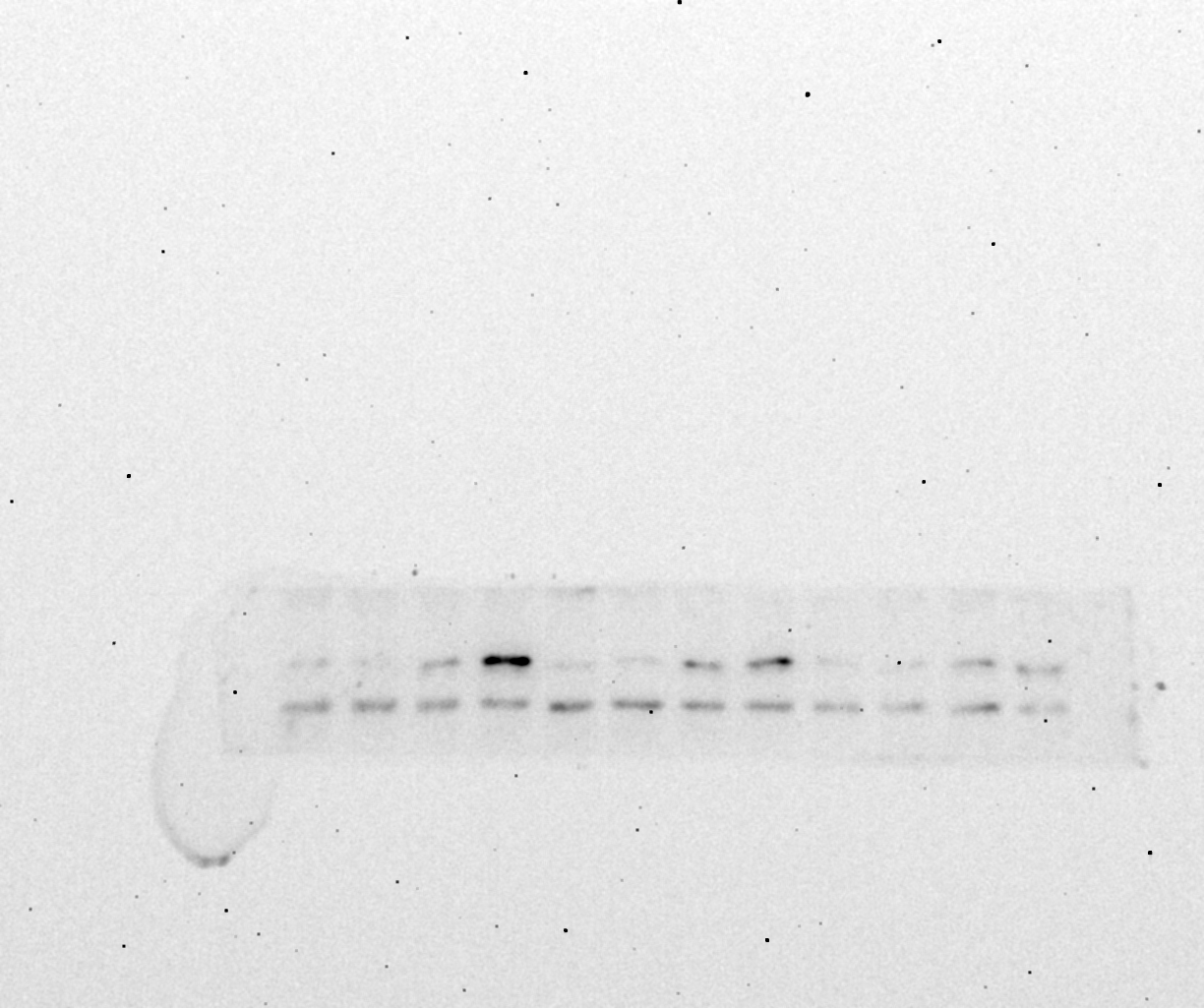

Supplement: Supplementary file 4 — Source data Fig. 1 [file 44319_2024_352_MOESM4_ESM.zip › Figure 1/1F/p-IKBa A549-WT A46 A40+HSV-1 -0-4-8-12h -2.tif]

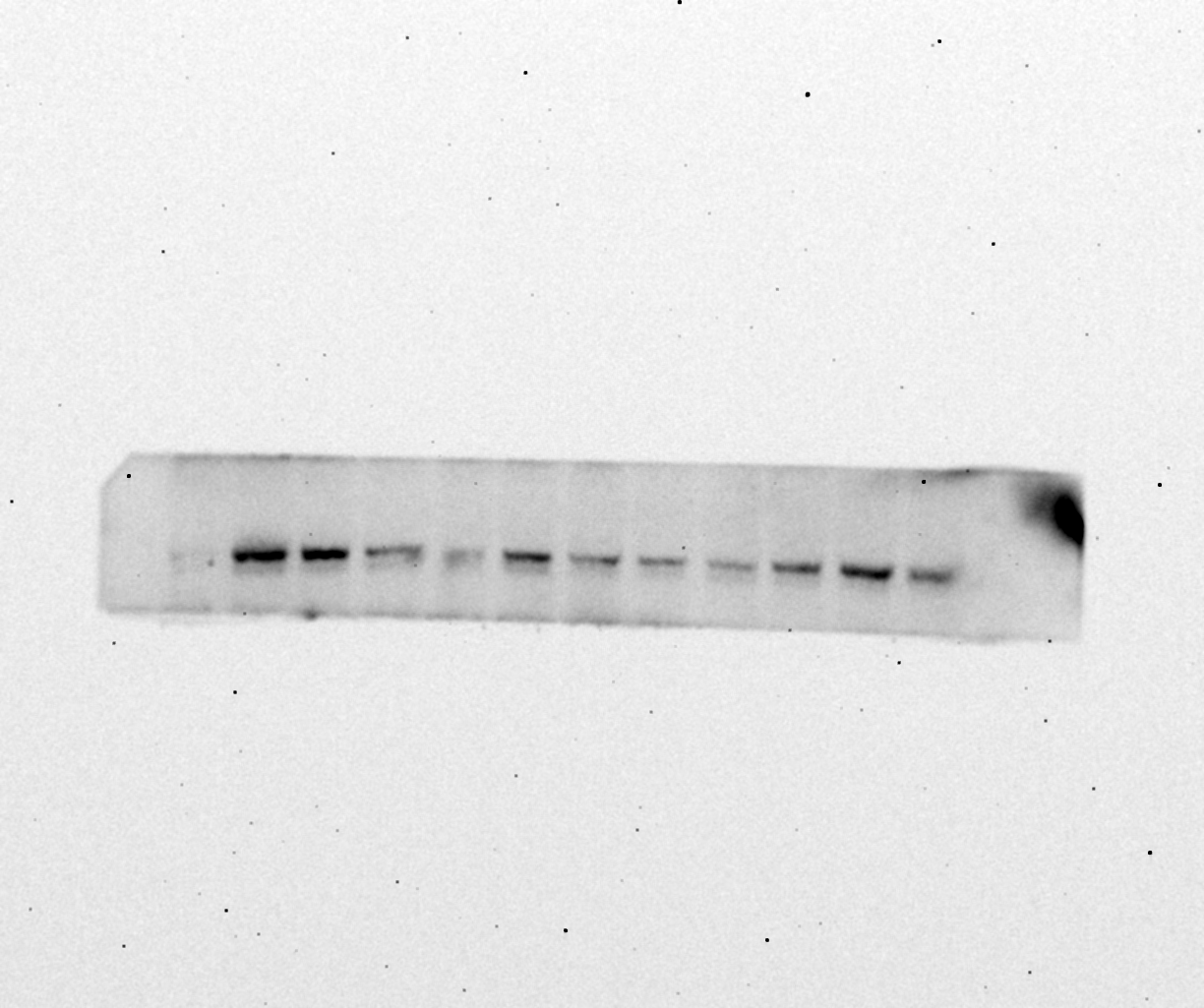

Supplement: Supplementary file 4 — Source data Fig. 1 [file 44319_2024_352_MOESM4_ESM.zip › Figure 1/1F/p-IRF3-S386 A549-WT A46 A40+HSV-1 -0-4-8-12h -8.tif]

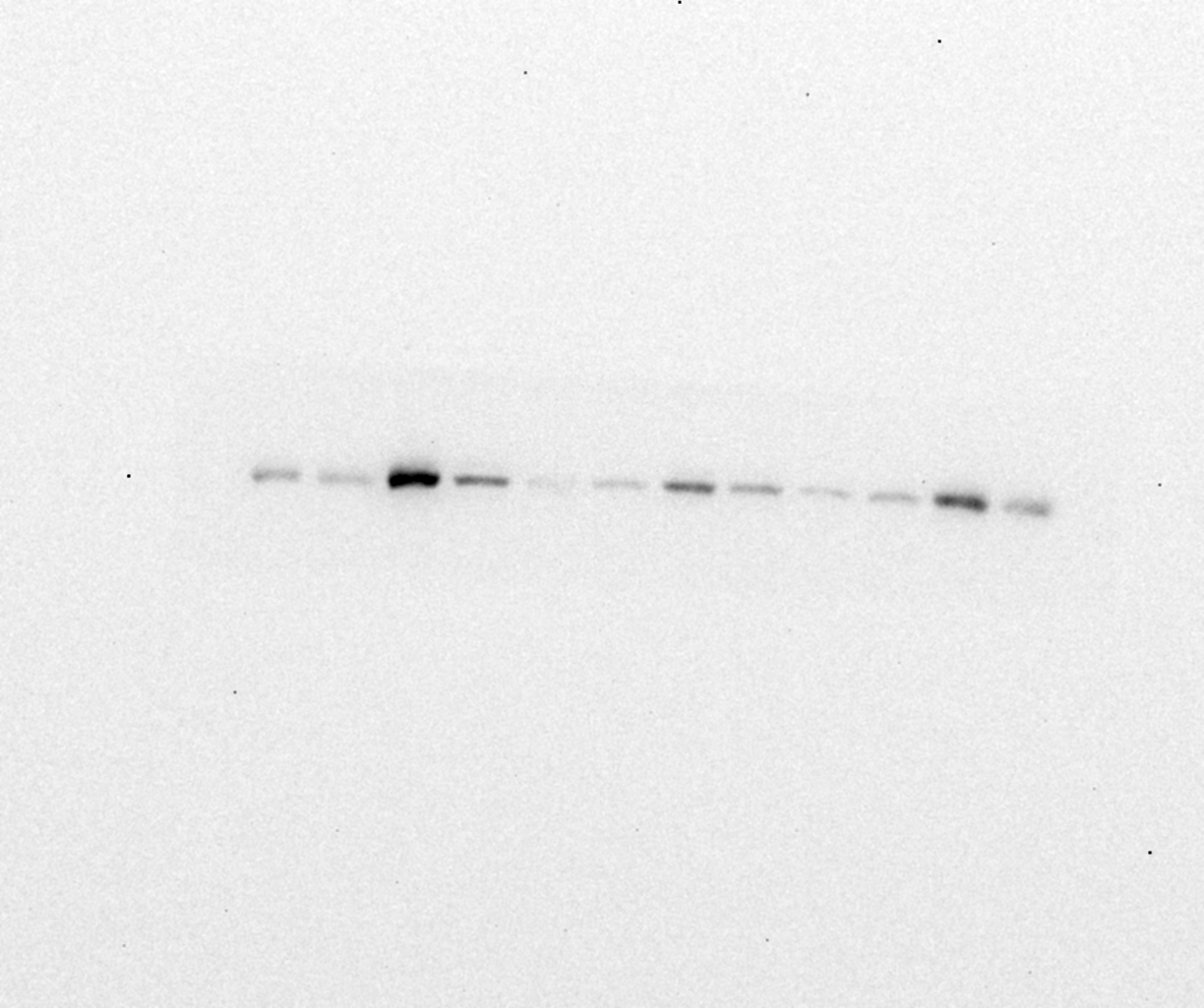

Supplement: Supplementary file 4 — Source data Fig. 1 [file 44319_2024_352_MOESM4_ESM.zip › Figure 1/1F/p-P65 A549-WT A46 A40+HSV-1 -0-4-8-12h -5.tif]

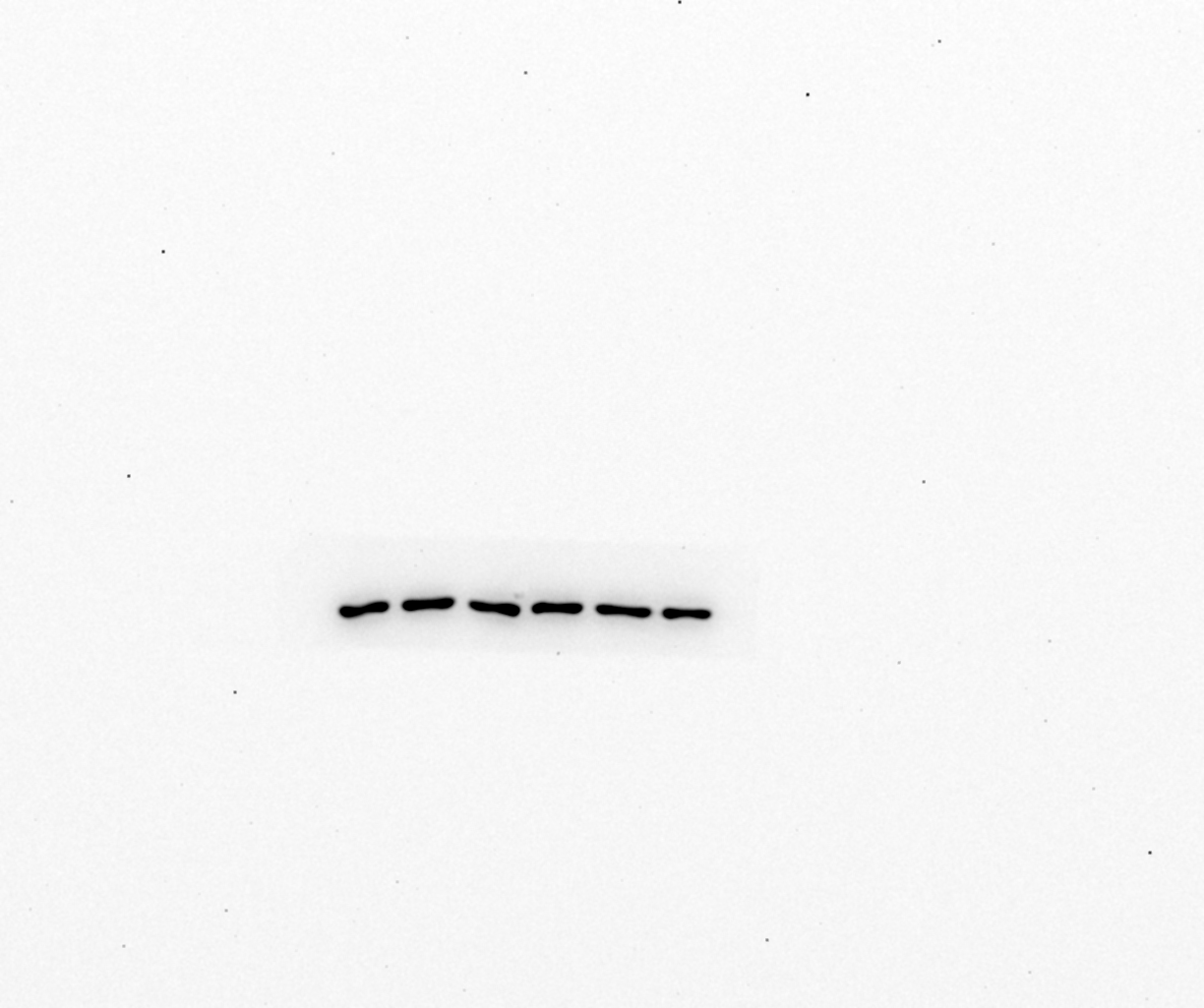

Supplement: Supplementary file 4 — Source data Fig. 1 [file 44319_2024_352_MOESM4_ESM.zip › Figure 1/1G/GAPDH-1-4 293T-WT 6-18 2-9 +mock-SeV IRF3 -4.tif]

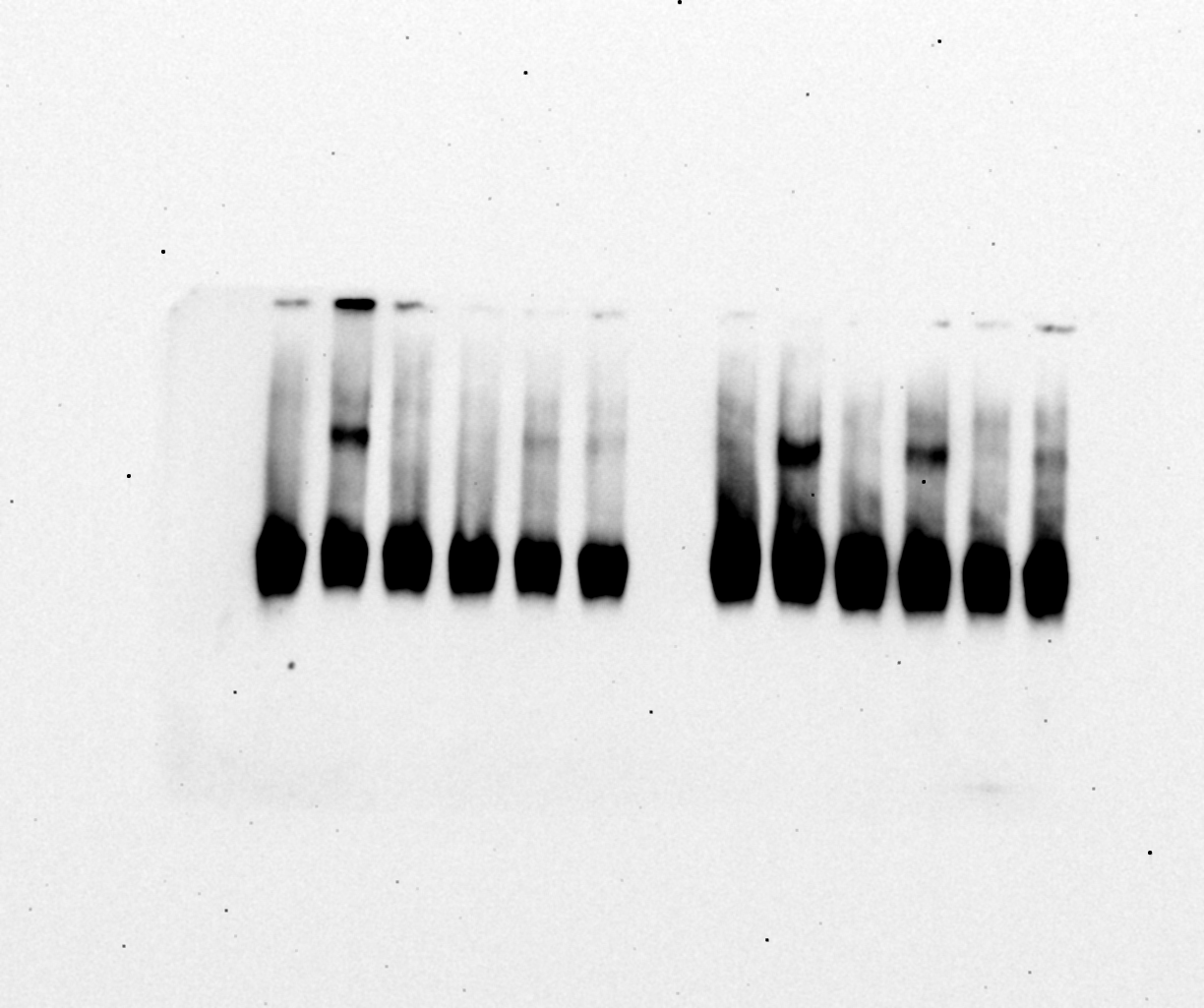

Supplement: Supplementary file 4 — Source data Fig. 1 [file 44319_2024_352_MOESM4_ESM.zip › Figure 1/1G/IRF3 1-12000 293T-WT 6-18 2-9 +mock-SeV IRF3 erjuhua fen -7.tif]

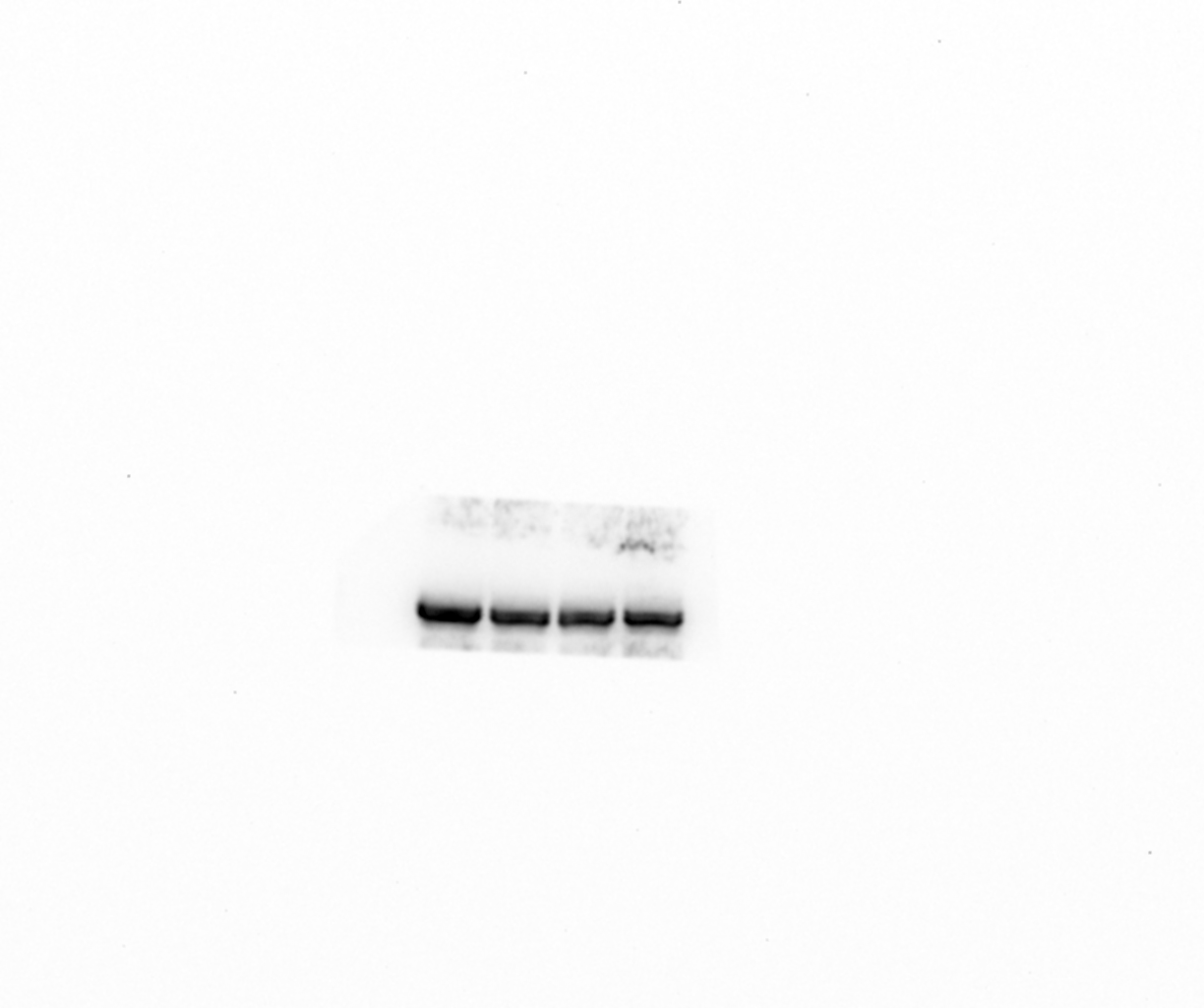

Supplement: Supplementary file 4 — Source data Fig. 1 [file 44319_2024_352_MOESM4_ESM.zip › Figure 1/1G/IRF3 1-12000 293T-WT 6-18 2-9 +mock-SeV IRF3-2.tif]

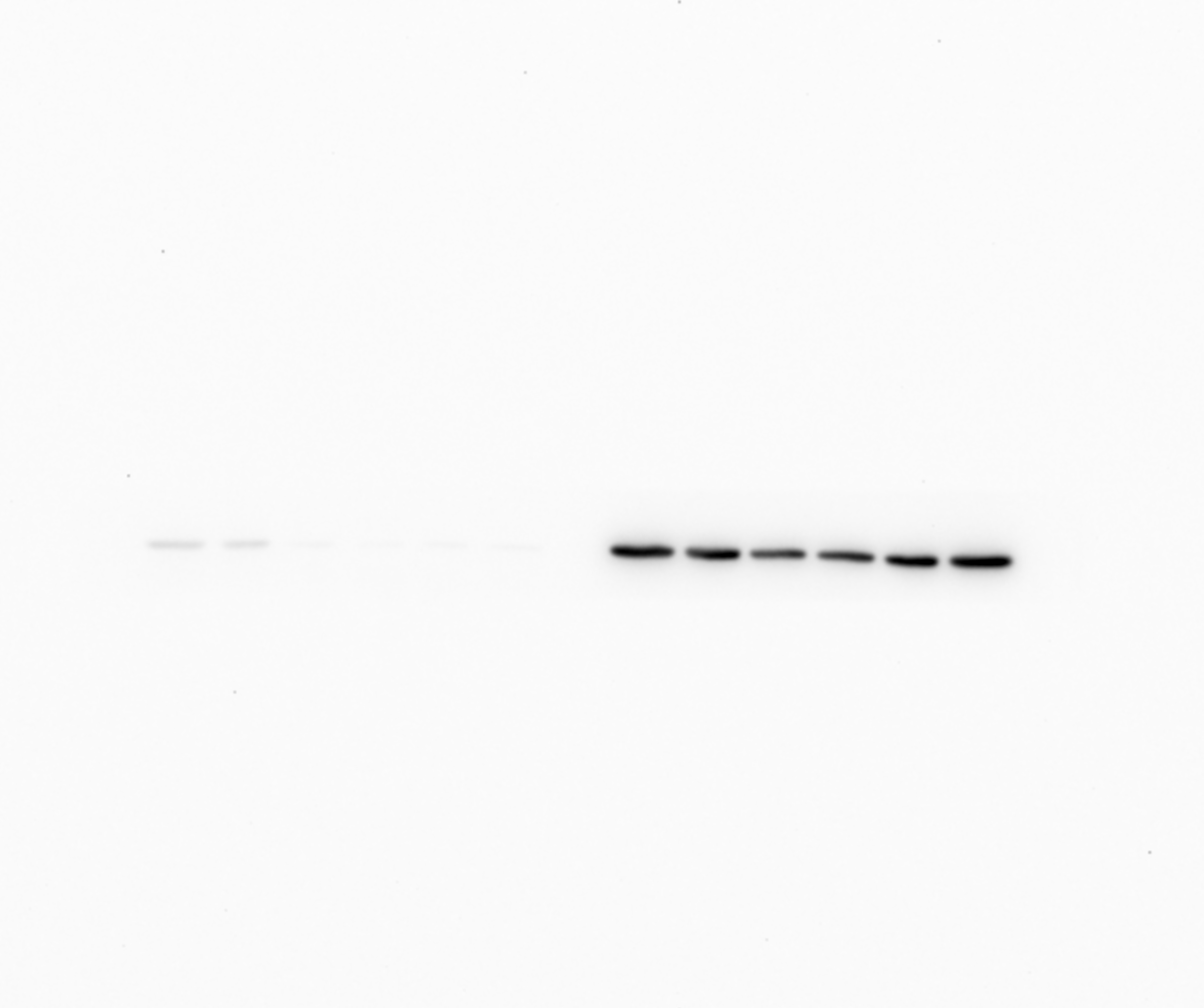

Supplement: Supplementary file 4 — Source data Fig. 1 [file 44319_2024_352_MOESM4_ESM.zip › Figure 1/1H/GAPDH 293T-WT+mock-sev 6-18+mock-sev 2-9+mock-sev he fenli -5.tif]

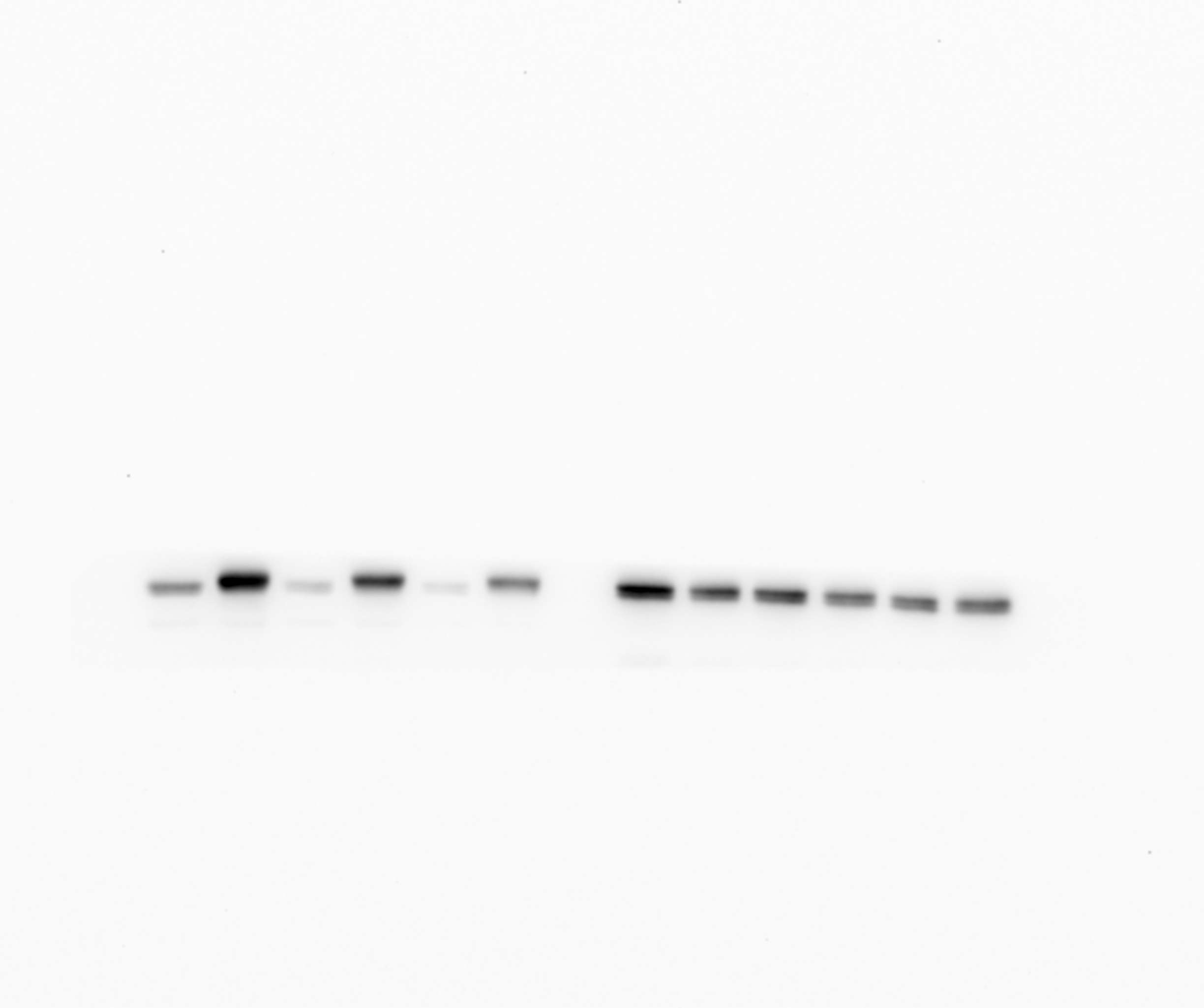

Supplement: Supplementary file 4 — Source data Fig. 1 [file 44319_2024_352_MOESM4_ESM.zip › Figure 1/1H/IRF3 293T-WT+mock-sev 6-18+mock-sev 2-9+mock-sev he fenli -5.tif]

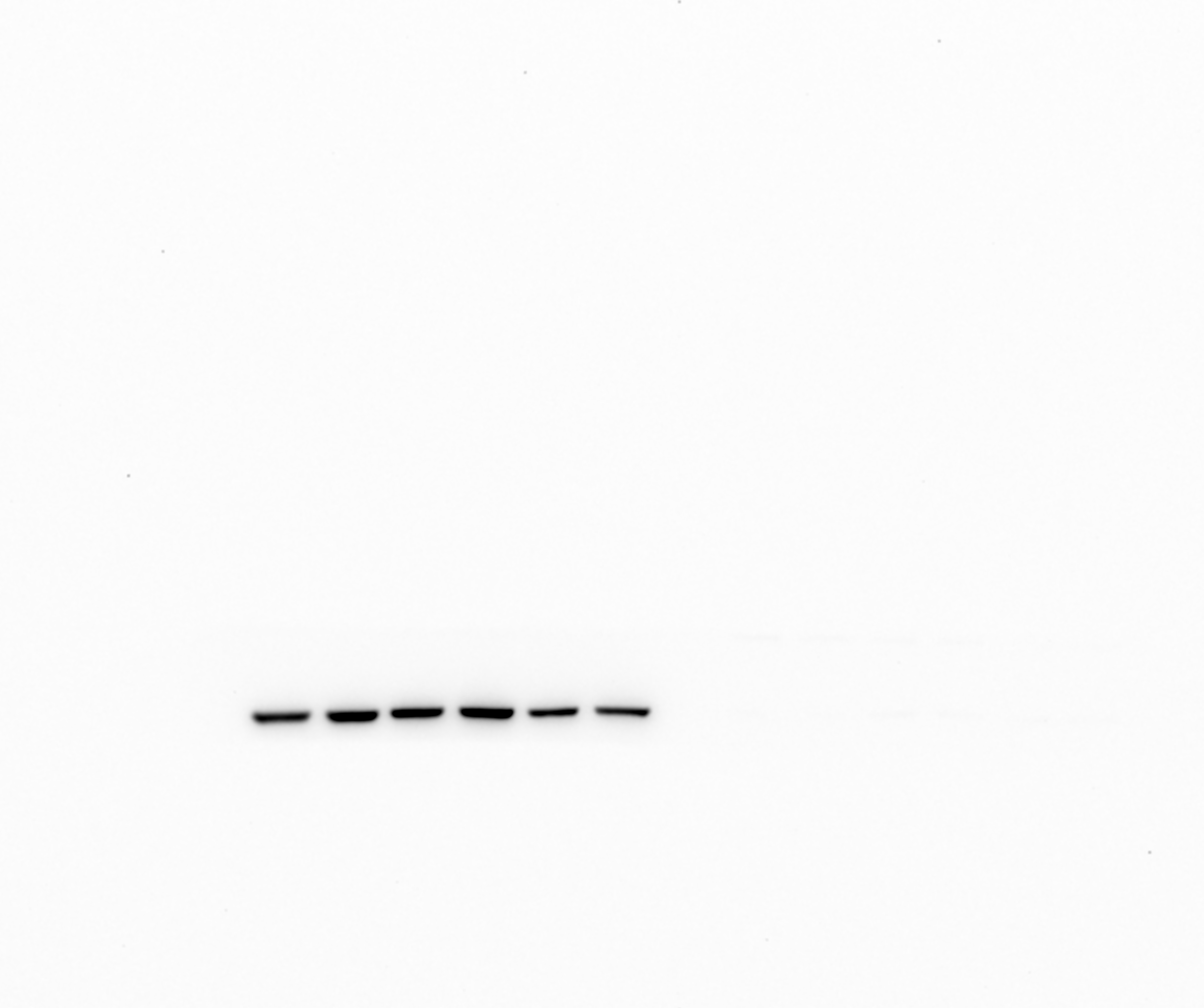

Supplement: Supplementary file 4 — Source data Fig. 1 [file 44319_2024_352_MOESM4_ESM.zip › Figure 1/1H/LaminB 293T-WT+mock-sev 6-18+mock-sev 2-9+mock-sev he fenli -4.tif]

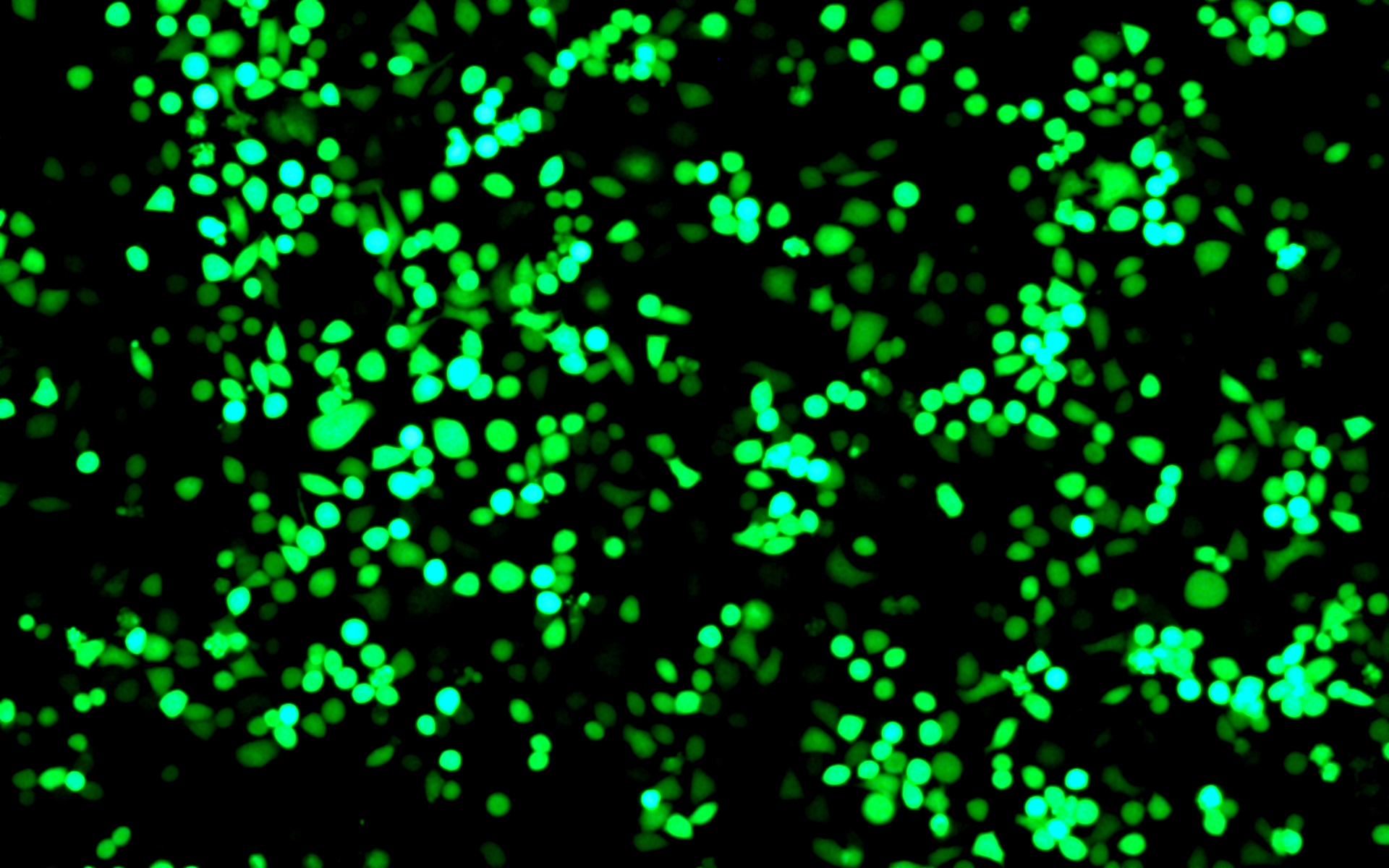

Supplement: Supplementary file 4 — Source data Fig. 1 [file 44319_2024_352_MOESM4_ESM.zip › Figure 1/1I/A14-2-1.jpg]

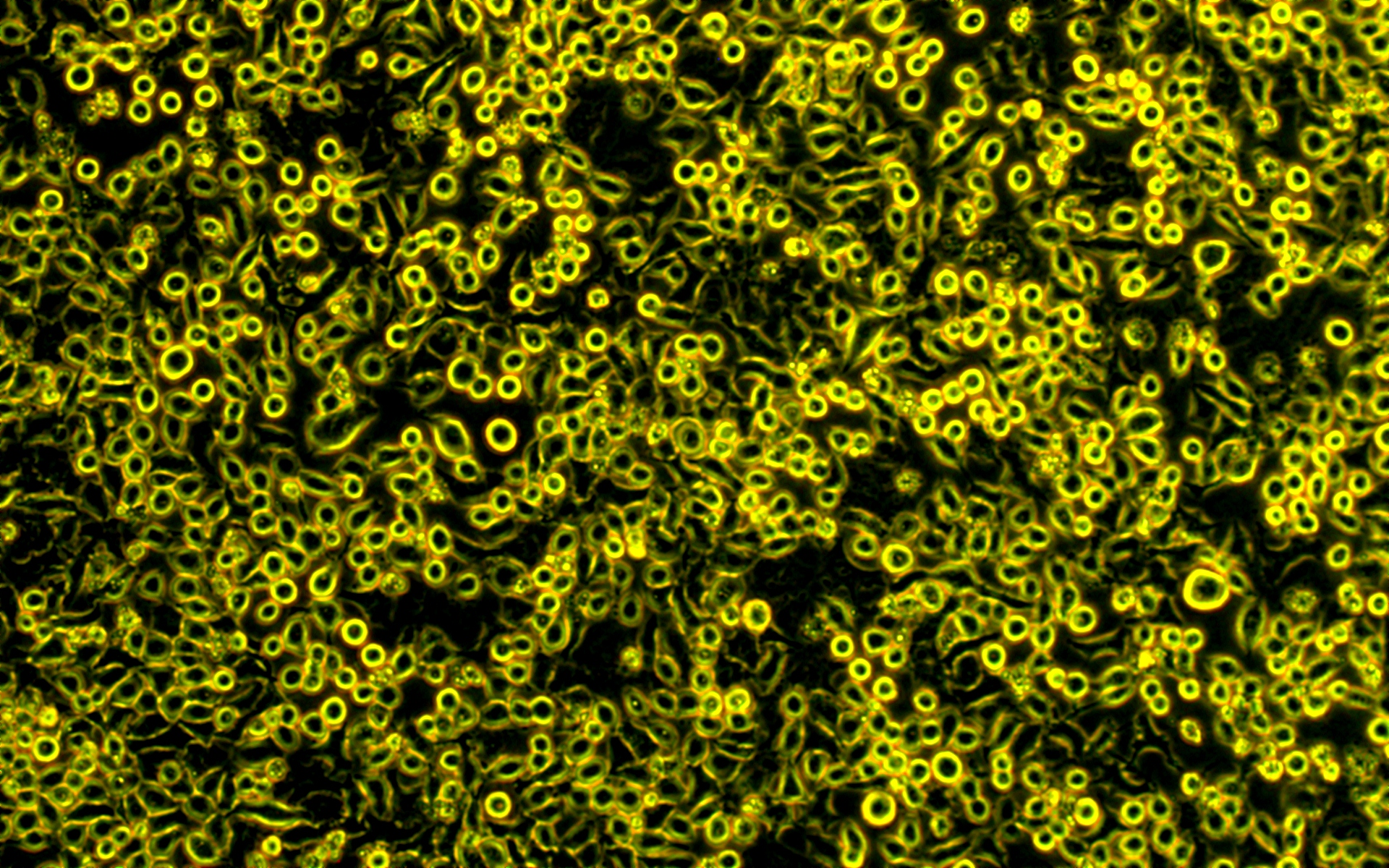

Supplement: Supplementary file 4 — Source data Fig. 1 [file 44319_2024_352_MOESM4_ESM.zip › Figure 1/1I/A14-2-2.jpg]

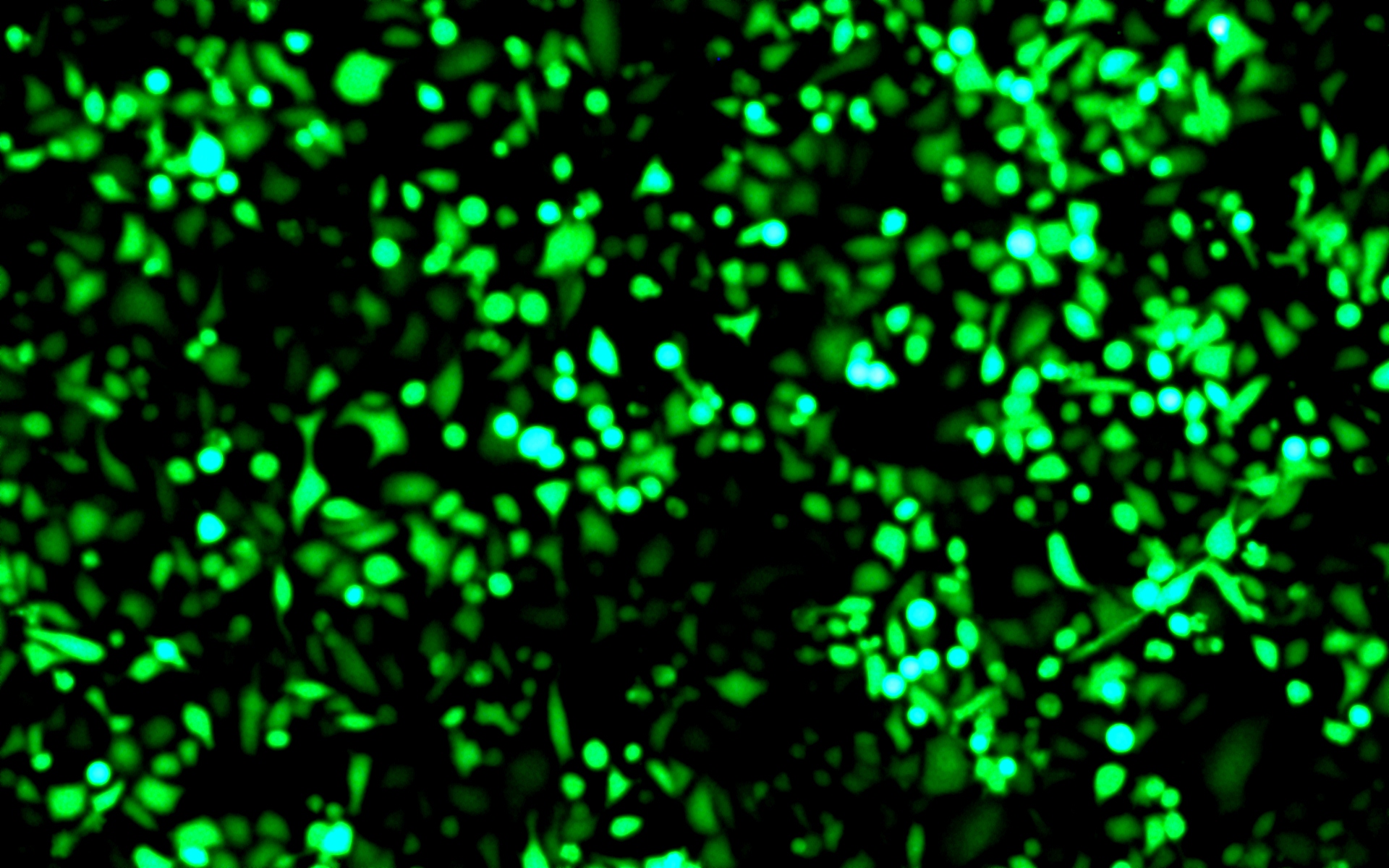

Supplement: Supplementary file 4 — Source data Fig. 1 [file 44319_2024_352_MOESM4_ESM.zip › Figure 1/1I/A46-2-1.jpg]

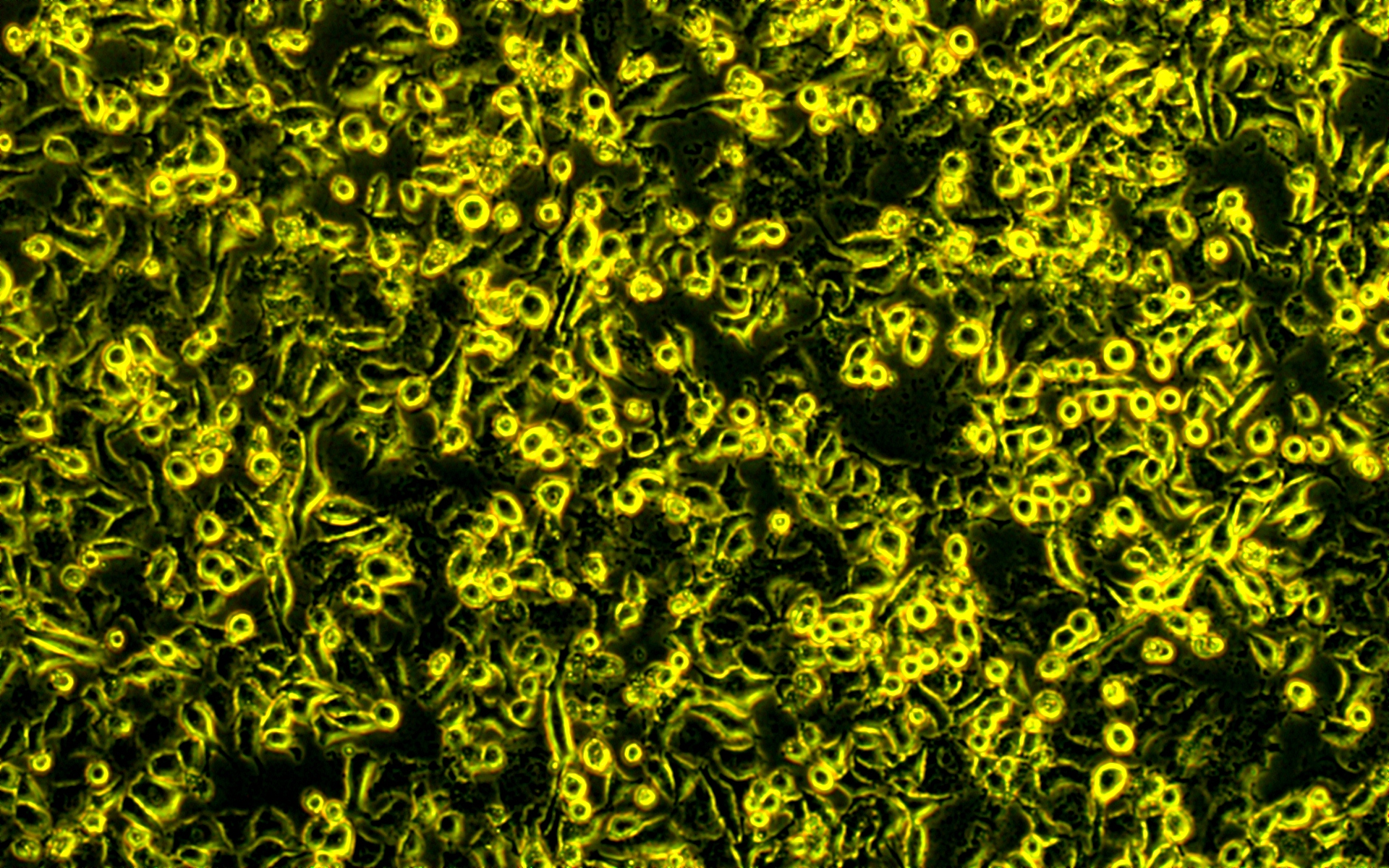

Supplement: Supplementary file 4 — Source data Fig. 1 [file 44319_2024_352_MOESM4_ESM.zip › Figure 1/1I/A46-2-2.jpg]

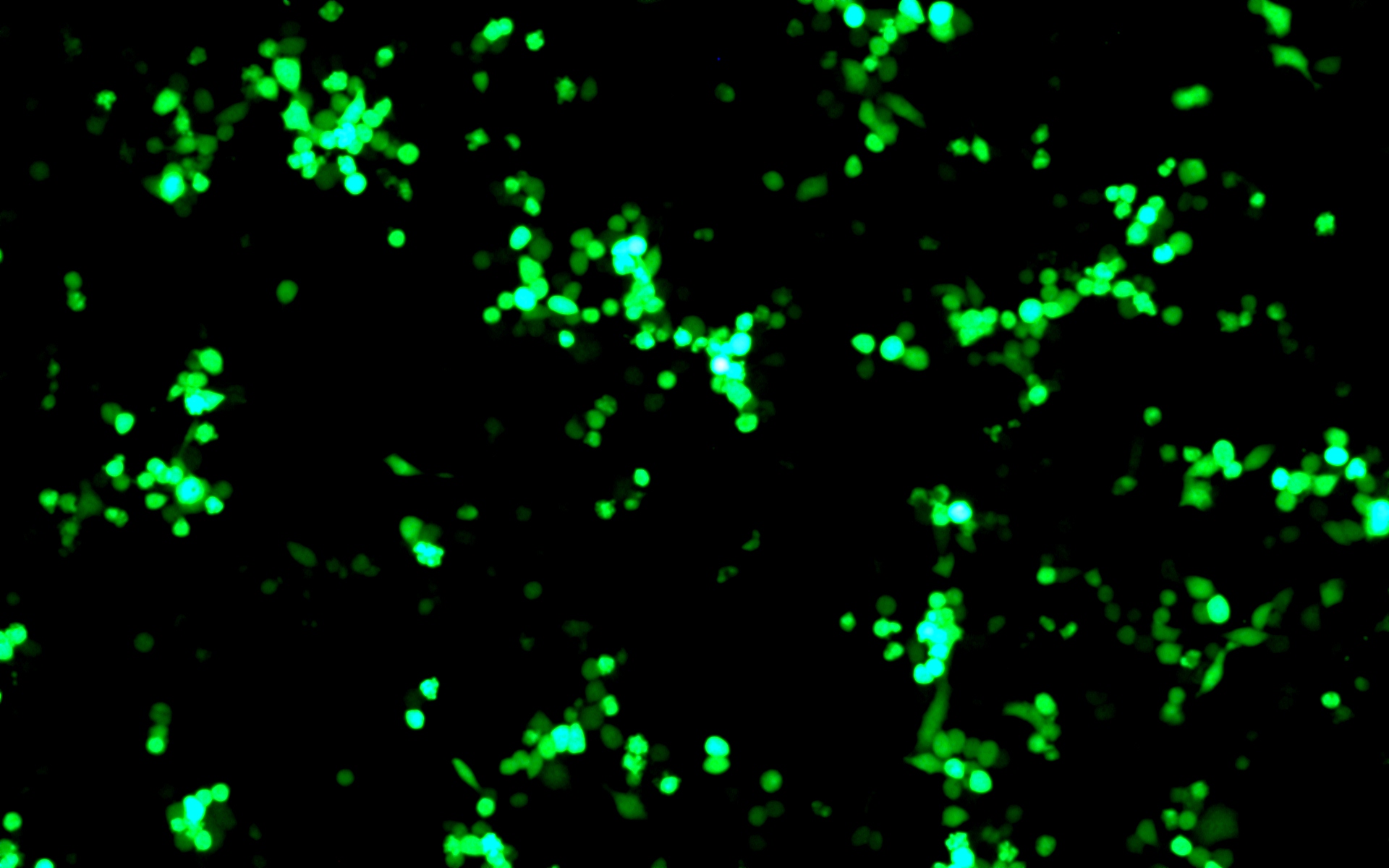

Supplement: Supplementary file 4 — Source data Fig. 1 [file 44319_2024_352_MOESM4_ESM.zip › Figure 1/1I/WT-1-1.jpg]

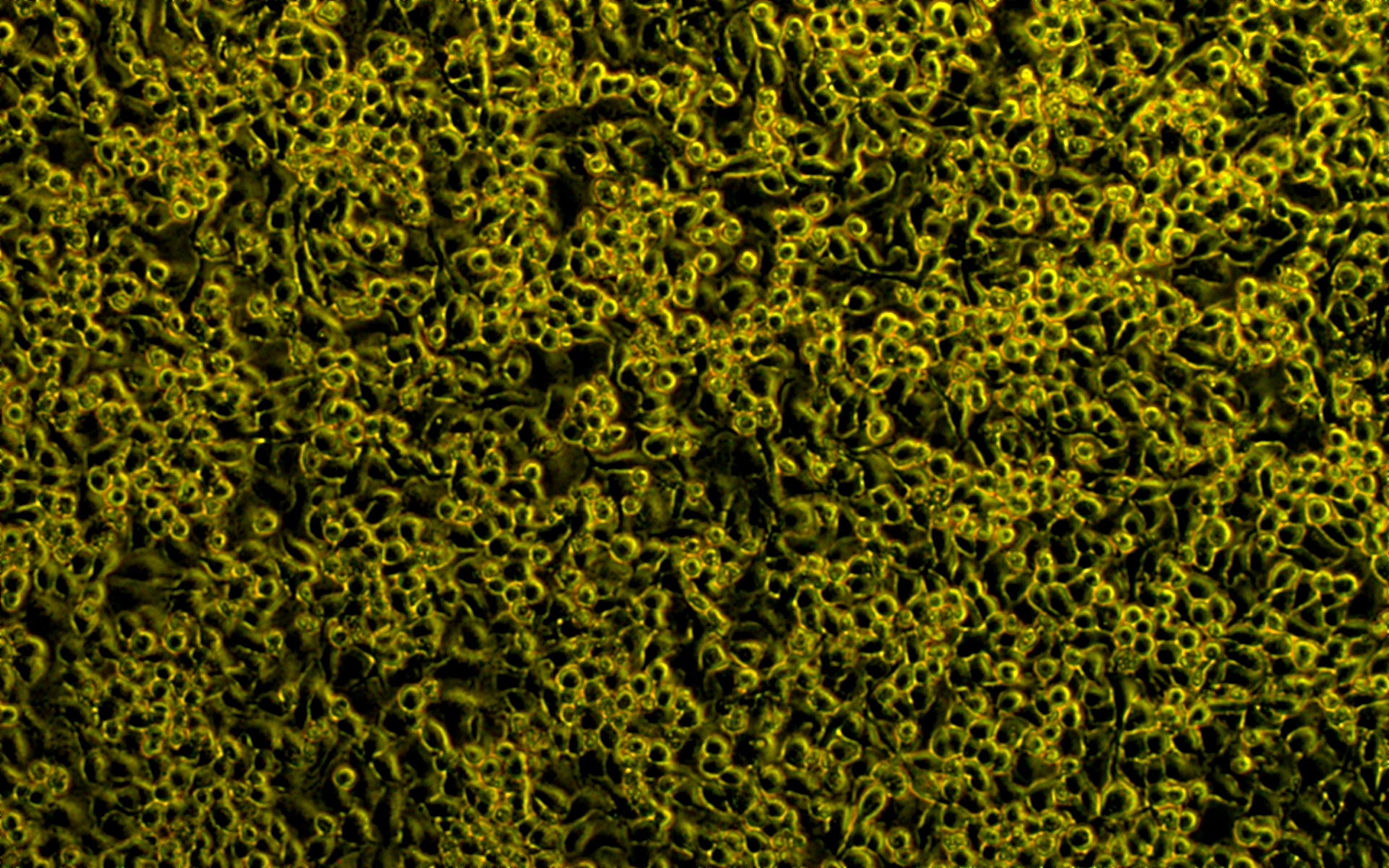

Supplement: Supplementary file 4 — Source data Fig. 1 [file 44319_2024_352_MOESM4_ESM.zip › Figure 1/1I/WT-1-2.jpg]

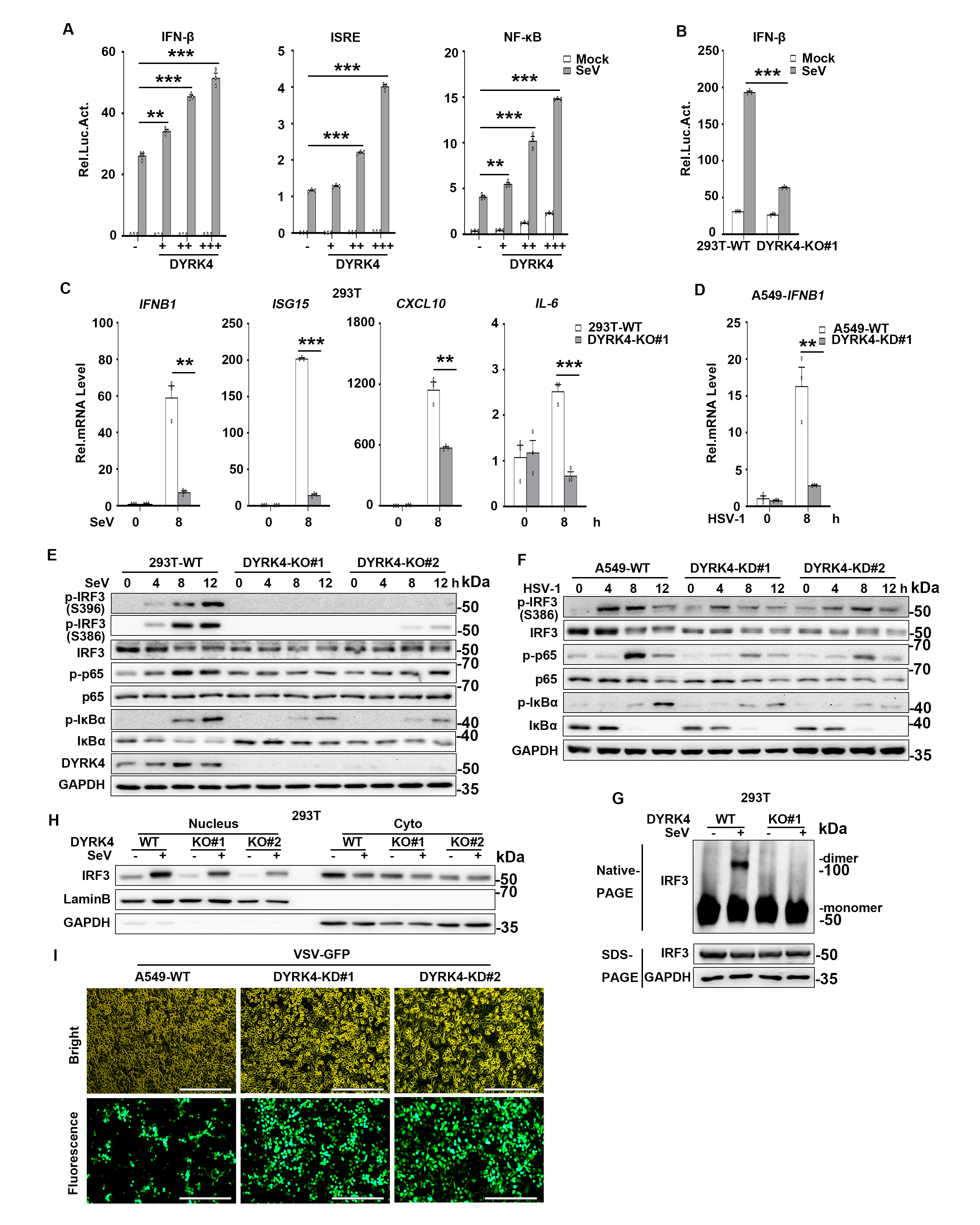

Supplement: Supplementary file 4 — Source data Fig. 1 [file 44319_2024_352_MOESM4_ESM.zip › Figure 1/Figure 1.tif]

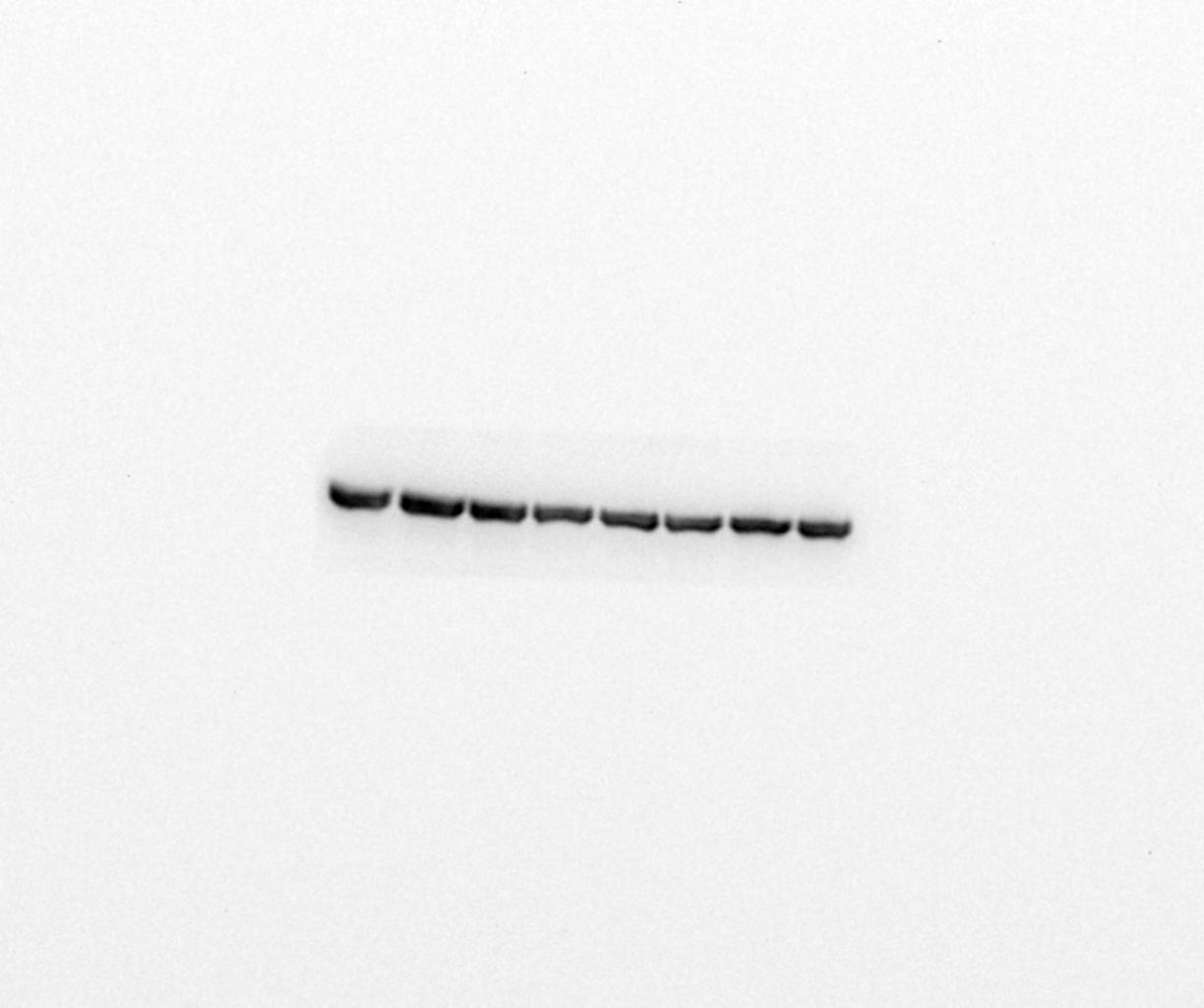

Supplement: Supplementary file 5 — Source data Fig. 2 [file 44319_2024_352_MOESM5_ESM.zip › Figure 2/2E/SeV/western IRF3.tif]

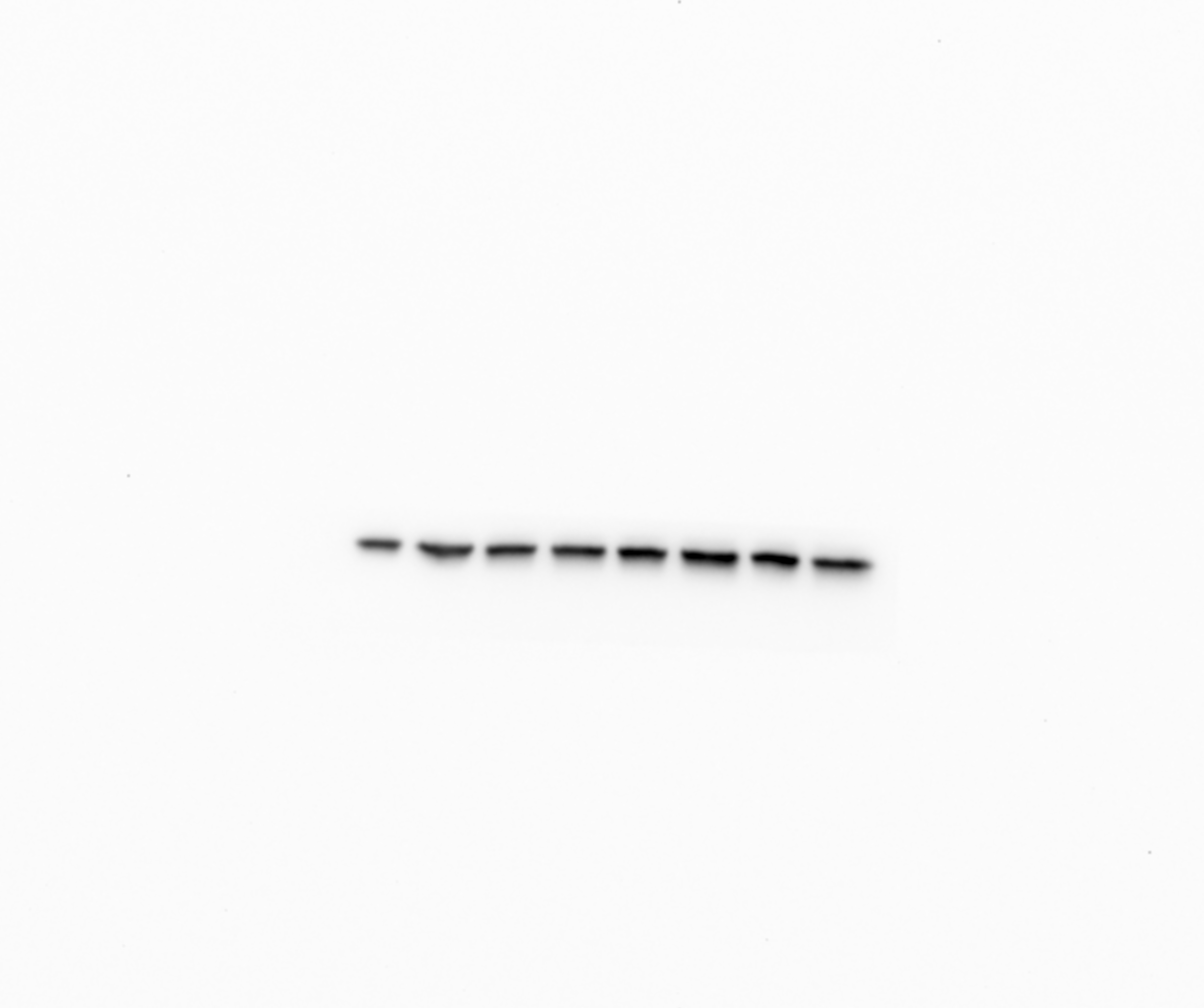

Supplement: Supplementary file 5 — Source data Fig. 2 [file 44319_2024_352_MOESM5_ESM.zip › Figure 2/2E/SeV/western GAPDH.tif]

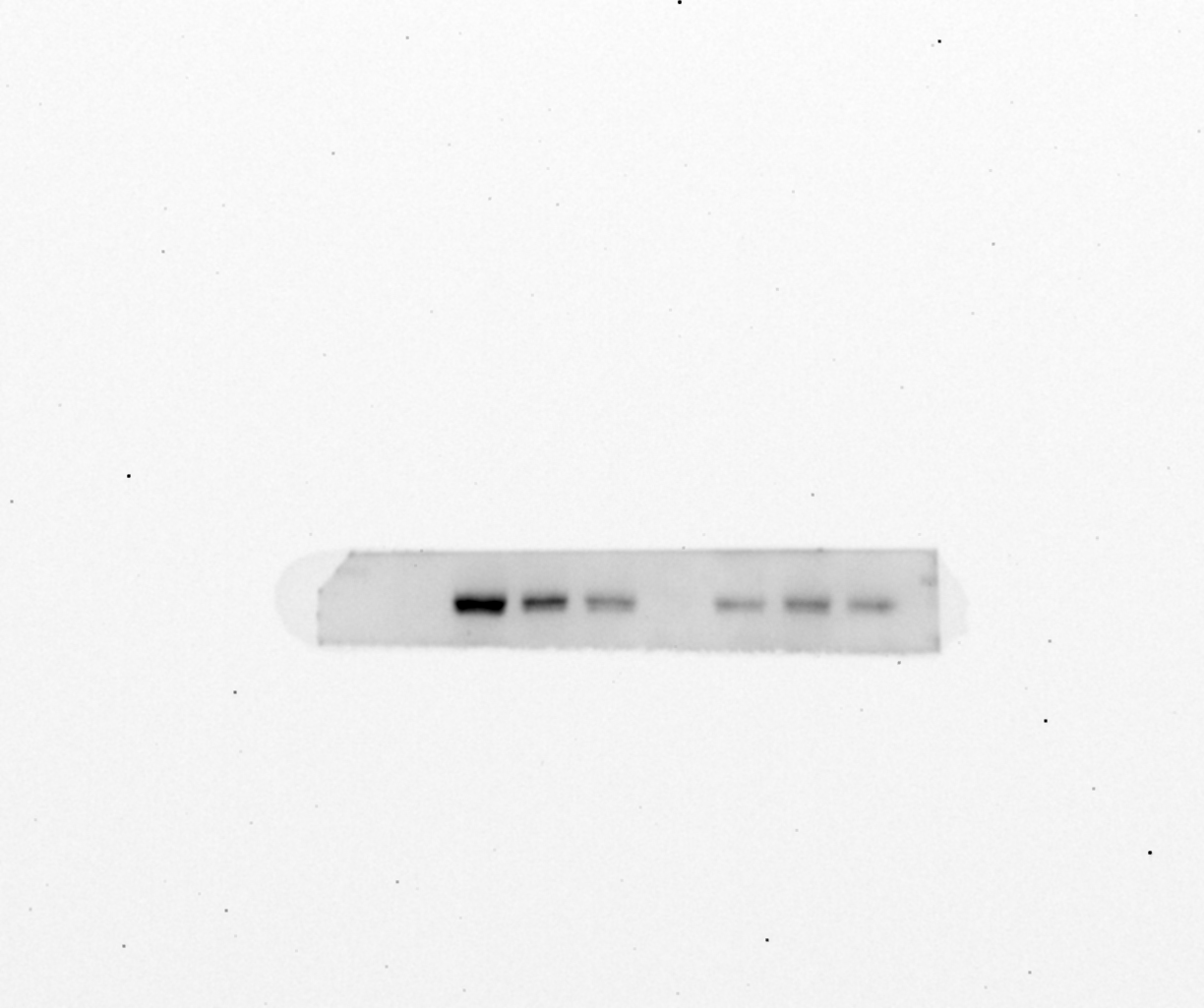

Supplement: Supplementary file 5 — Source data Fig. 2 [file 44319_2024_352_MOESM5_ESM.zip › Figure 2/2E/SeV/western p-IRF3.tif]

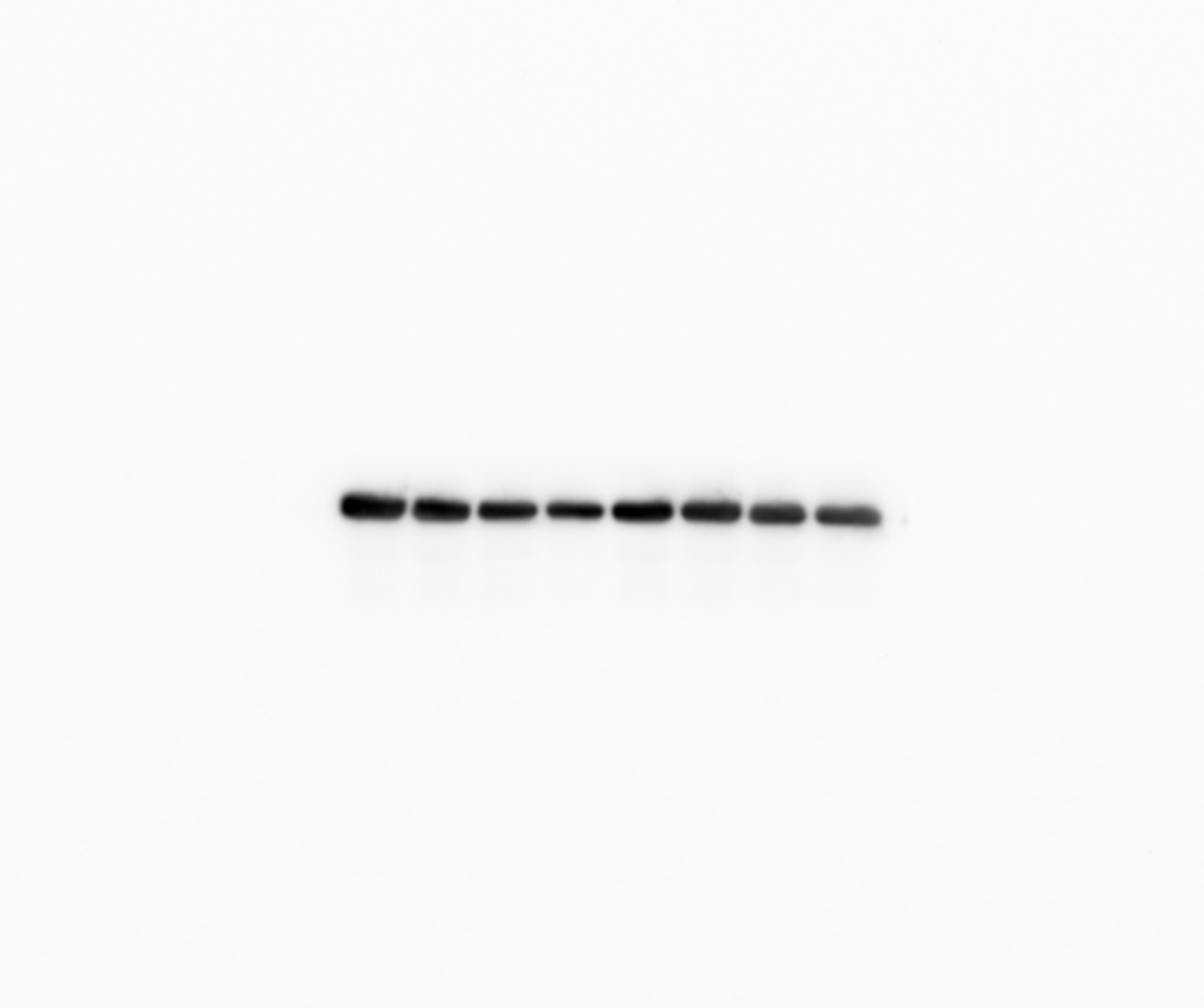

Supplement: Supplementary file 5 — Source data Fig. 2 [file 44319_2024_352_MOESM5_ESM.zip › Figure 2/2E/VSV/western GAPDH.tif]

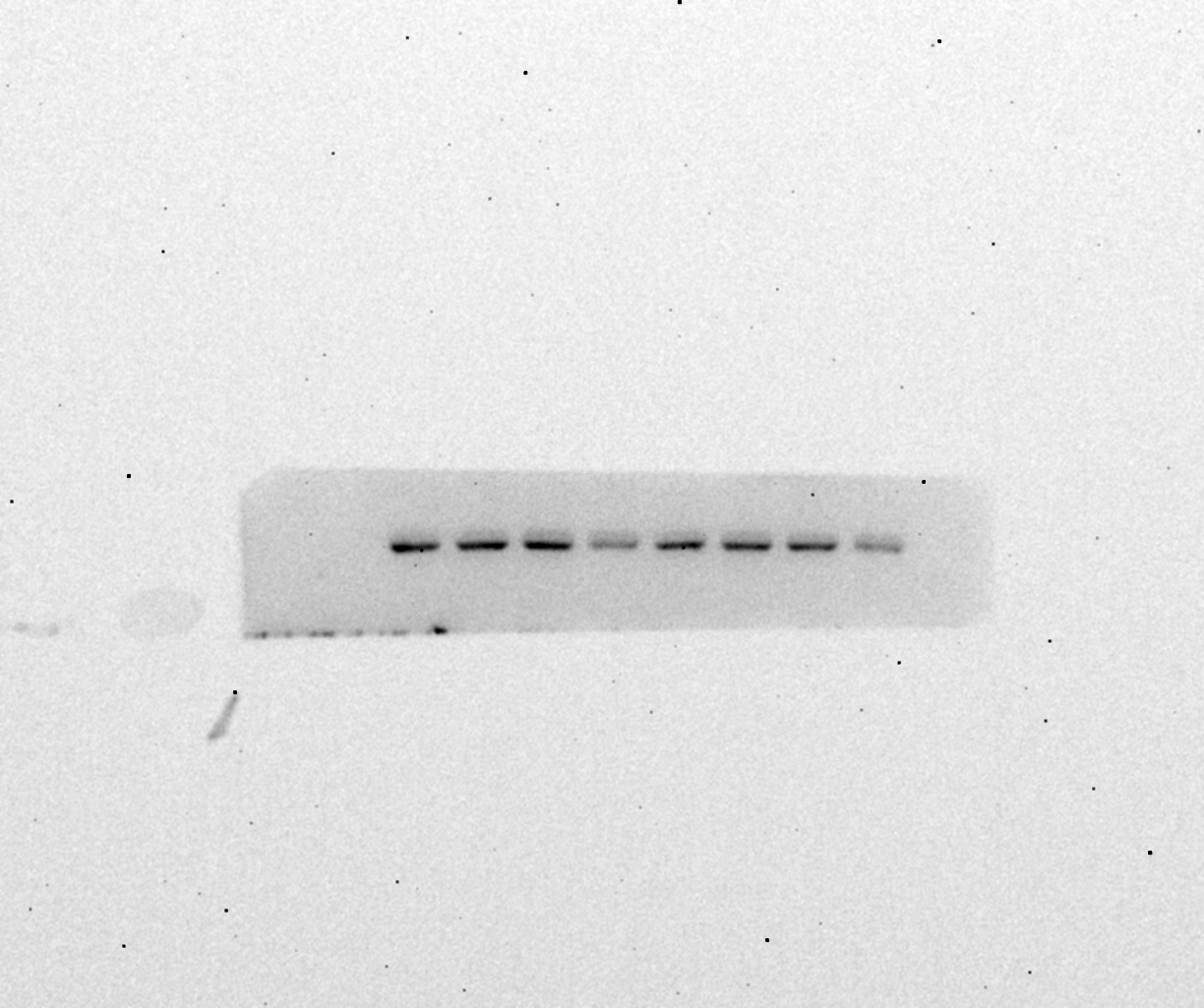

Supplement: Supplementary file 5 — Source data Fig. 2 [file 44319_2024_352_MOESM5_ESM.zip › Figure 2/2E/VSV/western IRF3.tif]

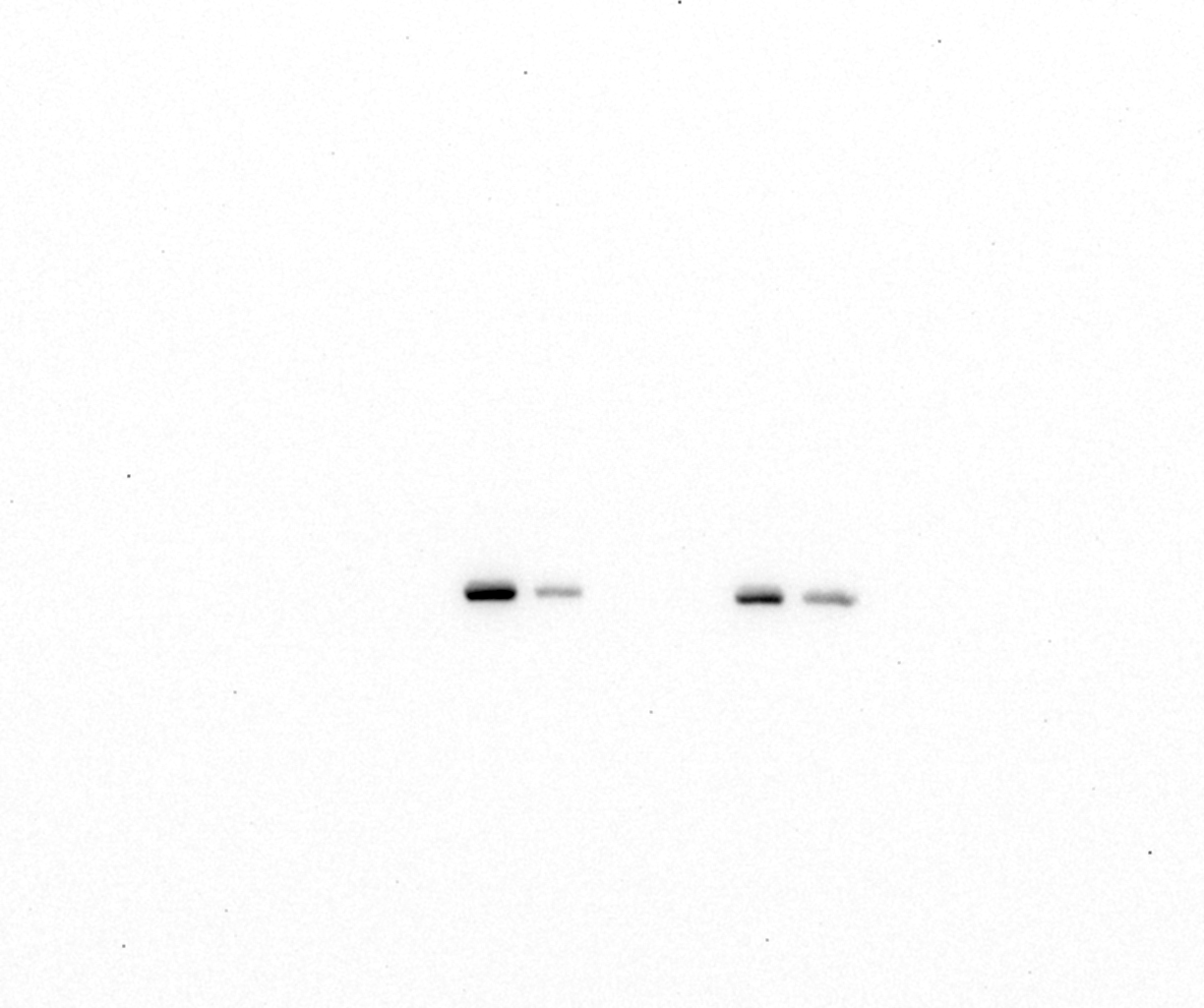

Supplement: Supplementary file 5 — Source data Fig. 2 [file 44319_2024_352_MOESM5_ESM.zip › Figure 2/2E/VSV/western p-IRF3.tif]

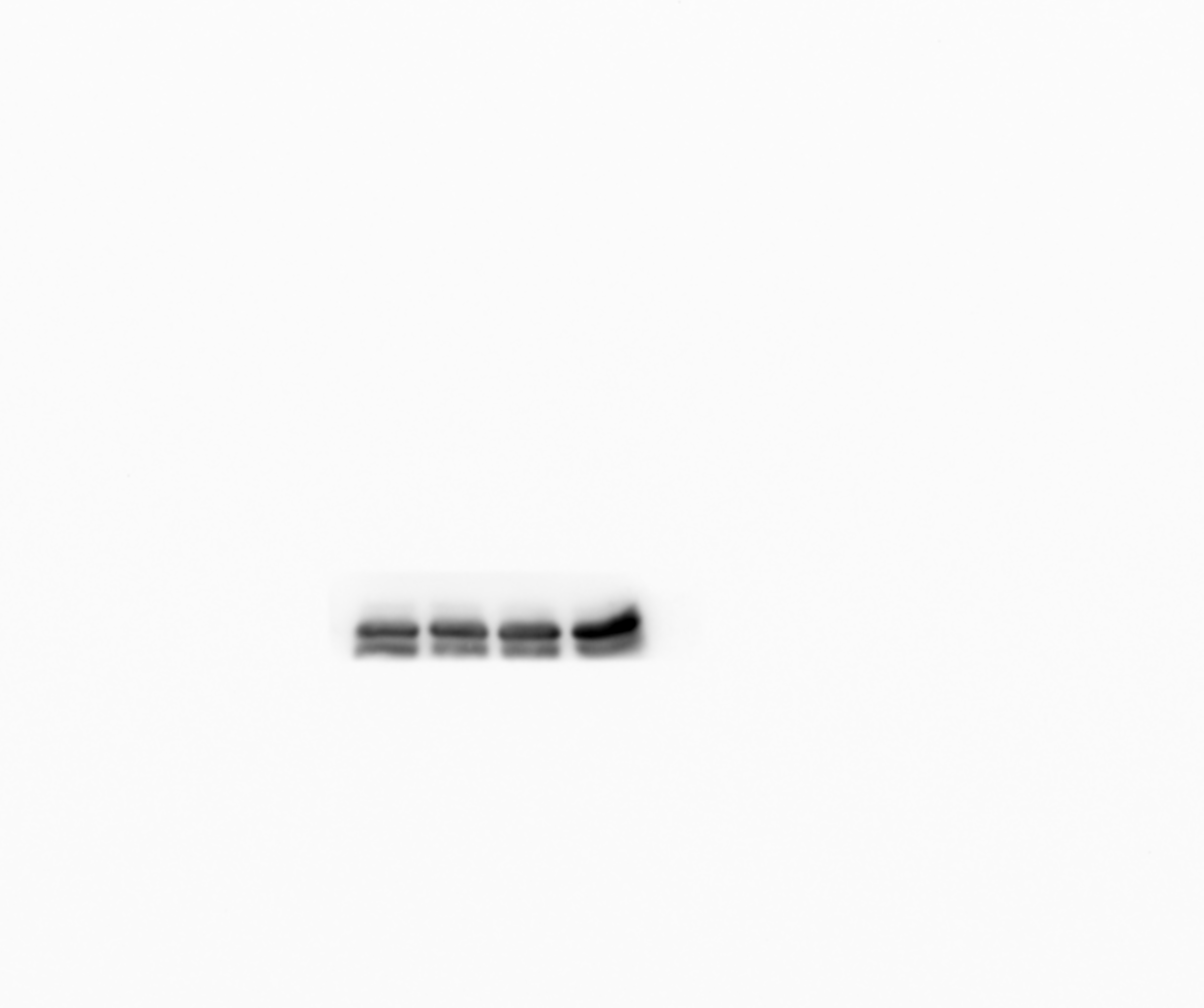

Supplement: Supplementary file 5 — Source data Fig. 2 [file 44319_2024_352_MOESM5_ESM.zip › Figure 2/2F/western GAPDH.tif]

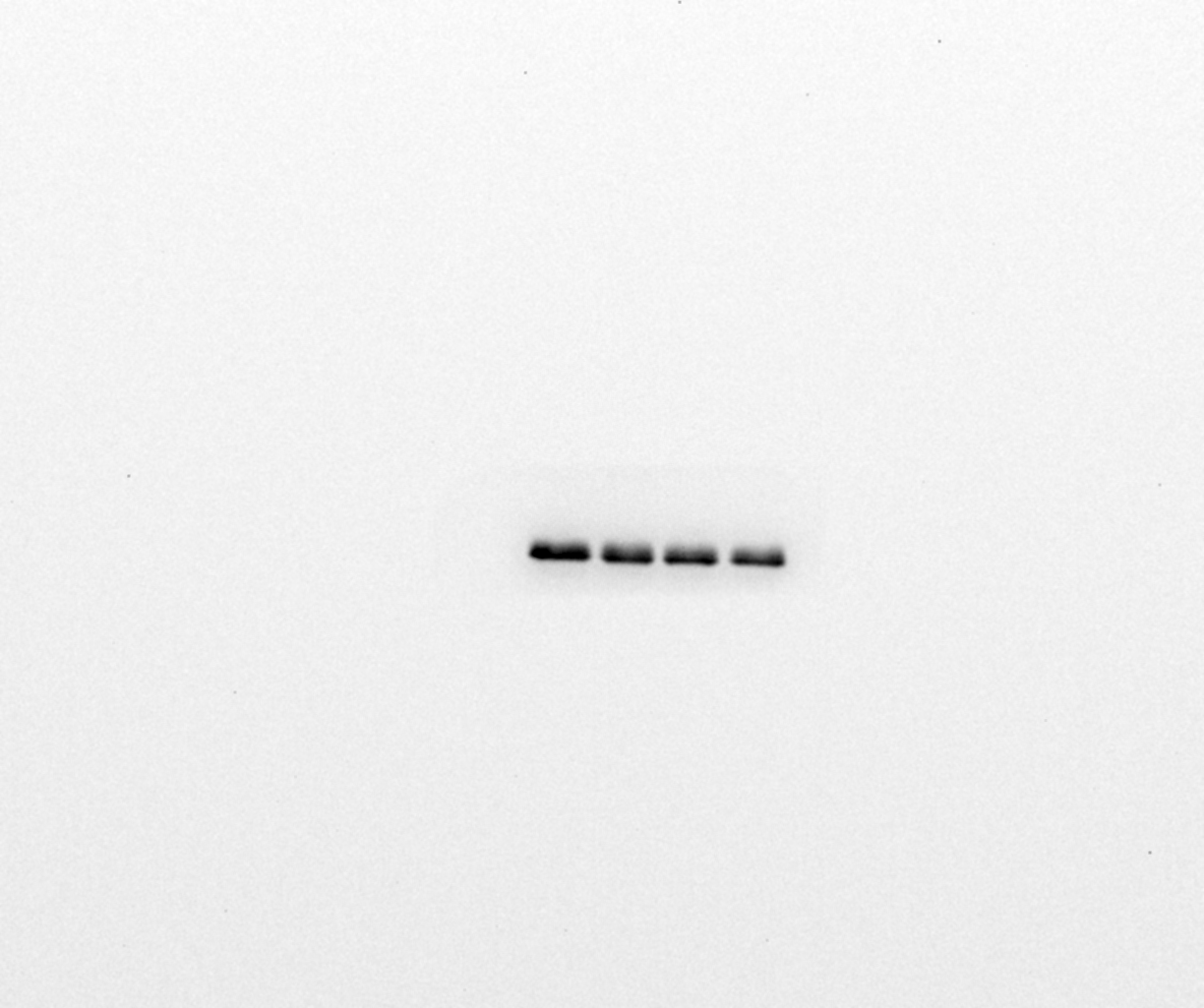

Supplement: Supplementary file 5 — Source data Fig. 2 [file 44319_2024_352_MOESM5_ESM.zip › Figure 2/2F/western IRF3.tif]

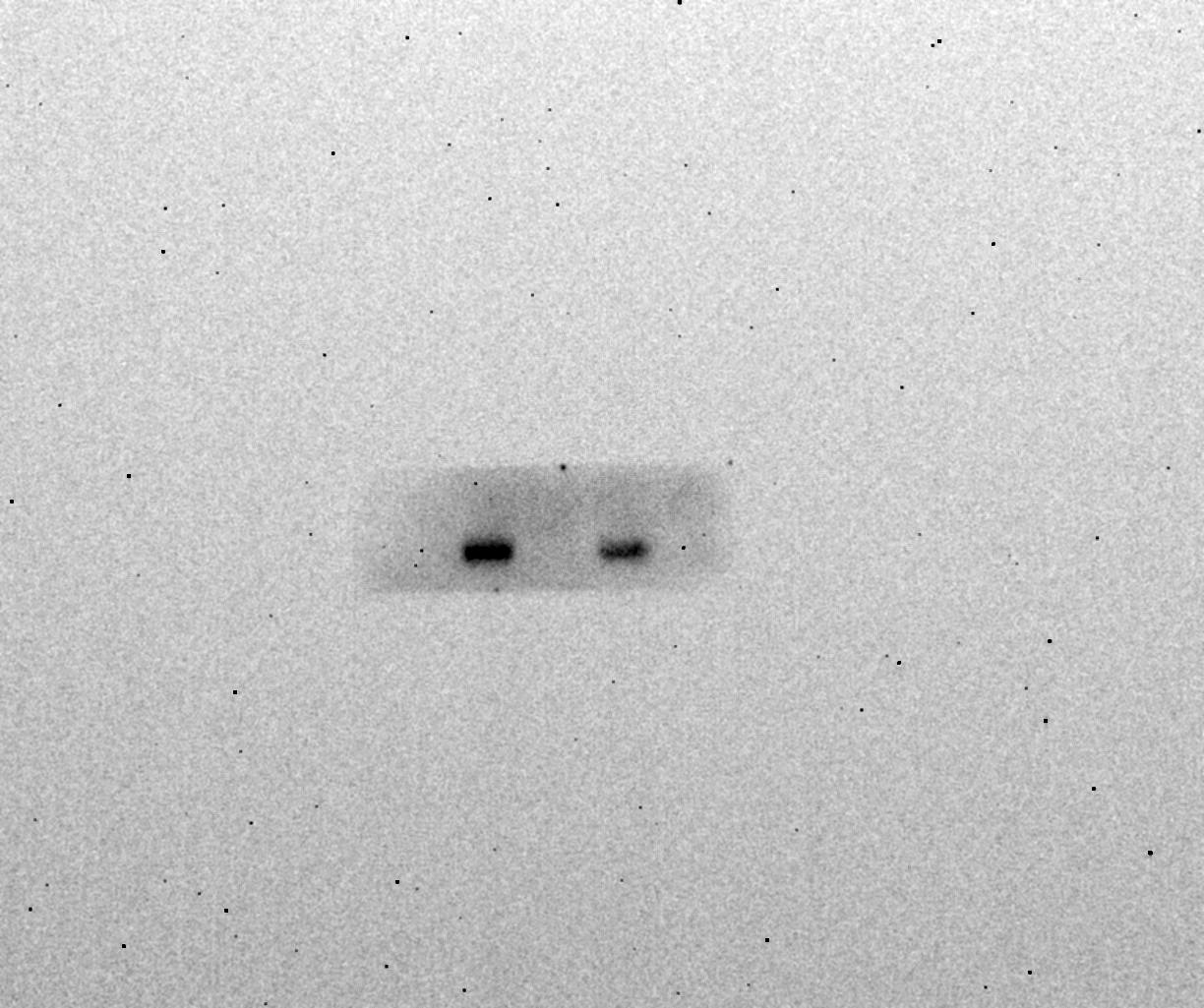

Supplement: Supplementary file 5 — Source data Fig. 2 [file 44319_2024_352_MOESM5_ESM.zip › Figure 2/2F/western p-IRF3.tif]

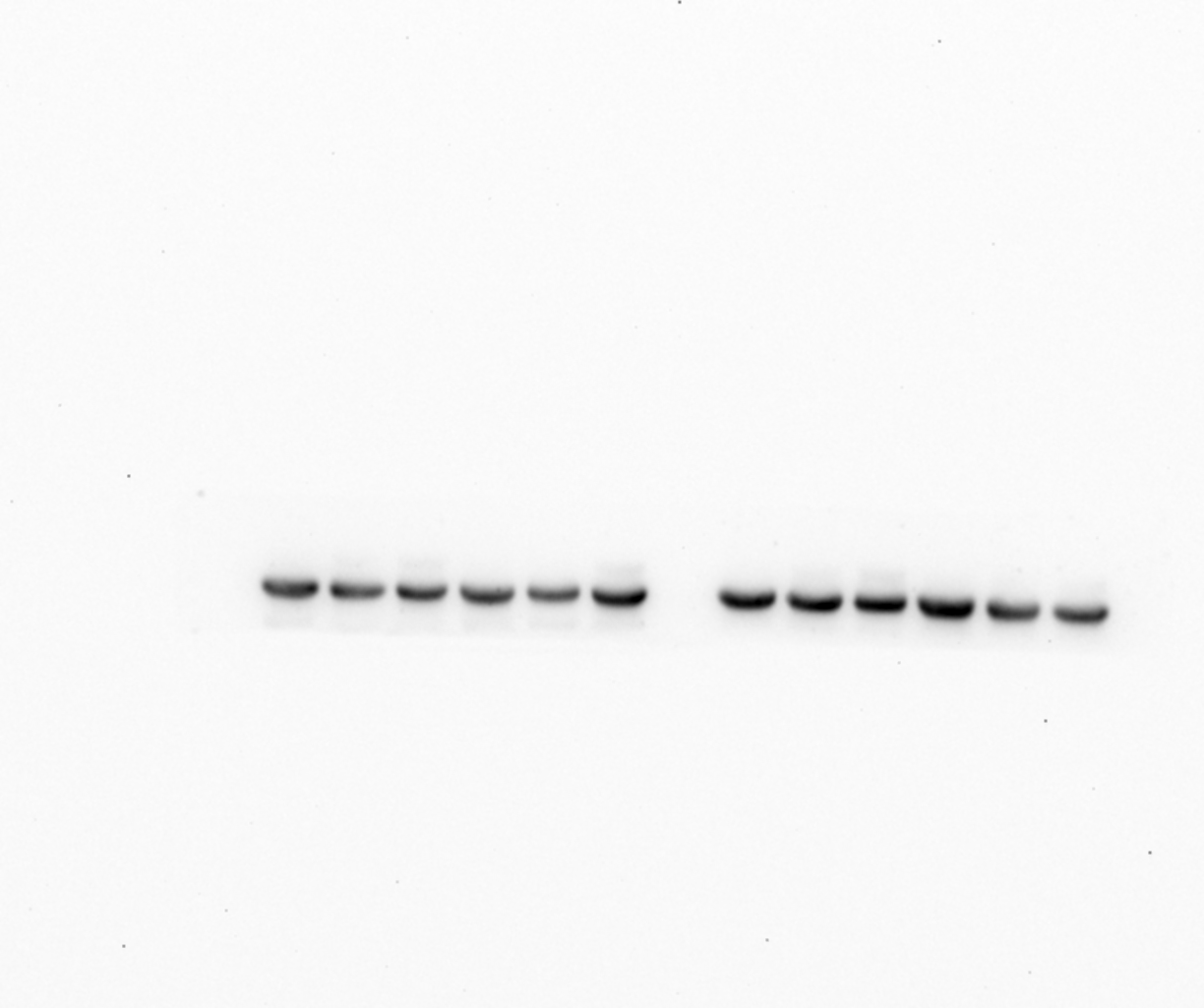

Supplement: Supplementary file 5 — Source data Fig. 2 [file 44319_2024_352_MOESM5_ESM.zip › Figure 2/2G/SeV/westren GAPDH.tif]

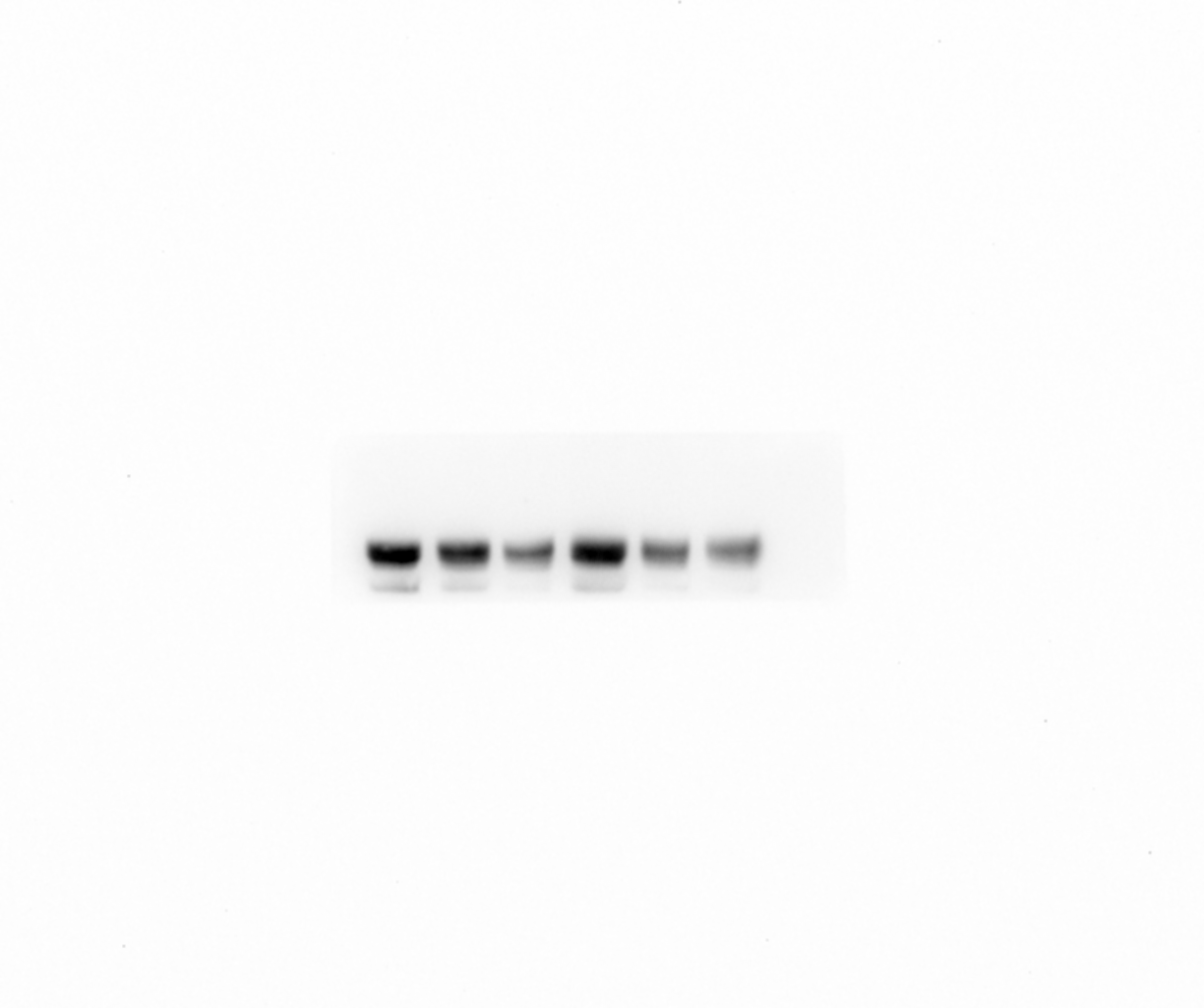

Supplement: Supplementary file 5 — Source data Fig. 2 [file 44319_2024_352_MOESM5_ESM.zip › Figure 2/2G/SeV/westren IRF3.tif]

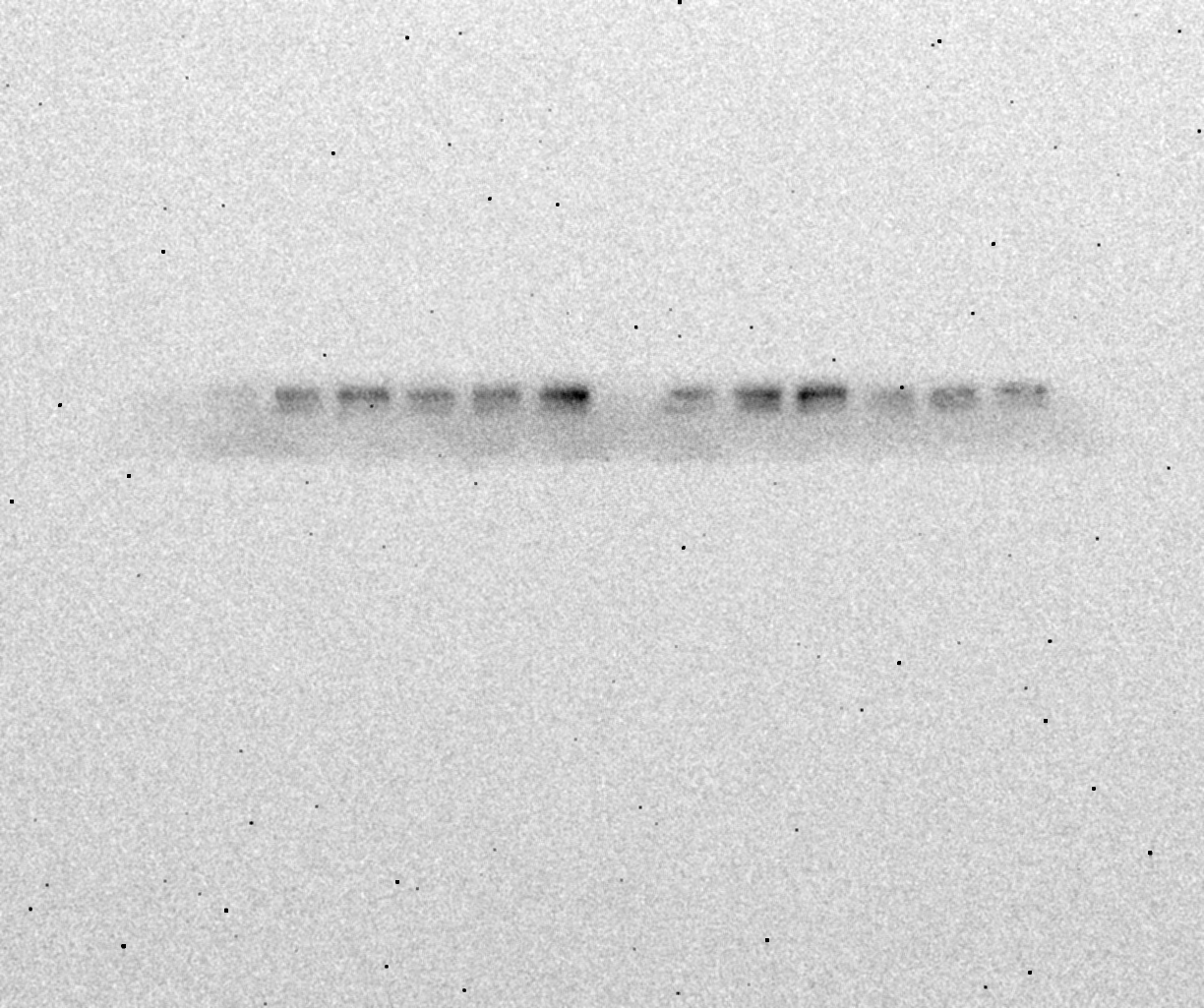

Supplement: Supplementary file 5 — Source data Fig. 2 [file 44319_2024_352_MOESM5_ESM.zip › Figure 2/2G/SeV/westren P65.tif]

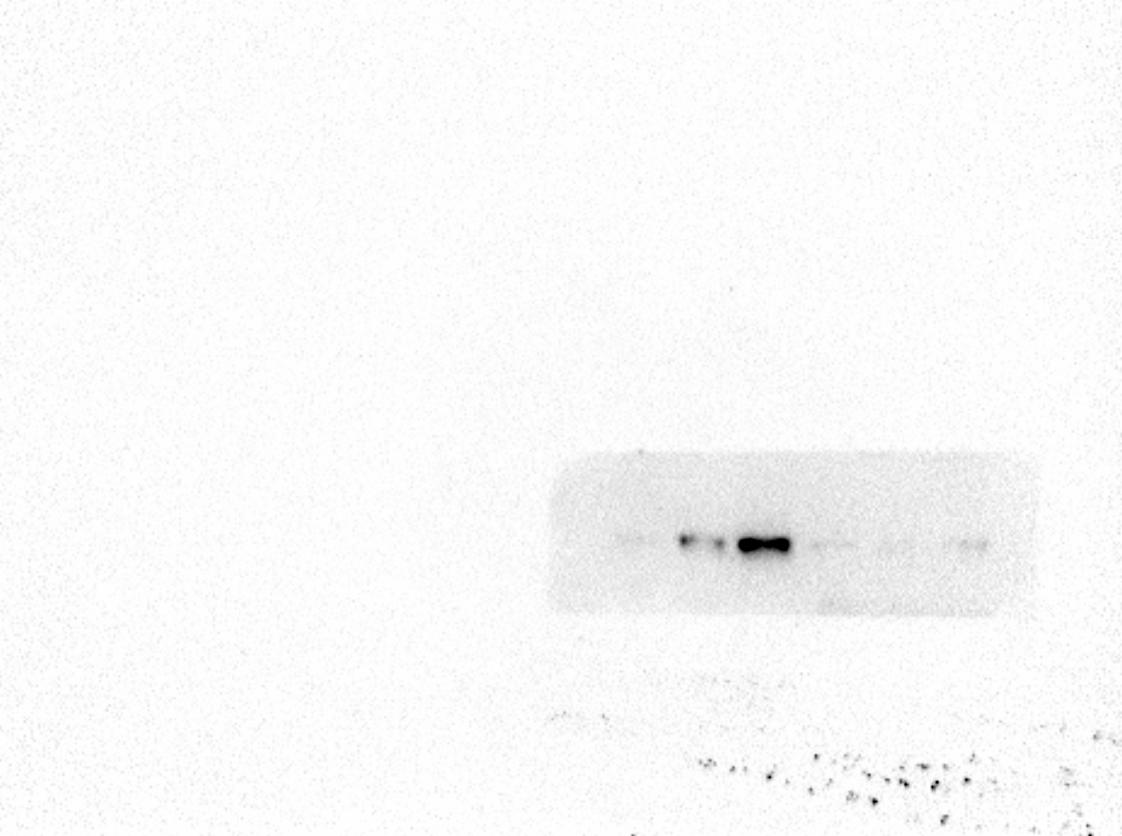

Supplement: Supplementary file 5 — Source data Fig. 2 [file 44319_2024_352_MOESM5_ESM.zip › Figure 2/2G/SeV/westren p-IRF3.tif]

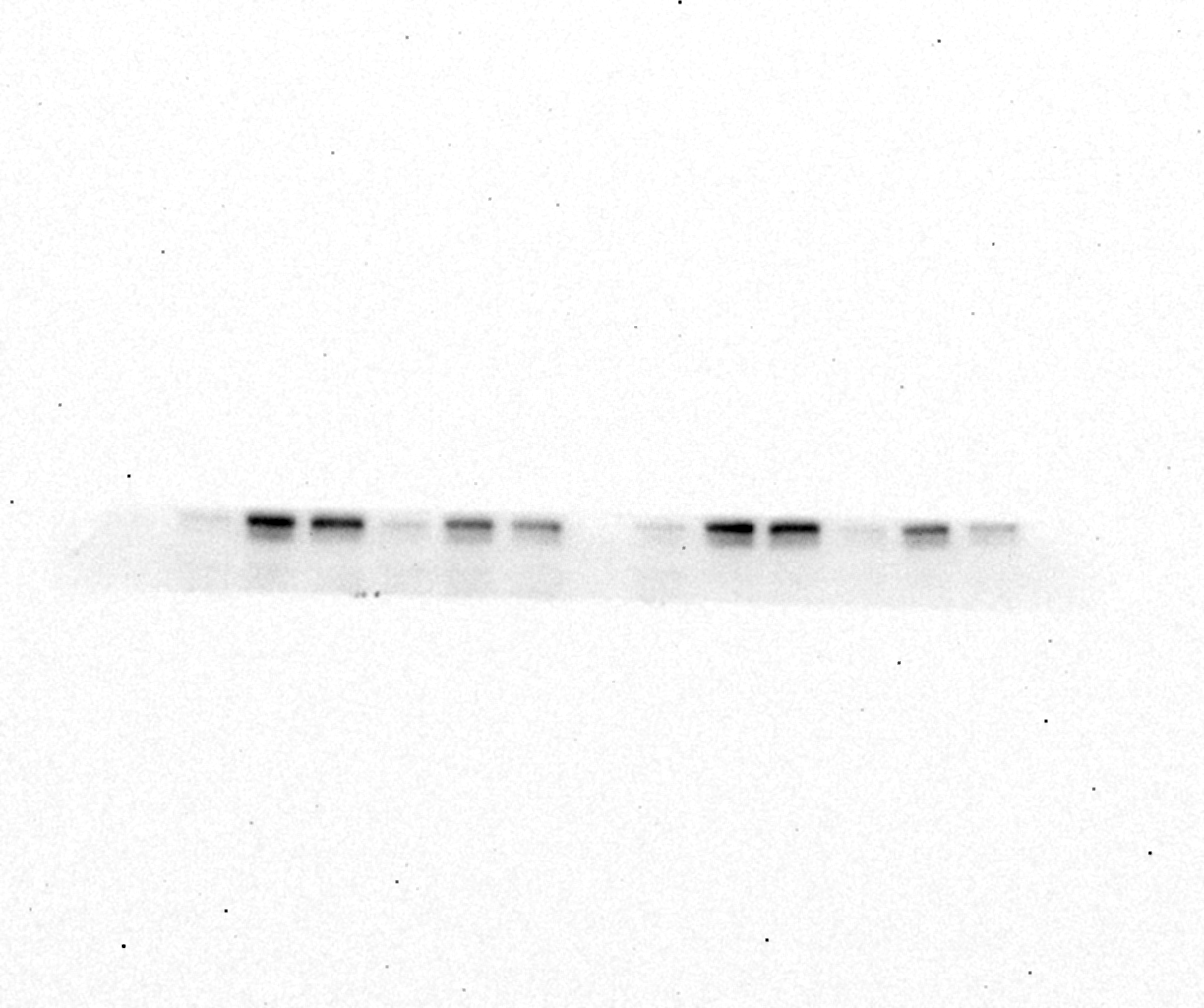

Supplement: Supplementary file 5 — Source data Fig. 2 [file 44319_2024_352_MOESM5_ESM.zip › Figure 2/2G/SeV/westren p-P65.tif]

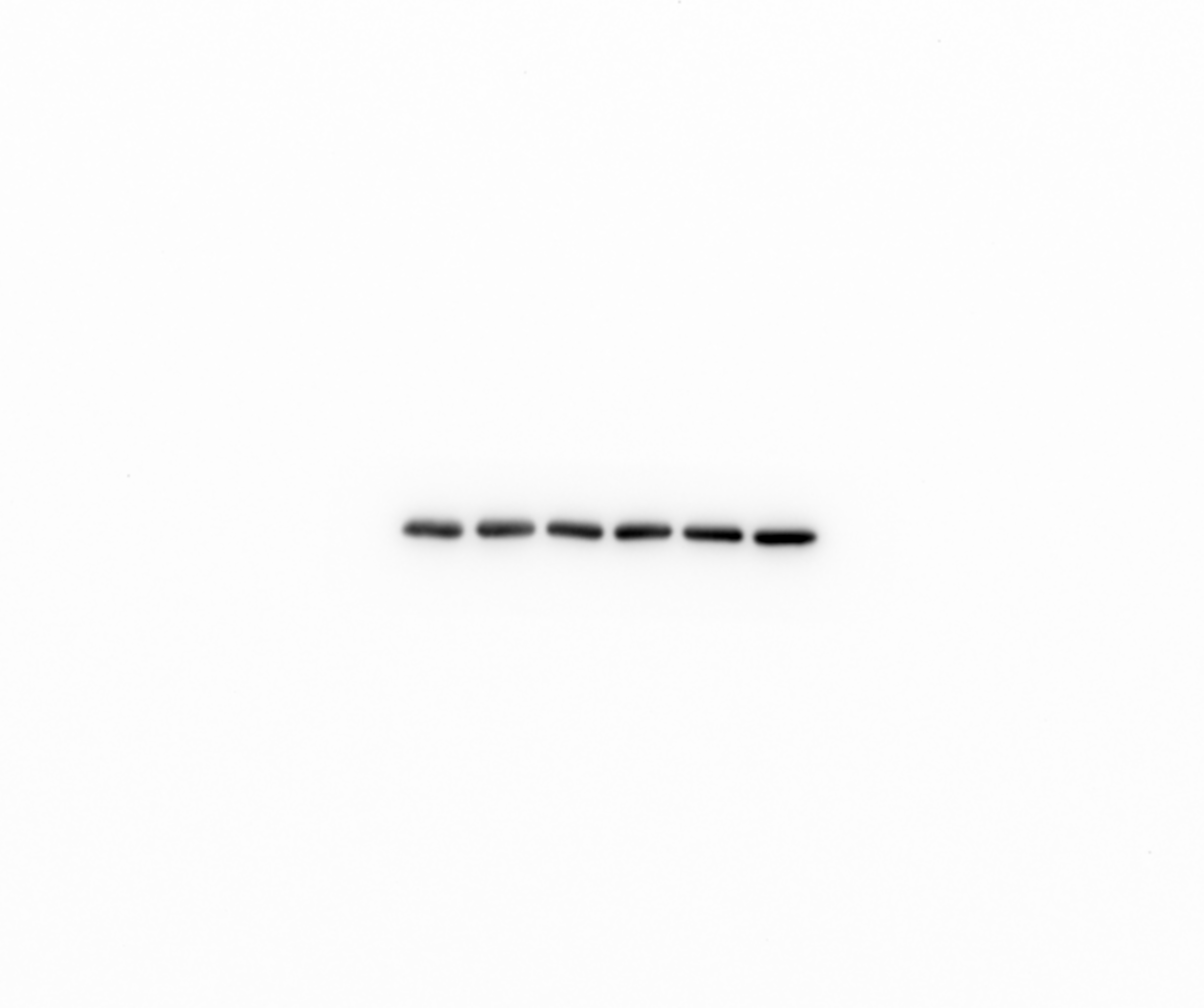

Supplement: Supplementary file 5 — Source data Fig. 2 [file 44319_2024_352_MOESM5_ESM.zip › Figure 2/2G/VSV/western GAPDH.tif]

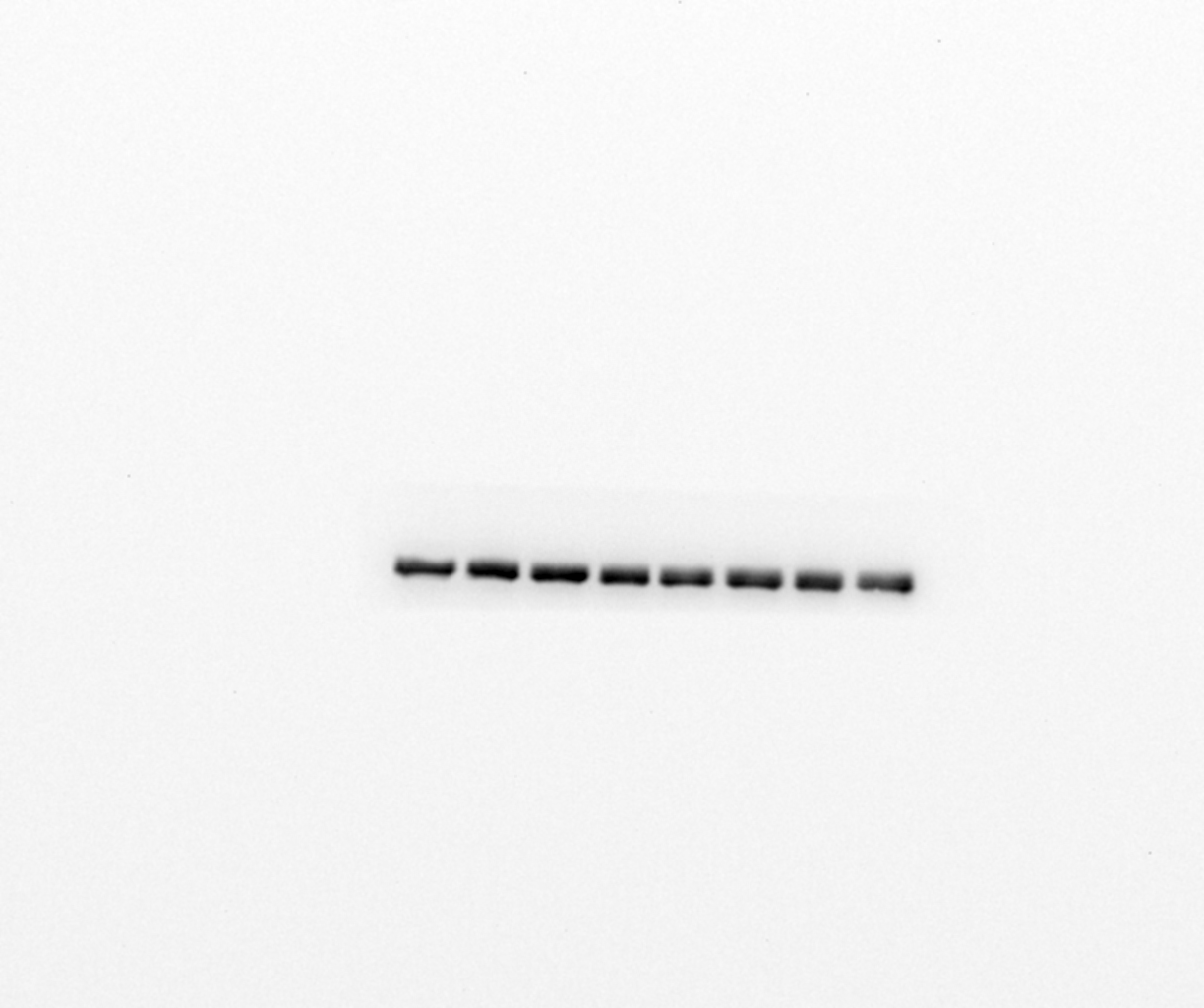

Supplement: Supplementary file 5 — Source data Fig. 2 [file 44319_2024_352_MOESM5_ESM.zip › Figure 2/2G/VSV/western IRF3.tif]

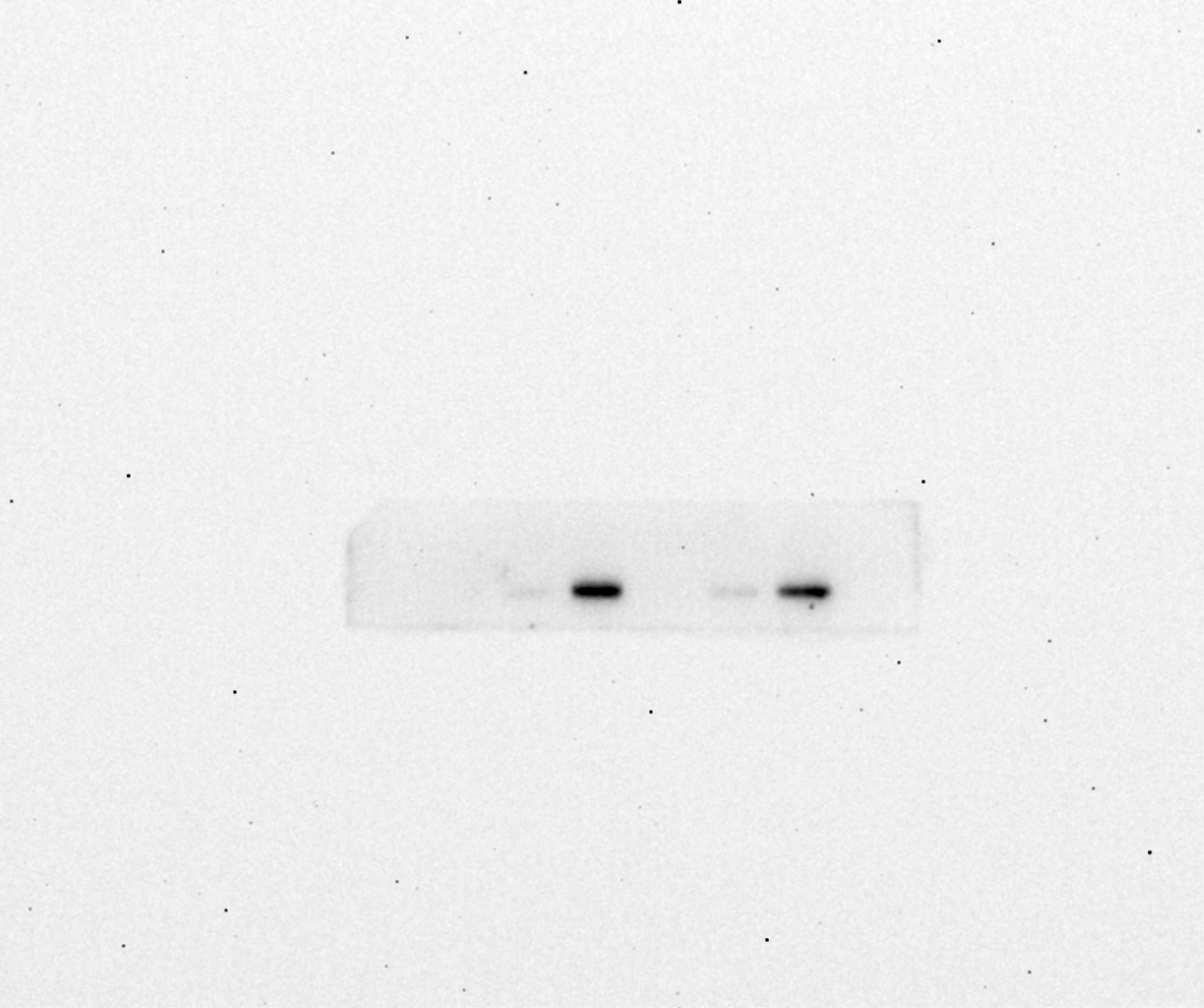

Supplement: Supplementary file 5 — Source data Fig. 2 [file 44319_2024_352_MOESM5_ESM.zip › Figure 2/2G/VSV/western p-IRF3.tif]

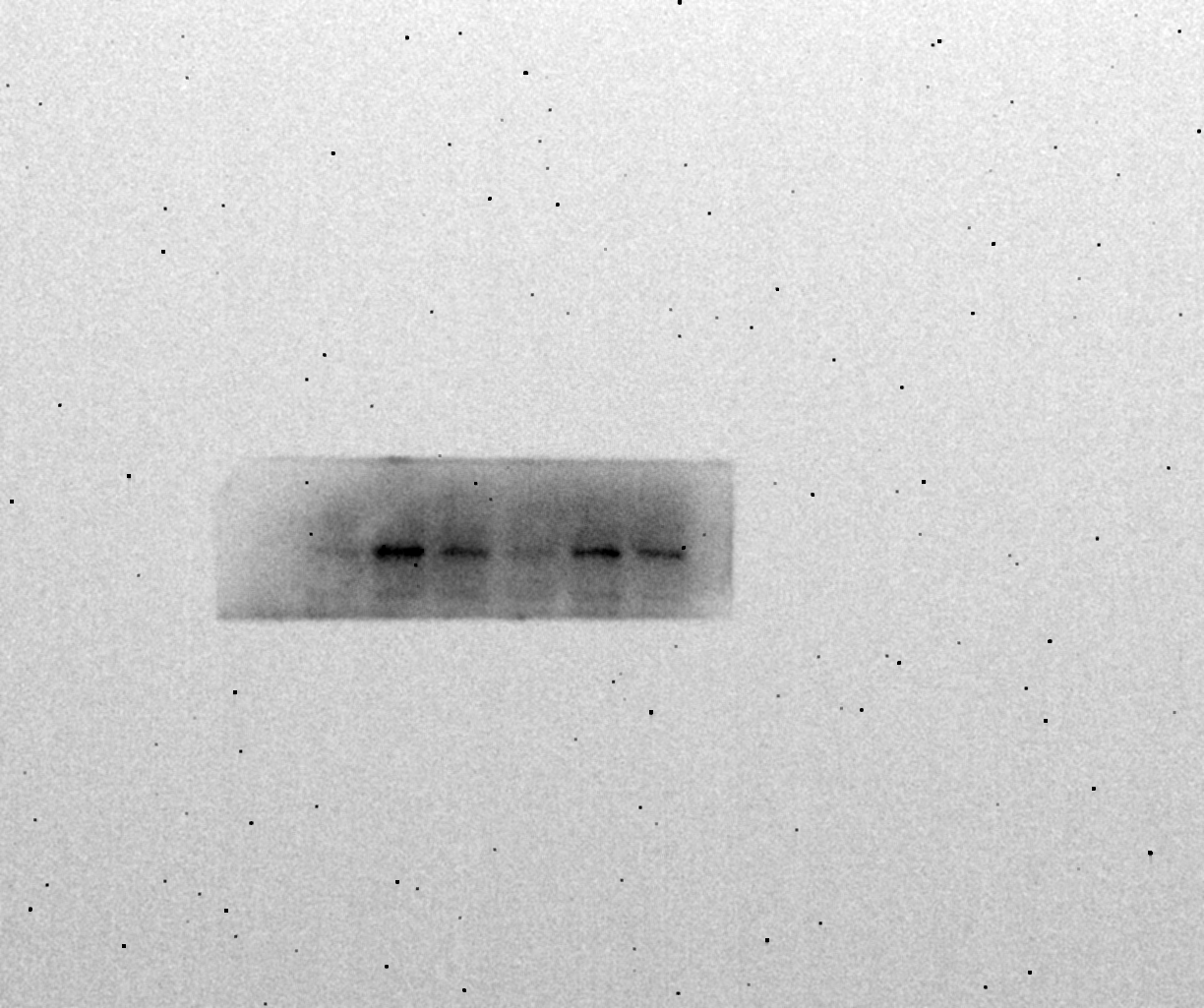

Supplement: Supplementary file 5 — Source data Fig. 2 [file 44319_2024_352_MOESM5_ESM.zip › Figure 2/2G/VSV/western p-P65.tif]

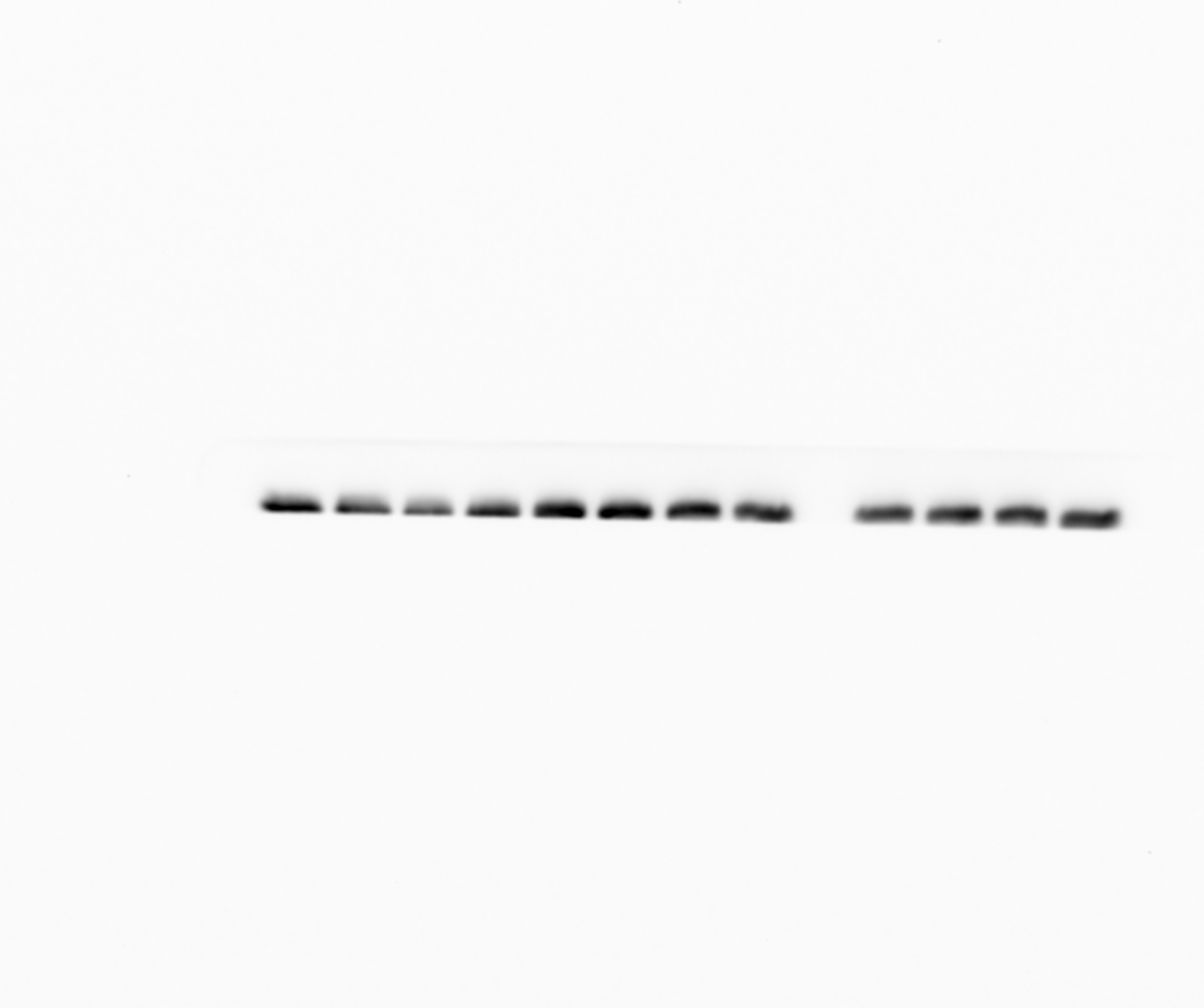

Supplement: Supplementary file 5 — Source data Fig. 2 [file 44319_2024_352_MOESM5_ESM.zip › Figure 2/2H/western GAPDH.tif]

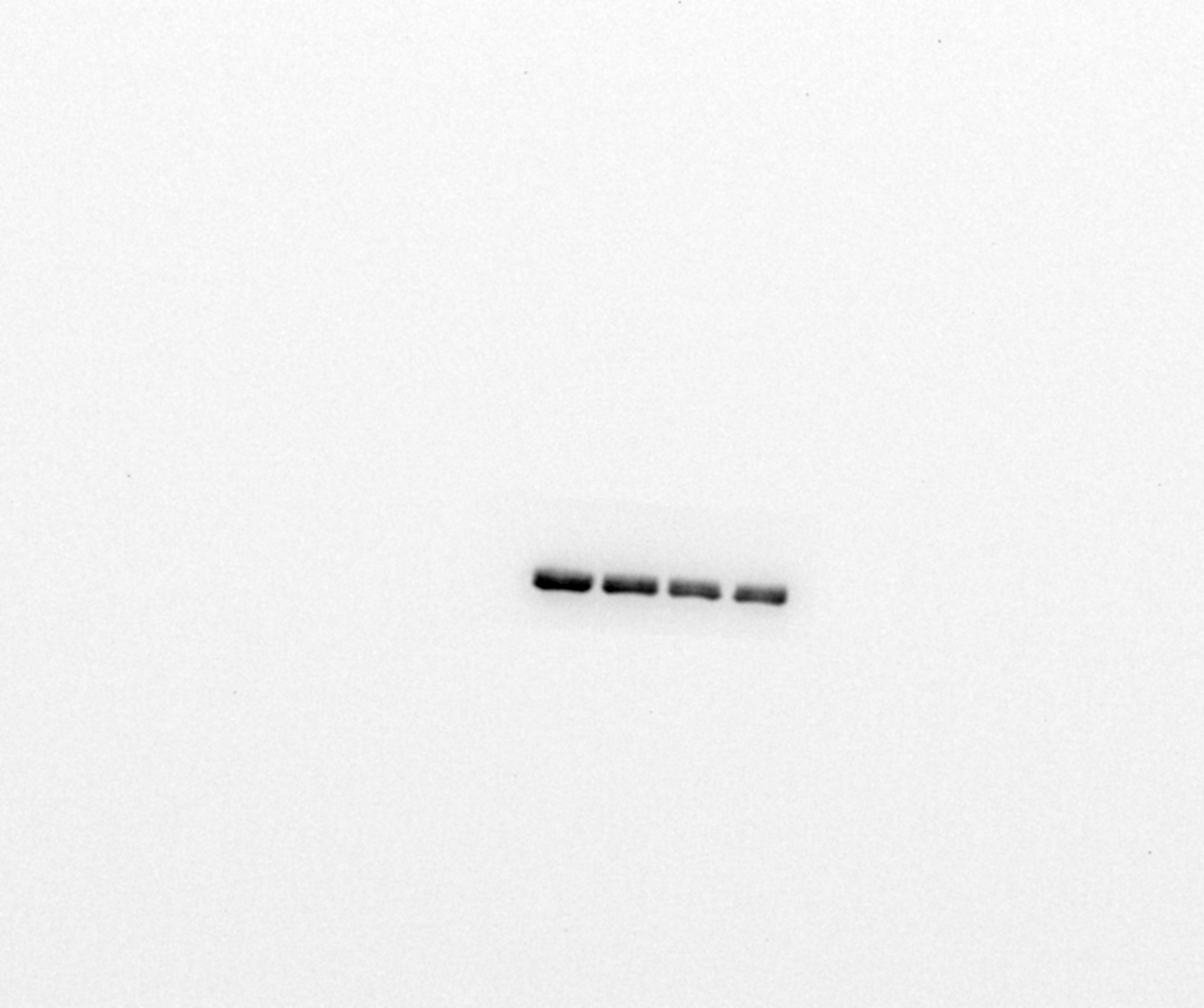

Supplement: Supplementary file 5 — Source data Fig. 2 [file 44319_2024_352_MOESM5_ESM.zip › Figure 2/2H/western IRF3.tif]

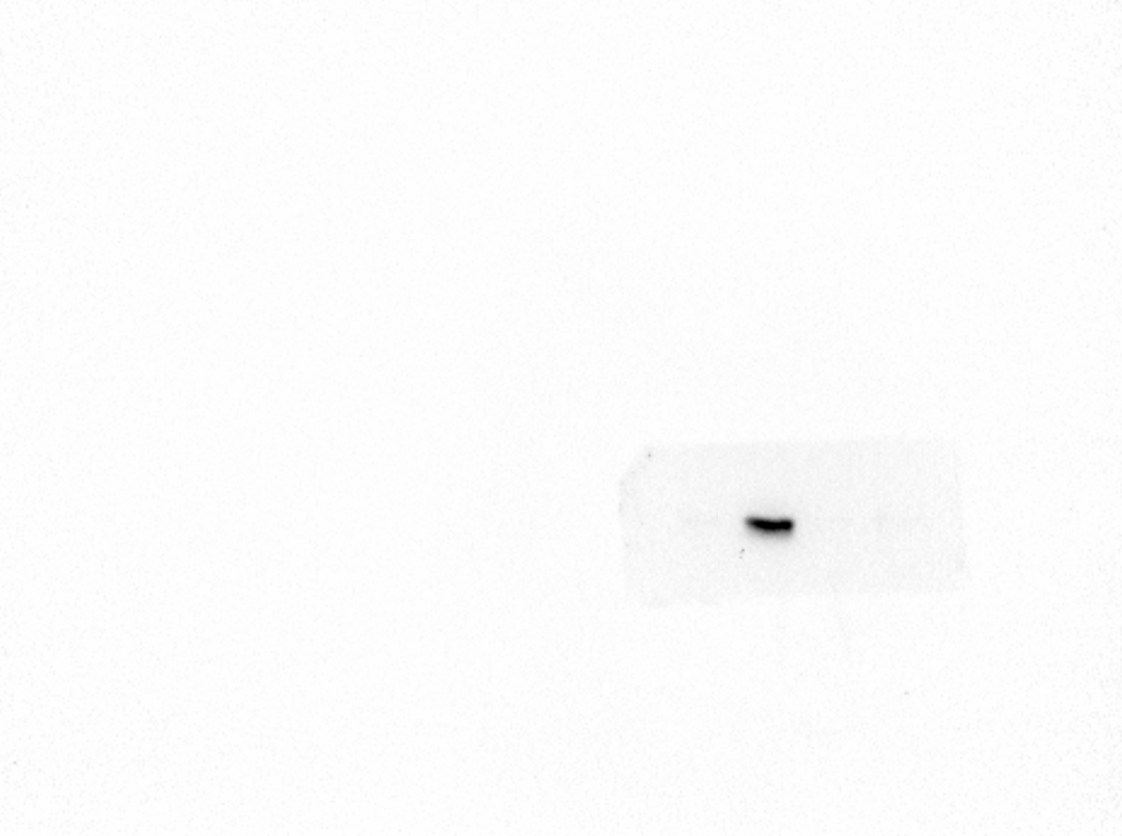

Supplement: Supplementary file 5 — Source data Fig. 2 [file 44319_2024_352_MOESM5_ESM.zip › Figure 2/2H/western p-IRF3.tif]

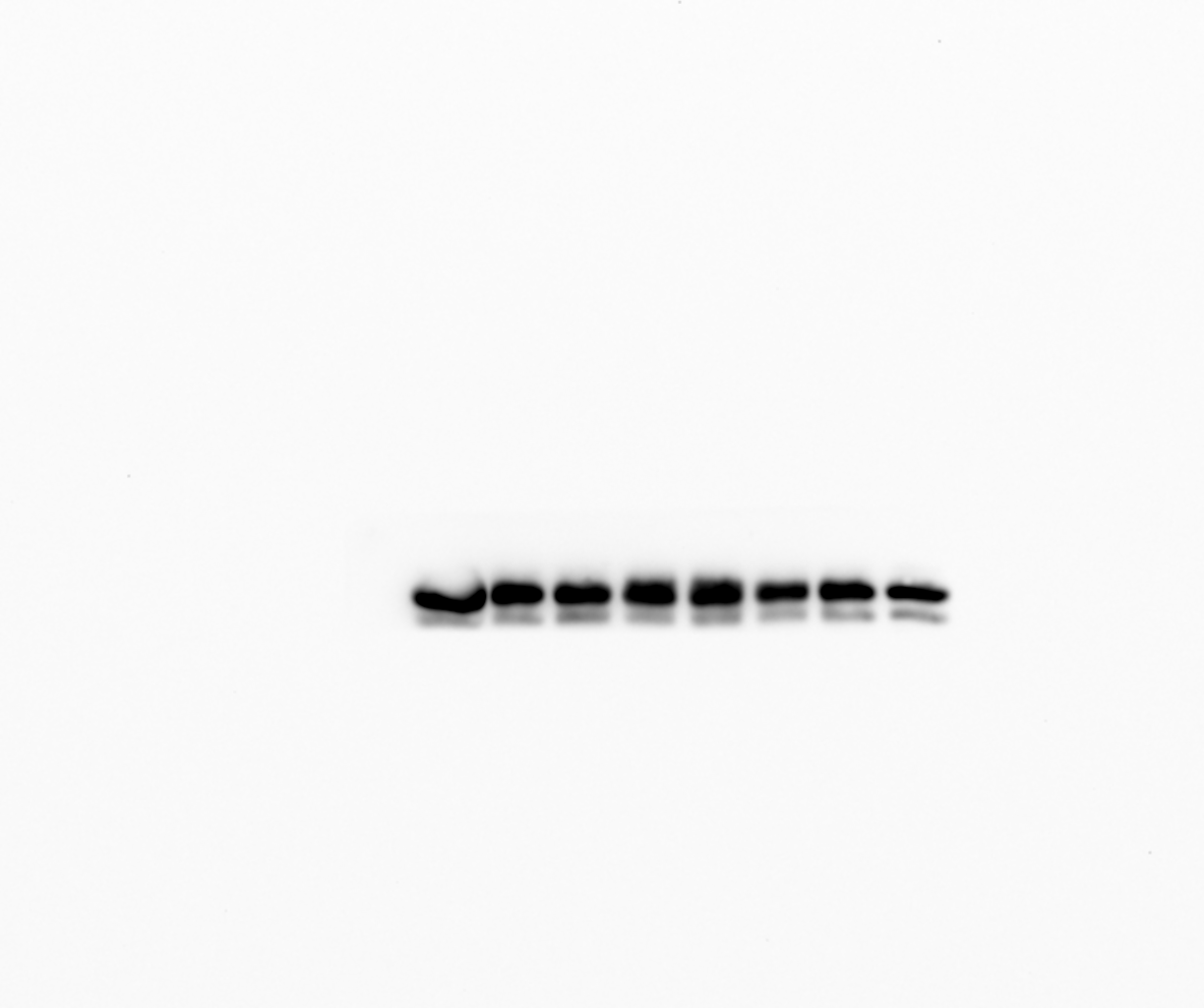

Supplement: Supplementary file 5 — Source data Fig. 2 [file 44319_2024_352_MOESM5_ESM.zip › Figure 2/2I/western GAPDH.tif]

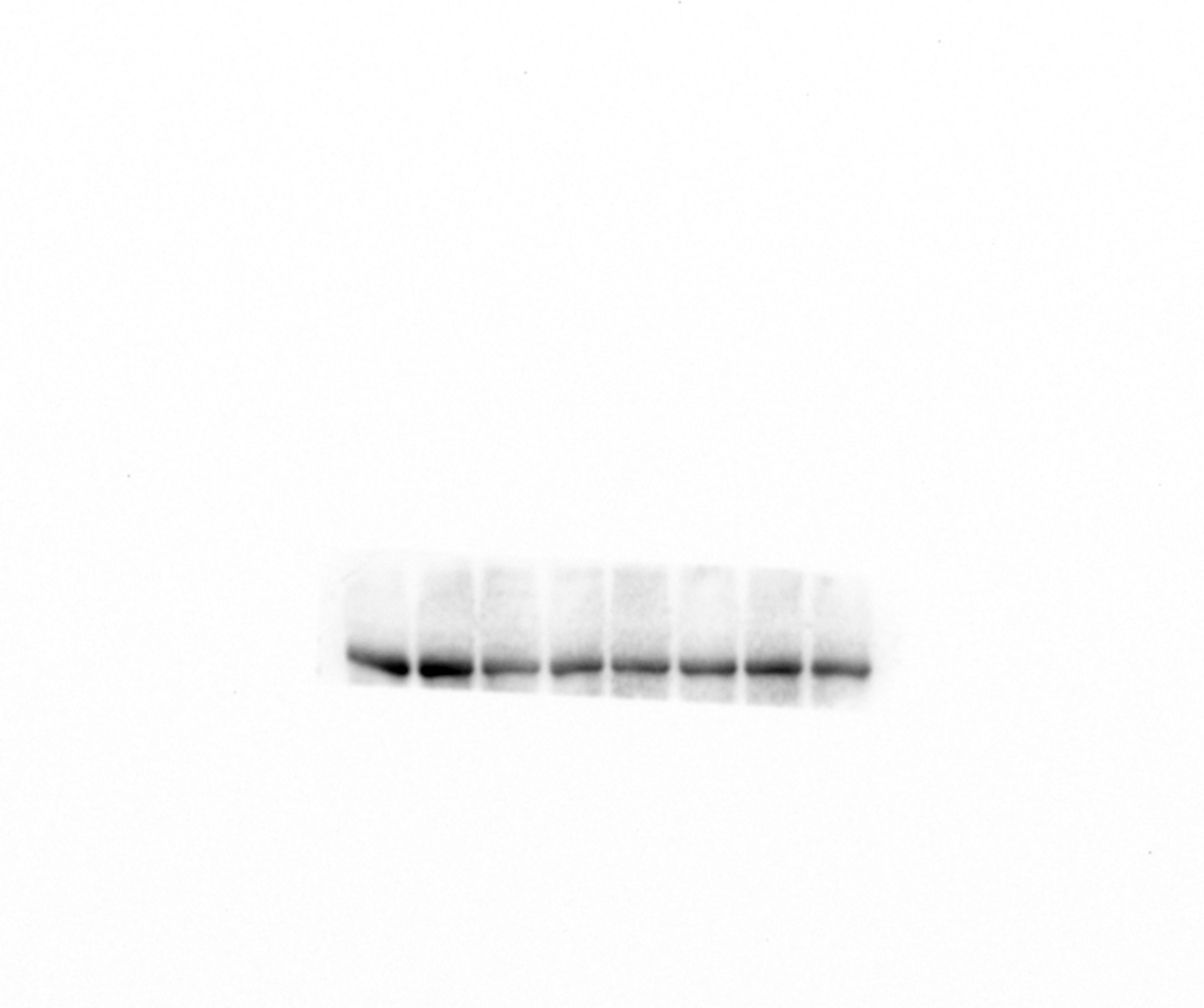

Supplement: Supplementary file 5 — Source data Fig. 2 [file 44319_2024_352_MOESM5_ESM.zip › Figure 2/2I/western IRF3.tif]

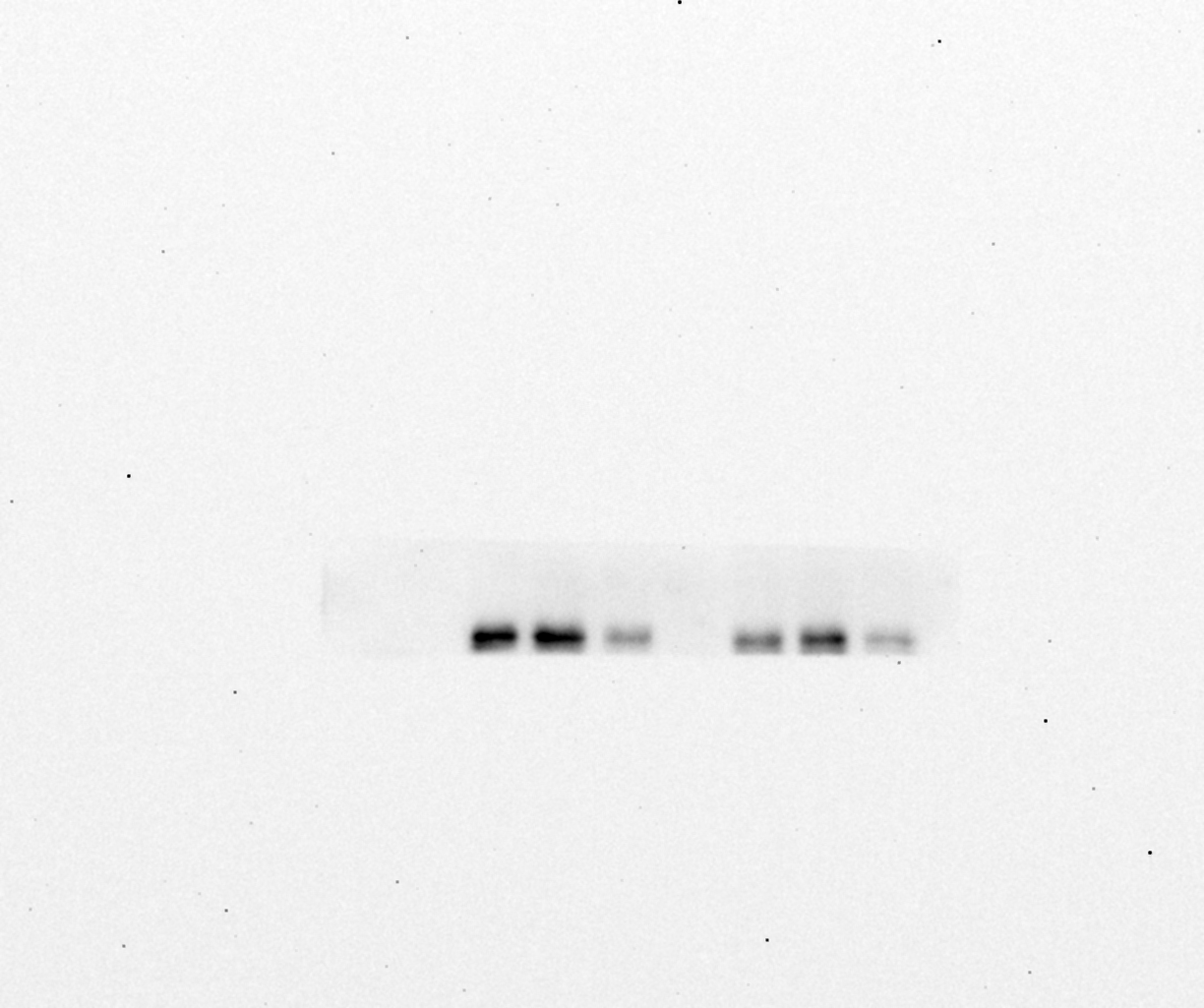

Supplement: Supplementary file 5 — Source data Fig. 2 [file 44319_2024_352_MOESM5_ESM.zip › Figure 2/2I/western p-IRF3.tif]

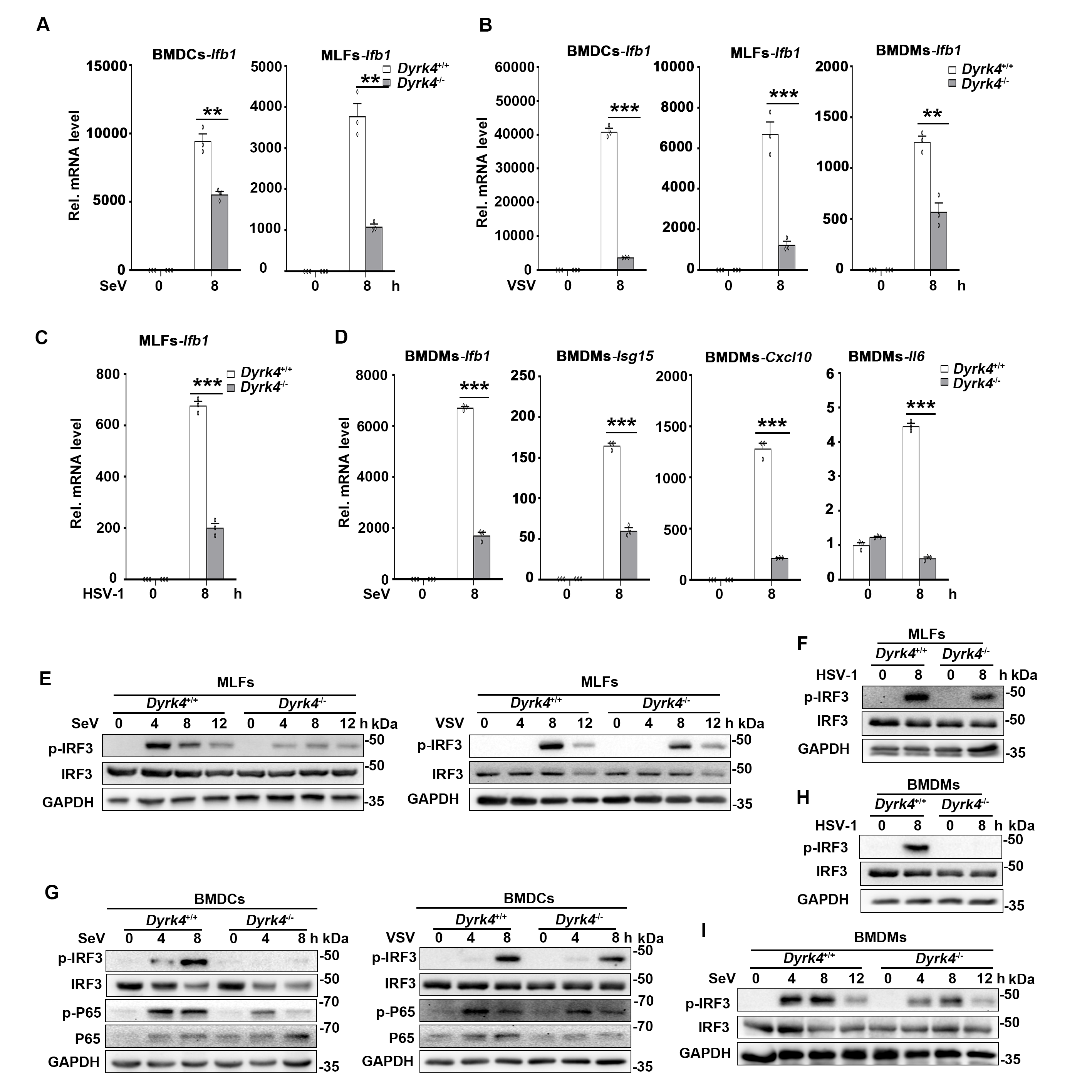

Supplement: Supplementary file 5 — Source data Fig. 2 [file 44319_2024_352_MOESM5_ESM.zip › Figure 2/Figure 2.tif]

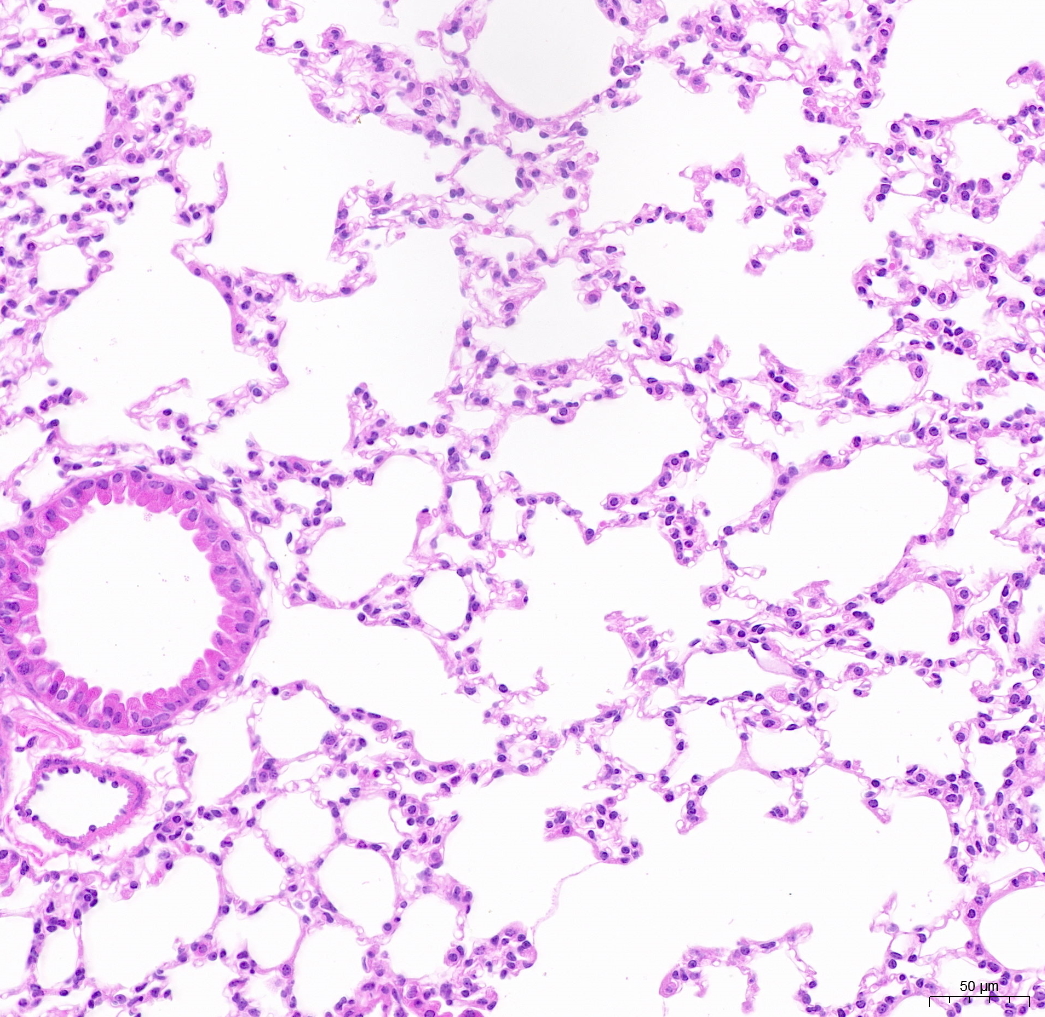

Supplement: Supplementary file 6 — Source data Fig. 3 [file 44319_2024_352_MOESM6_ESM.zip › Figure 3/3E/KO+PBS.jpg]

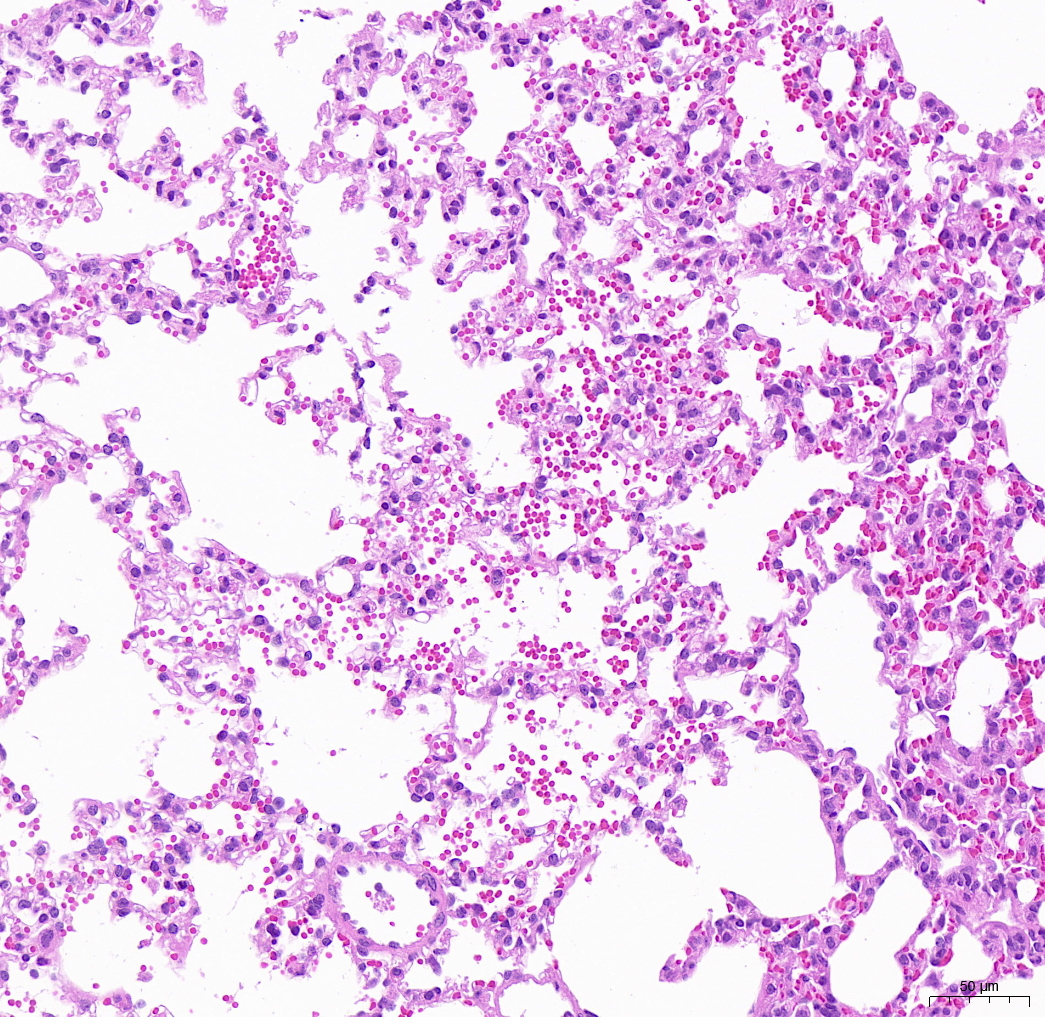

Supplement: Supplementary file 6 — Source data Fig. 3 [file 44319_2024_352_MOESM6_ESM.zip › Figure 3/3E/KO+VSV.jpg]

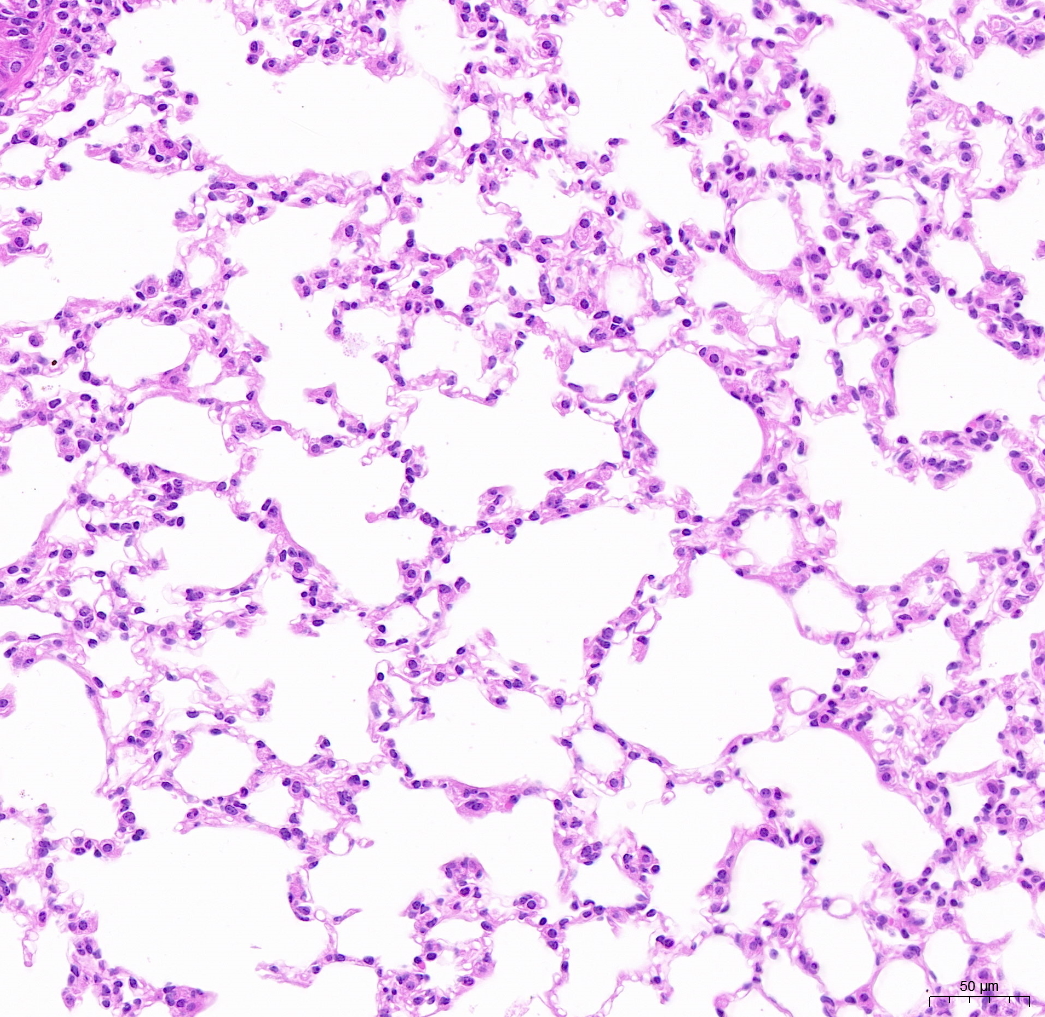

Supplement: Supplementary file 6 — Source data Fig. 3 [file 44319_2024_352_MOESM6_ESM.zip › Figure 3/3E/WT+PBS.jpg]

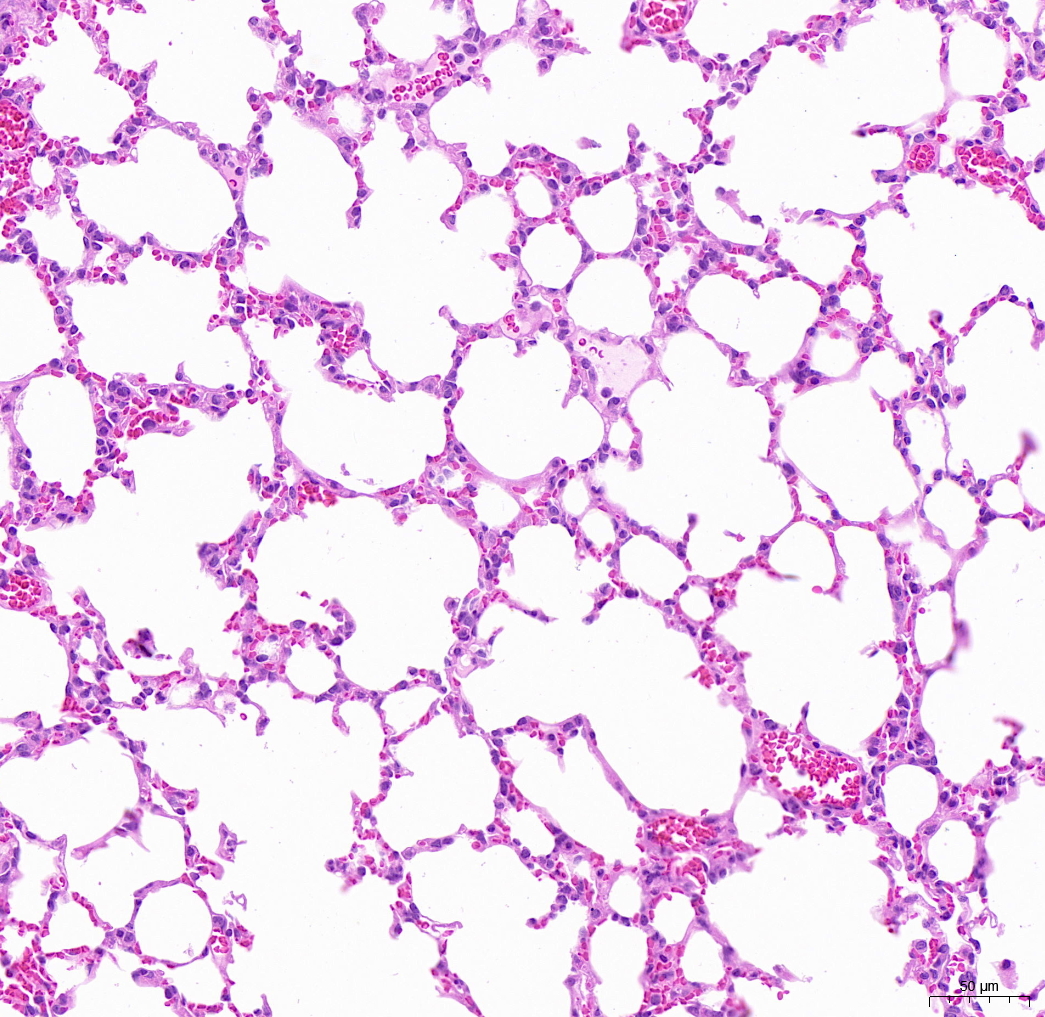

Supplement: Supplementary file 6 — Source data Fig. 3 [file 44319_2024_352_MOESM6_ESM.zip › Figure 3/3E/WT+VSV.jpg]

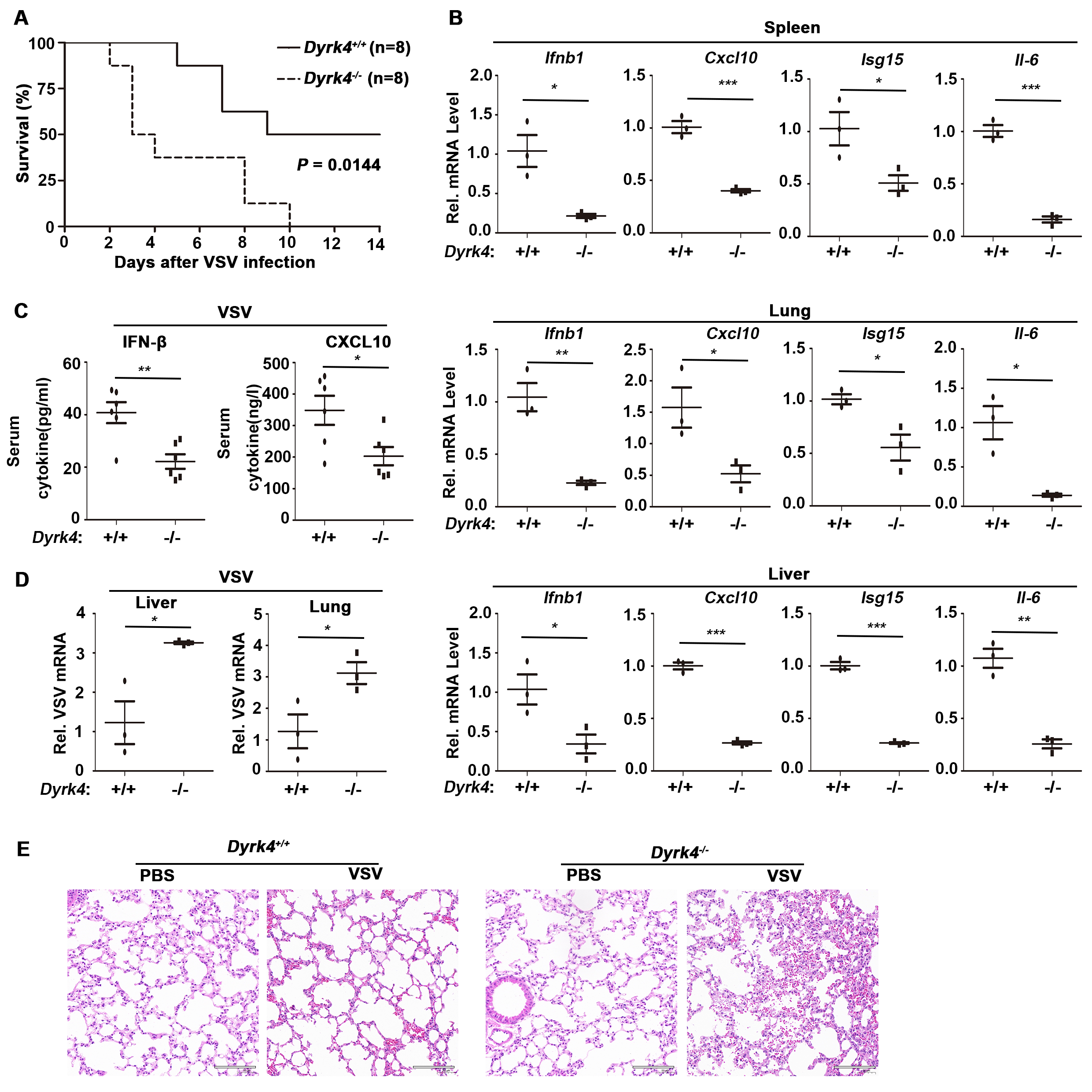

Supplement: Supplementary file 6 — Source data Fig. 3 [file 44319_2024_352_MOESM6_ESM.zip › Figure 3/Figure 3.tif]

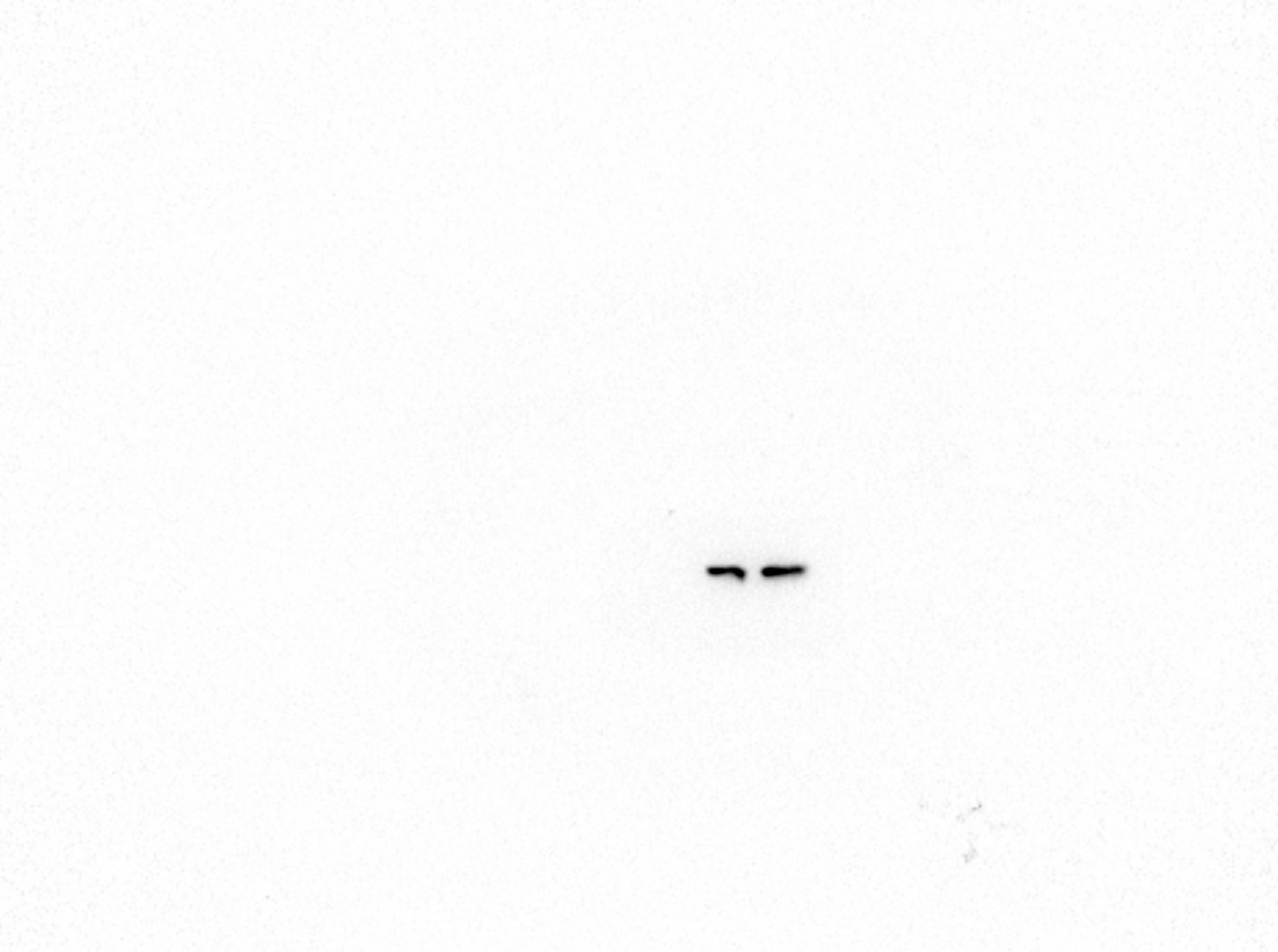

Supplement: Supplementary file 7 — Source data Fig. 4 [file 44319_2024_352_MOESM7_ESM.zip › Figure 4/4B/IRF3/western Flag-IRF3.tif]

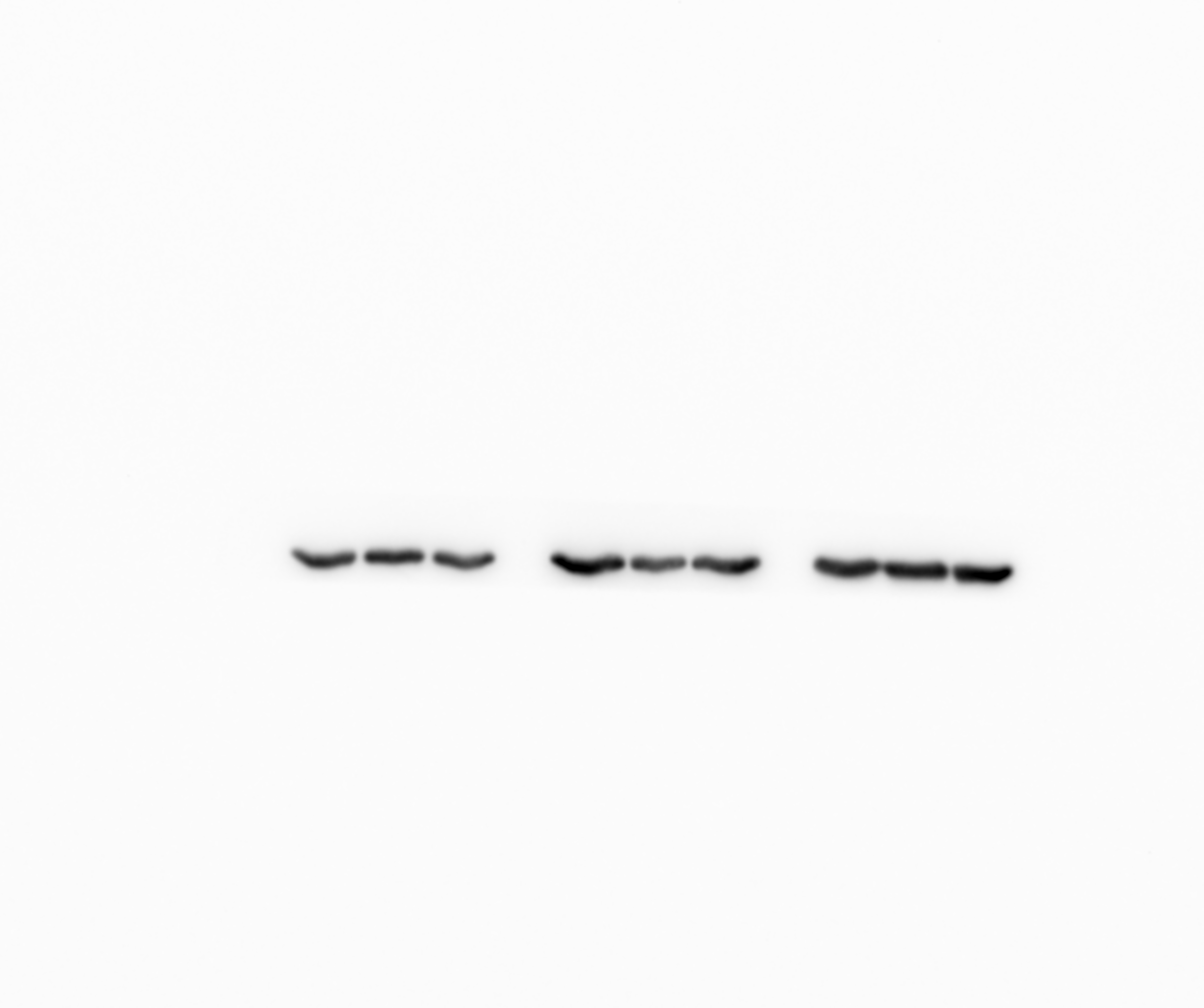

Supplement: Supplementary file 7 — Source data Fig. 4 [file 44319_2024_352_MOESM7_ESM.zip › Figure 4/4B/IRF3/western GAPDH.tif]

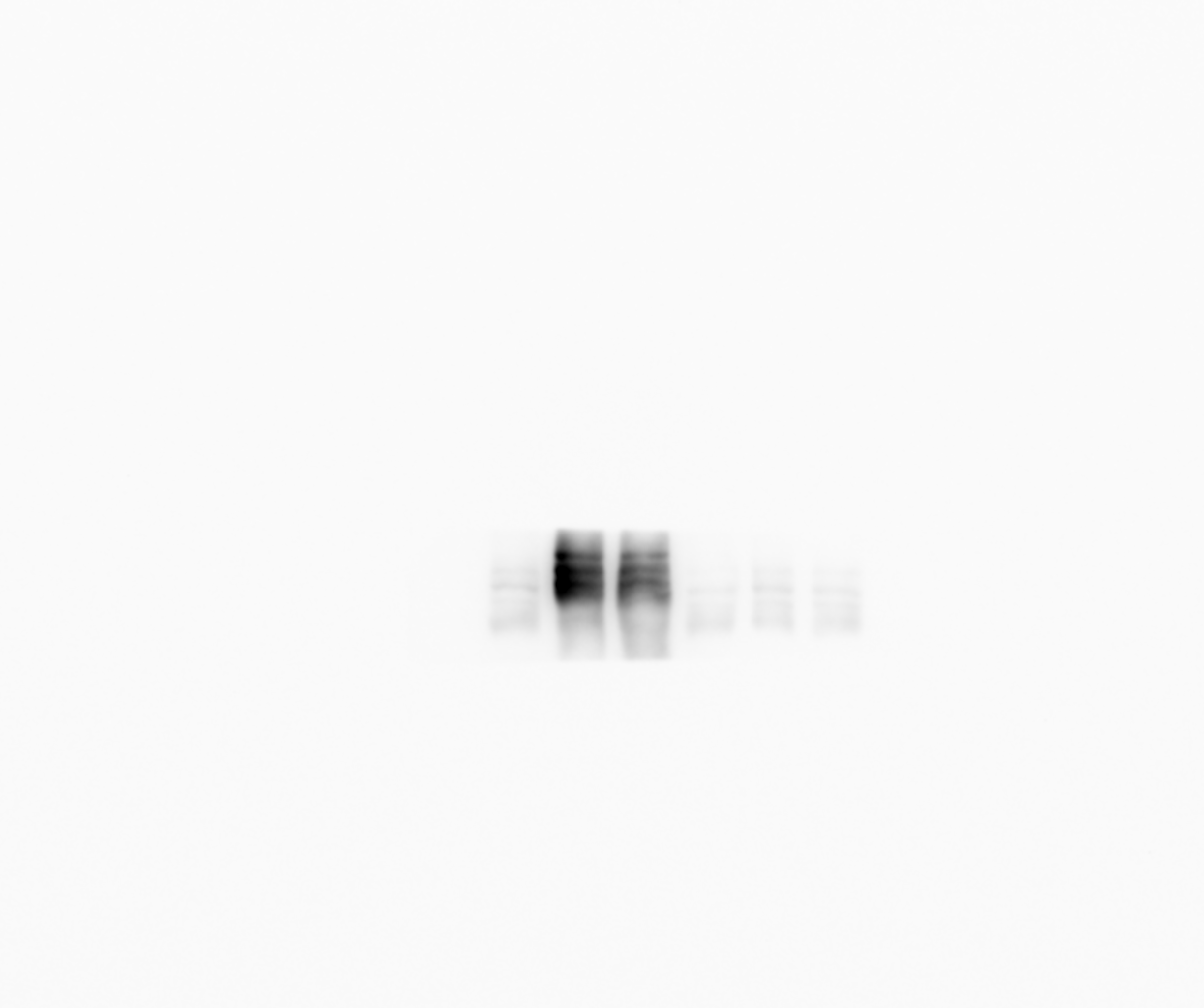

Supplement: Supplementary file 7 — Source data Fig. 4 [file 44319_2024_352_MOESM7_ESM.zip › Figure 4/4B/IRF3/western IRF3.tif]

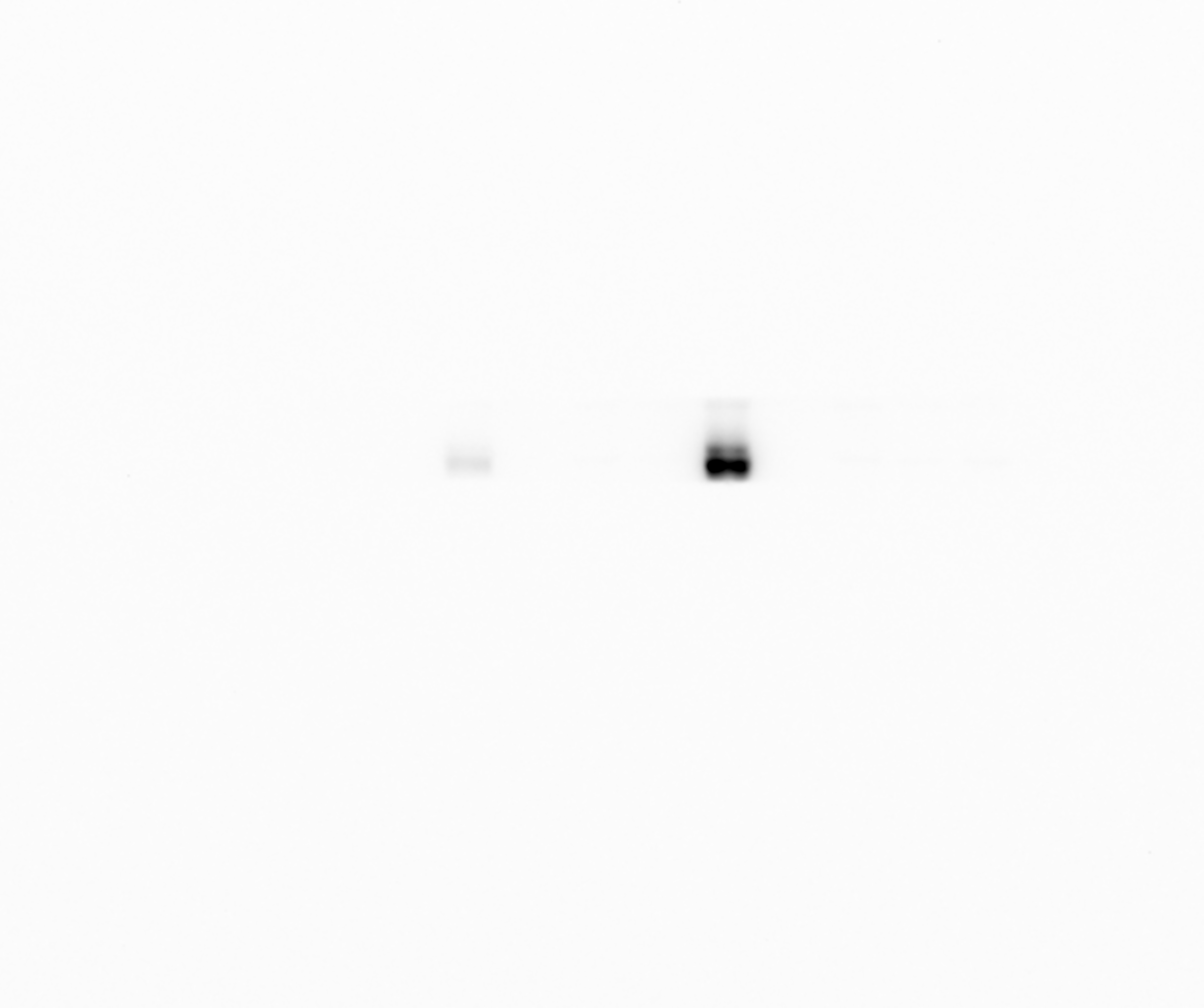

Supplement: Supplementary file 7 — Source data Fig. 4 [file 44319_2024_352_MOESM7_ESM.zip › Figure 4/4B/IRF3/western myc-DYRK4-.tif]

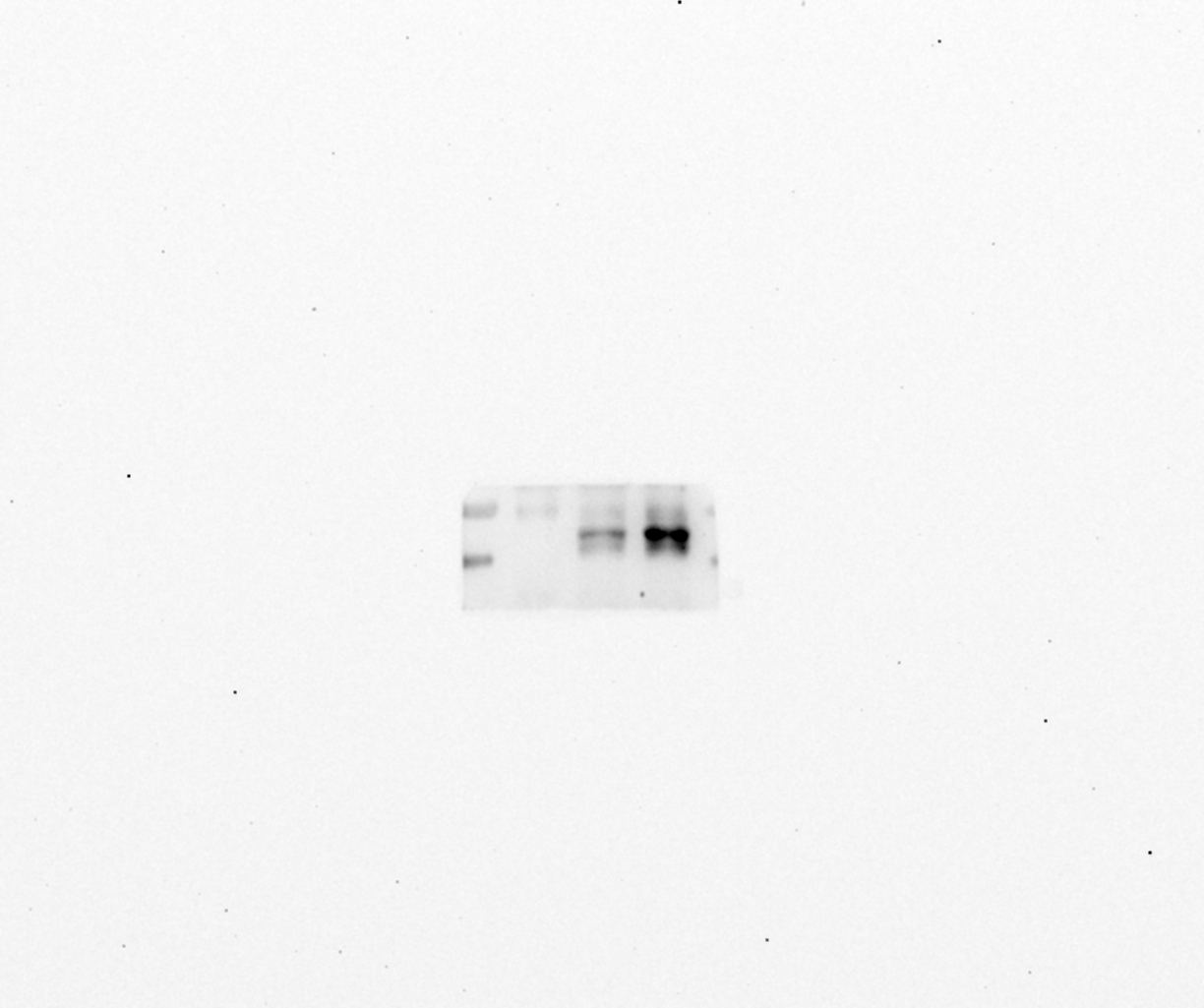

Supplement: Supplementary file 7 — Source data Fig. 4 [file 44319_2024_352_MOESM7_ESM.zip › Figure 4/4B/IRF3/western p-IRF3.tif]

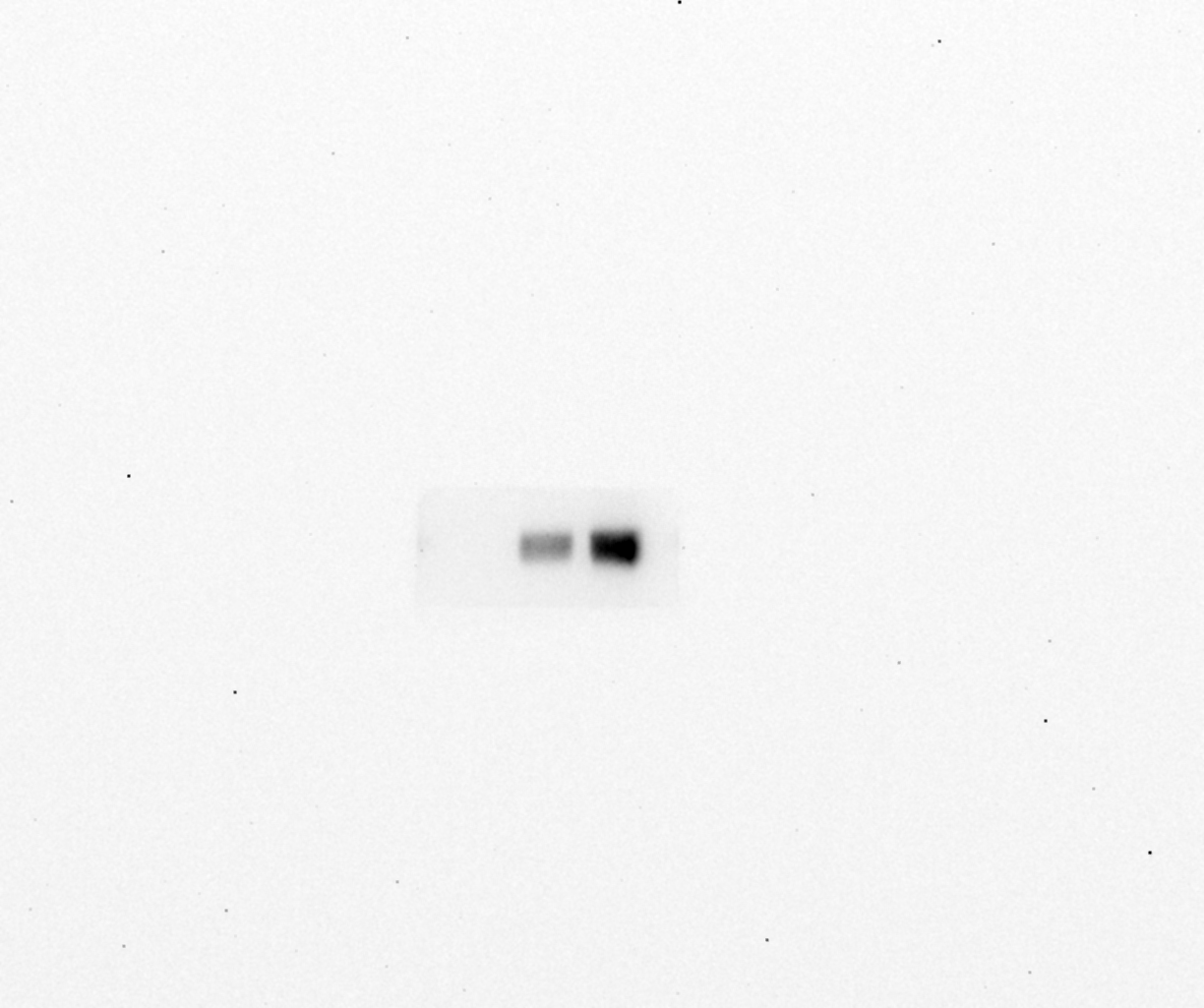

Supplement: Supplementary file 7 — Source data Fig. 4 [file 44319_2024_352_MOESM7_ESM.zip › Figure 4/4B/MAVS/western p-IRF3.tif]

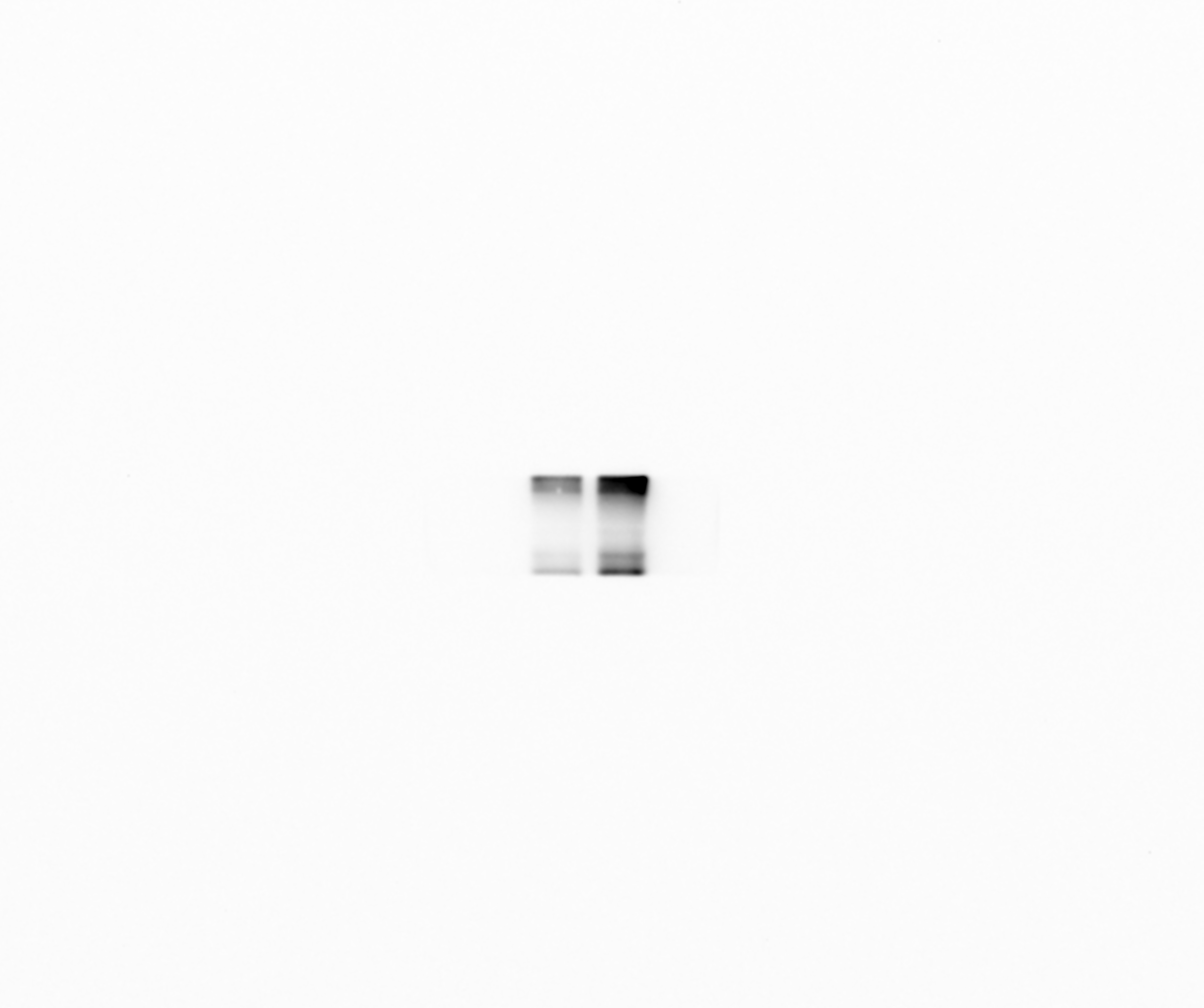

Supplement: Supplementary file 7 — Source data Fig. 4 [file 44319_2024_352_MOESM7_ESM.zip › Figure 4/4B/MAVS/western Falg-MAVS.tif]

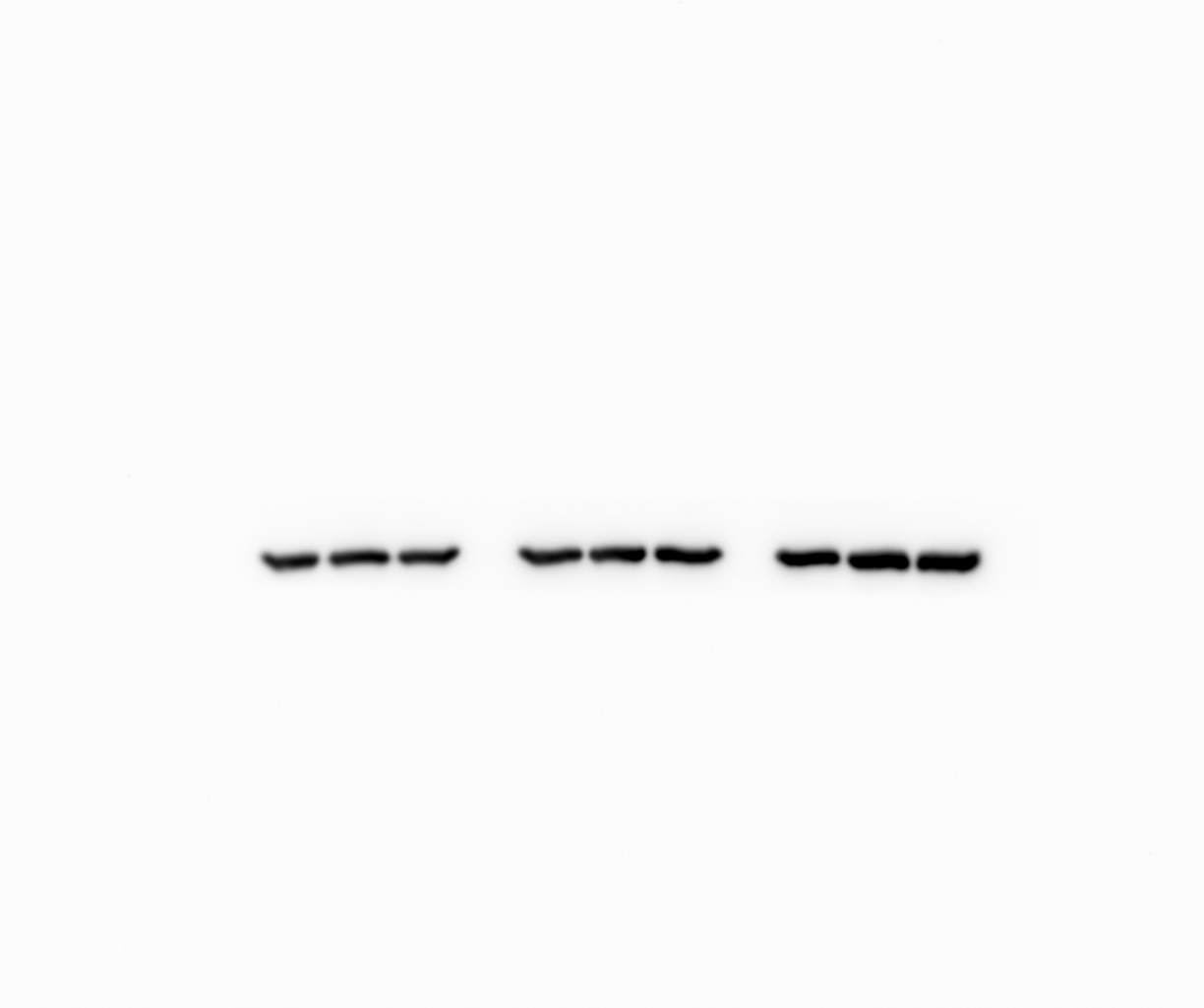

Supplement: Supplementary file 7 — Source data Fig. 4 [file 44319_2024_352_MOESM7_ESM.zip › Figure 4/4B/MAVS/western GAPDH.tif]

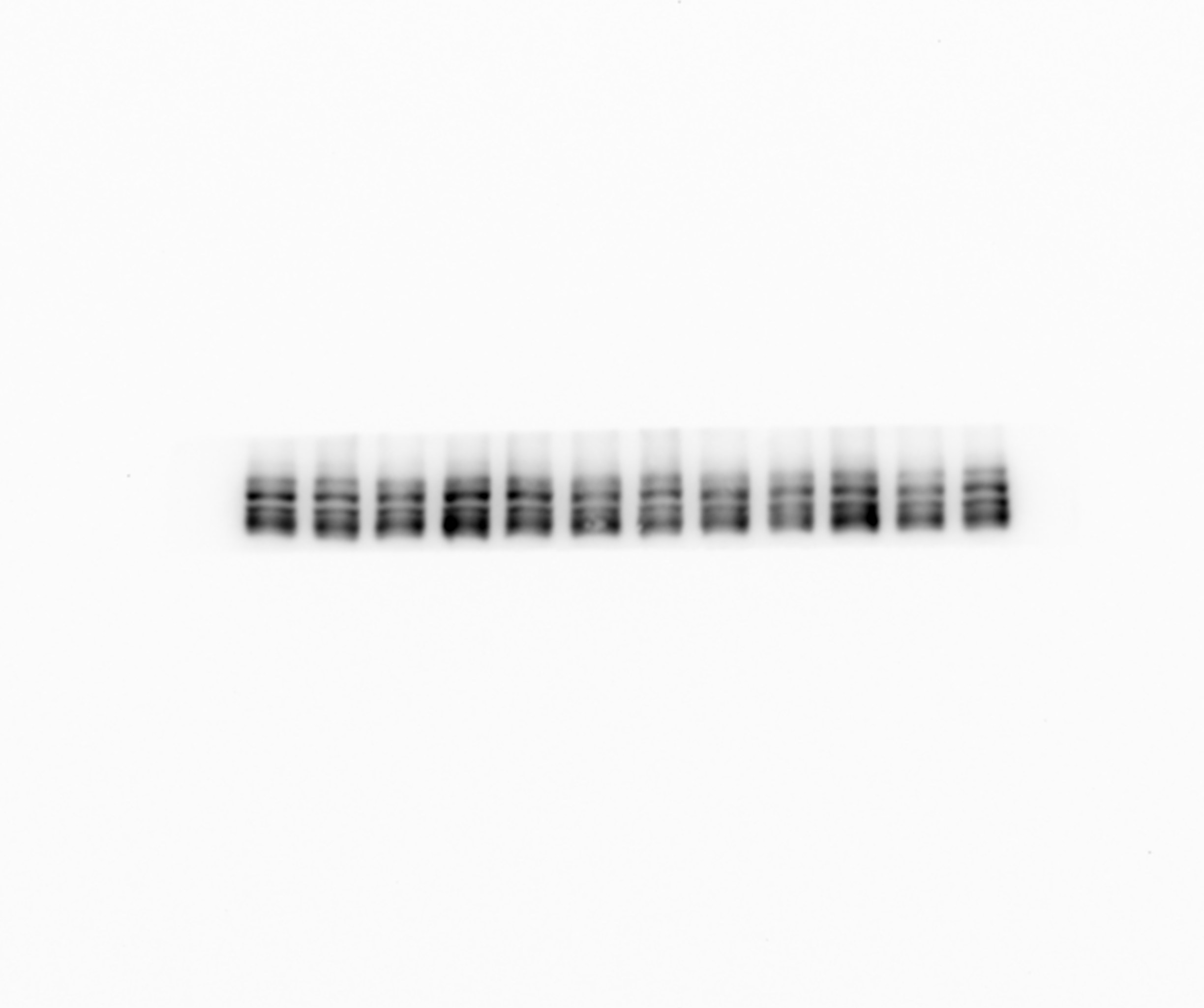

Supplement: Supplementary file 7 — Source data Fig. 4 [file 44319_2024_352_MOESM7_ESM.zip › Figure 4/4B/MAVS/western IRF3.tif]

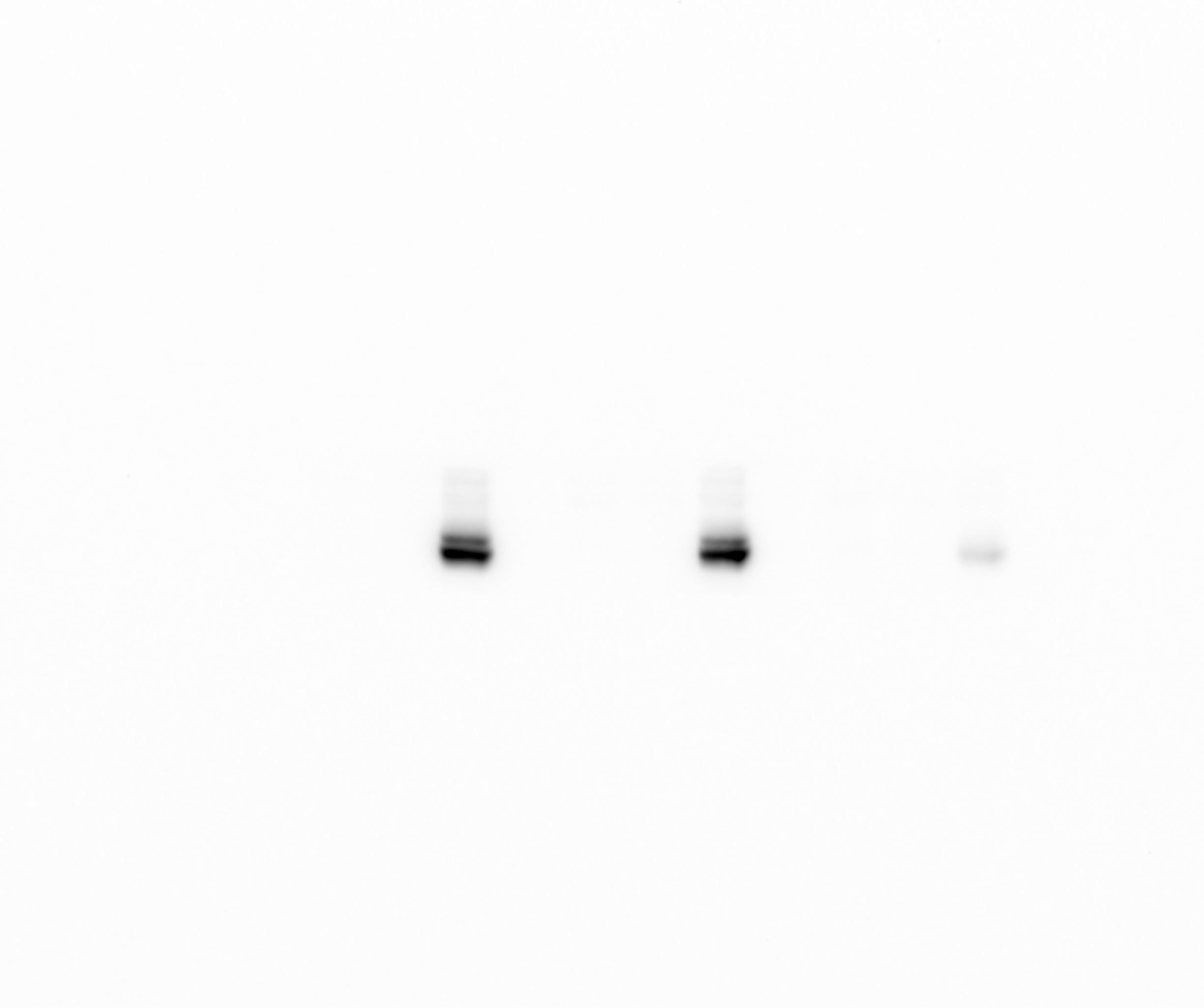

Supplement: Supplementary file 7 — Source data Fig. 4 [file 44319_2024_352_MOESM7_ESM.zip › Figure 4/4B/MAVS/western myc- DYRK4.tif]

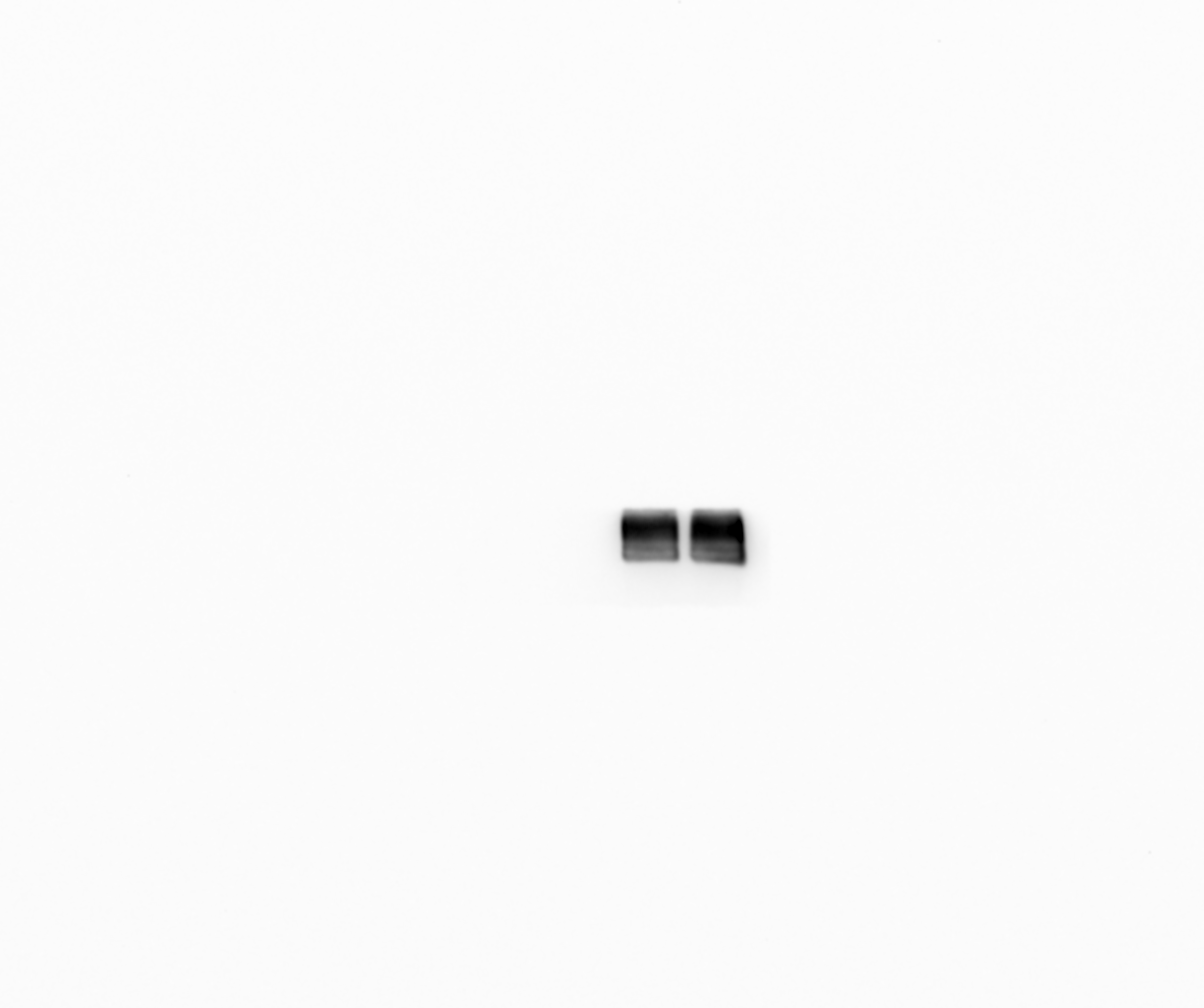

Supplement: Supplementary file 7 — Source data Fig. 4 [file 44319_2024_352_MOESM7_ESM.zip › Figure 4/4B/RIG-I/western Flag-RIG-I.tif]

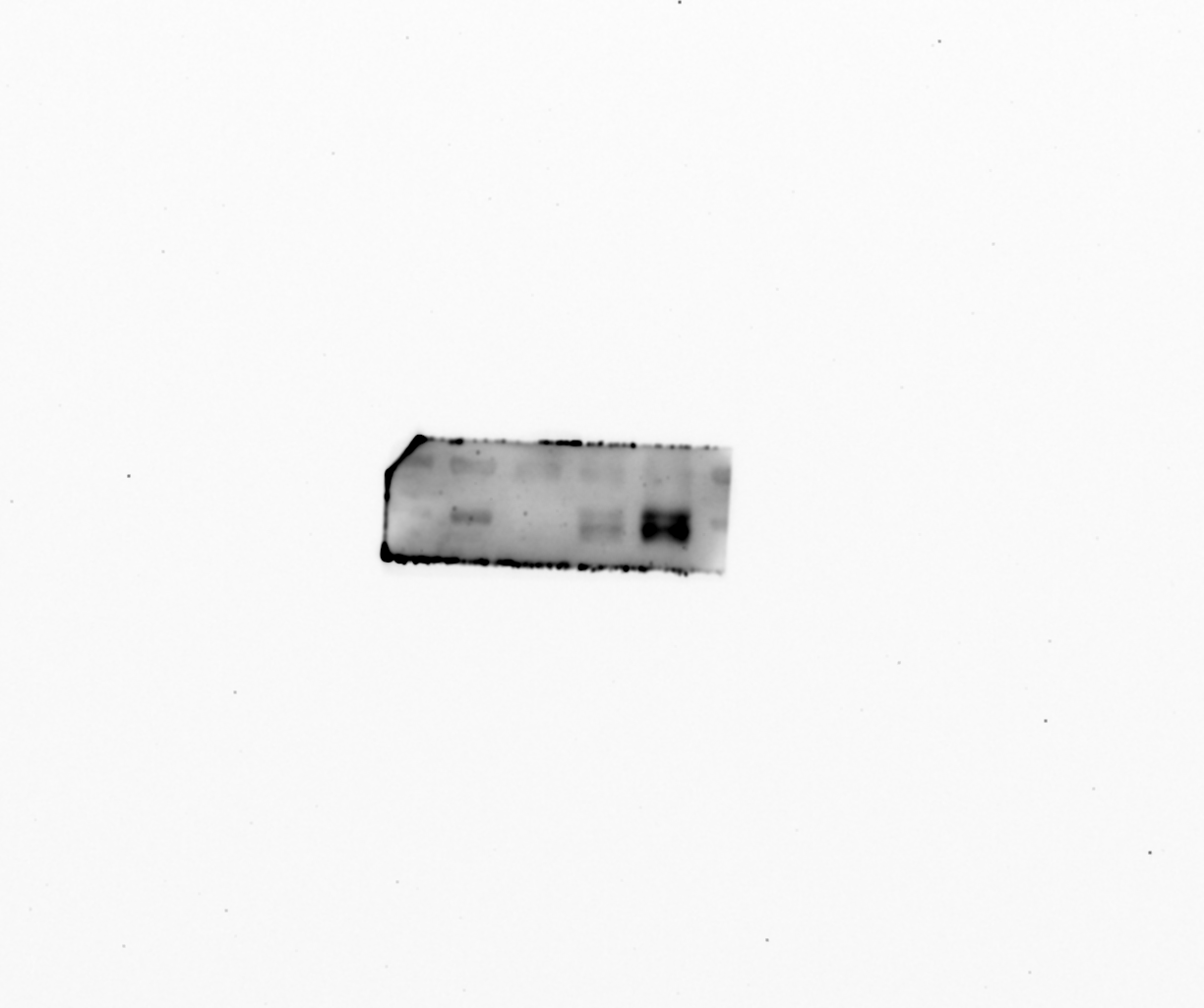

Supplement: Supplementary file 7 — Source data Fig. 4 [file 44319_2024_352_MOESM7_ESM.zip › Figure 4/4B/RIG-I/western p-IRF3.tif]

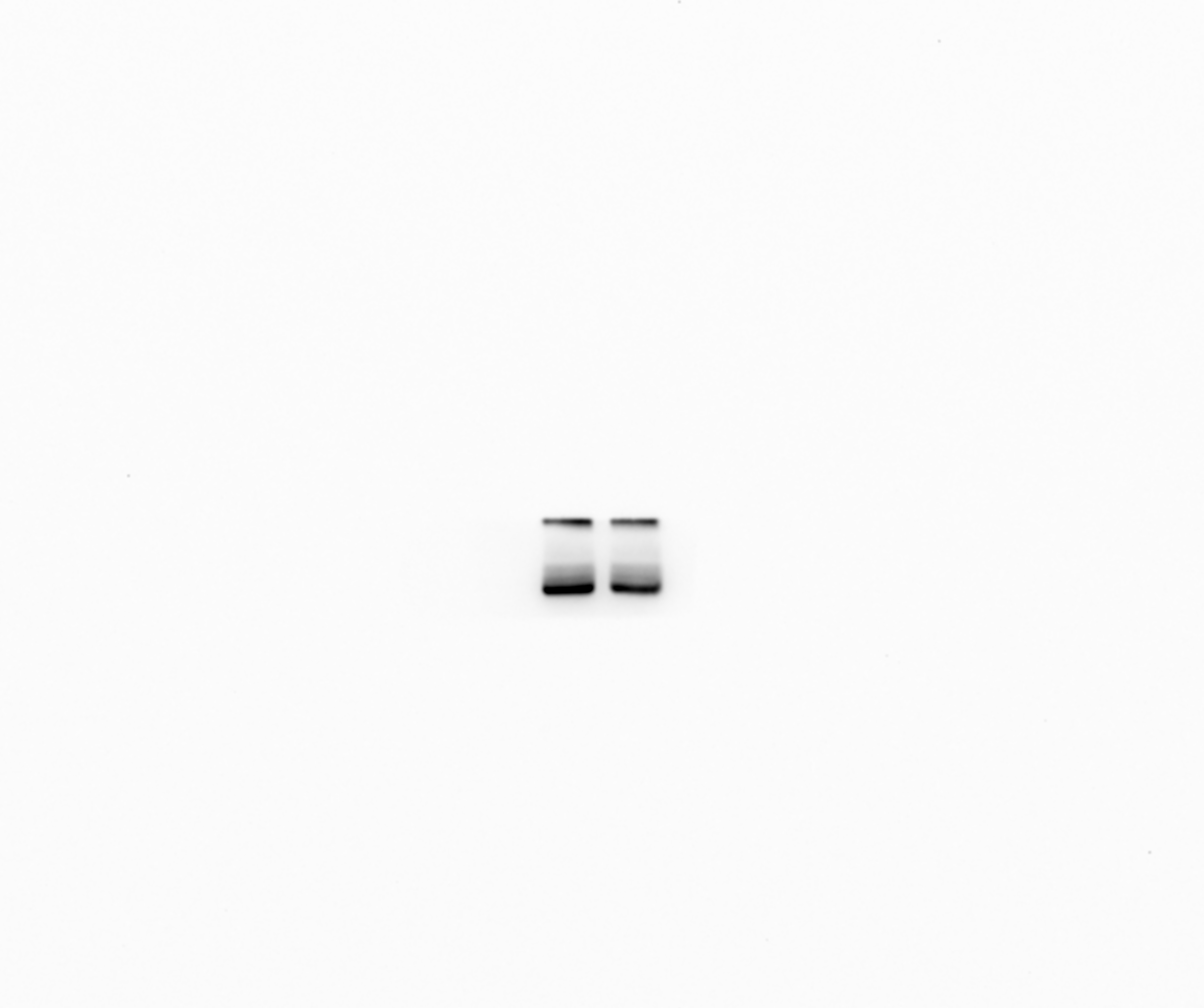

Supplement: Supplementary file 7 — Source data Fig. 4 [file 44319_2024_352_MOESM7_ESM.zip › Figure 4/4B/TBK1/western Falg-TBK1.tif]

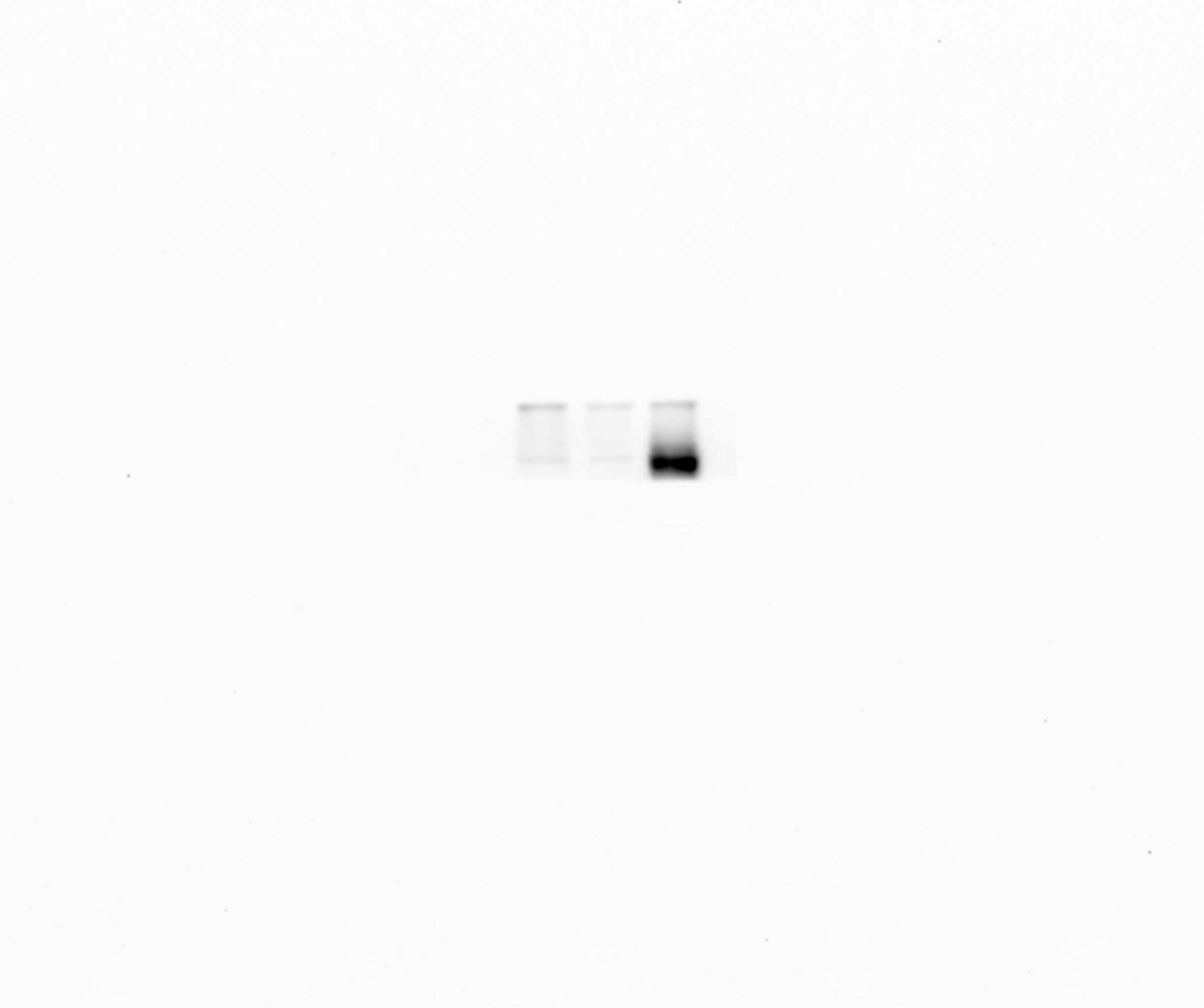

Supplement: Supplementary file 7 — Source data Fig. 4 [file 44319_2024_352_MOESM7_ESM.zip › Figure 4/4B/TBK1/western myc-DYRK4.tif]

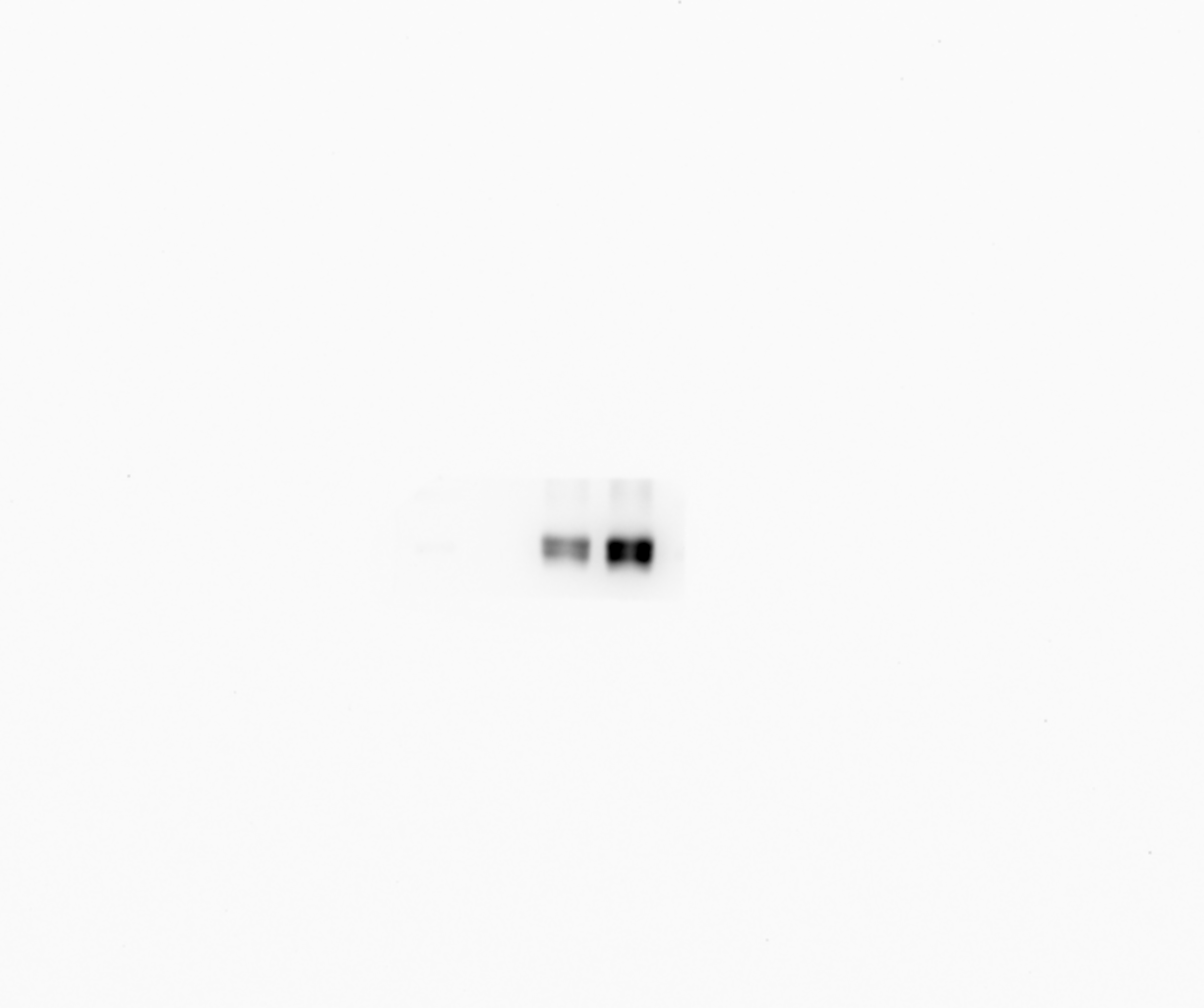

Supplement: Supplementary file 7 — Source data Fig. 4 [file 44319_2024_352_MOESM7_ESM.zip › Figure 4/4B/TBK1/western p-IRF3.tif]

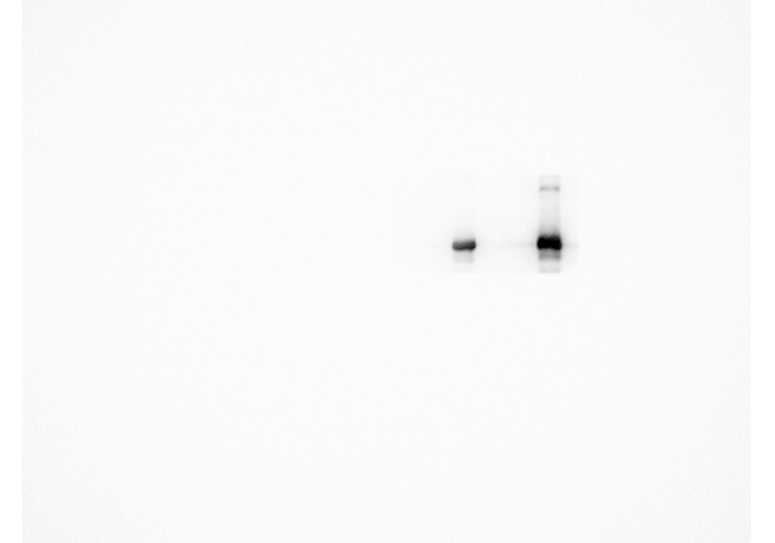

Supplement: Supplementary file 7 — Source data Fig. 4 [file 44319_2024_352_MOESM7_ESM.zip › Figure 4/4C/western Flag Input-IP.tif]

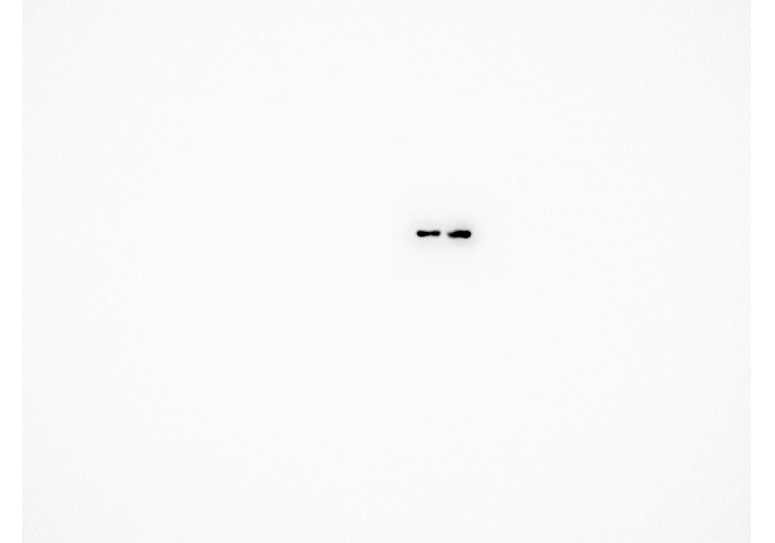

Supplement: Supplementary file 7 — Source data Fig. 4 [file 44319_2024_352_MOESM7_ESM.zip › Figure 4/4C/western GAPDH.tif]

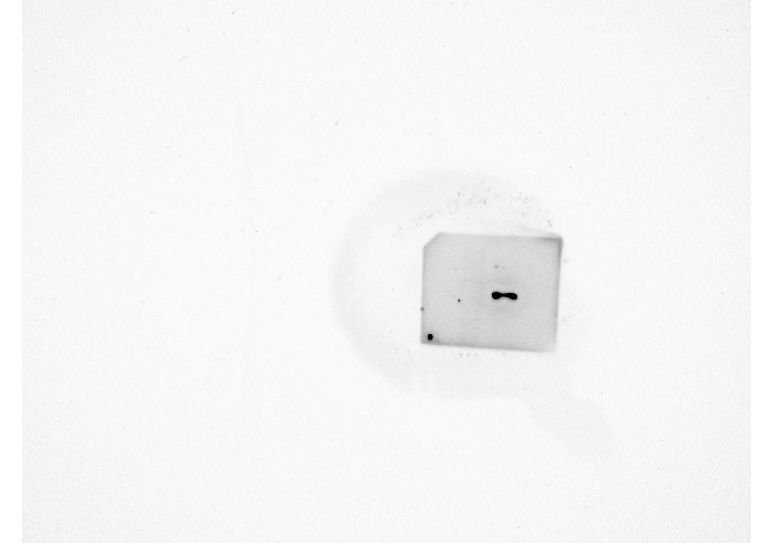

Supplement: Supplementary file 7 — Source data Fig. 4 [file 44319_2024_352_MOESM7_ESM.zip › Figure 4/4C/western myc IP.tif]

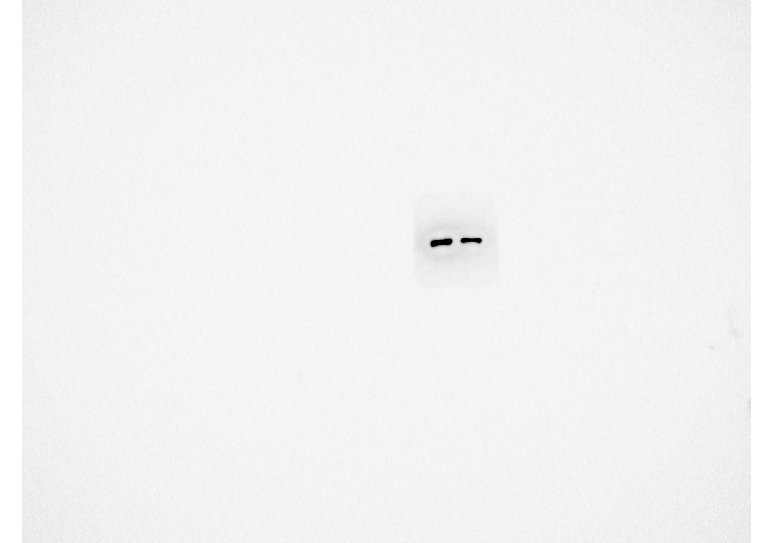

Supplement: Supplementary file 7 — Source data Fig. 4 [file 44319_2024_352_MOESM7_ESM.zip › Figure 4/4C/western myc Input.tif]

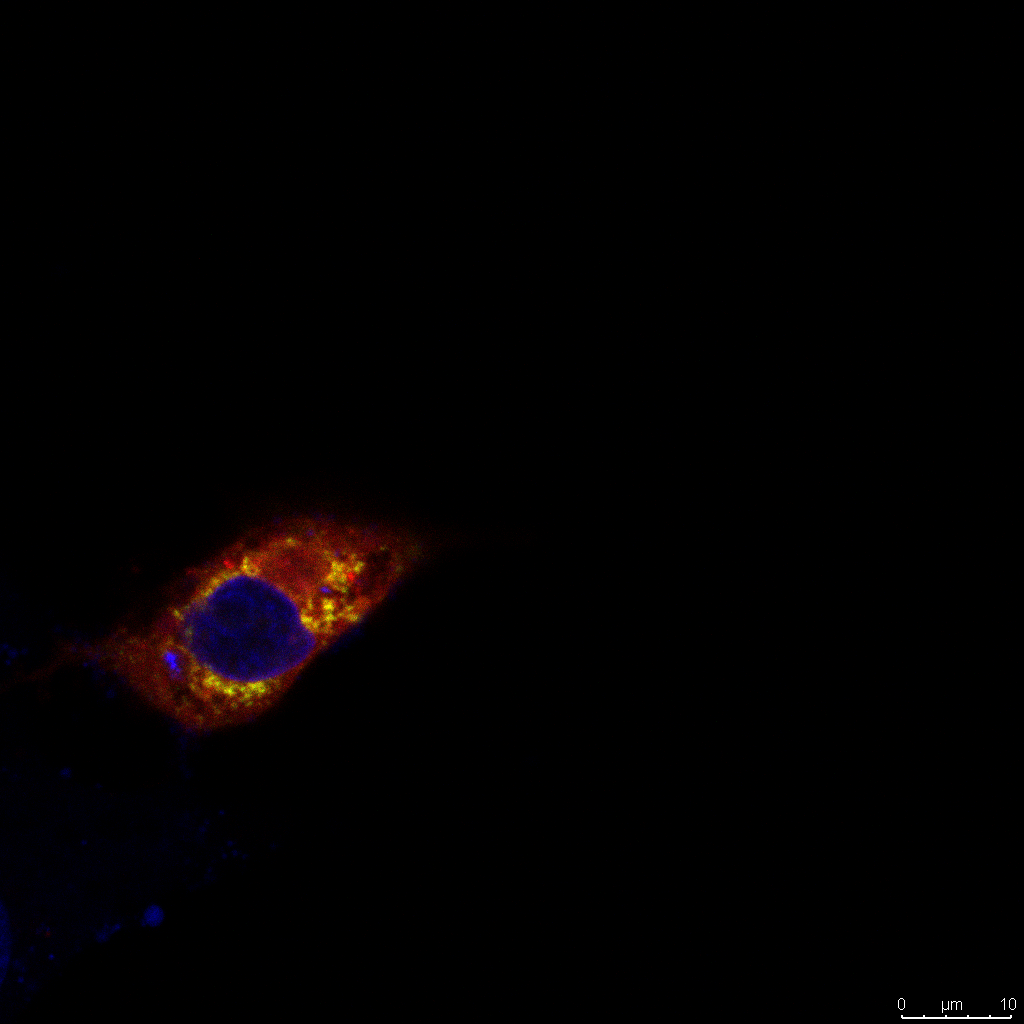

Supplement: Supplementary file 7 — Source data Fig. 4 [file 44319_2024_352_MOESM7_ESM.zip › Figure 4/4D/Micr.image DY4-GFP+IRF3-mcherry_Series017.tif]

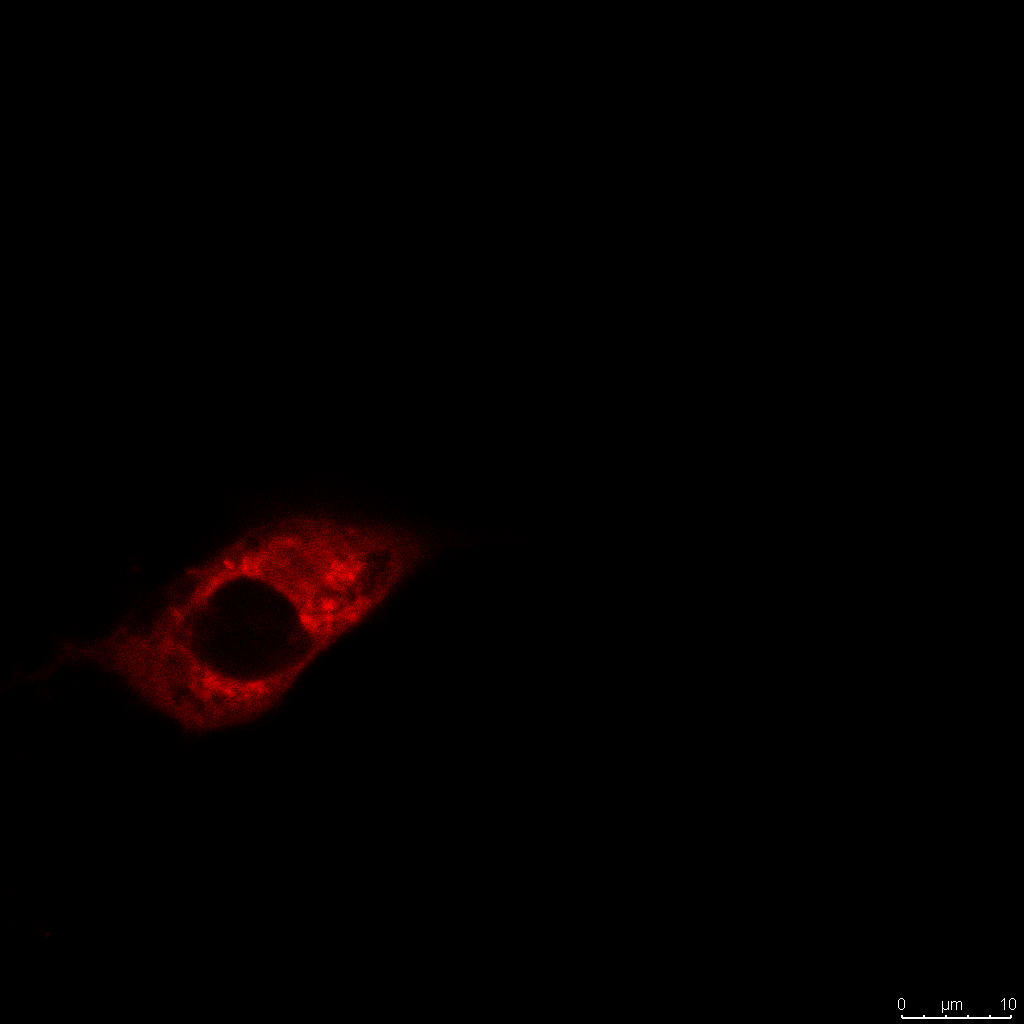

Supplement: Supplementary file 7 — Source data Fig. 4 [file 44319_2024_352_MOESM7_ESM.zip › Figure 4/4D/Micr.image DY4-GFP+IRF3-mcherry_Series017_ch00.tif]

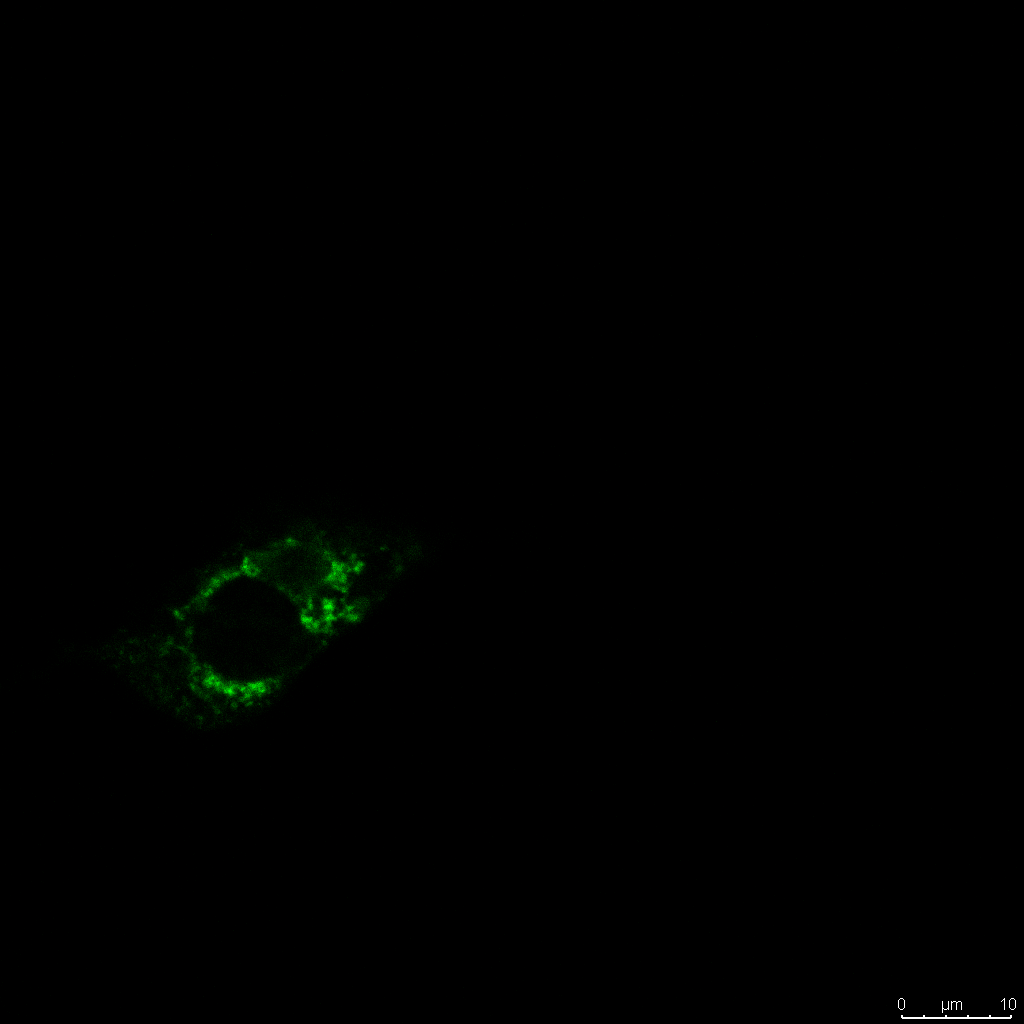

Supplement: Supplementary file 7 — Source data Fig. 4 [file 44319_2024_352_MOESM7_ESM.zip › Figure 4/4D/Micr.image DY4-GFP+IRF3-mcherry_Series017_ch01.tif]

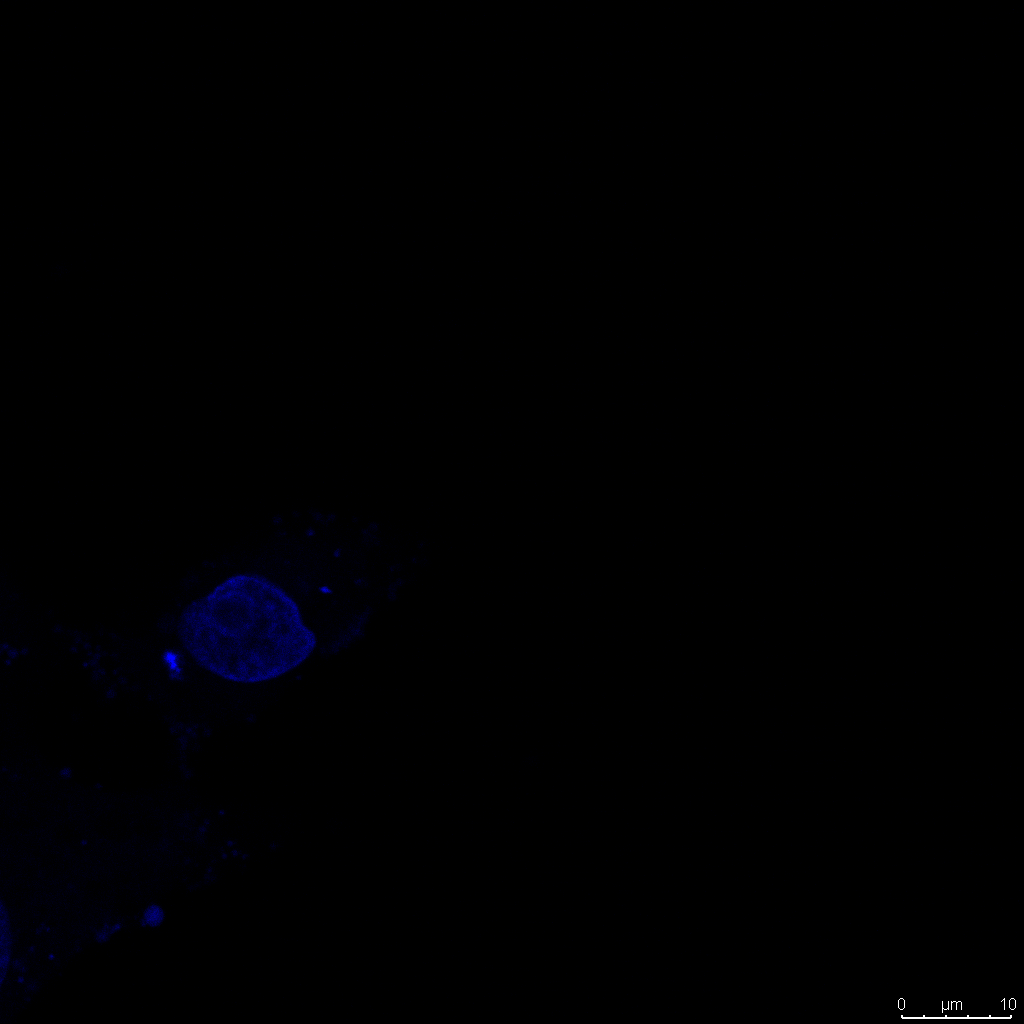

Supplement: Supplementary file 7 — Source data Fig. 4 [file 44319_2024_352_MOESM7_ESM.zip › Figure 4/4D/Micr.image DY4-GFP+IRF3-mcherry_Series017_ch02.tif]

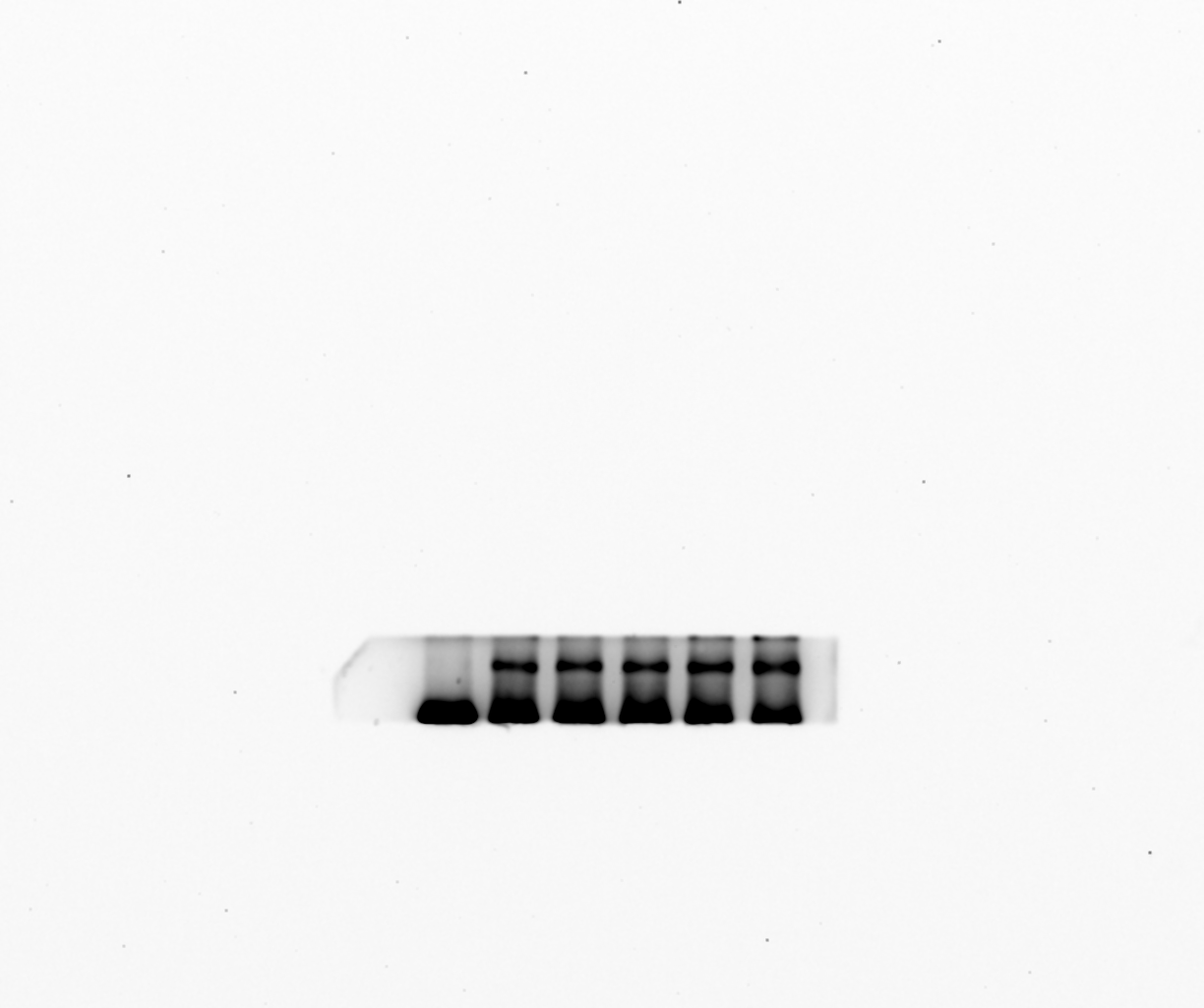

Supplement: Supplementary file 7 — Source data Fig. 4 [file 44319_2024_352_MOESM7_ESM.zip › Figure 4/4E/western DYRK4 IP.tif]

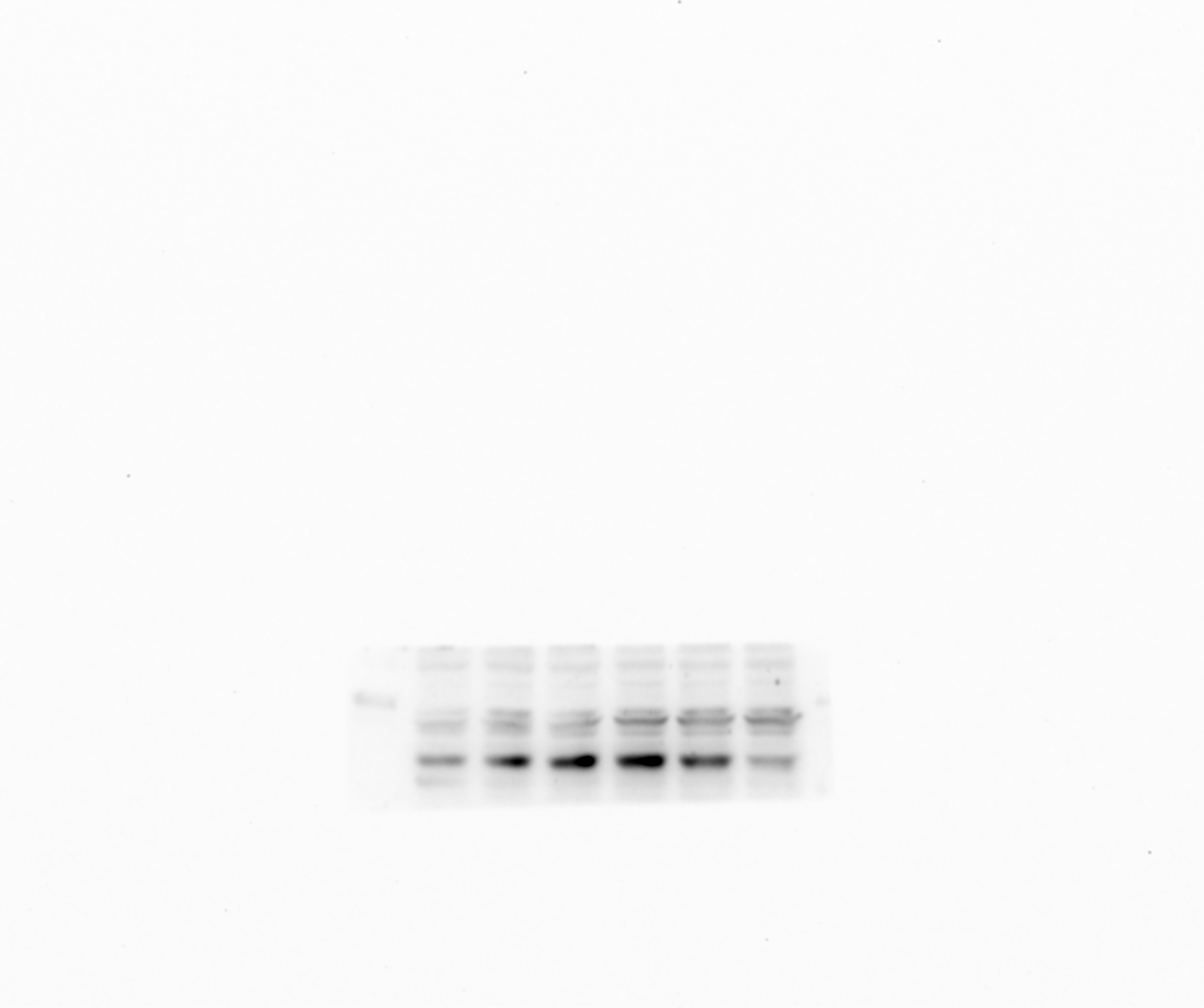

Supplement: Supplementary file 7 — Source data Fig. 4 [file 44319_2024_352_MOESM7_ESM.zip › Figure 4/4E/western DYRK4 Input.tif]

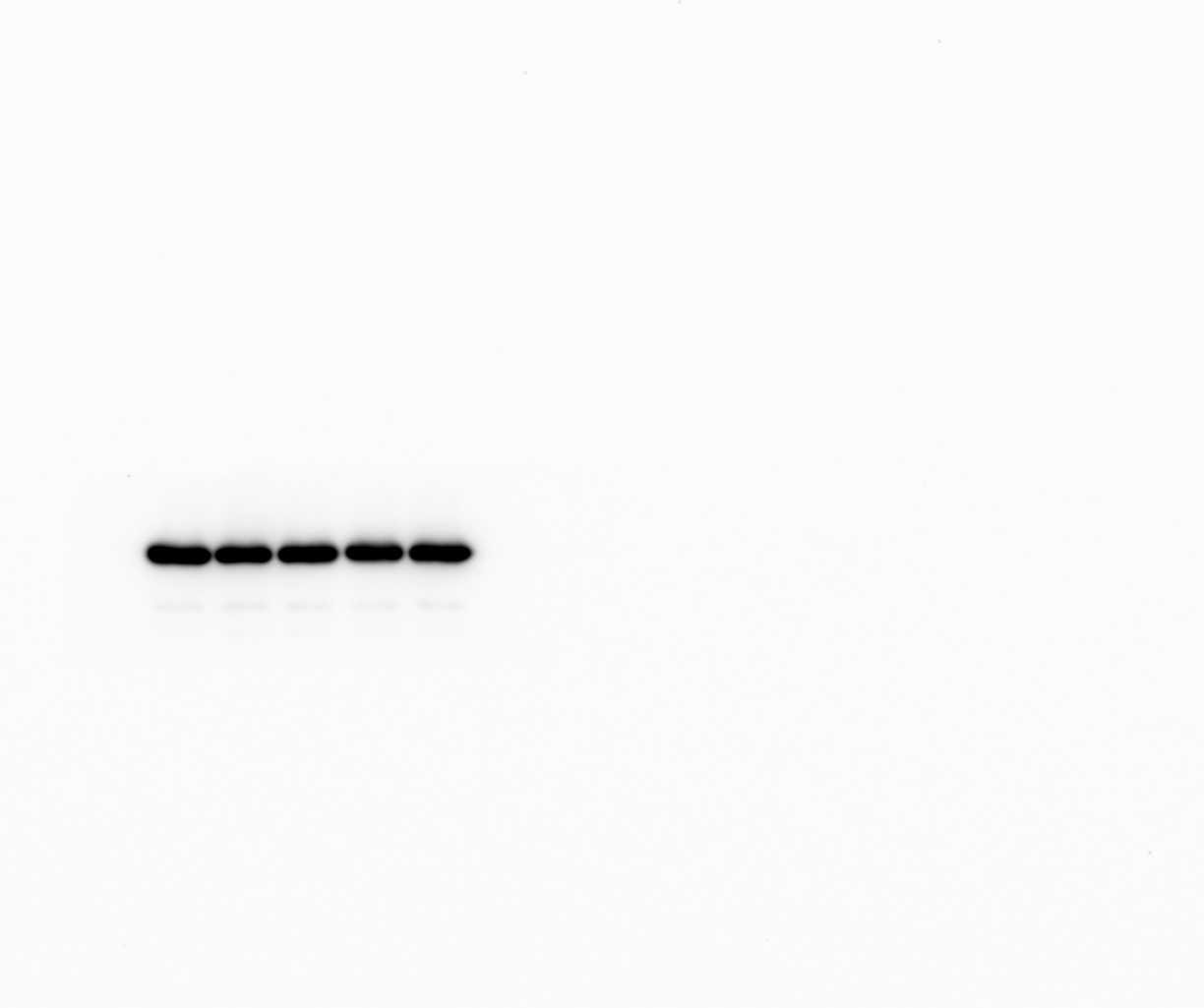

Supplement: Supplementary file 7 — Source data Fig. 4 [file 44319_2024_352_MOESM7_ESM.zip › Figure 4/4E/western GAPDH Input.tif]

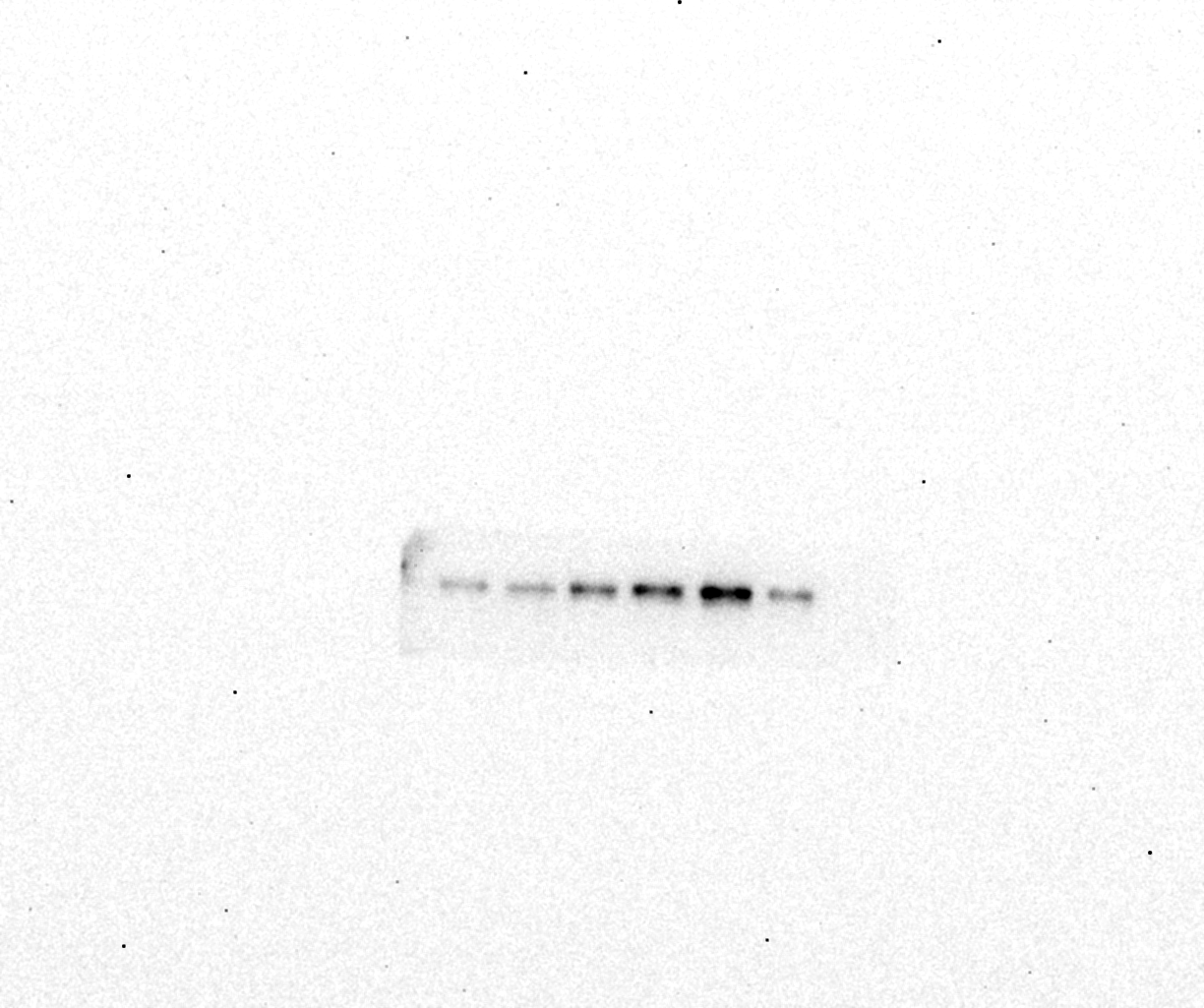

Supplement: Supplementary file 7 — Source data Fig. 4 [file 44319_2024_352_MOESM7_ESM.zip › Figure 4/4E/western IRF3 IP.tif]

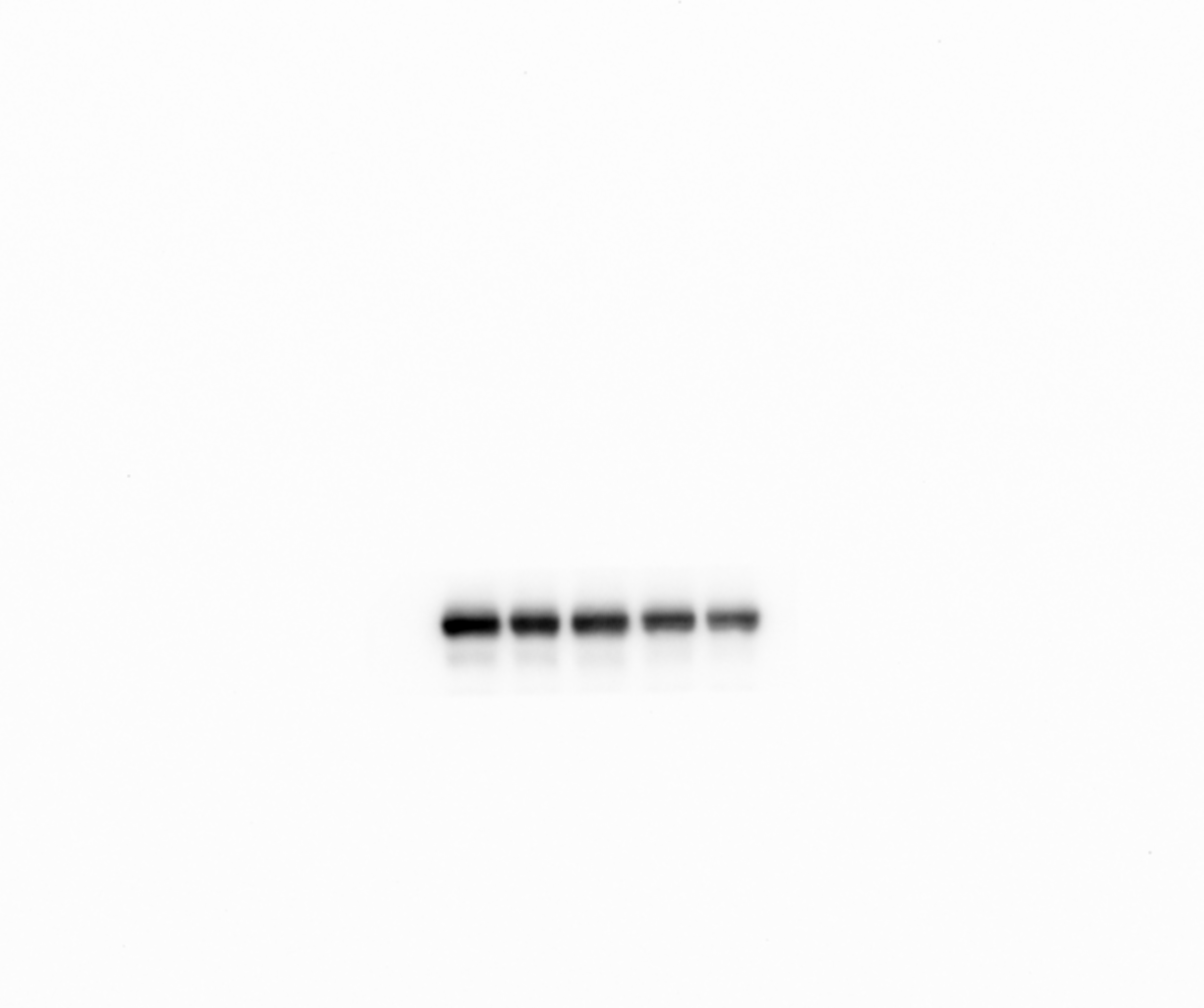

Supplement: Supplementary file 7 — Source data Fig. 4 [file 44319_2024_352_MOESM7_ESM.zip › Figure 4/4E/western IRF3 Input.tif]

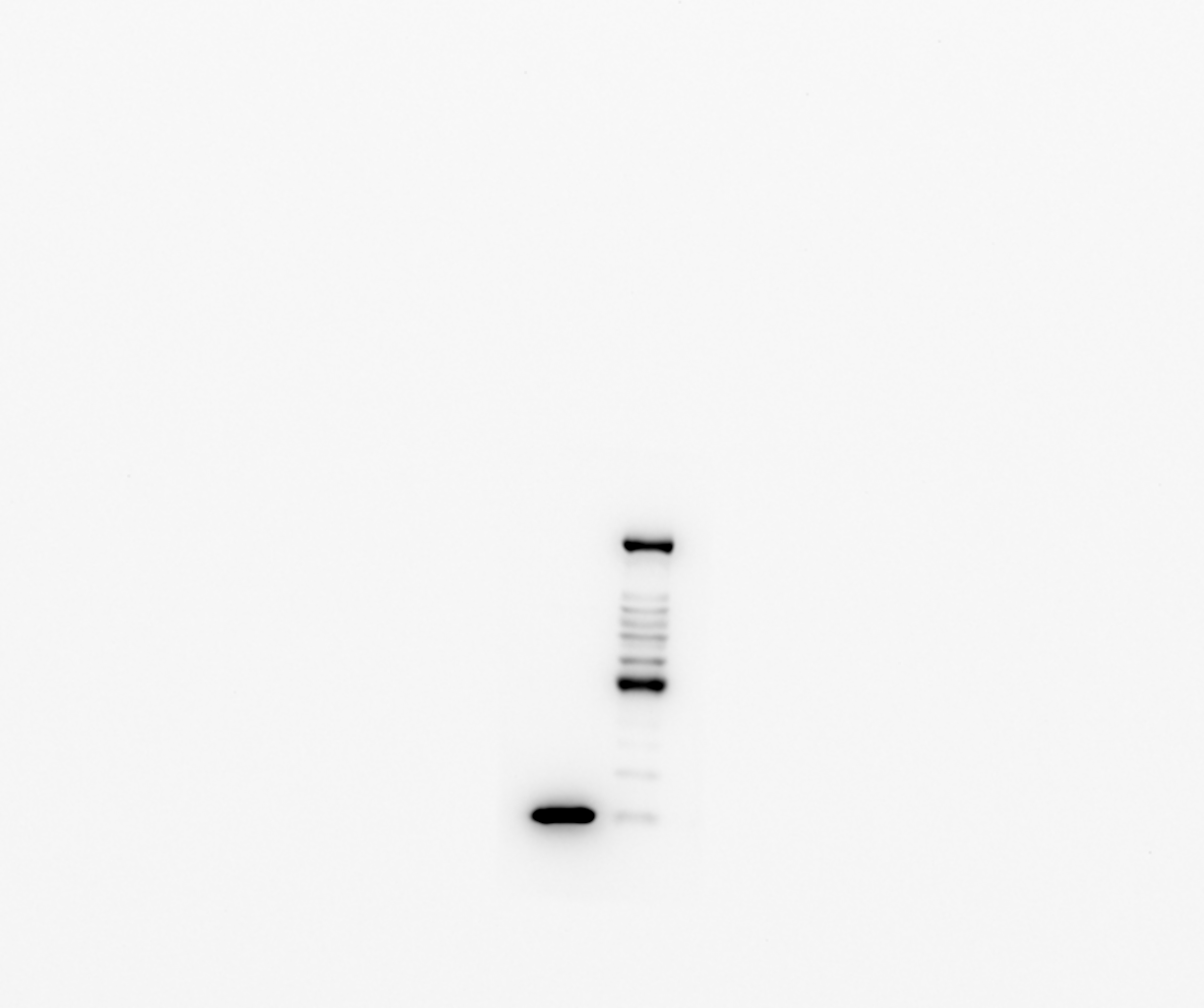

Supplement: Supplementary file 7 — Source data Fig. 4 [file 44319_2024_352_MOESM7_ESM.zip › Figure 4/4F/western Input GST-IRF3.tif]

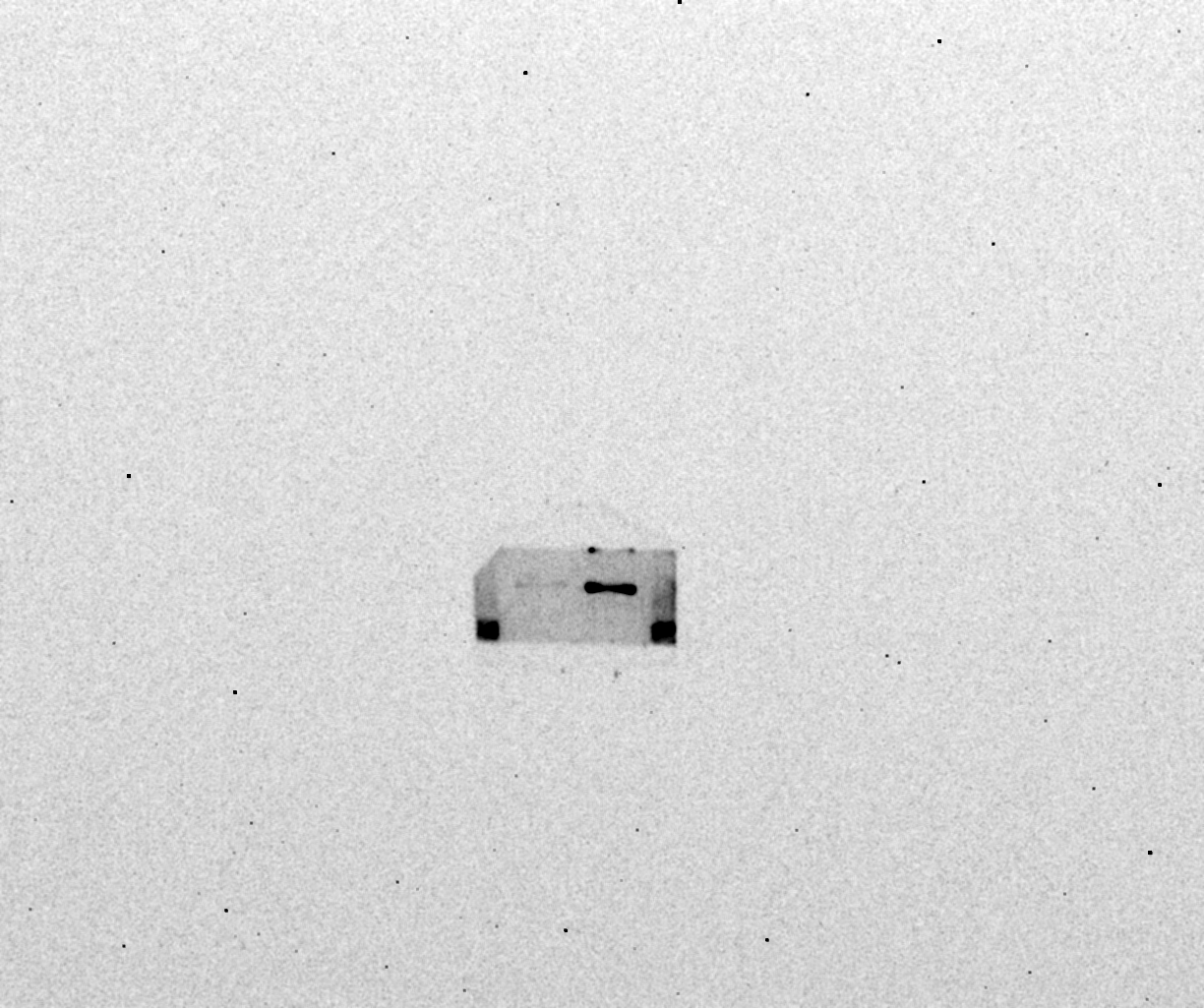

Supplement: Supplementary file 7 — Source data Fig. 4 [file 44319_2024_352_MOESM7_ESM.zip › Figure 4/4F/western DYRK4 IP.tif]

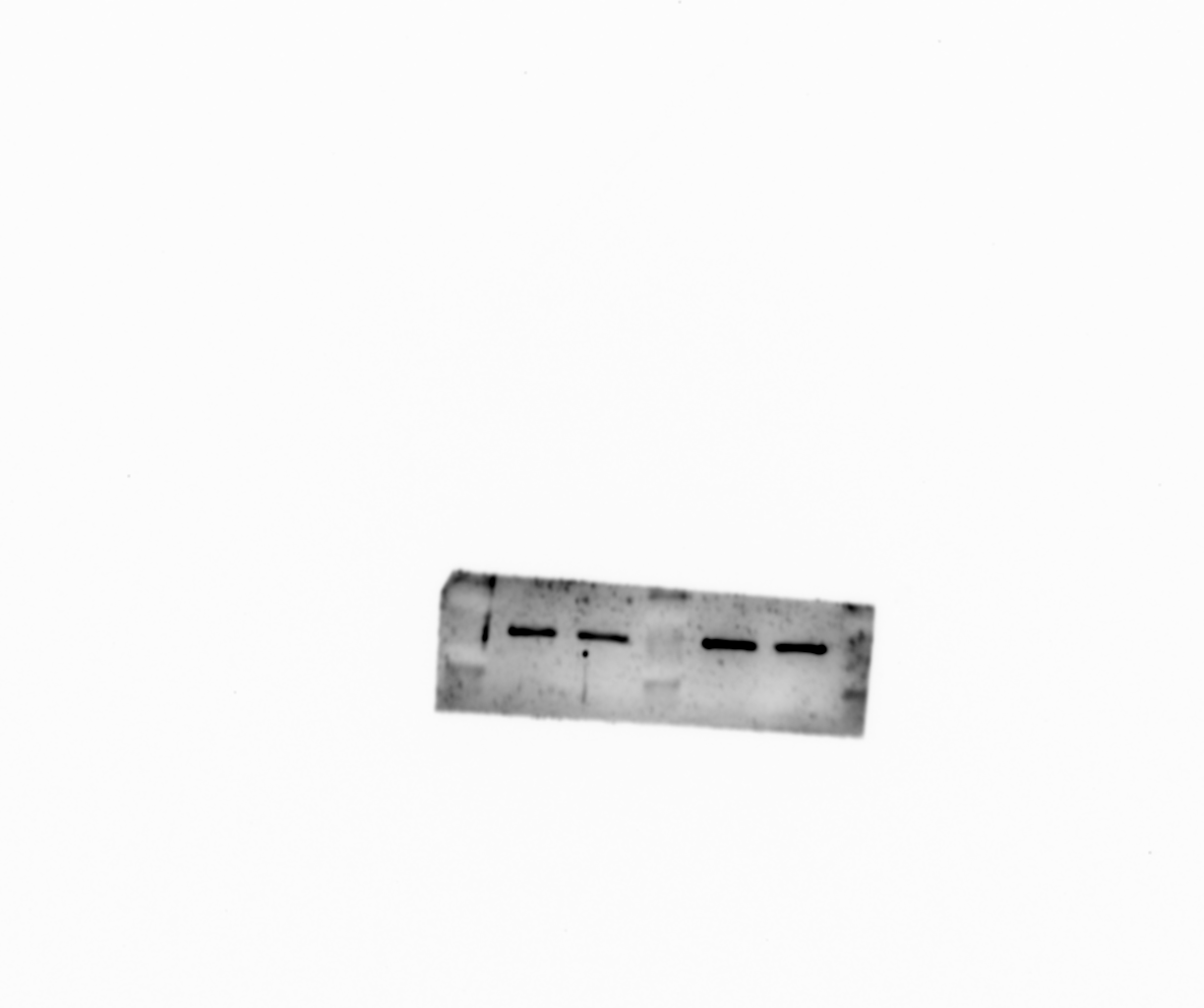

Supplement: Supplementary file 7 — Source data Fig. 4 [file 44319_2024_352_MOESM7_ESM.zip › Figure 4/4F/western DYRK4.tif]

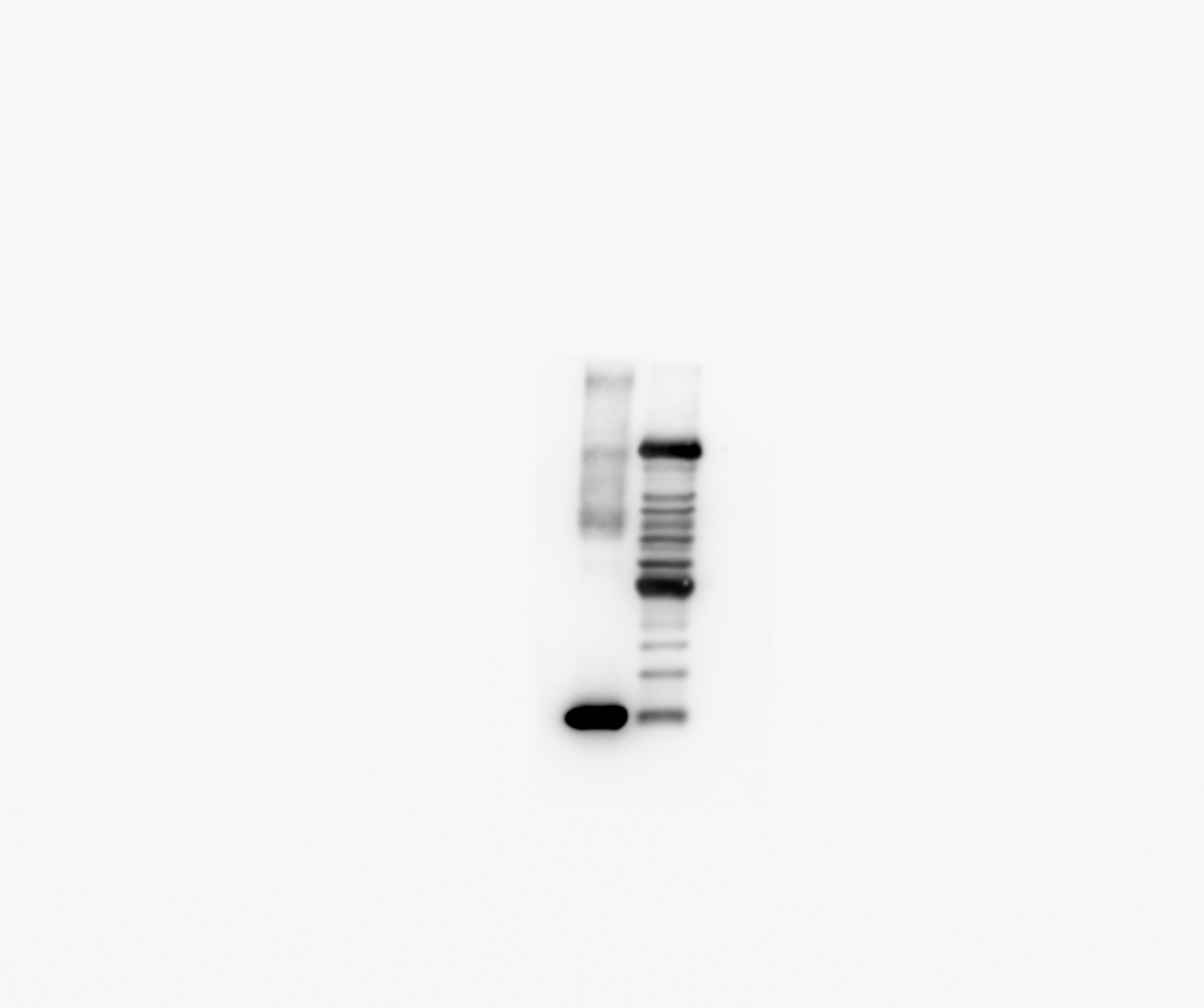

Supplement: Supplementary file 7 — Source data Fig. 4 [file 44319_2024_352_MOESM7_ESM.zip › Figure 4/4F/western GST IP GST-IRF3.tif]

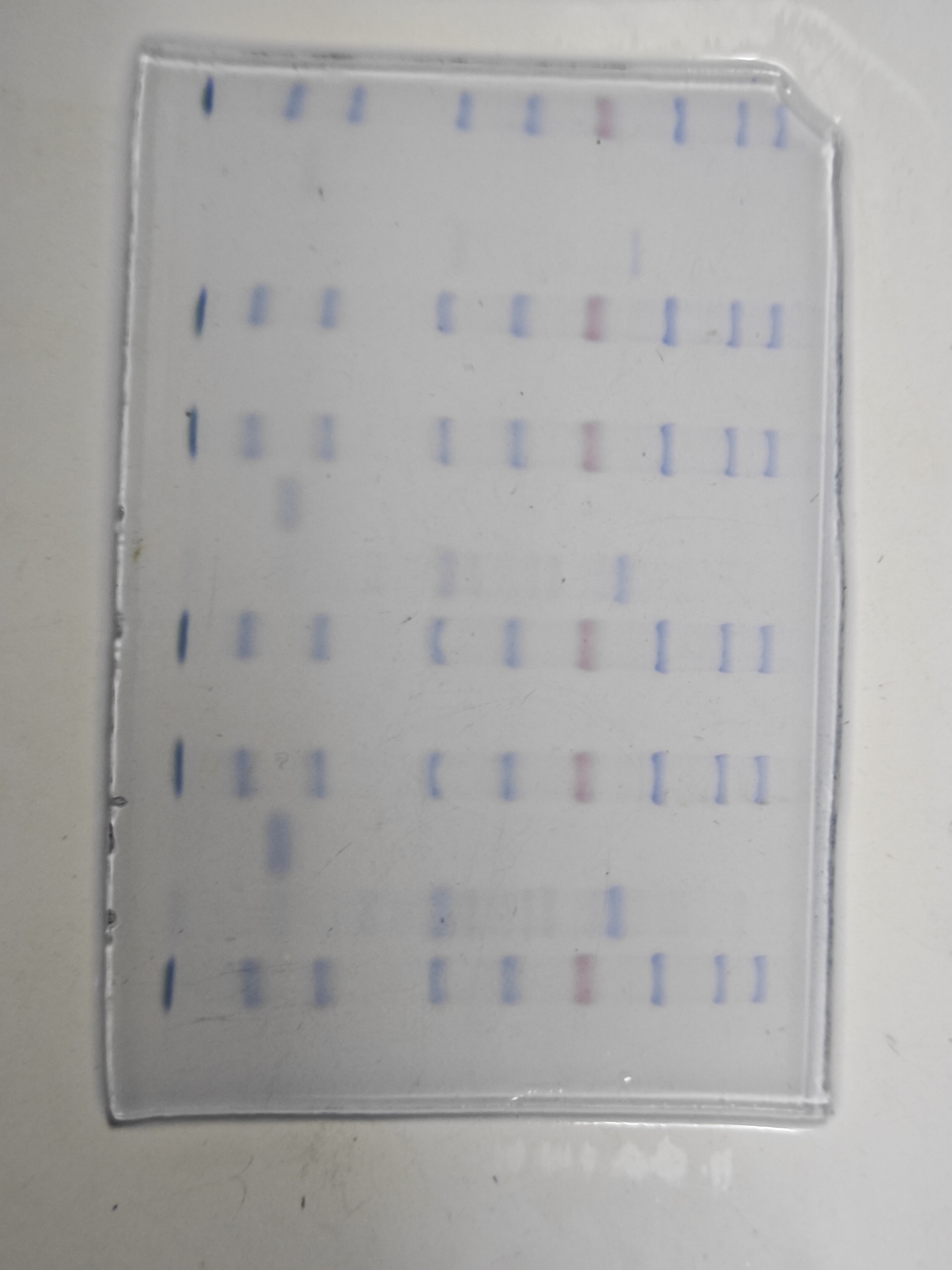

Supplement: Supplementary file 7 — Source data Fig. 4 [file 44319_2024_352_MOESM7_ESM.zip › Figure 4/4F/western IRF3-GST.jpg]

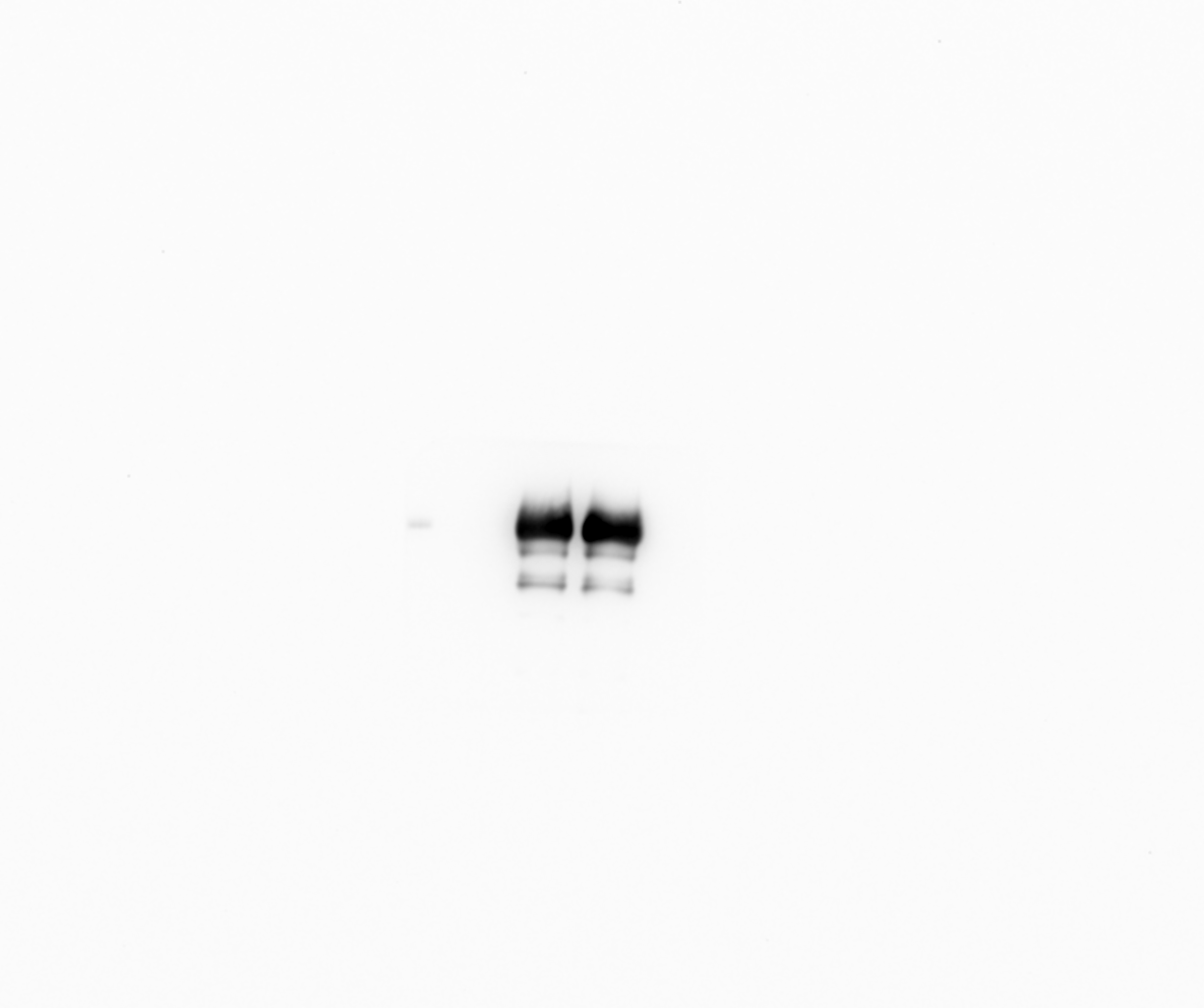

Supplement: Supplementary file 7 — Source data Fig. 4 [file 44319_2024_352_MOESM7_ESM.zip › Figure 4/4G/4G-1/western Flag IP.tif]

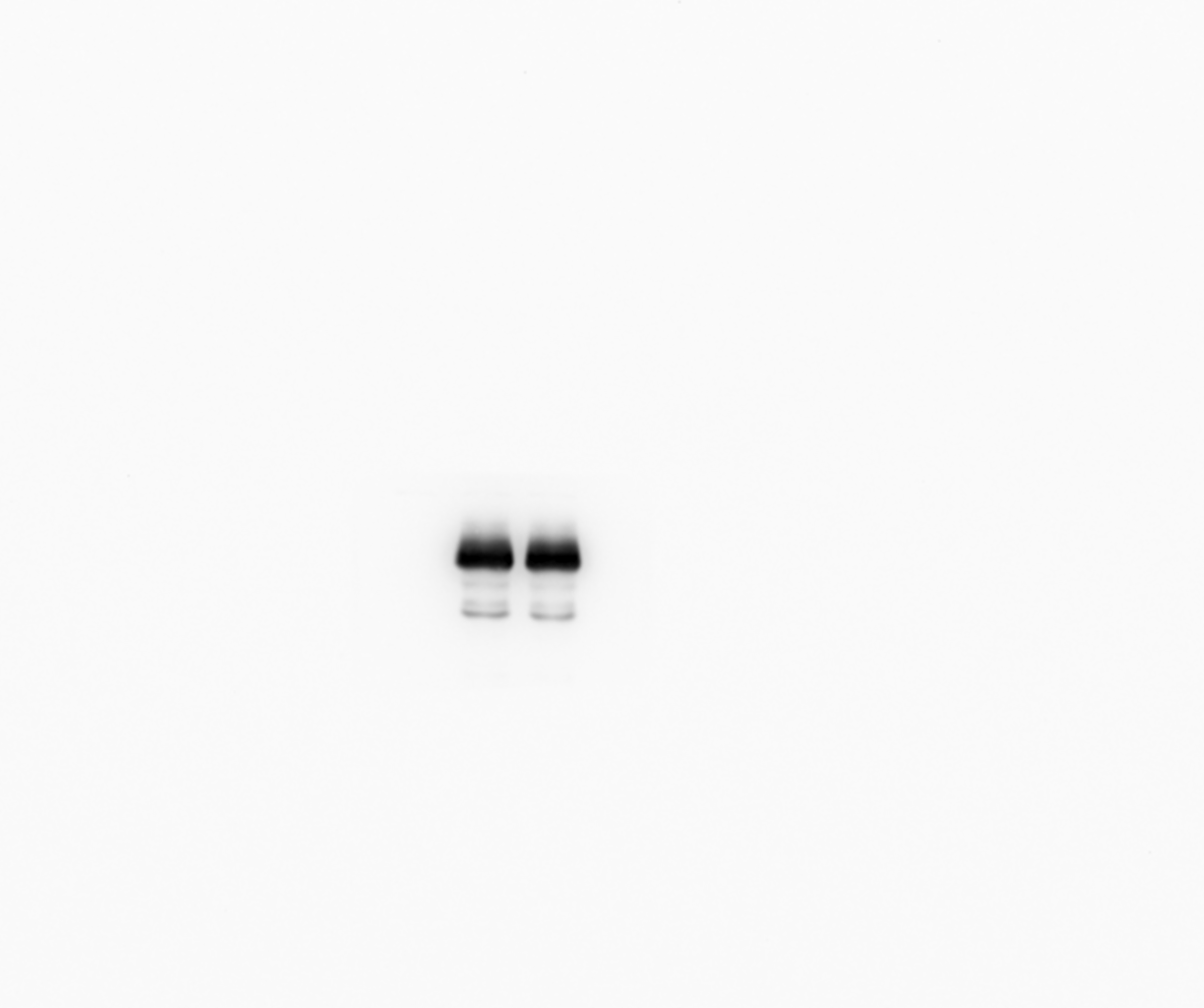

Supplement: Supplementary file 7 — Source data Fig. 4 [file 44319_2024_352_MOESM7_ESM.zip › Figure 4/4G/4G-1/western Flag Input.tif]

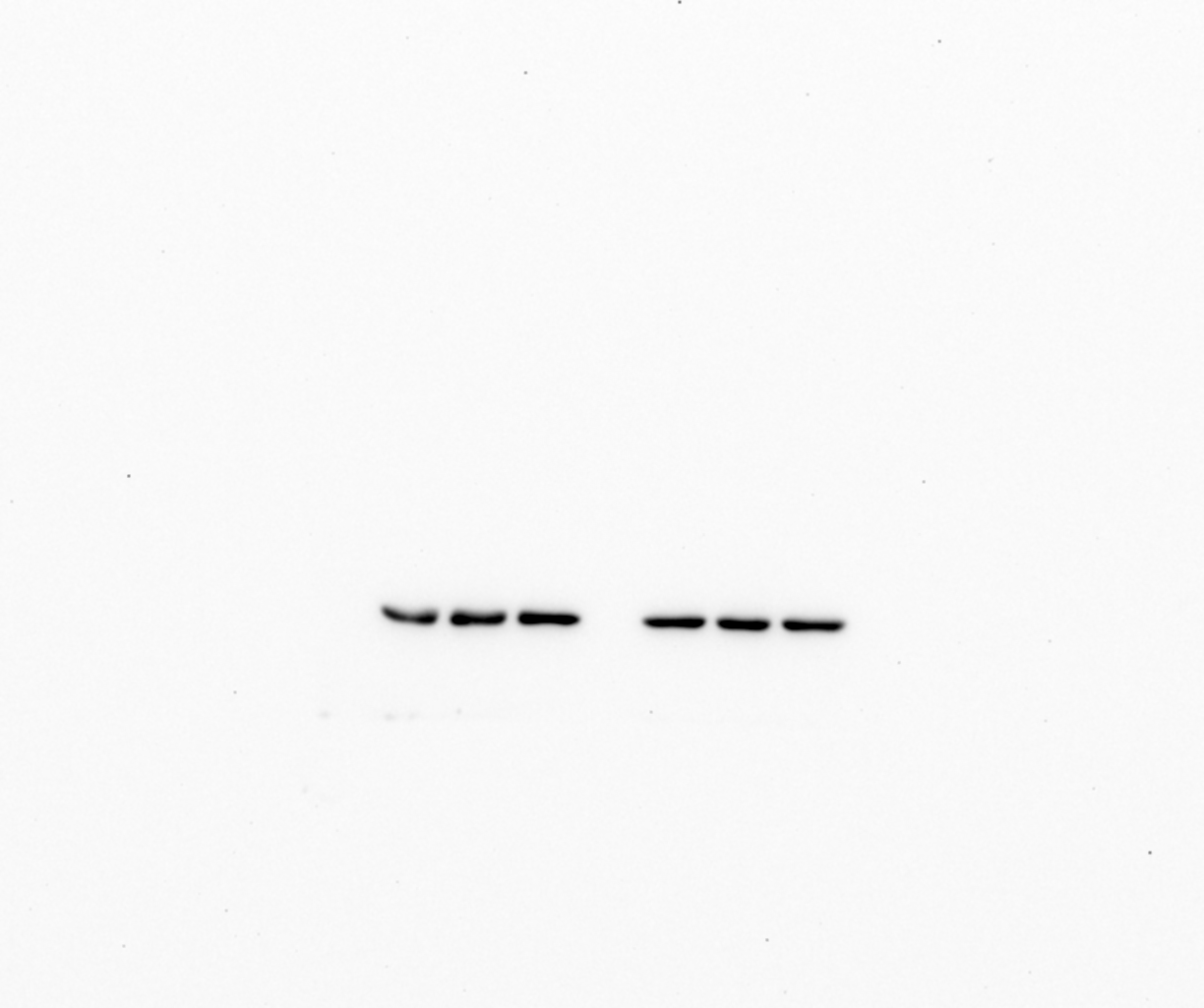

Supplement: Supplementary file 7 — Source data Fig. 4 [file 44319_2024_352_MOESM7_ESM.zip › Figure 4/4G/4G-1/western GAPDH.tif]

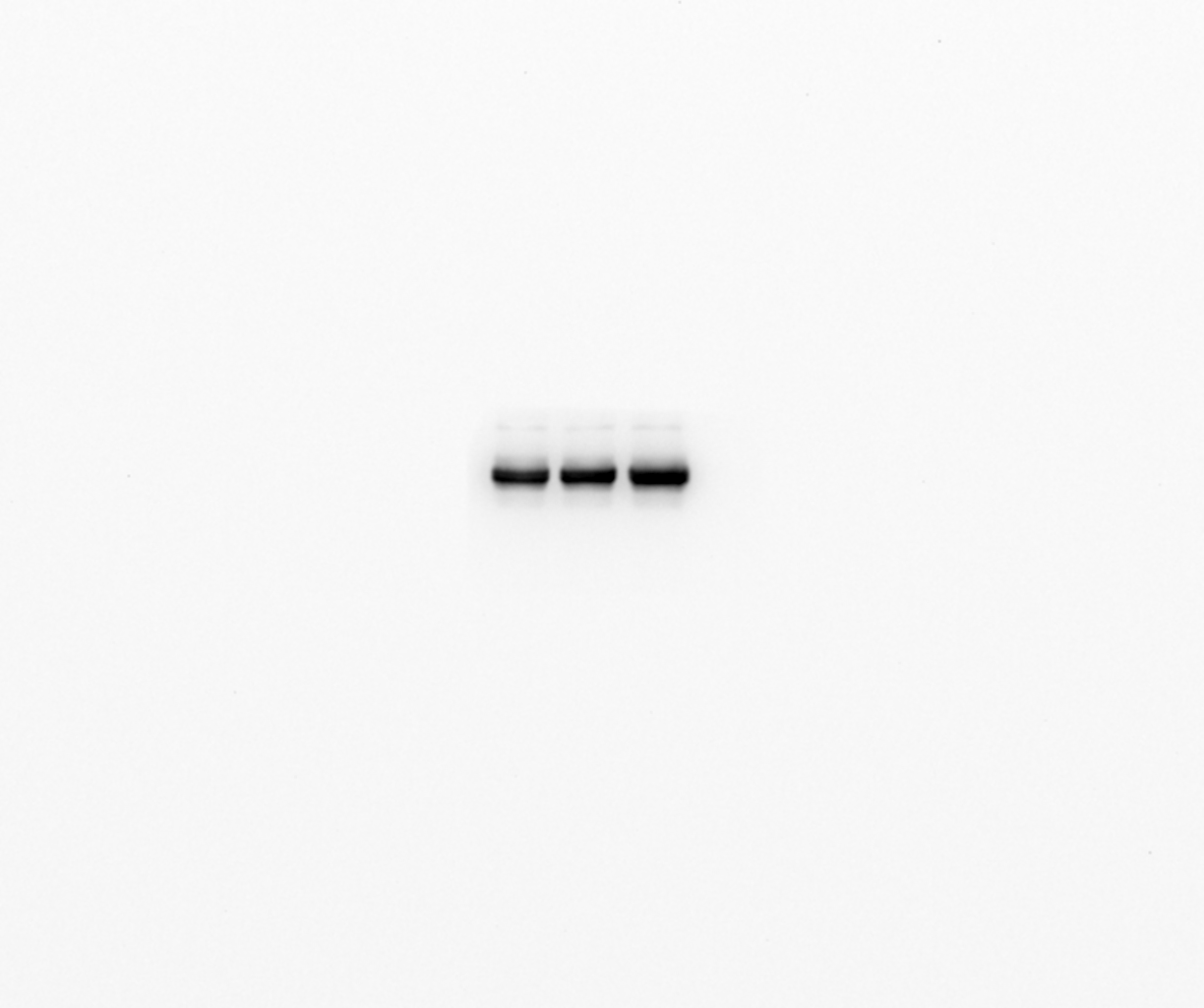

Supplement: Supplementary file 7 — Source data Fig. 4 [file 44319_2024_352_MOESM7_ESM.zip › Figure 4/4G/4G-1/western HA Input.tif]

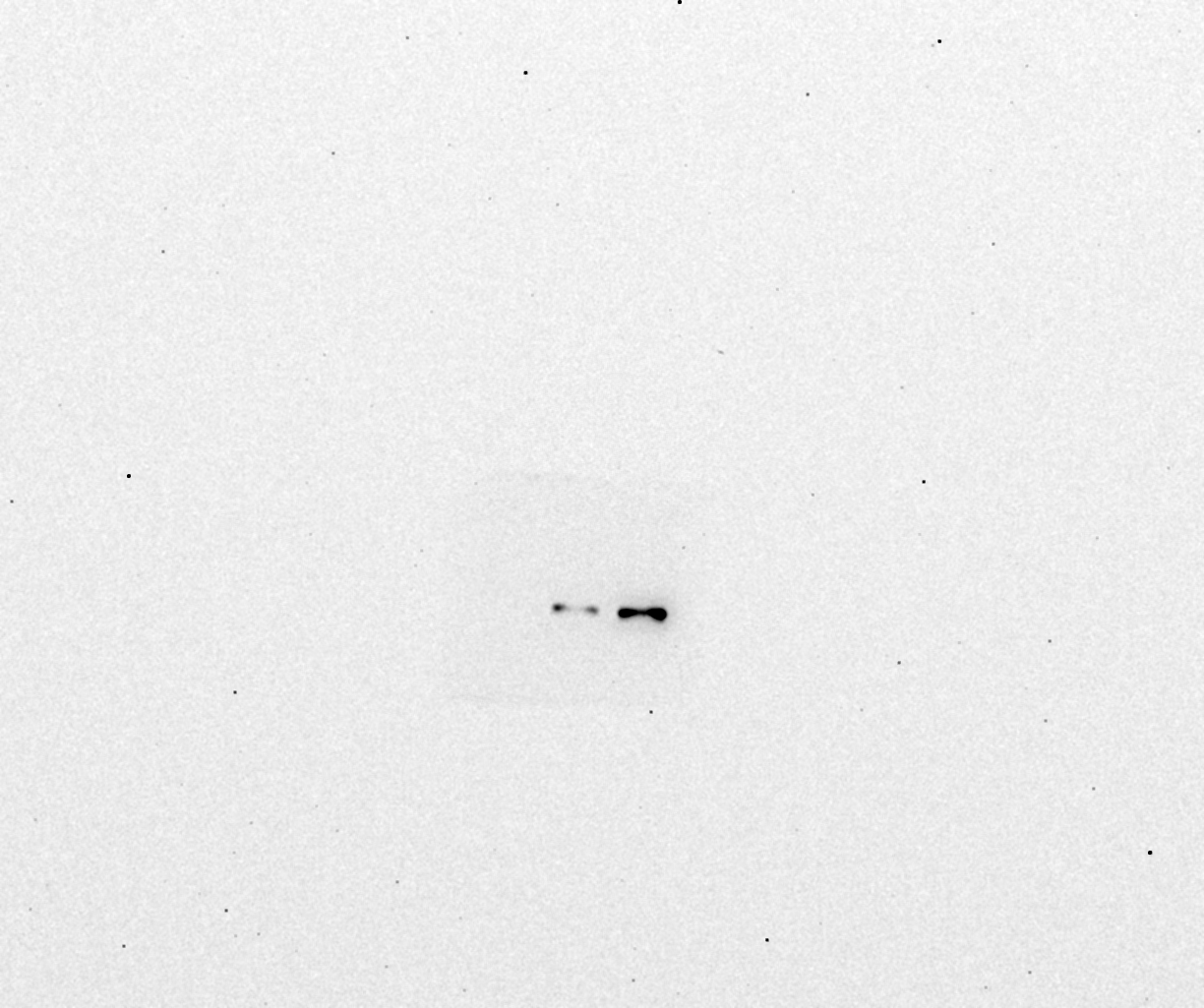

Supplement: Supplementary file 7 — Source data Fig. 4 [file 44319_2024_352_MOESM7_ESM.zip › Figure 4/4G/4G-1/western HA IP.tif]

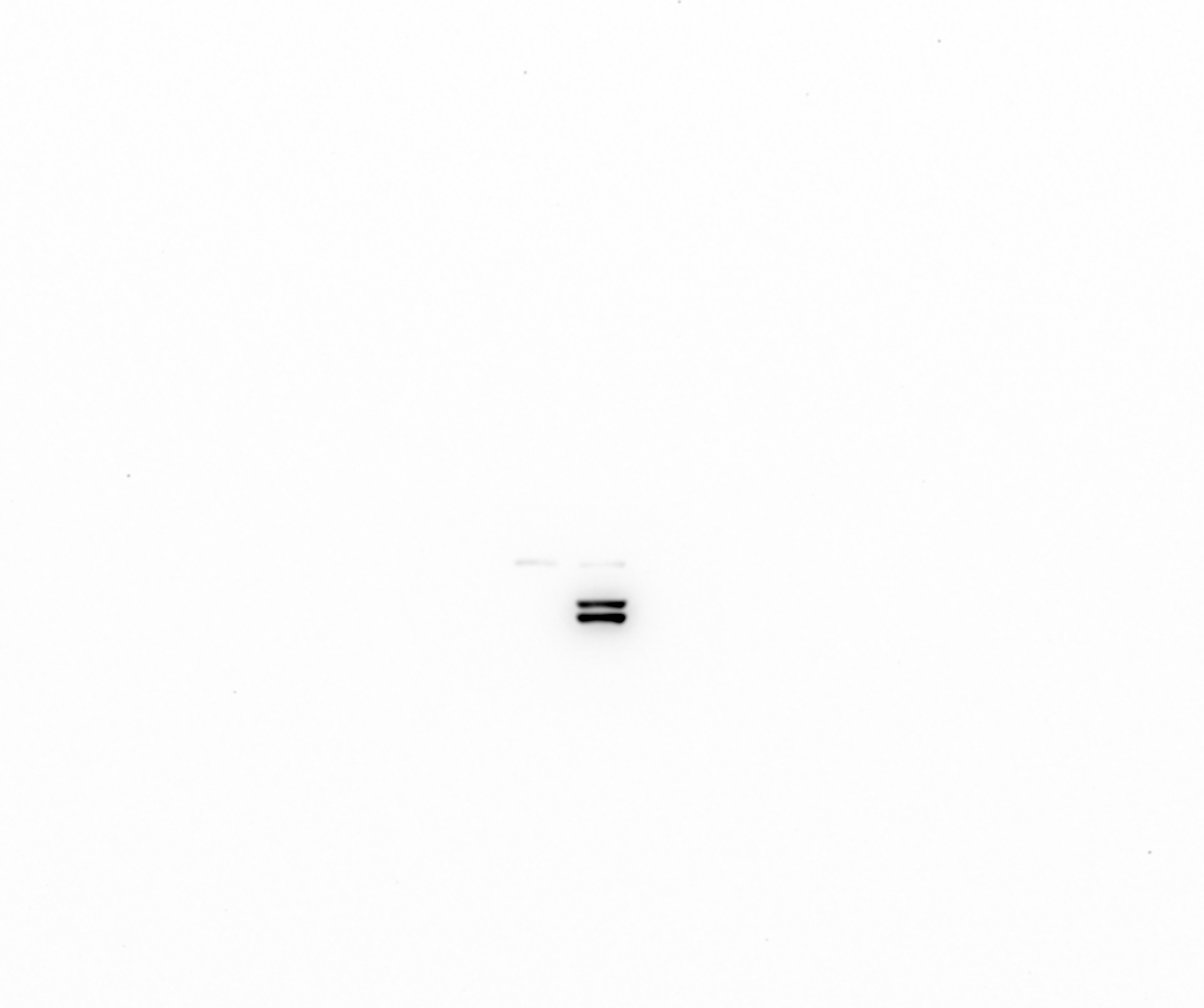

Supplement: Supplementary file 7 — Source data Fig. 4 [file 44319_2024_352_MOESM7_ESM.zip › Figure 4/4G/4G-1/western myc Input.tif]

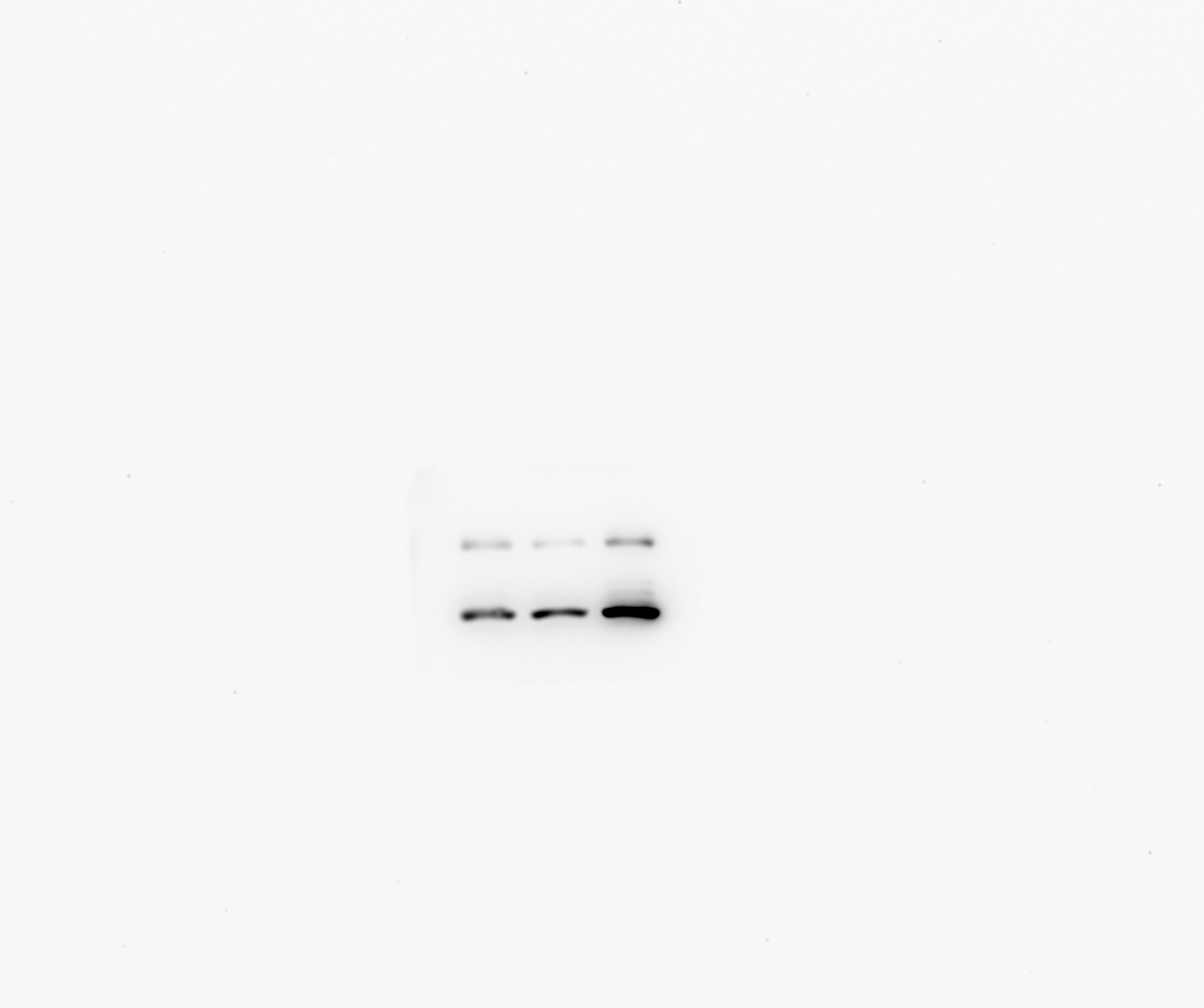

Supplement: Supplementary file 7 — Source data Fig. 4 [file 44319_2024_352_MOESM7_ESM.zip › Figure 4/4G/4G-2/western Flag Input.tif]

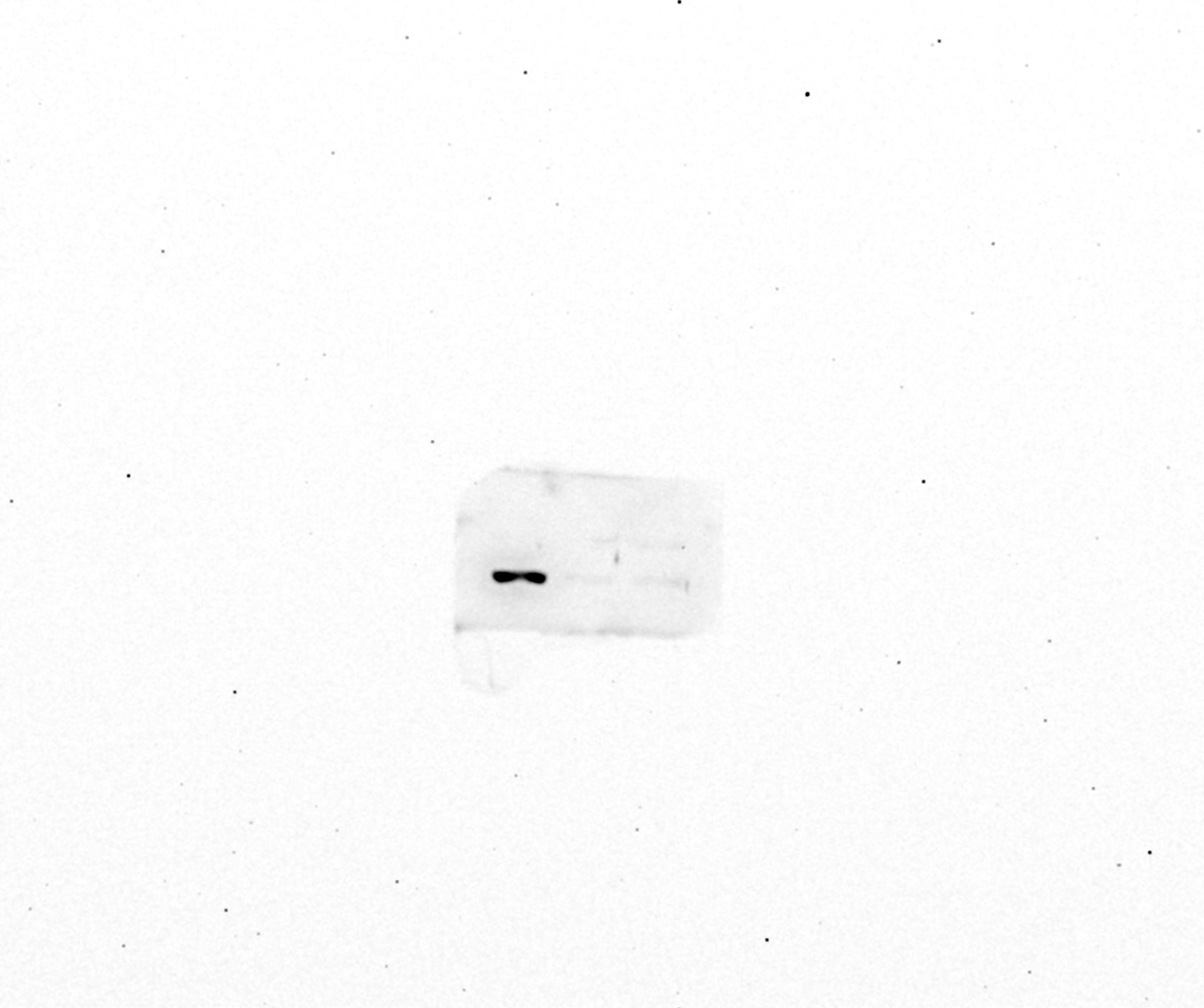

Supplement: Supplementary file 7 — Source data Fig. 4 [file 44319_2024_352_MOESM7_ESM.zip › Figure 4/4G/4G-2/western DYRK4.tif]
