# Supplementary figures and images for: DYRK4 upregulates antiviral innate immunity by promoting IRF3 activation (part 2 of 3)
Source: EMBO Rep. 2024 Dec 19;26(3):690–719. doi: 10.1038/s44319-024-00352-x (PMC11811199; doi:10.1038/s44319-024-00352-x)

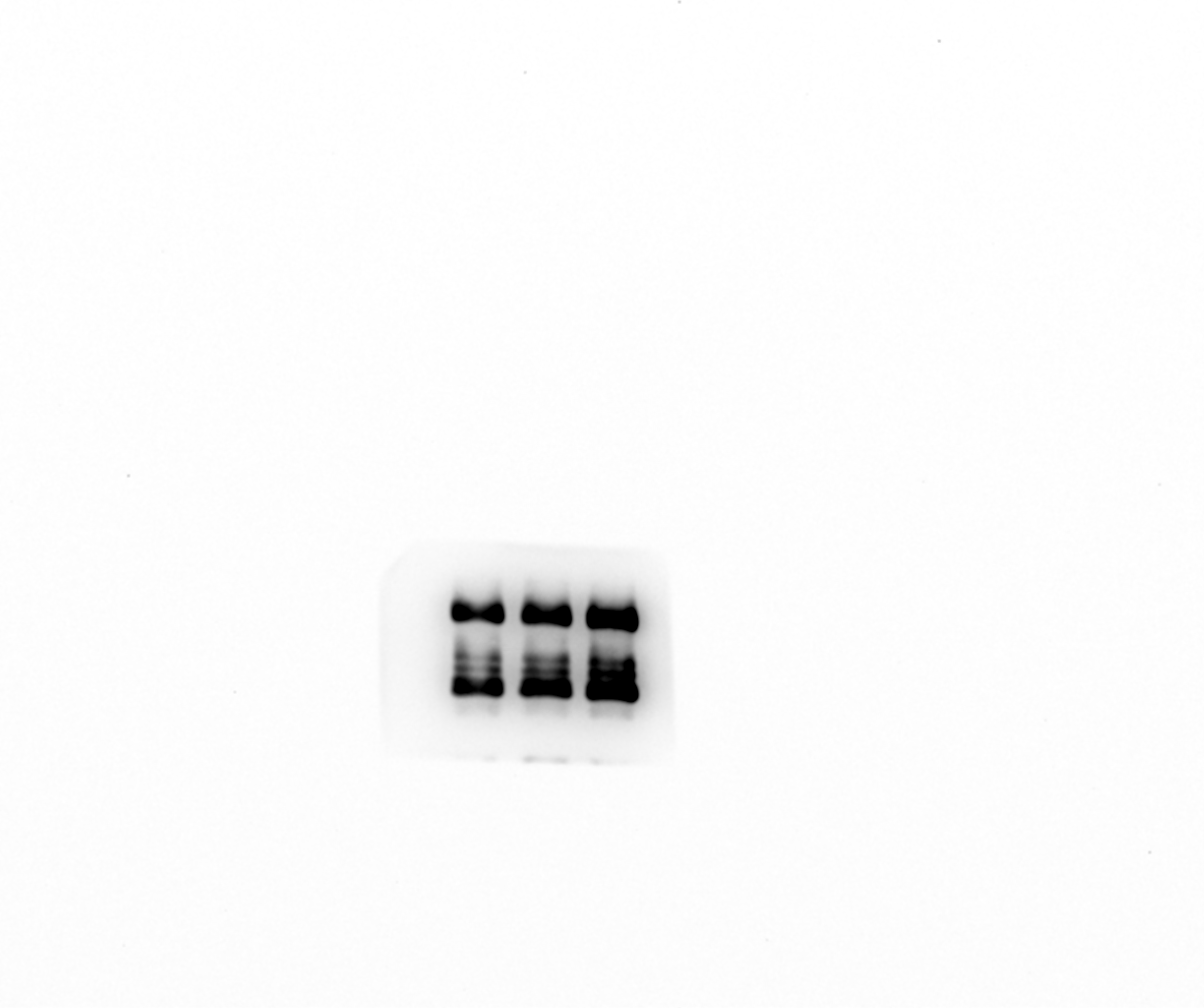

Supplement: Supplementary file 7 — Source data Fig. 4 [file 44319_2024_352_MOESM7_ESM.zip › Figure 4/4G/4G-2/western Flag IP.tif]

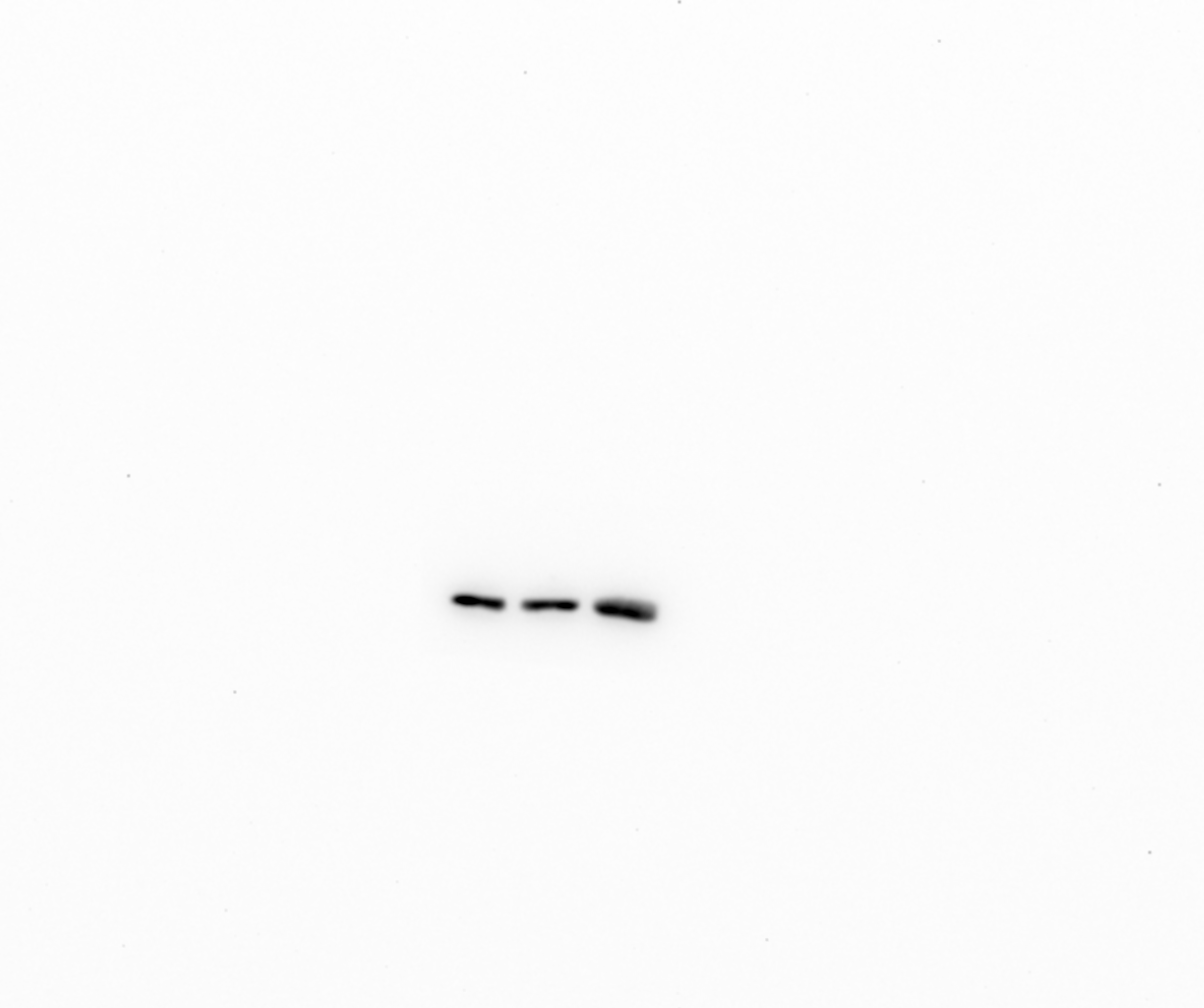

Supplement: Supplementary file 7 — Source data Fig. 4 [file 44319_2024_352_MOESM7_ESM.zip › Figure 4/4G/4G-2/western GAPDH Input.tif]

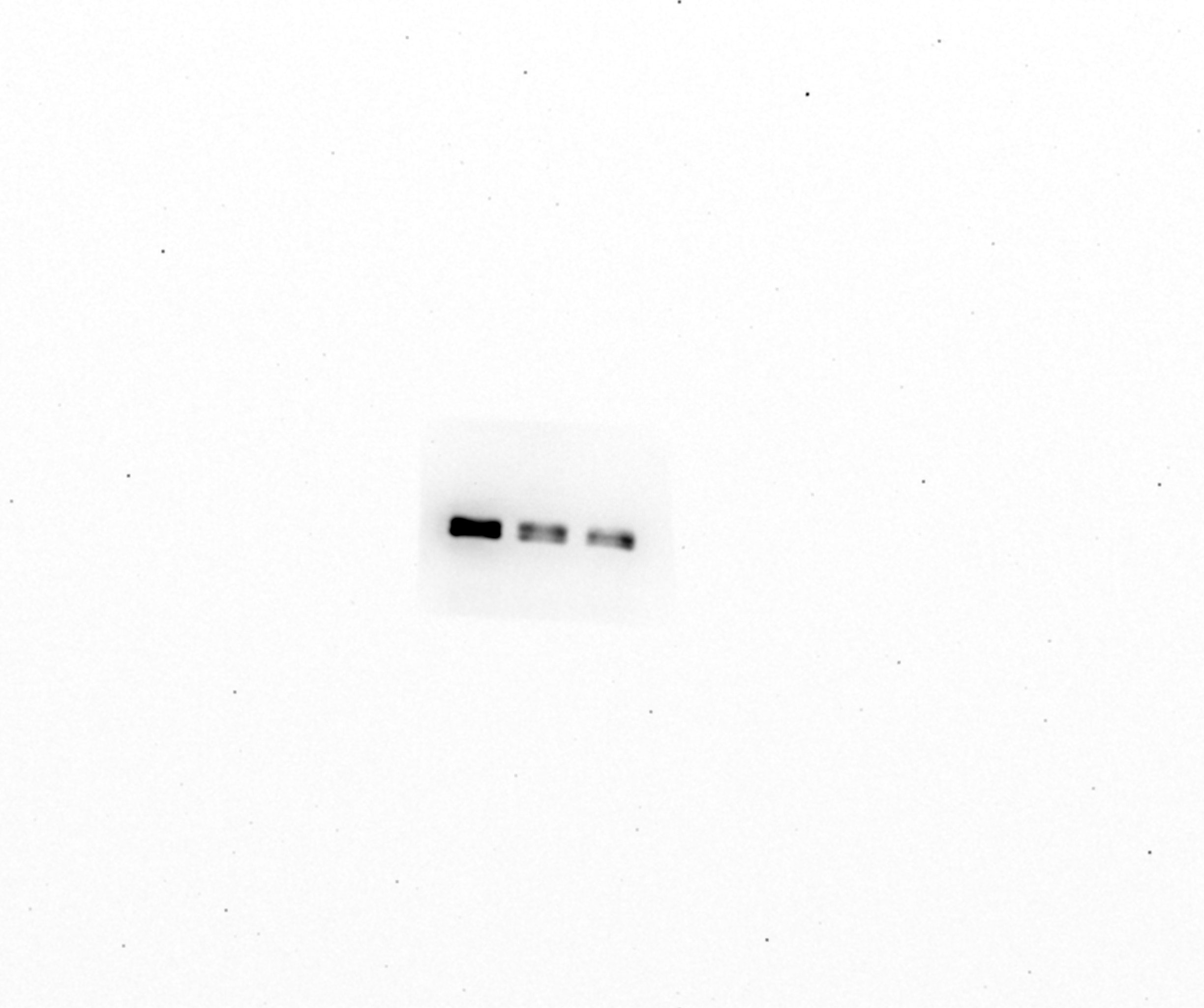

Supplement: Supplementary file 7 — Source data Fig. 4 [file 44319_2024_352_MOESM7_ESM.zip › Figure 4/4G/4G-2/western HA IP.tif]

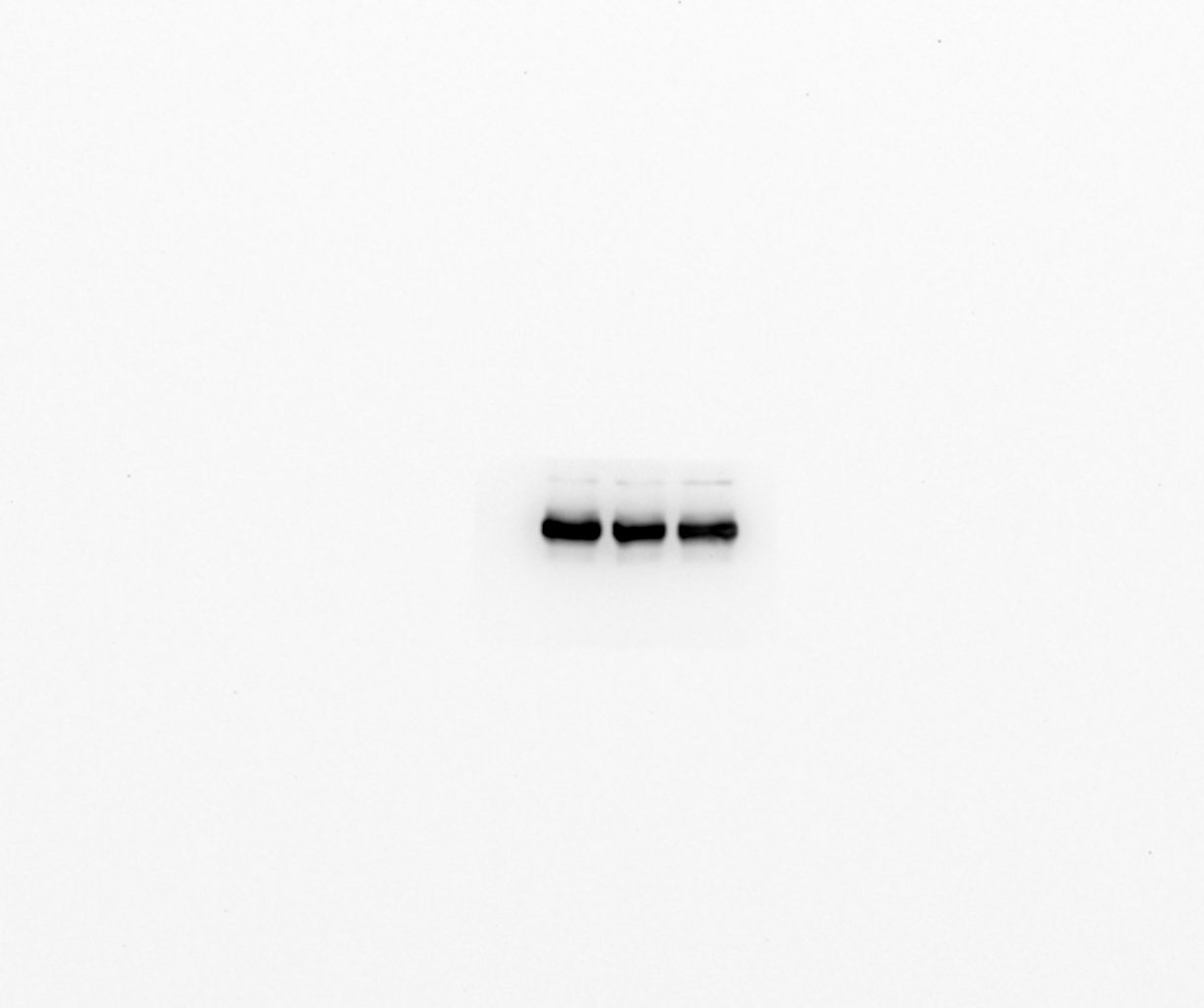

Supplement: Supplementary file 7 — Source data Fig. 4 [file 44319_2024_352_MOESM7_ESM.zip › Figure 4/4G/4G-2/western HA Input.tif]

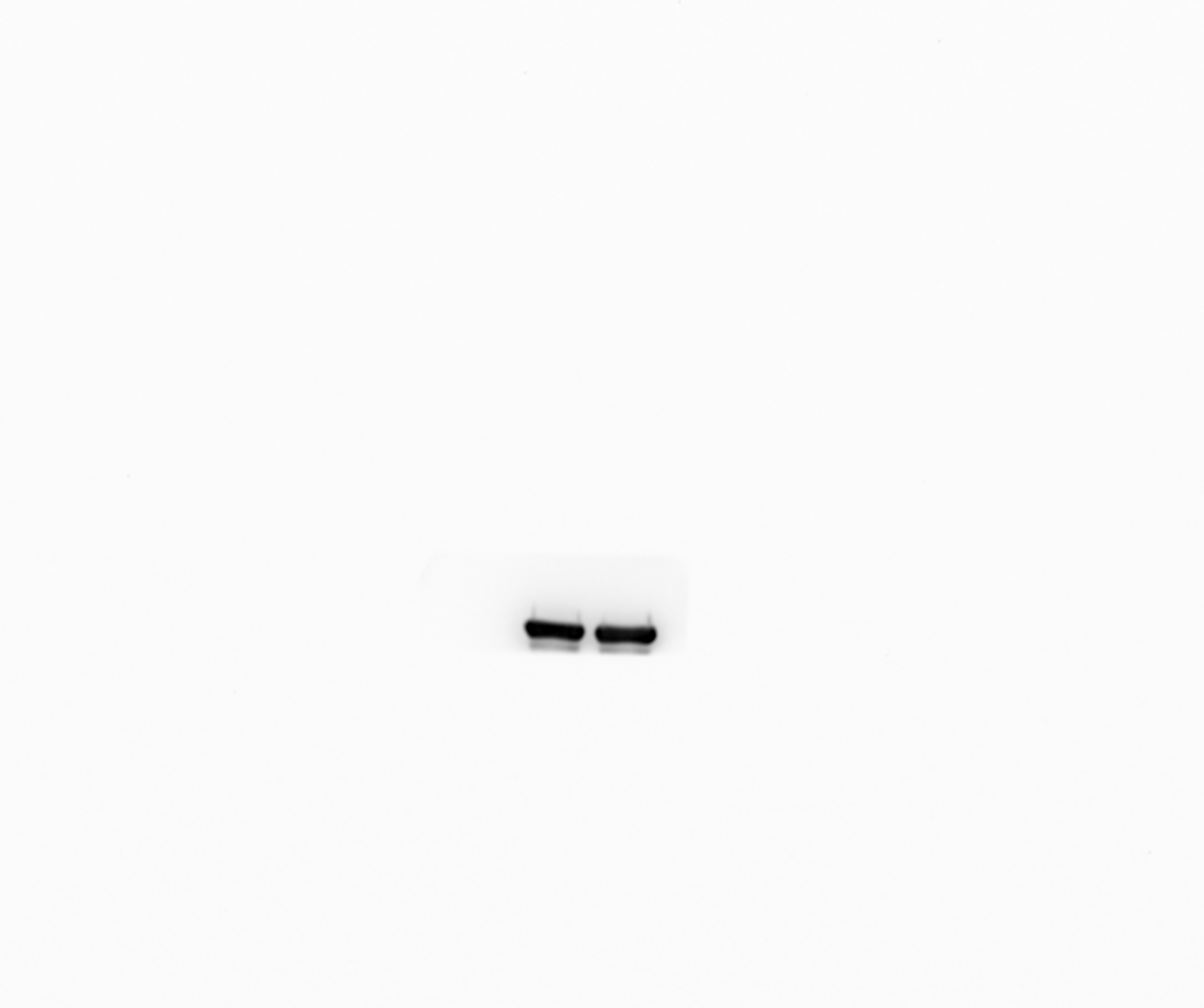

Supplement: Supplementary file 7 — Source data Fig. 4 [file 44319_2024_352_MOESM7_ESM.zip › Figure 4/4H/4H-1/western Flag IP.tif]

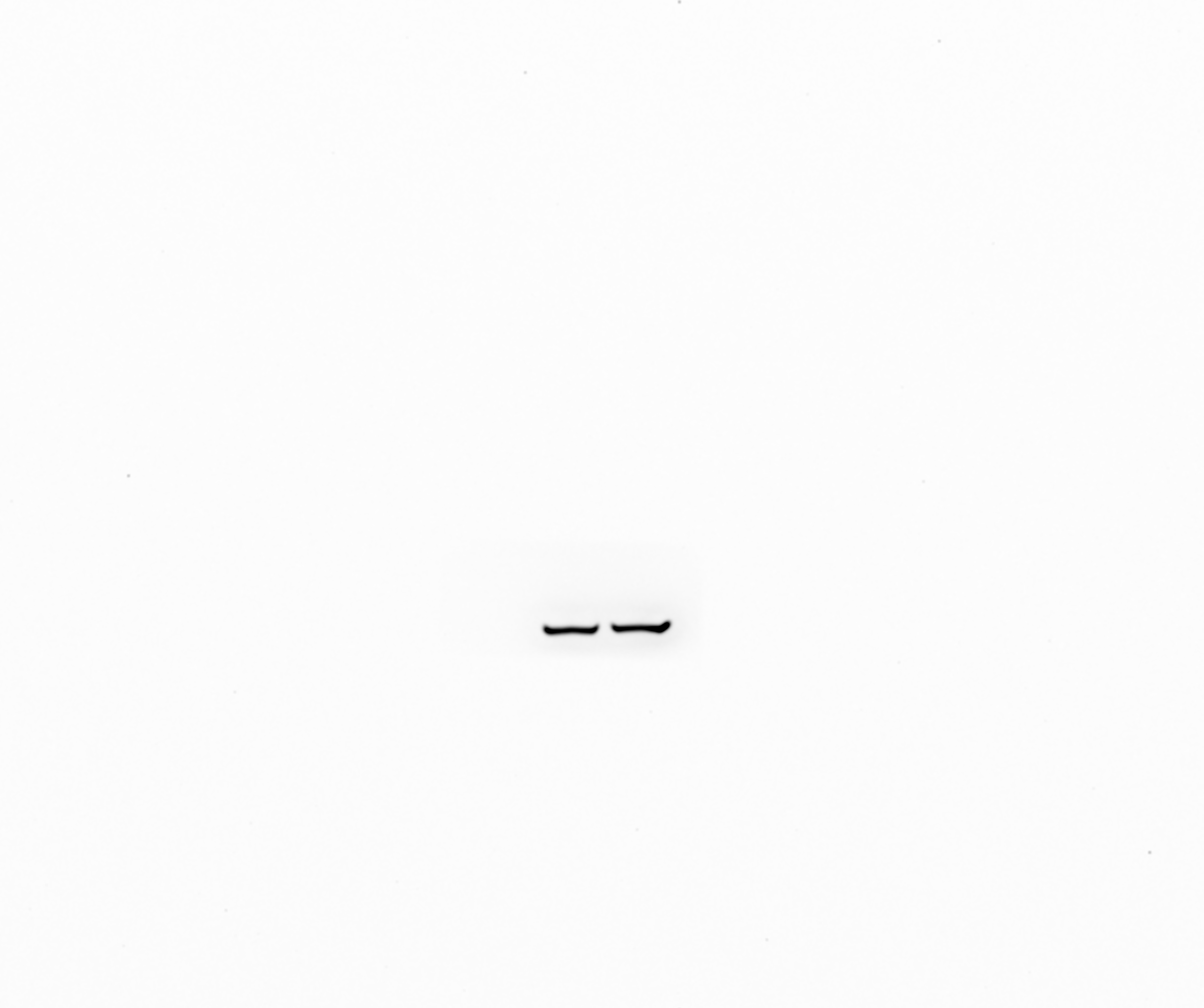

Supplement: Supplementary file 7 — Source data Fig. 4 [file 44319_2024_352_MOESM7_ESM.zip › Figure 4/4H/4H-1/western Flag Input.tif]

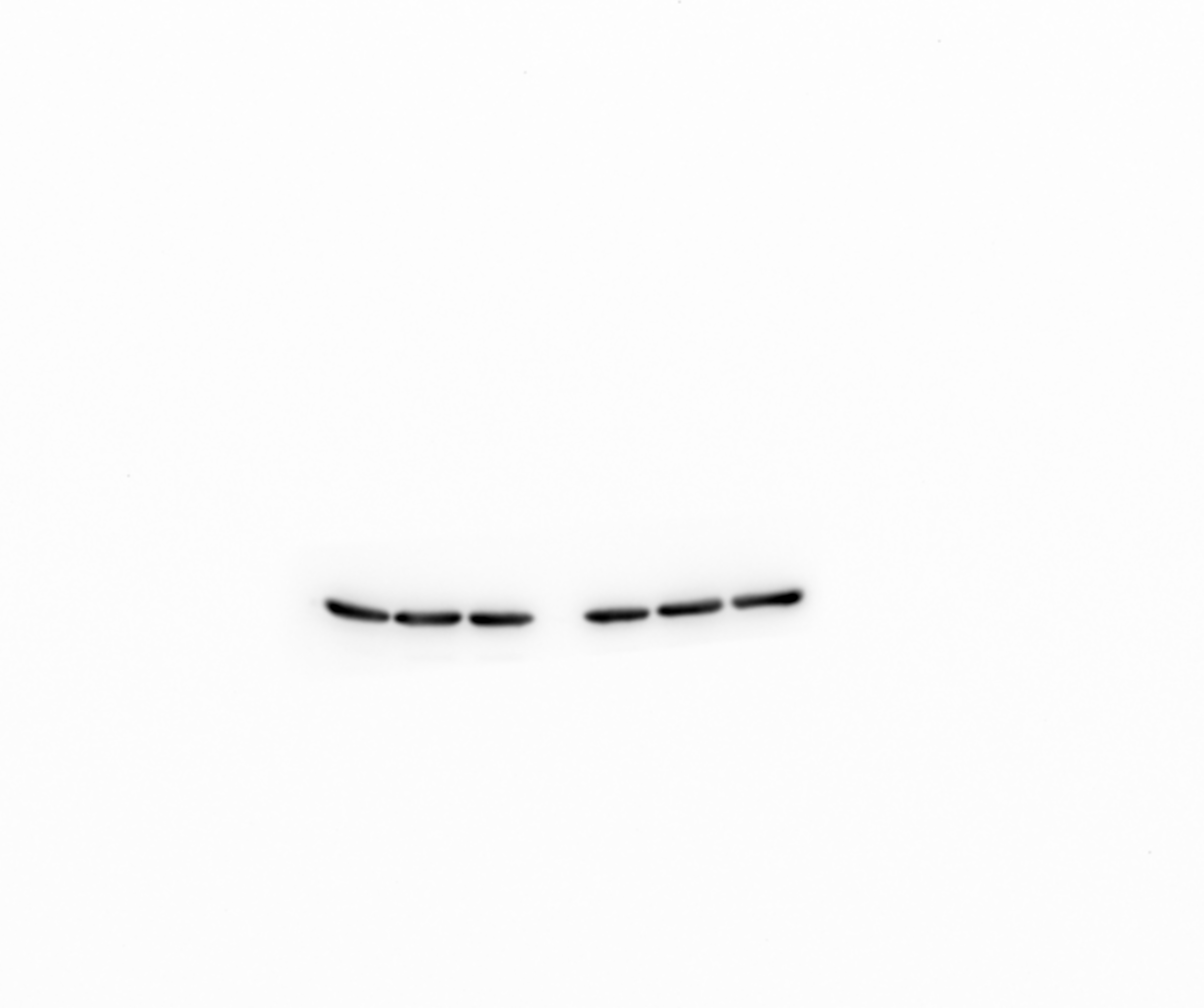

Supplement: Supplementary file 7 — Source data Fig. 4 [file 44319_2024_352_MOESM7_ESM.zip › Figure 4/4H/4H-1/western GAPDH.tif]

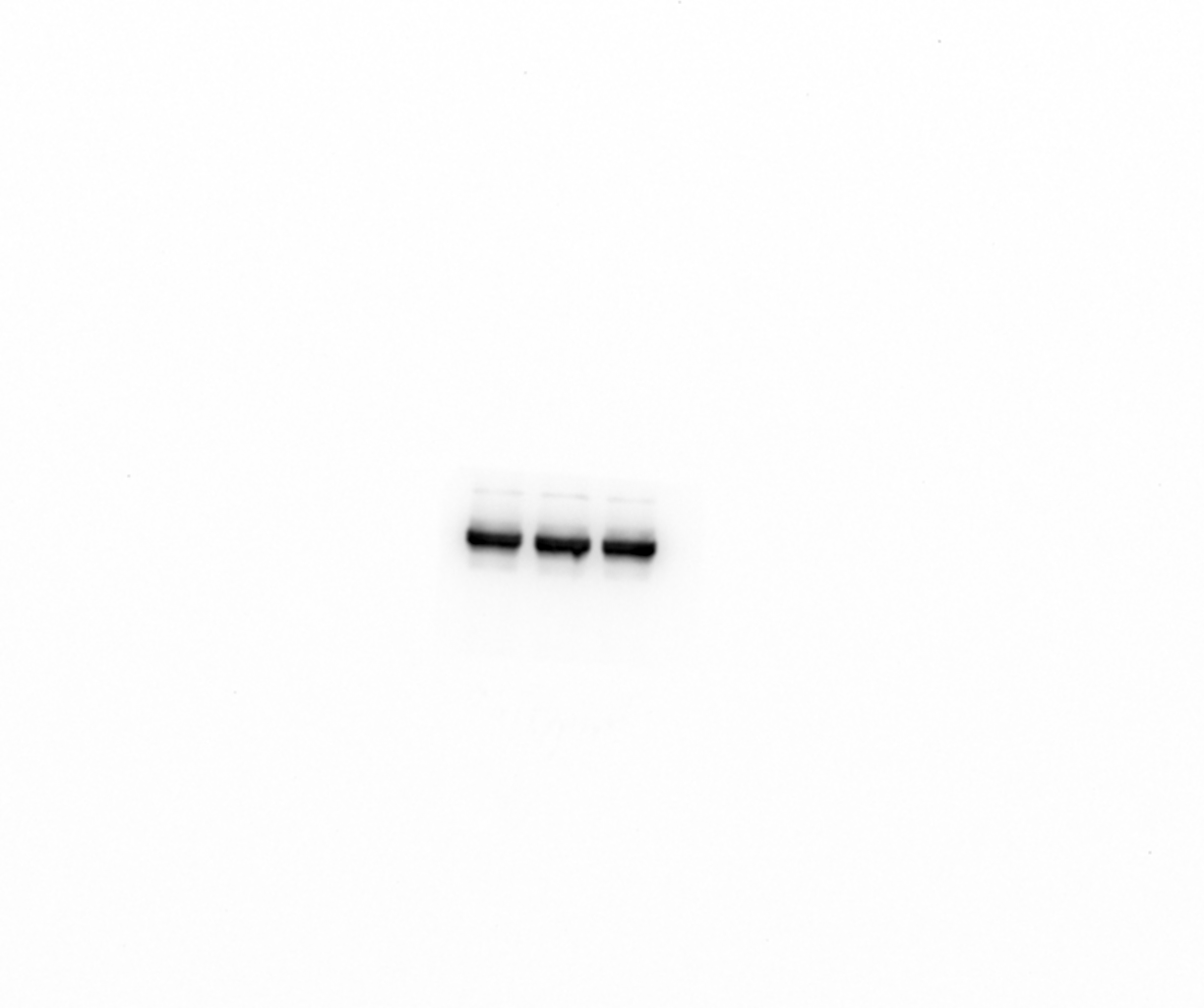

Supplement: Supplementary file 7 — Source data Fig. 4 [file 44319_2024_352_MOESM7_ESM.zip › Figure 4/4H/4H-1/western HA Input.tif]

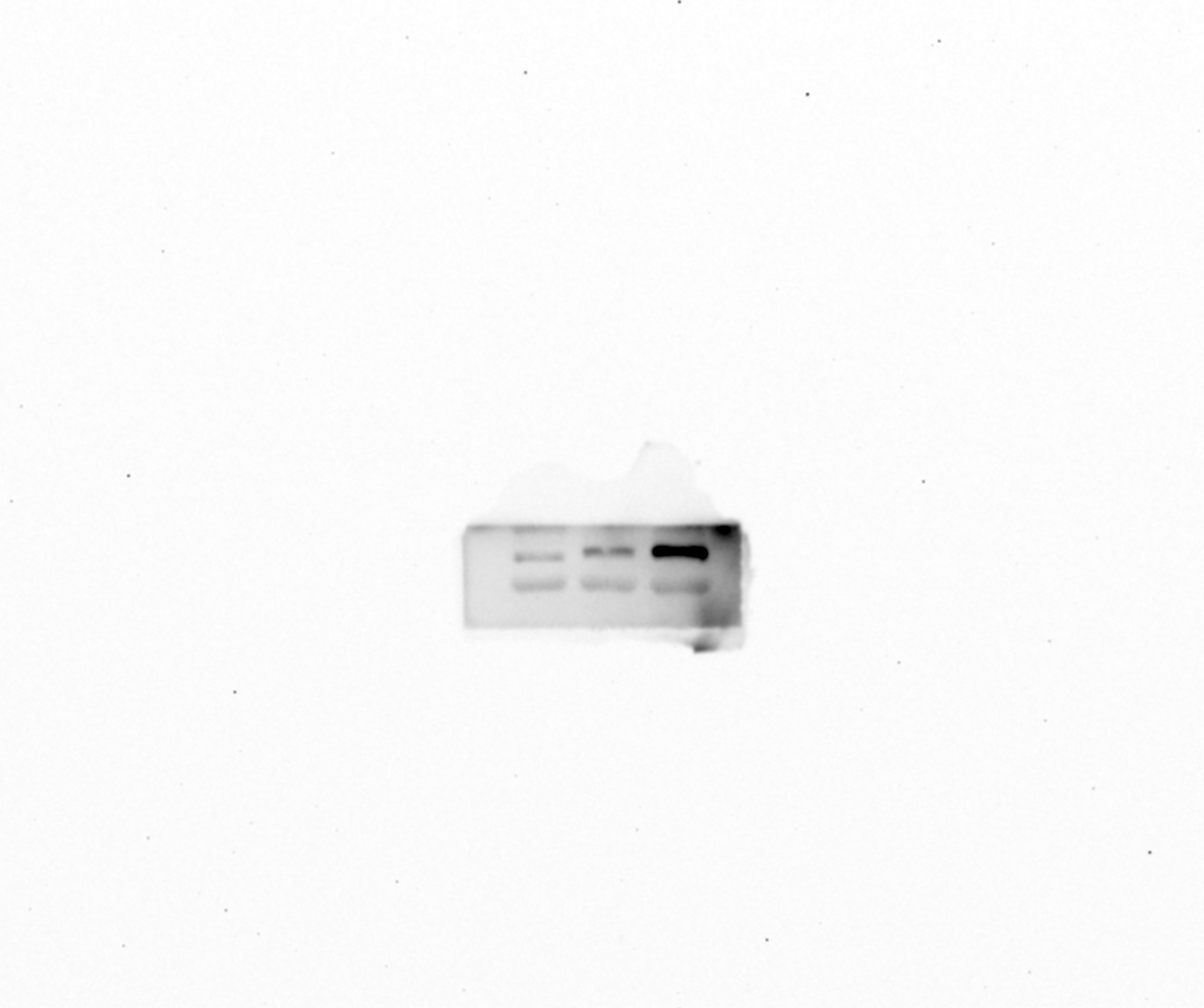

Supplement: Supplementary file 7 — Source data Fig. 4 [file 44319_2024_352_MOESM7_ESM.zip › Figure 4/4H/4H-1/western HA IP.tif]

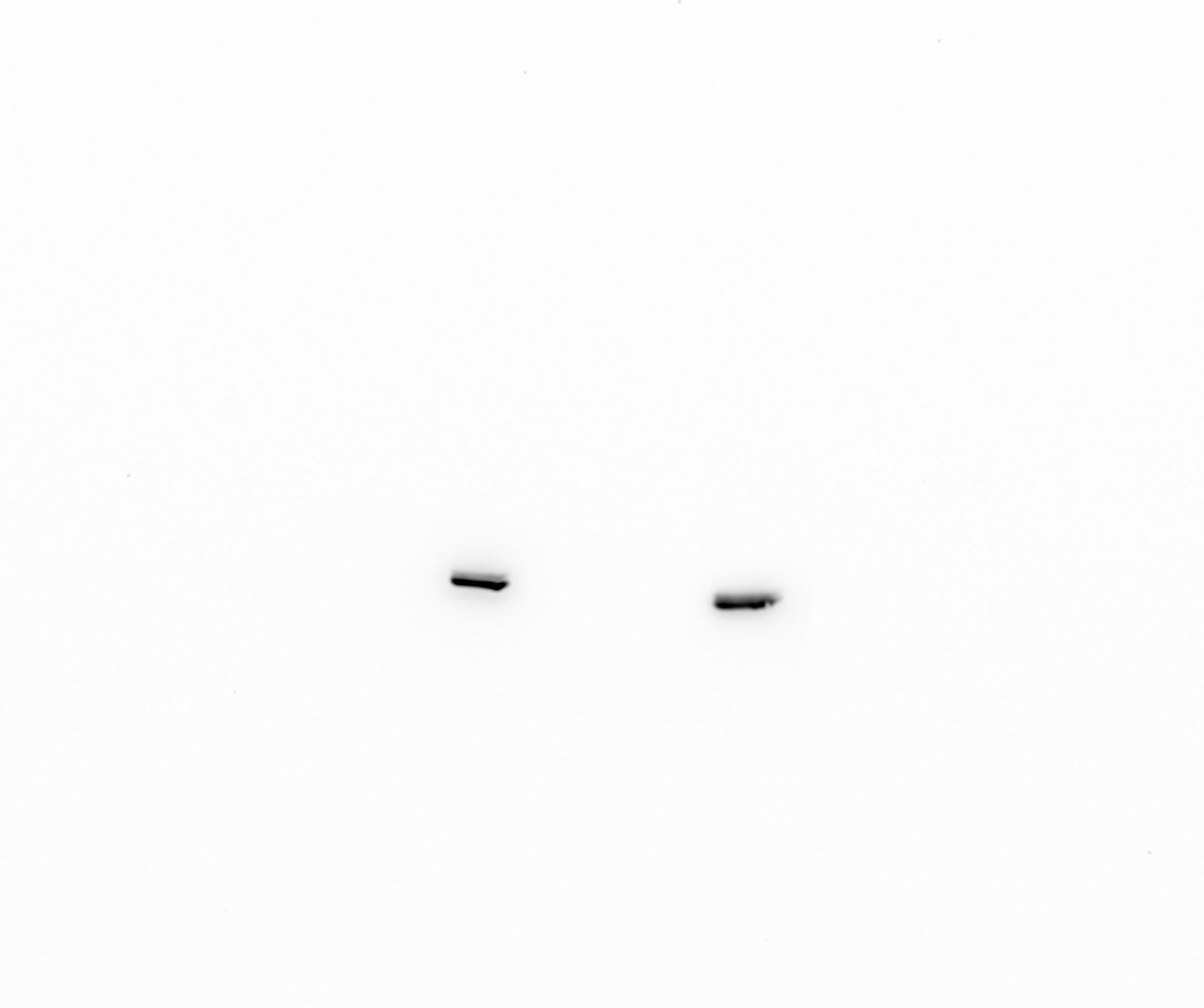

Supplement: Supplementary file 7 — Source data Fig. 4 [file 44319_2024_352_MOESM7_ESM.zip › Figure 4/4H/4H-1/western myc Input.tif]

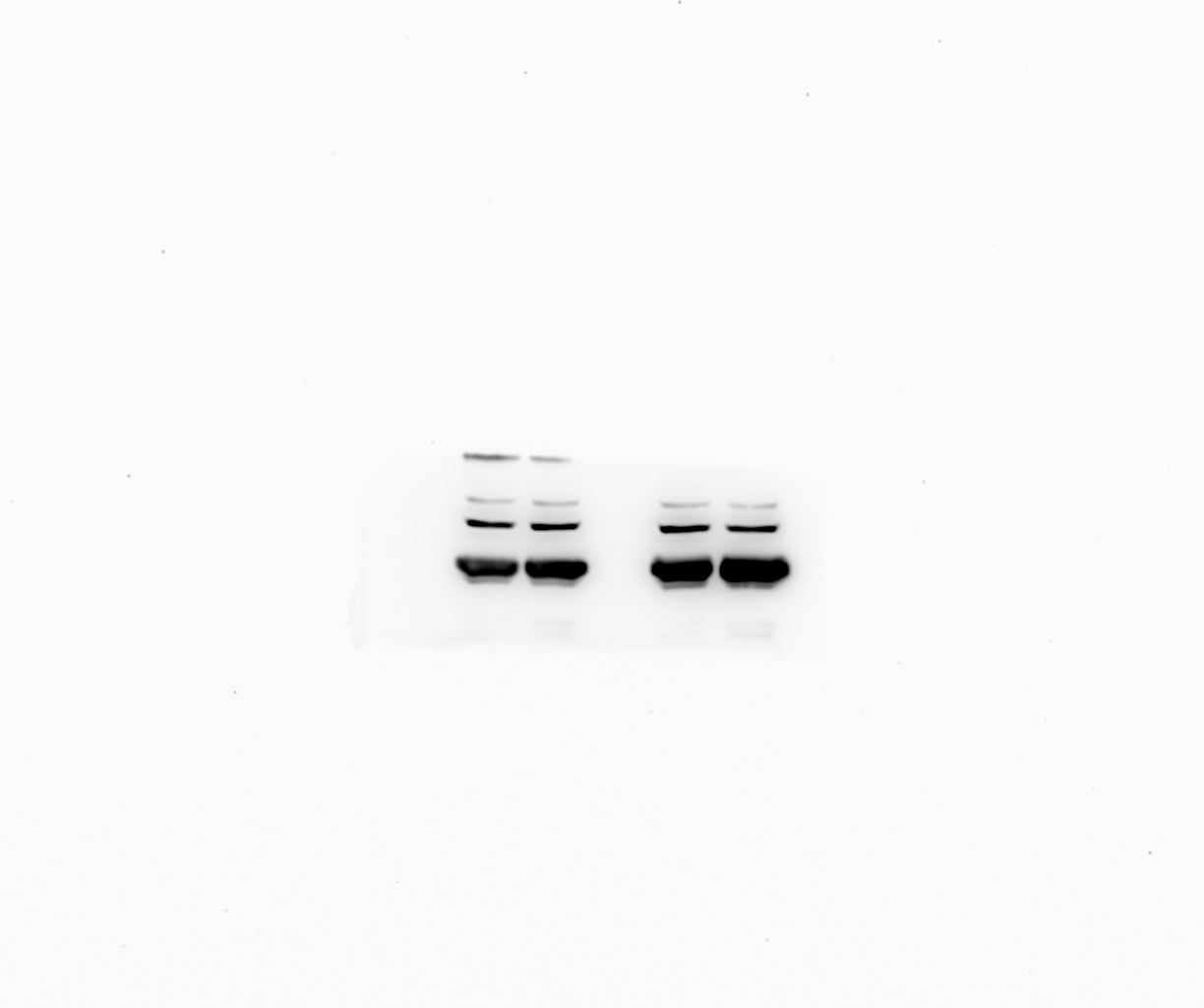

Supplement: Supplementary file 7 — Source data Fig. 4 [file 44319_2024_352_MOESM7_ESM.zip › Figure 4/4H/4H-2/western Flag Input.tif]

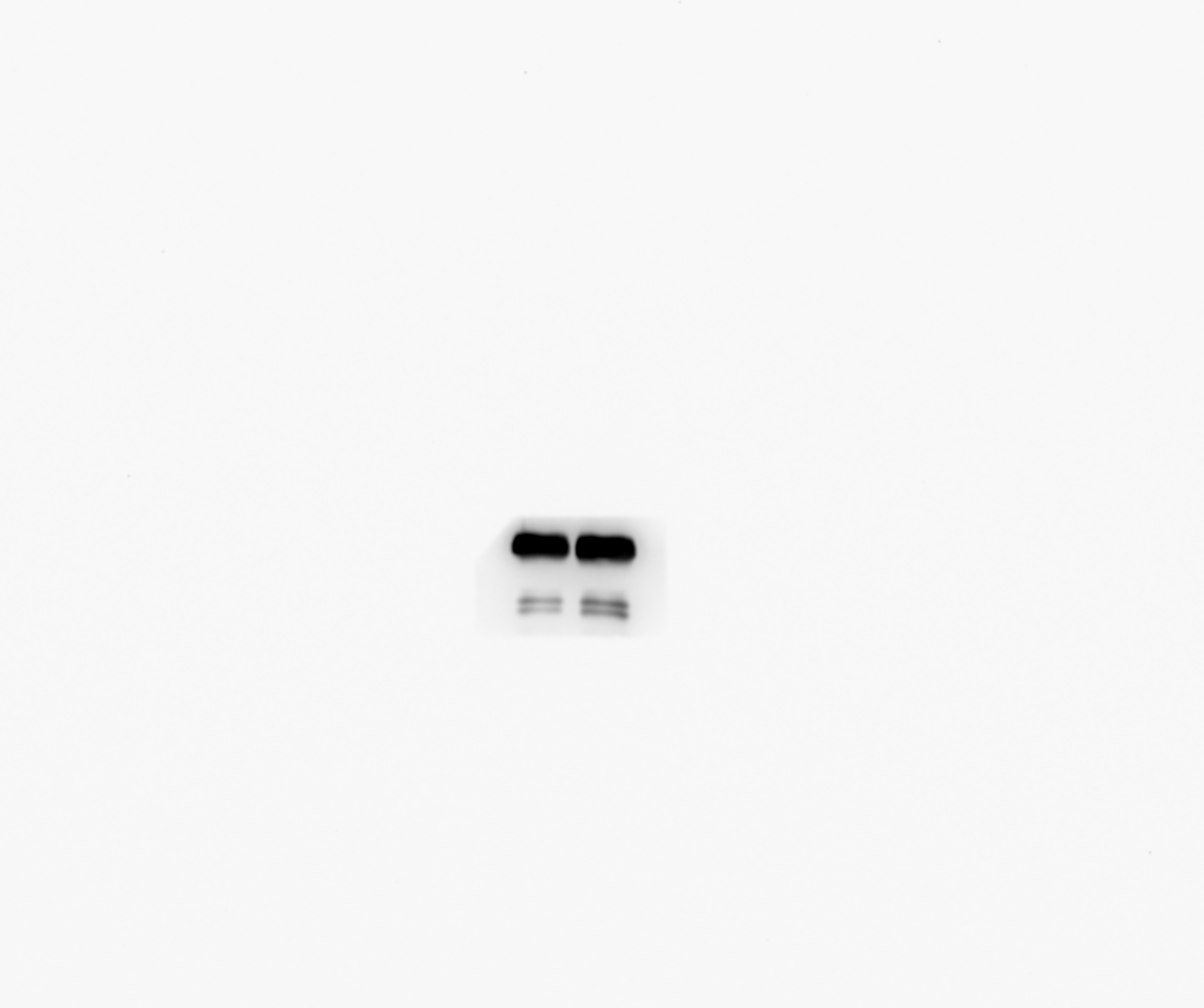

Supplement: Supplementary file 7 — Source data Fig. 4 [file 44319_2024_352_MOESM7_ESM.zip › Figure 4/4H/4H-2/western Flag IP.tif]

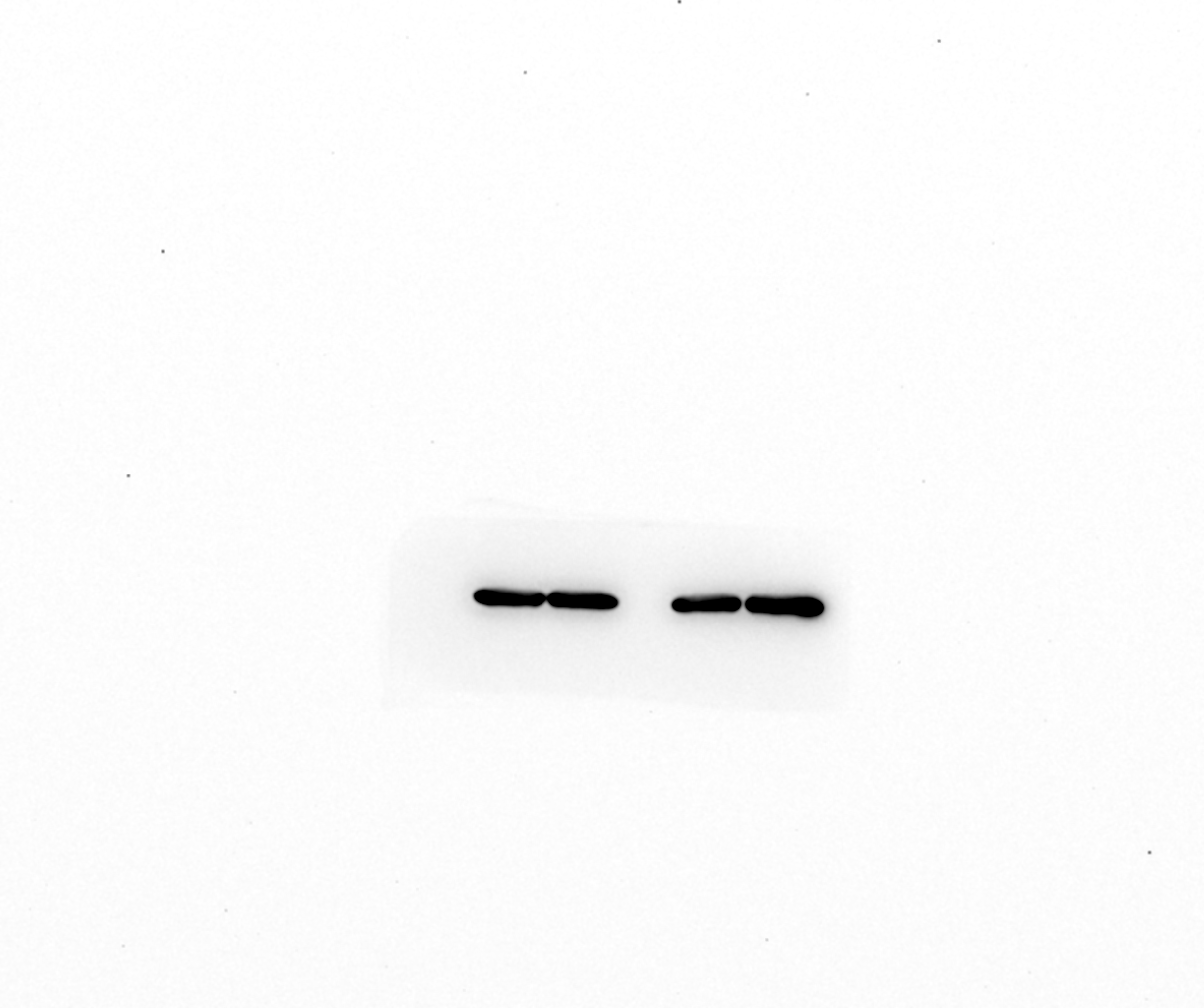

Supplement: Supplementary file 7 — Source data Fig. 4 [file 44319_2024_352_MOESM7_ESM.zip › Figure 4/4H/4H-2/western GAPDH.tif]

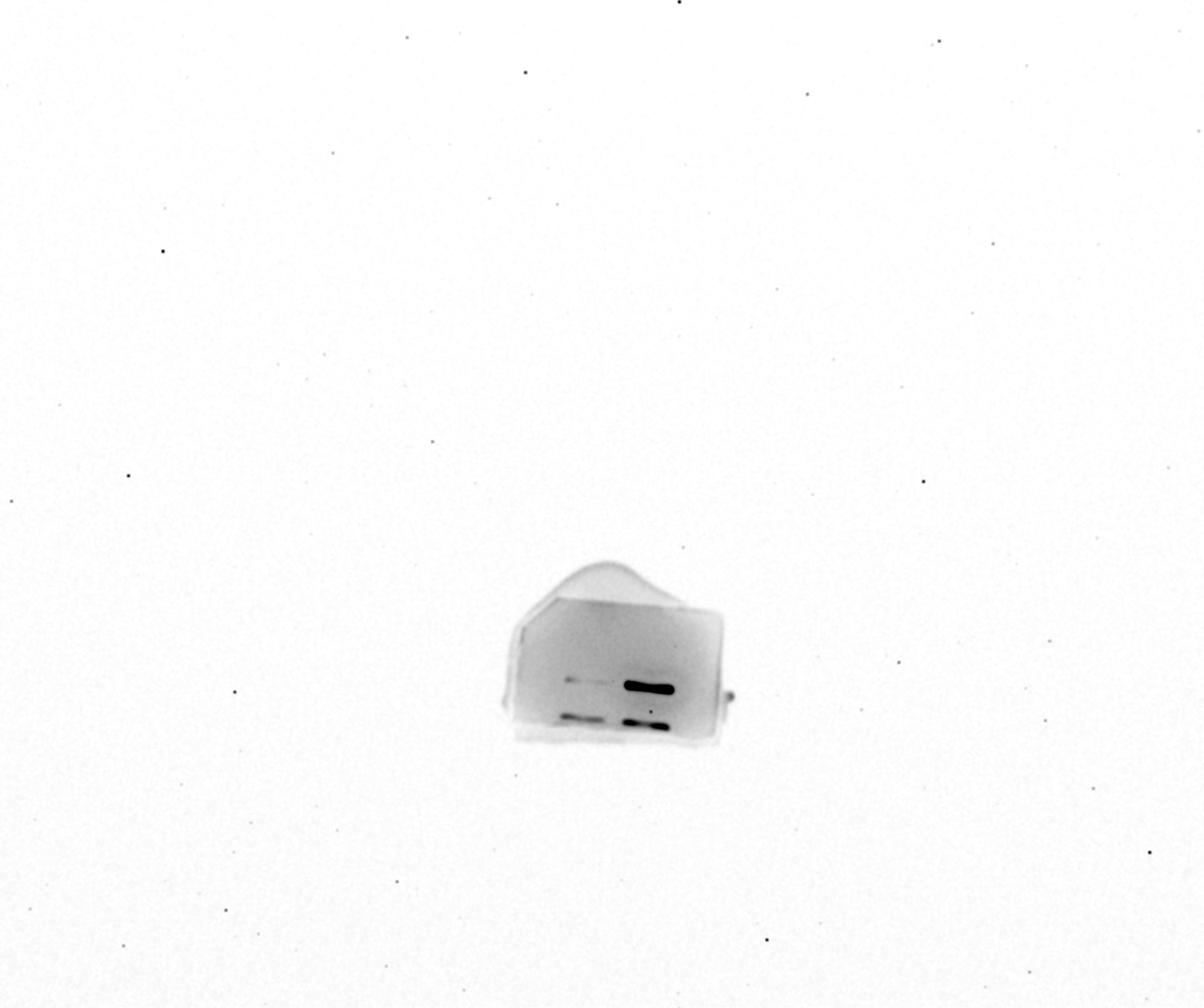

Supplement: Supplementary file 7 — Source data Fig. 4 [file 44319_2024_352_MOESM7_ESM.zip › Figure 4/4H/4H-2/western HA IP.tif]

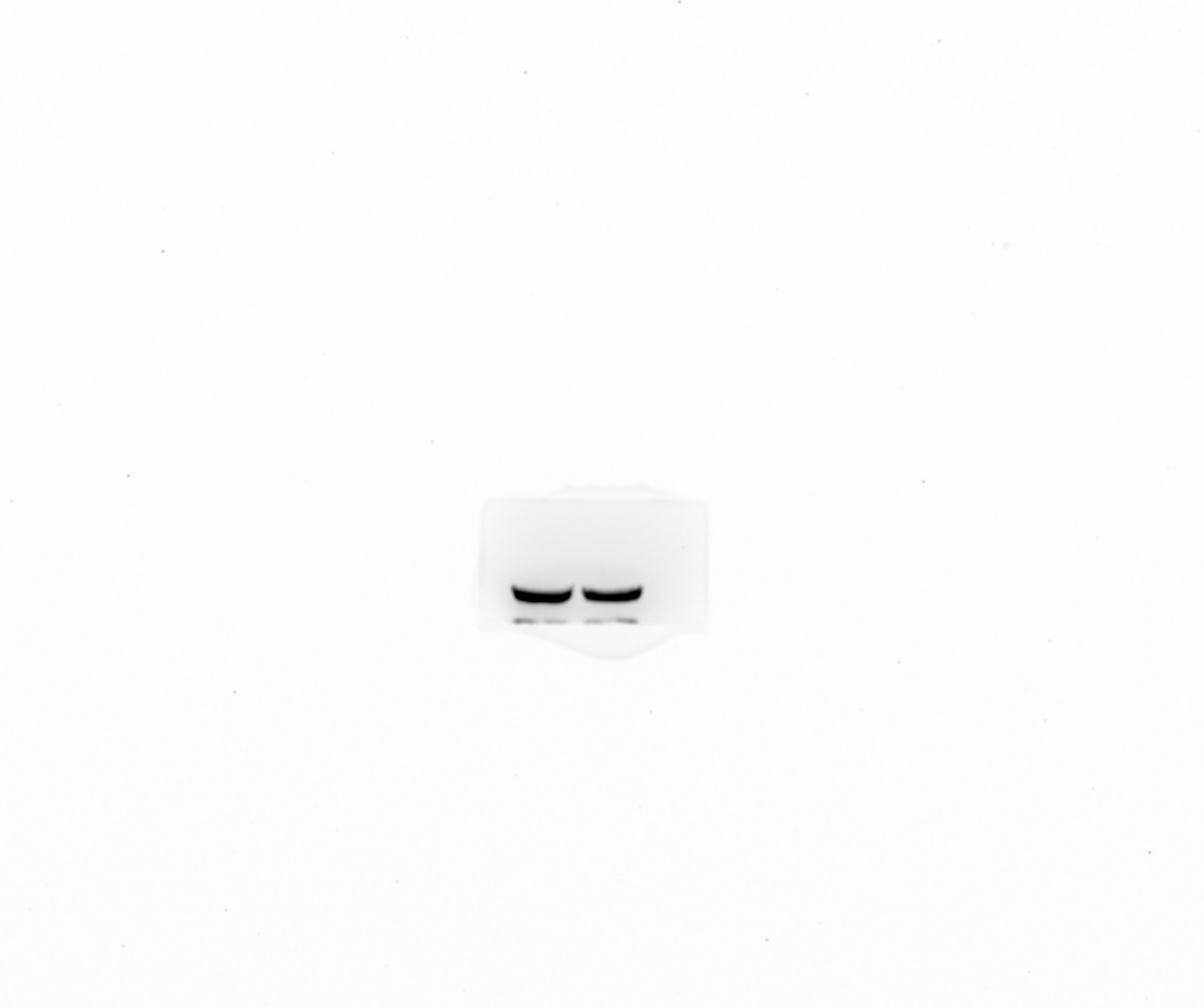

Supplement: Supplementary file 7 — Source data Fig. 4 [file 44319_2024_352_MOESM7_ESM.zip › Figure 4/4H/4H-2/western HA Input.tif]

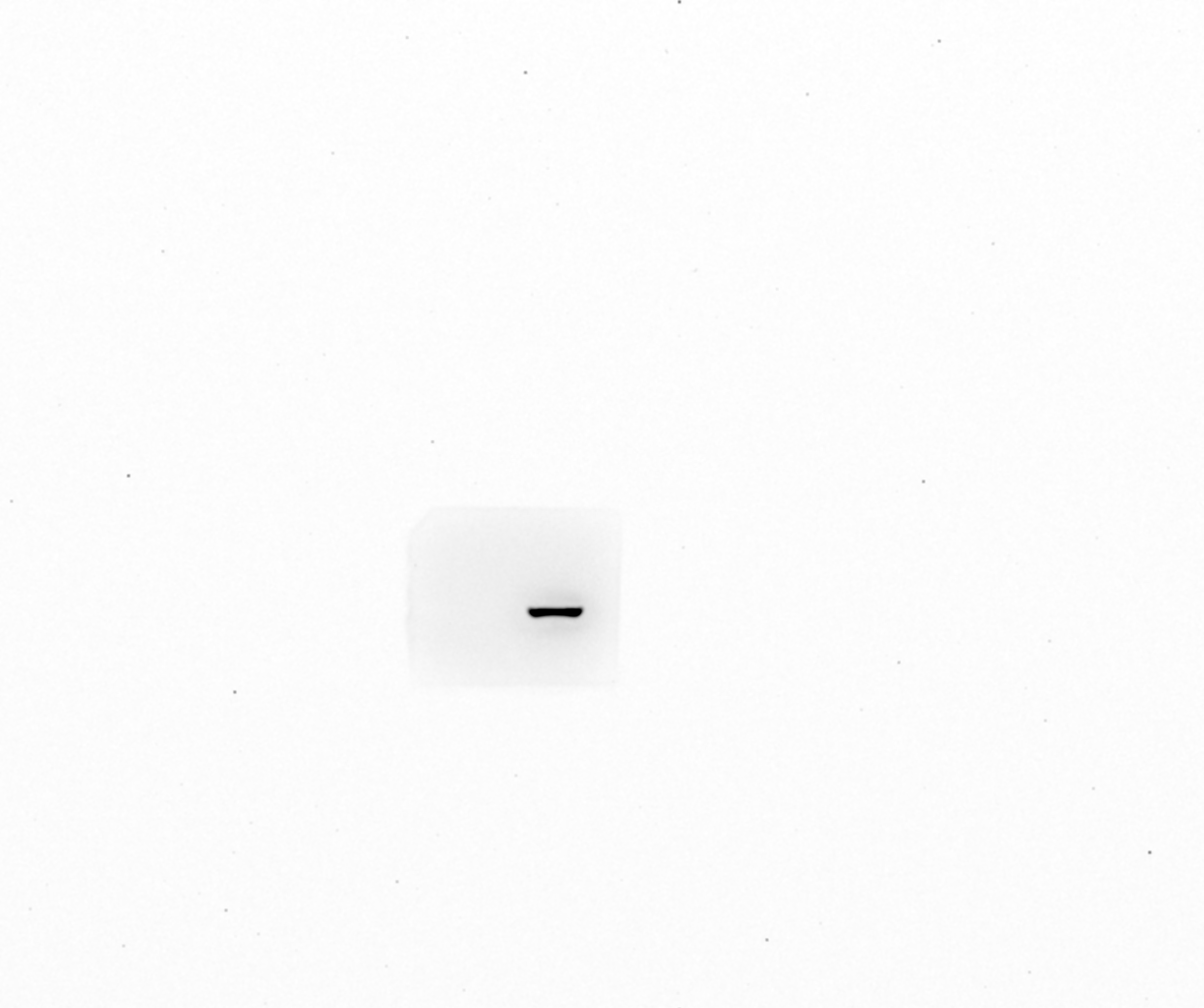

Supplement: Supplementary file 7 — Source data Fig. 4 [file 44319_2024_352_MOESM7_ESM.zip › Figure 4/4H/4H-2/western myc Input.tif]

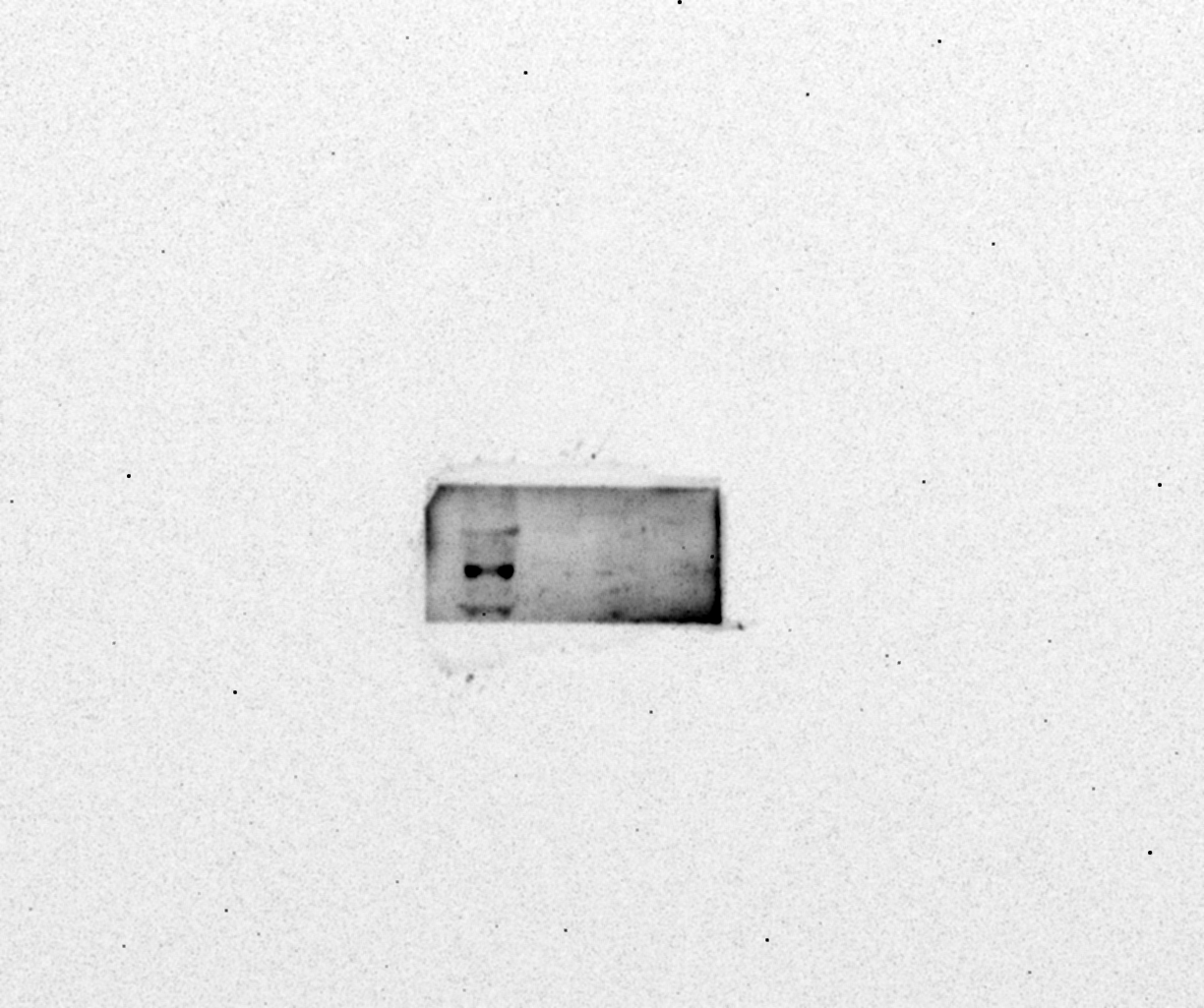

Supplement: Supplementary file 7 — Source data Fig. 4 [file 44319_2024_352_MOESM7_ESM.zip › Figure 4/4I/western DYRK4.tif]

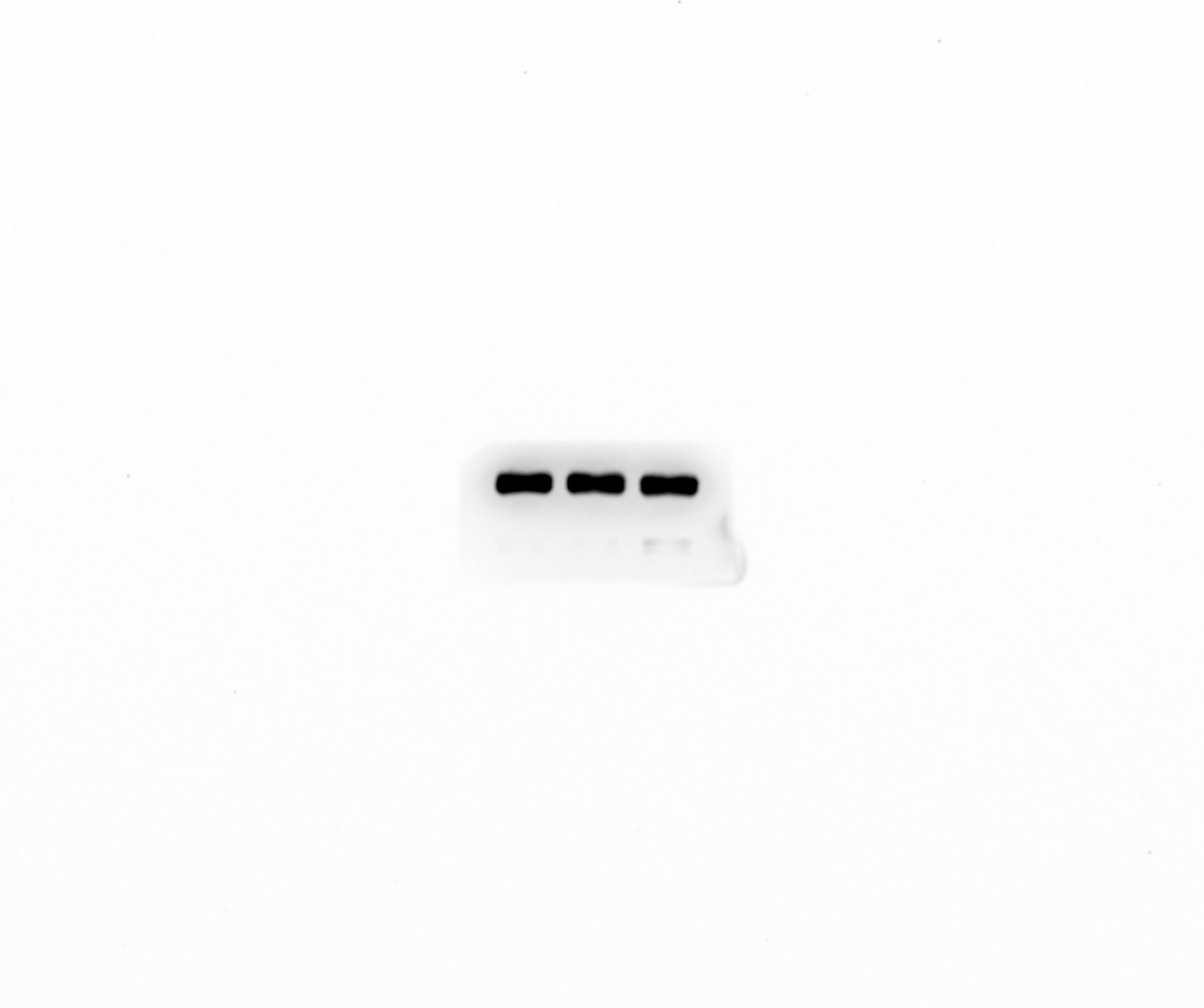

Supplement: Supplementary file 7 — Source data Fig. 4 [file 44319_2024_352_MOESM7_ESM.zip › Figure 4/4I/western Flag IP.tif]

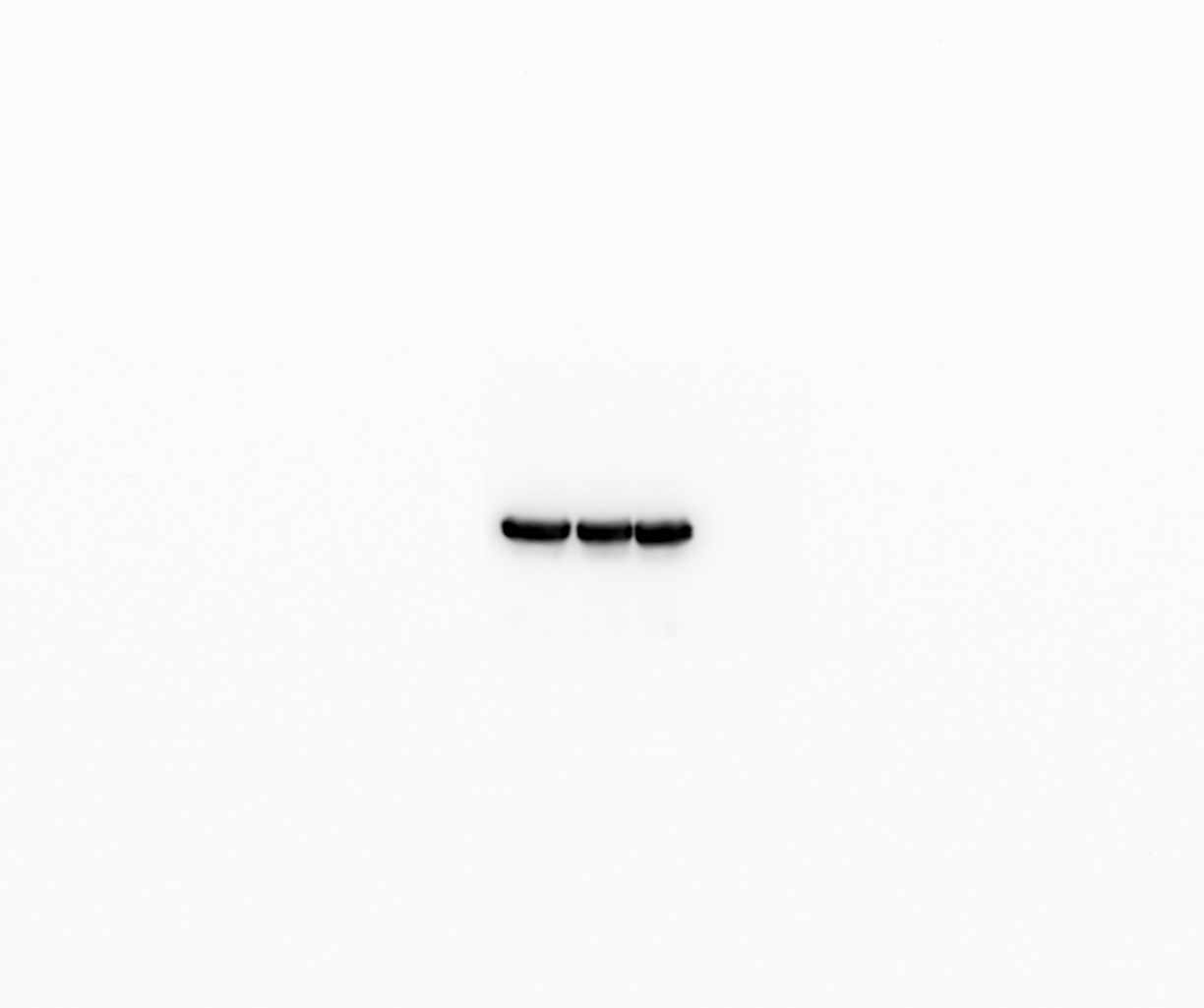

Supplement: Supplementary file 7 — Source data Fig. 4 [file 44319_2024_352_MOESM7_ESM.zip › Figure 4/4I/western Flag Input.tif]

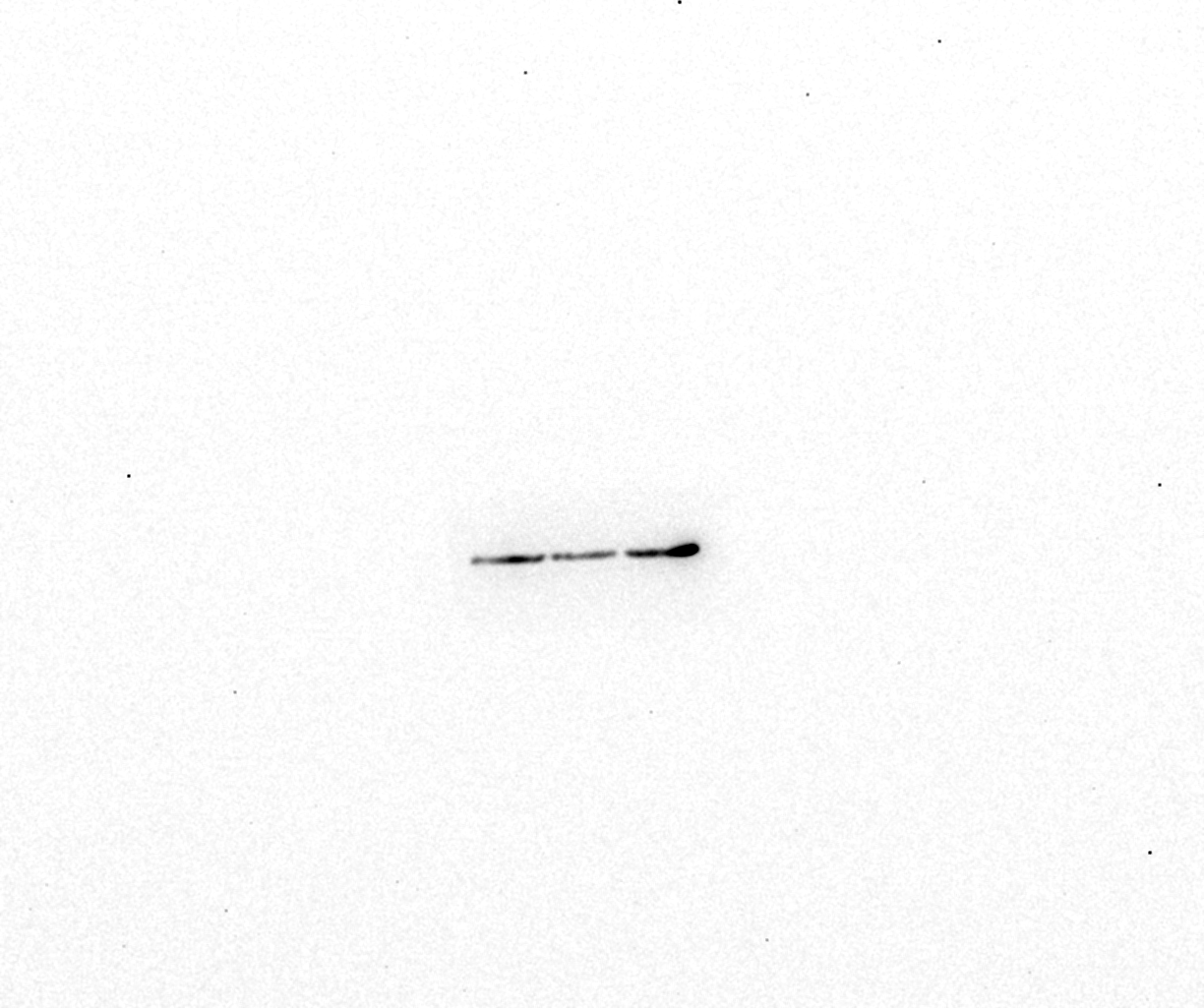

Supplement: Supplementary file 7 — Source data Fig. 4 [file 44319_2024_352_MOESM7_ESM.zip › Figure 4/4I/western GAPDH Input.tif]

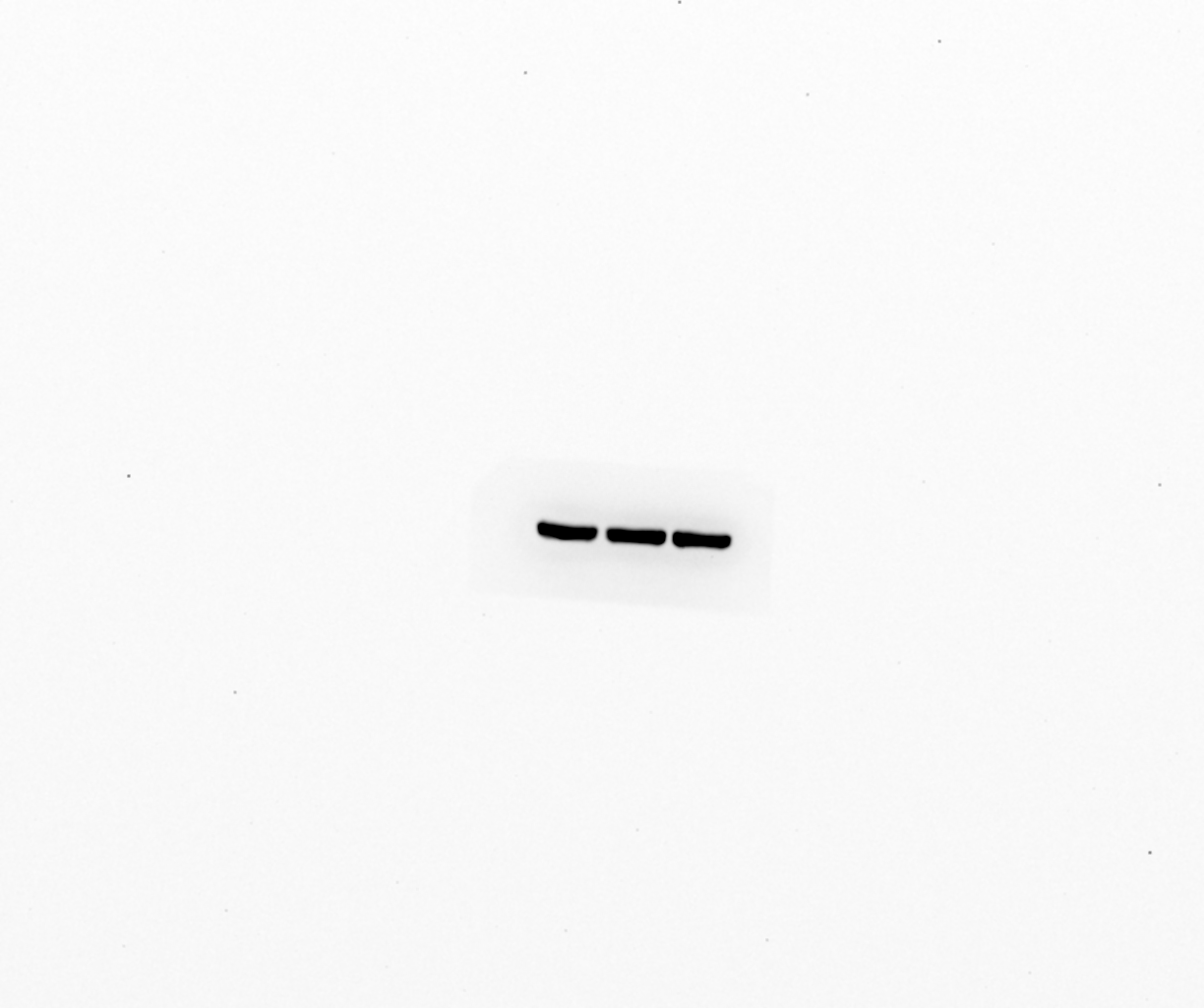

Supplement: Supplementary file 7 — Source data Fig. 4 [file 44319_2024_352_MOESM7_ESM.zip › Figure 4/4I/western HA Input.tif]

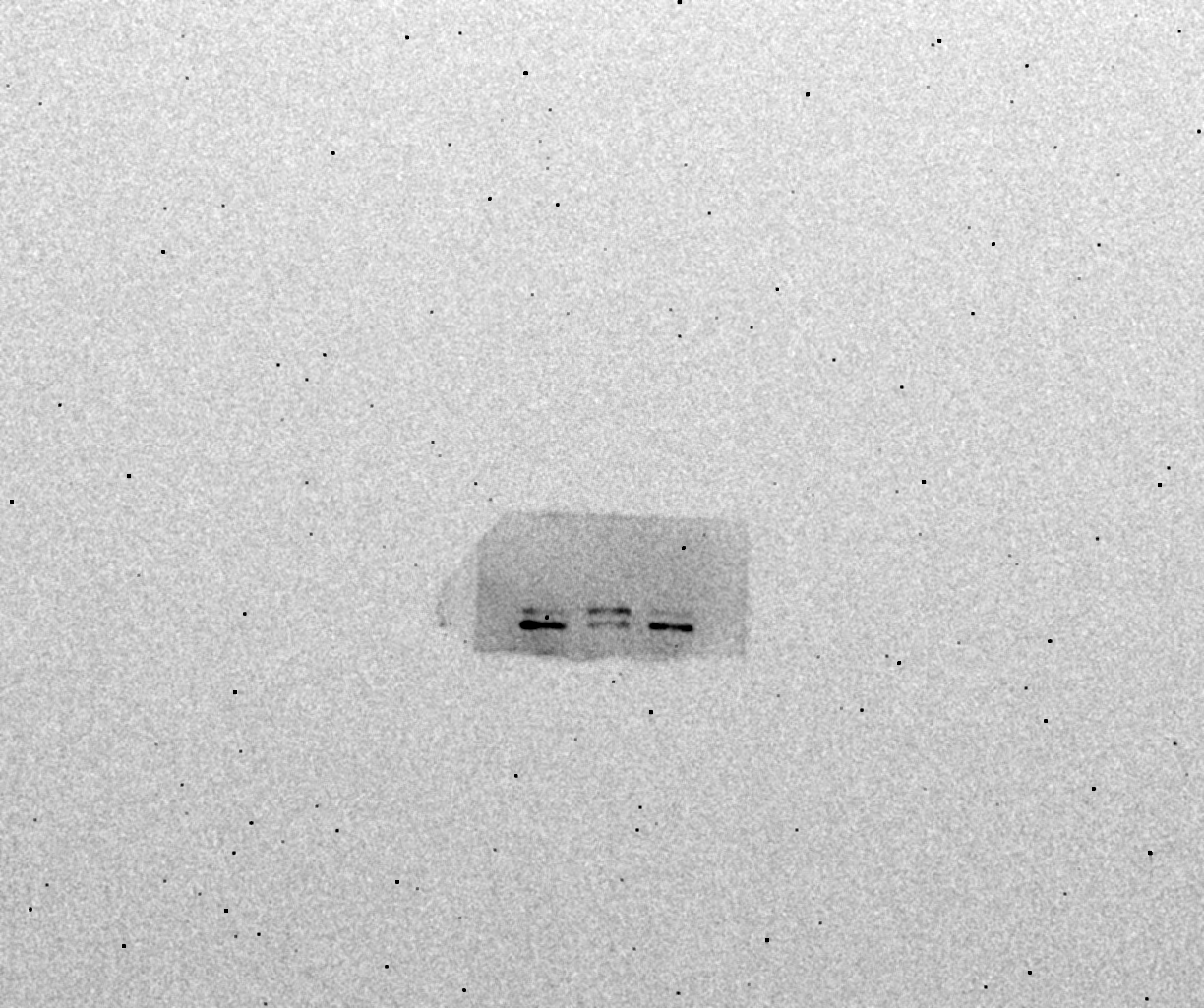

Supplement: Supplementary file 7 — Source data Fig. 4 [file 44319_2024_352_MOESM7_ESM.zip › Figure 4/4I/western TBK1 IP.tif]

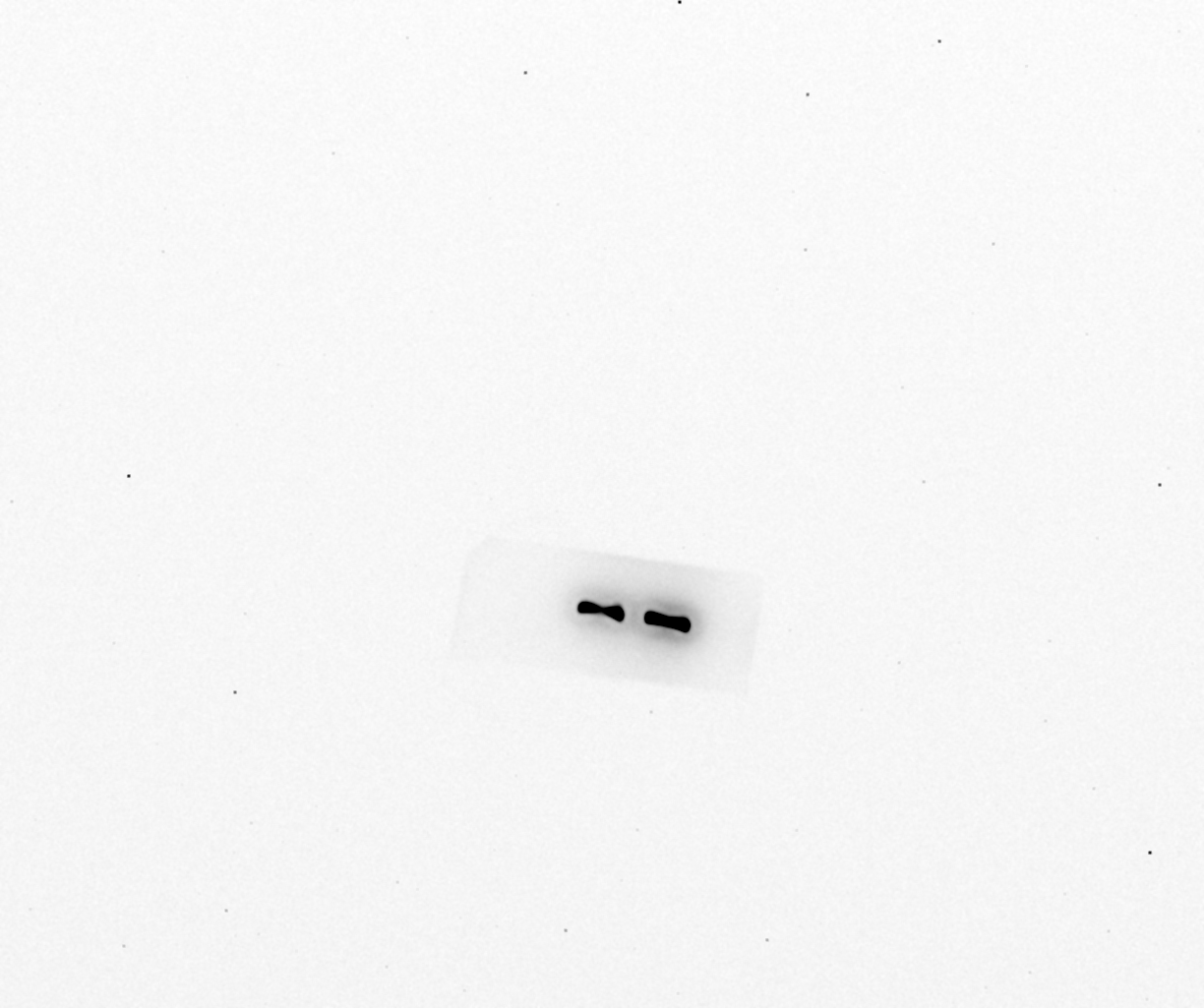

Supplement: Supplementary file 7 — Source data Fig. 4 [file 44319_2024_352_MOESM7_ESM.zip › Figure 4/4J/western Flag IP.tif]

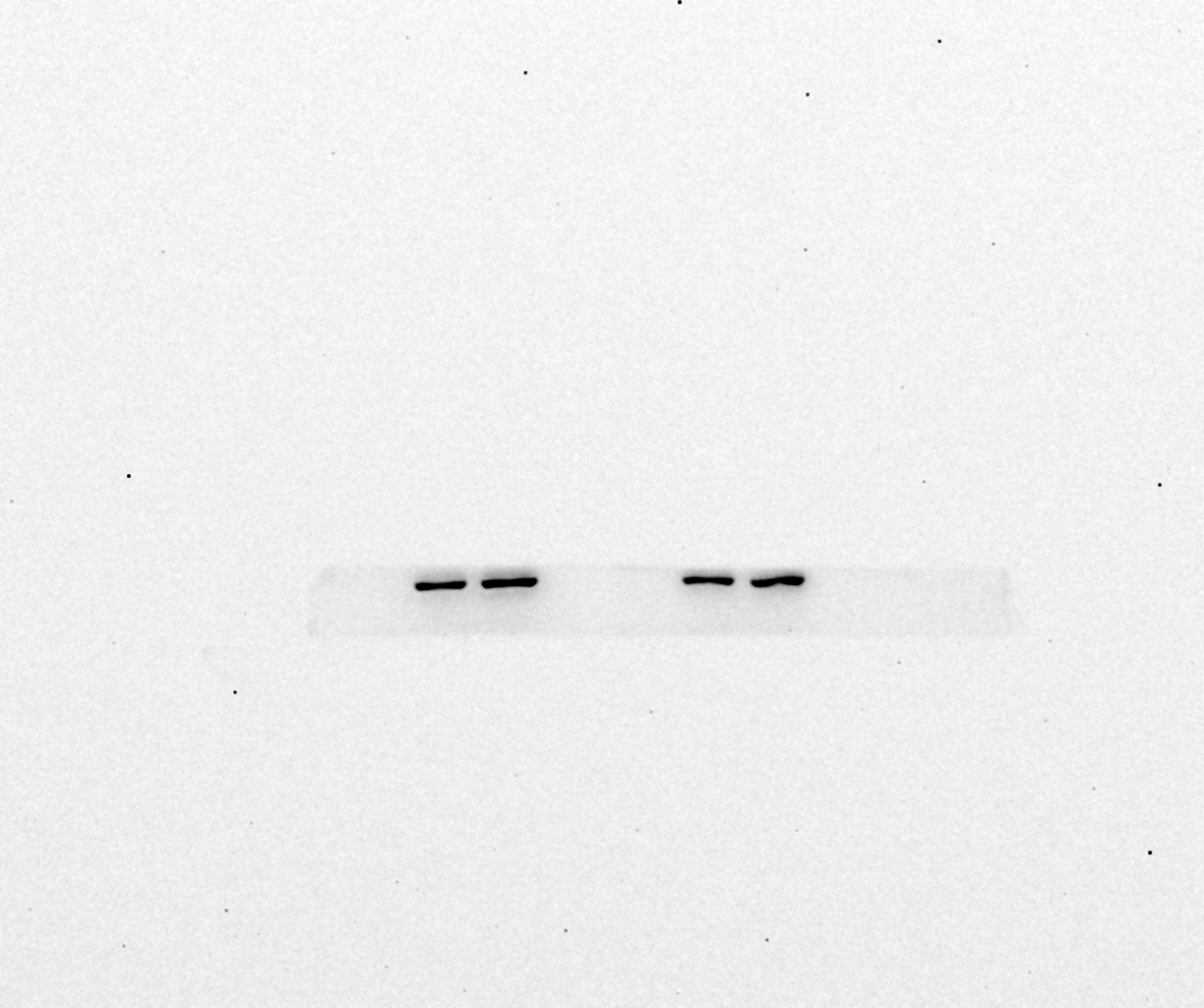

Supplement: Supplementary file 7 — Source data Fig. 4 [file 44319_2024_352_MOESM7_ESM.zip › Figure 4/4J/western Flag.tif]

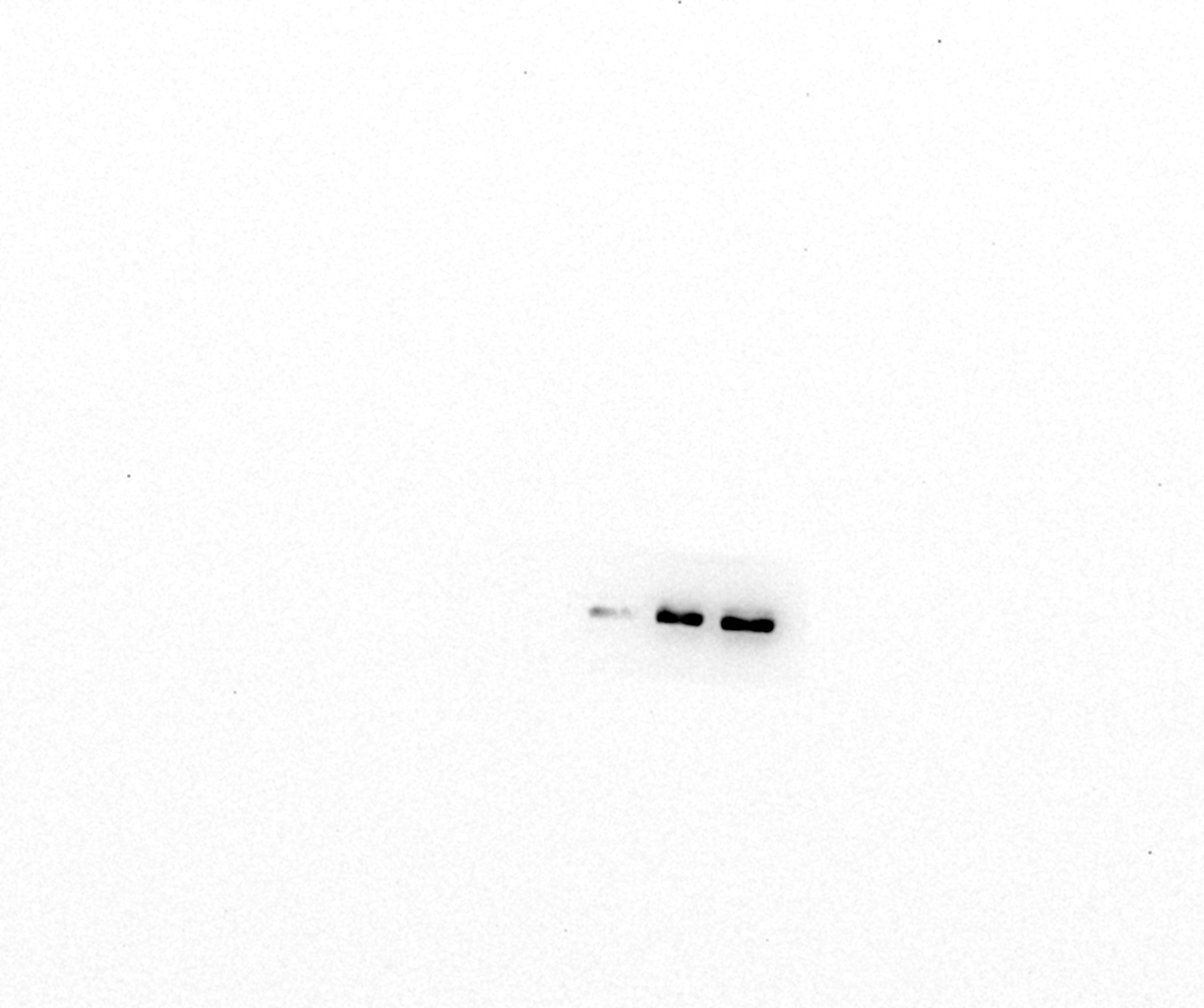

Supplement: Supplementary file 7 — Source data Fig. 4 [file 44319_2024_352_MOESM7_ESM.zip › Figure 4/4J/western HA IP.tif]

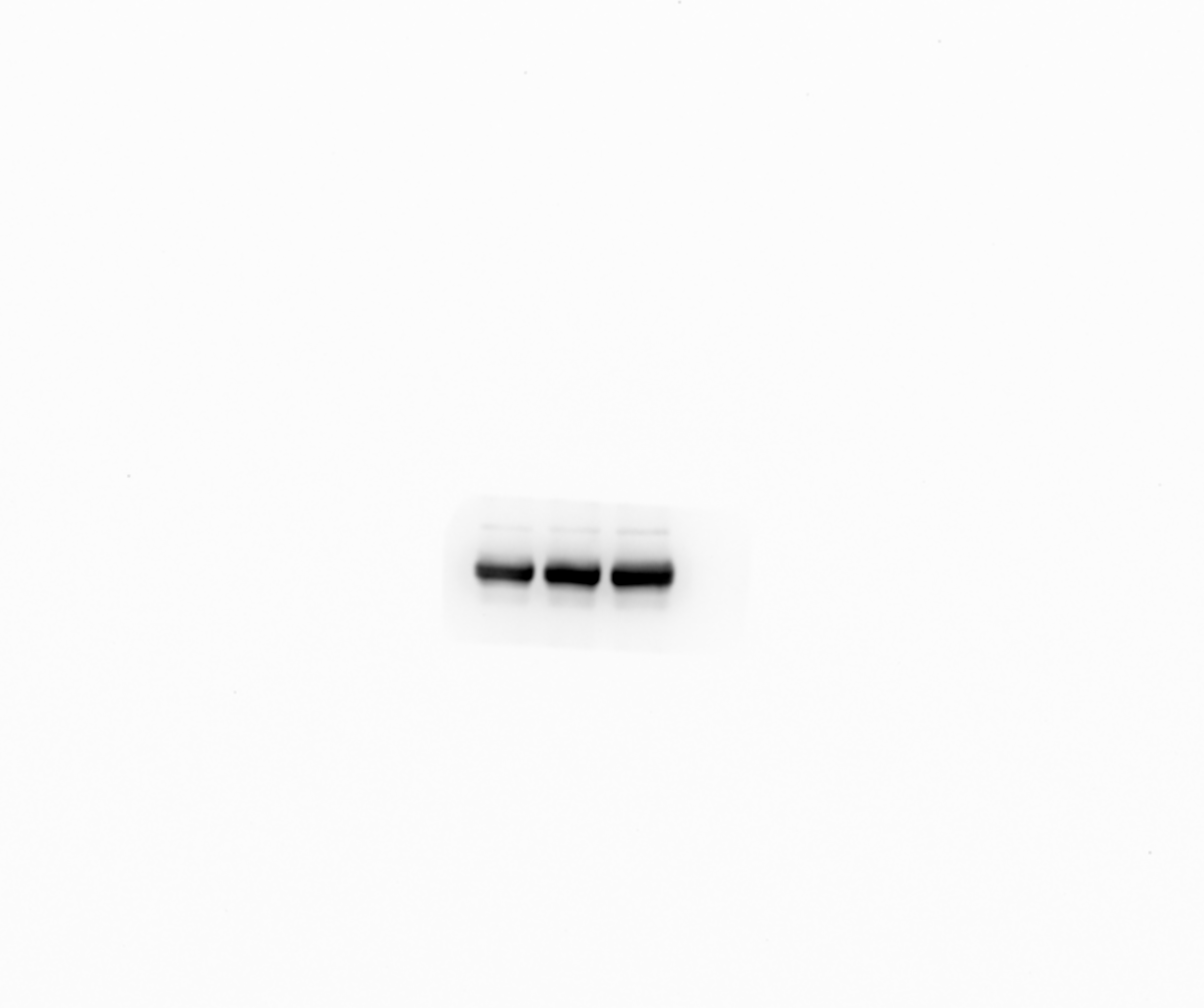

Supplement: Supplementary file 7 — Source data Fig. 4 [file 44319_2024_352_MOESM7_ESM.zip › Figure 4/4J/western HA Input.tif]

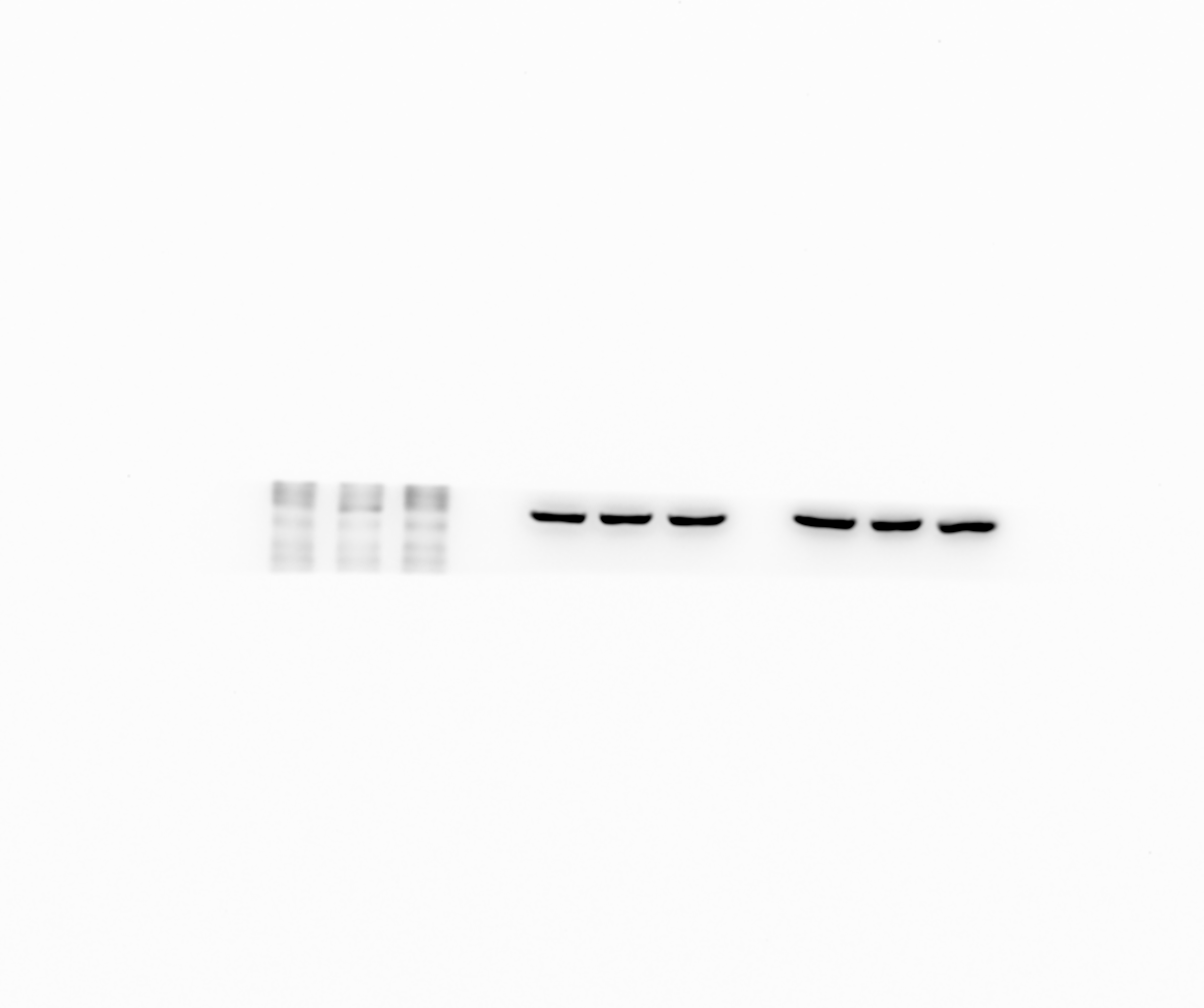

Supplement: Supplementary file 7 — Source data Fig. 4 [file 44319_2024_352_MOESM7_ESM.zip › Figure 4/4J/western actin.tif]

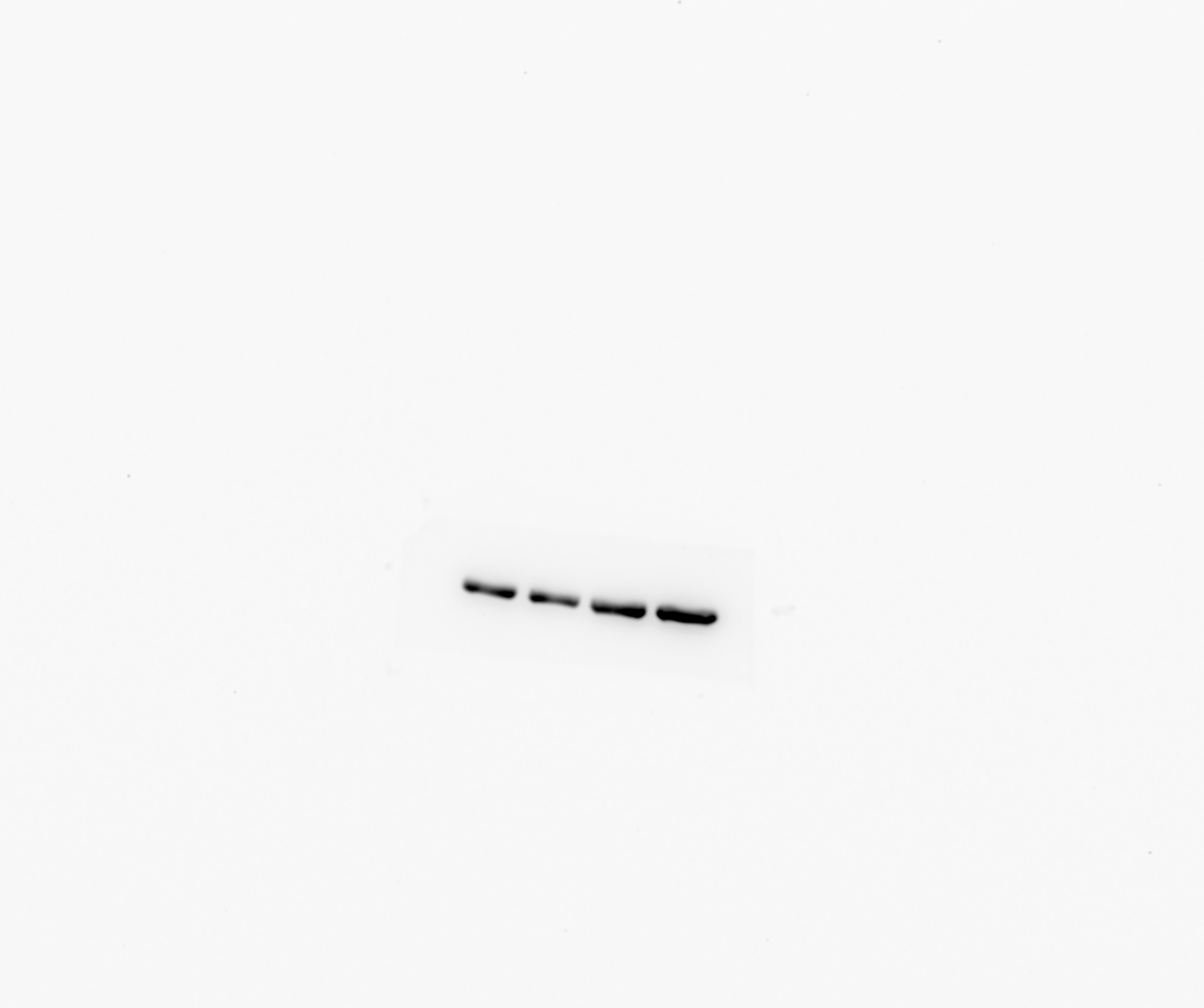

Supplement: Supplementary file 8 — Source data Fig. 5 [file 44319_2024_352_MOESM8_ESM.zip › Figure 5/5A/western Flag.tif]

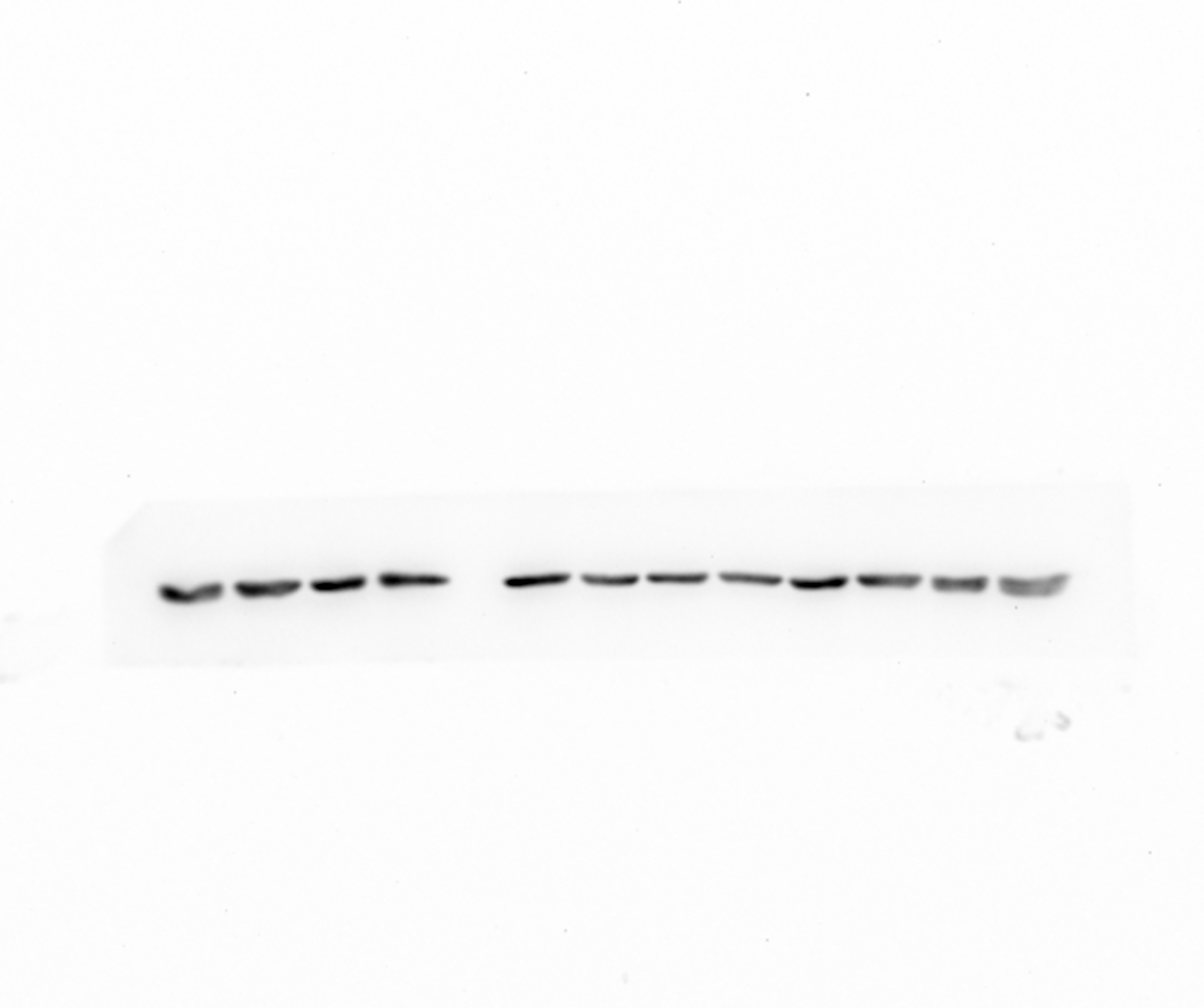

Supplement: Supplementary file 8 — Source data Fig. 5 [file 44319_2024_352_MOESM8_ESM.zip › Figure 5/5A/western GAPDH.tif]

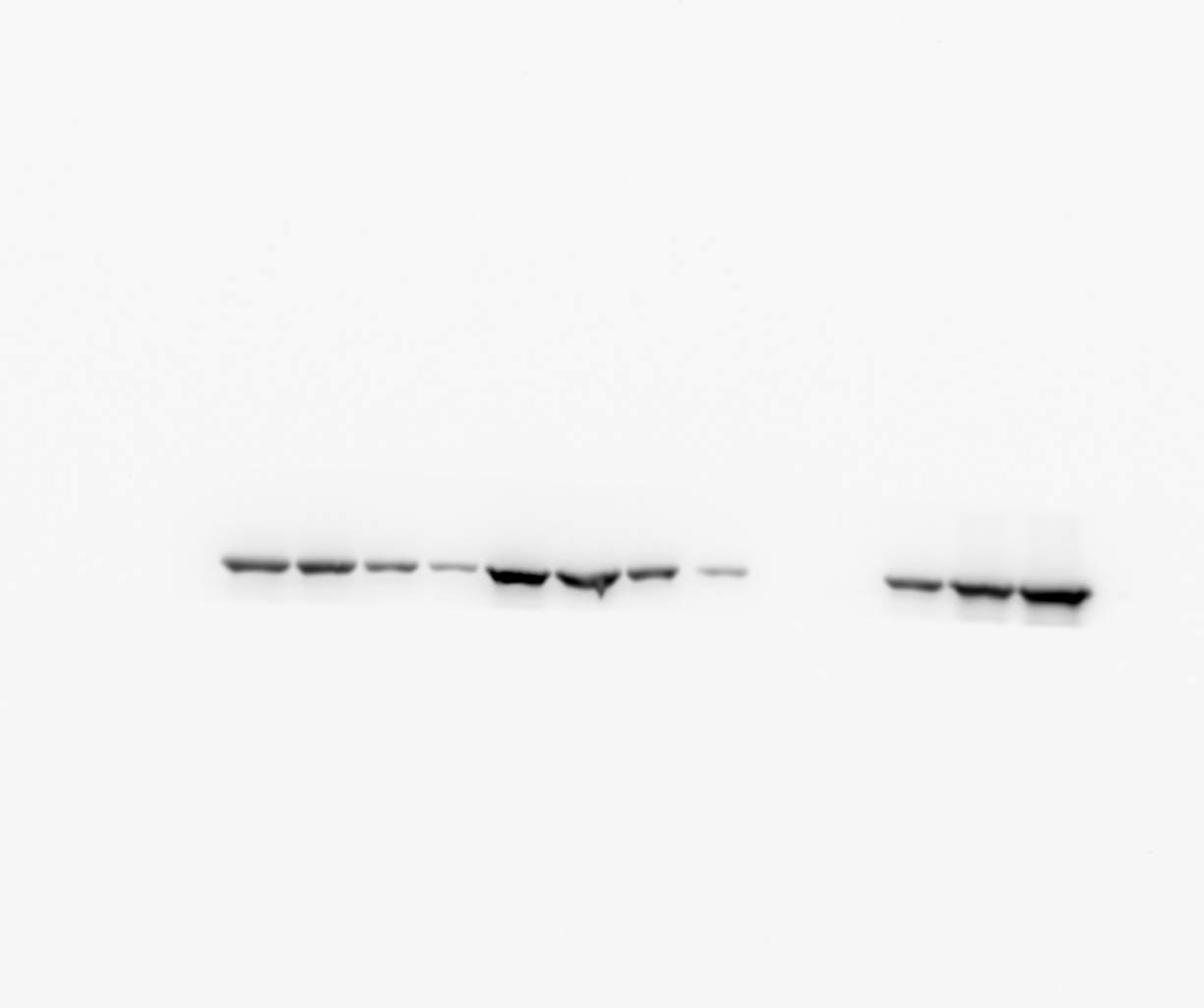

Supplement: Supplementary file 8 — Source data Fig. 5 [file 44319_2024_352_MOESM8_ESM.zip › Figure 5/5A/western GFP.tif]

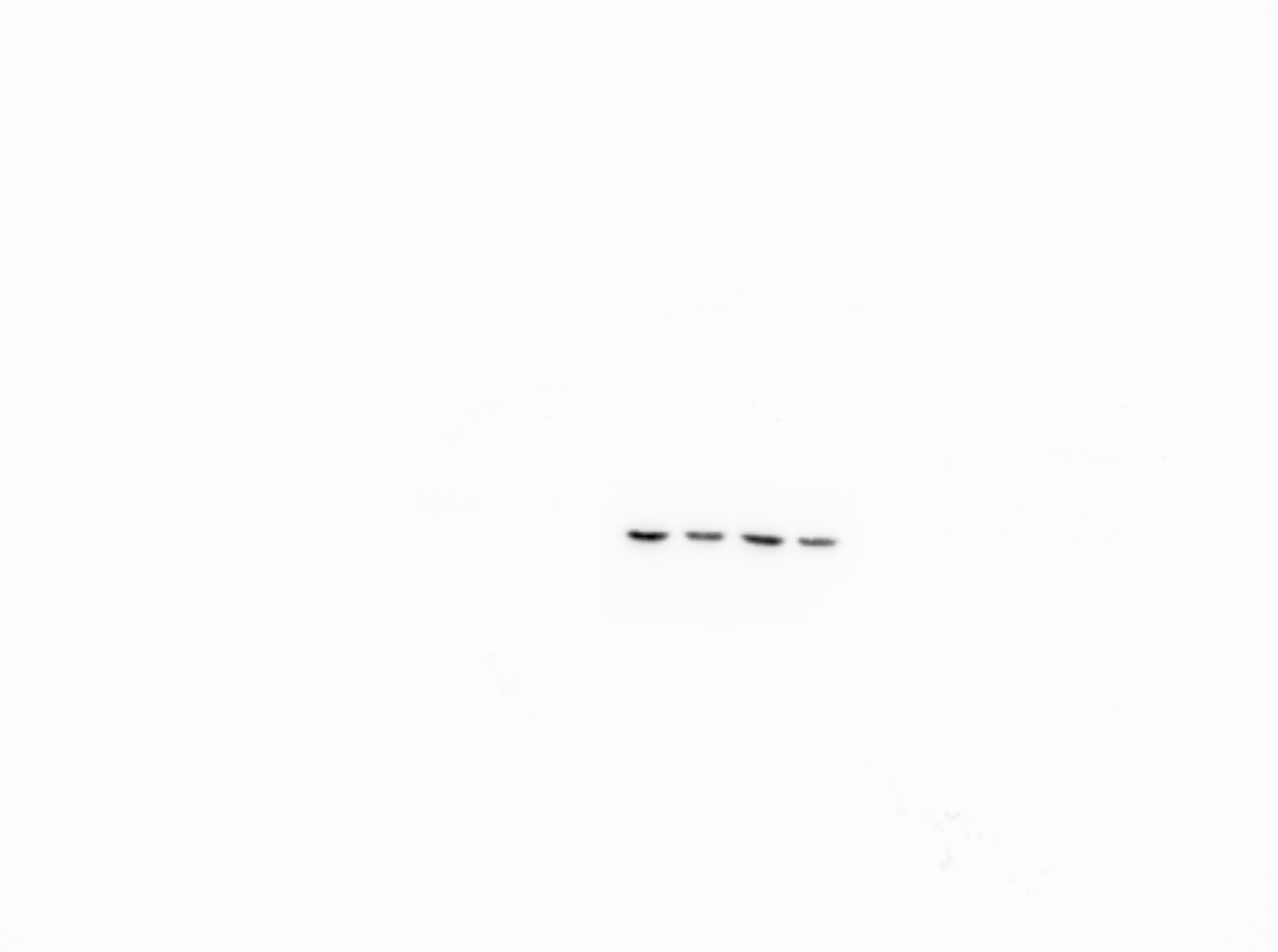

Supplement: Supplementary file 8 — Source data Fig. 5 [file 44319_2024_352_MOESM8_ESM.zip › Figure 5/5B/5B-MT/western Actin.tif]

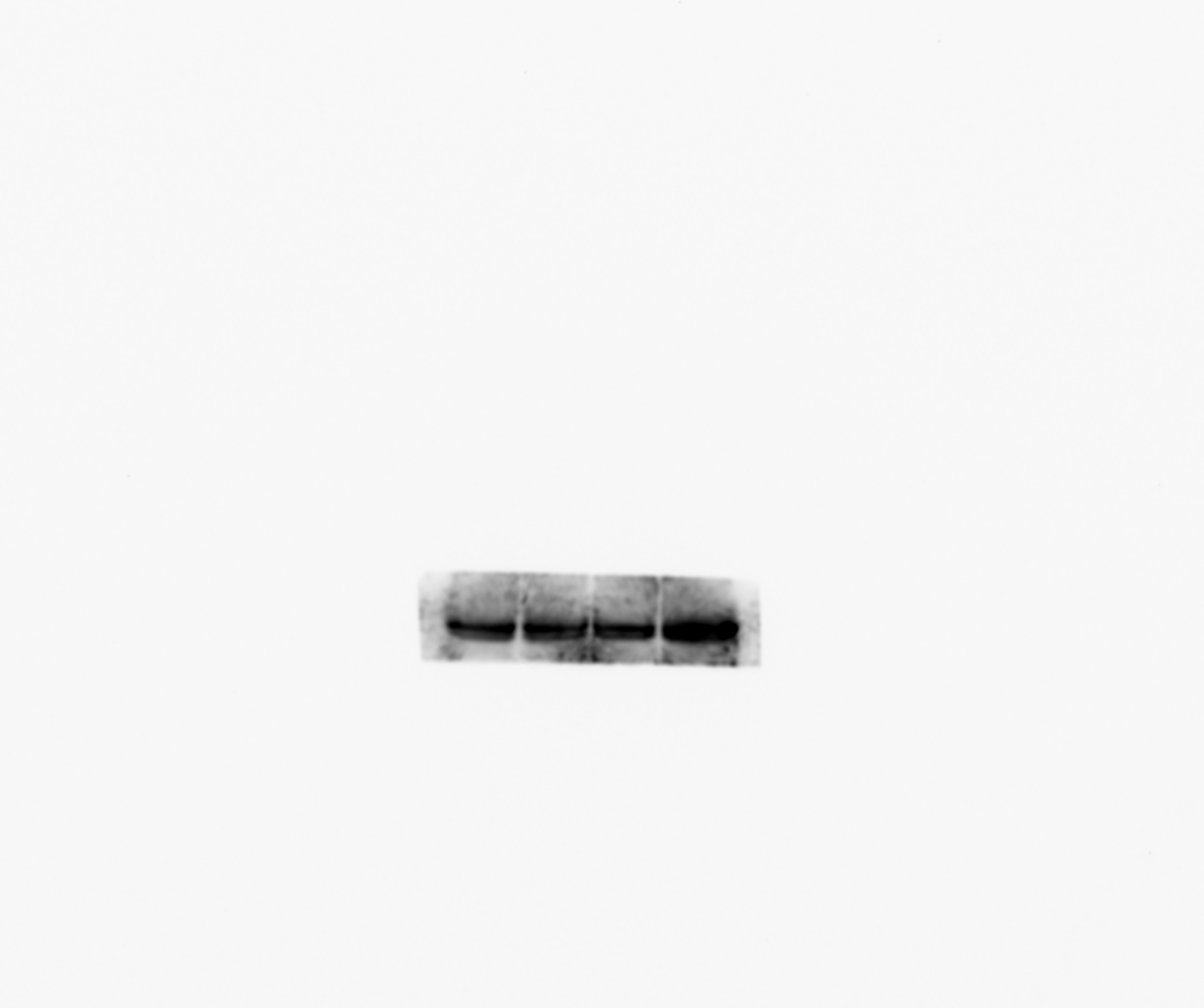

Supplement: Supplementary file 8 — Source data Fig. 5 [file 44319_2024_352_MOESM8_ESM.zip › Figure 5/5B/5B-MT/western IRF3.tif]

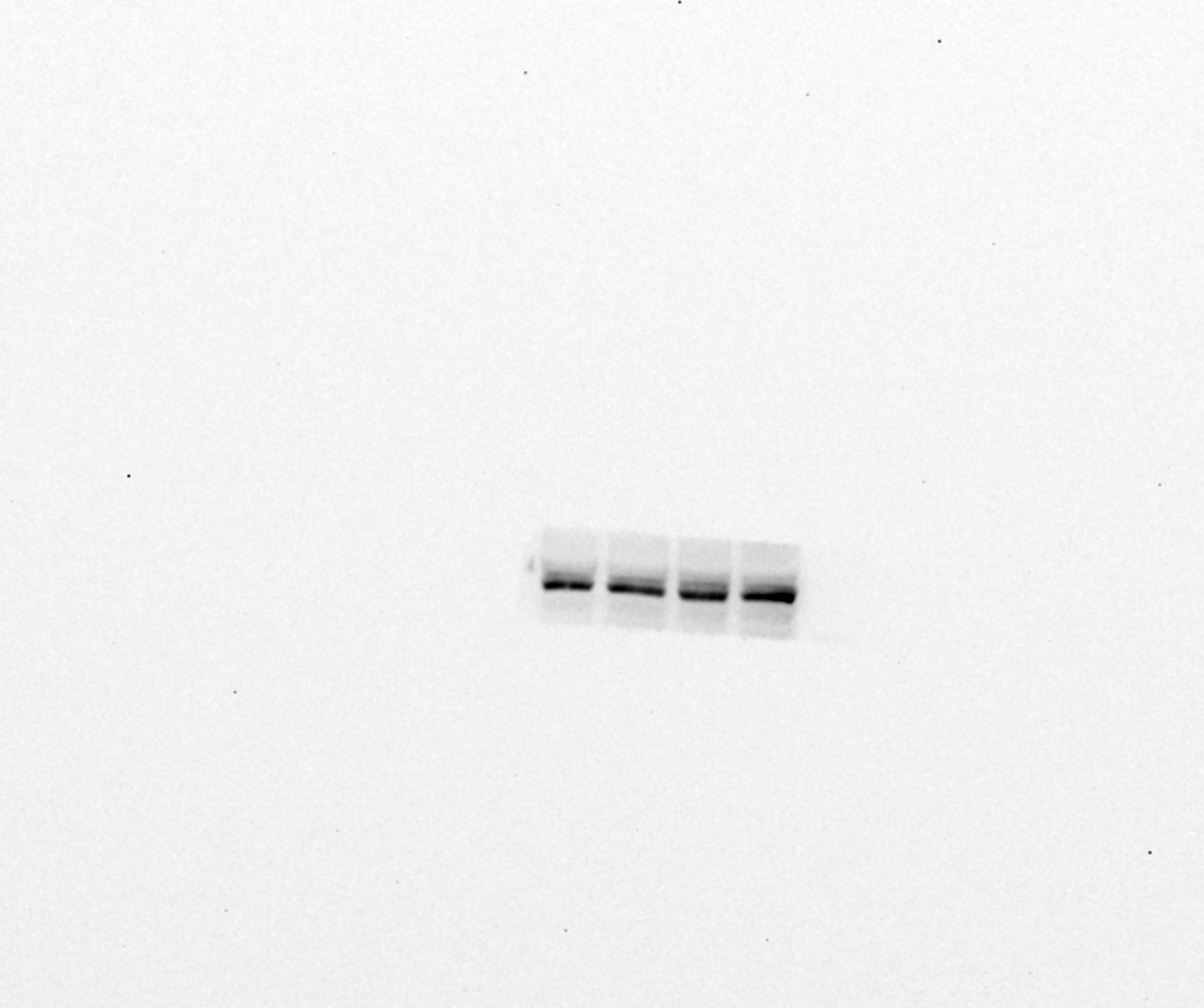

Supplement: Supplementary file 8 — Source data Fig. 5 [file 44319_2024_352_MOESM8_ESM.zip › Figure 5/5B/5B-MT/western TRIM71.tif]

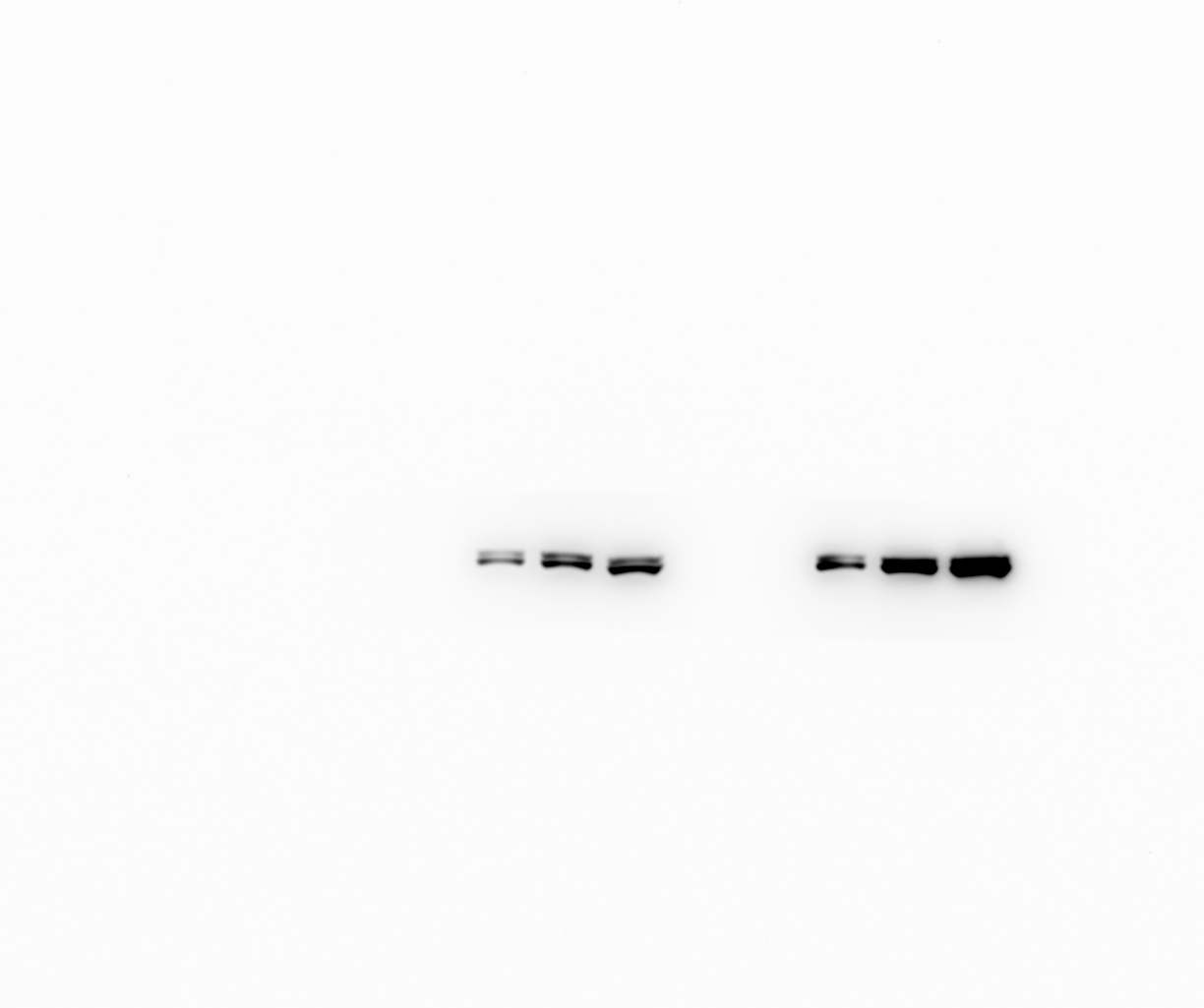

Supplement: Supplementary file 8 — Source data Fig. 5 [file 44319_2024_352_MOESM8_ESM.zip › Figure 5/5B/5B-MT/western myc.tif]

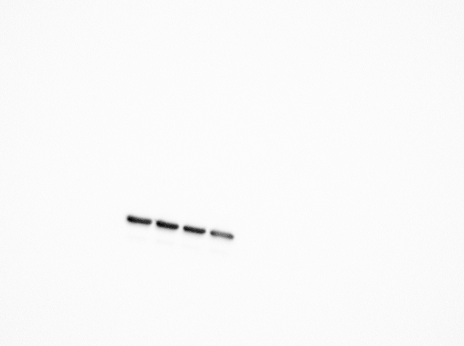

Supplement: Supplementary file 8 — Source data Fig. 5 [file 44319_2024_352_MOESM8_ESM.zip › Figure 5/5B/5B-WT/western ACTIN.tif]

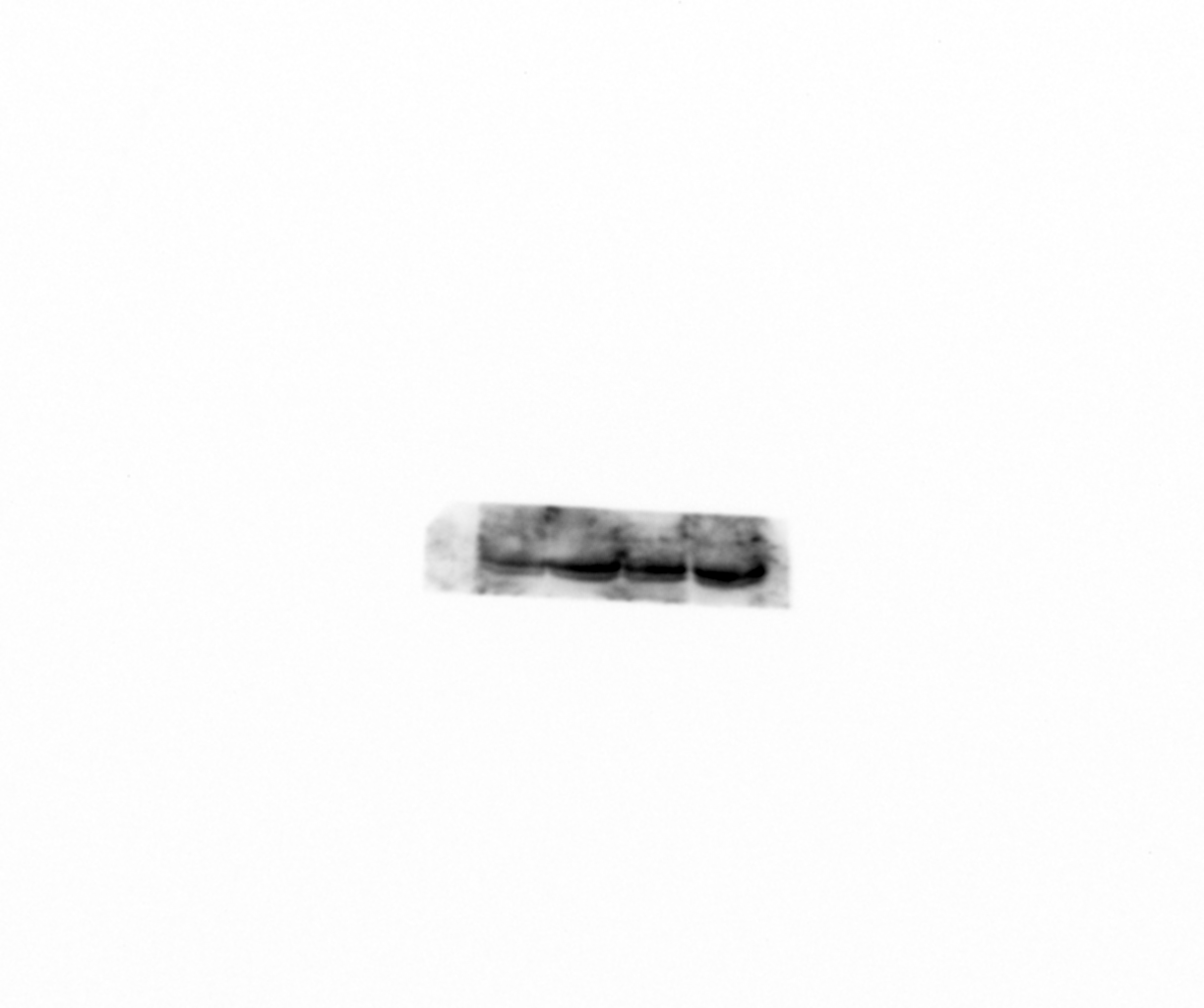

Supplement: Supplementary file 8 — Source data Fig. 5 [file 44319_2024_352_MOESM8_ESM.zip › Figure 5/5B/5B-WT/western IRF3.tif]

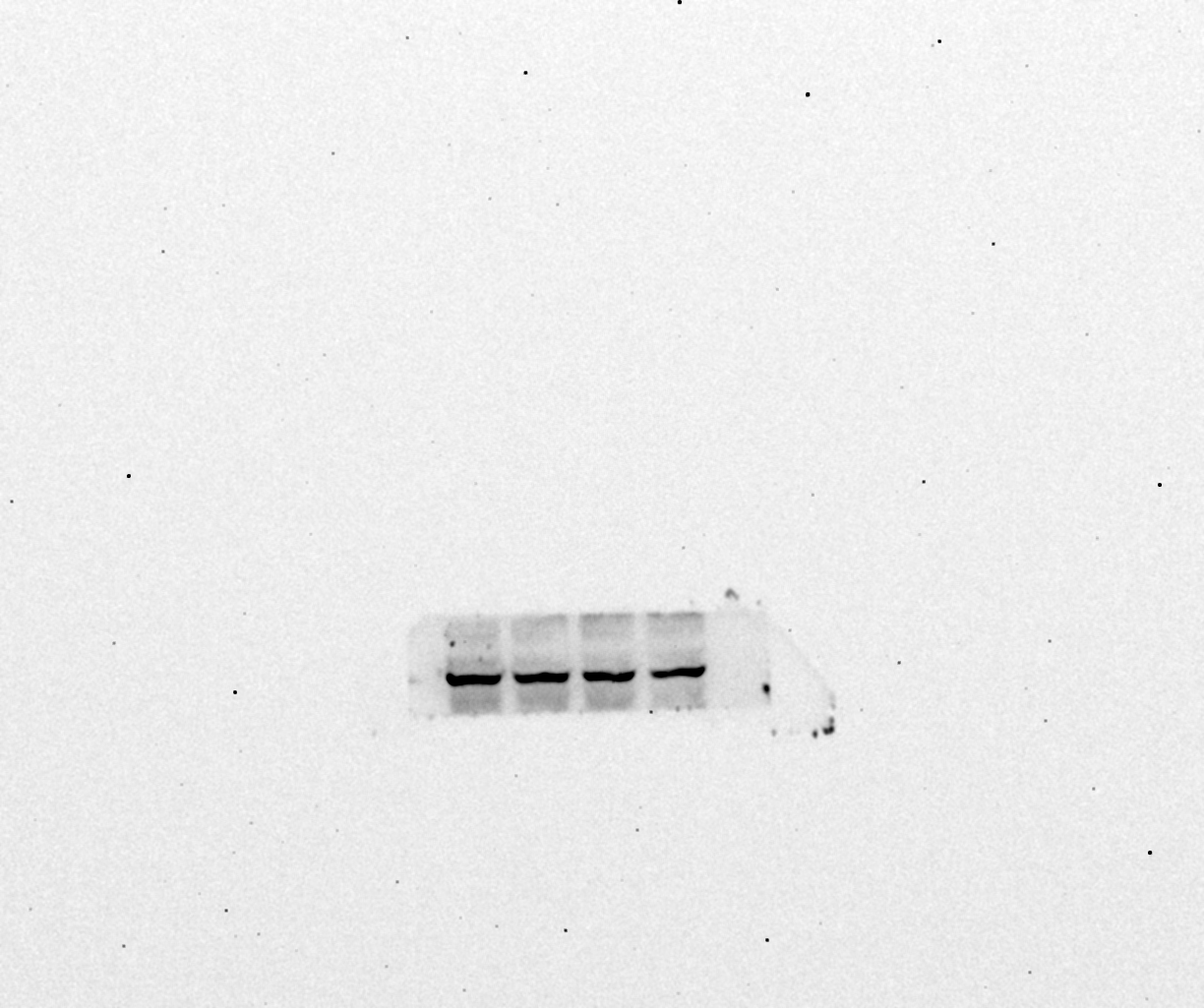

Supplement: Supplementary file 8 — Source data Fig. 5 [file 44319_2024_352_MOESM8_ESM.zip › Figure 5/5B/5B-WT/western TRIM71.tif]

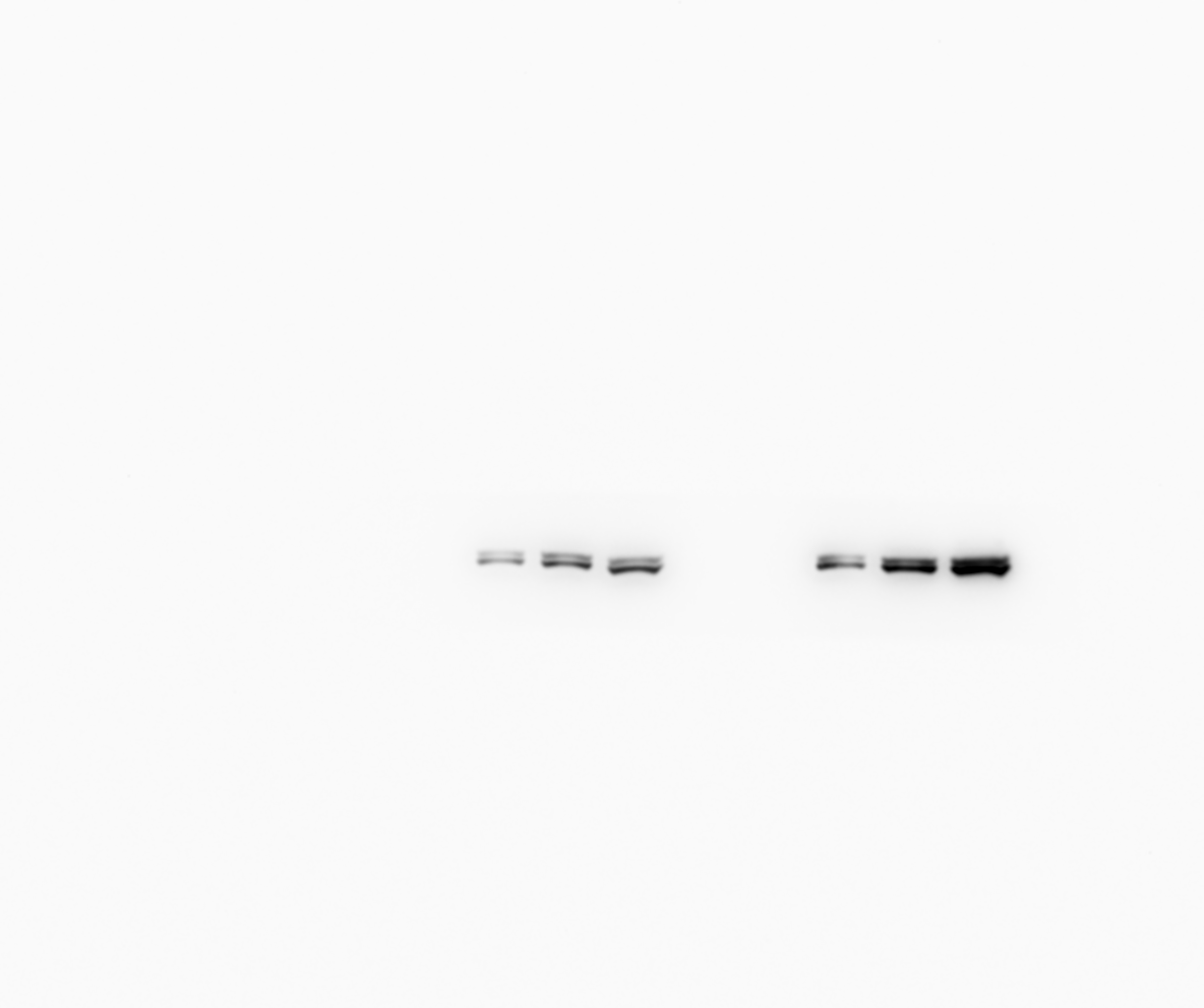

Supplement: Supplementary file 8 — Source data Fig. 5 [file 44319_2024_352_MOESM8_ESM.zip › Figure 5/5B/5B-WT/western myc.tif]

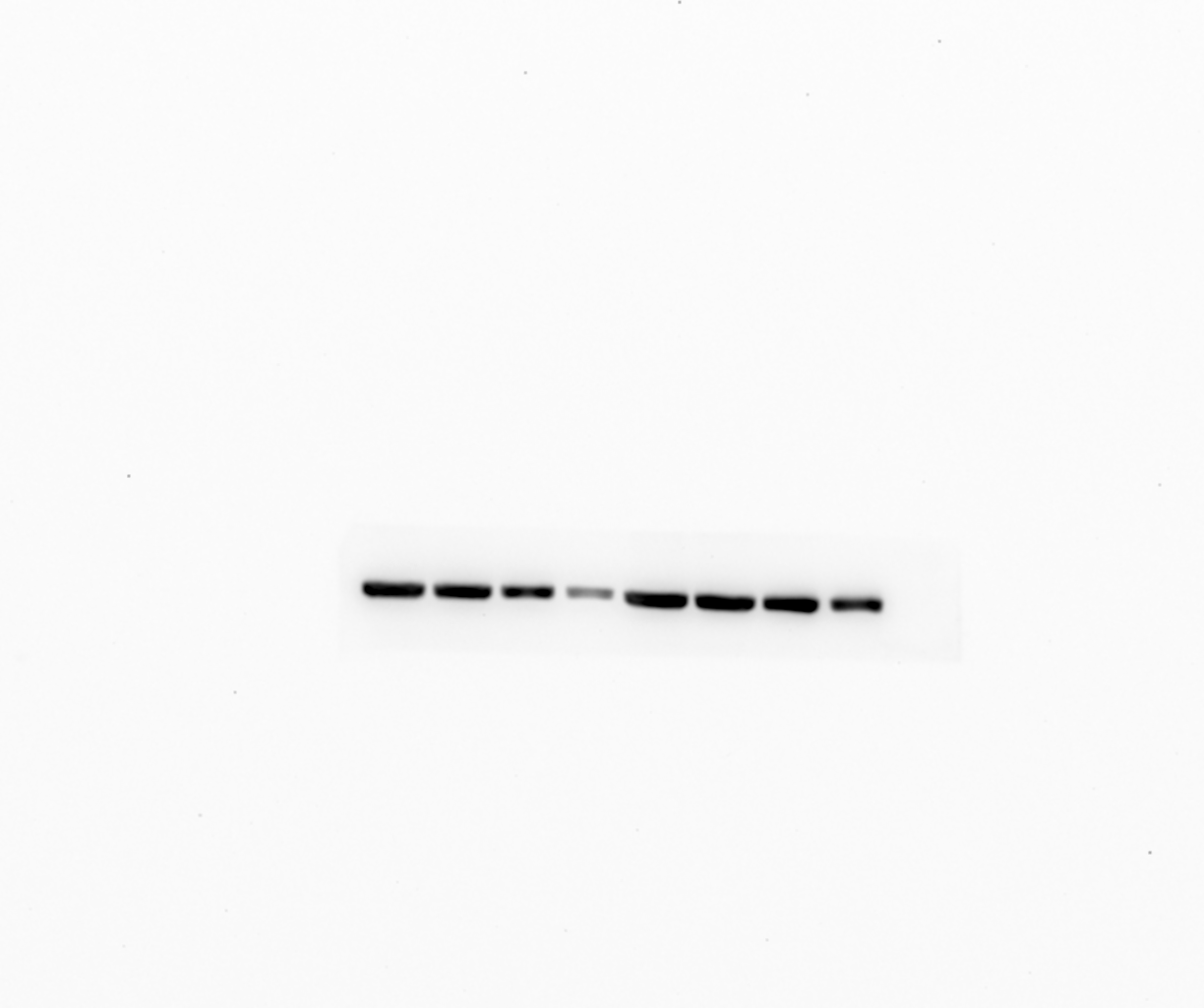

Supplement: Supplementary file 8 — Source data Fig. 5 [file 44319_2024_352_MOESM8_ESM.zip › Figure 5/5C/western Flag.tif]

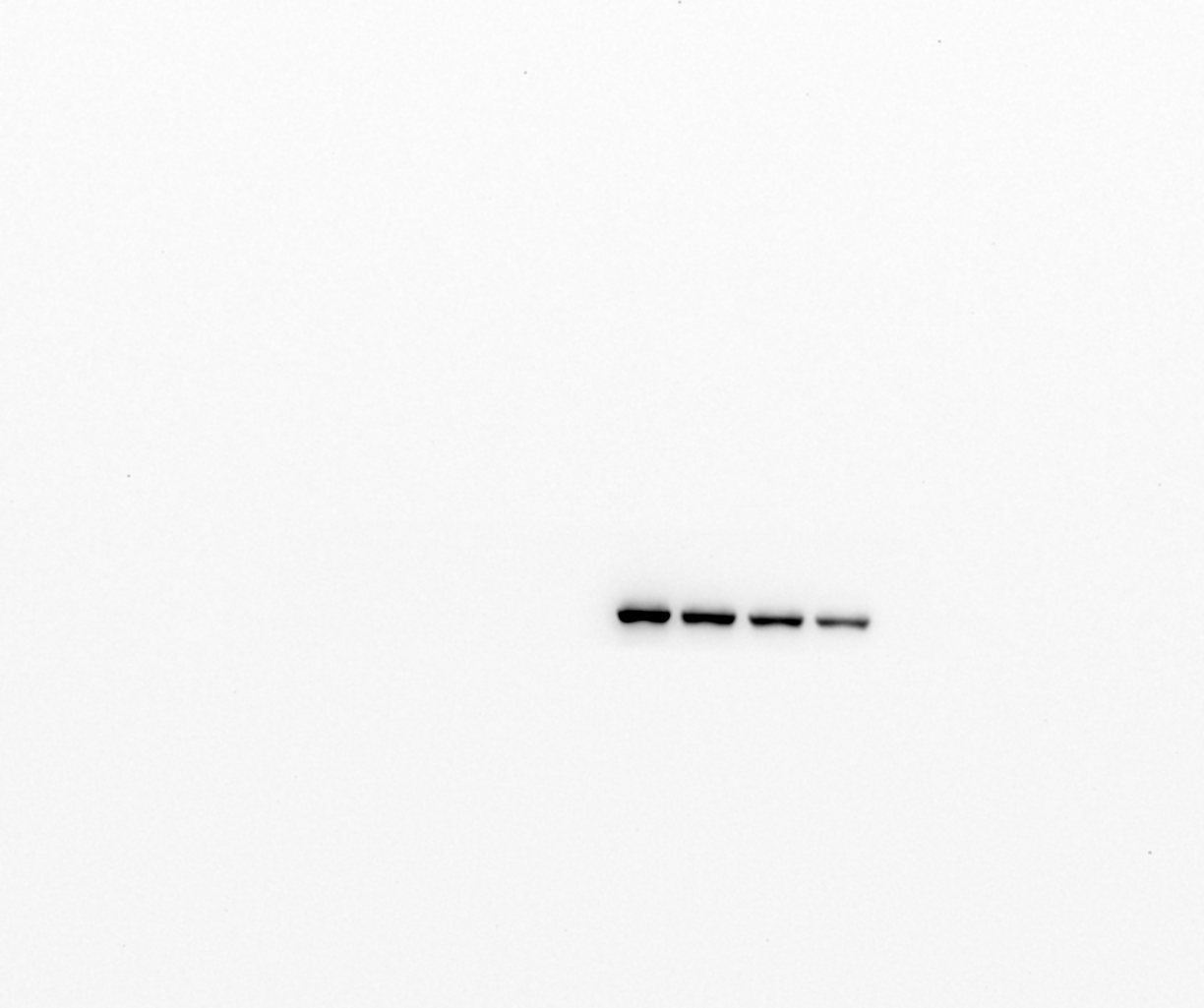

Supplement: Supplementary file 8 — Source data Fig. 5 [file 44319_2024_352_MOESM8_ESM.zip › Figure 5/5C/western GFP.tif]

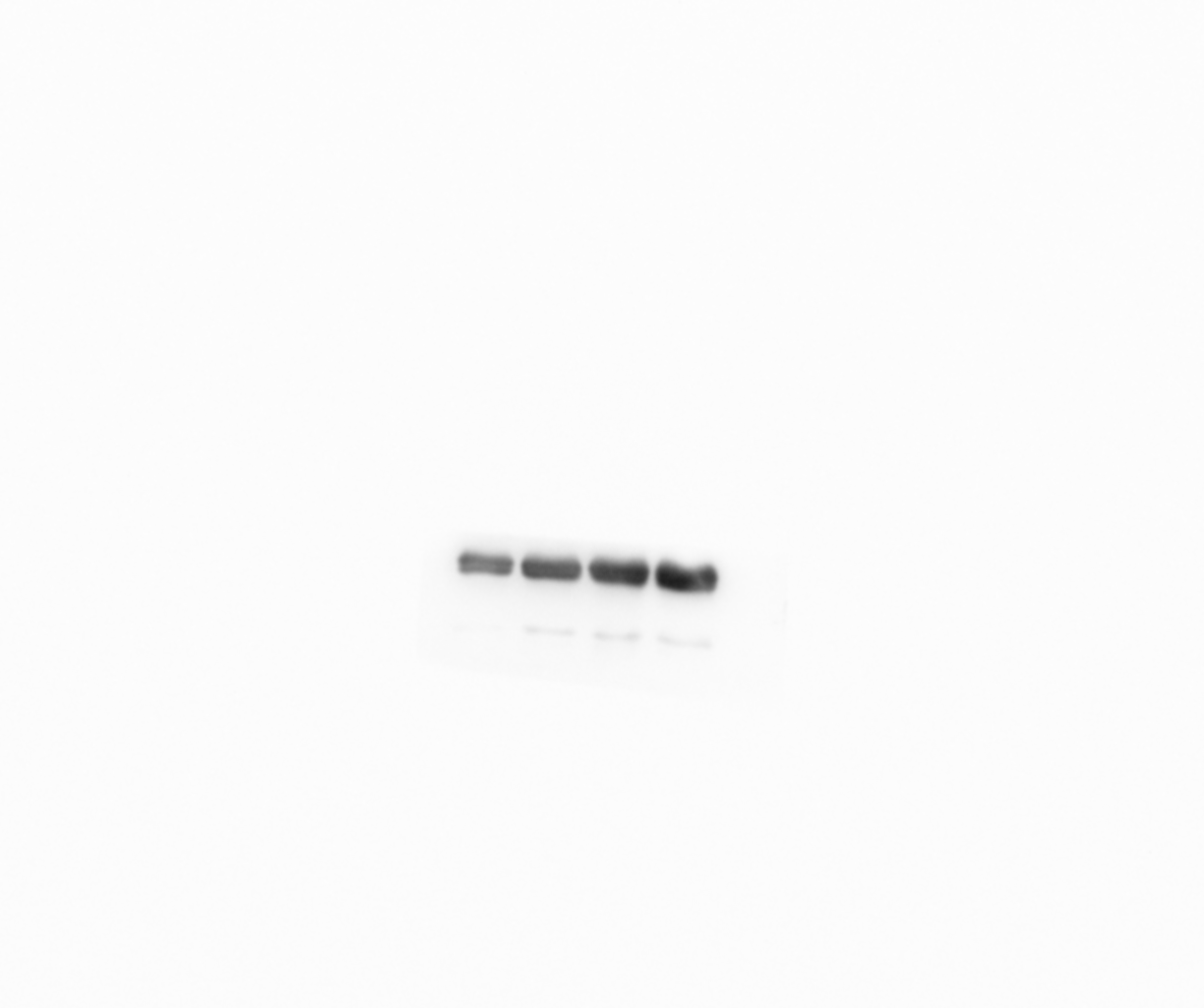

Supplement: Supplementary file 8 — Source data Fig. 5 [file 44319_2024_352_MOESM8_ESM.zip › Figure 5/5E/western Flag IP.tif]

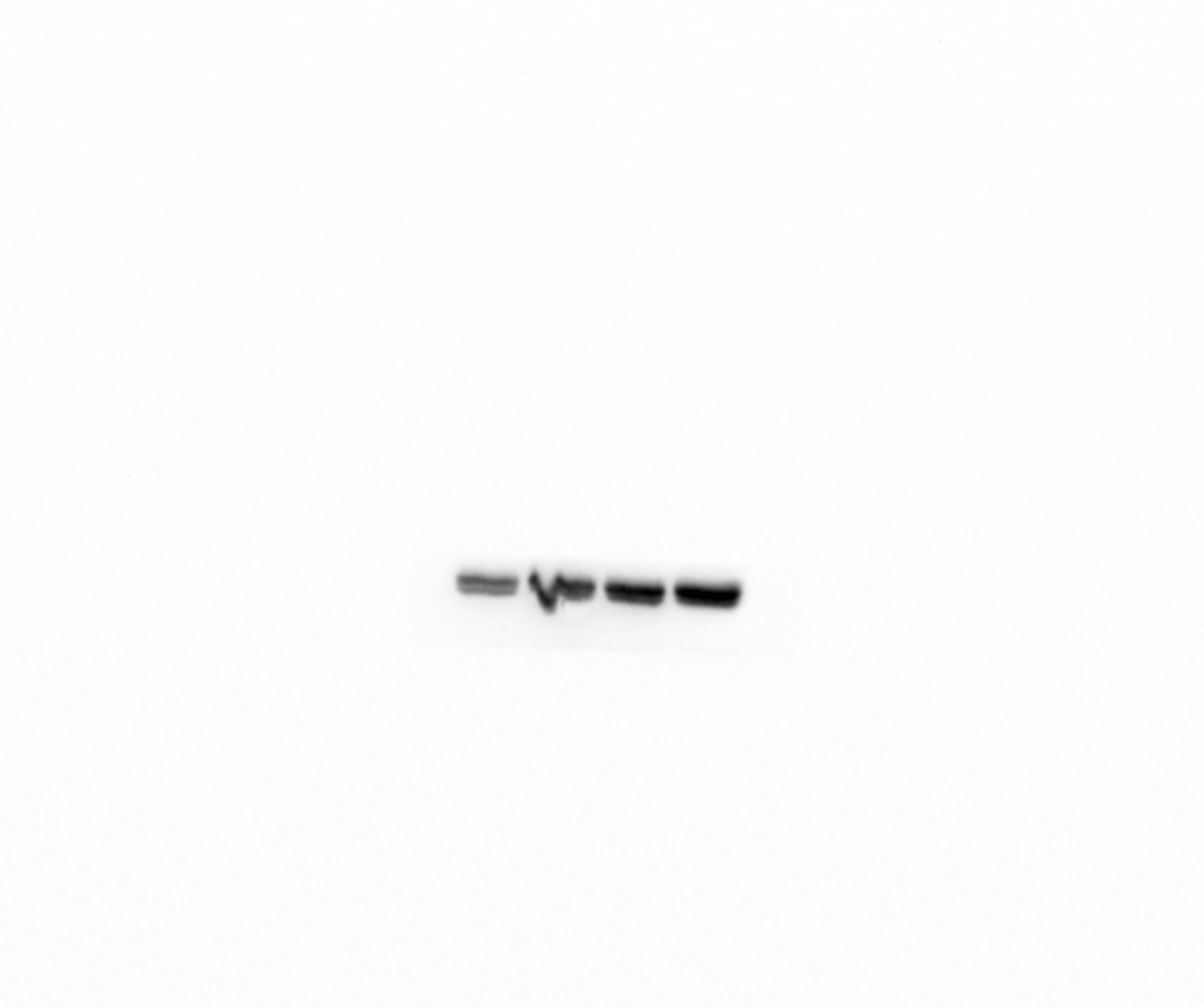

Supplement: Supplementary file 8 — Source data Fig. 5 [file 44319_2024_352_MOESM8_ESM.zip › Figure 5/5E/western Flag Input.tif]

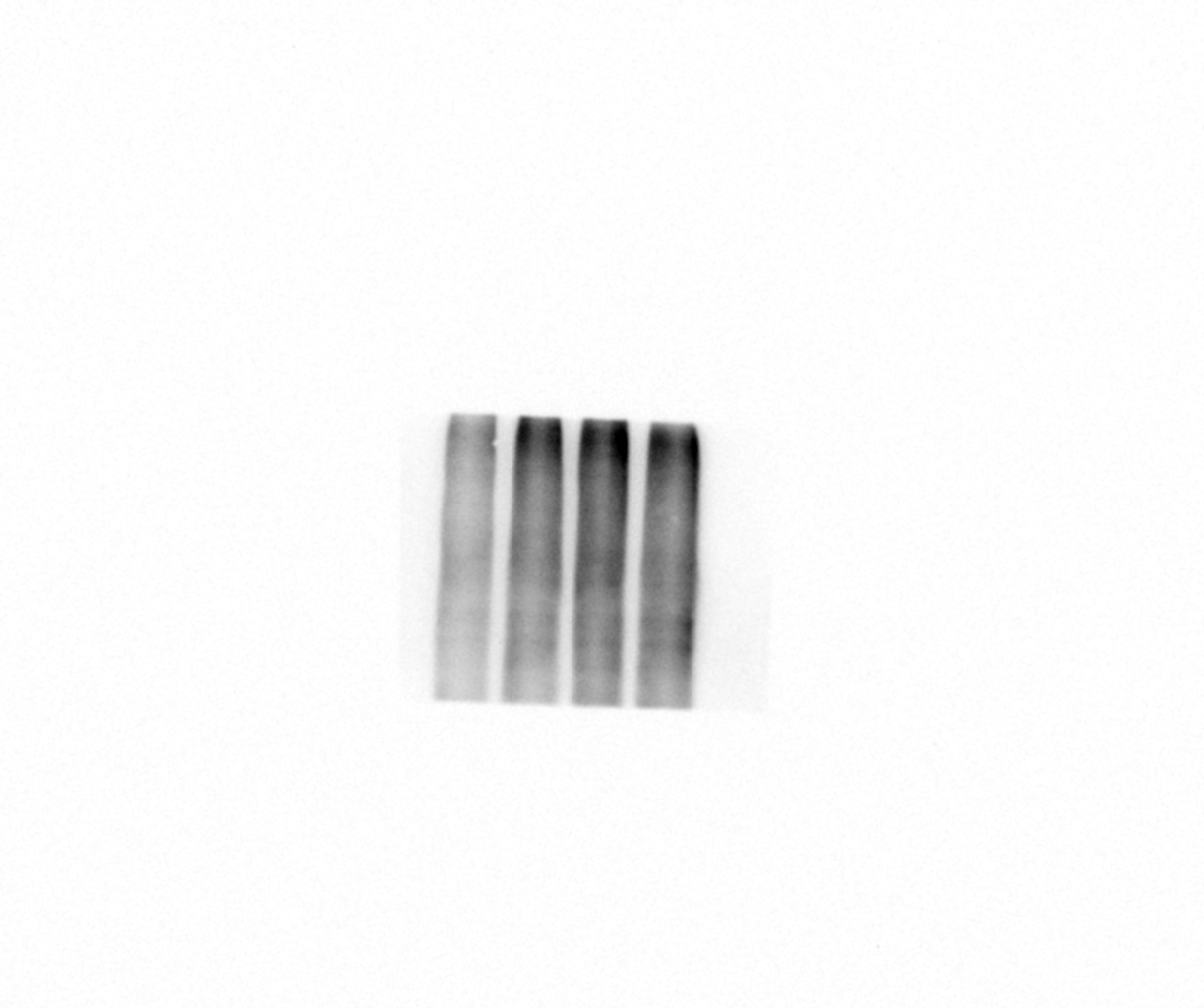

Supplement: Supplementary file 8 — Source data Fig. 5 [file 44319_2024_352_MOESM8_ESM.zip › Figure 5/5E/western HA Input.tif]

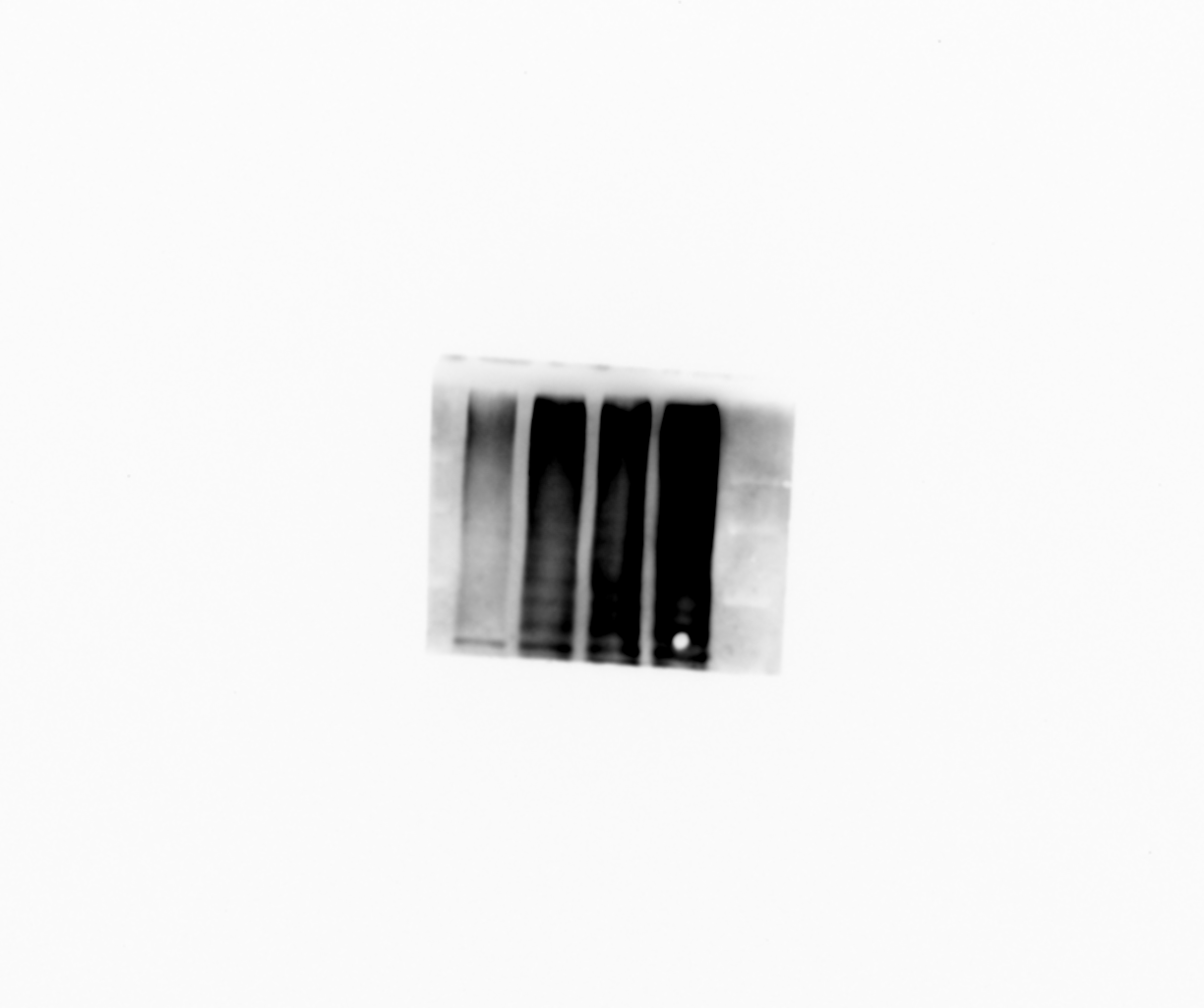

Supplement: Supplementary file 8 — Source data Fig. 5 [file 44319_2024_352_MOESM8_ESM.zip › Figure 5/5E/western HA IP.tif]

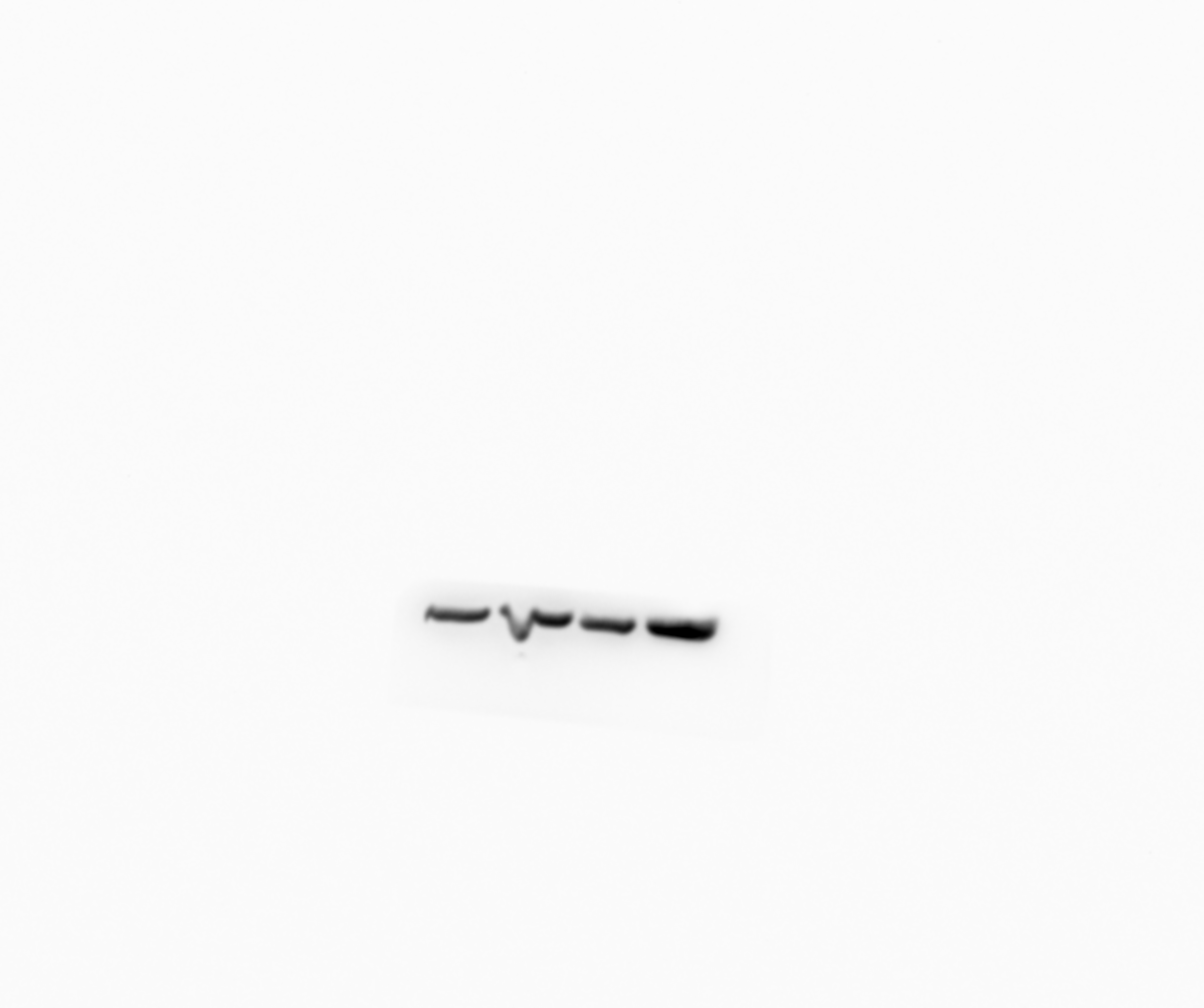

Supplement: Supplementary file 8 — Source data Fig. 5 [file 44319_2024_352_MOESM8_ESM.zip › Figure 5/5E/western actin.tif]

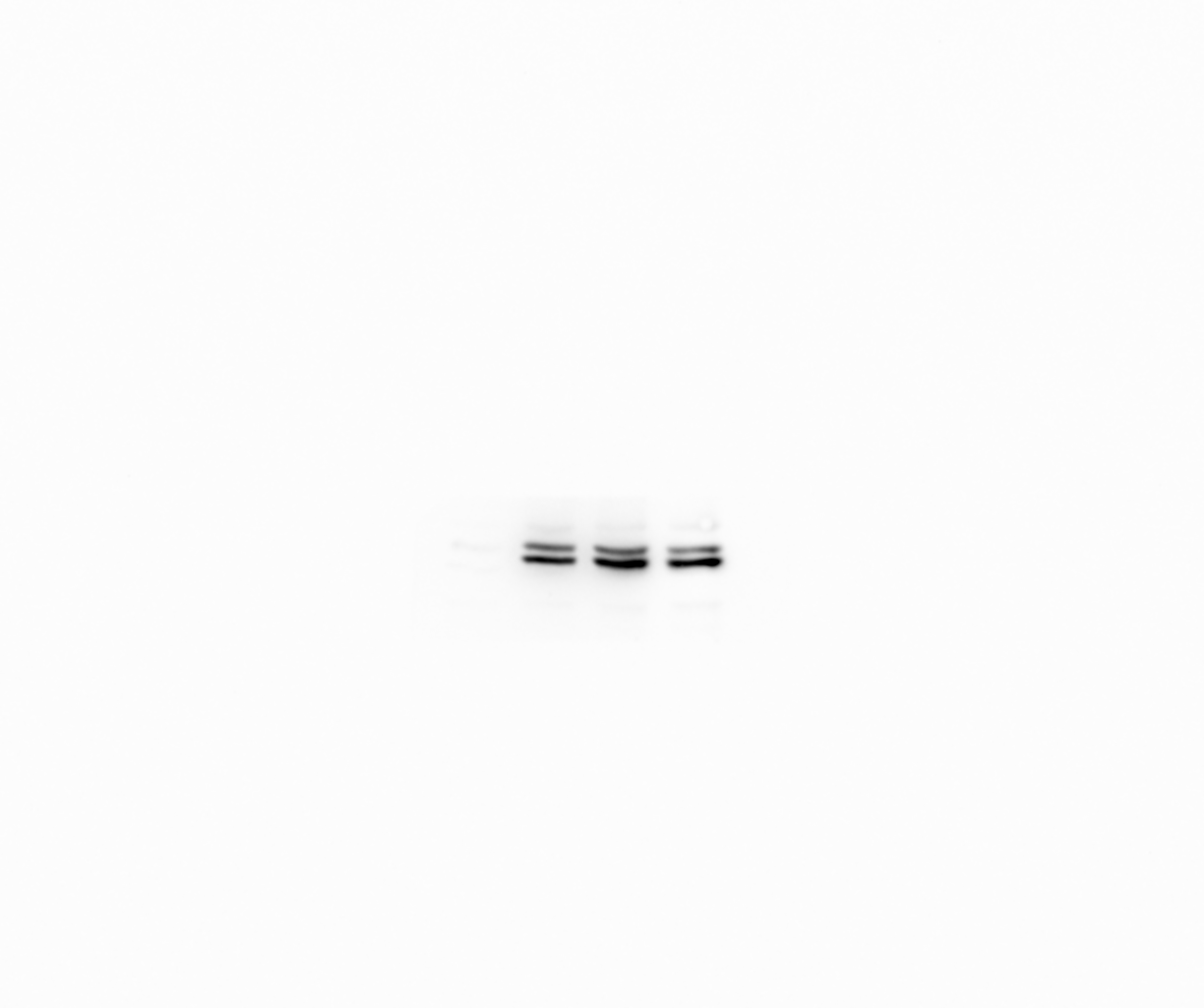

Supplement: Supplementary file 8 — Source data Fig. 5 [file 44319_2024_352_MOESM8_ESM.zip › Figure 5/5E/western myc Input.tif]

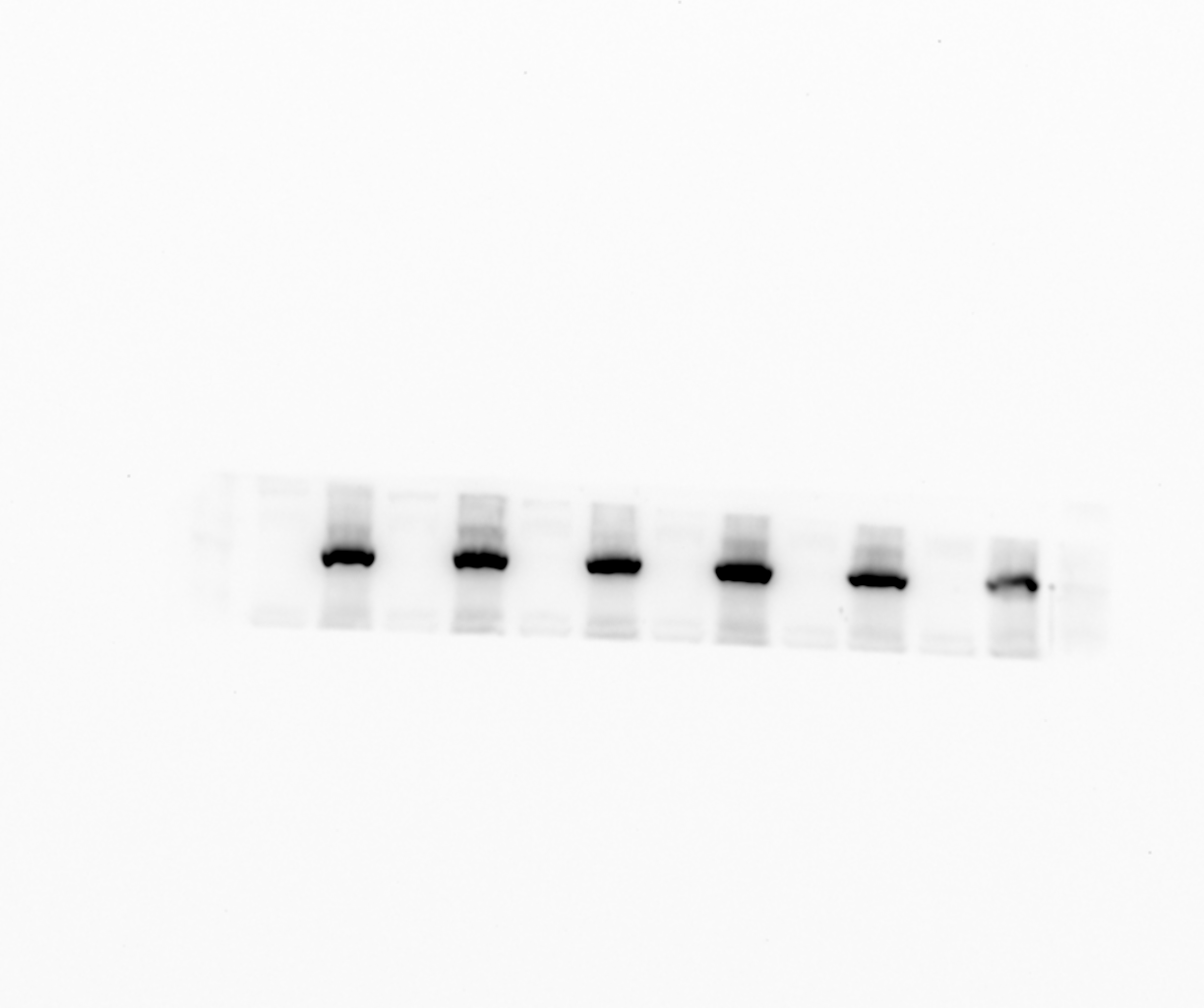

Supplement: Supplementary file 8 — Source data Fig. 5 [file 44319_2024_352_MOESM8_ESM.zip › Figure 5/5F/western DYRK4.tif]

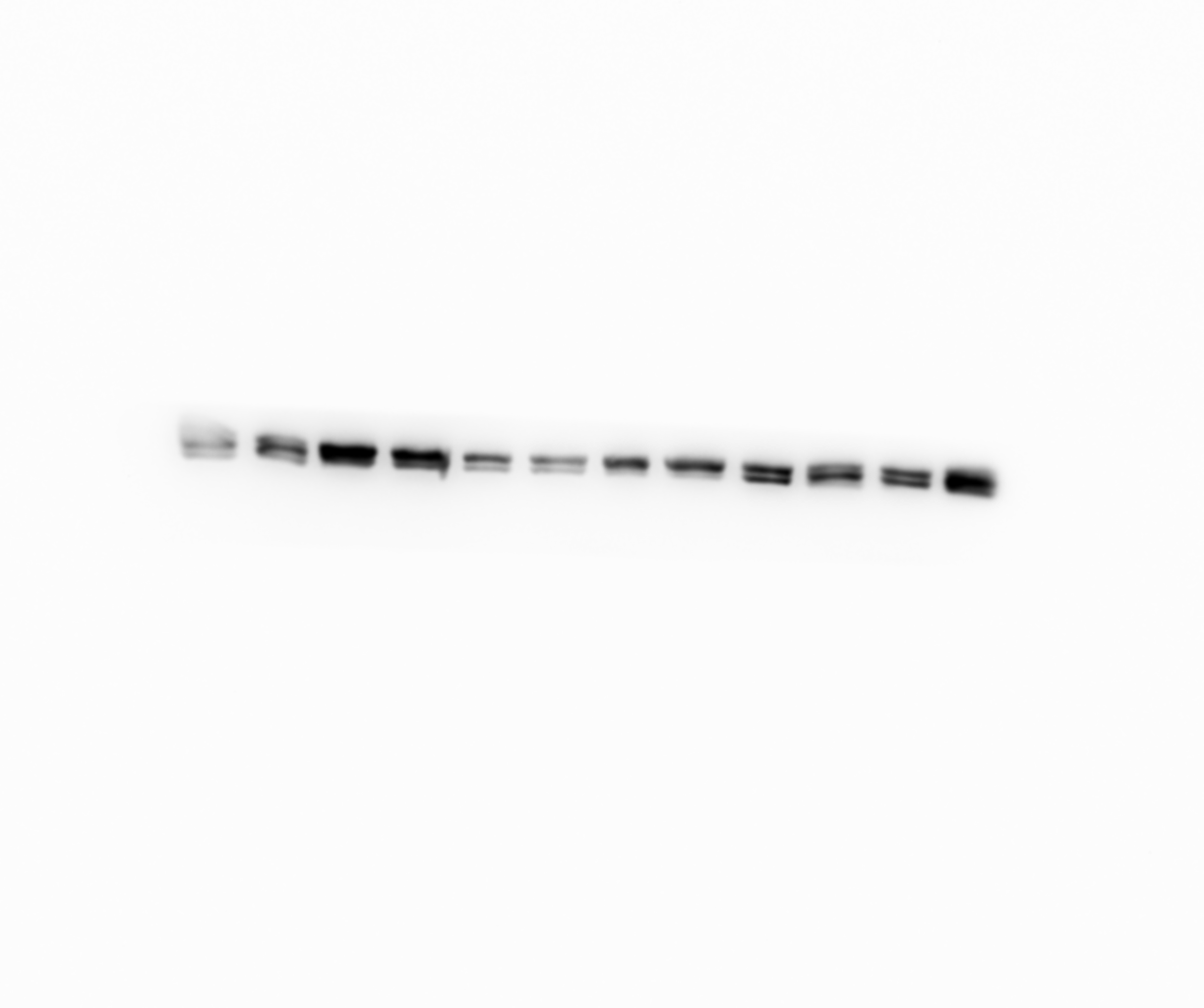

Supplement: Supplementary file 8 — Source data Fig. 5 [file 44319_2024_352_MOESM8_ESM.zip › Figure 5/5F/western Flag IP.tif]

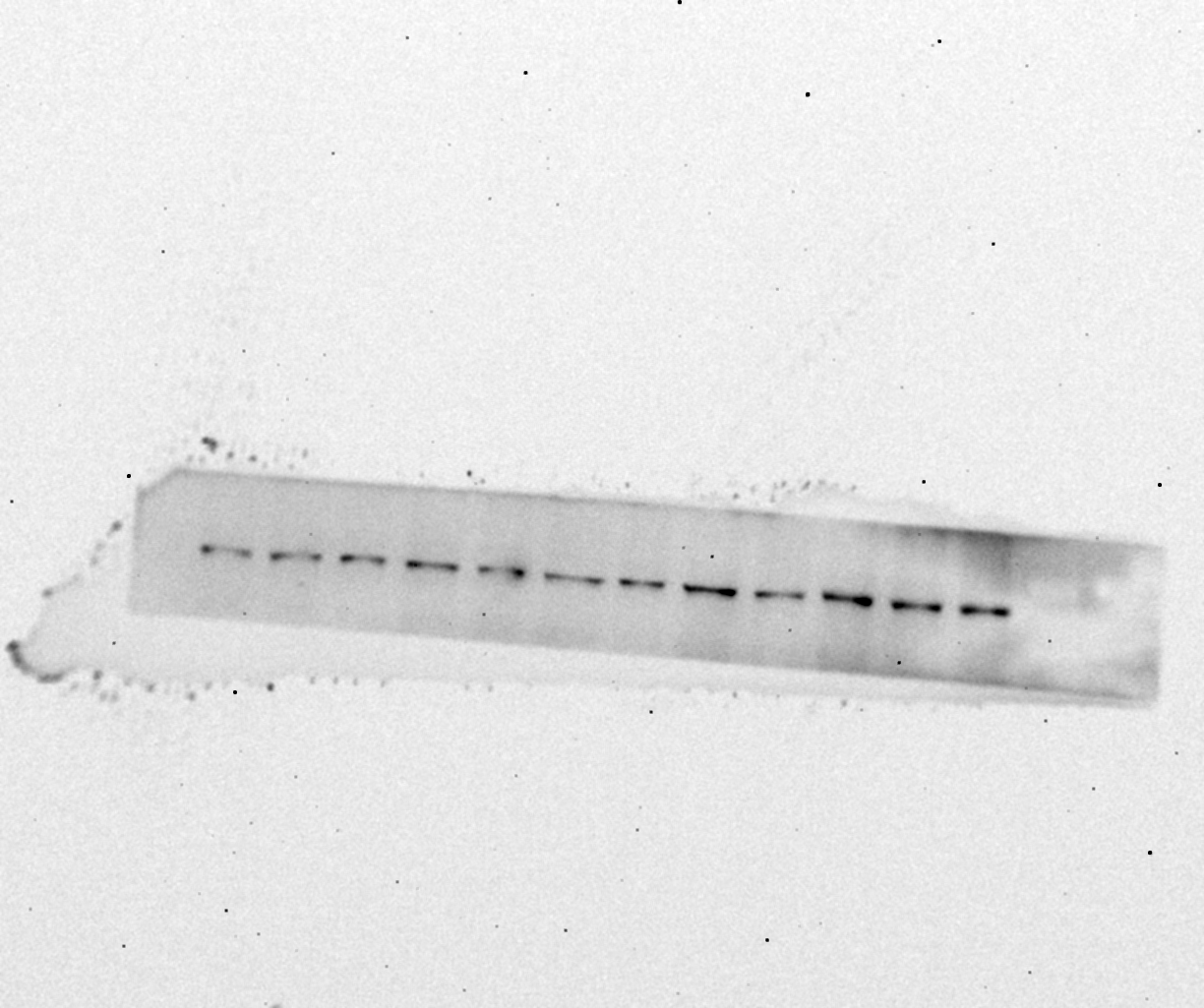

Supplement: Supplementary file 8 — Source data Fig. 5 [file 44319_2024_352_MOESM8_ESM.zip › Figure 5/5F/western Flag Input.tif]

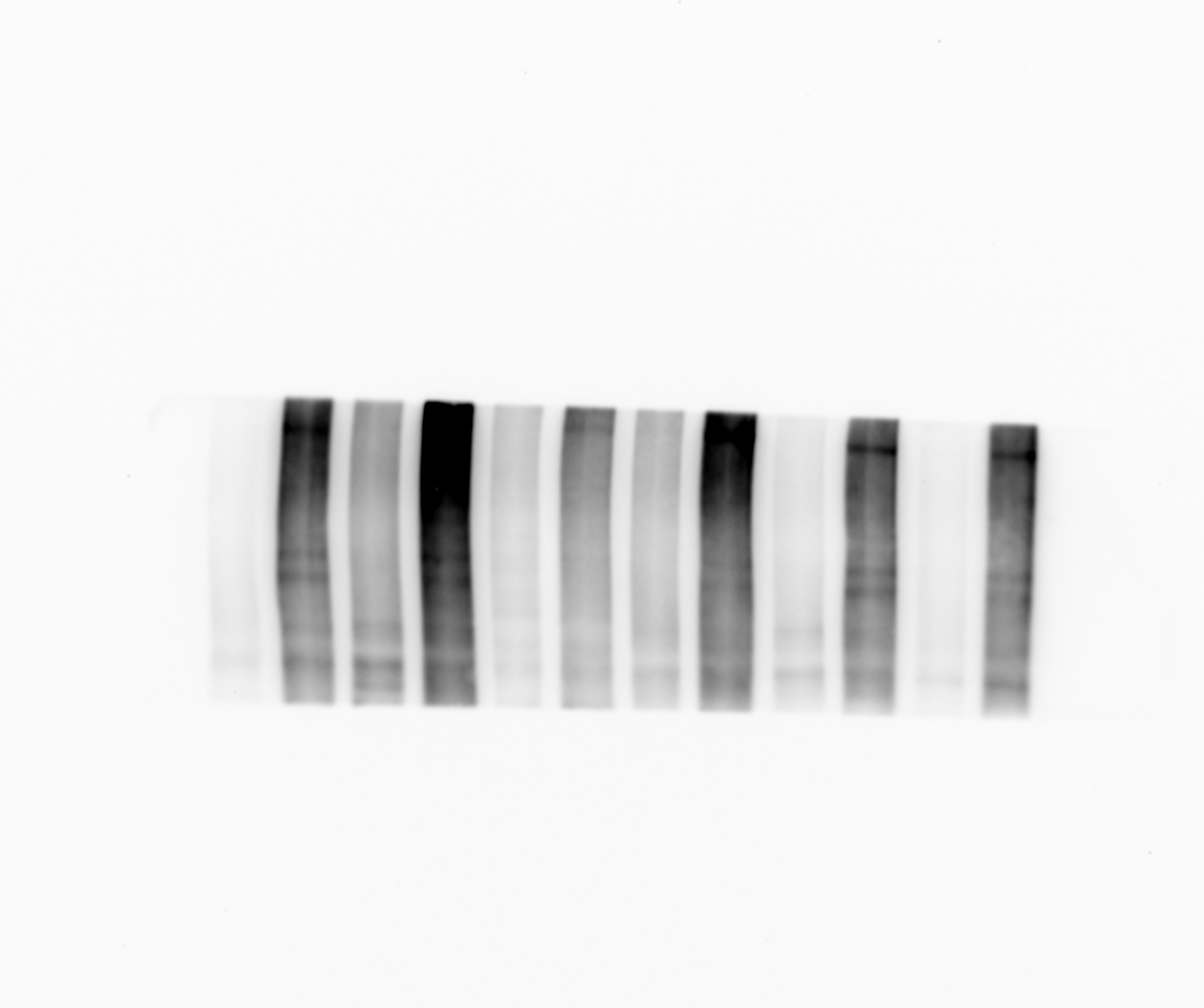

Supplement: Supplementary file 8 — Source data Fig. 5 [file 44319_2024_352_MOESM8_ESM.zip › Figure 5/5F/western HA IP.tif]

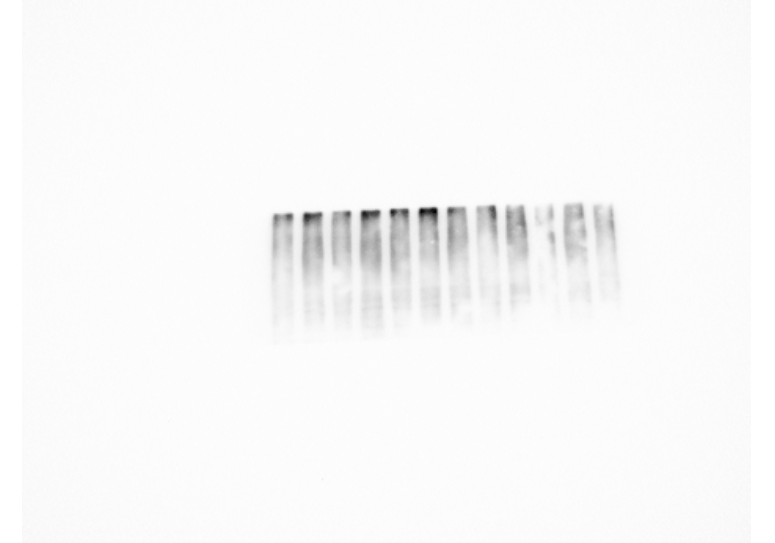

Supplement: Supplementary file 8 — Source data Fig. 5 [file 44319_2024_352_MOESM8_ESM.zip › Figure 5/5F/western HA Input.tif]

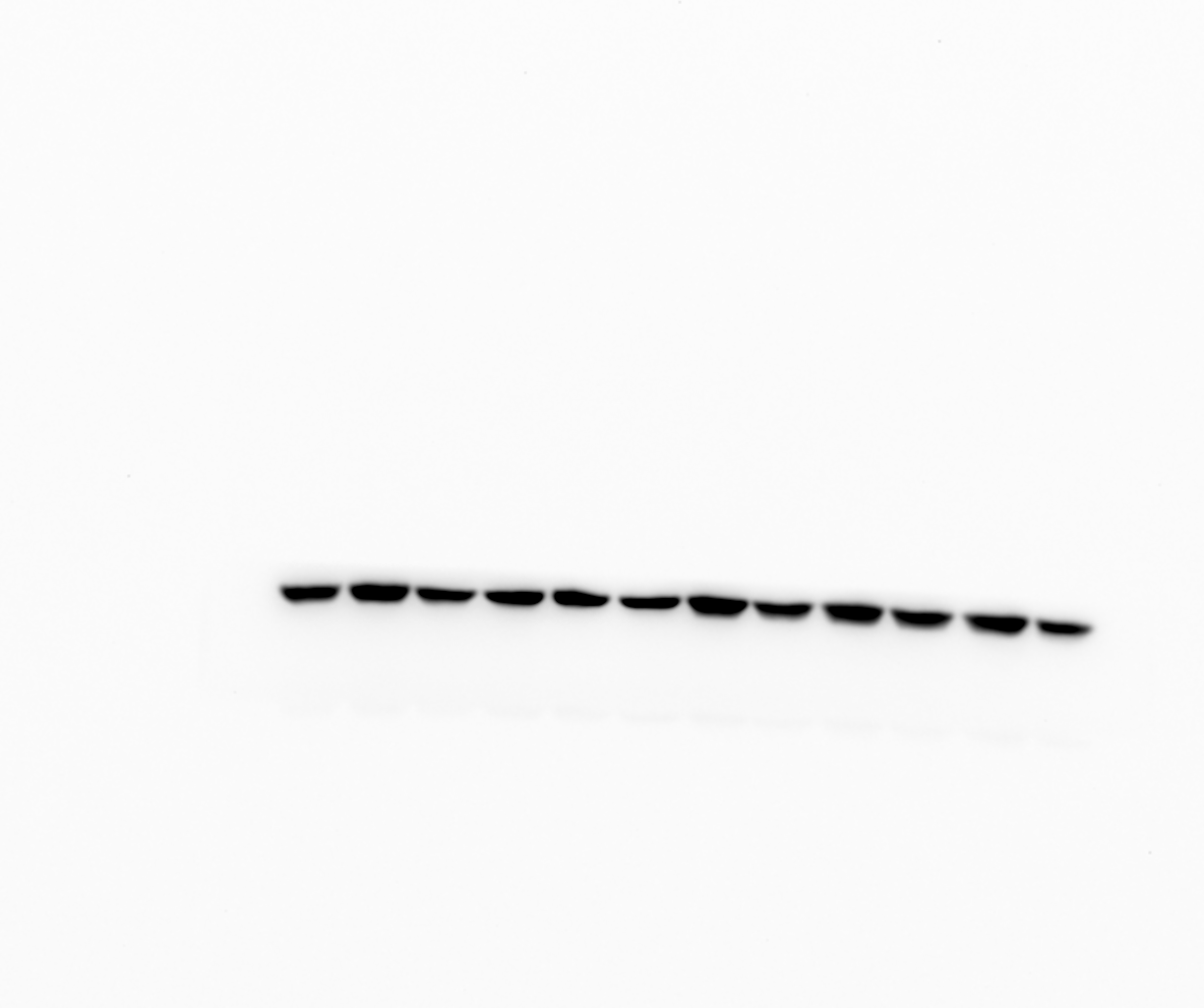

Supplement: Supplementary file 8 — Source data Fig. 5 [file 44319_2024_352_MOESM8_ESM.zip › Figure 5/5F/western actin.tif]

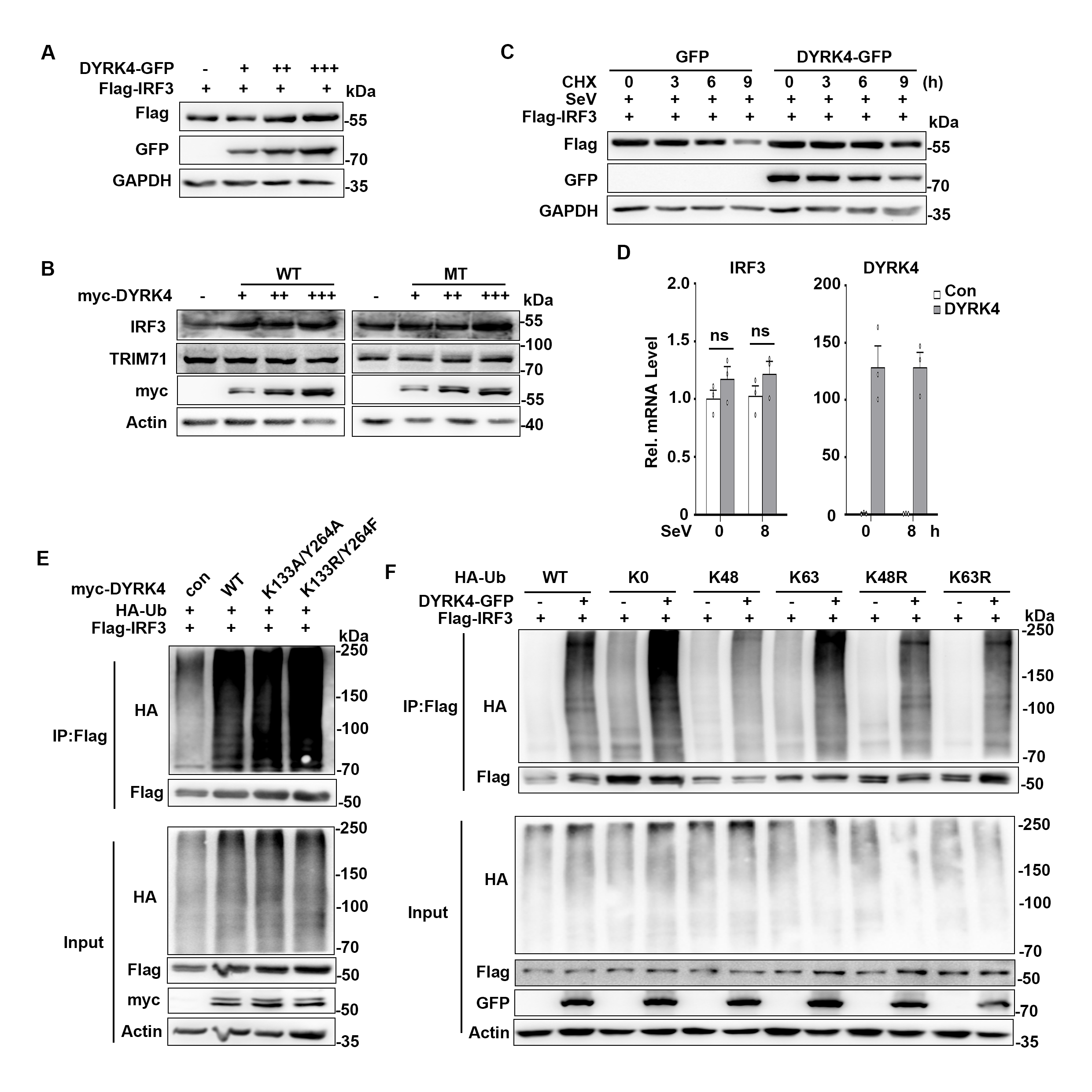

Supplement: Supplementary file 8 — Source data Fig. 5 [file 44319_2024_352_MOESM8_ESM.zip › Figure 5/Figure 5.tif]

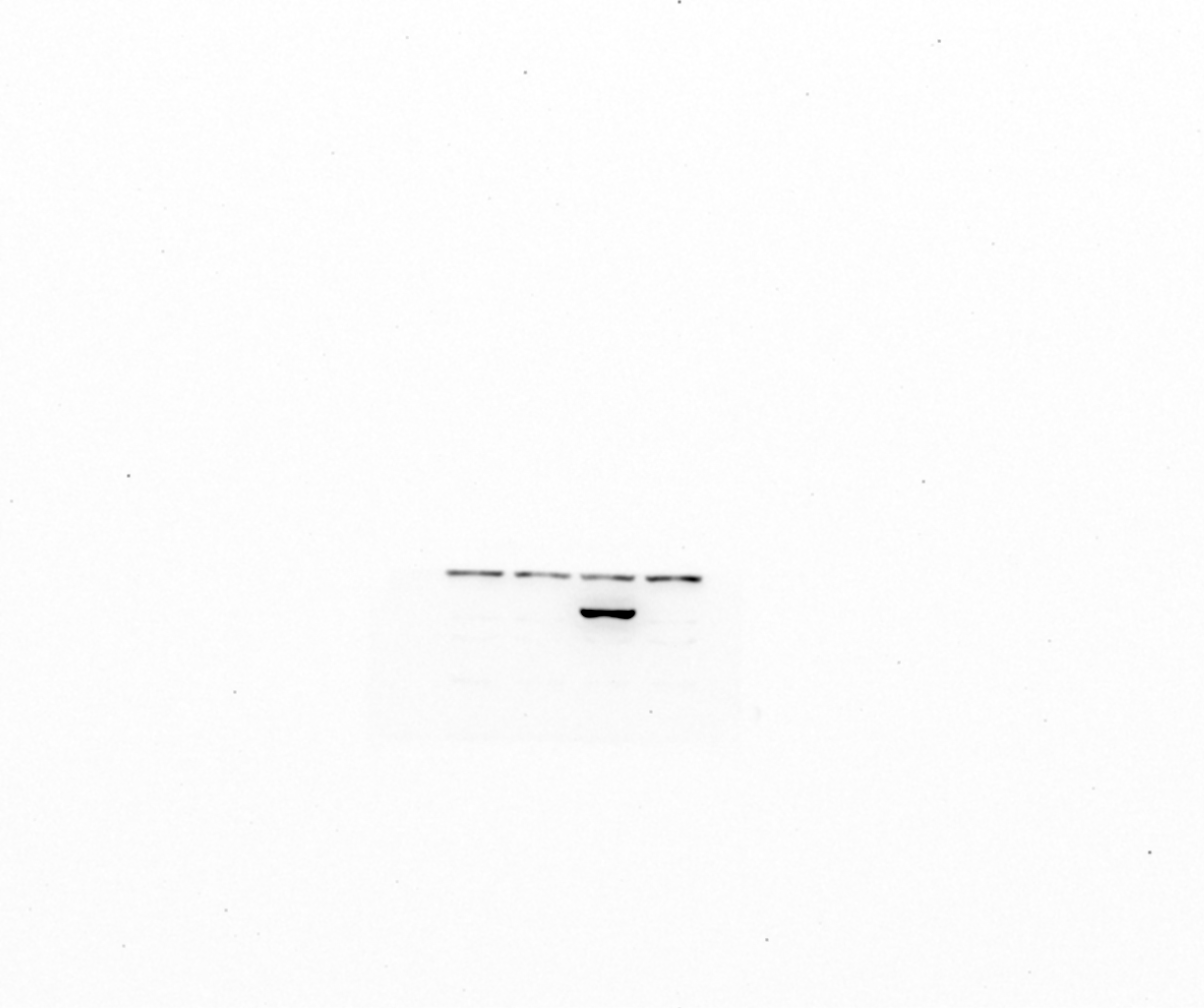

Supplement: Supplementary file 9 — Source data Fig. 6 [file 44319_2024_352_MOESM9_ESM.zip › Figure 6/6C/western Flag TRIM71 Input.tif]

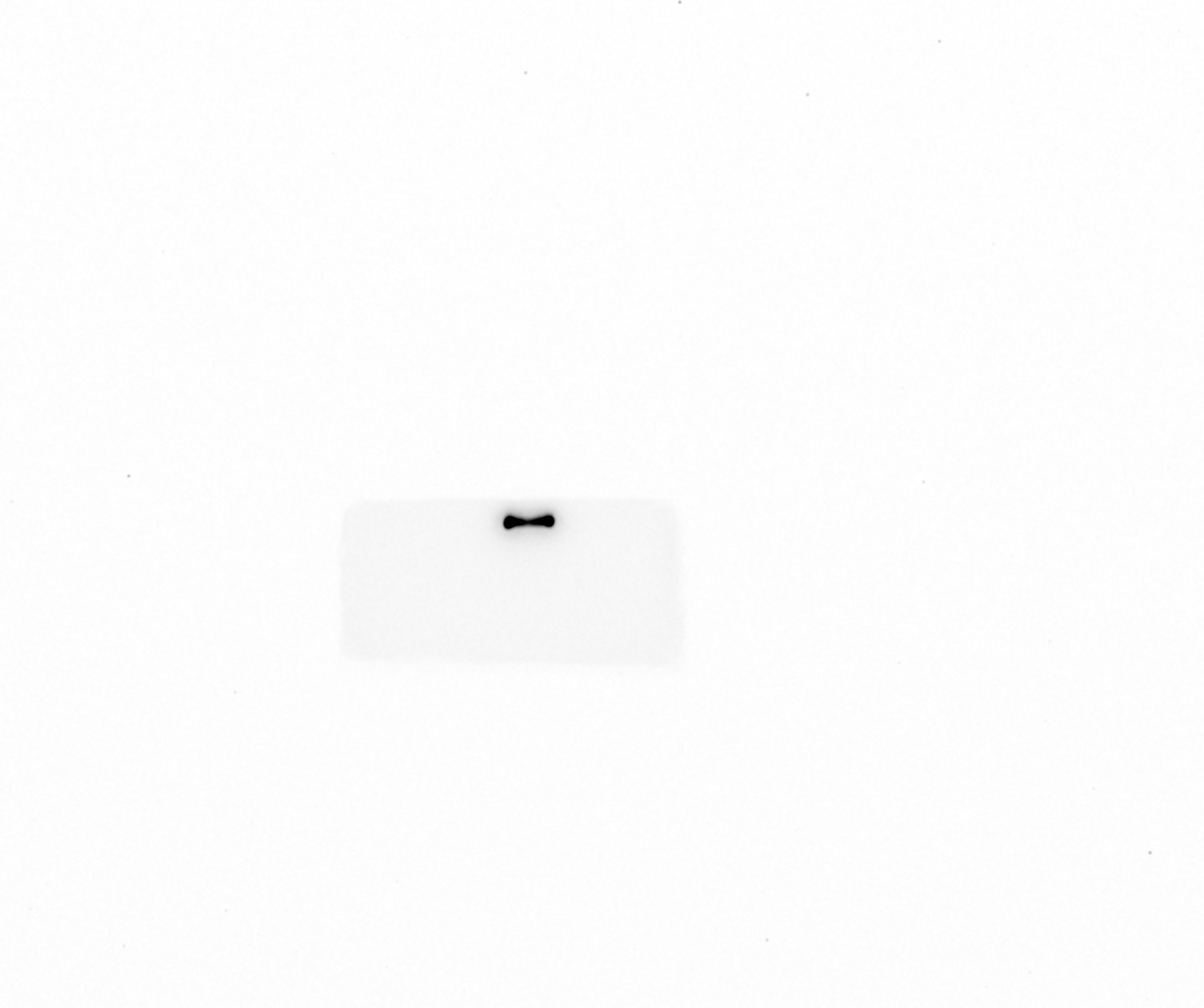

Supplement: Supplementary file 9 — Source data Fig. 6 [file 44319_2024_352_MOESM9_ESM.zip › Figure 6/6C/western Flag-TRIM71 IP.tif]

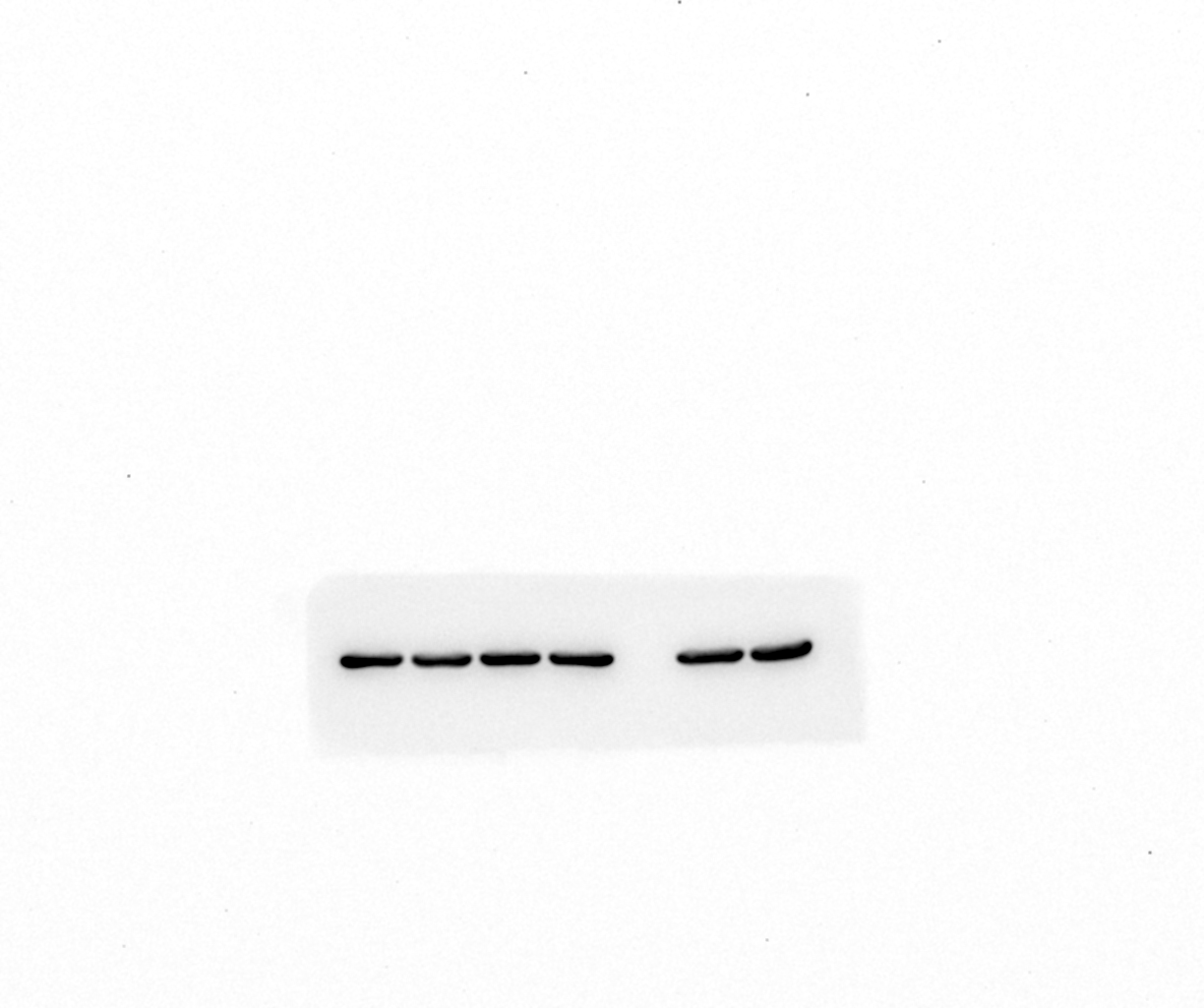

Supplement: Supplementary file 9 — Source data Fig. 6 [file 44319_2024_352_MOESM9_ESM.zip › Figure 6/6C/western GAPDH.tif]

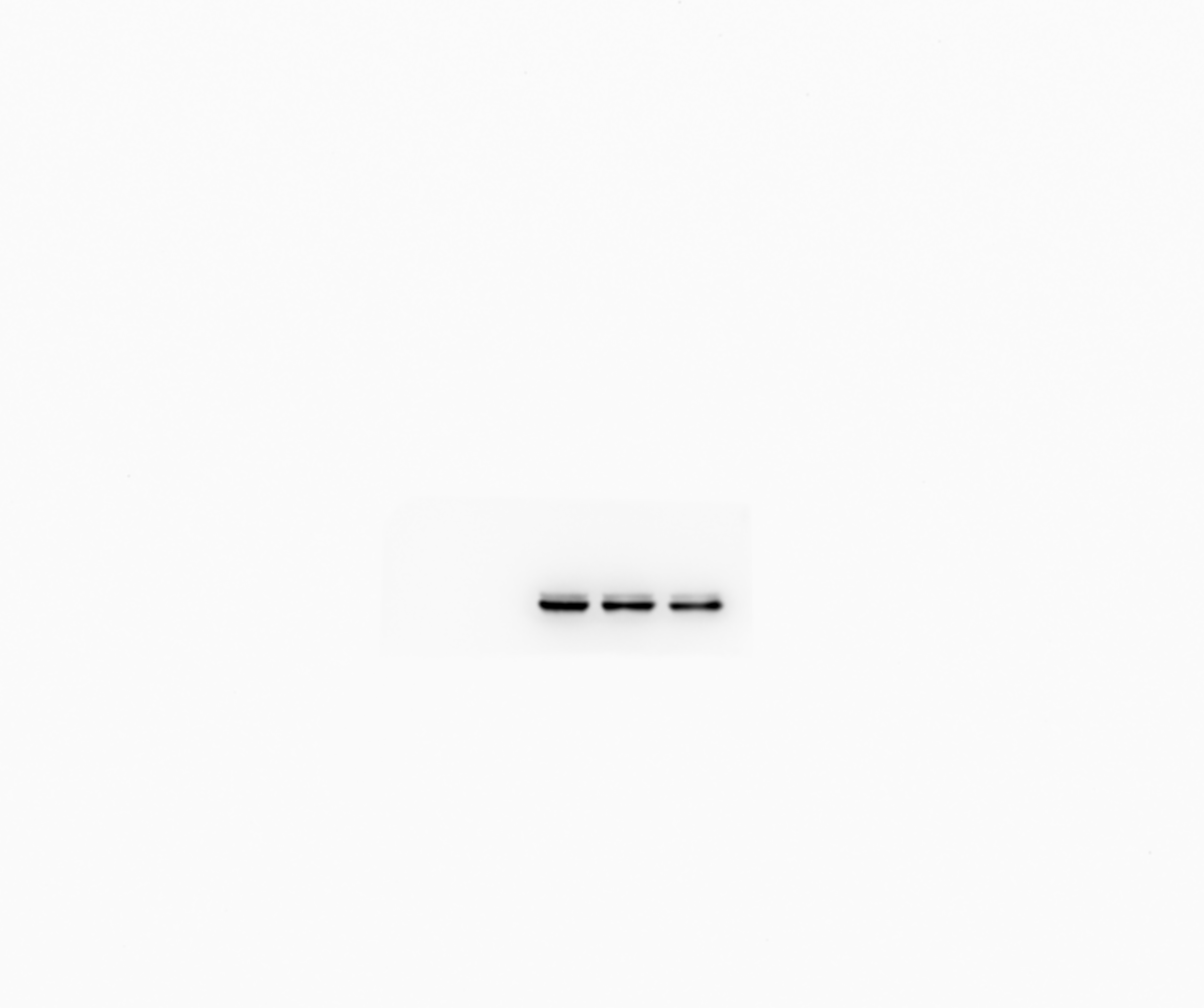

Supplement: Supplementary file 9 — Source data Fig. 6 [file 44319_2024_352_MOESM9_ESM.zip › Figure 6/6C/western myc Input.tif]

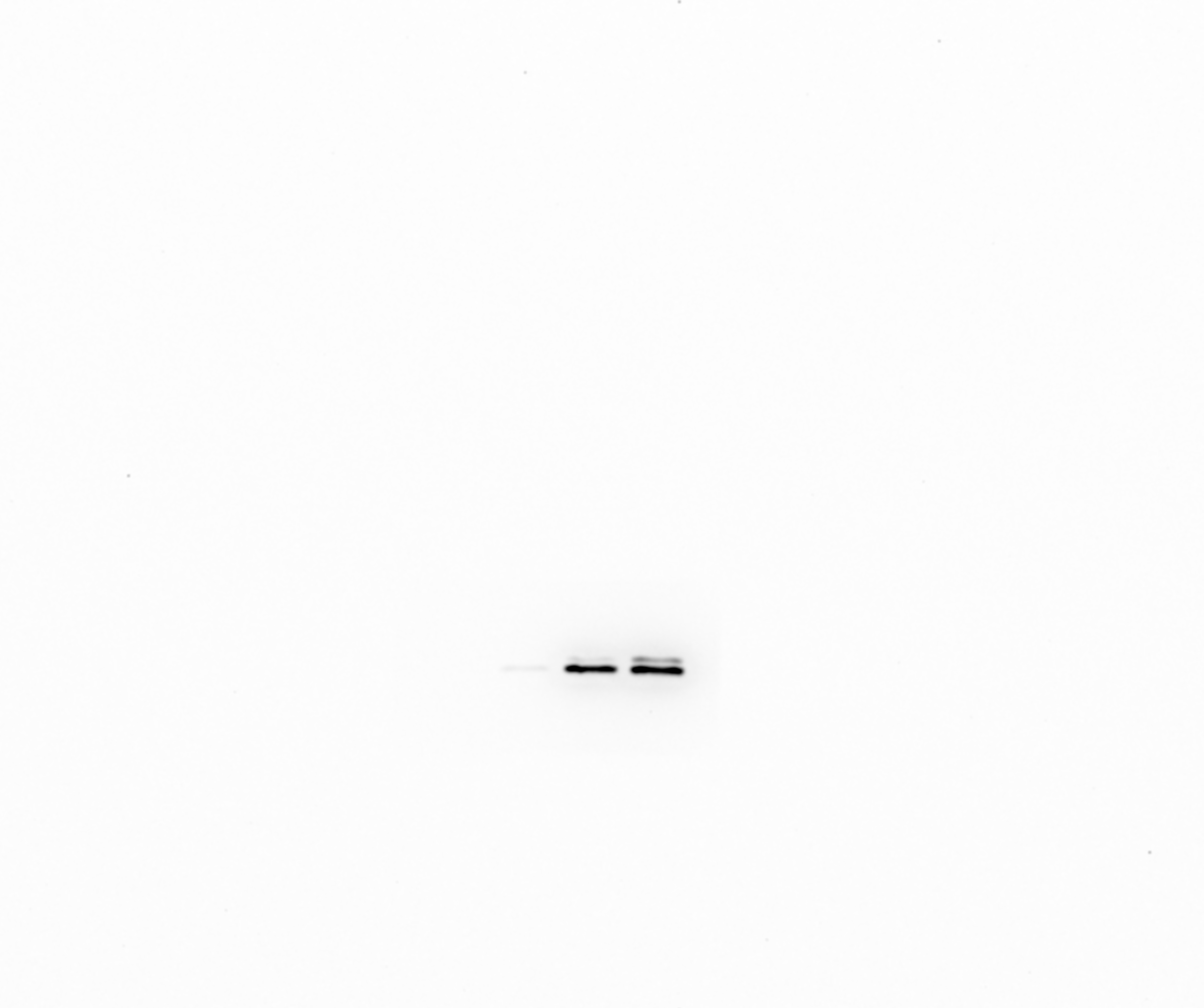

Supplement: Supplementary file 9 — Source data Fig. 6 [file 44319_2024_352_MOESM9_ESM.zip › Figure 6/6C/western myc IP.tif]

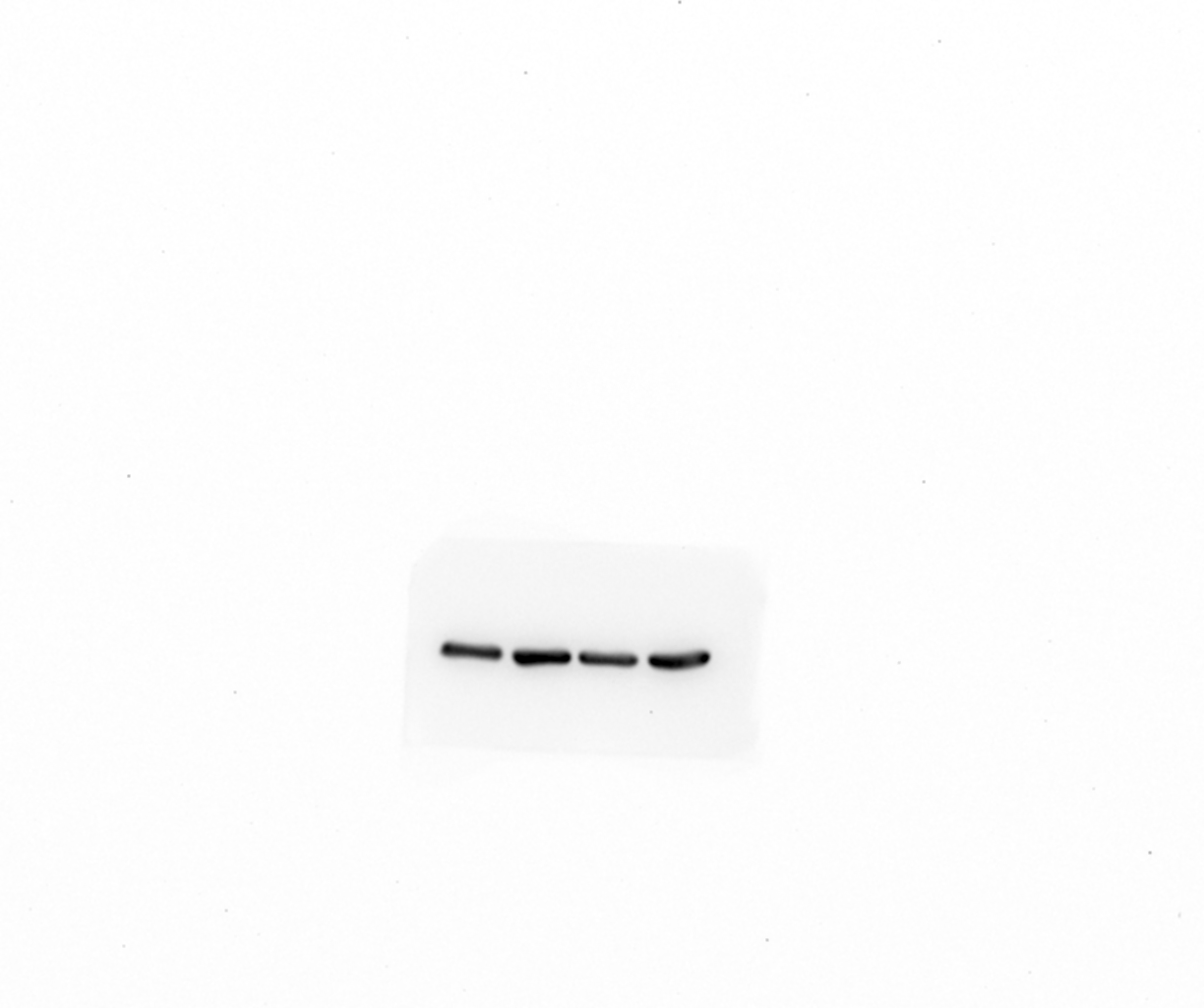

Supplement: Supplementary file 9 — Source data Fig. 6 [file 44319_2024_352_MOESM9_ESM.zip › Figure 6/6D/western GAPDH.tif]

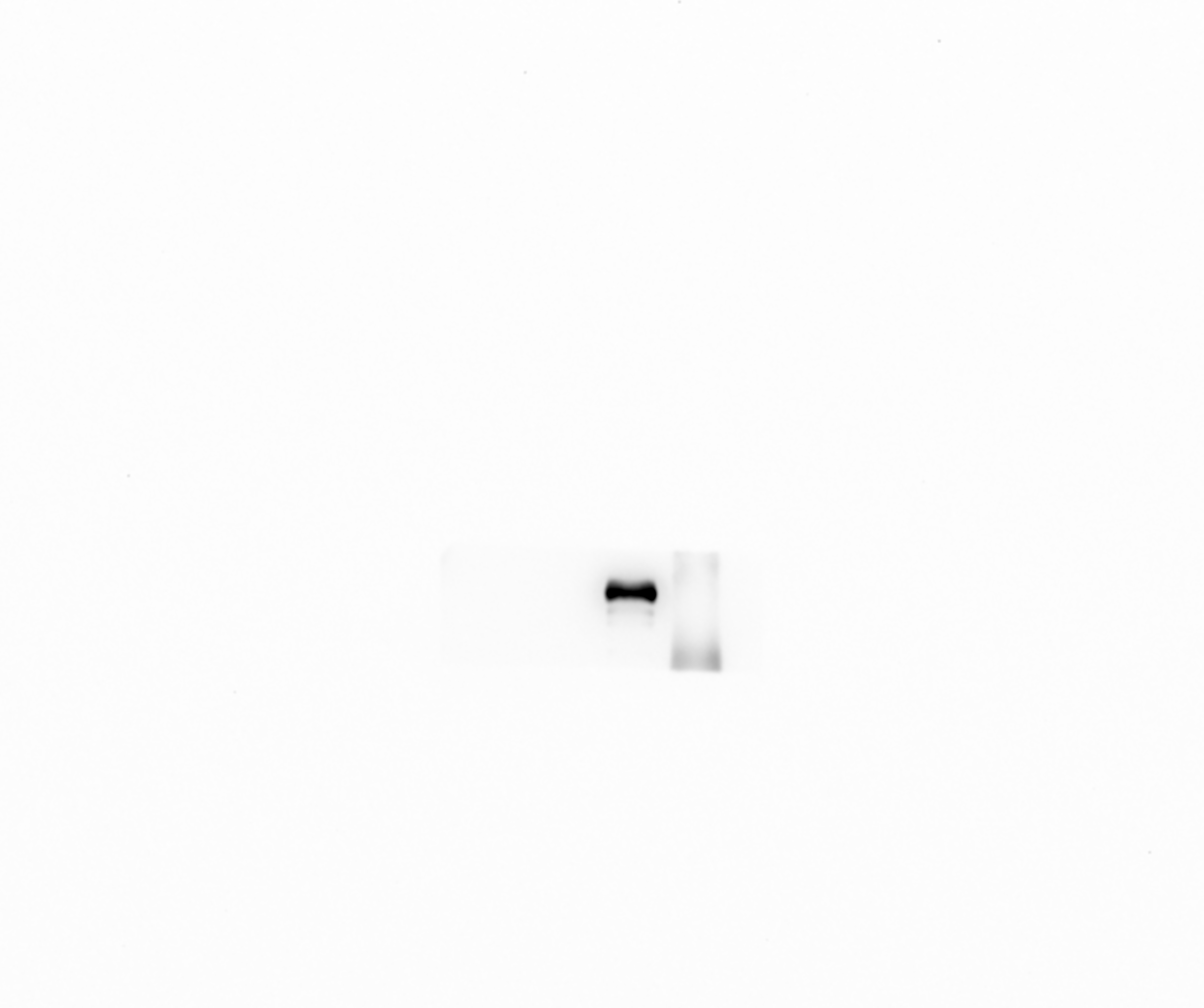

Supplement: Supplementary file 9 — Source data Fig. 6 [file 44319_2024_352_MOESM9_ESM.zip › Figure 6/6D/western Flag IP.tif]

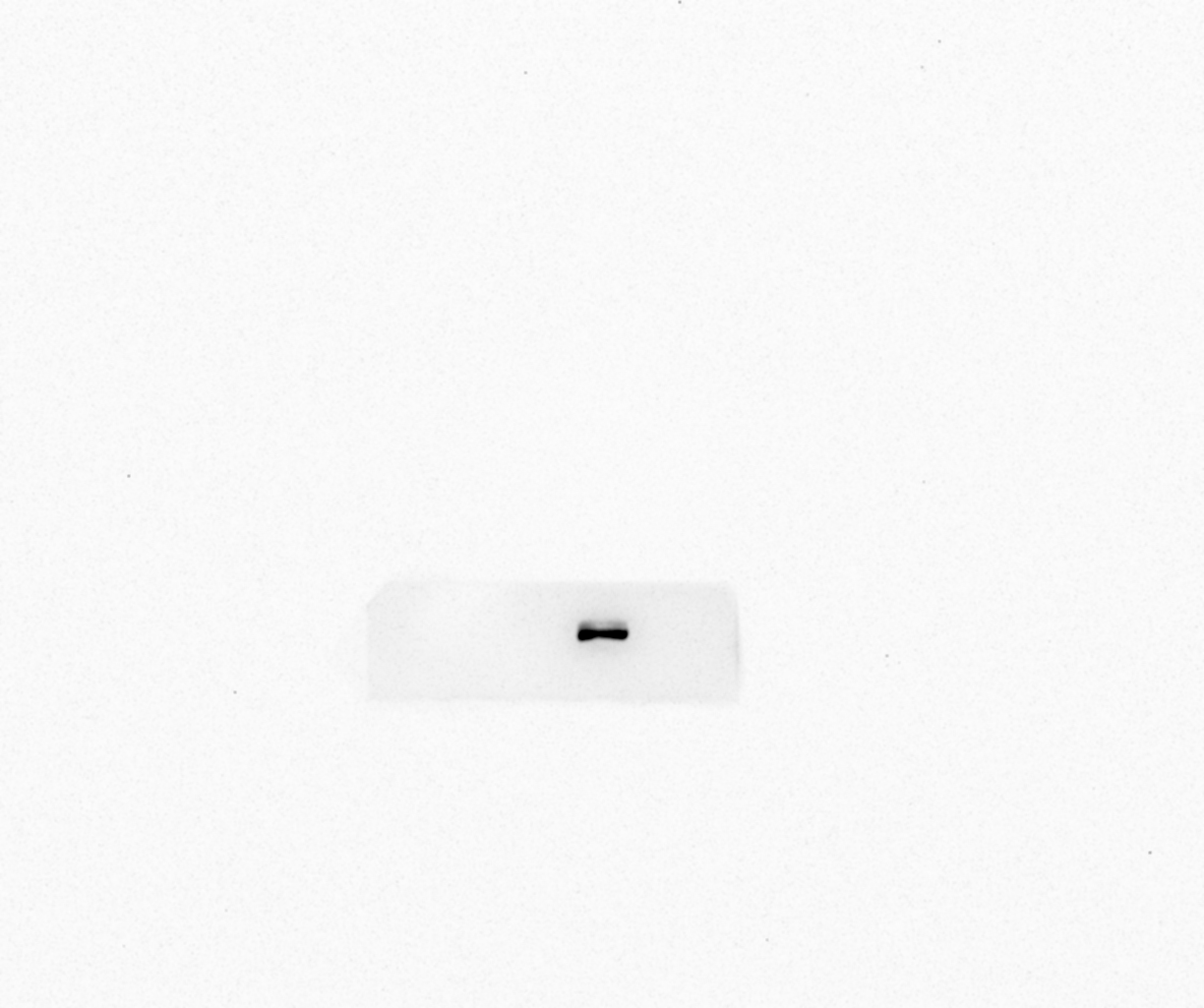

Supplement: Supplementary file 9 — Source data Fig. 6 [file 44319_2024_352_MOESM9_ESM.zip › Figure 6/6D/western Flag Input.tif]

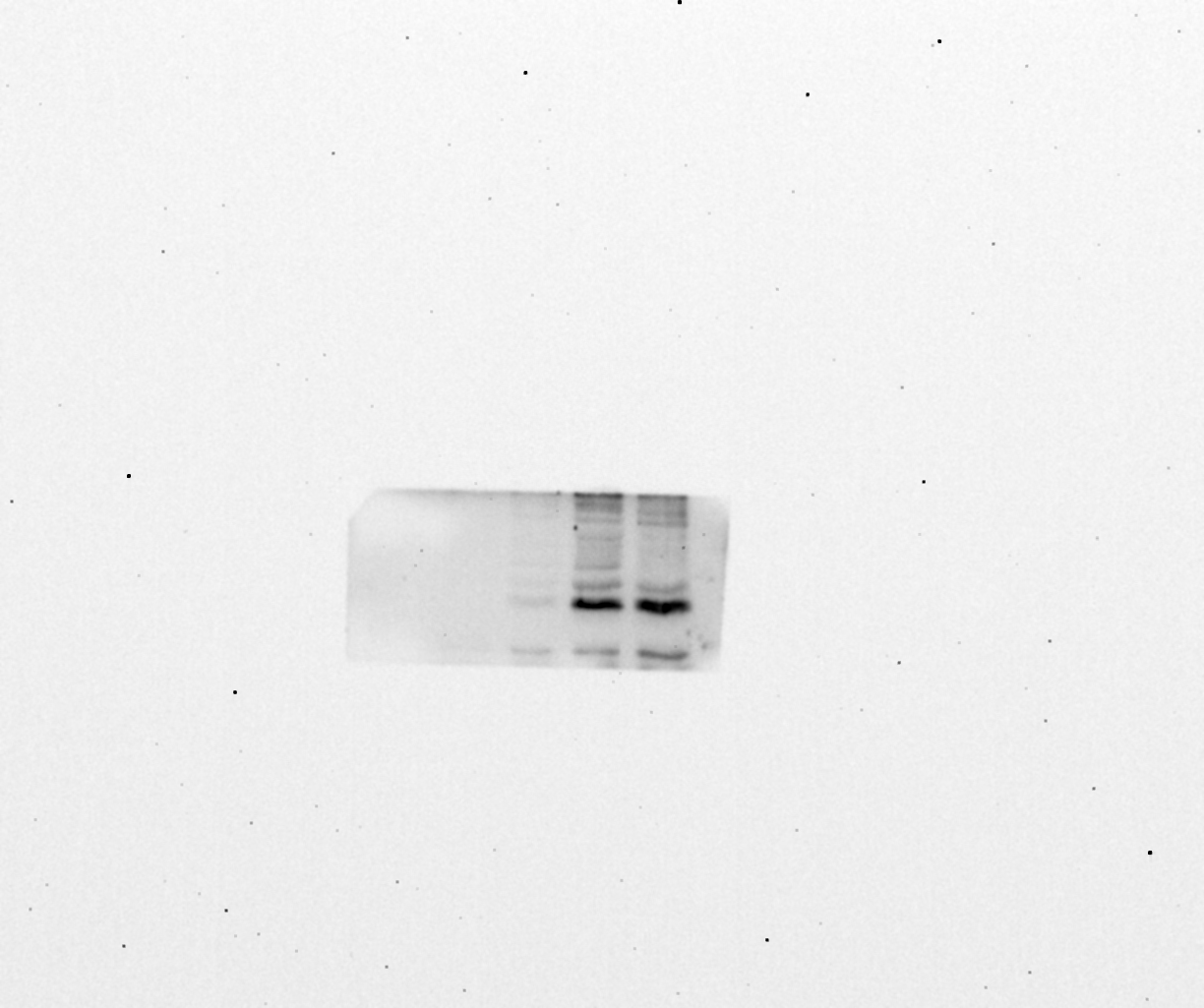

Supplement: Supplementary file 9 — Source data Fig. 6 [file 44319_2024_352_MOESM9_ESM.zip › Figure 6/6D/western HA IP.tif]

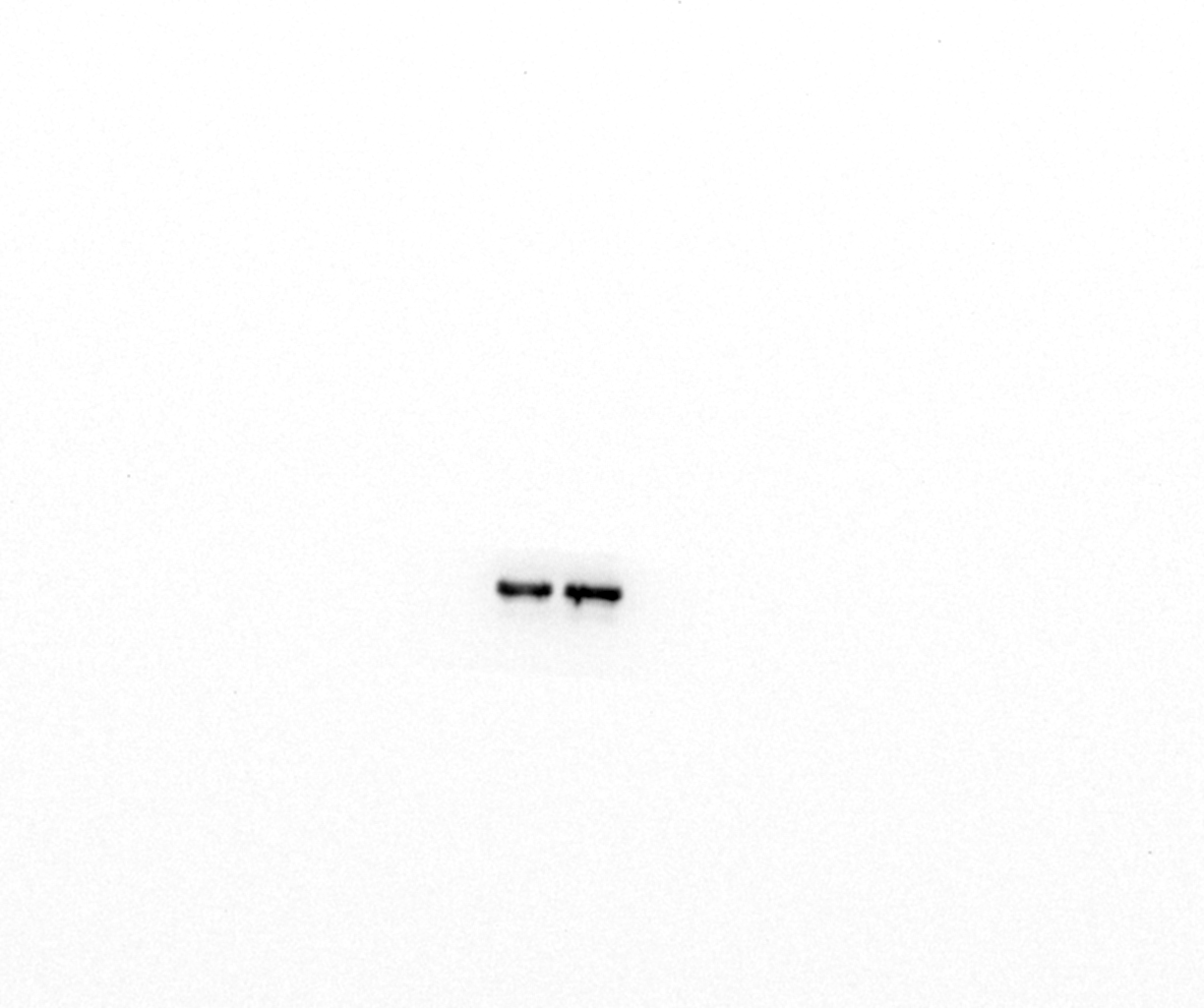

Supplement: Supplementary file 9 — Source data Fig. 6 [file 44319_2024_352_MOESM9_ESM.zip › Figure 6/6D/western HA Input.tif]

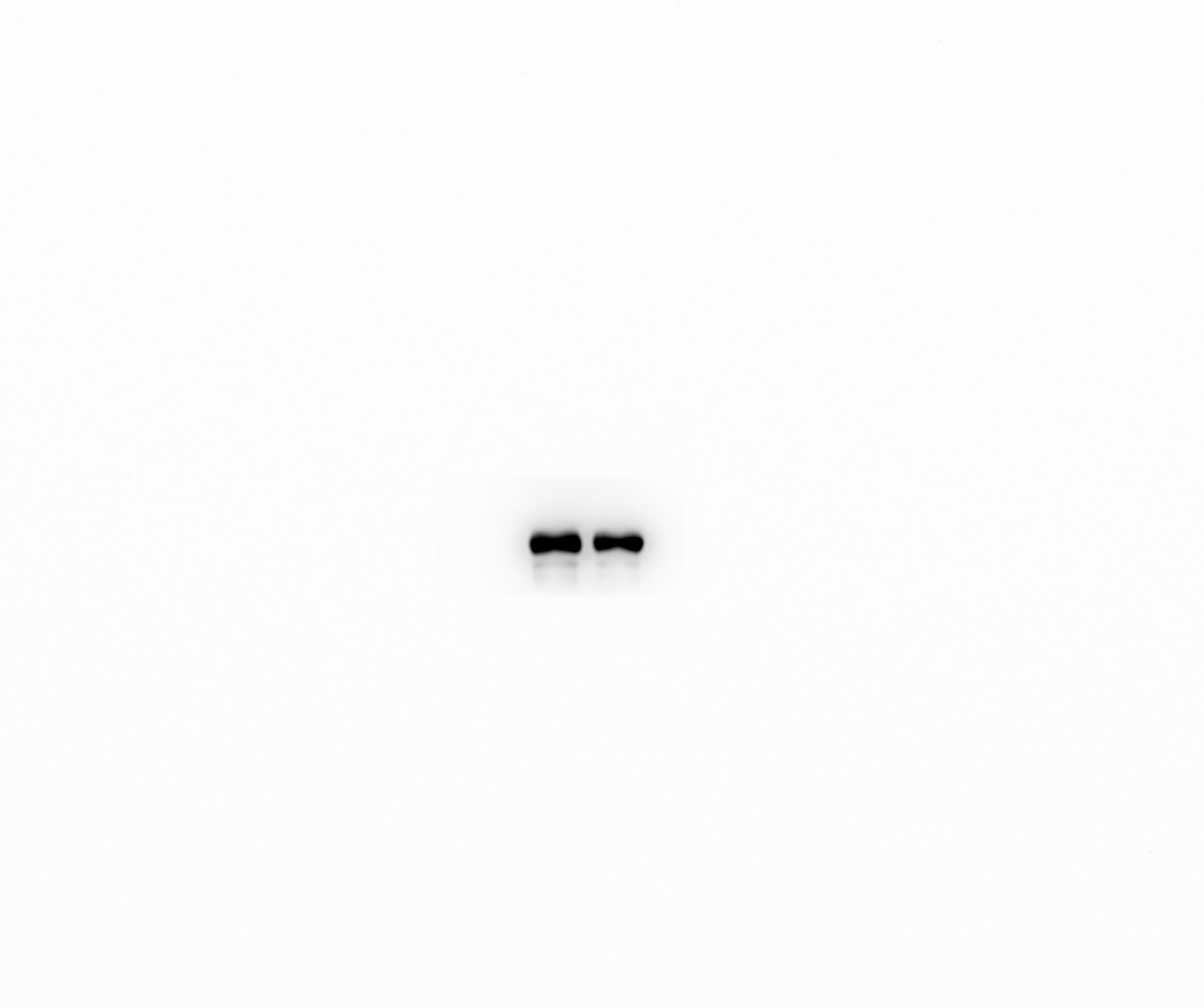

Supplement: Supplementary file 9 — Source data Fig. 6 [file 44319_2024_352_MOESM9_ESM.zip › Figure 6/6E/western Flag IP.tif]

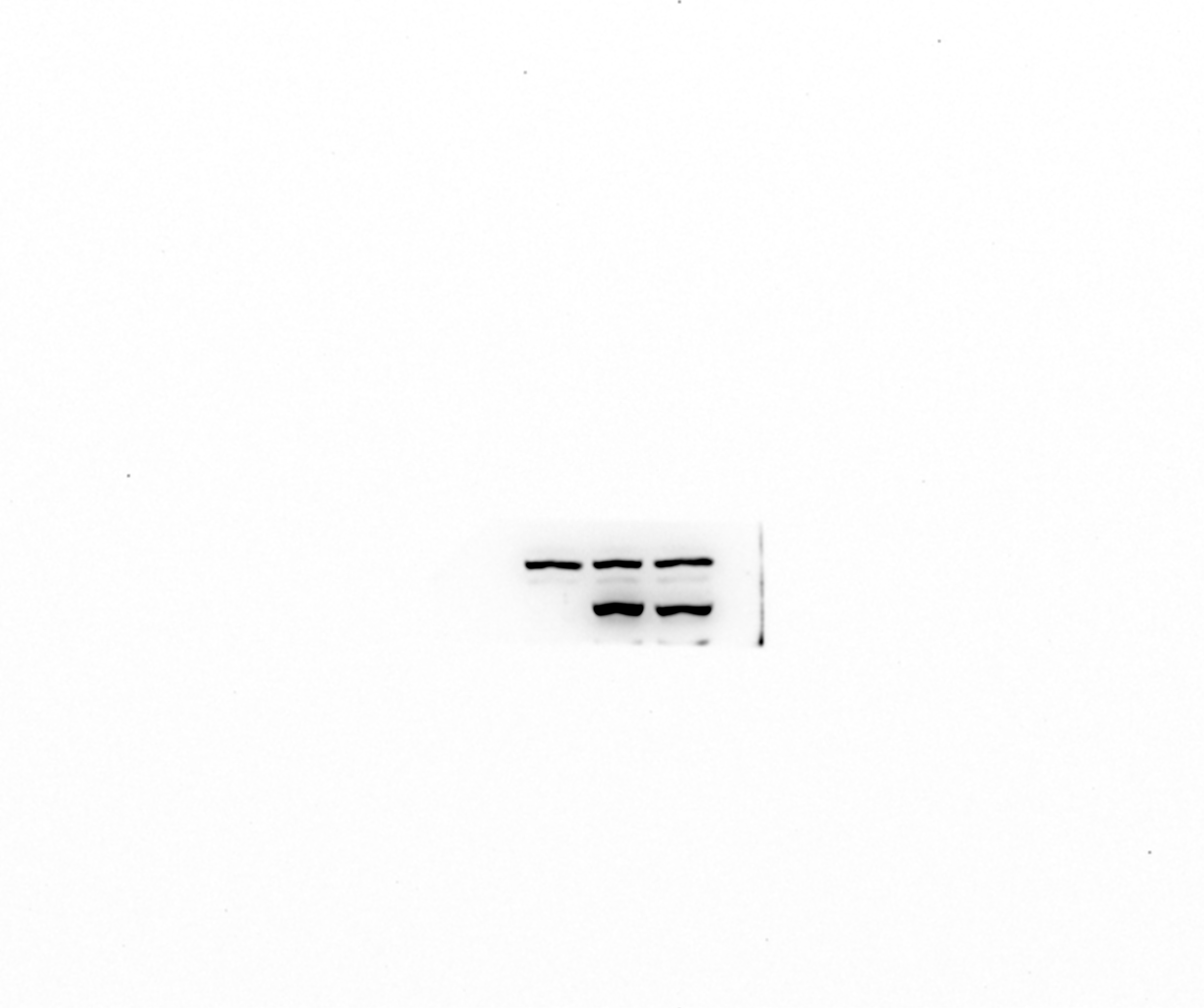

Supplement: Supplementary file 9 — Source data Fig. 6 [file 44319_2024_352_MOESM9_ESM.zip › Figure 6/6E/western Flag Input.tif]

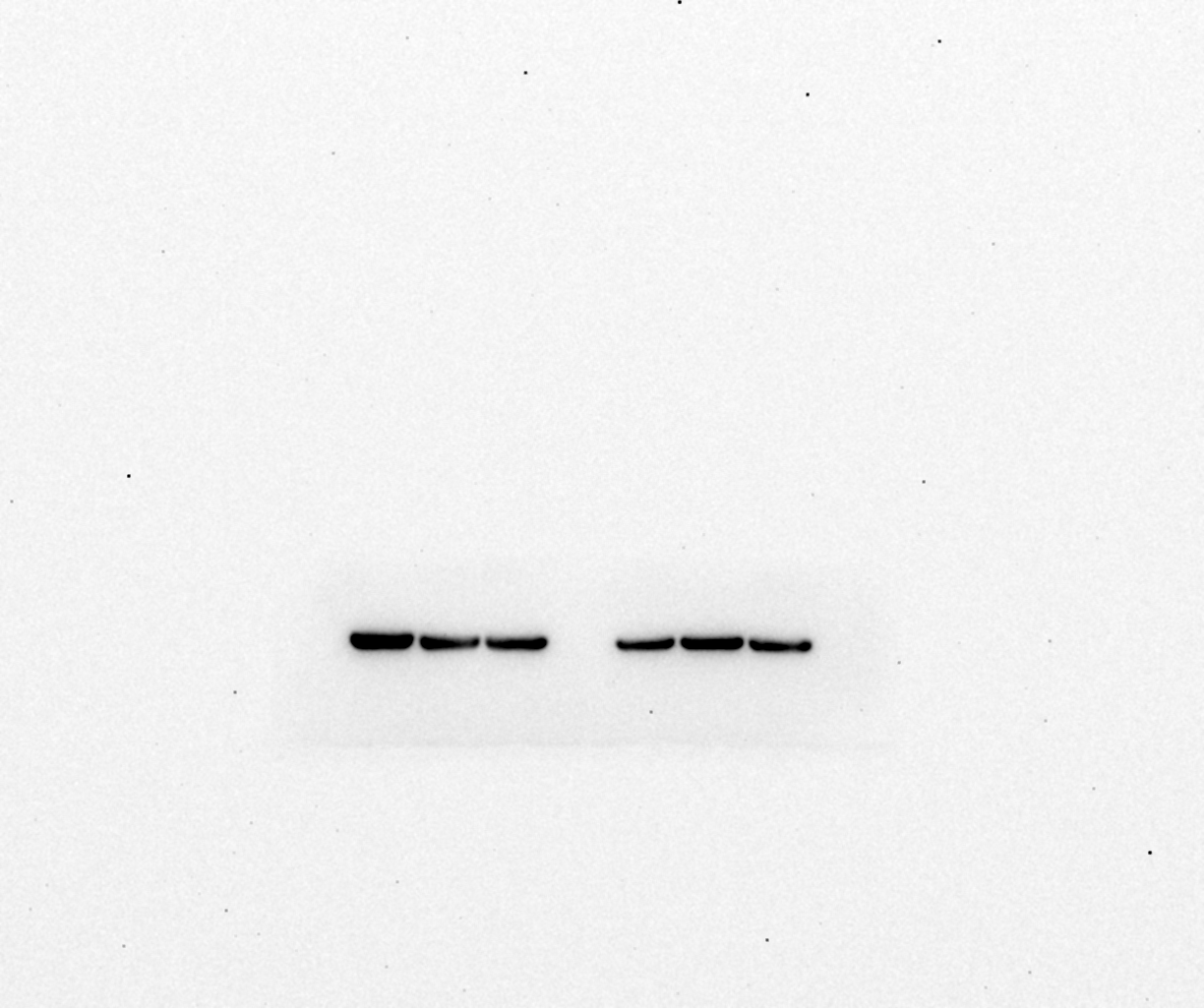

Supplement: Supplementary file 9 — Source data Fig. 6 [file 44319_2024_352_MOESM9_ESM.zip › Figure 6/6E/western GAPDH.tif]

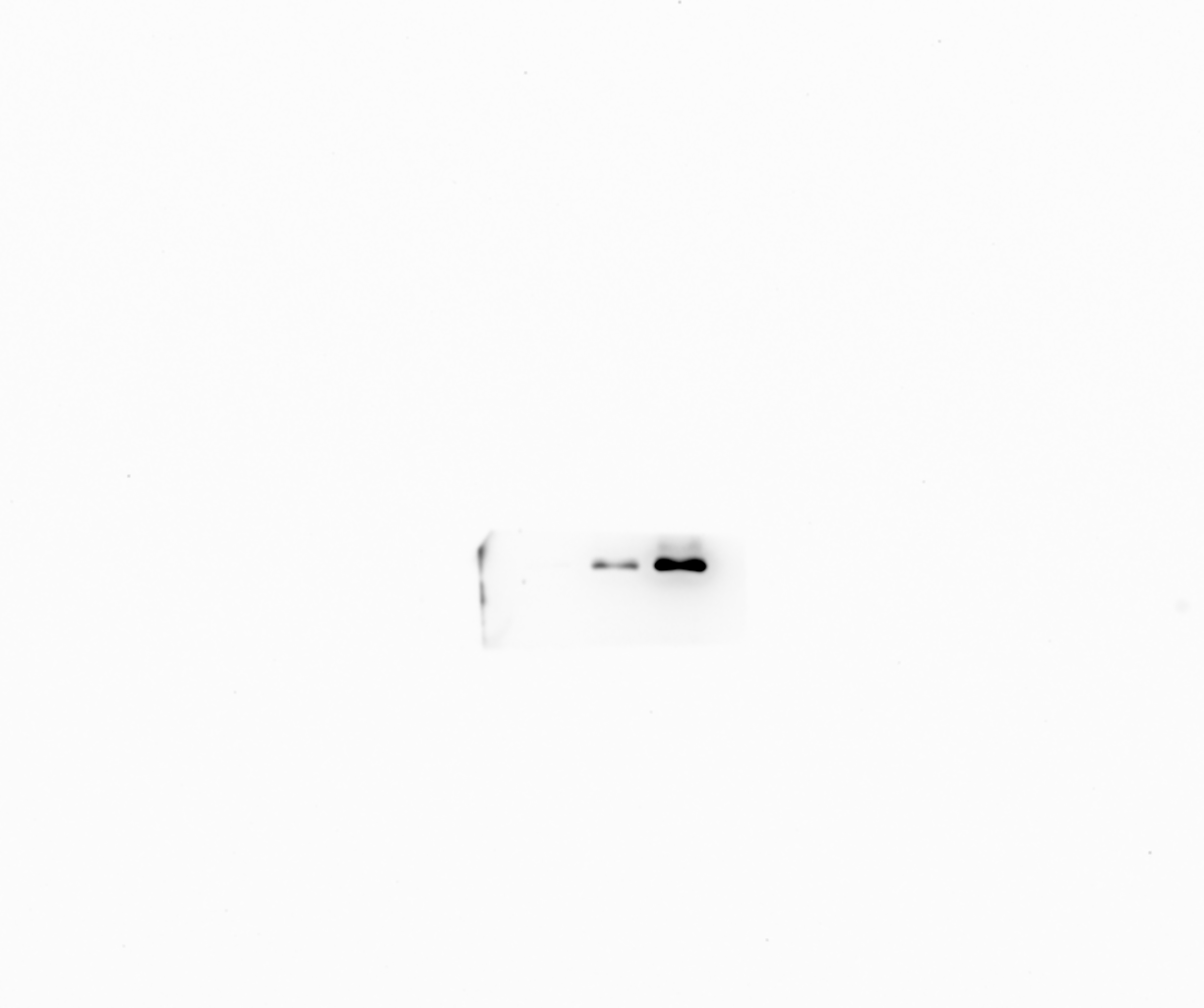

Supplement: Supplementary file 9 — Source data Fig. 6 [file 44319_2024_352_MOESM9_ESM.zip › Figure 6/6E/western HA IP -2.tif]

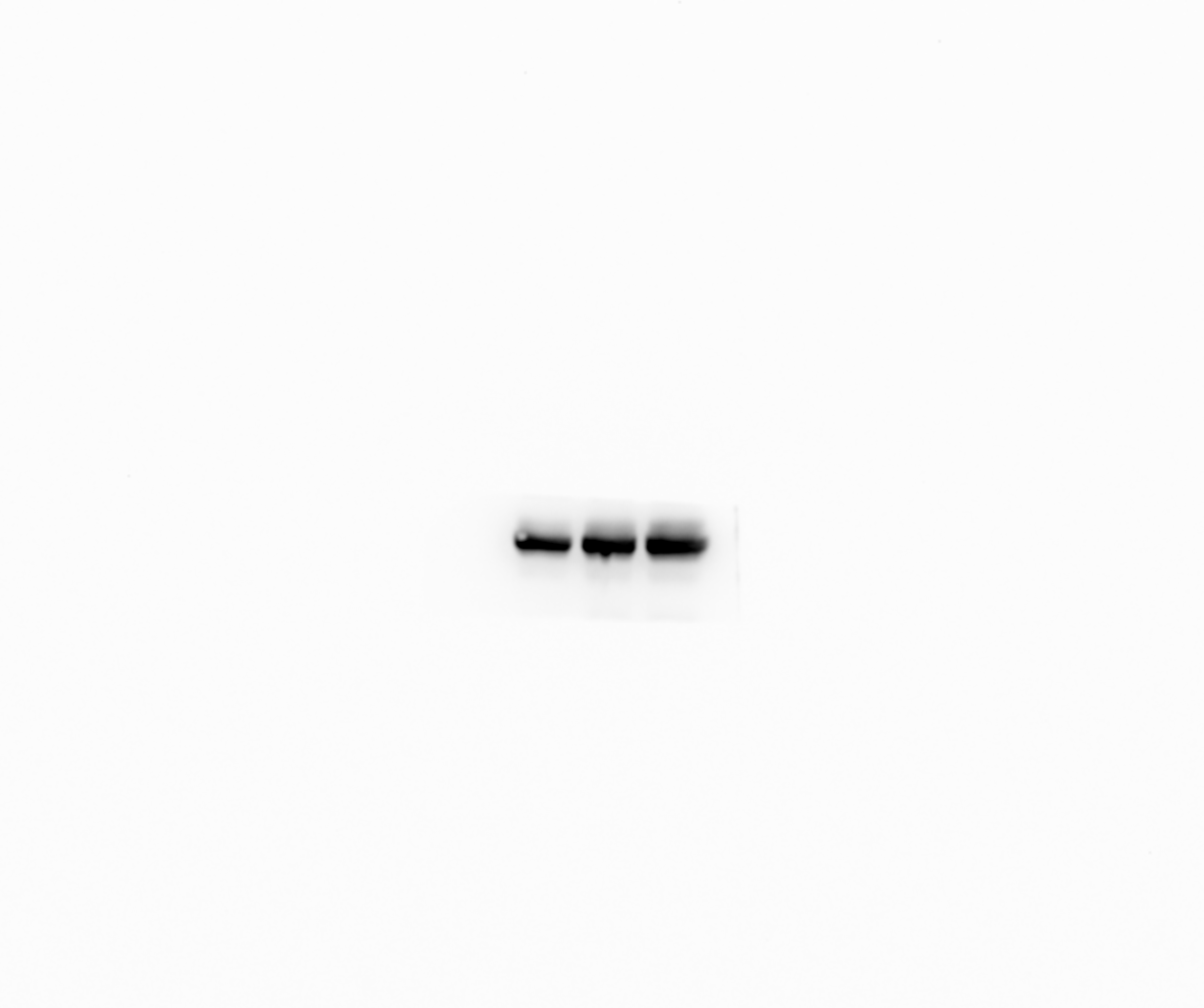

Supplement: Supplementary file 9 — Source data Fig. 6 [file 44319_2024_352_MOESM9_ESM.zip › Figure 6/6E/western HA Input.tif]

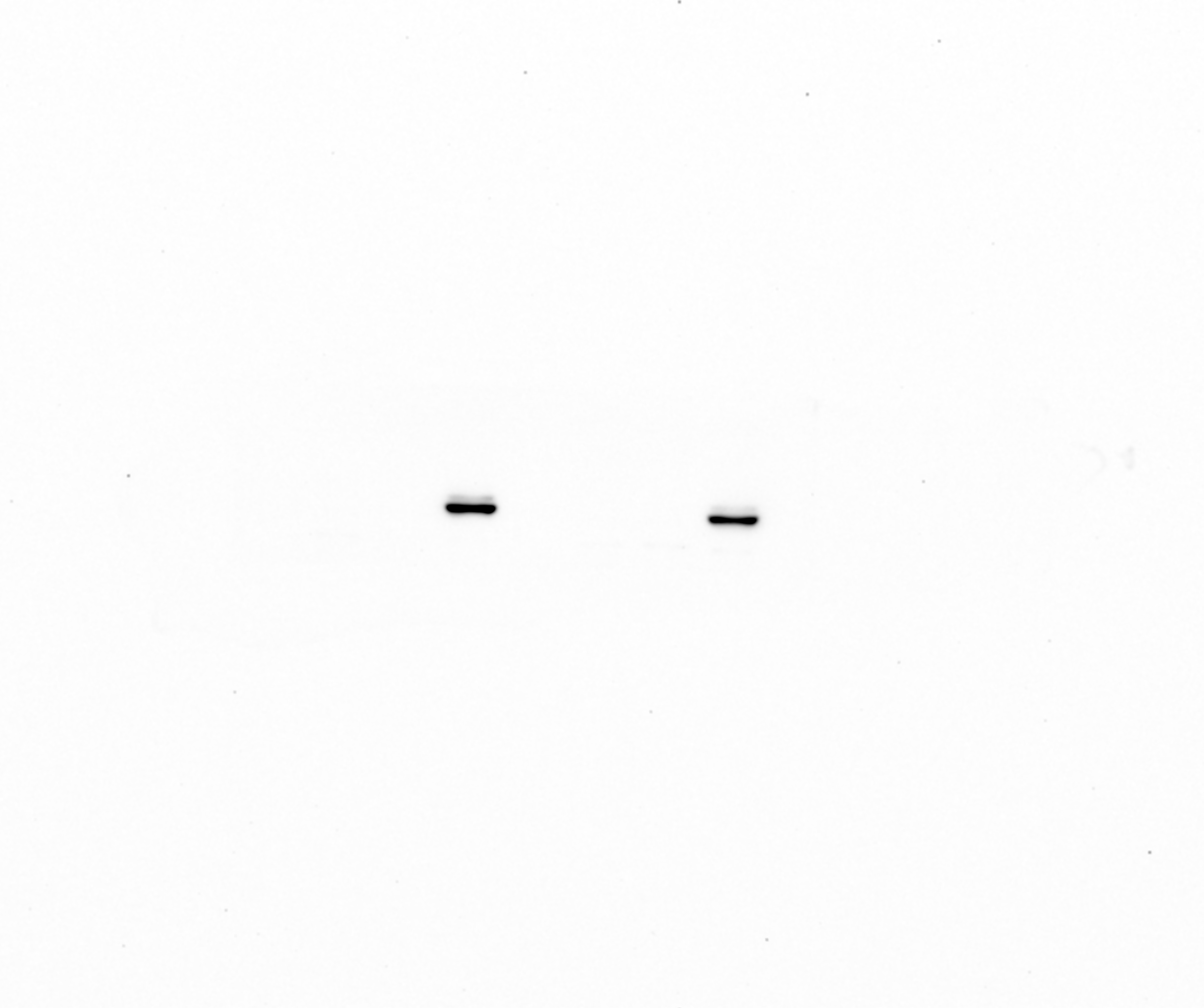

Supplement: Supplementary file 9 — Source data Fig. 6 [file 44319_2024_352_MOESM9_ESM.zip › Figure 6/6E/western myc Input.tif]

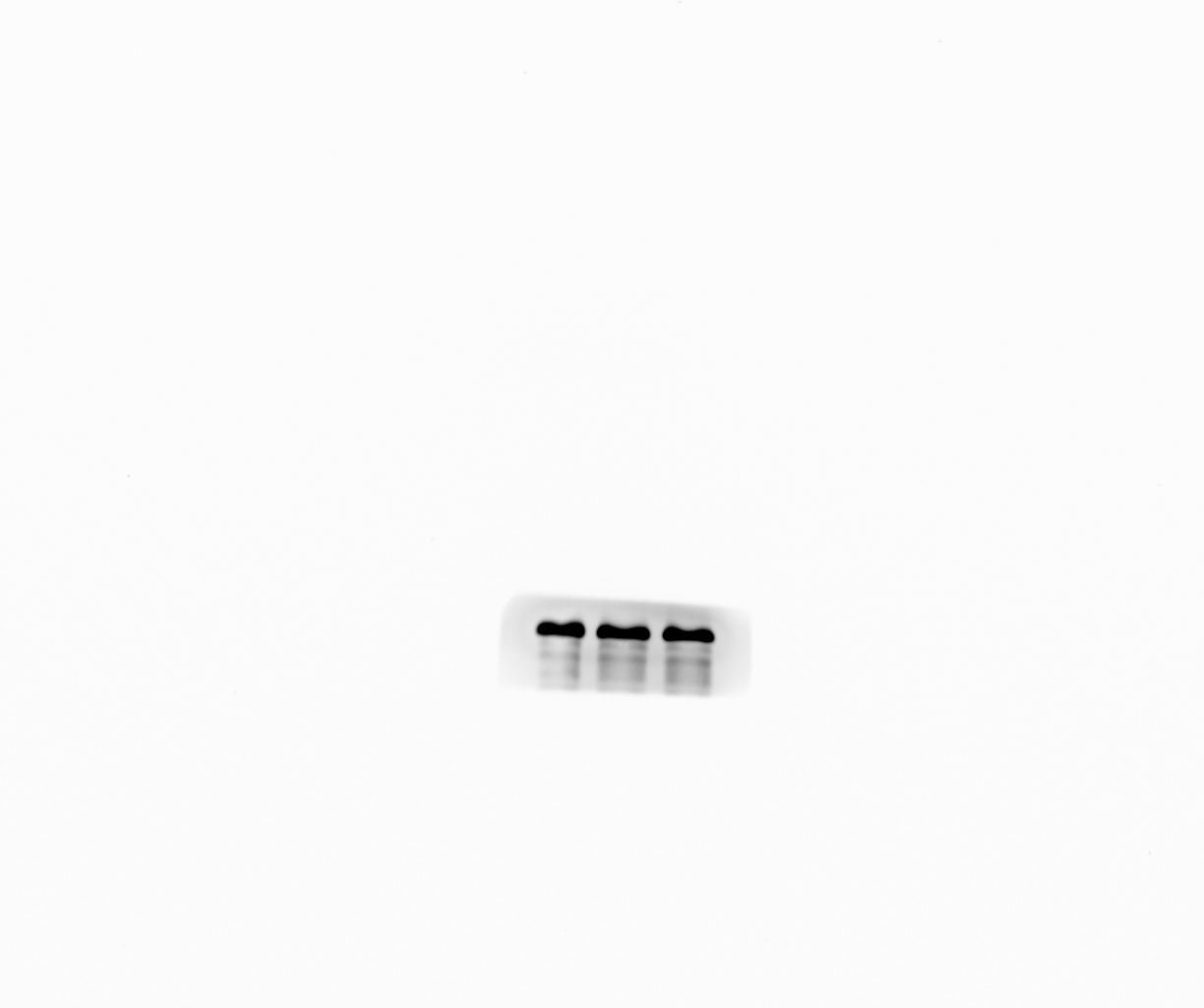

Supplement: Supplementary file 9 — Source data Fig. 6 [file 44319_2024_352_MOESM9_ESM.zip › Figure 6/6F/western IP Flag.tif]

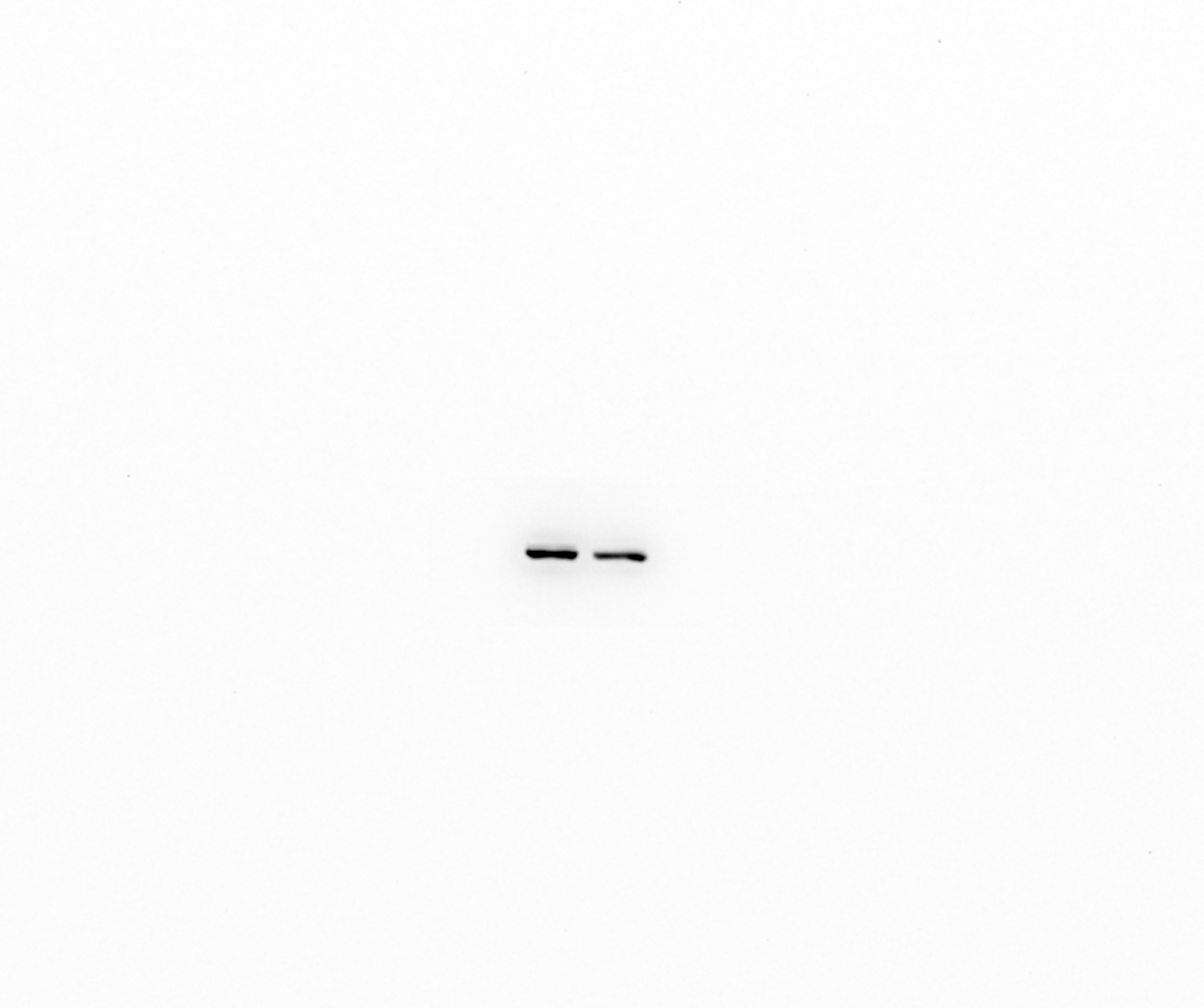

Supplement: Supplementary file 9 — Source data Fig. 6 [file 44319_2024_352_MOESM9_ESM.zip › Figure 6/6F/western myc Input.tif]

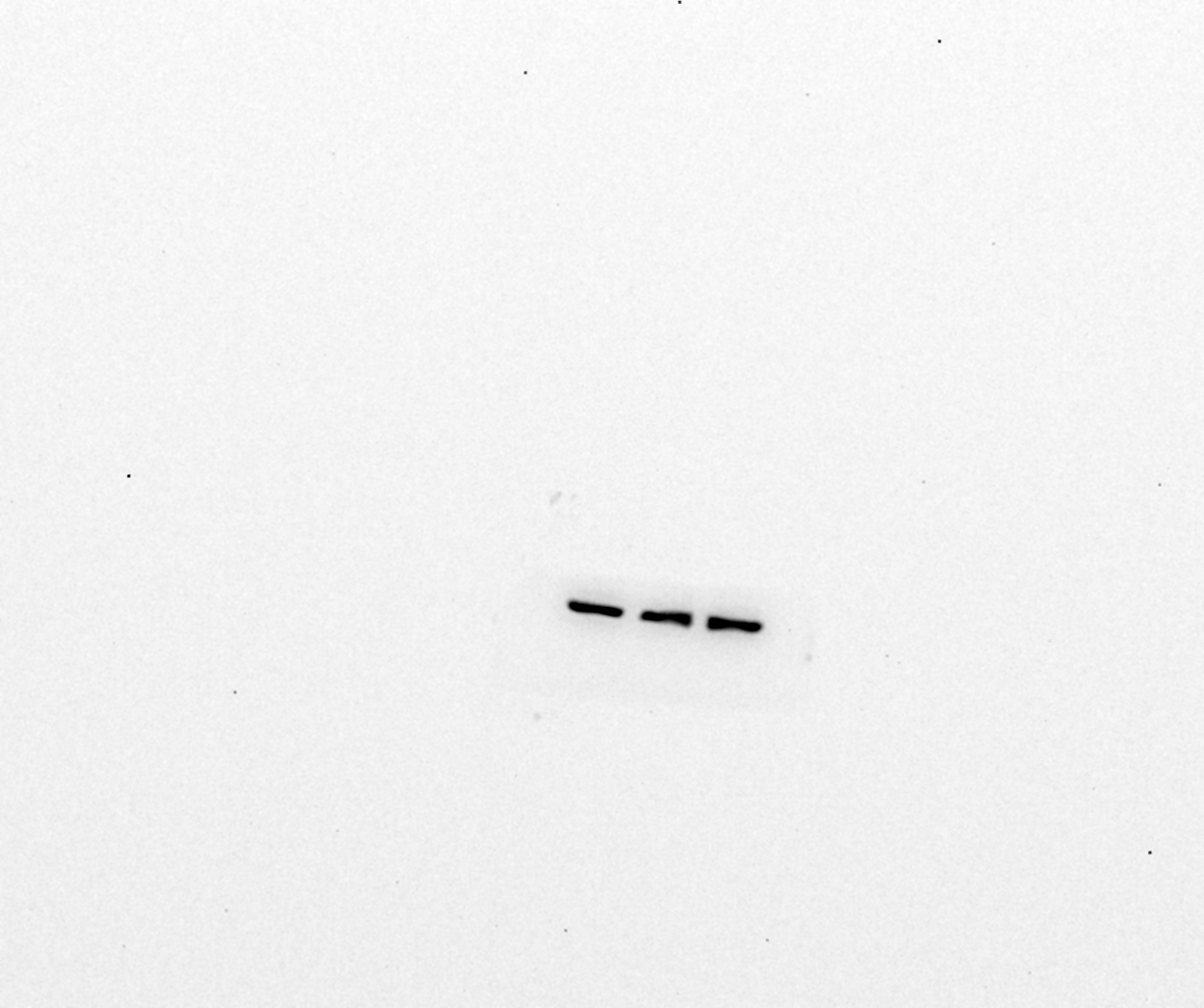

Supplement: Supplementary file 9 — Source data Fig. 6 [file 44319_2024_352_MOESM9_ESM.zip › Figure 6/6F/western ACTIN Input.tif]

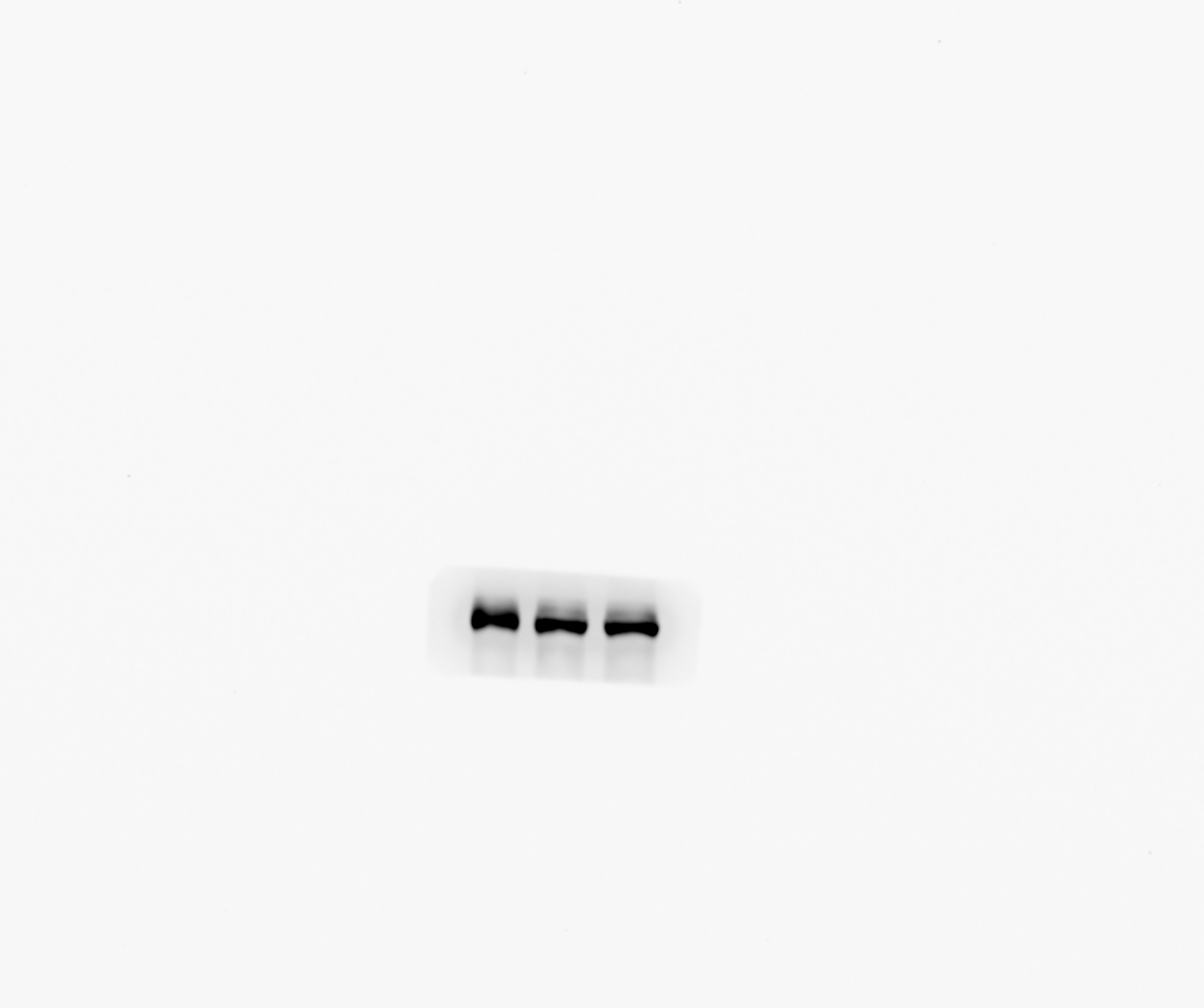

Supplement: Supplementary file 9 — Source data Fig. 6 [file 44319_2024_352_MOESM9_ESM.zip › Figure 6/6F/western Flag Input.tif]

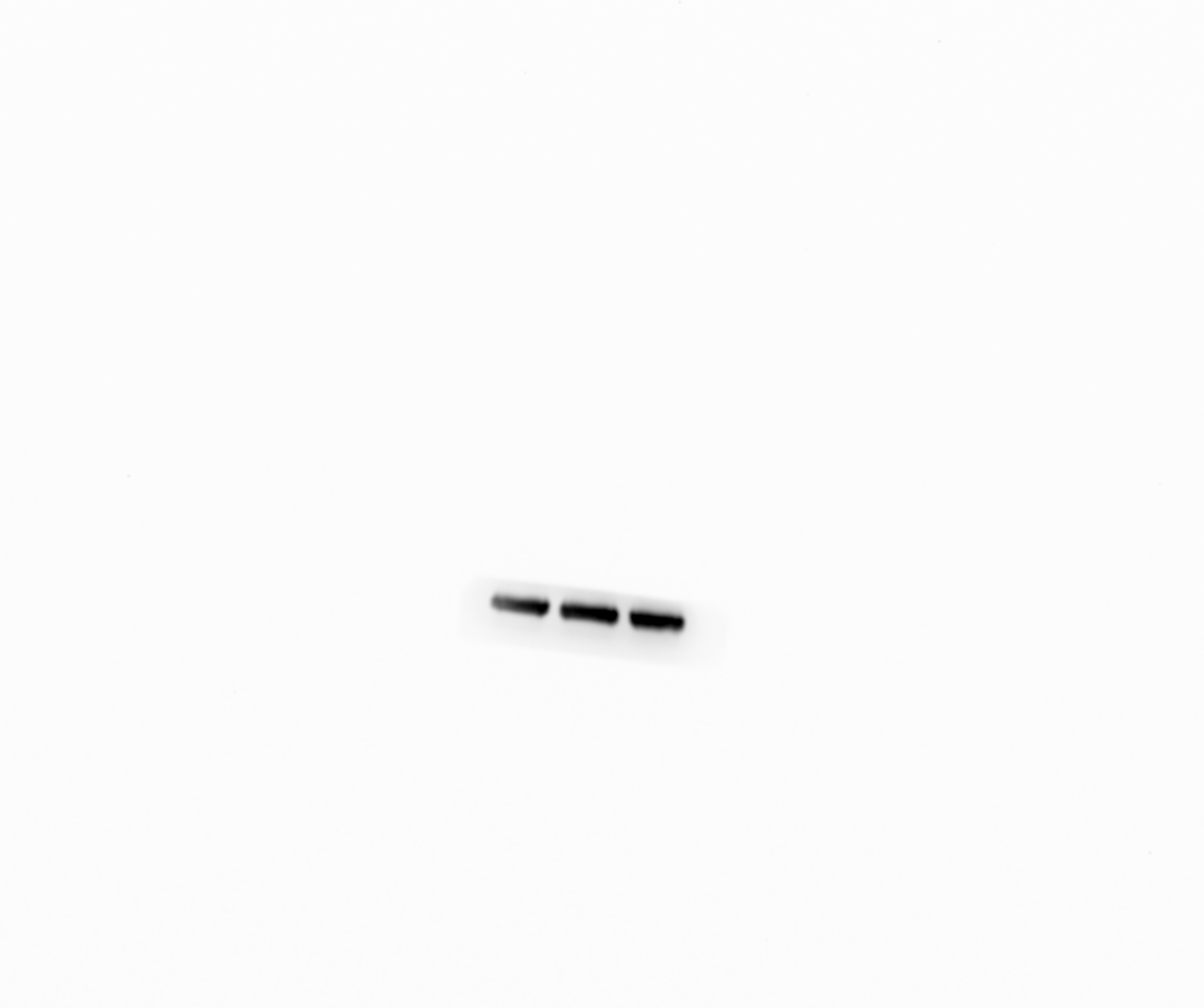

Supplement: Supplementary file 9 — Source data Fig. 6 [file 44319_2024_352_MOESM9_ESM.zip › Figure 6/6F/western HA Input.tif]

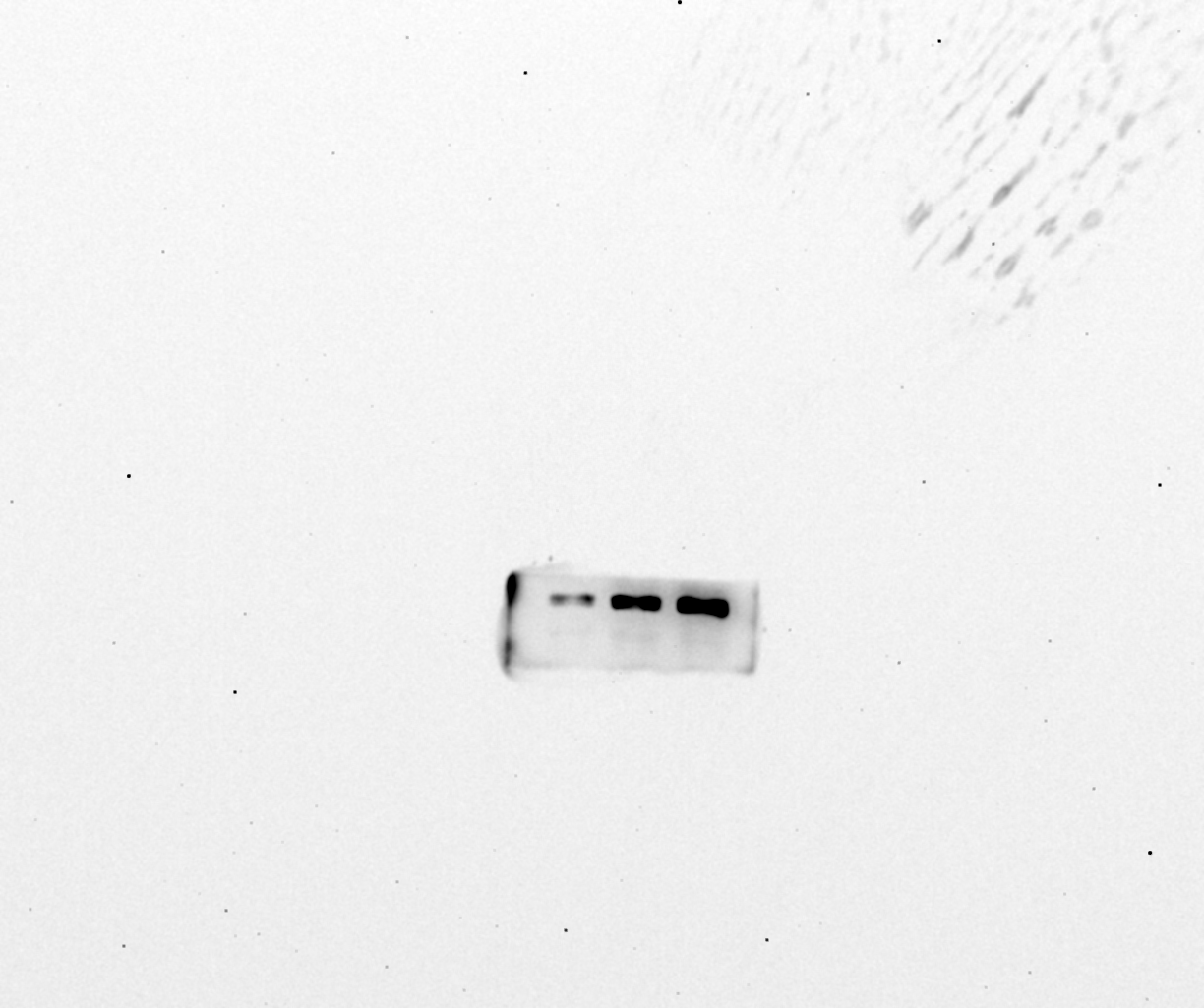

Supplement: Supplementary file 9 — Source data Fig. 6 [file 44319_2024_352_MOESM9_ESM.zip › Figure 6/6F/western HA IP.tif]

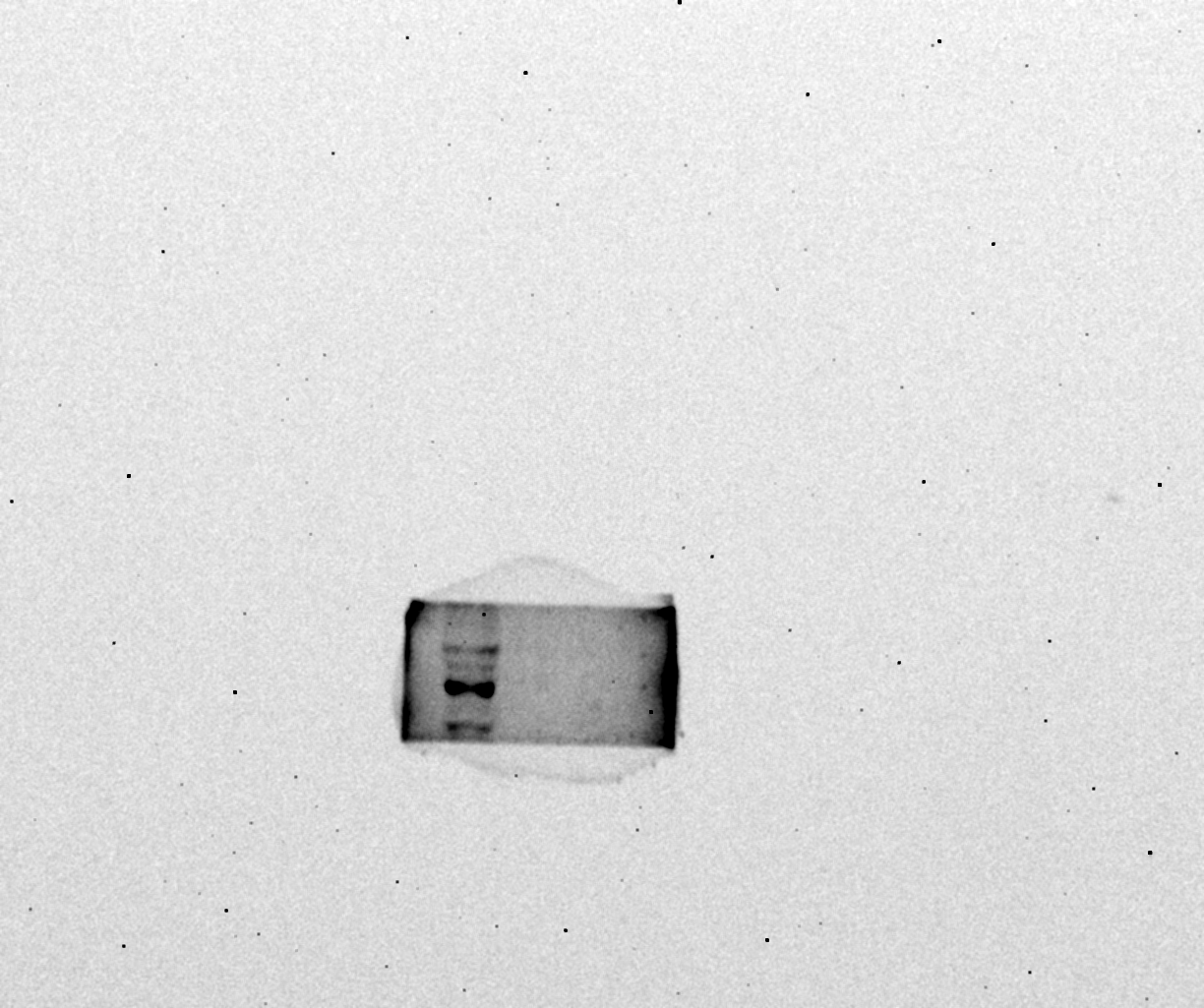

Supplement: Supplementary file 9 — Source data Fig. 6 [file 44319_2024_352_MOESM9_ESM.zip › Figure 6/6G/western DYRK4 Input.tif]

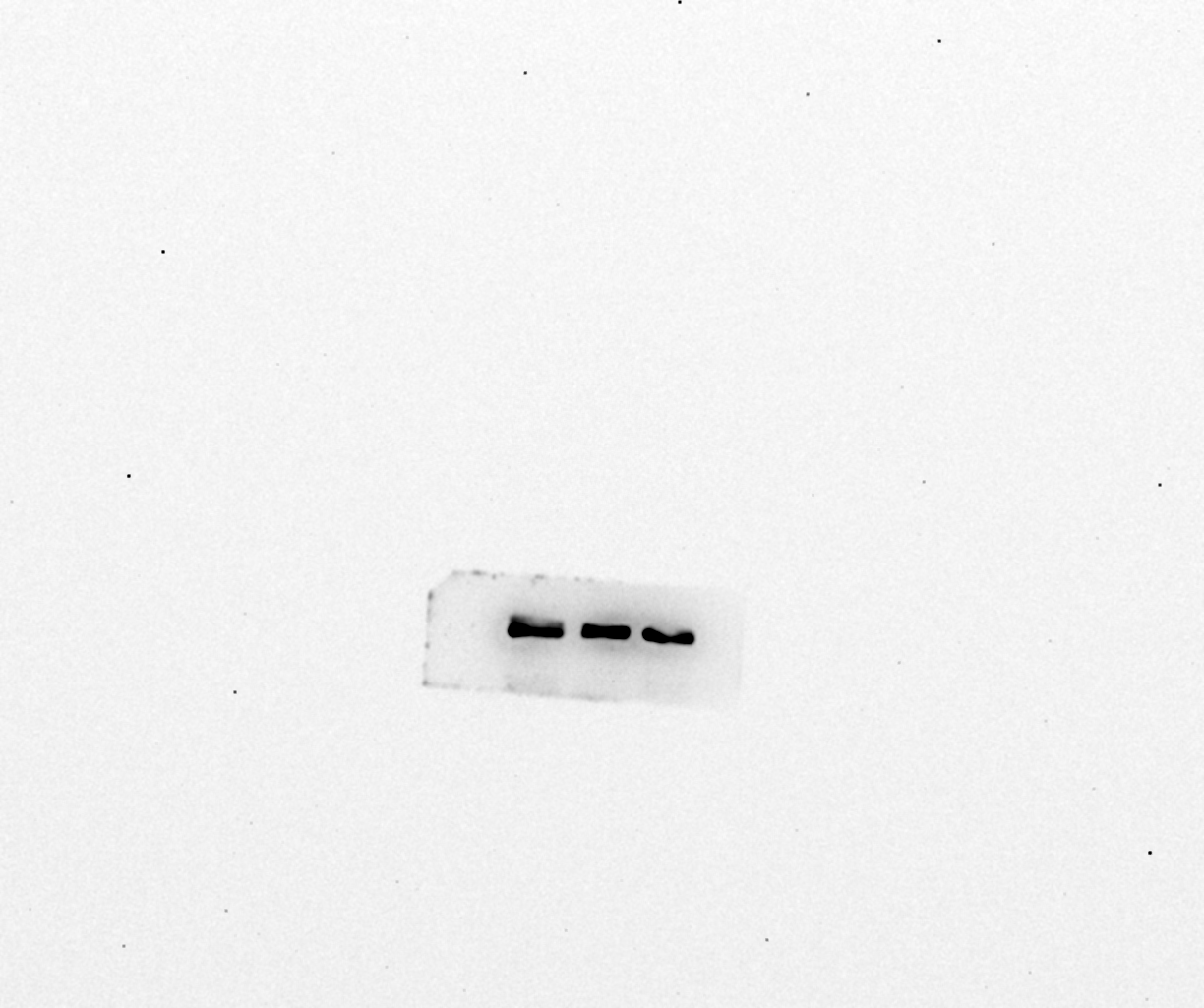

Supplement: Supplementary file 9 — Source data Fig. 6 [file 44319_2024_352_MOESM9_ESM.zip › Figure 6/6G/western Flag IP.tif]

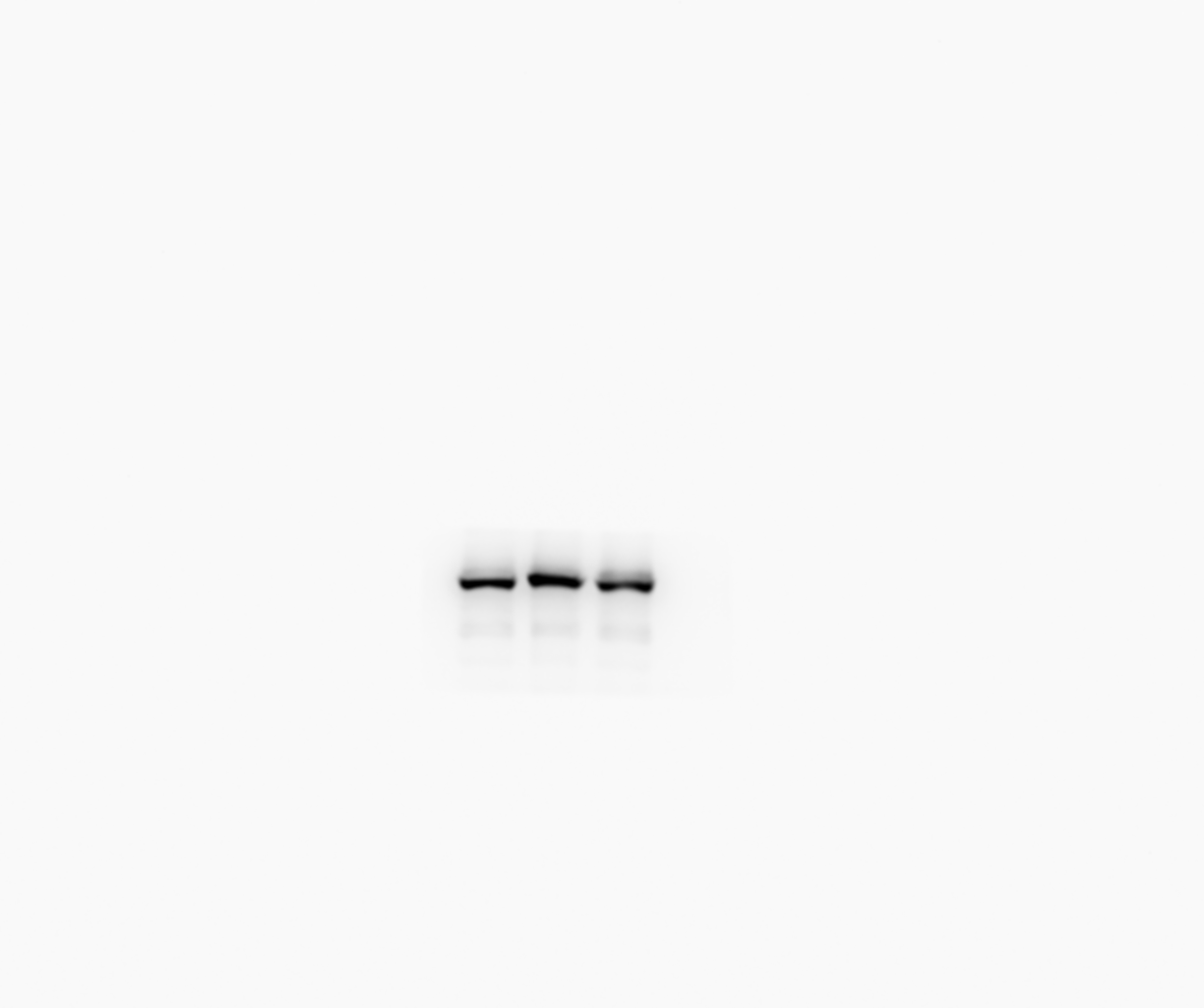

Supplement: Supplementary file 9 — Source data Fig. 6 [file 44319_2024_352_MOESM9_ESM.zip › Figure 6/6G/western Flag Input.tif]

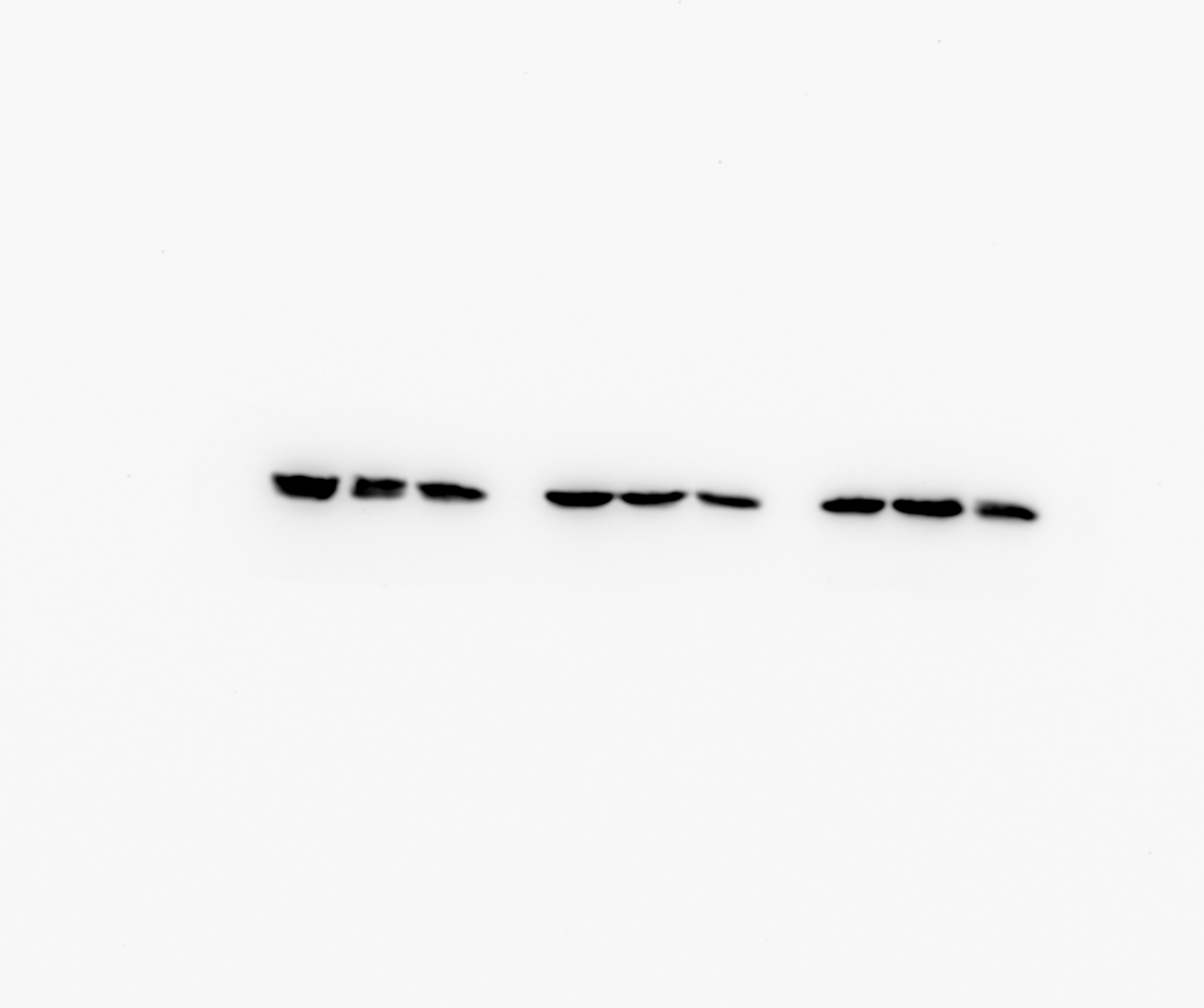

Supplement: Supplementary file 9 — Source data Fig. 6 [file 44319_2024_352_MOESM9_ESM.zip › Figure 6/6G/western GAPDH.tif]

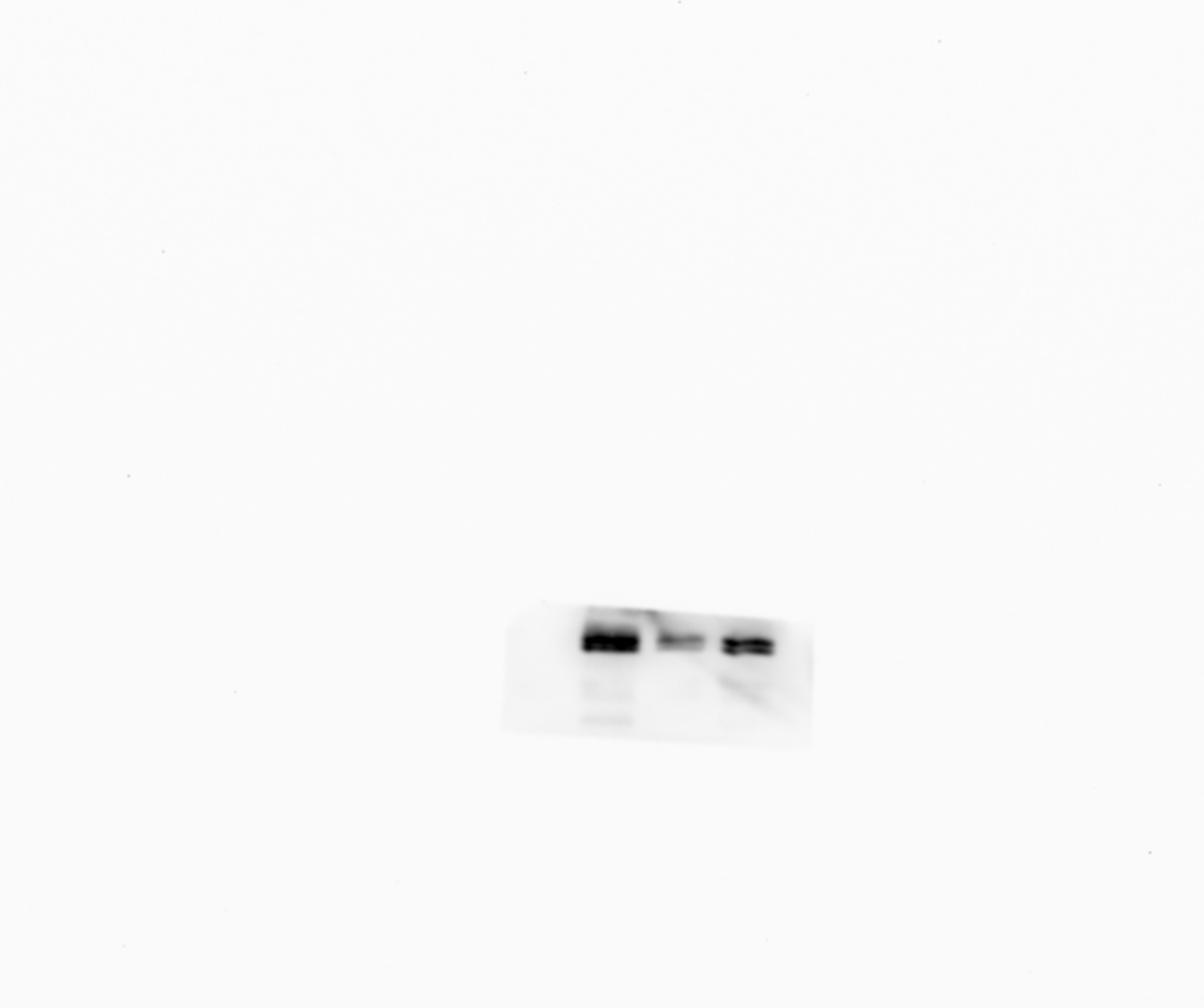

Supplement: Supplementary file 9 — Source data Fig. 6 [file 44319_2024_352_MOESM9_ESM.zip › Figure 6/6G/western HA IP.tif]

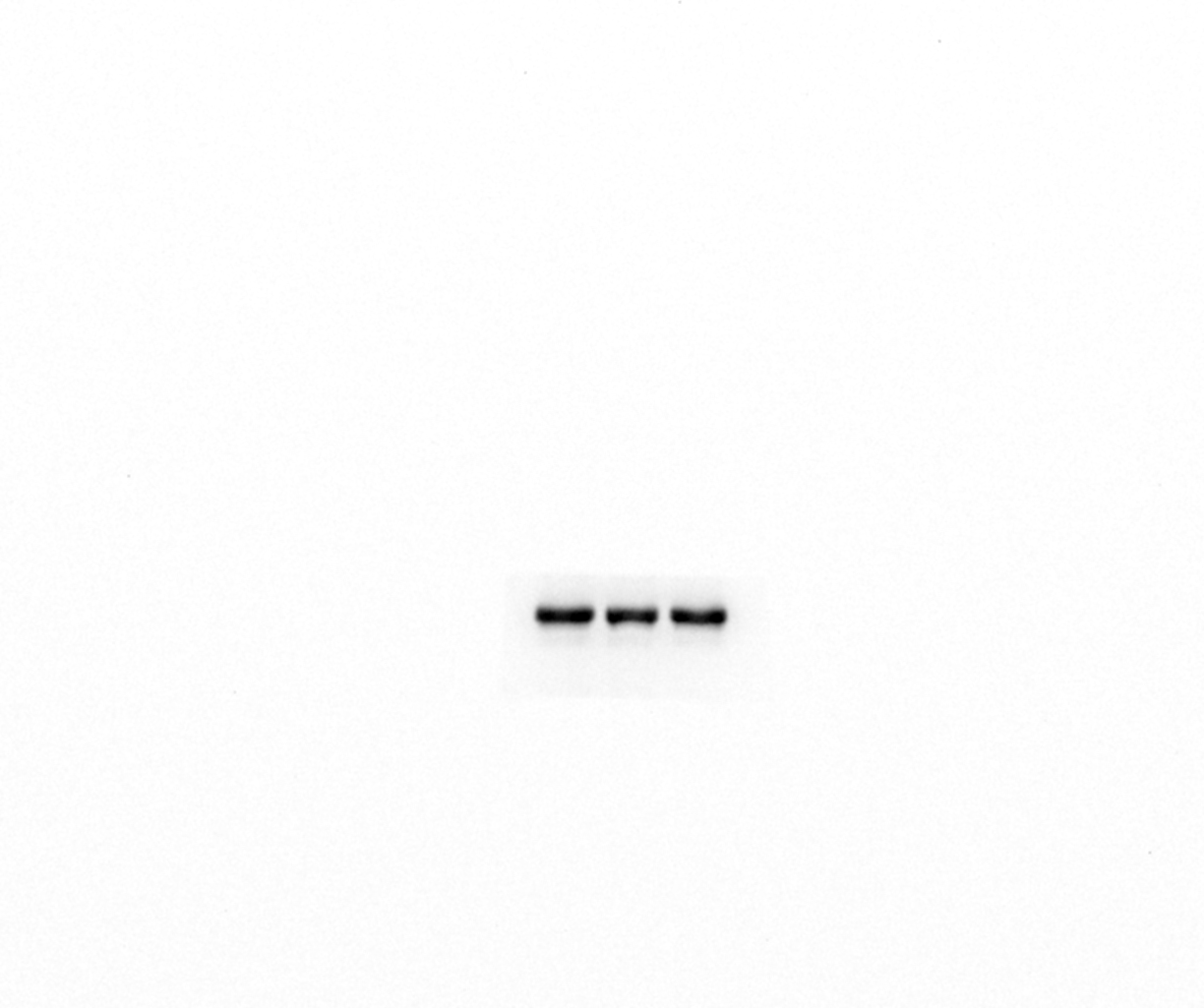

Supplement: Supplementary file 9 — Source data Fig. 6 [file 44319_2024_352_MOESM9_ESM.zip › Figure 6/6G/western HA Input.tif]

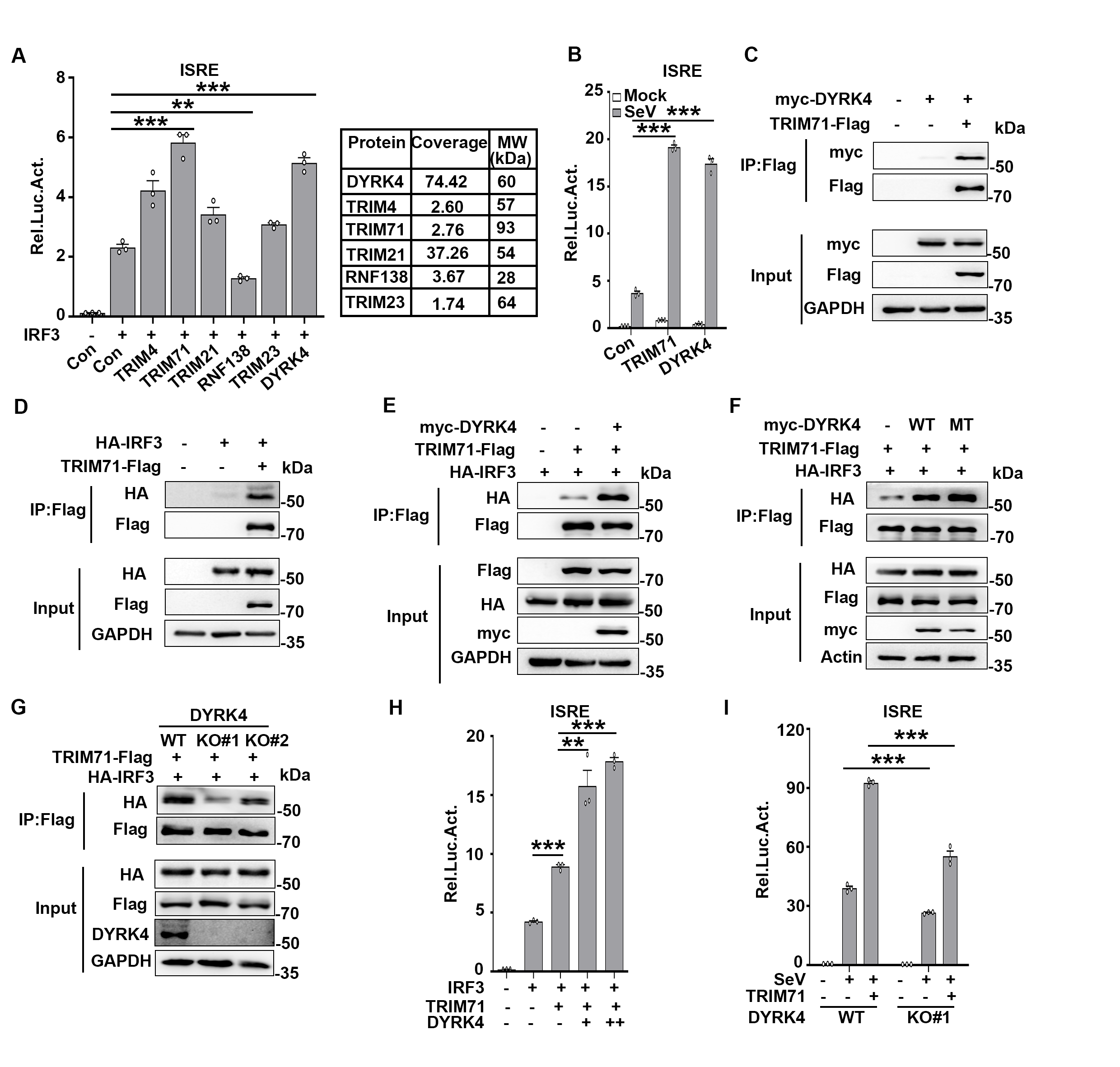

Supplement: Supplementary file 9 — Source data Fig. 6 [file 44319_2024_352_MOESM9_ESM.zip › Figure 6/Figure 6.tif]

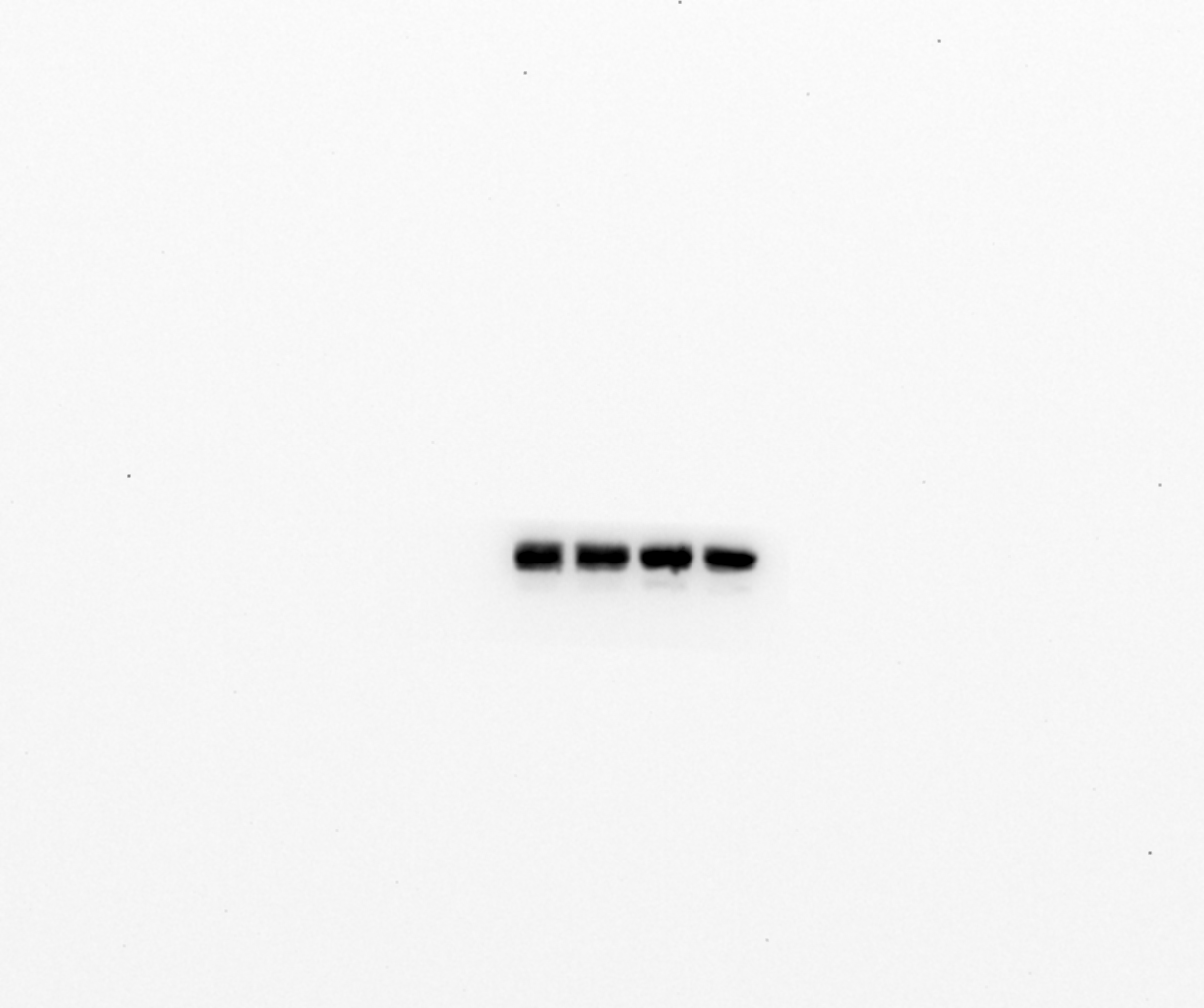

Supplement: Supplementary file 10 — Source data Fig. 7 [file 44319_2024_352_MOESM10_ESM.zip › Figure 7/7A/western Flag IP.tif]

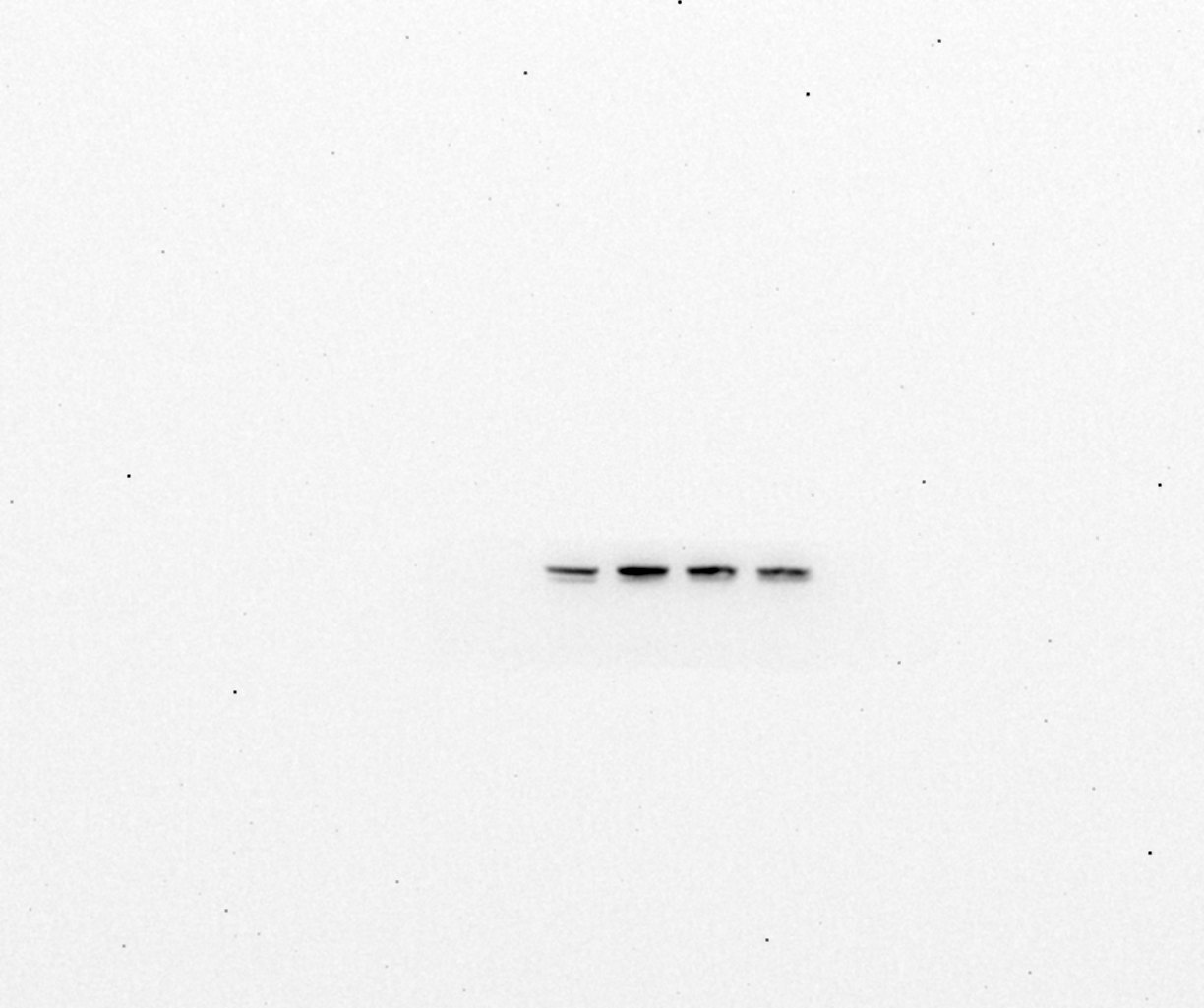

Supplement: Supplementary file 10 — Source data Fig. 7 [file 44319_2024_352_MOESM10_ESM.zip › Figure 7/7A/western Flag Input.tif]

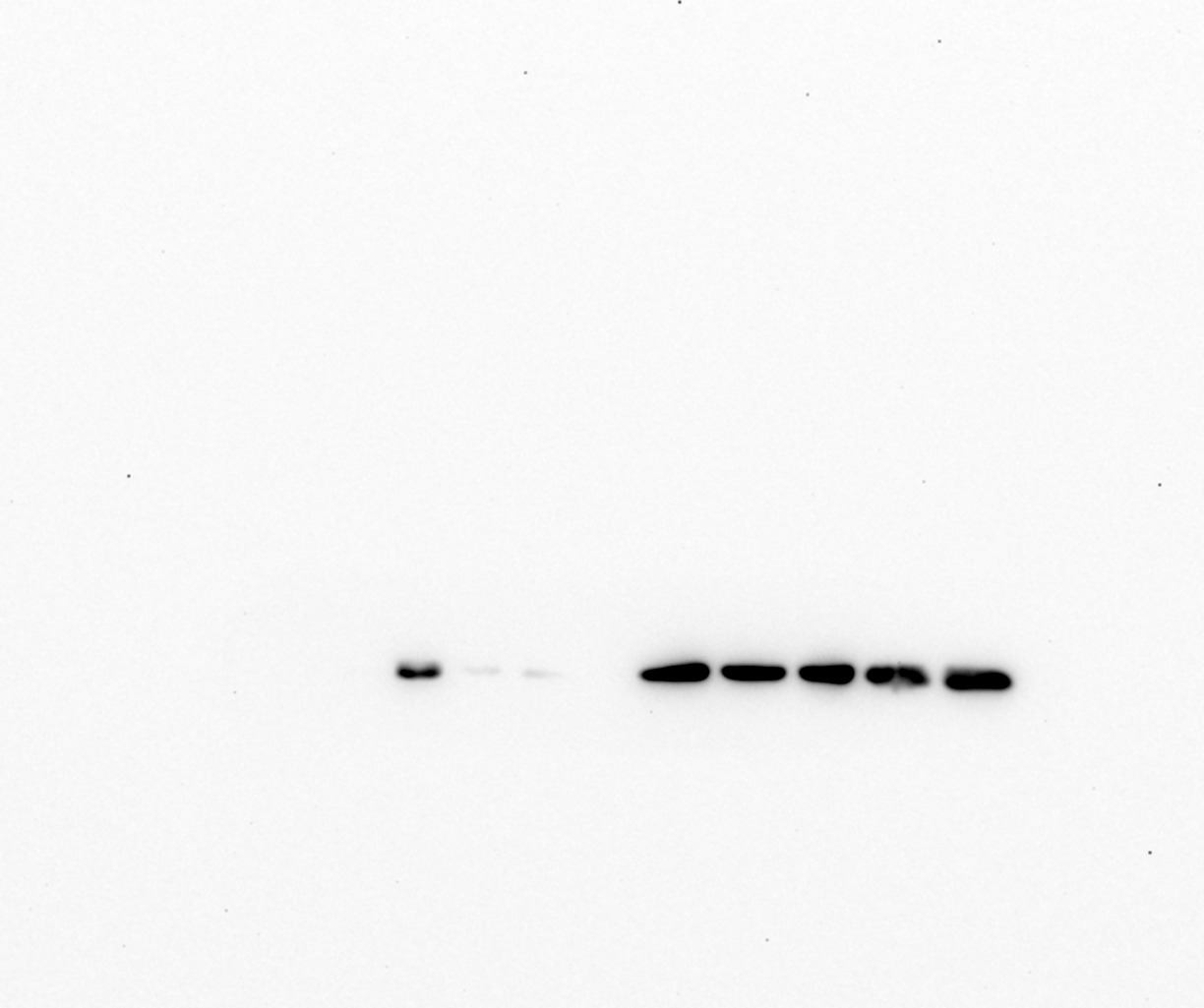

Supplement: Supplementary file 10 — Source data Fig. 7 [file 44319_2024_352_MOESM10_ESM.zip › Figure 7/7A/western GAPDH Input.tif]

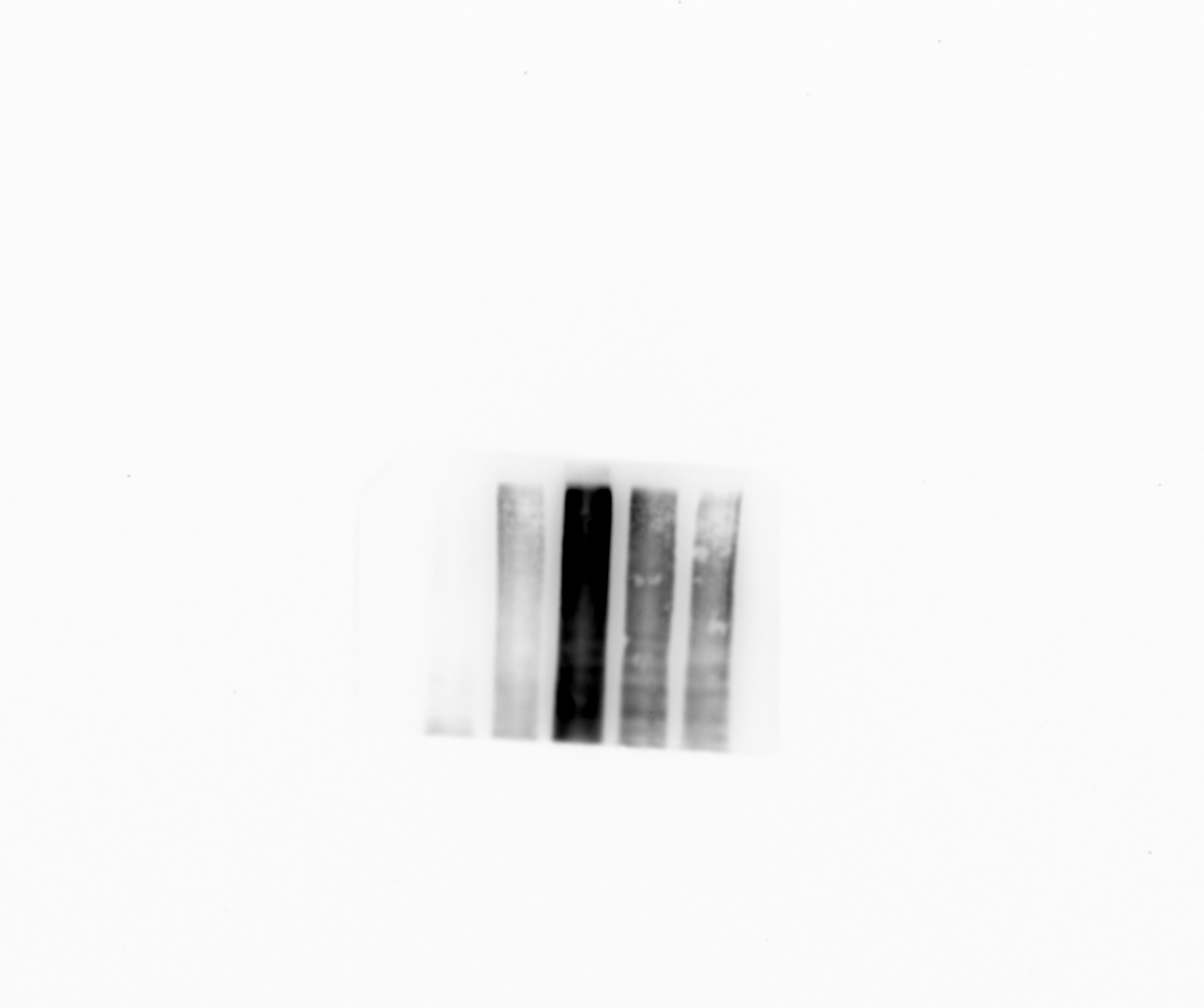

Supplement: Supplementary file 10 — Source data Fig. 7 [file 44319_2024_352_MOESM10_ESM.zip › Figure 7/7A/western HA IP.tif]

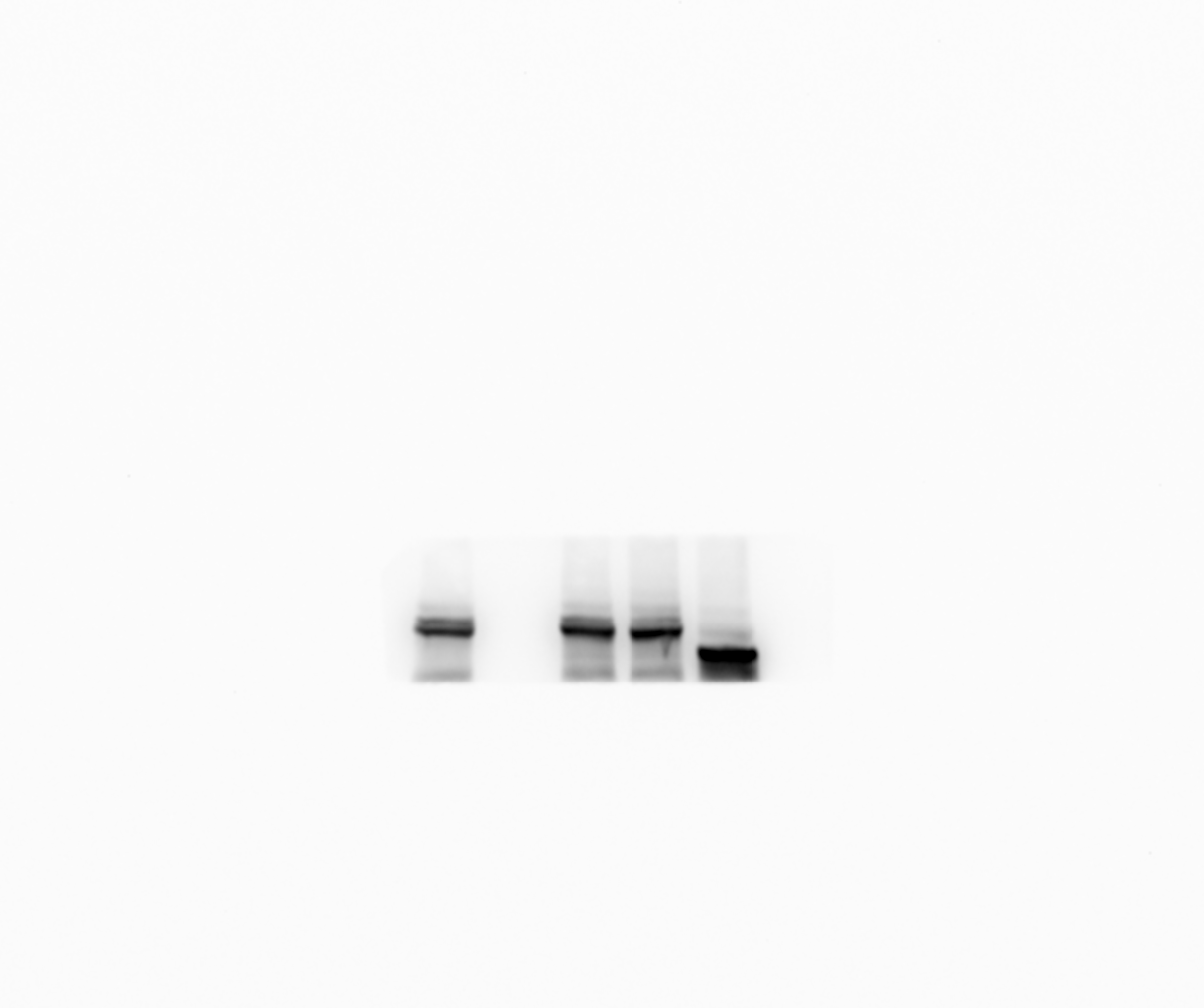

Supplement: Supplementary file 10 — Source data Fig. 7 [file 44319_2024_352_MOESM10_ESM.zip › Figure 7/7A/western TRIM71 Input.tif]

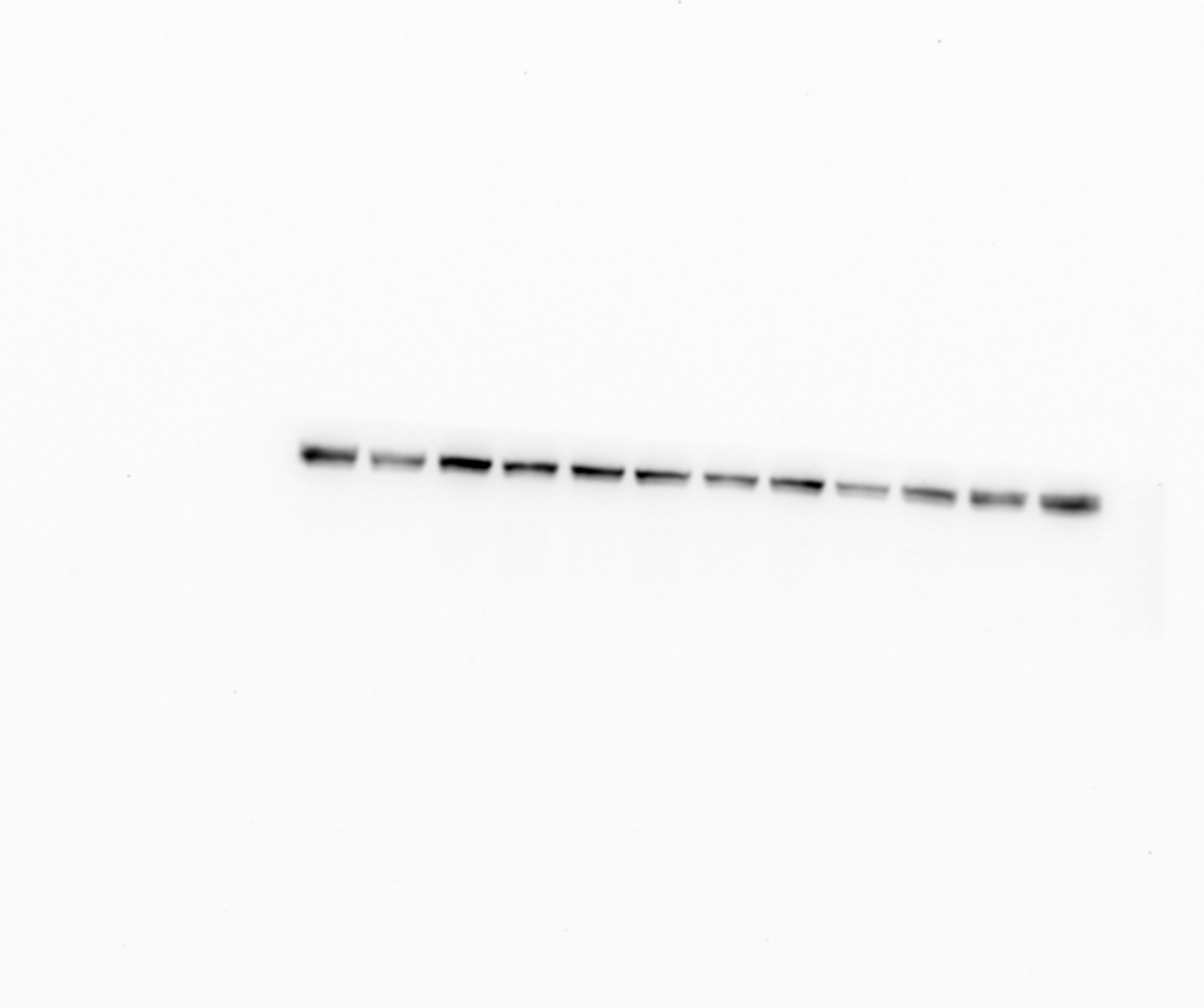

Supplement: Supplementary file 10 — Source data Fig. 7 [file 44319_2024_352_MOESM10_ESM.zip › Figure 7/7C/western Flag Input.tif]

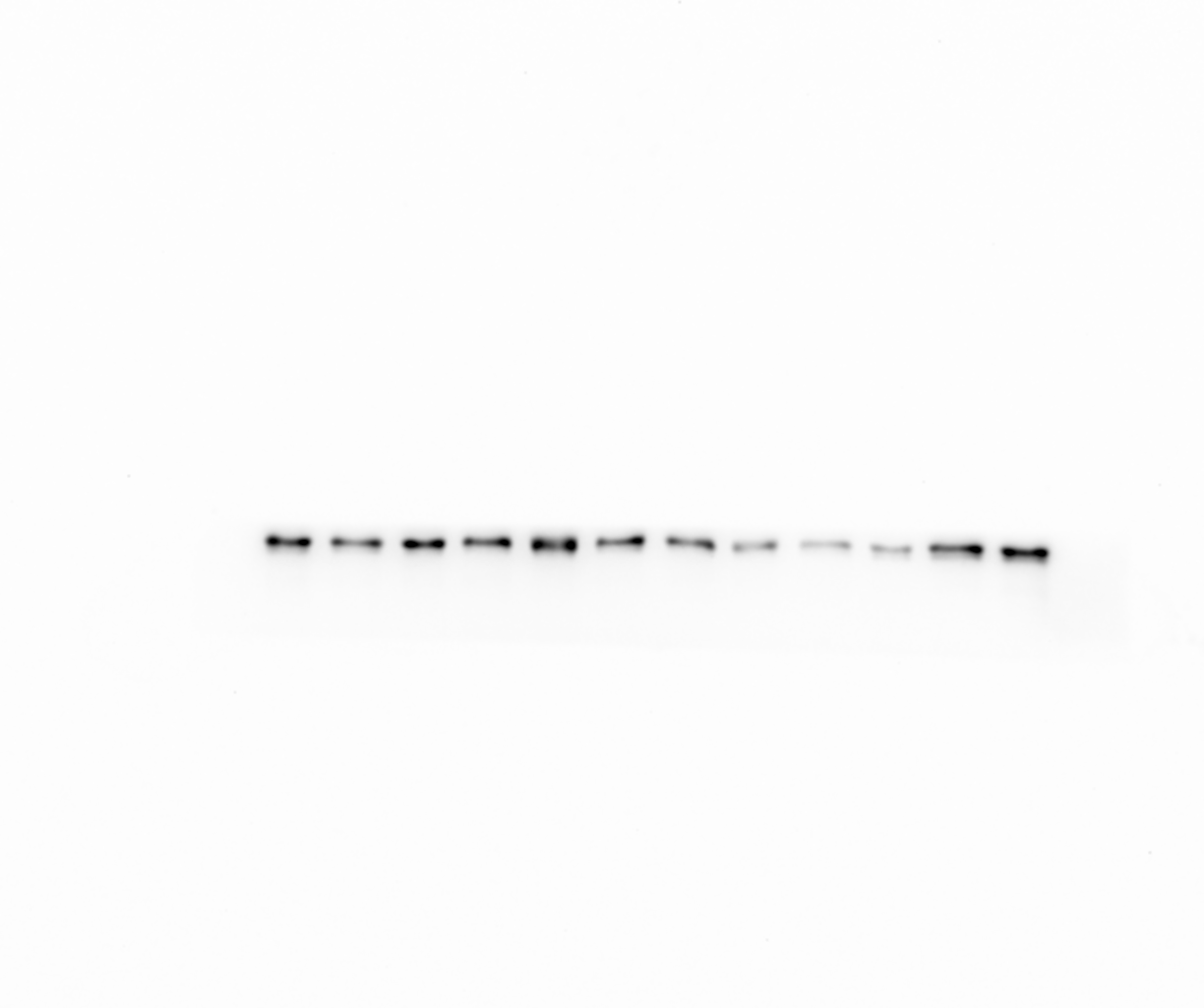

Supplement: Supplementary file 10 — Source data Fig. 7 [file 44319_2024_352_MOESM10_ESM.zip › Figure 7/7C/western Flag IP.tif]

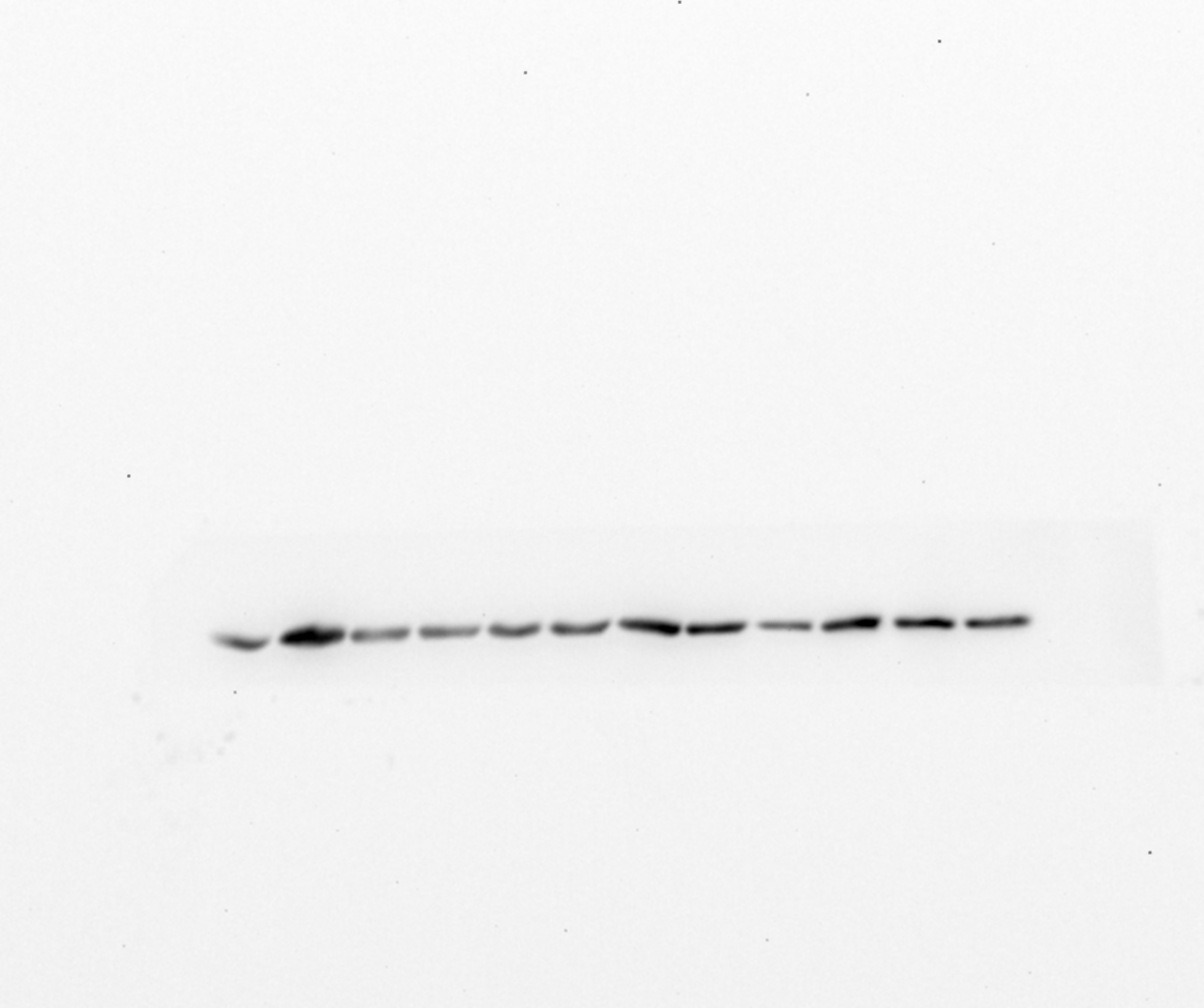

Supplement: Supplementary file 10 — Source data Fig. 7 [file 44319_2024_352_MOESM10_ESM.zip › Figure 7/7C/western GAPDH.tif]

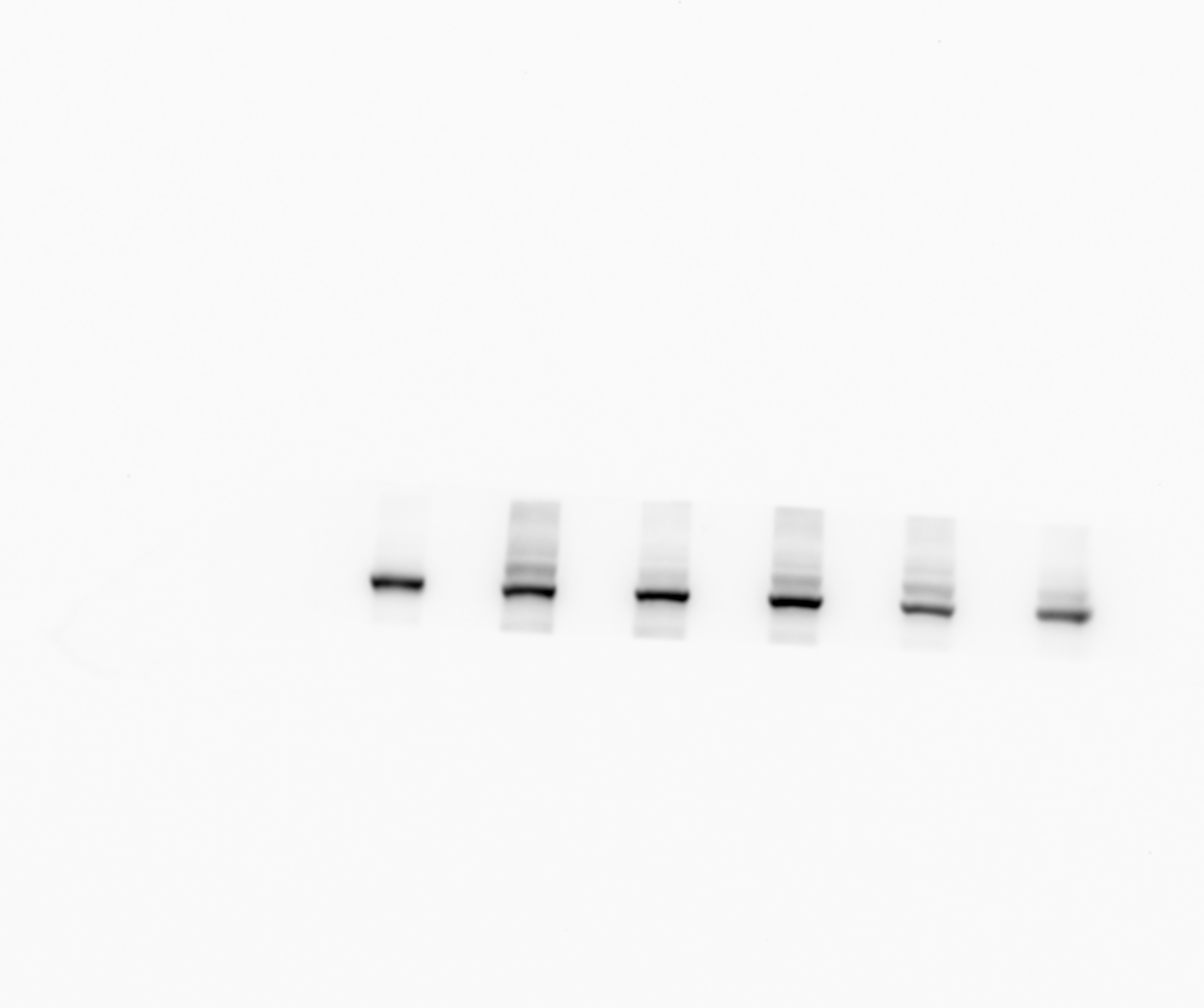

Supplement: Supplementary file 10 — Source data Fig. 7 [file 44319_2024_352_MOESM10_ESM.zip › Figure 7/7C/western GFP Input.tif]

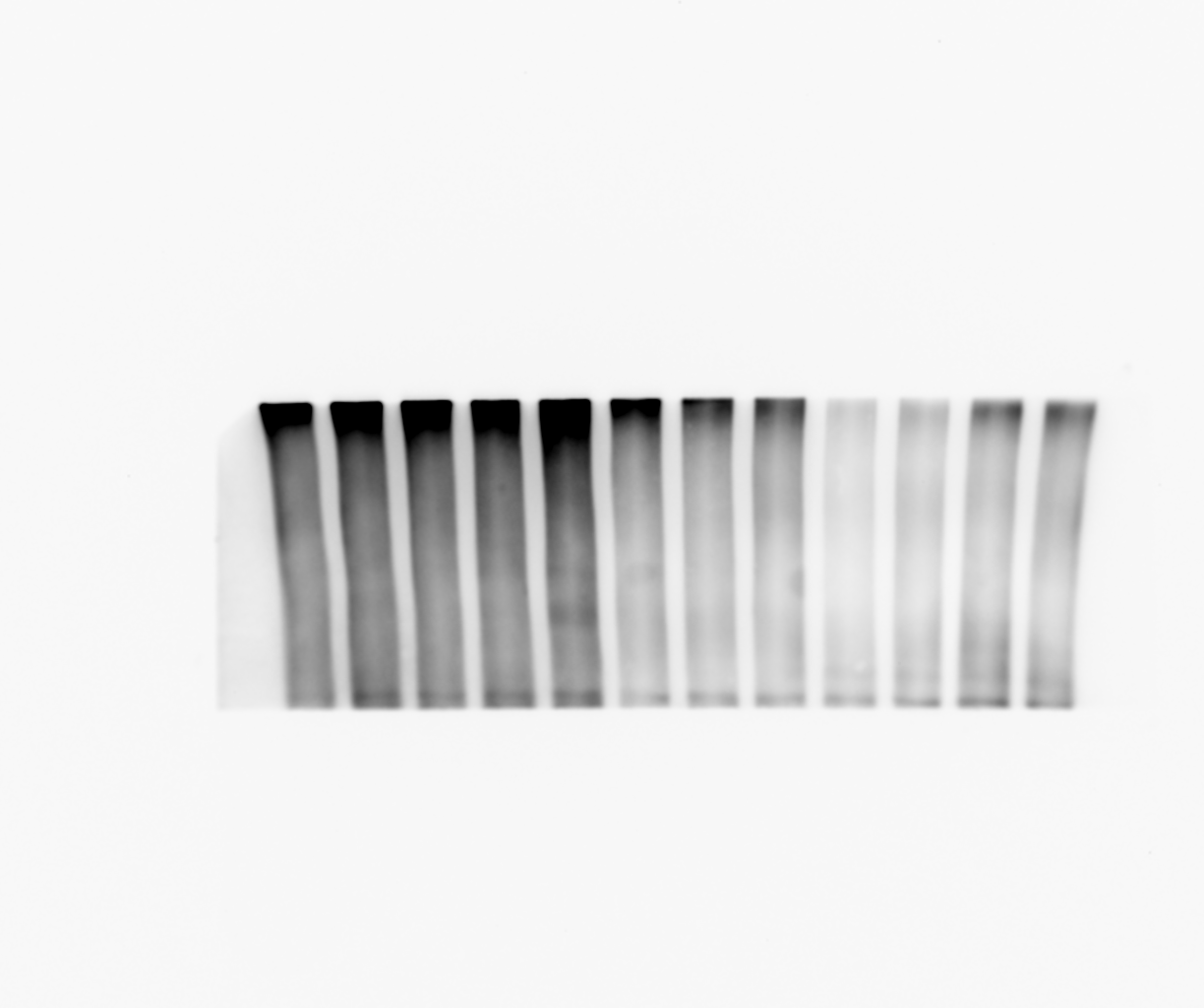

Supplement: Supplementary file 10 — Source data Fig. 7 [file 44319_2024_352_MOESM10_ESM.zip › Figure 7/7C/western HA IP.tif]

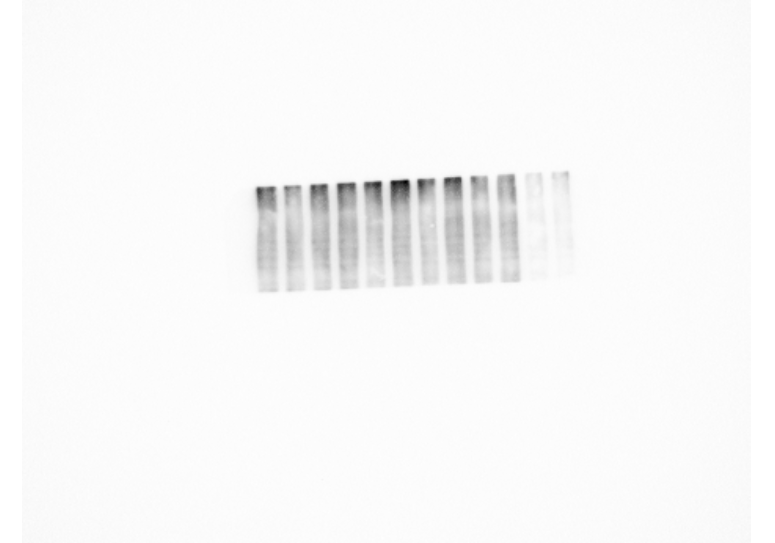

Supplement: Supplementary file 10 — Source data Fig. 7 [file 44319_2024_352_MOESM10_ESM.zip › Figure 7/7C/western HA Input.tif]

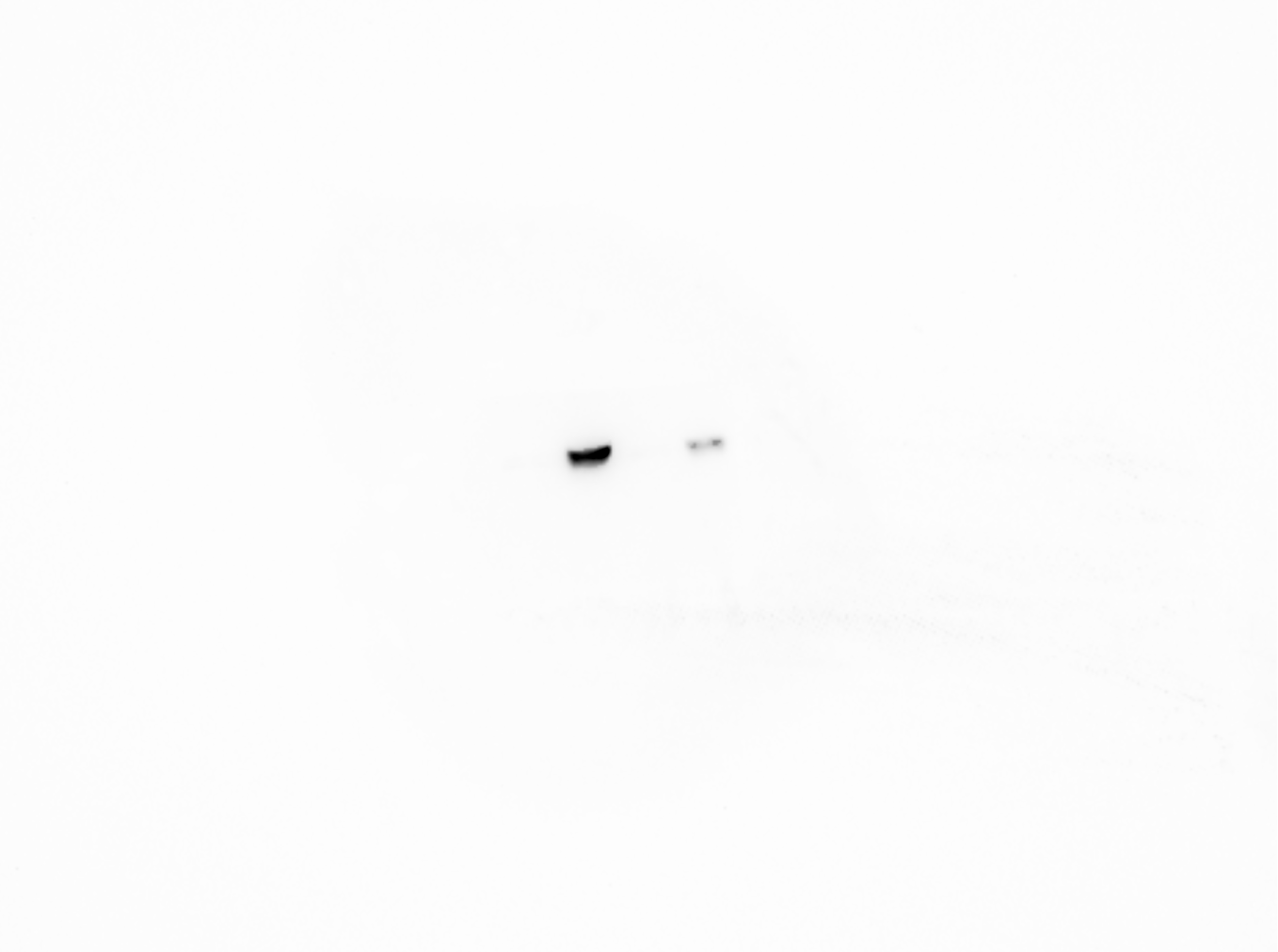

Supplement: Supplementary file 10 — Source data Fig. 7 [file 44319_2024_352_MOESM10_ESM.zip › Figure 7/7E/western p-IRF3.tif]

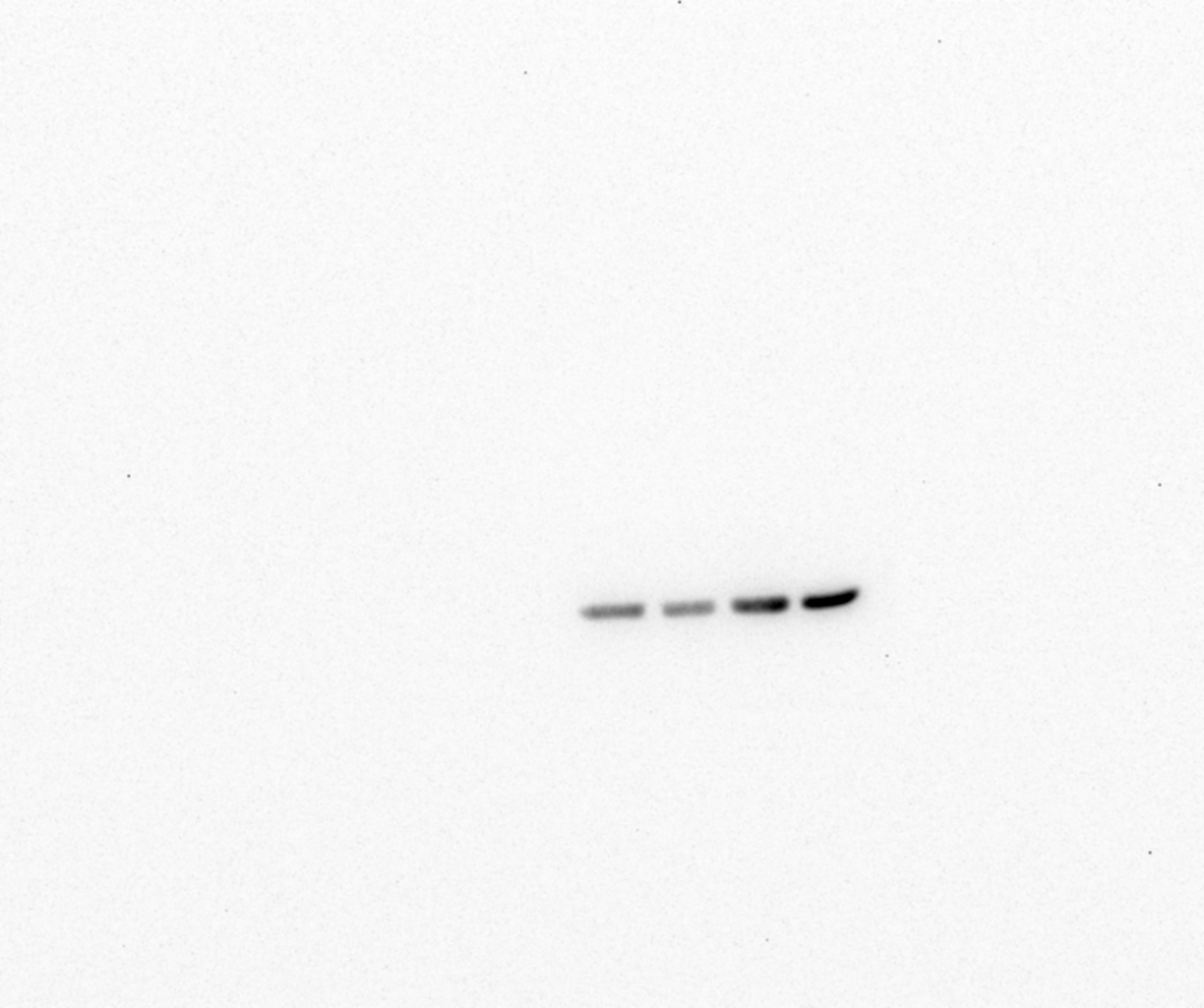

Supplement: Supplementary file 10 — Source data Fig. 7 [file 44319_2024_352_MOESM10_ESM.zip › Figure 7/7E/western GAPDH.tif]

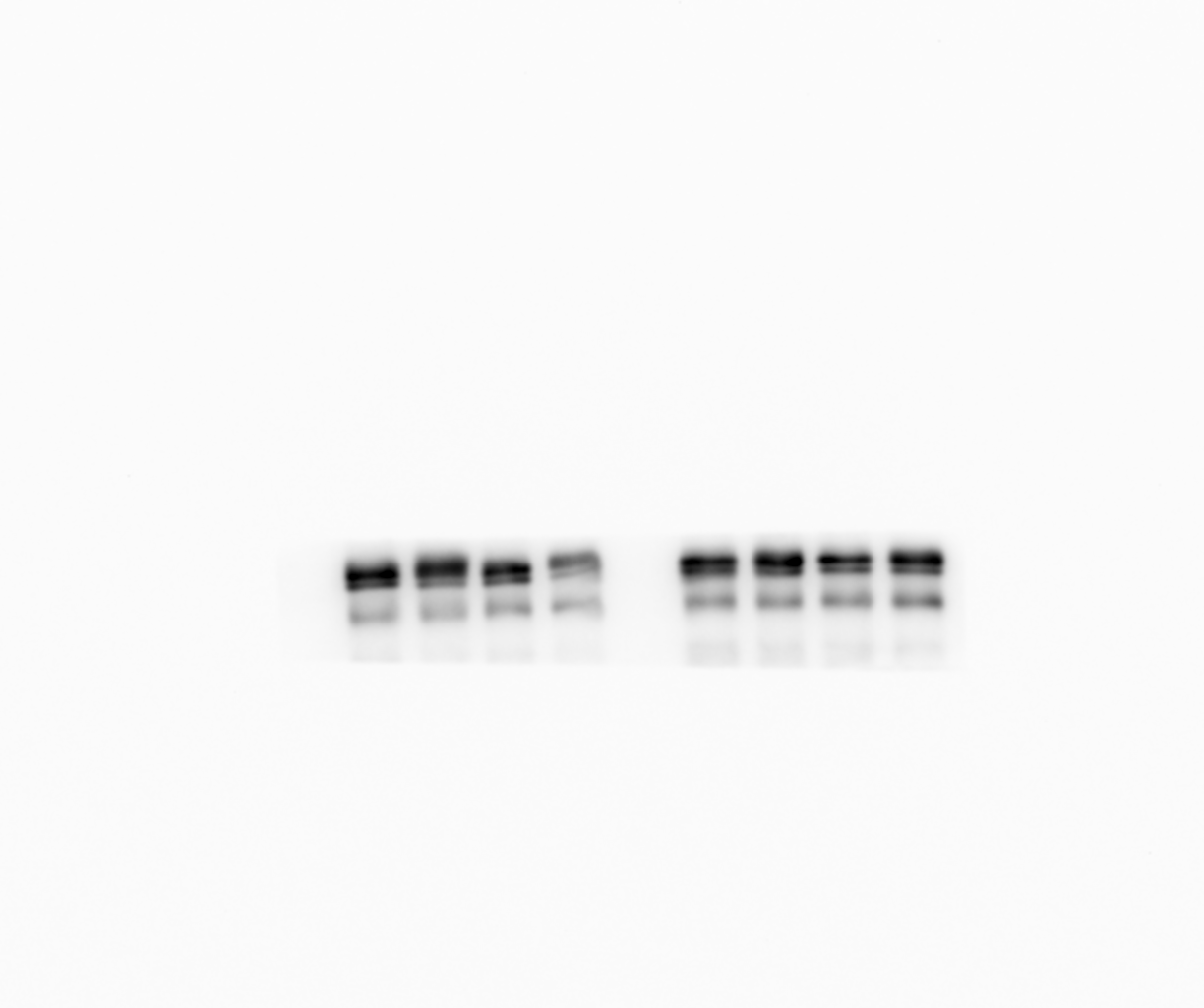

Supplement: Supplementary file 10 — Source data Fig. 7 [file 44319_2024_352_MOESM10_ESM.zip › Figure 7/7E/western IRF3 IP.tif]

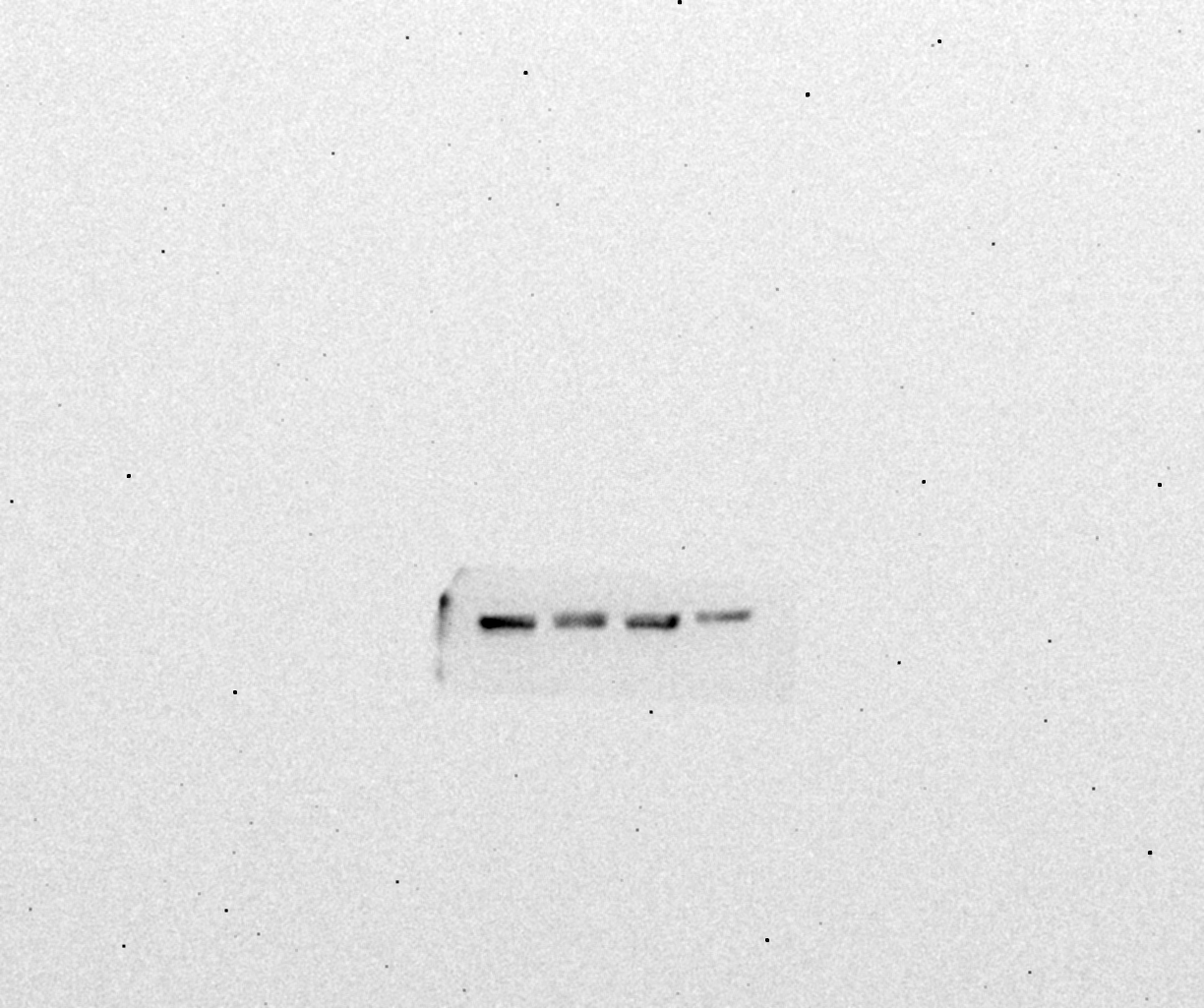

Supplement: Supplementary file 10 — Source data Fig. 7 [file 44319_2024_352_MOESM10_ESM.zip › Figure 7/7E/western IRF3 Input.tif]

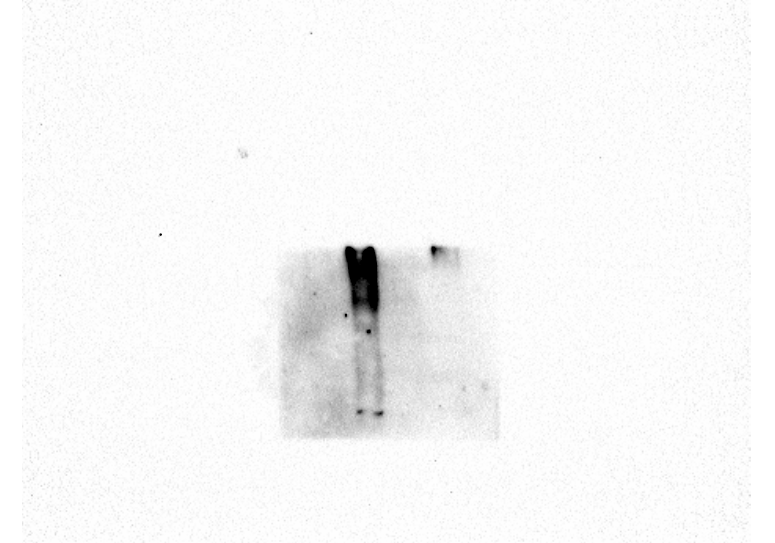

Supplement: Supplementary file 10 — Source data Fig. 7 [file 44319_2024_352_MOESM10_ESM.zip › Figure 7/7E/western Ub IP.tif]

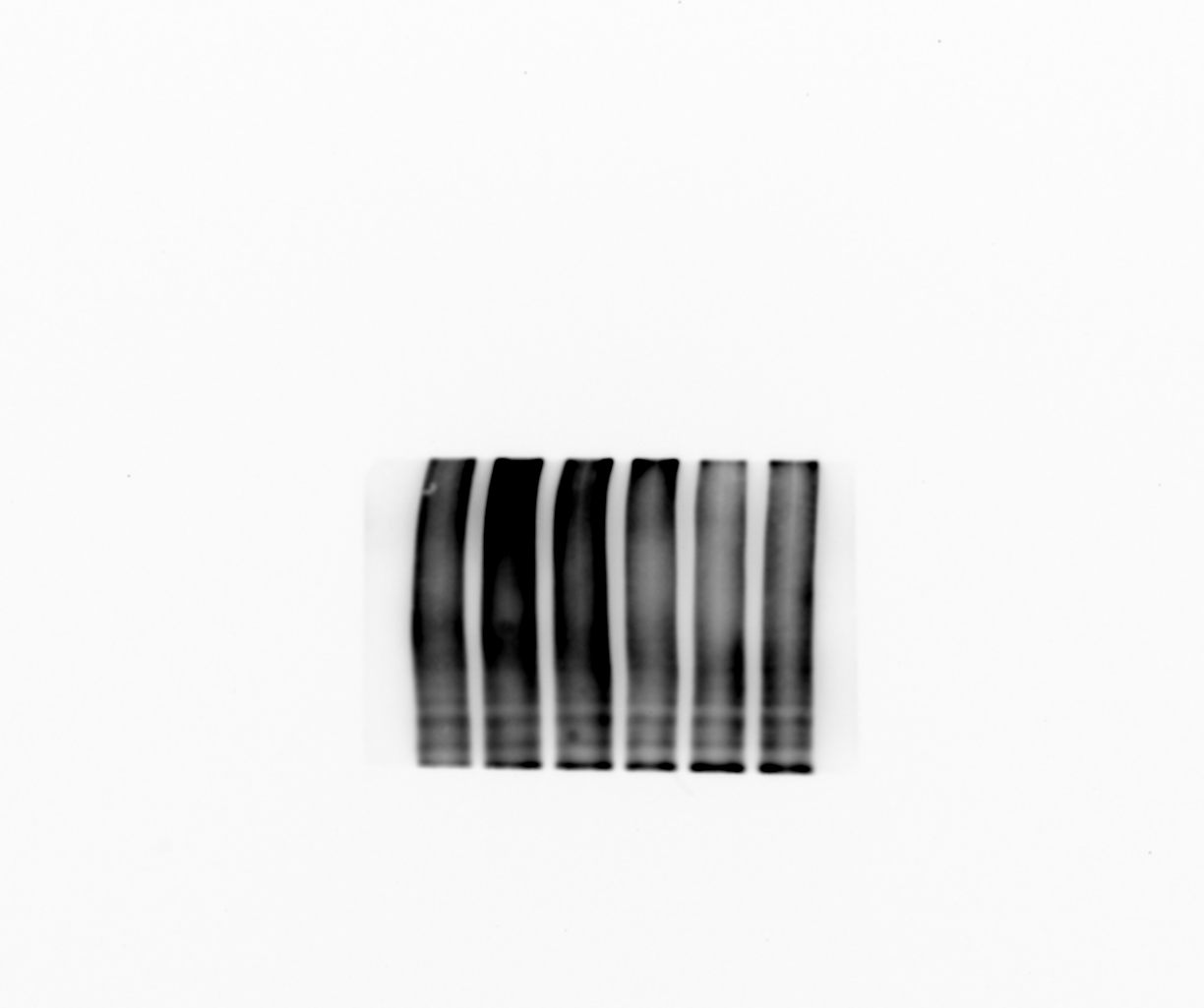

Supplement: Supplementary file 10 — Source data Fig. 7 [file 44319_2024_352_MOESM10_ESM.zip › Figure 7/7F/western HA IP.tif]

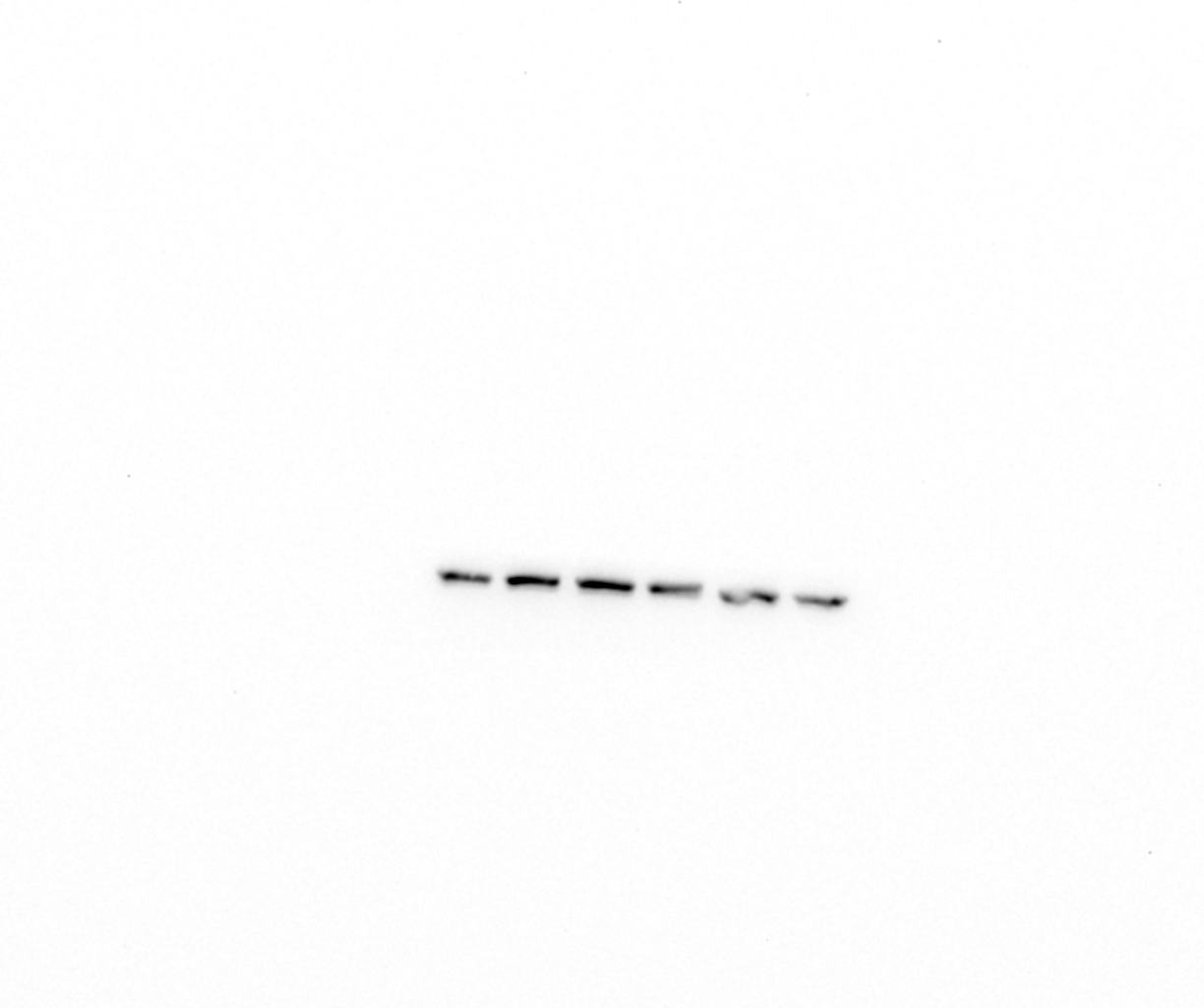

Supplement: Supplementary file 10 — Source data Fig. 7 [file 44319_2024_352_MOESM10_ESM.zip › Figure 7/7F/western Flag Input.tif]
