# Supplementary figures and images for: DYRK4 upregulates antiviral innate immunity by promoting IRF3 activation (part 3 of 3)
Source: EMBO Rep. 2024 Dec 19;26(3):690–719. doi: 10.1038/s44319-024-00352-x (PMC11811199; doi:10.1038/s44319-024-00352-x)

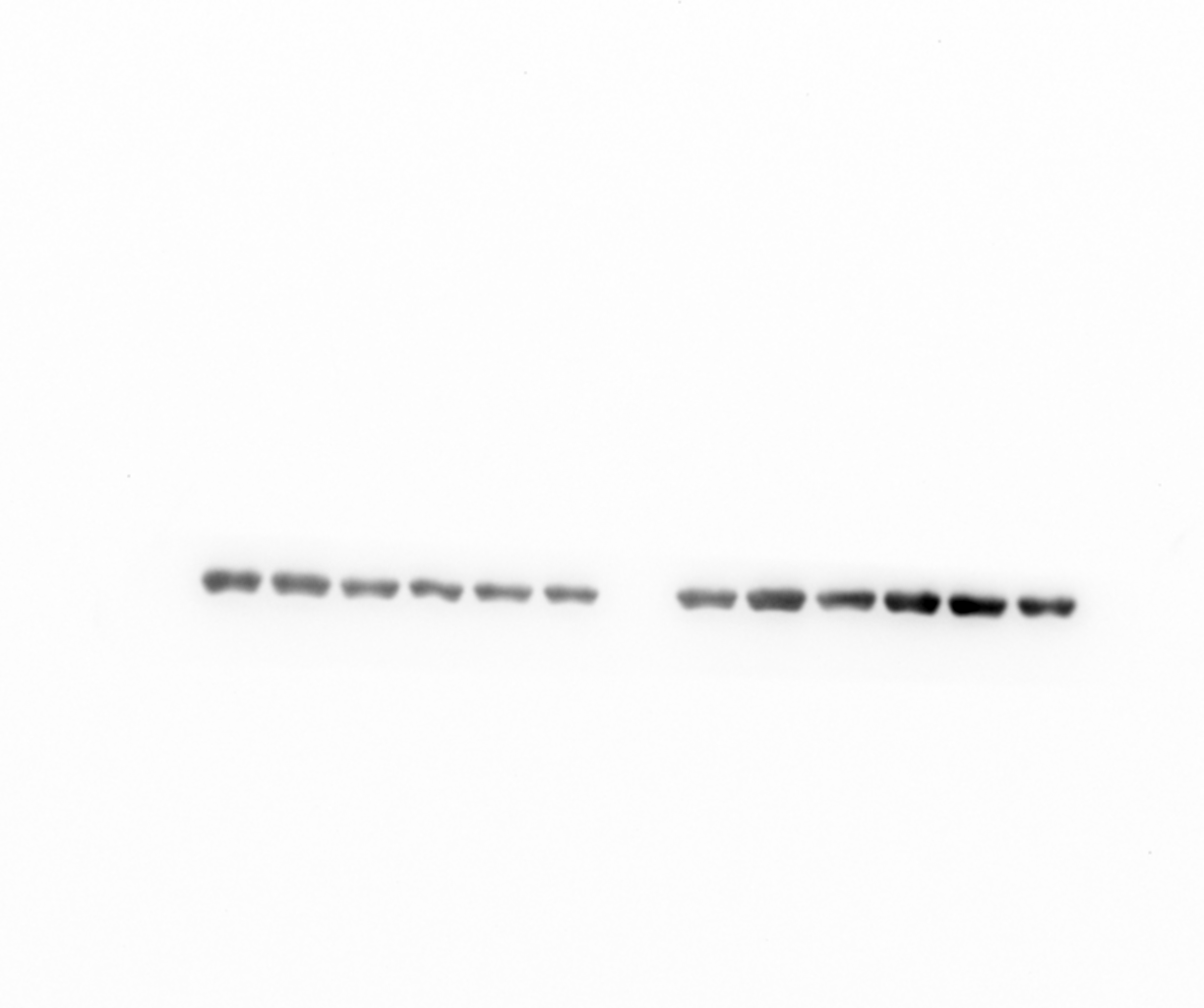

Supplement: Supplementary file 10 — Source data Fig. 7 [file 44319_2024_352_MOESM10_ESM.zip › Figure 7/7F/western Flag IP.tif]

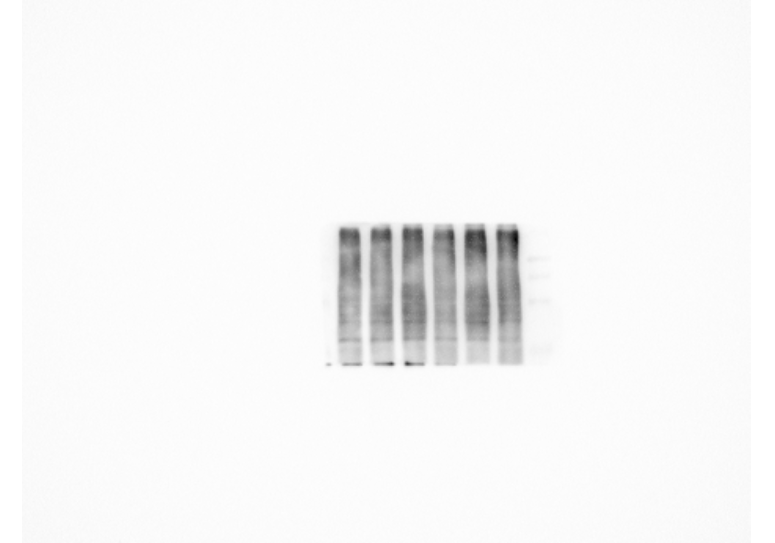

Supplement: Supplementary file 10 — Source data Fig. 7 [file 44319_2024_352_MOESM10_ESM.zip › Figure 7/7F/western HA Input.tif]

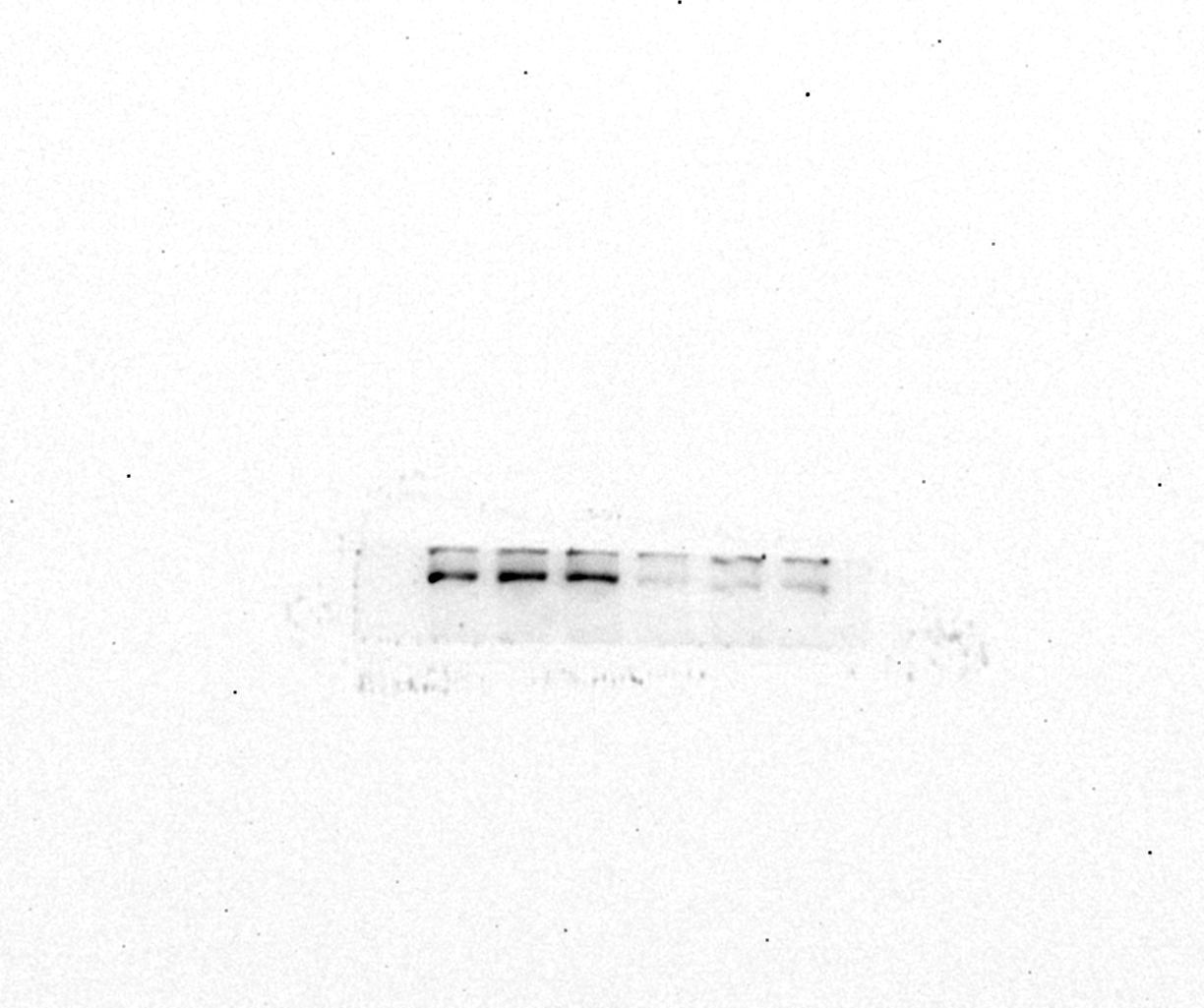

Supplement: Supplementary file 10 — Source data Fig. 7 [file 44319_2024_352_MOESM10_ESM.zip › Figure 7/7F/western TRIM71.tif]

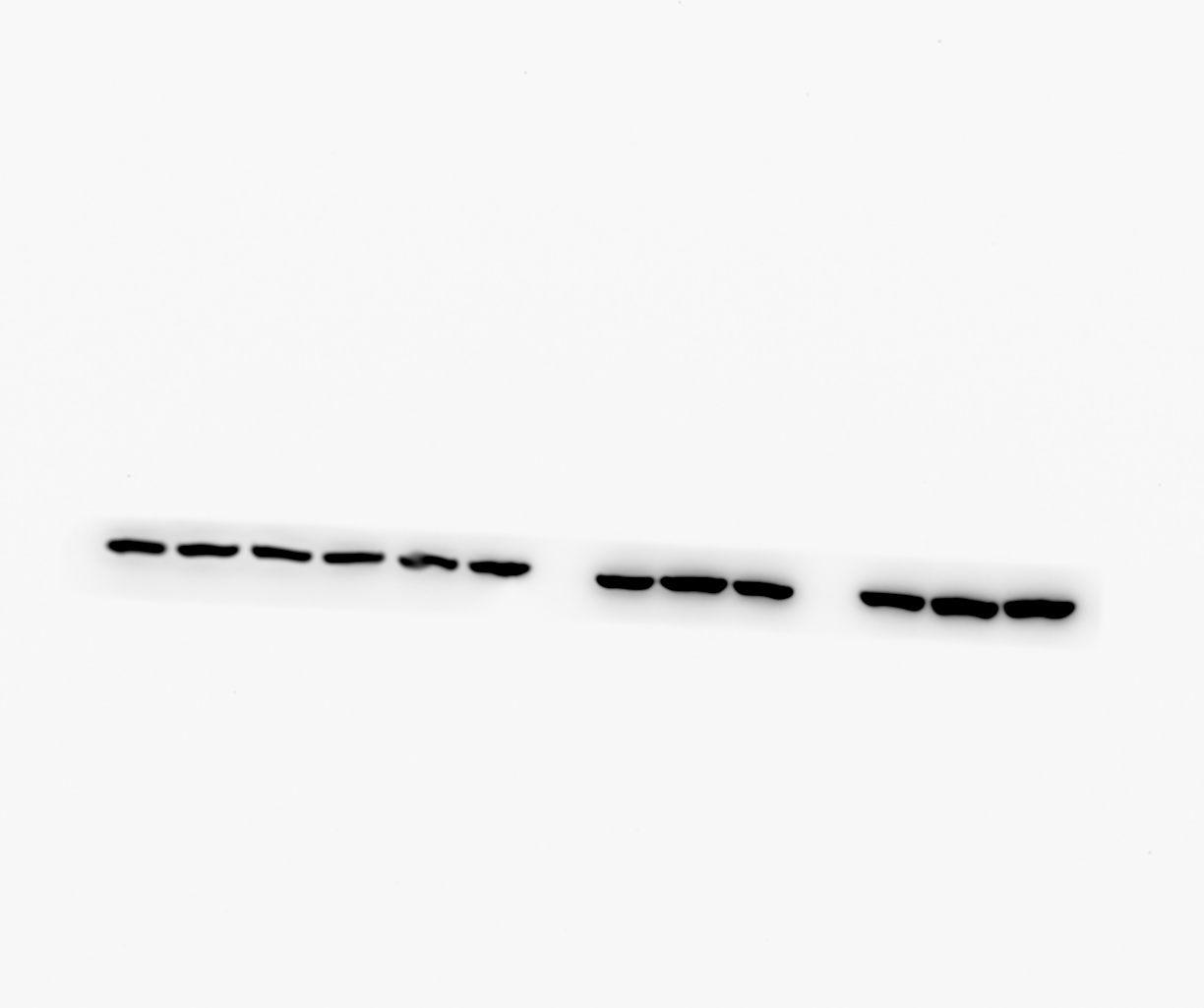

Supplement: Supplementary file 10 — Source data Fig. 7 [file 44319_2024_352_MOESM10_ESM.zip › Figure 7/7F/western actin.tif]

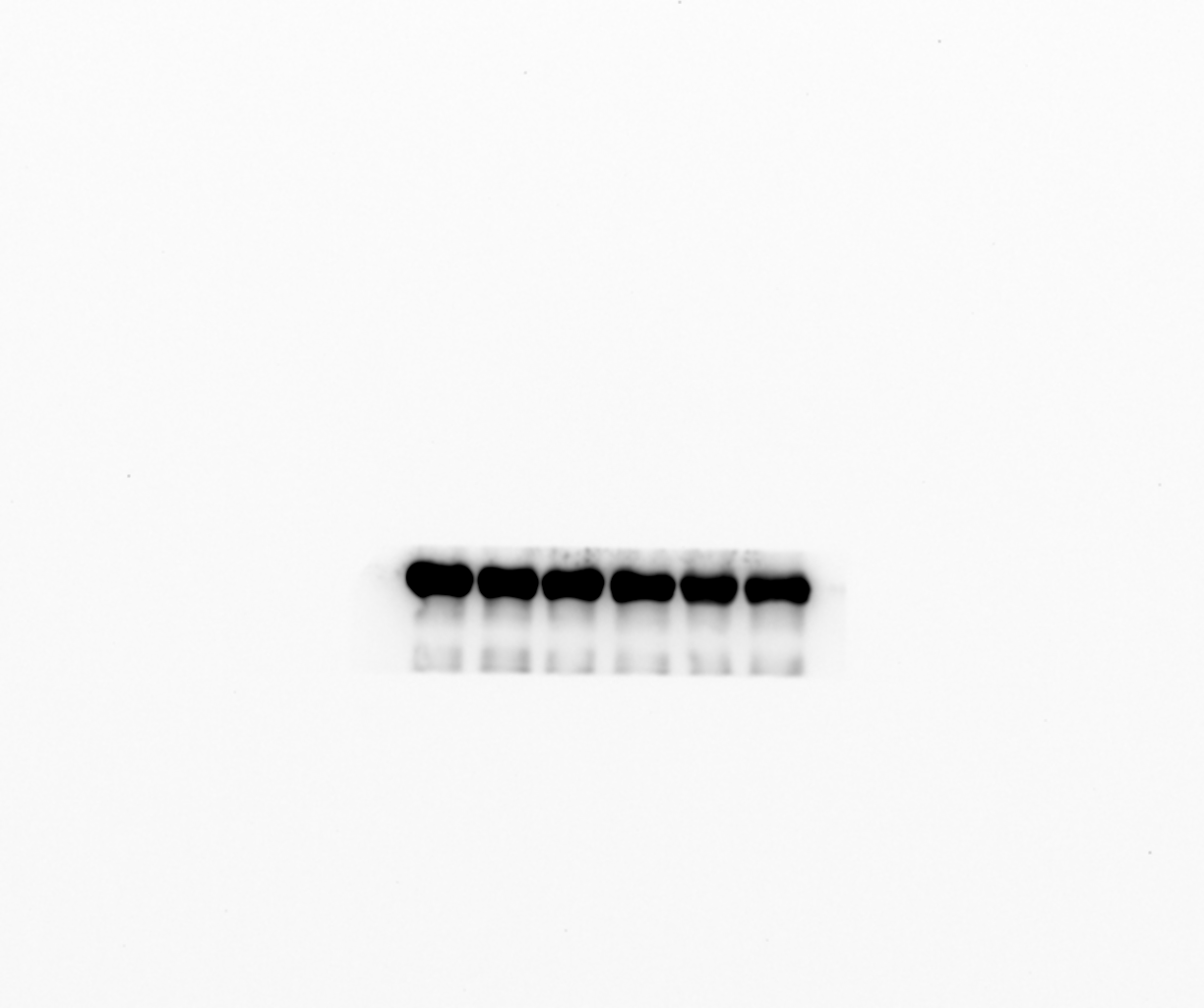

Supplement: Supplementary file 10 — Source data Fig. 7 [file 44319_2024_352_MOESM10_ESM.zip › Figure 7/7G/western Flag IP.tif]

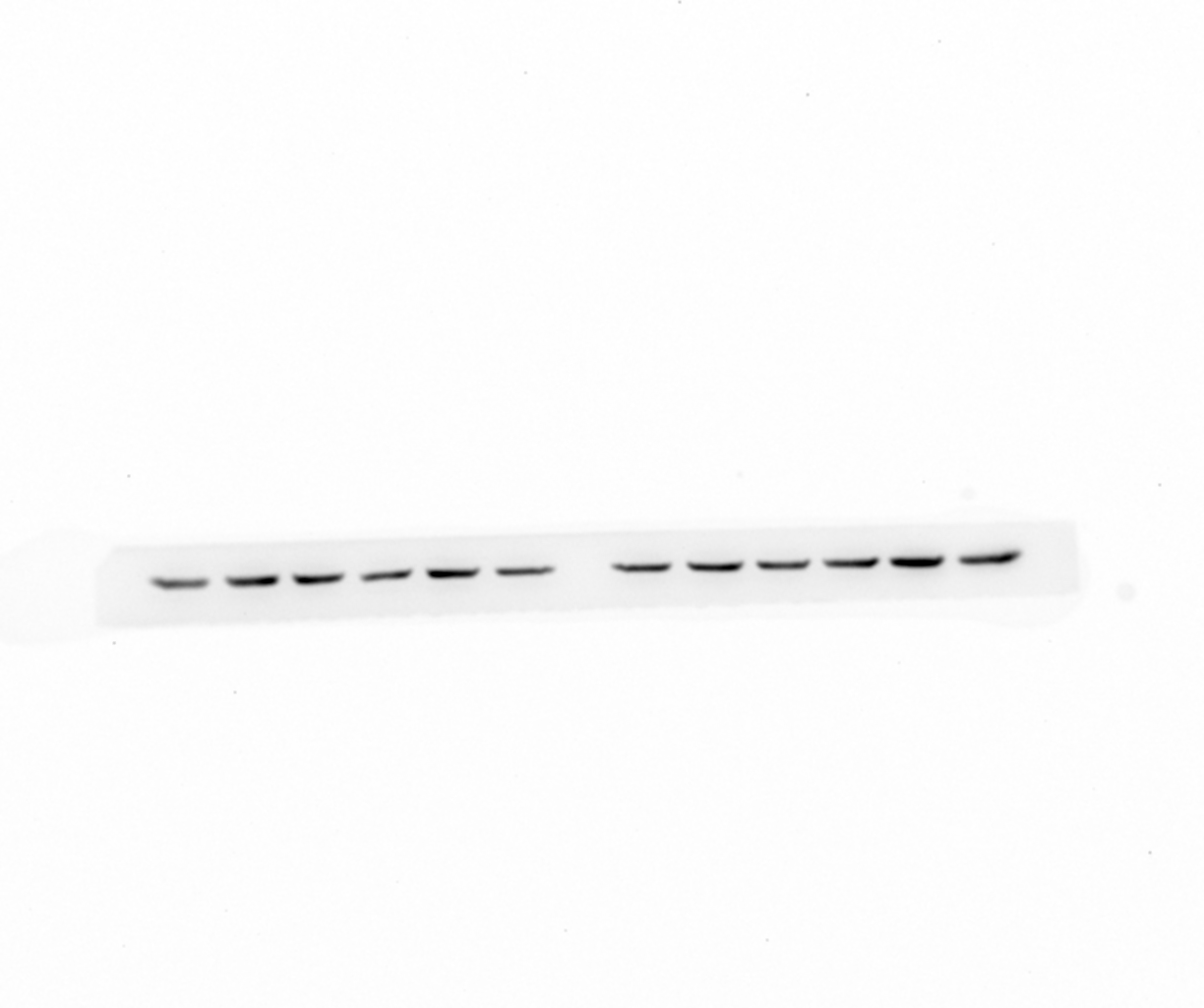

Supplement: Supplementary file 10 — Source data Fig. 7 [file 44319_2024_352_MOESM10_ESM.zip › Figure 7/7G/western Flag Input.tif]

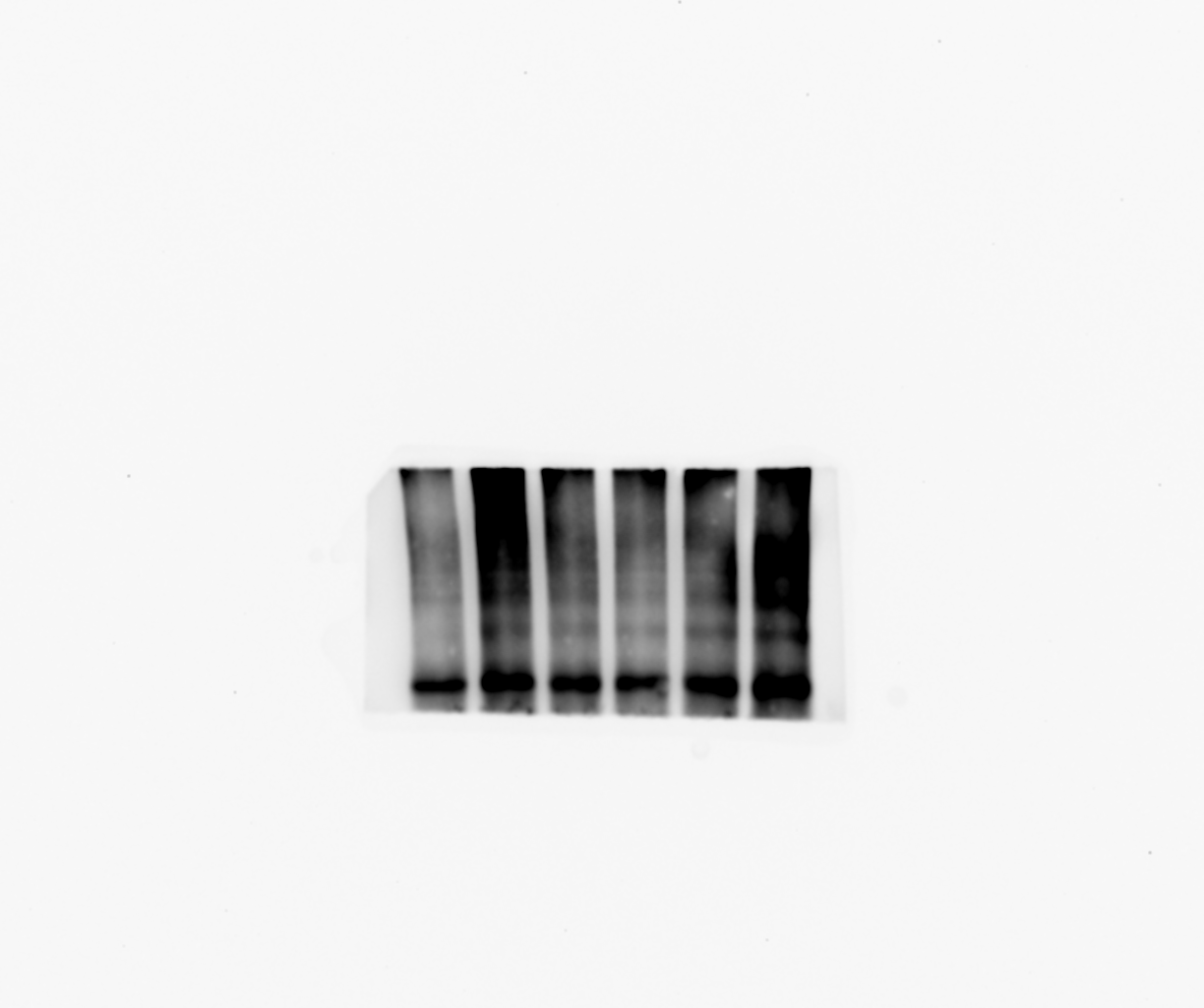

Supplement: Supplementary file 10 — Source data Fig. 7 [file 44319_2024_352_MOESM10_ESM.zip › Figure 7/7G/western HA IP.tif]

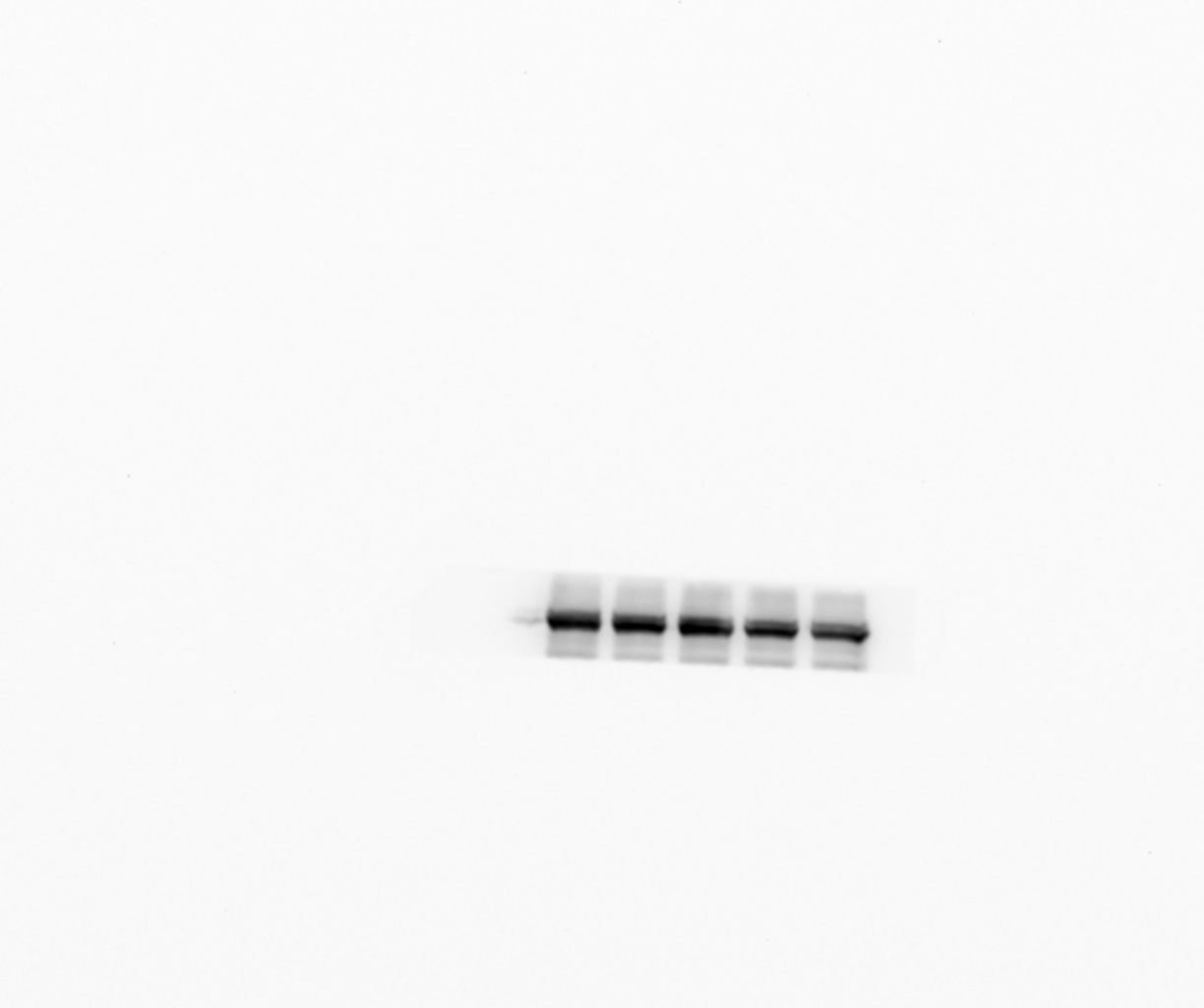

Supplement: Supplementary file 10 — Source data Fig. 7 [file 44319_2024_352_MOESM10_ESM.zip › Figure 7/7G/western TRIM71 Input.tif]

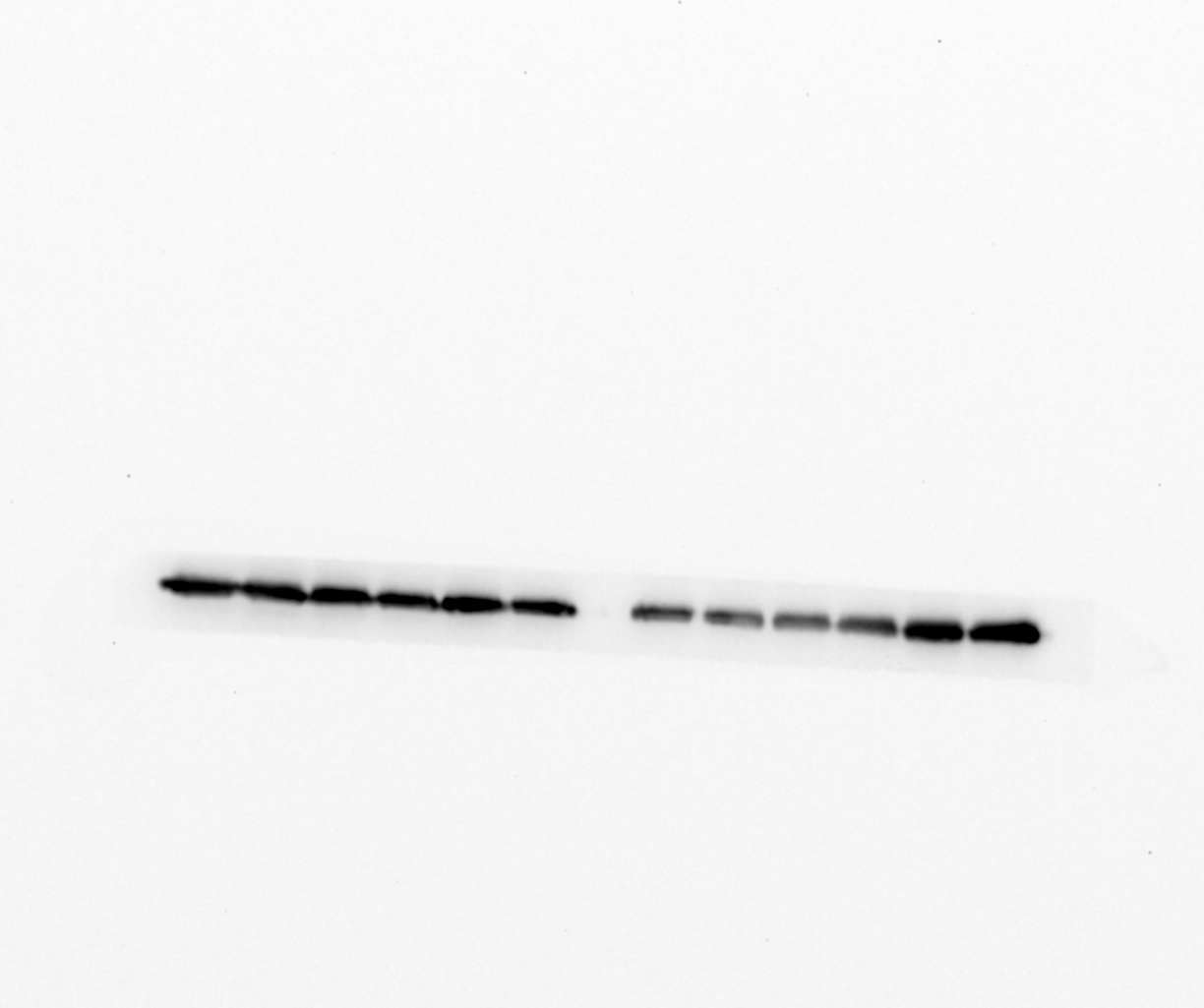

Supplement: Supplementary file 10 — Source data Fig. 7 [file 44319_2024_352_MOESM10_ESM.zip › Figure 7/7G/western actin.tif]

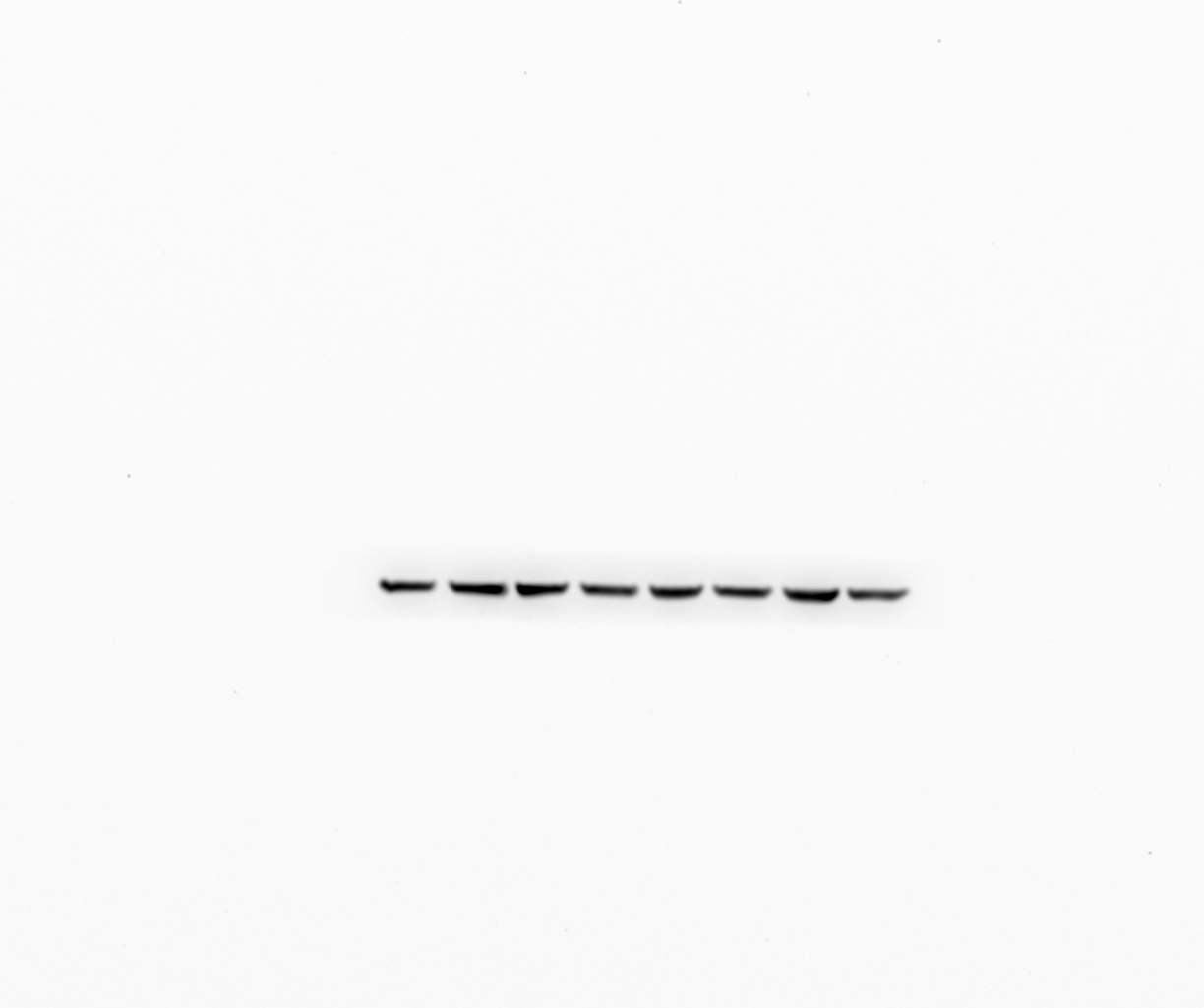

Supplement: Supplementary file 10 — Source data Fig. 7 [file 44319_2024_352_MOESM10_ESM.zip › Figure 7/7J/western ACTIN.tif]

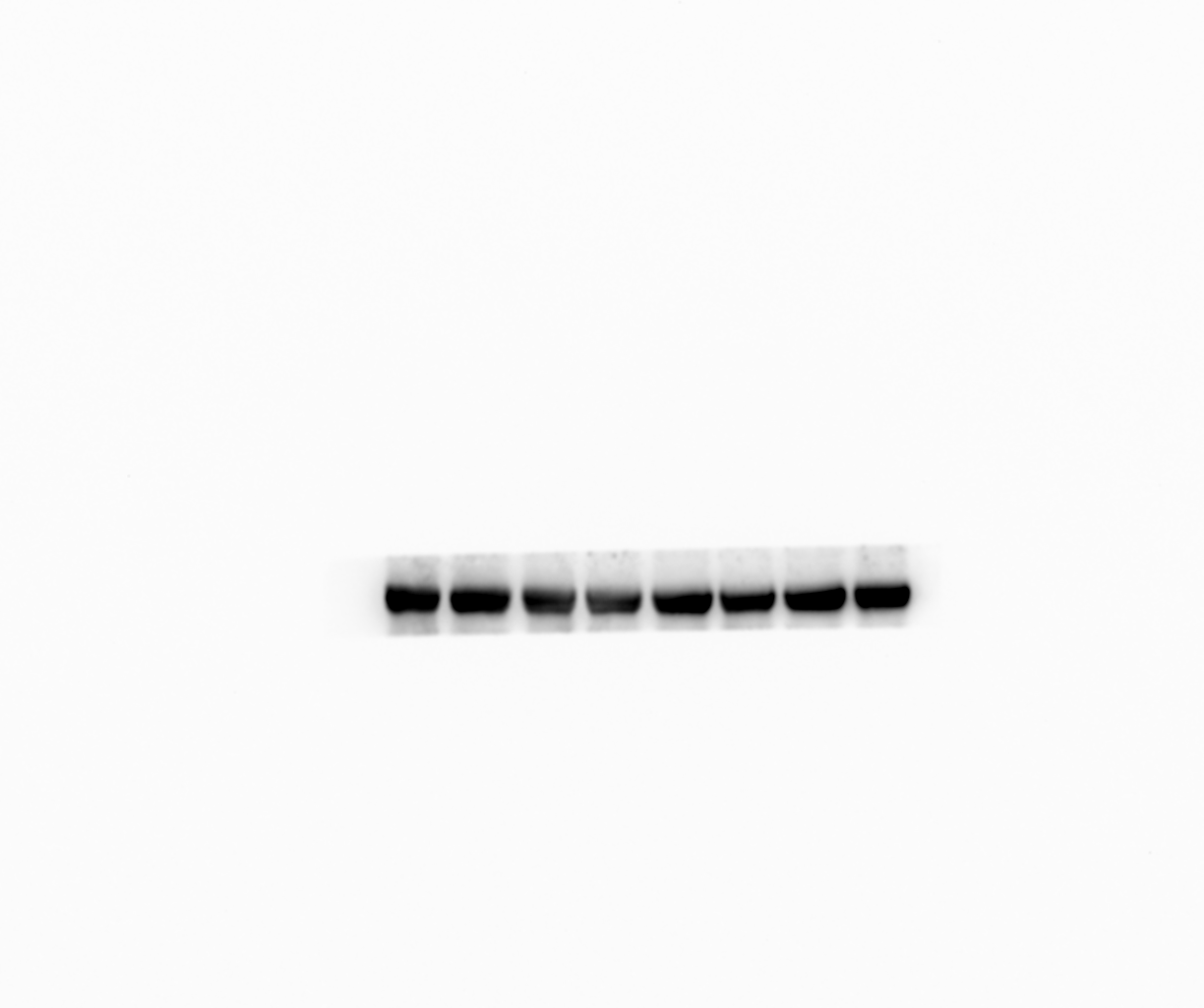

Supplement: Supplementary file 10 — Source data Fig. 7 [file 44319_2024_352_MOESM10_ESM.zip › Figure 7/7J/western IRF3.tif]

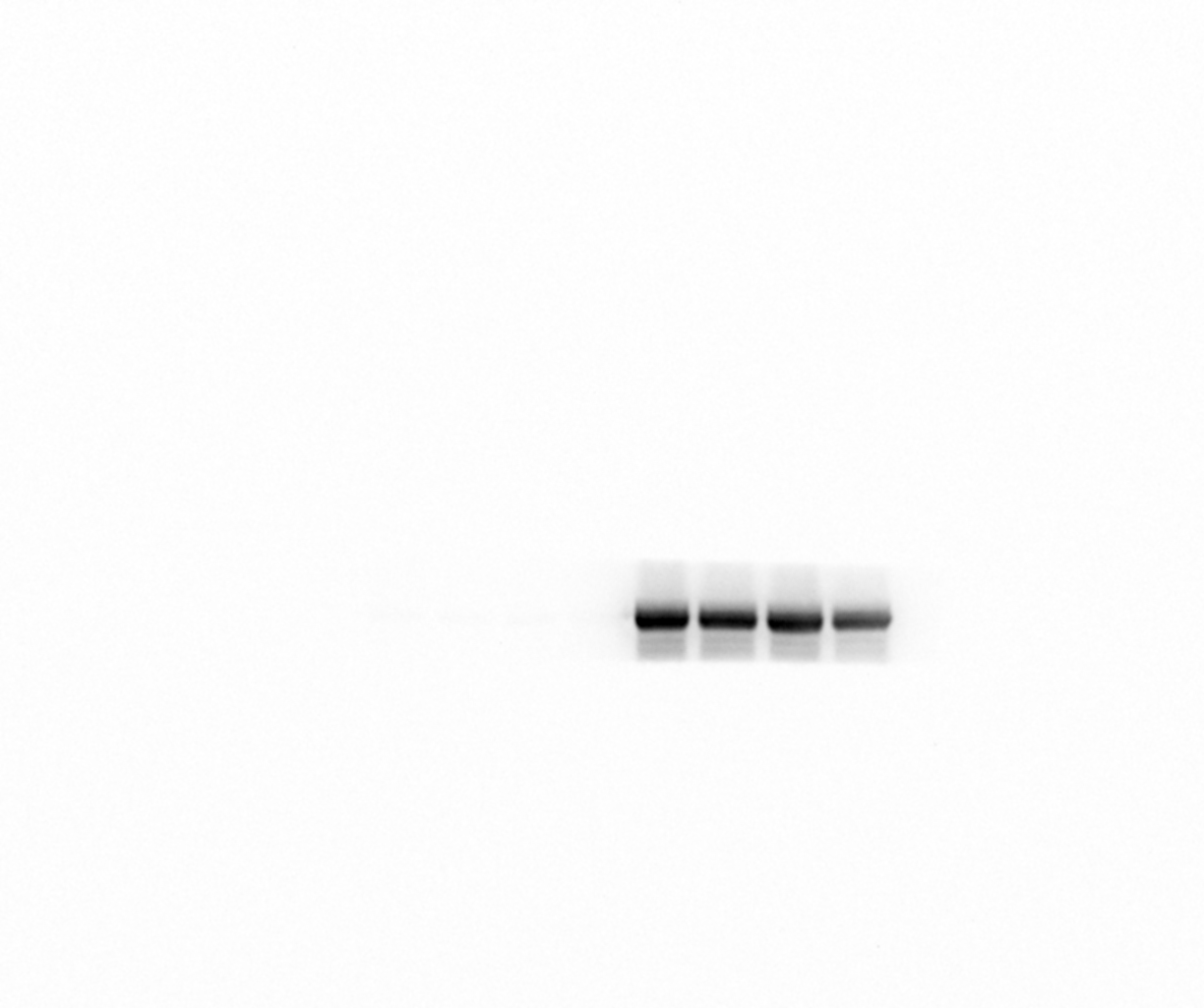

Supplement: Supplementary file 10 — Source data Fig. 7 [file 44319_2024_352_MOESM10_ESM.zip › Figure 7/7J/western TRIM71.tif]

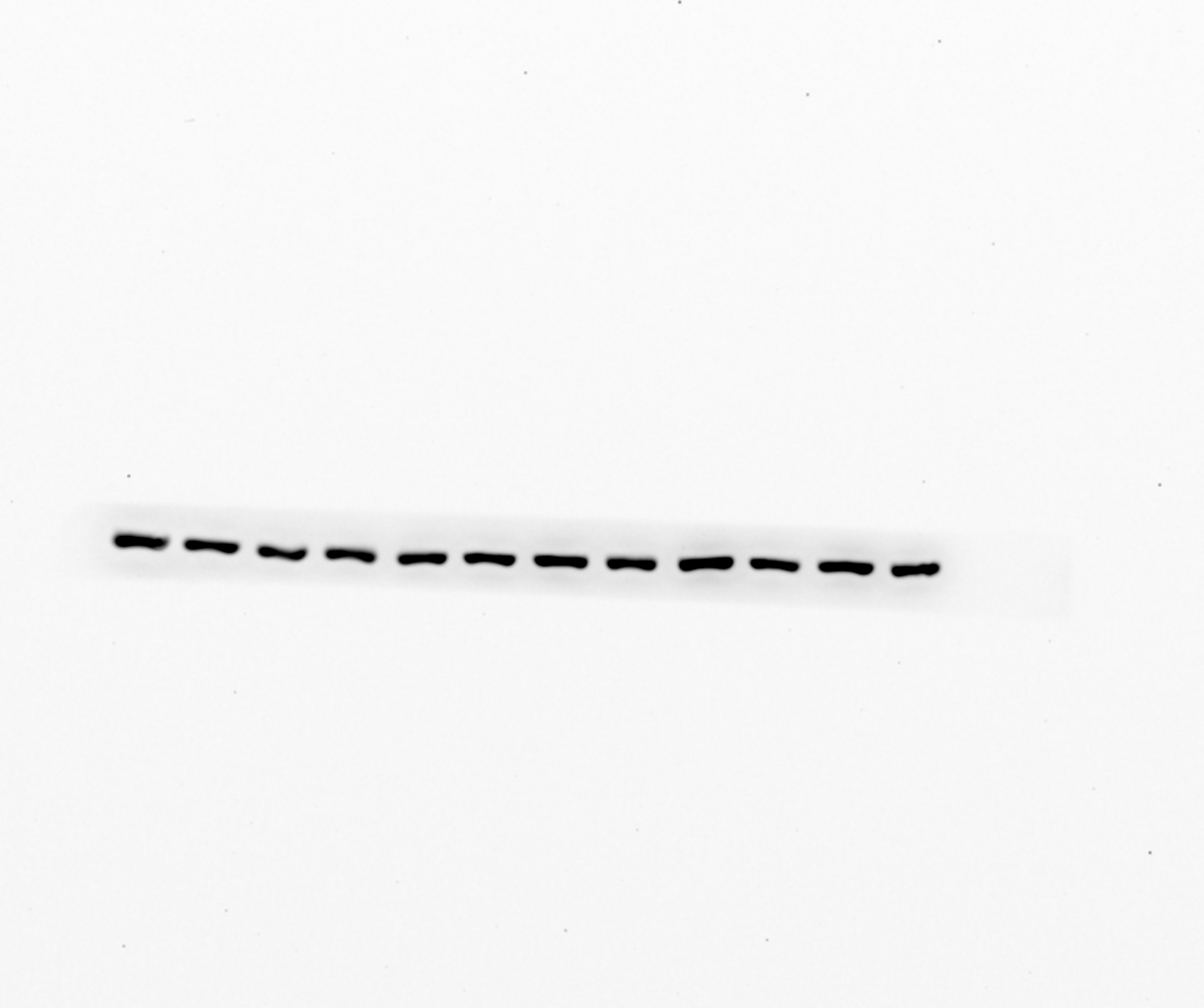

Supplement: Supplementary file 10 — Source data Fig. 7 [file 44319_2024_352_MOESM10_ESM.zip › Figure 7/7K/western ACTIN.tif]

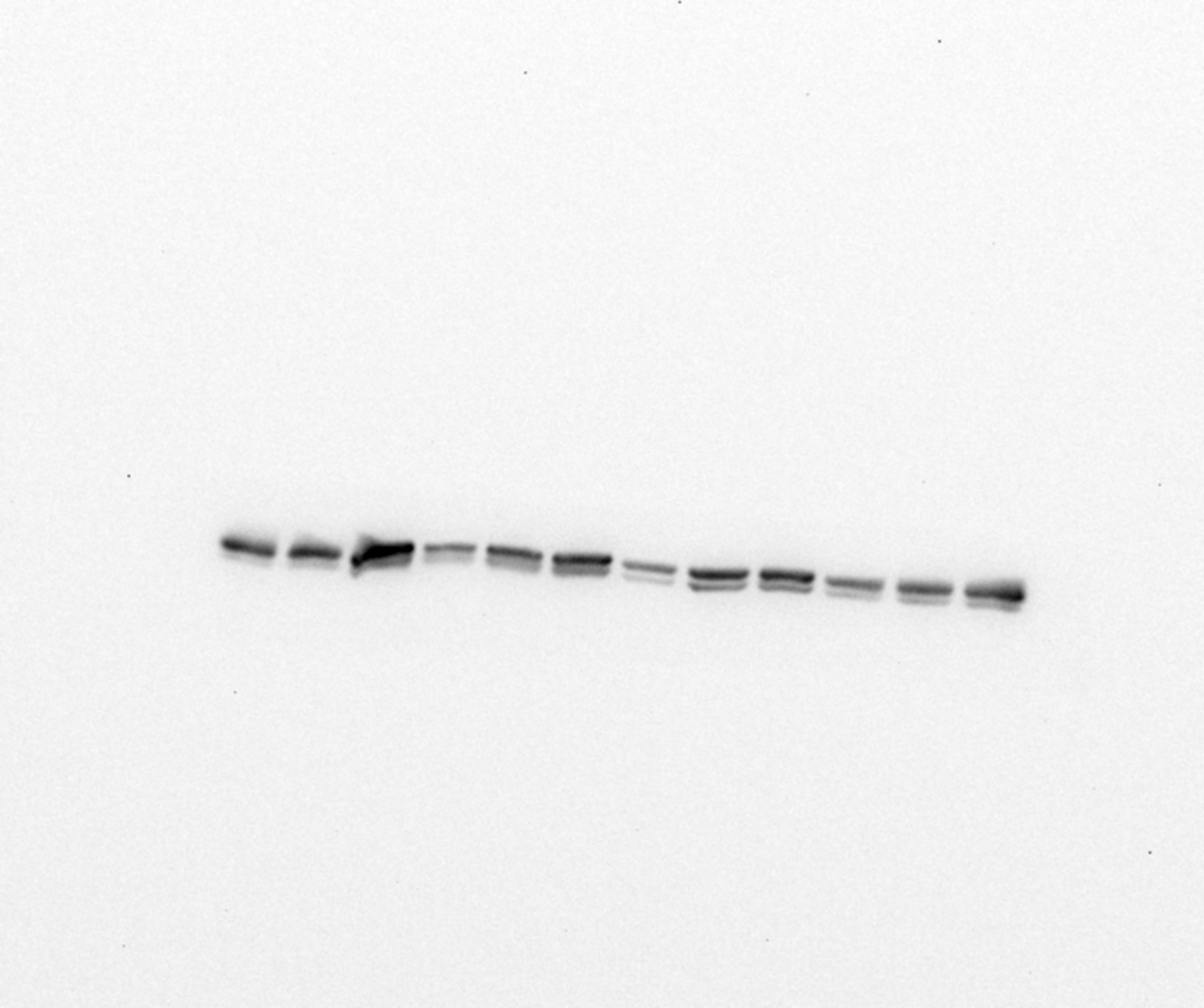

Supplement: Supplementary file 10 — Source data Fig. 7 [file 44319_2024_352_MOESM10_ESM.zip › Figure 7/7K/western Flag.tif]

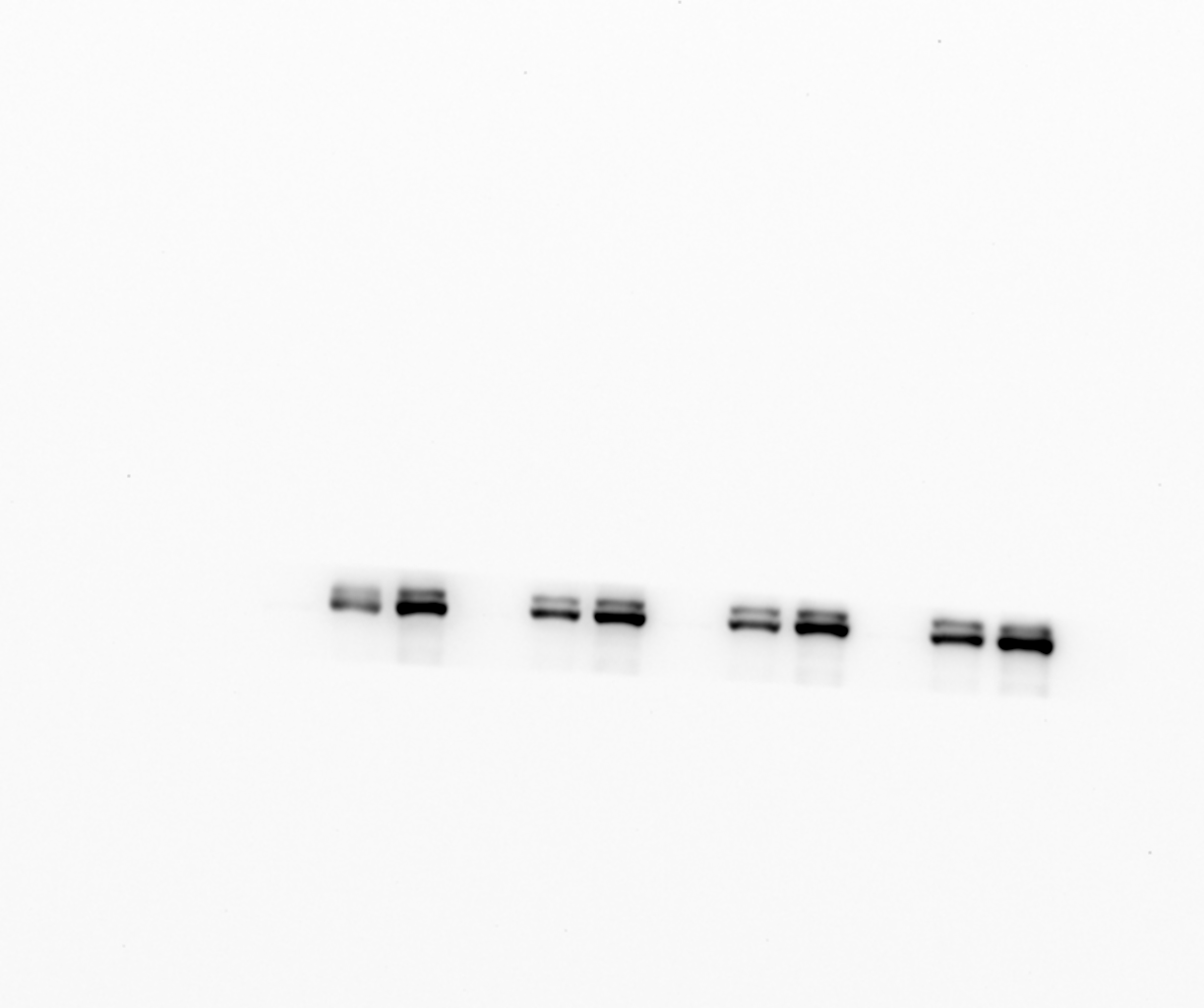

Supplement: Supplementary file 10 — Source data Fig. 7 [file 44319_2024_352_MOESM10_ESM.zip › Figure 7/7K/western TRIM71.tif]

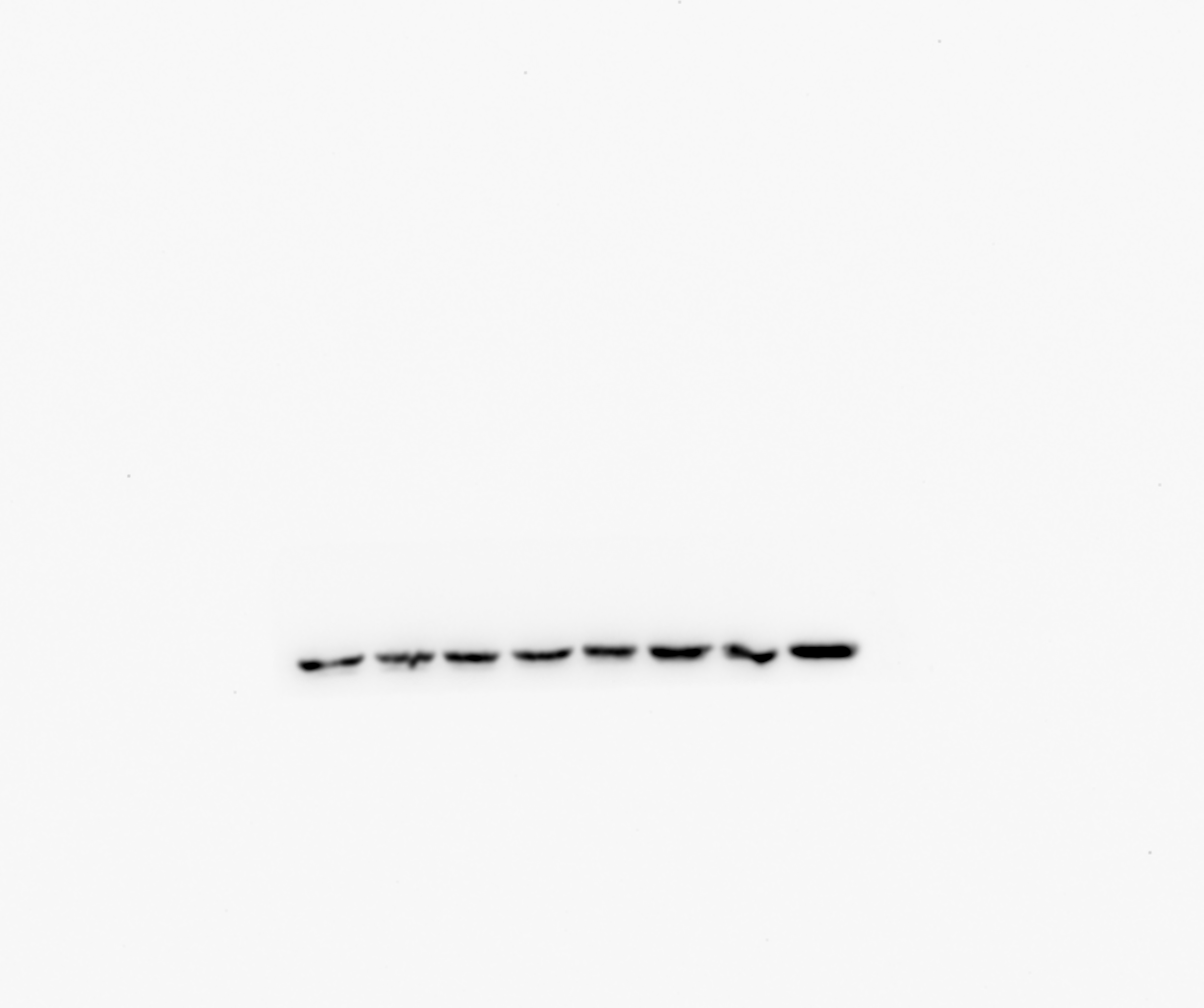

Supplement: Supplementary file 10 — Source data Fig. 7 [file 44319_2024_352_MOESM10_ESM.zip › Figure 7/7L/western GAPDH.tif]

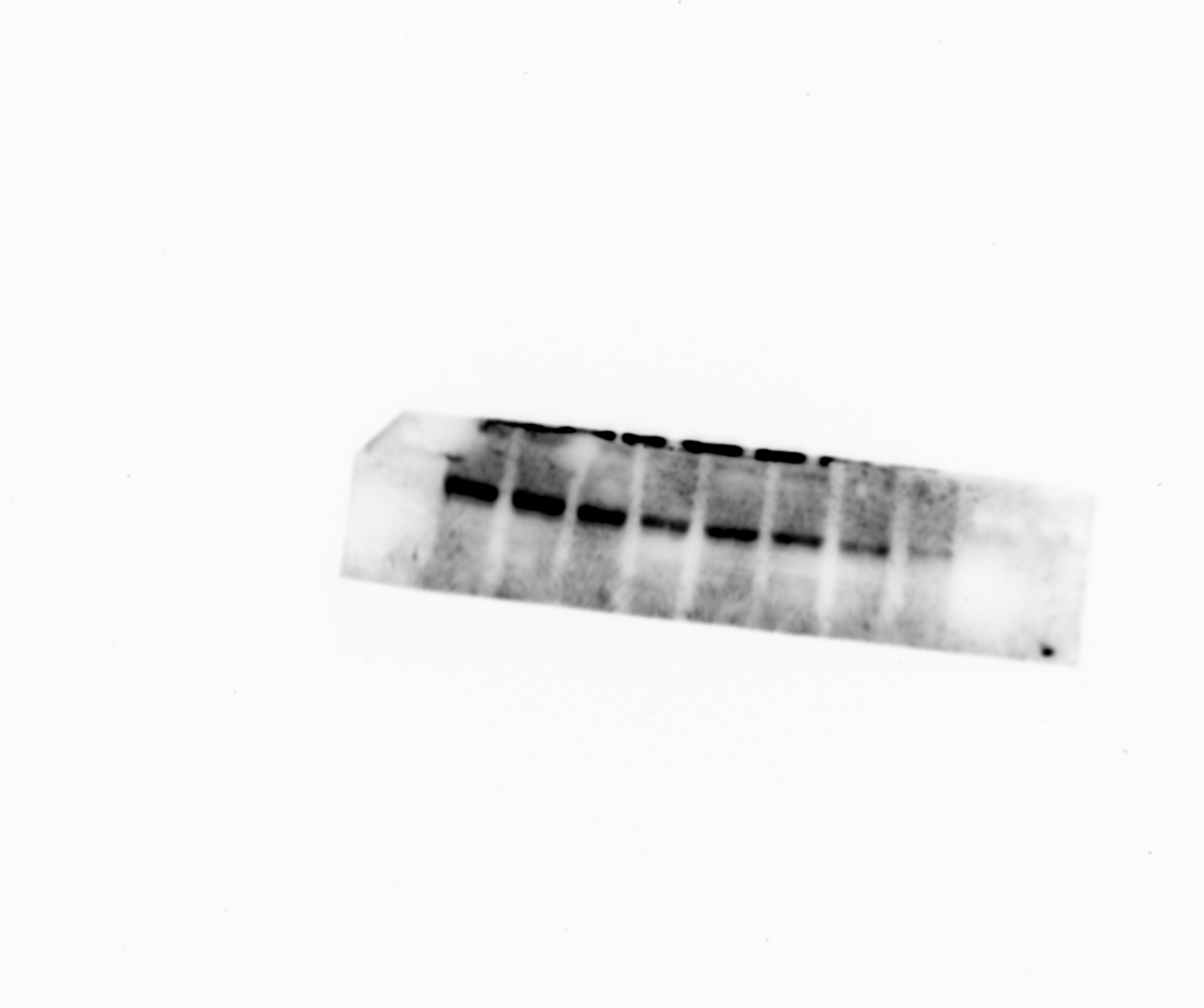

Supplement: Supplementary file 10 — Source data Fig. 7 [file 44319_2024_352_MOESM10_ESM.zip › Figure 7/7L/western IRF3.tif]

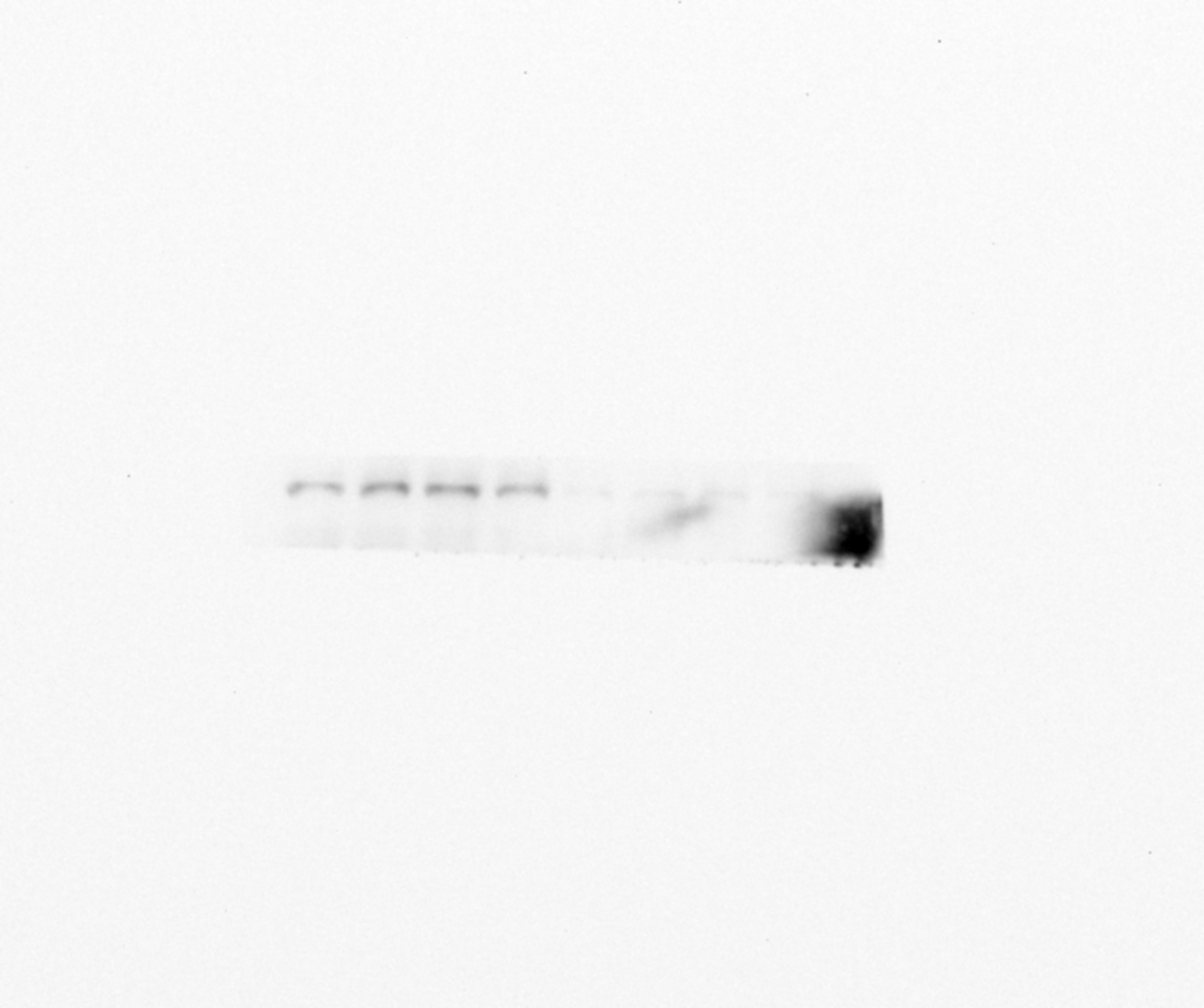

Supplement: Supplementary file 10 — Source data Fig. 7 [file 44319_2024_352_MOESM10_ESM.zip › Figure 7/7L/western TRIM71.tif]

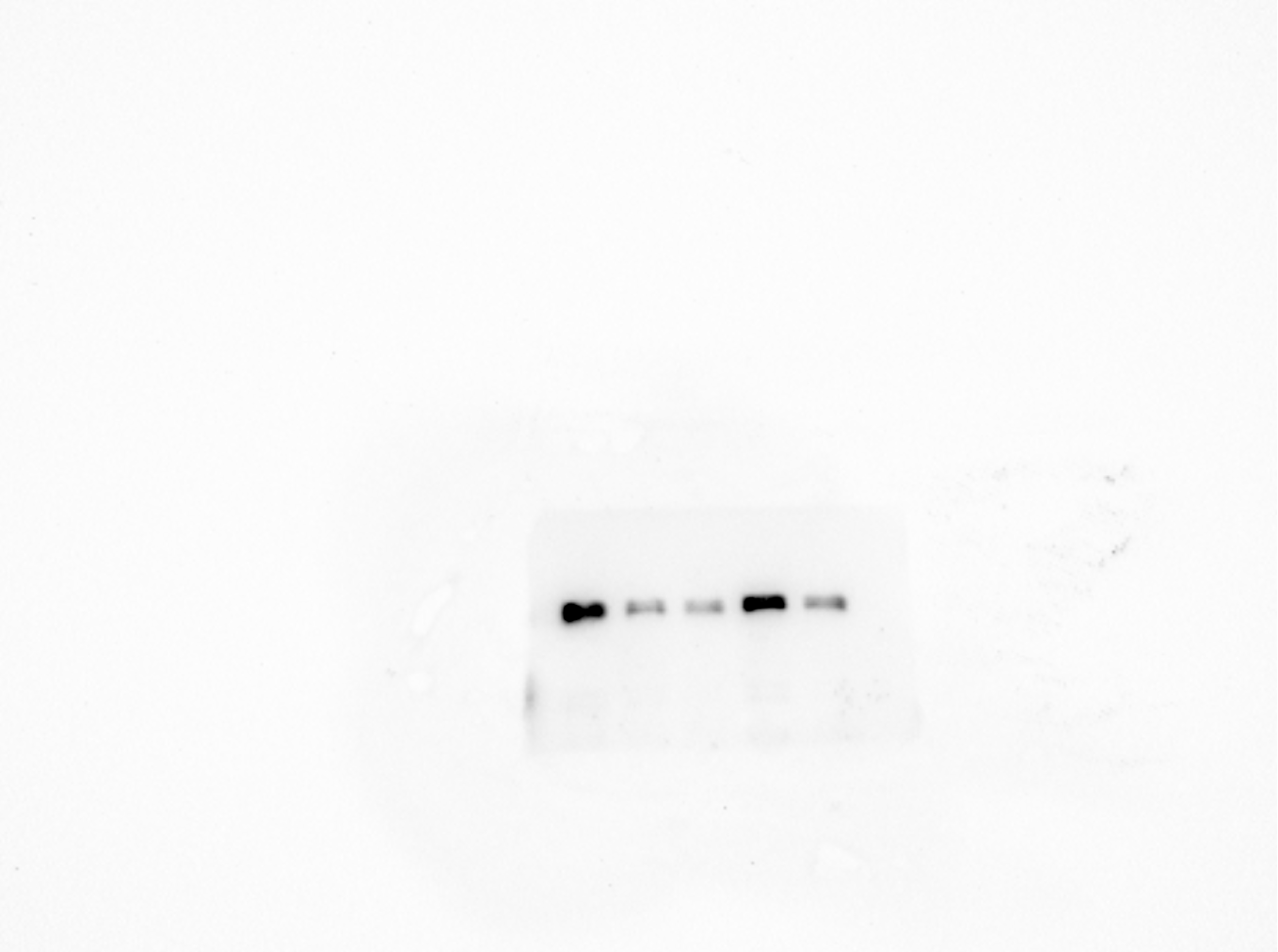

Supplement: Supplementary file 10 — Source data Fig. 7 [file 44319_2024_352_MOESM10_ESM.zip › Figure 7/7M/western IRF3.tif]

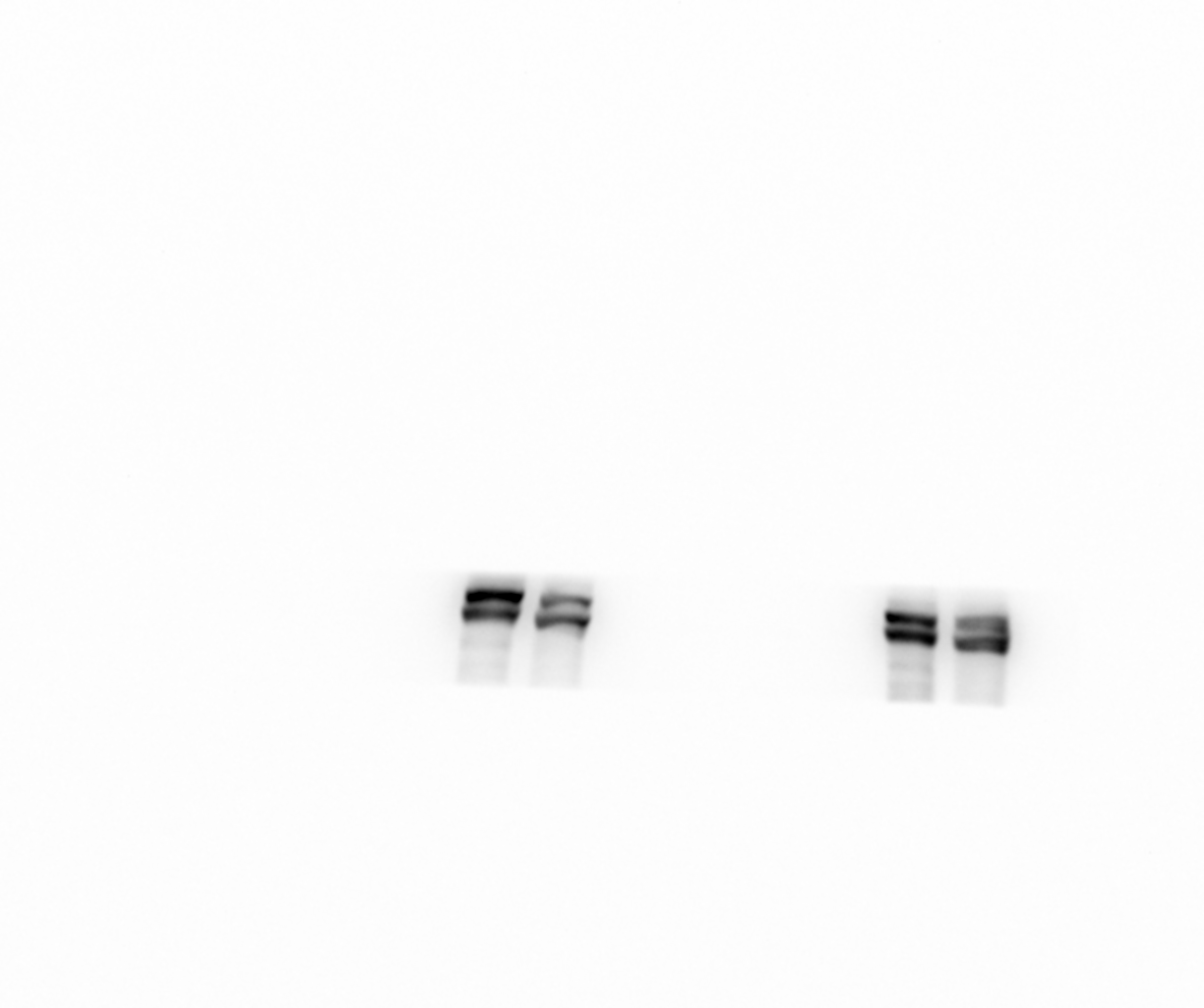

Supplement: Supplementary file 10 — Source data Fig. 7 [file 44319_2024_352_MOESM10_ESM.zip › Figure 7/7M/western TRIM71.tif]

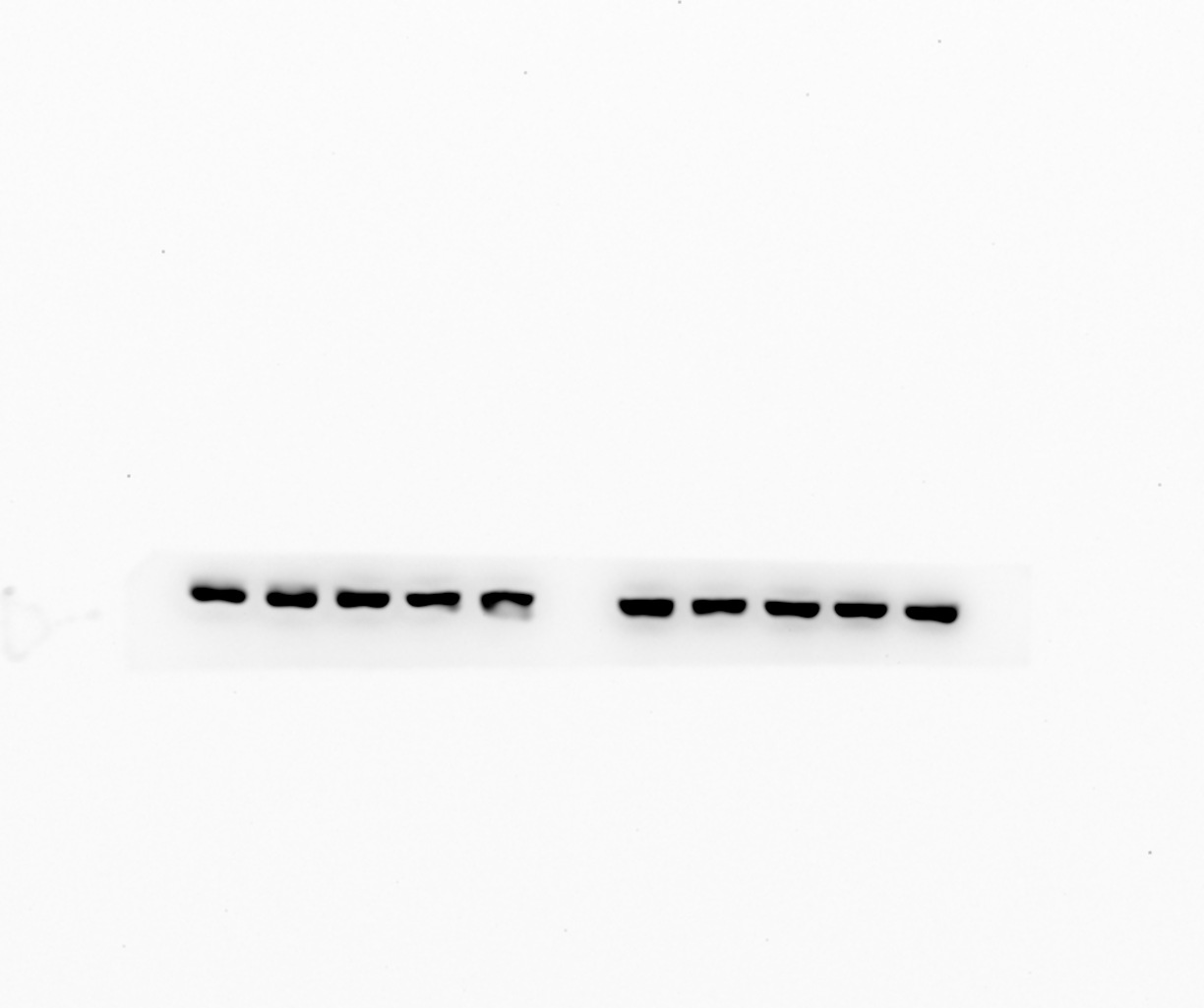

Supplement: Supplementary file 10 — Source data Fig. 7 [file 44319_2024_352_MOESM10_ESM.zip › Figure 7/7M/western actin.tif]

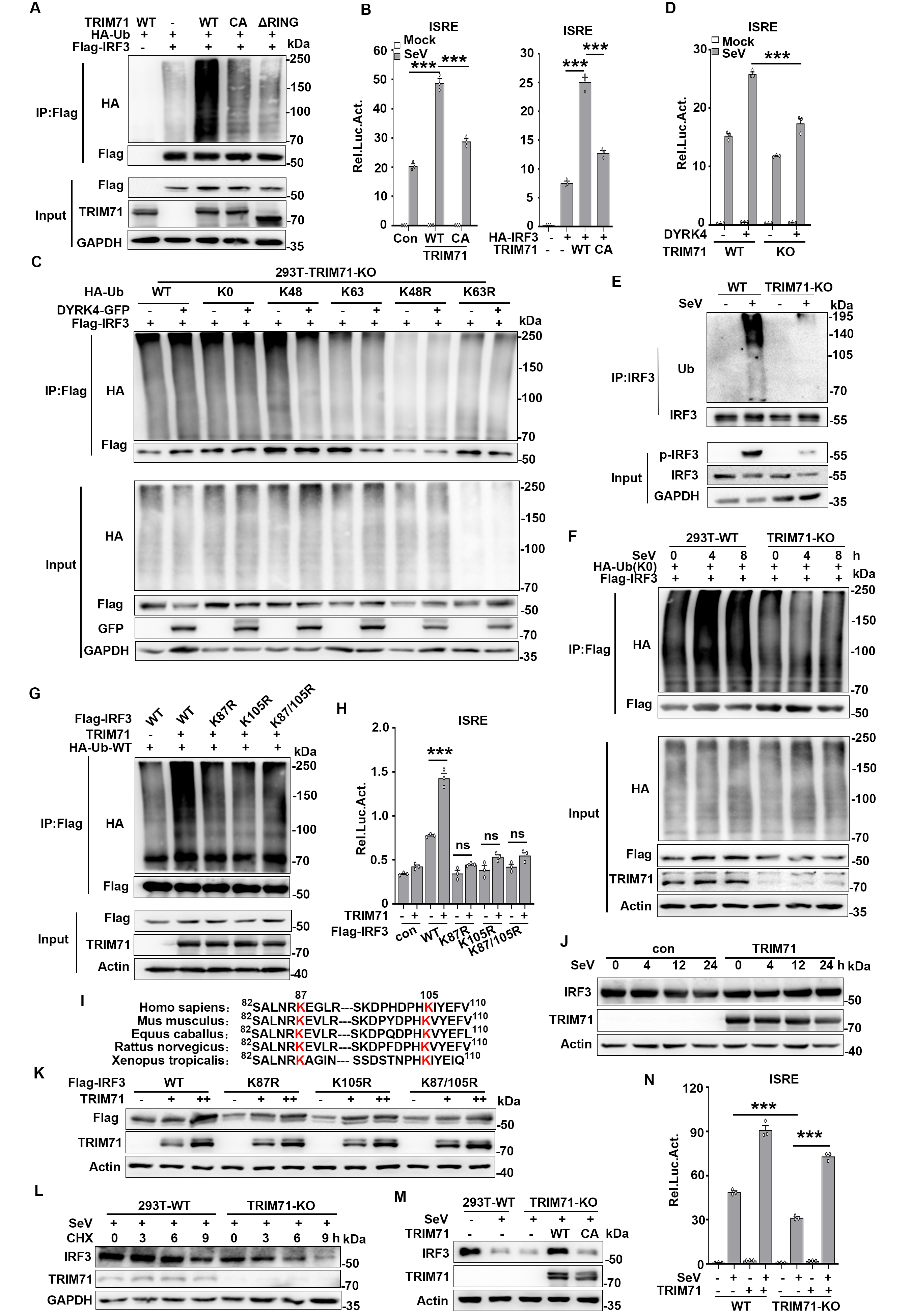

Supplement: Supplementary file 10 — Source data Fig. 7 [file 44319_2024_352_MOESM10_ESM.zip › Figure 7/Figure 7.tif]

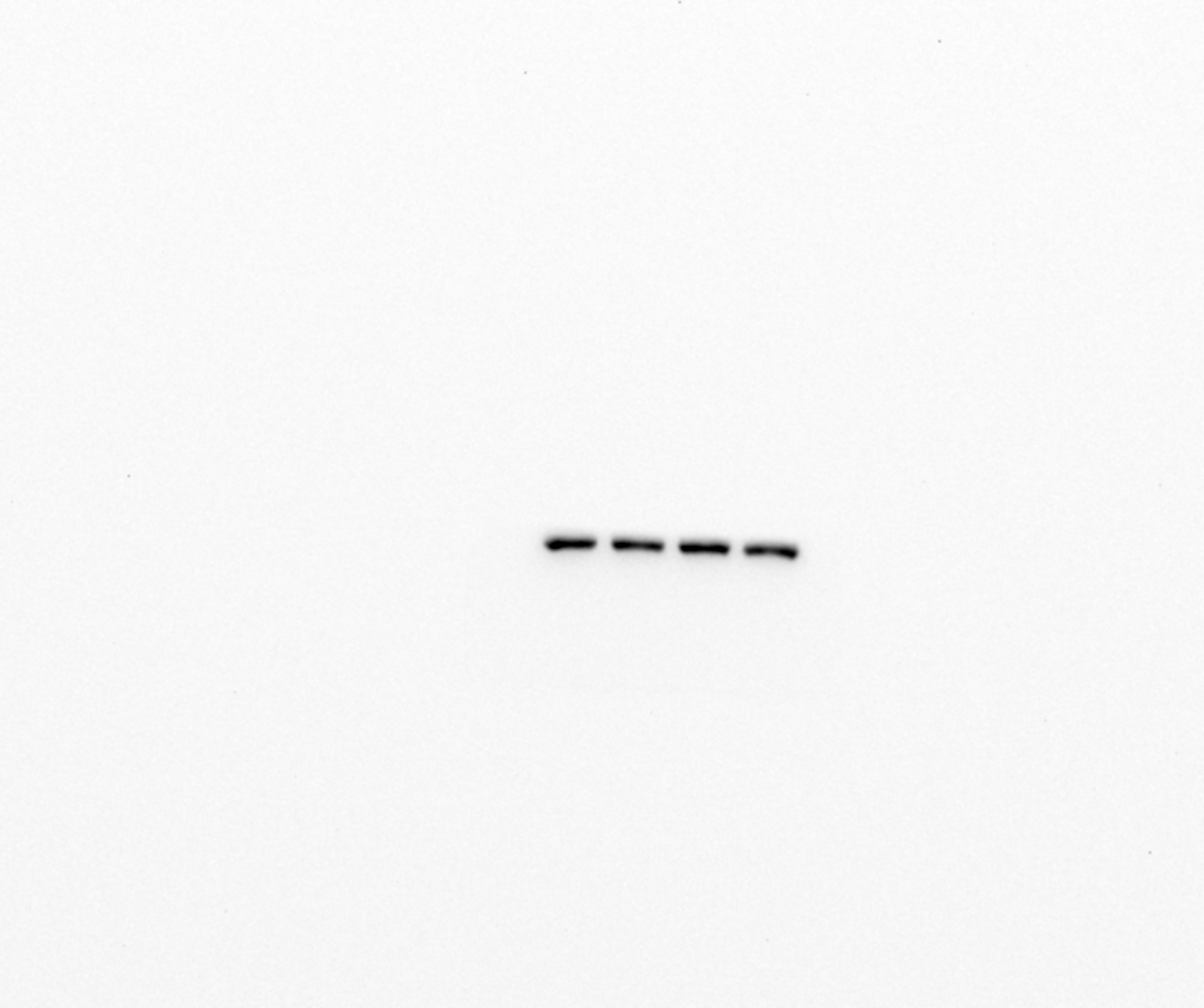

Supplement: Supplementary file 11 — Source data Fig. 8 [file 44319_2024_352_MOESM11_ESM.zip › Figure 8/8A/western myc Input.tif]

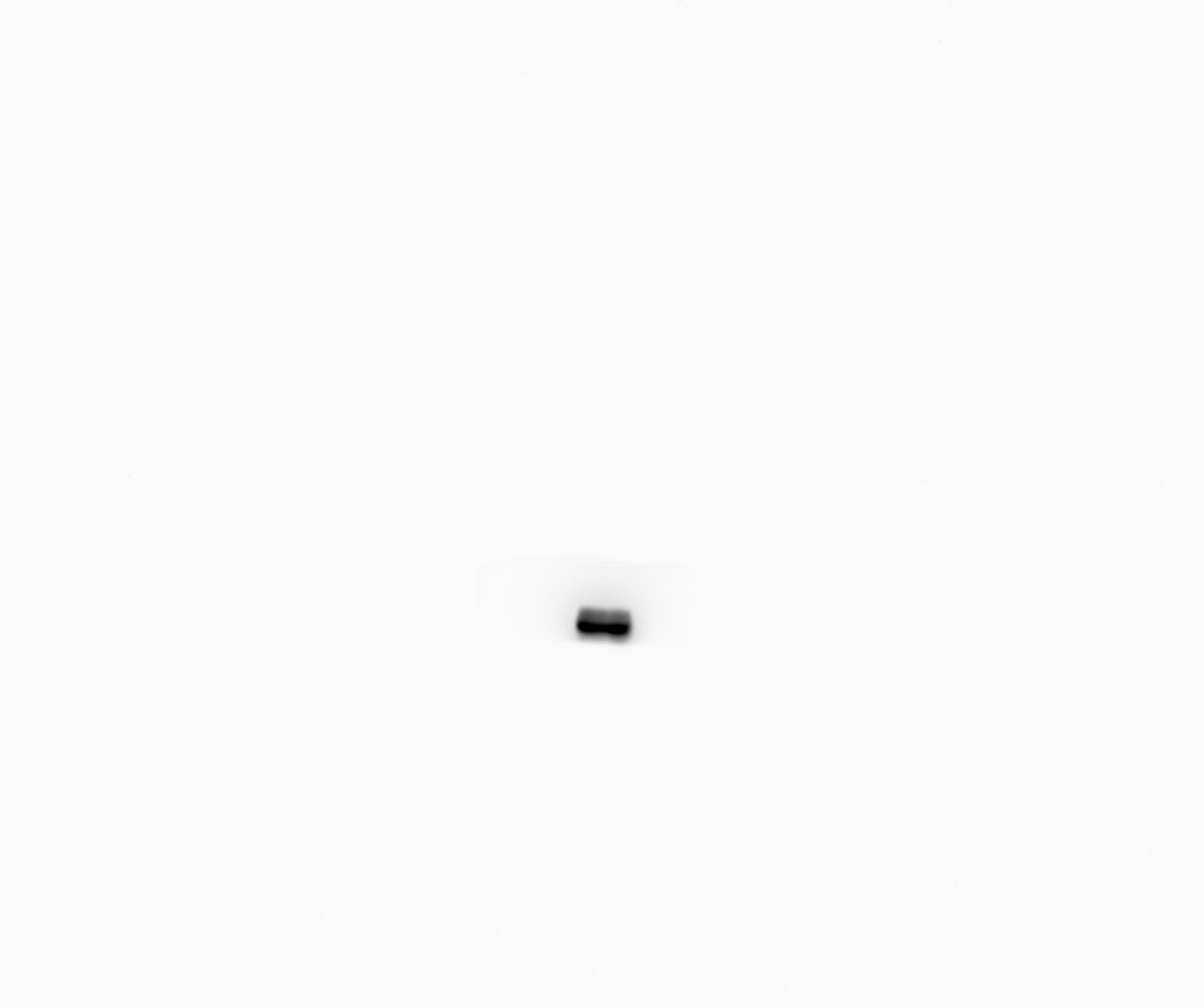

Supplement: Supplementary file 11 — Source data Fig. 8 [file 44319_2024_352_MOESM11_ESM.zip › Figure 8/8A/western Flag-HOIL IP.tif]

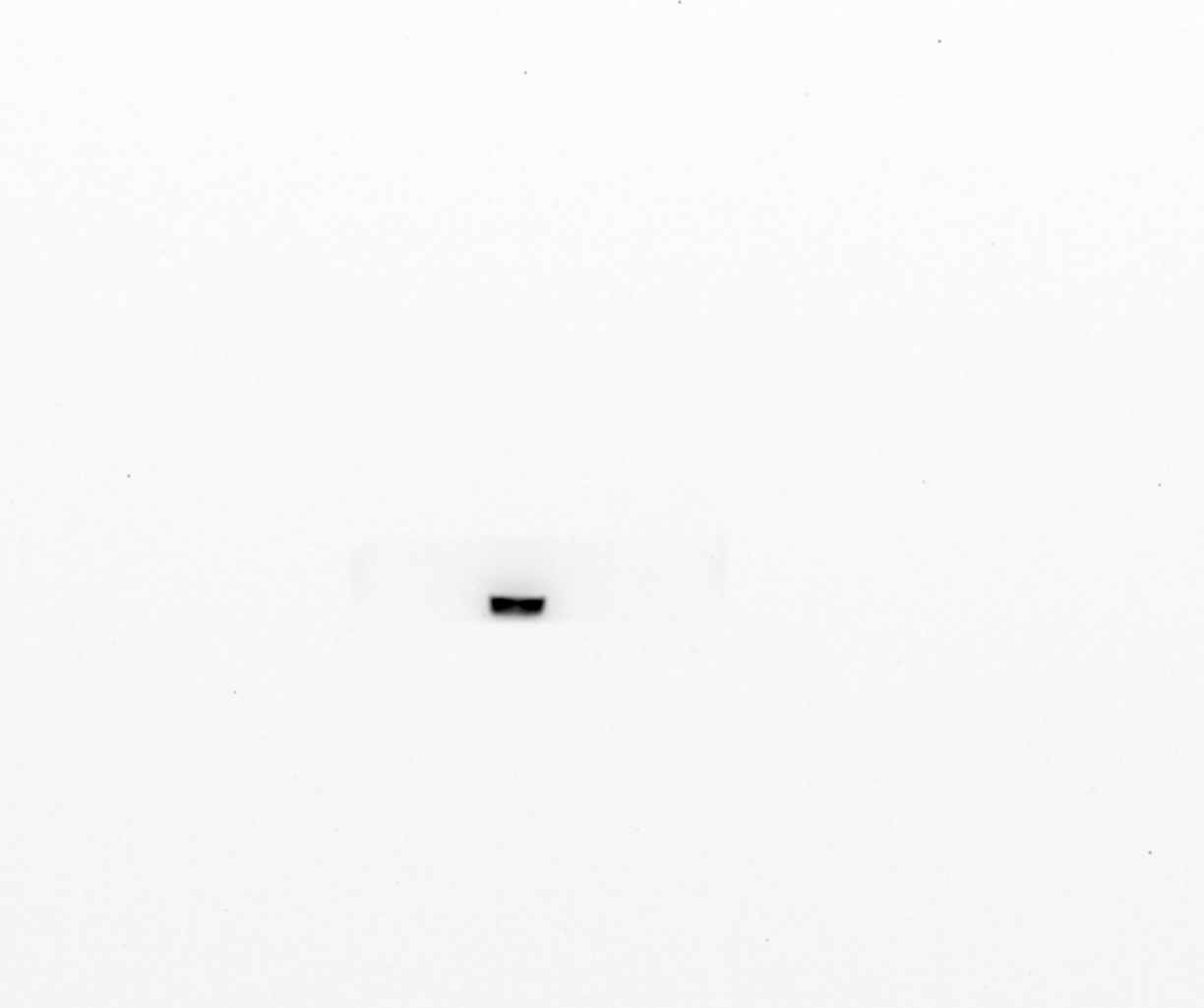

Supplement: Supplementary file 11 — Source data Fig. 8 [file 44319_2024_352_MOESM11_ESM.zip › Figure 8/8A/western Flag-HOIL Input.tif]

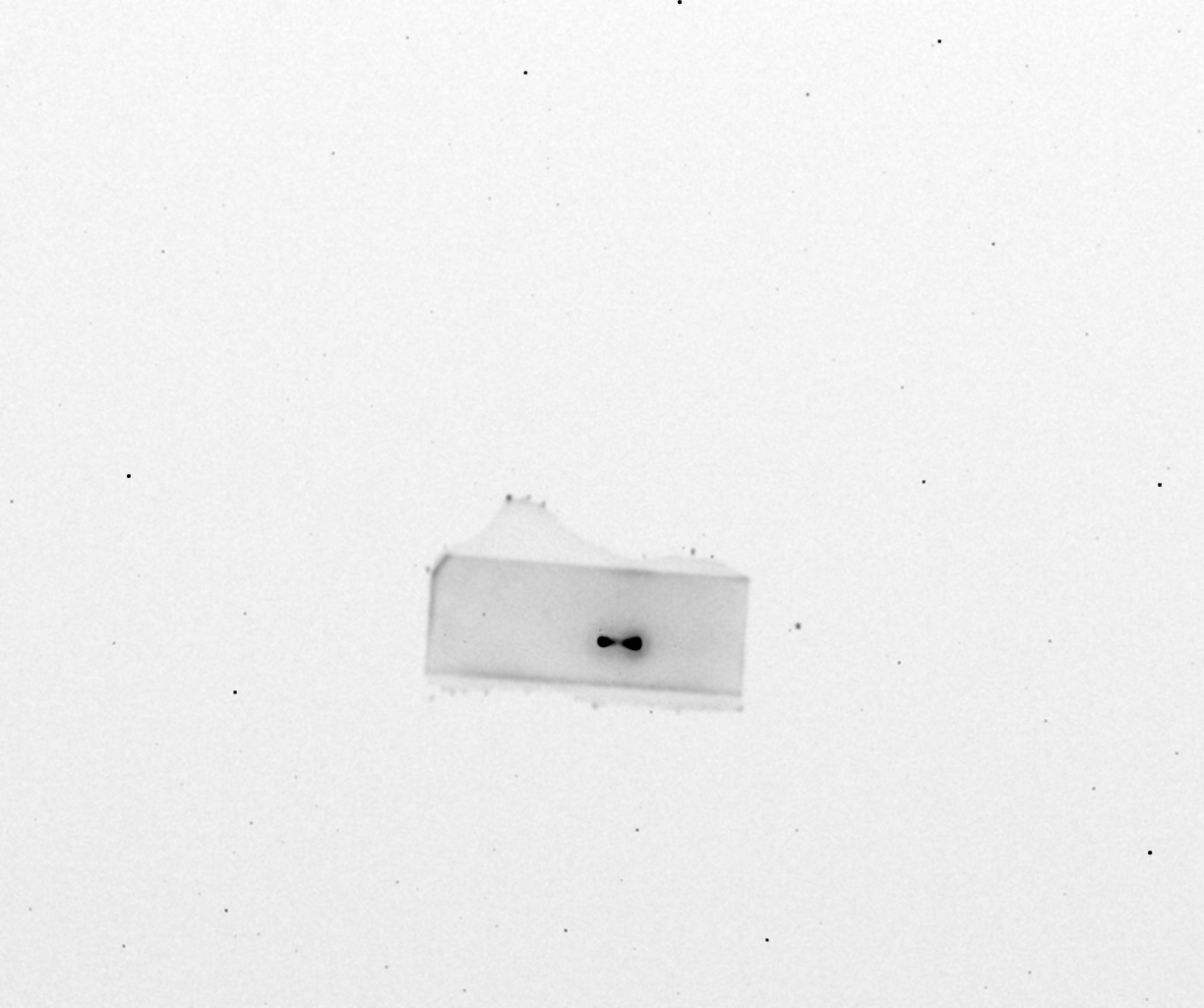

Supplement: Supplementary file 11 — Source data Fig. 8 [file 44319_2024_352_MOESM11_ESM.zip › Figure 8/8A/western Flag-HOIP IP.tif]

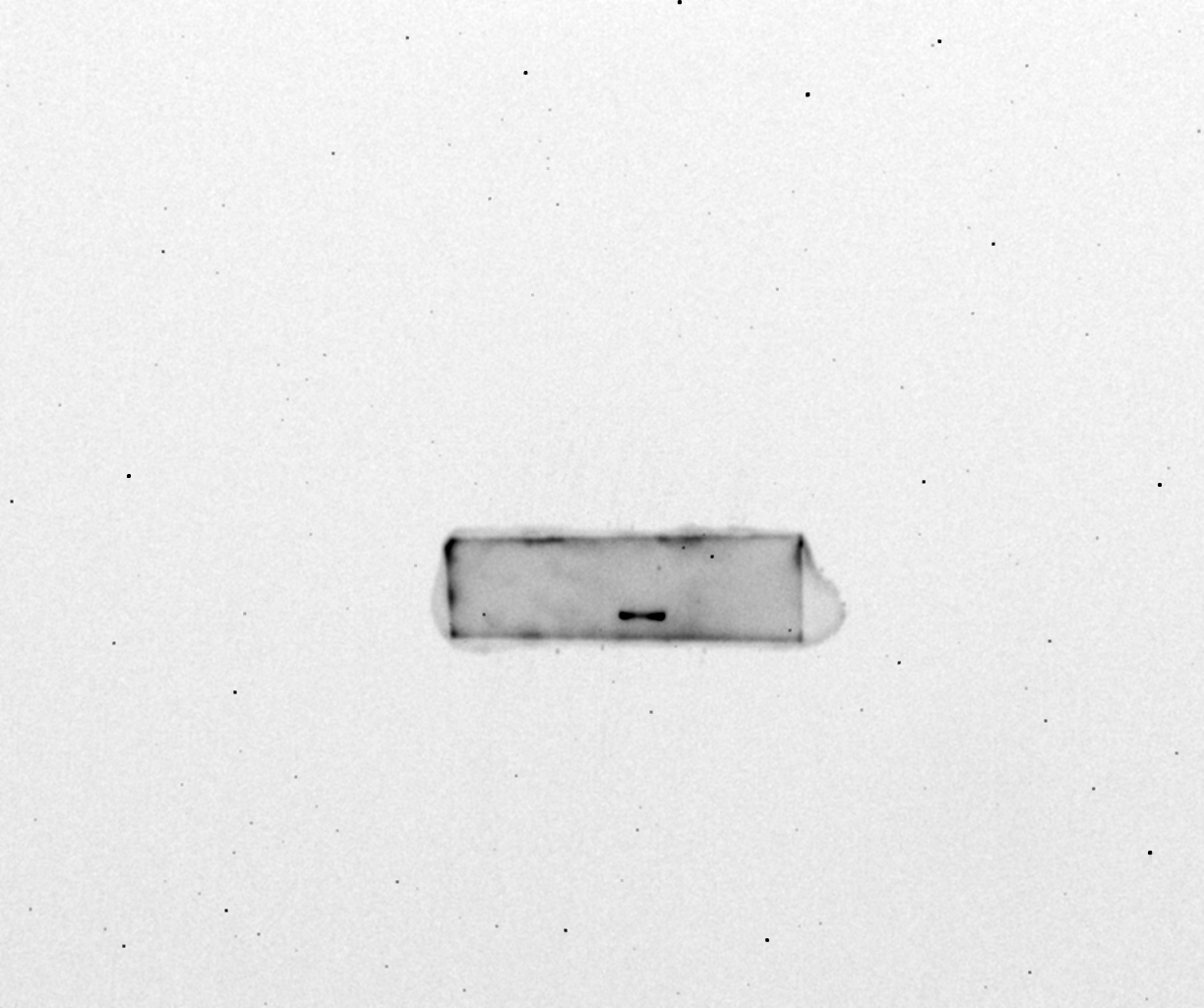

Supplement: Supplementary file 11 — Source data Fig. 8 [file 44319_2024_352_MOESM11_ESM.zip › Figure 8/8A/western Flag-HOIP Input.tif]

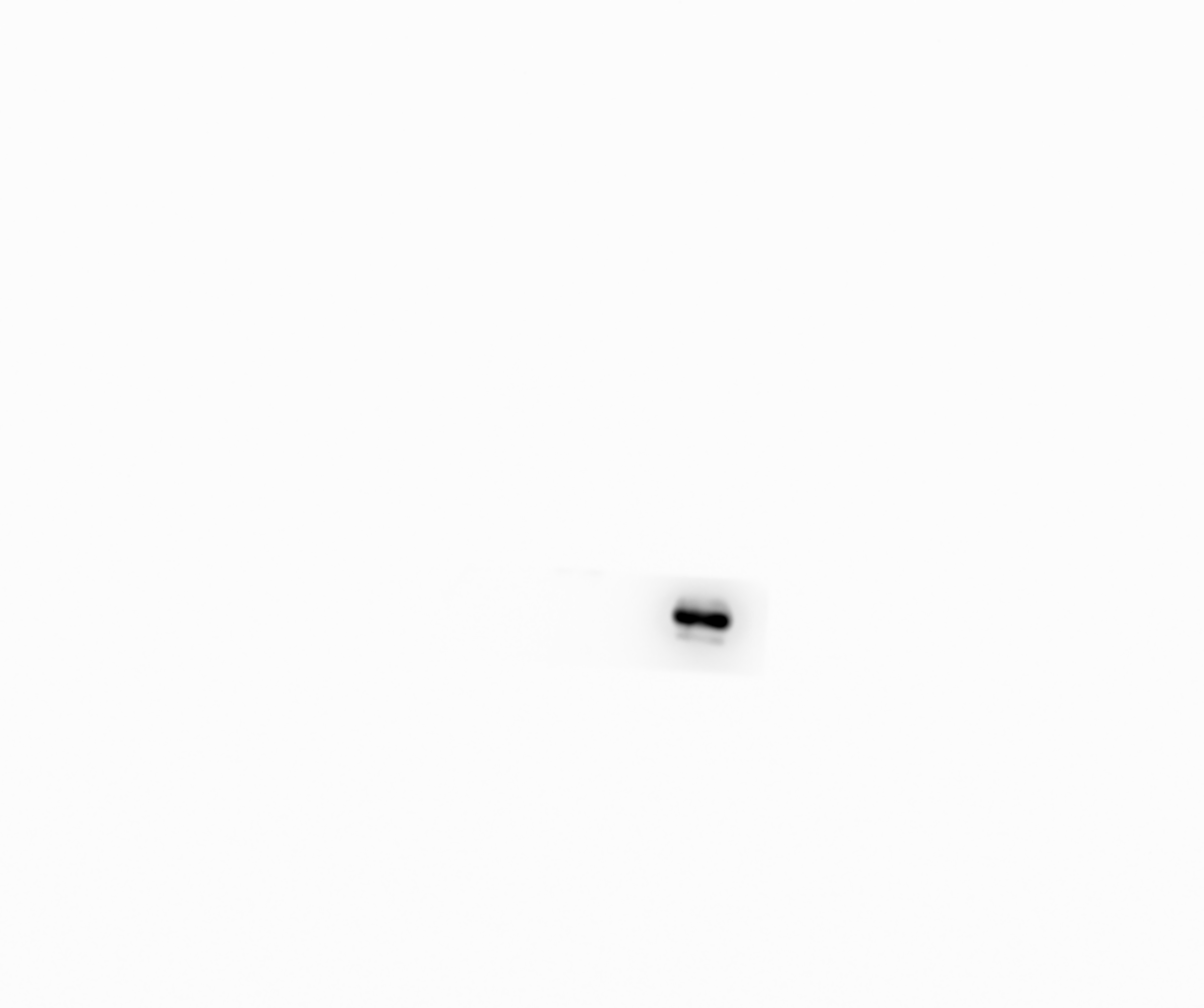

Supplement: Supplementary file 11 — Source data Fig. 8 [file 44319_2024_352_MOESM11_ESM.zip › Figure 8/8A/western Flag-sharpin IP.tif]

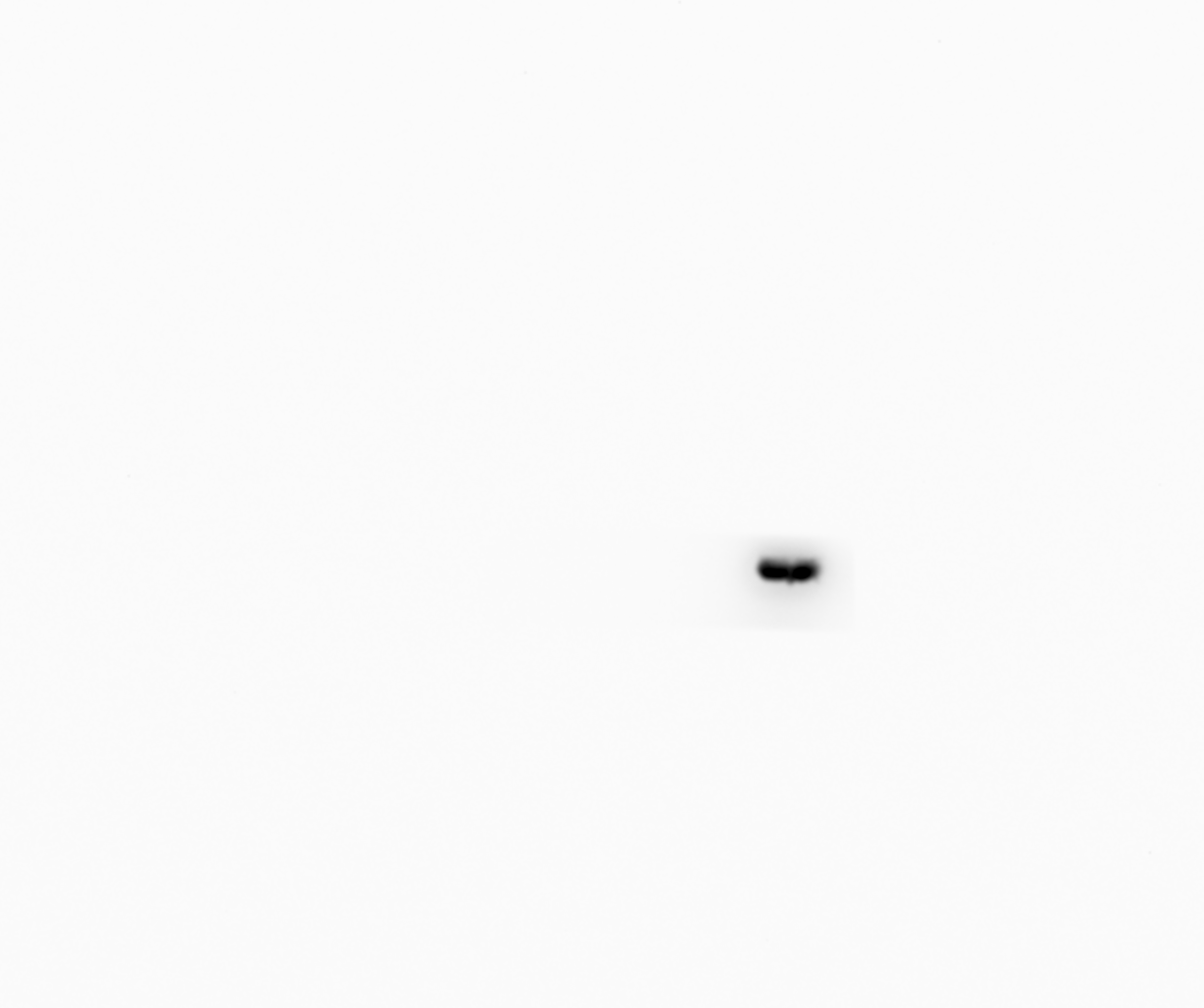

Supplement: Supplementary file 11 — Source data Fig. 8 [file 44319_2024_352_MOESM11_ESM.zip › Figure 8/8A/western Flag-sharpin Input.tif]

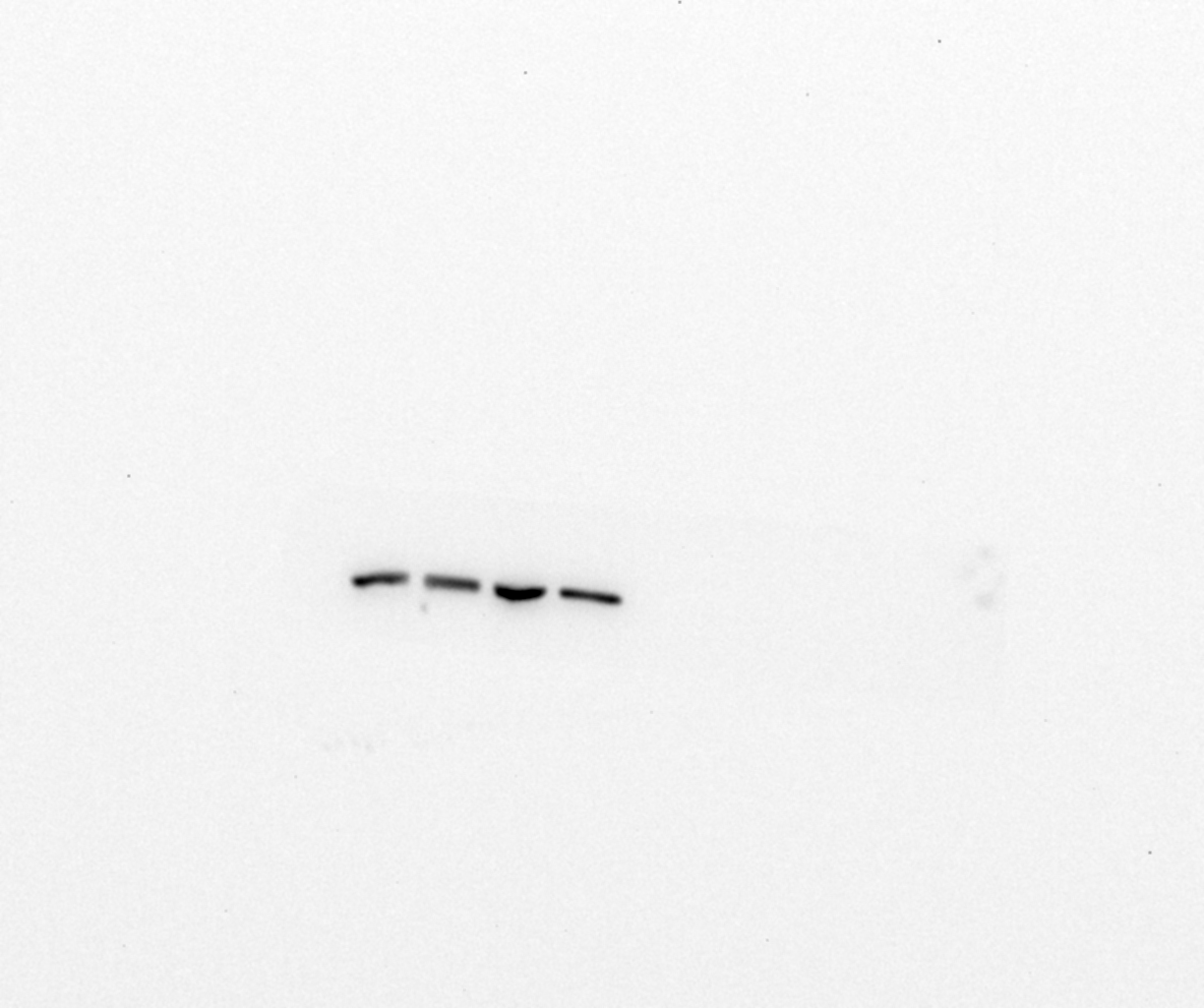

Supplement: Supplementary file 11 — Source data Fig. 8 [file 44319_2024_352_MOESM11_ESM.zip › Figure 8/8A/western GAPDH.tif]

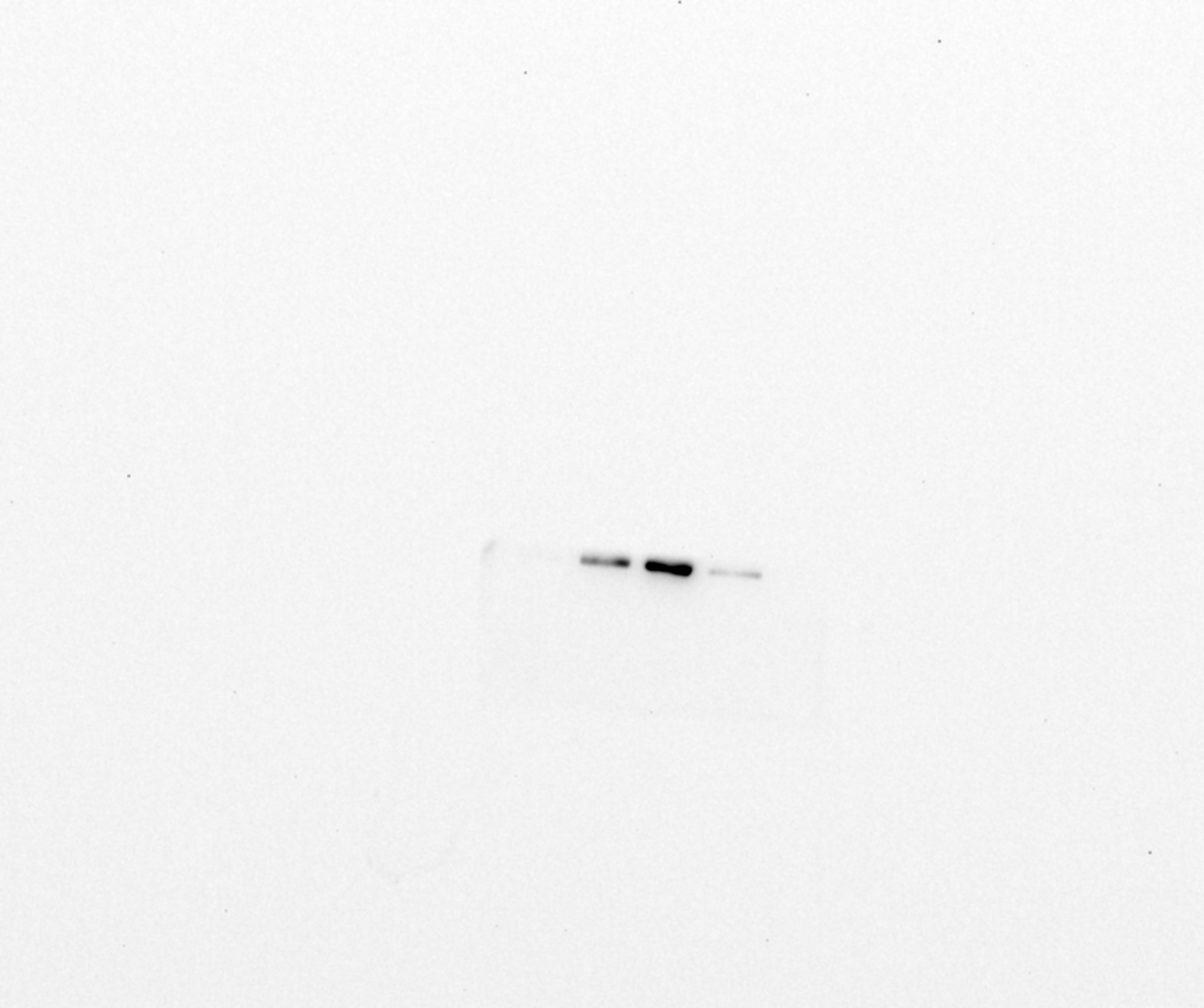

Supplement: Supplementary file 11 — Source data Fig. 8 [file 44319_2024_352_MOESM11_ESM.zip › Figure 8/8A/western myc IP.tif]

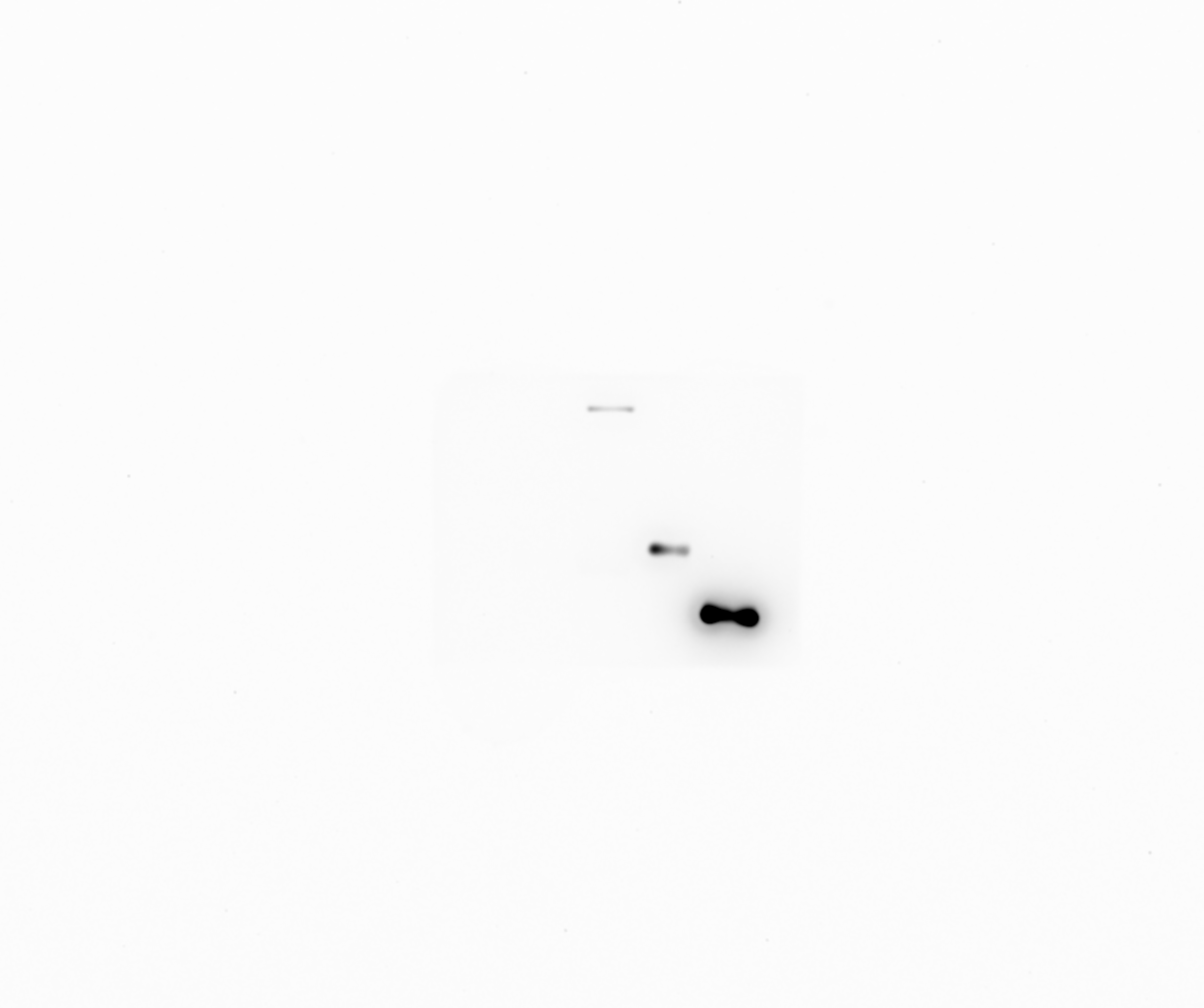

Supplement: Supplementary file 11 — Source data Fig. 8 [file 44319_2024_352_MOESM11_ESM.zip › Figure 8/8B/western Flag IP.tif]

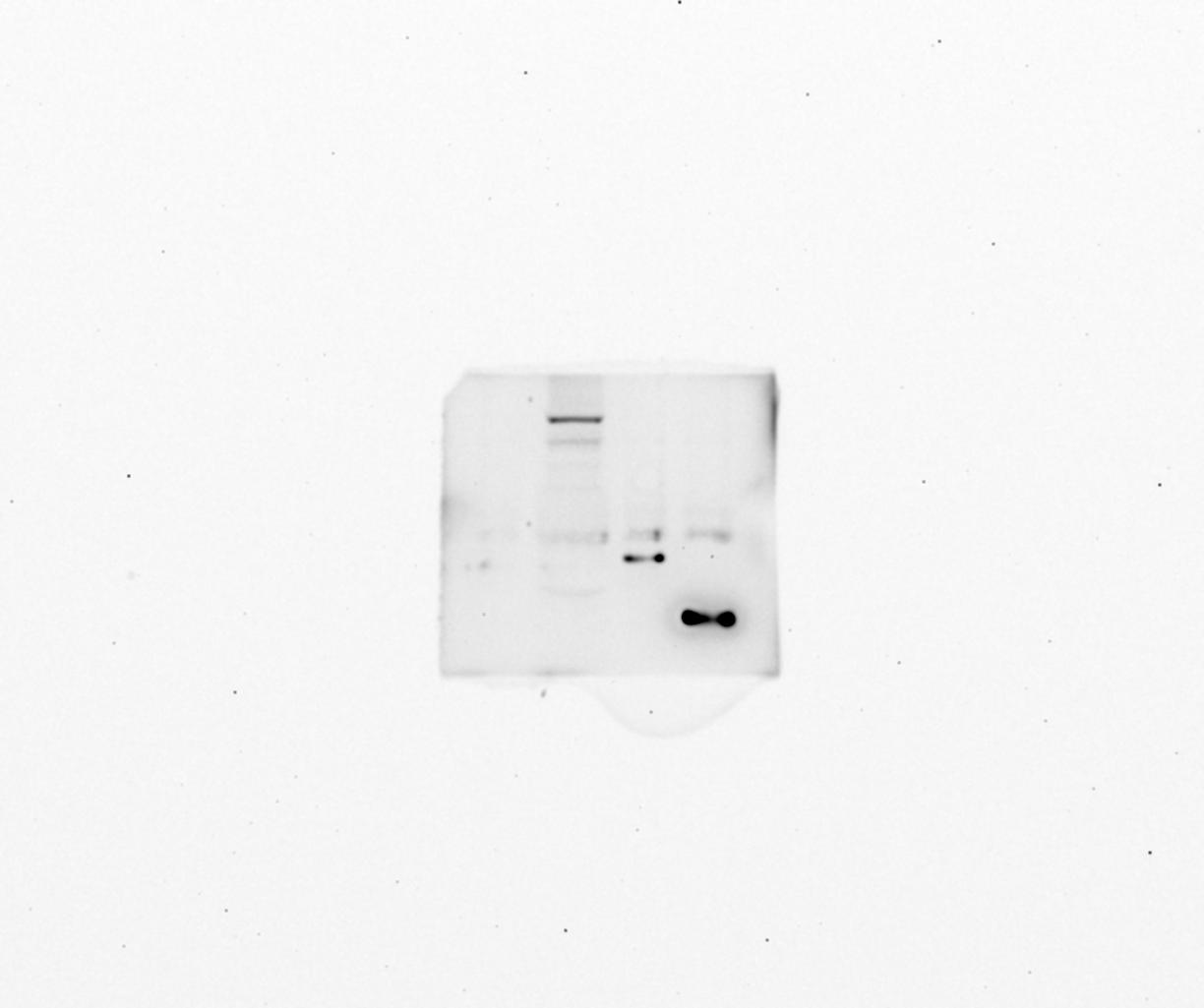

Supplement: Supplementary file 11 — Source data Fig. 8 [file 44319_2024_352_MOESM11_ESM.zip › Figure 8/8B/western Flag Input.tif]

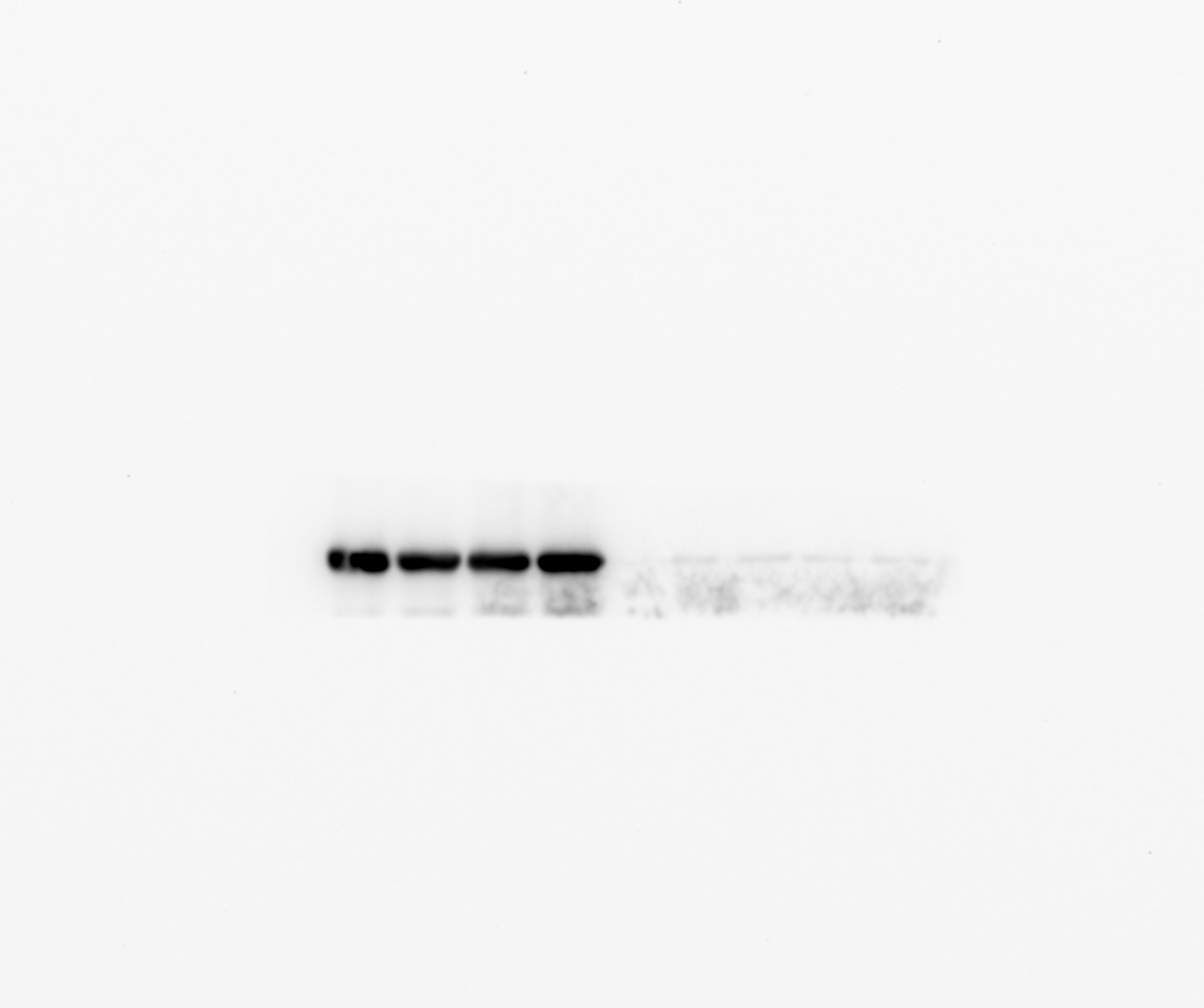

Supplement: Supplementary file 11 — Source data Fig. 8 [file 44319_2024_352_MOESM11_ESM.zip › Figure 8/8B/western GAPDH.tif]

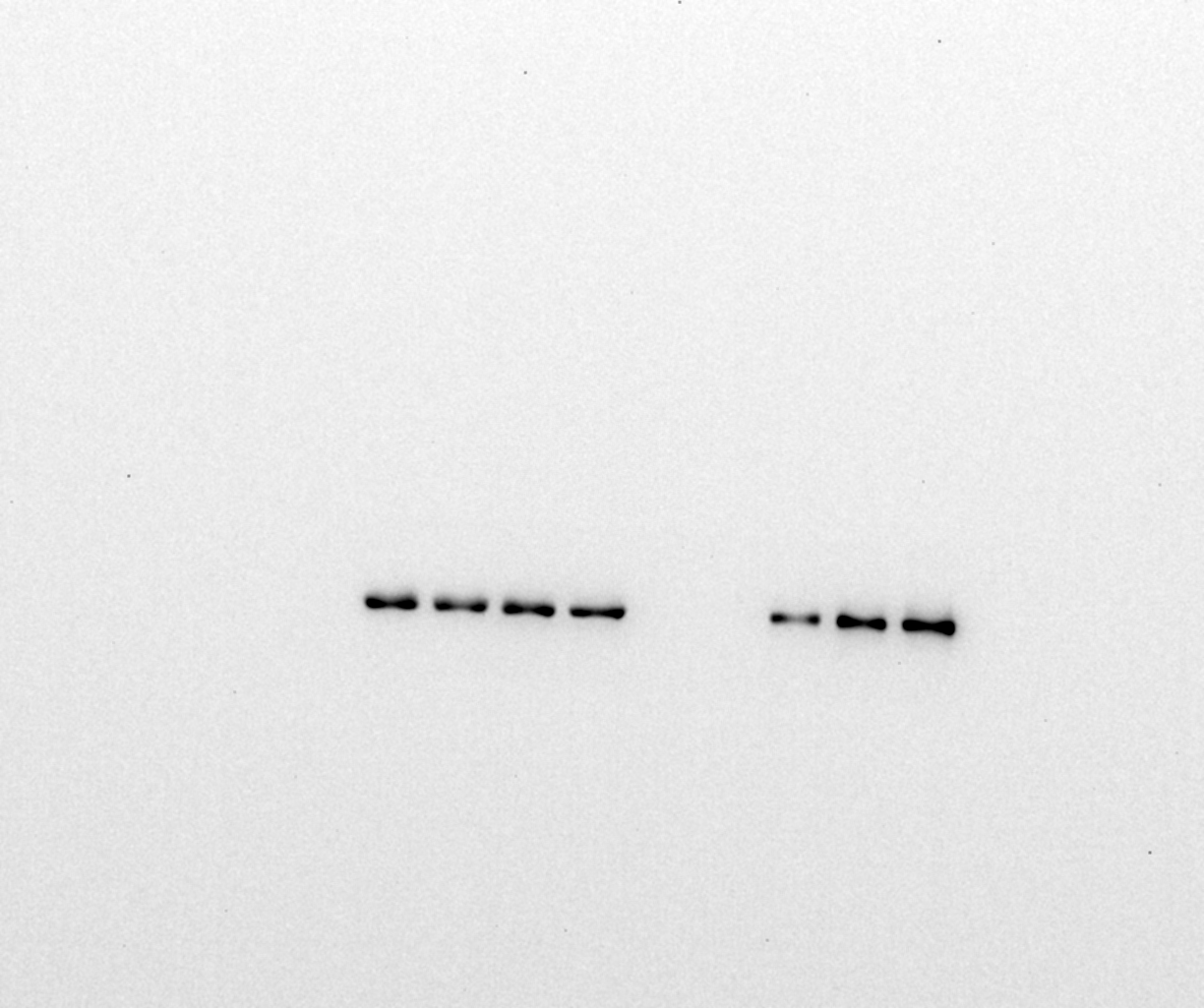

Supplement: Supplementary file 11 — Source data Fig. 8 [file 44319_2024_352_MOESM11_ESM.zip › Figure 8/8B/western GFP Input-IP.tif]

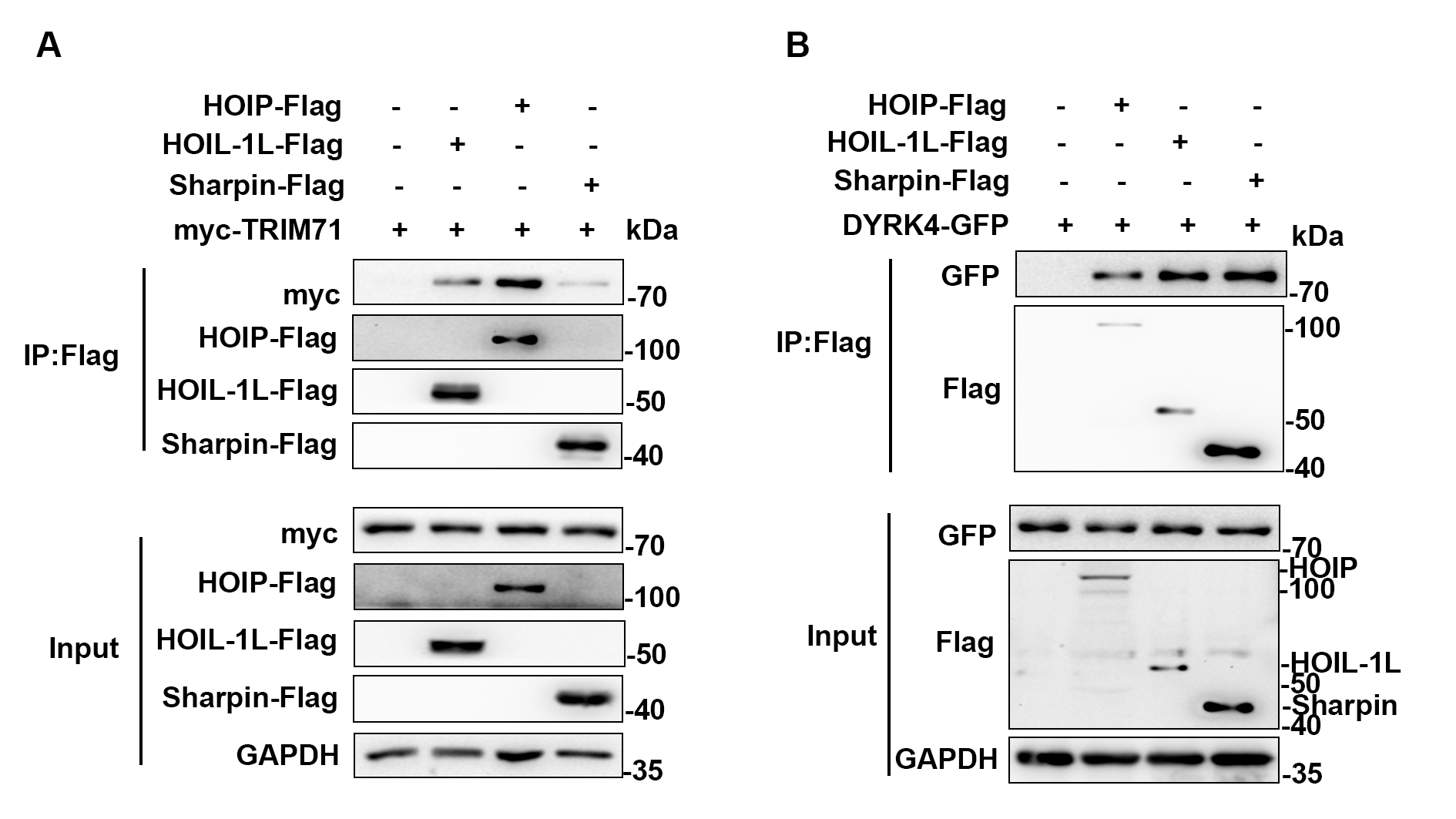

Supplement: Supplementary file 11 — Source data Fig. 8 [file 44319_2024_352_MOESM11_ESM.zip › Figure 8/Figure 8.tif]

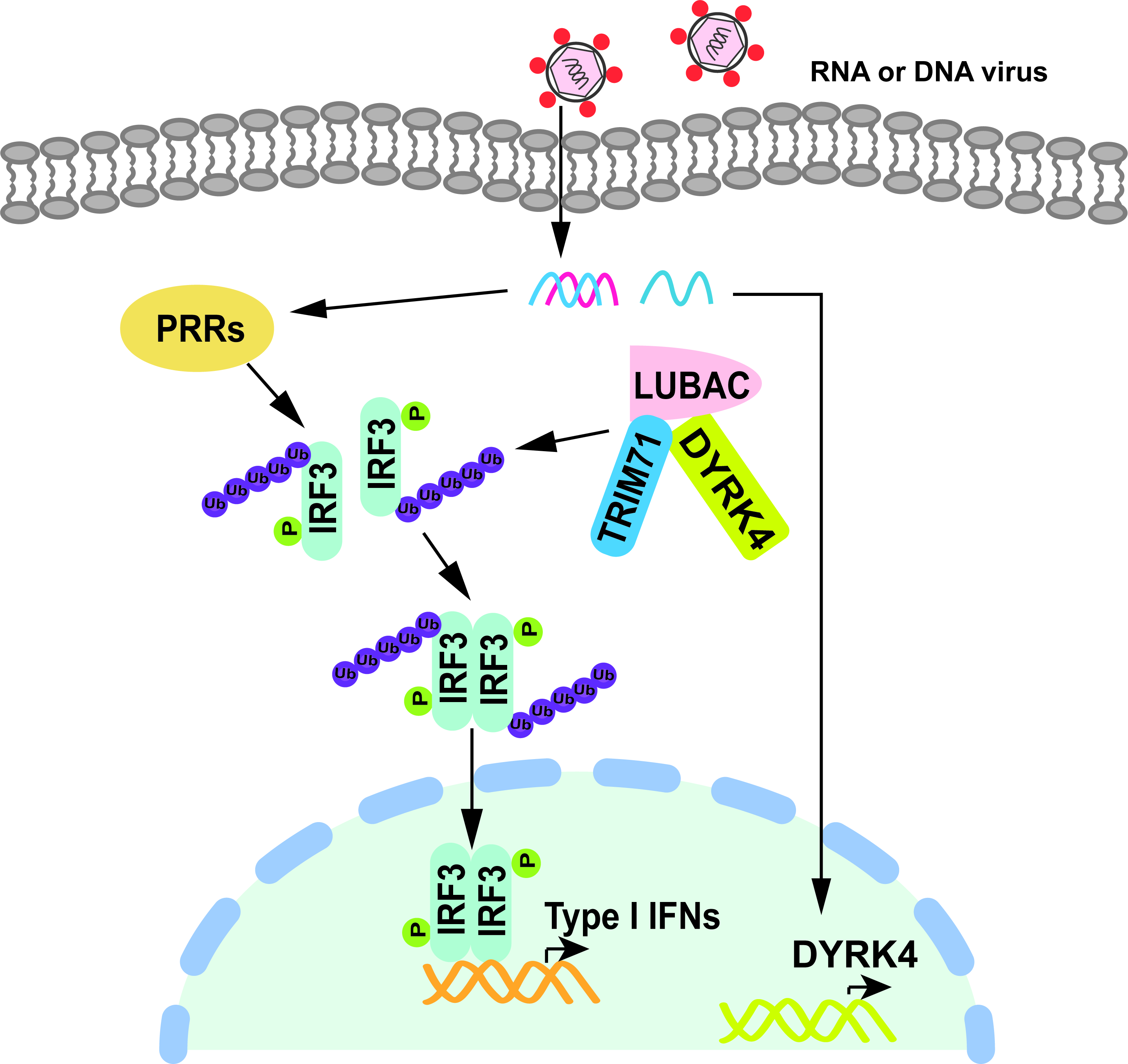

Supplement: Supplementary file 12 — Source data Fig. 9 [file 44319_2024_352_MOESM12_ESM.zip › Figure 9/Figure 9 working model.tif]
