# Supplementary material for: DYRK4 upregulates antiviral innate immunity by promoting IRF3 activation
Source: EMBO Rep. 2024 Dec 19;26(3):690–719. doi: 10.1038/s44319-024-00352-x (PMC11811199; doi:10.1038/s44319-024-00352-x)
Supplement: Supplementary file 13 — Expanded View Figures [file 44319_2024_352_MOESM13_ESM.pdf]

Expanded View Figures

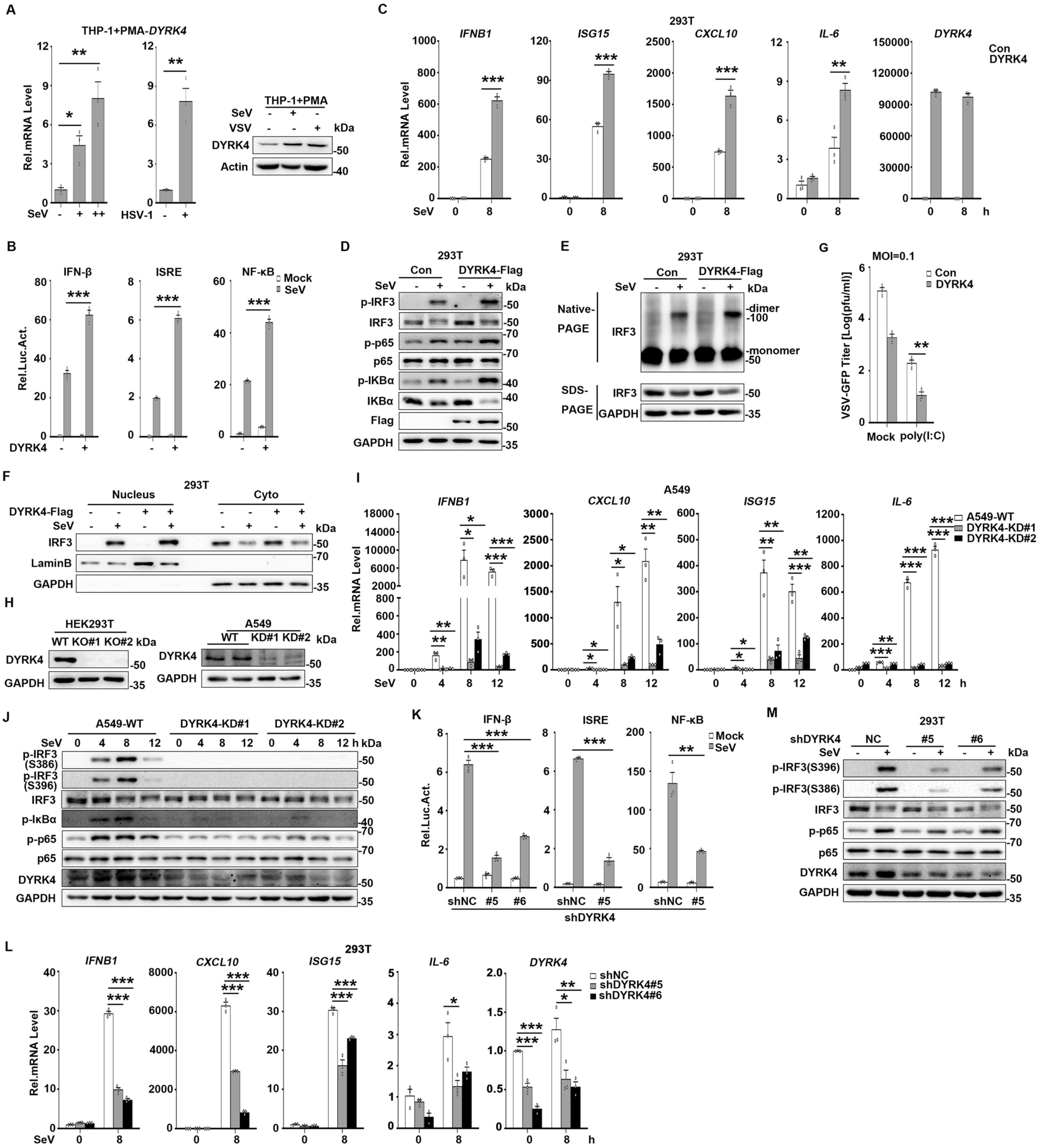

 © The Author(s)

Figure EV1. DYRK4 overexpression or knockdown promotes or inhibits virus-triggered signaling.

Effects of viral infection on DYRK4 mRNA and protein levels. THP-1-derived macrophages were infected with SeV or HSV-1 for 8 h before qPCR analysis and were infected with SeV or VSV for 8 h before immunoblotting analysis with anti-DYRK4 (right). (*P* value; SeV +: 0.01049, SeV + +: 0.00497, HSV-1: 0.00216) ($n = 3$ biological replicates). (**A**) DYRK4 activated the IFN-β promoter, ISRE, and NF-κB. HEK293T cells were transfected with the IFN-β, ISRE, and NF-κB reporters and the control or DYRK4 plasmid for 24 h and then infected with SeV for 12 h before luciferase assays. (*P* value; IFN-β: 0.00036, ISRE: 1.1×10^{-5}, NF-κB: 4.0×10^{-5}) ($n = 3$ biological replicates). (**B**) Effects of DYRK4 on the SeV-induced transcription of downstream genes. HEK293T cells were transfected with the control or DYRK4 plasmid for 24 h and then infected with SeV for 8 h before qPCR analysis. (*P* value; IFNB1: 7.6×10^{-5}, CXCL10: 0.00058, ISG15: 0.000143, IL-6: 0.0097) ($n = 3$ biological replicates). (**C**) Effects of DYRK4 on SeV-induced phosphorylation of IRF3 (Ser396), p65 and IκBα. HEK293T cells were transfected with the control or DYRK4 plasmid for 24 h and then infected with SeV for 8 h before immunoblotting analysis with the indicated antibodies. (**D**) DYRK4 enhanced the SeV-induced dimerization of IRF3. HEK293T cells were transfected with the control or DYRK4 plasmid for 24 h and then infected with SeV for 8 h. Cell lysates were separated by native (upper panel) or SDS (bottom panel) PAGE and analyzed by immunoblotting with the indicated antibodies. (**E**) Effects of DYRK4 on the SeV-induced nuclear translocation of IRF3. HEK293T cells were transfected with the control or DYRK4 plasmid for 24 h and then infected with SeV for 8 h. Immunoblot analysis of IRF3 in the cytoplasmic (Cyto) and nuclear fractions was performed with the indicated antibodies. (**F**) Effects of DYRK4 on VSV-GFP replication. HEK293T cells were transfected with the control or DYRK4 plasmid for 24 h, mock-transfected or transfected with poly(I:C) (1 μg) for 16 h, and then infected with VSV-GFP (MOI of 0.1). The supernatants were harvested 24 h after infection for standard plaque assays. (*P* value = 0.00177) ($n = 3$ biological replicates). (**G**) DYRK4-deficient (KO) HEK293T clones and DYRK4-knockdown (KD) A549 cells were generated via the CRISPR-Cas9 method. Deficiencies of DYRK4 in the KO clones and DYRK4-KD A549 cells were confirmed by immunoblotting analysis with anti-DYRK4. (**H**) Effects of DYRK4 knockdown on the SeV-induced transcription of downstream genes. DYRK4-KD and control A549 cells were infected with SeV for the indicated times before qPCR analysis. (*P* value; IFNB1; 4 h; KD#1: 0.0053, KD#2: 0.0048, 8 h; KD#1: 0.0228, KD#2: 0.0254, 12 h; KD#1: 0.00065, KD#2: 0.00072, CXCL10; 4 h; KD#1: 0.0259, KD#2: 0.0307, 8 h; KD#1: 0.014, KD#2: 0.0213, 12 h; KD#1: 0.00106, KD#2: 0.0033, ISG15; 4 h; KD#1: 0.048, KD#2: 0.044, 8 h; KD#1: 0.0023, KD#2: 0.0048, 12 h; KD#1: 0.00096, KD#2: 0.0029, IL-6; 4 h; KD#1: 2.1×10^{-5}, KD#2: 0.0079, 8 h; KD#1: 9.3×10^{-6}, KD#2: 1.1×10^{-5}, 12 h; KD#1: 4.2×10^{-6}, KD#2: 4.6×10^{-6}) ($n = 3$ biological replicates). (**I**) Effects of DYRK4 knockdown on SeV-induced phosphorylation of IRF3 (Ser396, Ser386), p65 and IκBα. DYRK4-KD and control A549 cells were infected with SeV for the indicated times before immunoblotting analysis with the indicated antibodies. (**J**) Effects of DYRK4 knockdown on Sev-induced activation of the IFN-β promoter, ISRE, and NF-κB. HEK293T cells were transfected with the IFN-β, ISRE, NF-κB reporter, and control or DYRK4 RNAi plasmids for 36 h and then infected with SeV for 12 h before luciferase assays. (*P* value; IFN-β; #5: 3.6×10^{-5}, #6: 7.0×10^{-5}, ISRE; #5: 5.1×10^{-6}, NF-κB; #5: 0.00305) ($n = 3$ biological replicates). (**K**) Effects of DYRK4 knockdown on the SeV-induced transcription of downstream genes. HEK293T cells were transfected with control or DYRK4 RNAi plasmids for 36 h, after which the cells were infected with SeV for 8 h before qPCR analysis. (*P* value; IFNB1; #5: 1.2×10^{-5}, #6: 4.0×10^{-6}, CXCL10; #5: 4.5×10^{-5}, #6: 7.9×10^{-6}, ISG15; #5: 0.00065, #6: 0.00023, IL-6; #5: 0.0272, DYRK4; 0 h; #5: 0.00057, #6: 1.9×10^{-5}, 8 h; #5: 0.0245, #6: 0.0089) ($n = 3$ biological replicates). (**L**) Effects of DYRK4 knockdown on SeV-induced phosphorylation of IRF3 (Ser396, Ser386) and p65. HEK293T cells were transfected with control or DYRK4 RNAi plasmids for 36 h and then infected with SeV for 8 h before immunoblotting analysis with the indicated antibodies. Data information: Data are representative of three biological replicates and are shown as the means with SEMs (**A–C**, **G**, **I**, **K**, **L**); data in (**D–F**, **H**, **J**, **M**) are representative of two replicates. $^{*}P < 0.05$, $^{**}P < 0.01$, $^{***}P < 0.001$; two-tailed unpaired Student's *t* test.

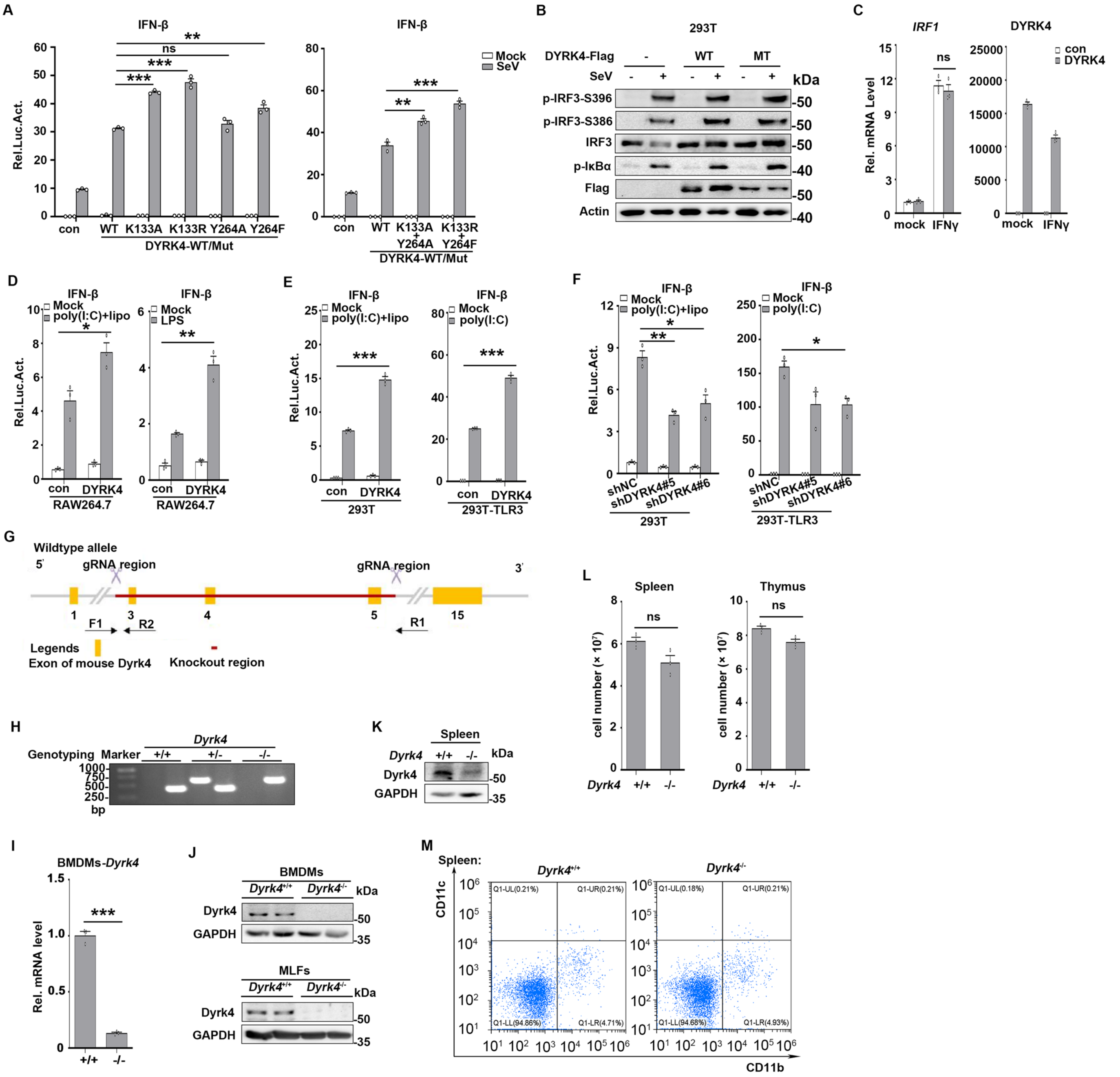
A
IFN-β
Rel.Luc.Act.
con WT K133A K133R Y264A Y264F
DYRK4-WT/Mut
Mock
SeV
B
293T
DYRK4-Flag
SeV
kDa
p-IRF3-S396
p-IRF3-S386
IRF3
p-IκBα
Flag
Actin
C
IRF1
DYRK4
Rel. mRNA Level
mock IFNγ
con
D
Mock
poly(I:C)+lipo
LPS
RAW264.7
E
293T
293T-TLR3
poly(I:C)
F
shNC shDYRK4#5 shDYRK4#6
G
Wildtype allele
gRNA region
Legends
Exon of mouse Dyrk4
Knockout region
H
Genotyping Marker
Dyrk4
bp
I
BMDMs-Dyrk4
Rel. mRNA level
J
BMDMs
MLFs
GAPDH
K
Spleen
L
Spleen
Thymus
cell number (× 10⁷)
M
Spleen:
CD11c
CD11b

Figure EV2. DYRK4 promotes virus-triggered signaling independent of its kinase activity and the generation and analysis of Dyrk4-knockout mice.

DYRK4 and its mutant (left) or double mutants (right) potentiate SeV-induced activation of the IFN-β promoter. The experiments were performed as described in Fig. S1B. (*P* value; K133A: 8.8×10^{-6}, K133R: 0.00015, Y264A: ns, Y264F: 0.00302, K133A + Y264A: 4.4×10^{-6}, K133R + Y264F: 3.3×10^{-6}) ($n = 3$ biological replicates). (**A**) Effects of DYRK4-MT on SeV-induced phosphorylation of IRF3 (Ser396, Ser386) and IκBα. HEK293T cells were transfected with control or DYRK4-WT or DYRK4-MT (K133R/Y264F) plasmids for 24 h and then infected with SeV for 8 h before immunoblotting analysis with the indicated antibodies. (**B**) DYRK4 does not potentiate the IFN-γ-induced transcription of *IRF1*. HEK293T cells were transfected with the control or DYRK4 plasmid for 24 h and then treated with IFN-γ (100 μg/ml) for 8 h before qPCR analysis. (ns: not significant) ($n = 3$ biological replicates). (**C**) Effects of DYRK4 on cytoplasmic poly(I:C)-induced or LPS-induced activation of the IFN-β promoter. RAW264.7 cells were transfected with the IFN-β reporter and control or DYRK4 plasmid for 24 h, mock-transfected or transfected with poly(I:C) (5 μg) with Lipofectamine 2000, and untreated or treated with LPS (10 μg/ml) for 12 h before luciferase assays. (*P* value; poly(I:C): 0.0229, LPS: 0.0012) ($n = 3$ biological replicates). (**D**) Effects of DYRK4 on cytoplasmic poly(I:C)-induced or TLR3-mediated activation of the IFN-β promoter. HEK293T or HEK293T-TLR3 cells were transfected with the IFN-β reporter and control or DYRK4 plasmid for 24 h, mock-transfected or transfected with poly(I:C) (5 μg) with Lipofectamine 2000, and untreated or treated with poly(I:C) (25 μg/ml) for 12 h before luciferase assays. (*P* value; 293 T: 7.3×10^{-5}, 293T-TLR3: 2.4×10^{-5}) ($n = 3$ biological replicates). (**E**) Effects of DYRK4 knockdown on cytoplasmic poly(I:C)-induced or TLR3-mediated activation of the IFN-β promoter. HEK293T or HEK293T-TLR3 cells were transfected with the IFN-β reporter and control or DYRK4 RNAi plasmids for 36 h, mock-transfected or transfected with poly(I:C) (5 μg) with Lipofectamine 2000, and untreated or treated with poly(I:C) (25 μg/ml) for 12 h before luciferase assays. (*P* value; 293 T; #5: 0.00128, #6: 0.011, 293T-TLR3; #6: 0.0103) ($n = 3$ biological replicates). (**G**) A scheme for CRISPR/Cas9-mediated genome editing of the Dyrk4 gene locus. (**H**) Genotyping analysis of $Dyrk4^{+/+}$, $Dyrk4^{+/-}$ and $Dyrk4^{-/-}$ mice. (**I–K**) qPCR and immunoblot analysis of Dyrk4 mRNA or protein levels in $Dyrk4^{+/+}$ and $Dyrk4^{-/-}$ BMDMs, MLFs and spleens. (*P* value; I: 2.2×10^{-5}, L: 2.4×10^{-5}) ($n = 3$ biological replicates). (**L**) Cell counts in the spleen and thymus of $Dyrk4^{+/+}$ and $Dyrk4^{-/-}$ mice. (ns: not significant) ($n = 3$ biological replicates). (**M**) Flow cytometry analysis of the percentages of myeloid cells isolated from the spleens of $Dyrk4^{+/+}$ and $Dyrk4^{-/-}$ mice. Data information: Data are representative of three biological replicates and are shown as the means with SEMs (**A**, **C–F**, **I**, **L**); data in (**B**, **H**, **J**, **K**, **M**) are representative of two replicates. *$P < 0.05$, **$P < 0.01$, ***$P < 0.001$; two-tailed unpaired Student's *t* test.

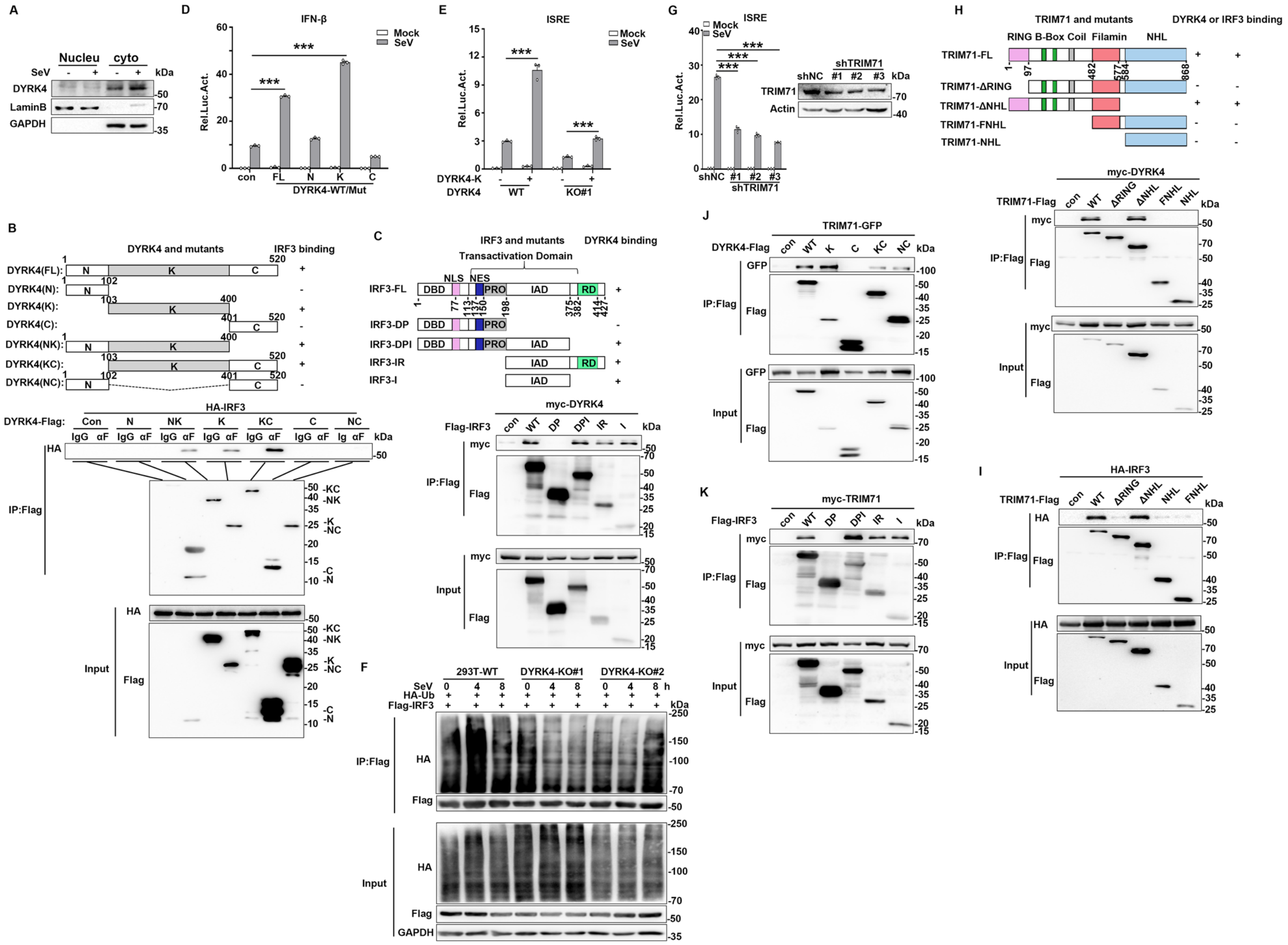

Figure EV3. Domain mapping of the interaction of DYRK4 with TRIM71 or IRF3.

Immunoblot analysis of DYRK4 in the cytoplasmic (Cyto) and nuclear (Nuc) fractions of HEK293T cells infected with SeV for 8 h. (**B**, **C**) Domain mapping of the interaction of DYRK4 with IRF3. HEK293T cells were transfected with the indicated truncations before coimmunoprecipitation and immunoblotting analysis with the indicated antibodies. Schematic representations of DYRK4 and IRF3 truncations are shown at the top. (DBD: DNA-binding structural domain, NLS: nuclear localization sequence, NES: nuclear export sequence, PRO: proline-rich region, IAD: IRF-related structural domain). (**D**) Effects of DYRK4 and its truncations on SeV-induced activation of the IFN-β promoter. HEK293T cells were transfected with the IFN-β reporter and the indicated plasmids for 24 h and then infected with SeV for 12 h before luciferase assays. (*P* value; FL: 3.7×10^{-7}, K: 2.5×10^{-7}) ($n = 3$ biological replicates). (**E**) Effects of DYRK4-KO on SeV-induced activation of the IFN-β promoter. DYRK4-KO and control HEK293T cells were transfected with DYRK4-K truncation plasmids for 24 h and then infected with SeV for 12 h before luciferase assays. (*P* value; WT: 0.00013, KO#1: 5.9×10^{-5}) ($n = 3$ biological replicates). (**F**) Effects of DYRK4 deficiency on the SeV-induced polyubiquitination of IRF3. DYRK4-KO and control HEK293T cells were transfected with Flag-IRF3 and HA-Ub for 24 h and then infected with SeV for the indicated times before immunoblotting and coimmunoprecipitation analysis with the indicated antibodies. (**G**) Effects of TRIM71 knockdown on SeV-induced activation of ISRE. HEK293T cells were transfected with the ISRE reporter and TRIM71-RNAi plasmids for 36 h and then infected with SeV for 12 h before luciferase assays were performed, and the knockdown efficiency of TRIM71 was determined via immunoblot analysis. (*P* value; #1: 9.6×10^{-6}, #2: 1.4×10^{-6}, #3: 2.9×10^{-7}) ($n = 3$ biological replicates). (**H–K**) Domain mapping of the interaction of TRIM71 with DYRK4 or IRF3. HEK293T cells were transfected with the indicated truncations for 24 h before immunoblotting and coimmunoprecipitation analysis with the indicated antibodies. Schematic representations of TRIM71 truncations are shown at the top. Data information: Data are representative of three biological replicates and are shown as the means with SEMs (**D**, **E**, **G**); data in (**A–C**, **F**, **H–K**) are representative of two replicates. ***$P < 0.001$, two-tailed unpaired Student's t test.

 © The Author(s)

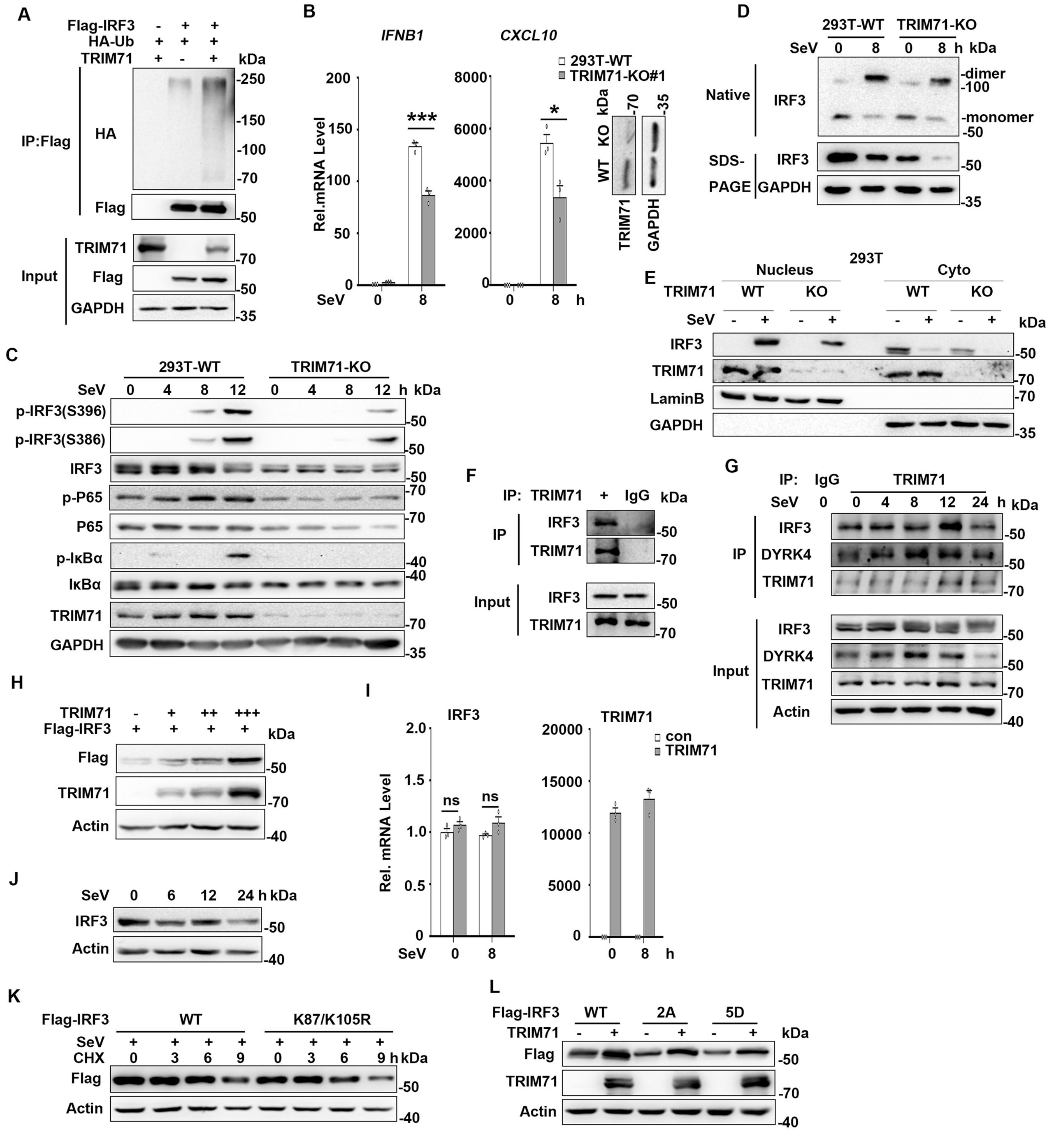
A
Flag-IRF3 - + +
HA-Ub + + +
TRIM71 + - +
kDa
IP:Flag
HA
Flag
Input
TRIM71
Flag
GAPDH
-250
-150
-100
-70
-50
-35
B
IFNB1
CXCL10
293T-WT
TRIM71-KO#1
Rel.mRNA Level
SeV 0 8 h
***
*
WT KO
TRIM71
GAPDH
C
293T-WT
TRIM71-KO
SeV 0 4 8 12 0 4 8 12 h kDa
p-IRF3(S396)
p-IRF3(S386)
IRF3
p-P65
P65
p-IκBα
IκBα
TRIM71
GAPDH
D
293T-WT TRIM71-KO
SeV 0 8 0 8 h kDa
Native
IRF3
-dimer
-100
-monomer
-50
SDS-PAGE
IRF3
GAPDH
E
293T
Nucleus
Cyto
TRIM71 WT KO WT KO
SeV - + - + - + - +
IRF3
TRIM71
LaminB
GAPDH
F
IP: TRIM71 + IgG kDa
IP
IRF3
TRIM71
Input
IRF3
TRIM71
G
IP: IgG TRIM71
SeV 0 0 4 8 12 24 h kDa
IP
IRF3
DYRK4
TRIM71
Input
IRF3
DYRK4
TRIM71
Actin
H
TRIM71 - + ++ +++
Flag-IRF3 + + + +
Flag
TRIM71
Actin
I
IRF3
TRIM71
con
TRIM71
Rel. mRNA Level
ns
ns
SeV 0 8
0 8 h
J
SeV 0 6 12 24 h kDa
IRF3
Actin
K
Flag-IRF3 WT K87/K105R
SeV + + + + + + + +
CHX 0 3 6 9 0 3 6 9 h kDa
Flag
Actin
L
Flag-IRF3 WT 2A 5D
TRIM71 - + - + - + kDa
Flag
TRIM71
Actin

Figure EV4. TRIM71 positively regulates RNA virus-triggered signaling by promoting IRF3 stability and activation.

(**A**) Effects of TRIM71 on the polyubiquitination of IRF3. HEK293T cells were transfected with Flag-IRF3 or HA-Ub and control or TRIM71 plasmids for 24 h, followed by immunoblotting and coimmunoprecipitation analysis with the indicated antibodies. (**B**) Effects of TRIM71 deficiency on the SeV-induced transcription of downstream genes. TRIM71-KO HEK293T clones were generated via the CRISPR-Cas9 method. TRIM71-KO and control HEK293T cells were infected with SeV for 8 h before qPCR analysis, and the TRIM71 knockout efficiency was determined via immunoblot analysis. (*P* value; *IFNB1*: 0.00078, *CXCL10*: 0.01818) ($n = 3$ biological replicates). (**C**) Effects of TRIM71 deficiency on SeV-induced phosphorylation of IRF3 (Ser386, Ser396), p65 and IκBα. TRIM71-KO and control HEK293T cells were infected with SeV for the indicated times before immunoblotting analysis with the indicated antibodies. (**D**) Effects of TRIM71 deficiency on SeV-induced dimerization of IRF3. TRIM71-KO and control HEK293T cells were infected with SeV for 8 h, and then, the cell lysates were separated via native PAGE and analyzed by immunoblotting with the indicated antibodies. (**E**) Effects of TRIM71 deficiency on the SeV-induced nuclear translocation of IRF3. TRIM71-KO and control HEK293T cells were infected with SeV for 8 h, and then, immunoblot analysis of IRF3 in the cytoplasmic (Cyto) and nuclear fractions was performed with the indicated antibodies. (**F**) Endogenous immunoprecipitation analysis of the interaction between TRIM71 and IRF3 in HEK293T cells. (**G**) Endogenous association of TRIM71 with IRF3 and DYRK4. HEK293T cells were infected with SeV for the indicated times. Immunoblotting and immunoprecipitation analysis were performed with the indicated antibodies. (**H**) Immunoblot analysis of the protein level of IRF3 in HEK293T cells transfected with different amounts of Flag-IRF3 or TRIM71 at different dosages. (**I**) qPCR analysis of the mRNA level of IRF3 in HEK293T cells transfected with control or TRIM71 for 24 h and then infected with SeV for 8 h. (ns: not significant) ($n = 3$ biological replicates). (**J**) Immunoblot analysis of the protein level of IRF3 in HEK293T cells infected with SeV for the indicated times. (**K**) Immunoblot analysis of the protein level of IRF3 in HEK293T cells transfected with Flag-IRF3 and its mutants for 20 h, preinfected with SeV for 12 h, and then treated with CHX for the indicated times. (**L**) Immunoblot analysis of the protein level of IRF3 in HEK293T cells transfected with Flag-IRF3 and its mutants (IRF3-2A, IRF3-5D) together with a control and TRIM71 for 24 h. Data information: Data are representative of three biological replicates and are shown as the means with SEMs (**B**, **I**); data in (**A**, **C**–**H**, **J**–**L**) are representative of two replicates. *$P < 0.05$, ***$P < 0.001$; two-tailed unpaired Student's *t* test.

 © The Author(s)

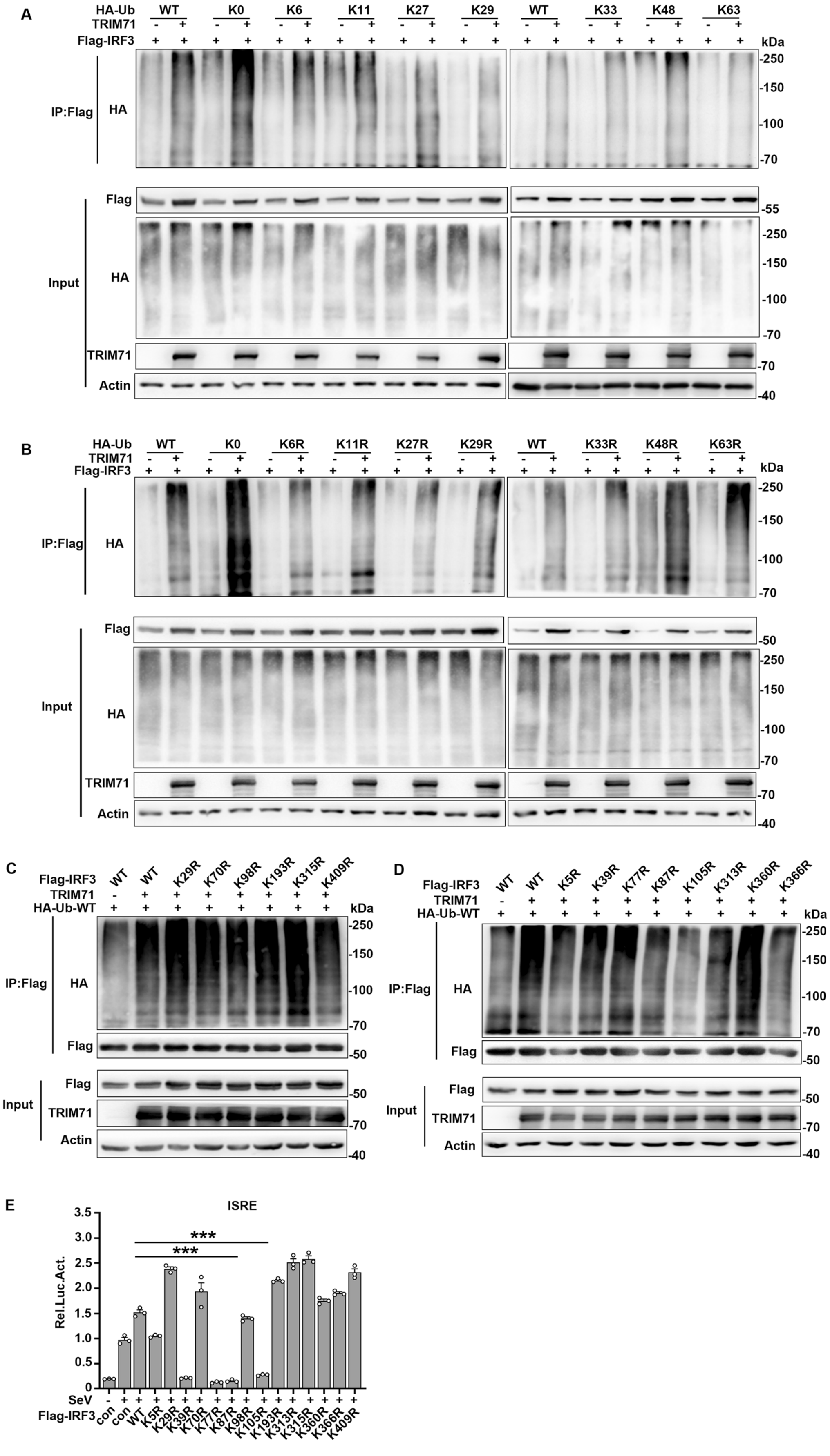
A
HA-Ub
WT
K0
K6
K11
K27
K29
K33
K48
K63
TRIM71
Flag-IRF3
kDa
IP:Flag
HA
Input
Flag
TRIM71
Actin
-250
-150
-100
-70
-55
-40
B
K6R
K11R
K27R
K29R
K33R
K48R
K63R
-50
C
WT
K29R
K70R
K98R
K193R
K315R
K409R
HA-Ub-WT
D
K5R
K39R
K77R
K87R
K105R
K313R
K360R
K366R
E
ISRE
Rel.Luc.Act.
***
SeV
con
3.5
3.0
2.5
2.0
1.5
1.0
0.5
0

◀ **Figure EV5. TRIM71 catalyzes the K0-linked ubiquitination of IRF3 at Lys87 and Lys105.**

(**A**, **B**) Coimmunoprecipitation analysis of the ubiquitination of IRF3 in HEK293T cells transfected with plasmids encoding Flag-IRF3, TRIM7, HA-Ub (WT) and its mutants for 24 h. (K48 cells were treated with MG132 for 4 h before sample collection). (**C**, **D**) Coimmunoprecipitation analysis of the ubiquitination of IRF3 in HEK293T cells transfected with plasmids encoding Flag-IRF3 or its mutants, TRIM71 and HA-Ub (WT), for 24 h. (**E**) Effects of IRF3 and its mutants on SeV-induced activation of ISRE. HEK293T cells were transfected with the ISRE reporter, Flag-IRF3, or its mutant plasmids for 24 h and then infected with SeV for 12 h before luciferase assays. (*P* value; K87R: 1.2×10^{-5}, K105R: 1.6×10^{-5}) ($n = 3$ biological replicates). Data information: Data are representative of three biological replicates and are shown as the mean with SEM (**E**); data in (**A**–**D**) are representative of two replicates. ****P* < 0.001, two-tailed unpaired Student's *t* test.

 © The Author(s)
